# Supplementary material for: C-to-N atom swapping and skeletal editing in indoles and benzofurans
Source: Nature. 2025 May 21;642(8066):92–8. doi: 10.1038/s41586-025-09019-6 (PMC12137124; doi:10.1038/s41586-025-09019-6)

nature portfolio

https://doi.org/10.1038/s41586-025-09019-6

Supplementary information

C-to-N atom swapping and skeletal editing in indoles and benzofurans

In the format provided by the authors and unedited

C-to-N atom swapping and skeletal editing in indoles and benzofurans

Zhe Wang[1], Pengwei Xu[1,2], Shu-Min Guo[1,2], Constantin G. Daniliuc[1], Armido Studer[1,*]

[1]Organisch-Chemisches Institut, Universität Münster, 48149 Münster, Germany

[2]These authors contributed equally to this work

*Email: studer@uni-muenster.de

Table of Contents

1. General information 2

2. Preparation of starting materials 3

3. Detailed optimization of reaction conditions 28

4. Reactions with oxime intermediates 31

5. General procedure 33

5.1 General procedure III: skeletal editing of indoles to give indazoles through radical pathway (A) 33

5.2 General procedure IV: skeletal editing of indoles to give benzimidazoles through radical pathway (A) 33

5.3 General procedure V: skeletal editing of benzofurans to give benzisoxazoles through radical pathway (A) 33

5.4 General procedure VI: skeletal editing of benzofurans to give benzoxazoles through radical pathway (A) 34

5.5 General procedure VII: skeletal editing of indoles to give indazoles through ionic pathway (B) 34

5.6 General procedure VIII: skeletal editing of indoles to give benzimidazoles through ionic pathway (B) 35

5.7 General procedure IX: skeletal editing of benzofurans to give benzisoxazoles through ionic pathway (B) 35

5.8 General procedure X: skeletal editing of benzofurans to give benzoxazoles through ionic pathway (B) 35

6. Spectral data of products 37

7. X-ray crystal structure analysis of **2aa**, **5r** and **6s** 92

8. References 95

9. NMR spectra of products 98

1. General information

All air- or moisture-sensitive reactions and manipulations were carried out using standard Schlenk techniques under an argon atmosphere and magnetic induced stirring. Solvents were removed by rotary evaporation at 40 °C at an appropriate pressure.

Reagents and solvents: Reagents were purchased at the highest commercial grade from *ABCR*, *BLD Pharm*, *Sigma-Aldrich* or *TCI* and were used as received. Commercial solvents were used without further purification. All deuterated solvents were purchased from *Sigma-Aldrich* or *Euriso-Top*.

Chromatography: All reactions were monitored by thin layer chromatography (**TLC**) using *silica gel 60* F_{254} plates (*Merck*). The spots were visualized using UV light (254 nm). Column chromatography was performed on silica gel (40-63 µm) (*Merck* or *VWR*) using pressurized air. Reversed phase medium pressure liquid chromatography (**RP-MPLC**) was performed on a *C-850 FlashPrep device* (*Büchi*) using *FlashPure EcoFlex* C18 50 µm flash cartridges (spherical, 4 g) (*Büchi*) as the stationary phase. Water (Milli-Q grade) and acetonitrile (HPLC grade) were used as the mobile phase in gradient elution modes. Detection was carried out using UV absorption (λ = 210 nm, 230 nm, 254 nm, 320 nm).

Spectroscopy and instruments: NMR spectra of **^{1}H** (300 MHz, 400 MHz and 600 MHz), **^{13}C** (75 MHz, 100 MHz and 150 MHz) and **^{19}F** (282 MHz, 376 MHz and 564 MHz) spectra were measured on a *DPX 300 (Bruker), DD2 500 (Agilent) or DD2 600 (Agilent)* spectrometer. Chemical shifts are reported in parts per million (ppm) and the spectra were referenced using the residual $CDCl_3$ signals (δ_H = 7.26 ppm, δ_C = 77.16 ppm). Peak multiplicities are defined as s (singlet), brs (broad singlet), d (doublet), t (triplet), q (quartet) and m (multiplet). High-resolution mass spectroscopic measurements (**HRMS**) were conducted on a *Bruker MicroTof* device with electrospray ionization. The computer software UMC (Universal Mass Calculator) was used to (e.g. simulate isotope patterns, evaluate the degree of deuteration/labelling, evaluate elemental compositions). UMC Version 3.14.0.101, Dr. Matthias C. Letzel, WWU Münster, Org.-Chem. Institut, Germany. Melting points (**Mp**) were determined using a M-560 melting point apparatus (Büchi) and are uncorrected. **IR** spectra were measured on a FTIR-4600LE FTIR spectrometer (Jasco). IR signals are categorized as strong (s), medium (m) and weak (w). Absorption maxima are reported in cm^{-1}.

X-Ray diffraction: Data sets for compounds **2aa**, **5r** and **6s** were collected with a Bruker D8 Venture Photon III Diffractometer. Programs used: data collection: *APEX4* Version 2021.4-0[1] (Bruker AXS Inc., **2021**); cell refinement: *SAINT* Version 8.40B (Bruker AXS Inc., **2021**); data reduction: *SAINT* Version 8.40B (Bruker AXS Inc., **2021**); absorption correction, *SADABS* Version 2016/2 (Bruker AXS Inc., **2021**); structure solution *SHELXT*-Version 2018-3[2] (Sheldrick, G. M. *Acta Cryst.,* **2015**, *A71*, 3-8); structure refinement *SHELXL*- Version 2018-3[3] (Sheldrick, G. M. *Acta Cryst.,* **2015**, *C71* (1), 3-8) and graphics, *XP*[4] (Version 5.1, Bruker AXS Inc., Madison, Wisconsin, USA, **1998**). *R*-values are given for observed reflections, and wR^2 values are given for all reflections.

2. Preparation of starting materials

➢ **Synthesis of indoles and benzofurans**

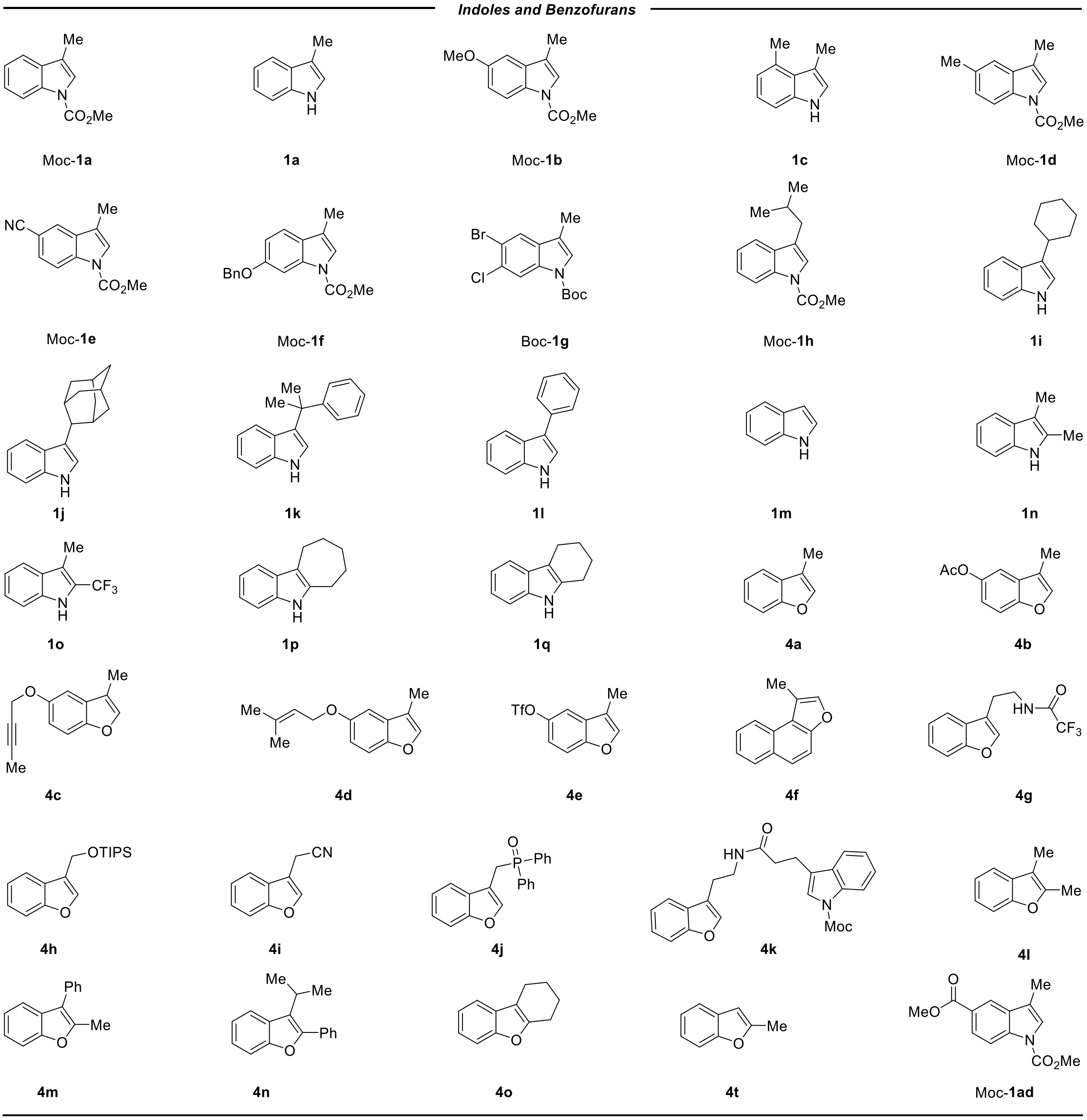

Indoles **1a, 1m-1n, 1p-1q** and benzofurans **4a**, **4i**, **4l** as well as **4t** were purchased and used as received. Indoles Moc-**1a**, Moc-**1b**, **1c**, Moc-**1d** to Moc-**1h**, **1i-1l**, Moc-**1ad** and benzofurans **4b-4h**, **4j-4k, 4m-4o** were prepared according to literature procedures.[5-20]

General procedure I

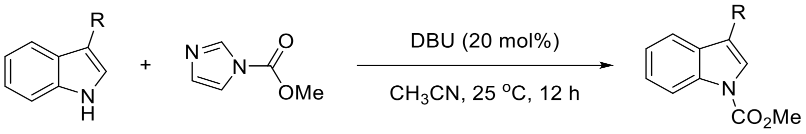

The corresponding indole (1.0 equiv.) was dissolved in anhydrous acetonitrile and then imidazole carbamates (1.2 equiv.) was added at room temperature followed by DBU (20 mol%). The reaction mixture was stirred at room temperature for 16 h. After completion, the solvent was removed with a rotary evaporator under reduced pressure and the residue was subjected to flash column chromatography over silica gel to give the corresponding Moc-protected indoles. The methyl imidazole carbamate was prepared according to a literature procedure and analytic data are in agreements with the reported literature data.[5]

General procedure II:

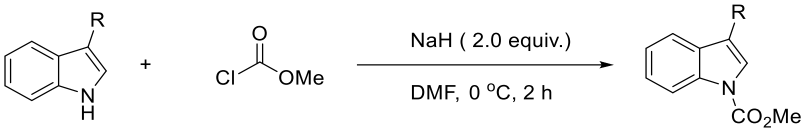

The corresponding indole (1.0 equiv.) was dissolved in anhydrous DMF and sodium hydride (2.0 equiv., 60% dispersion in mineral oil) was added in portions at 0 °C using an ice-water bath. Then, the mixture was stirred for 30 min at 0 °C. Afterwards, methyl chloroformate (2.0 equiv.) was added dropwise and the reaction mixture was stirred for another 2 h. The reaction was carefully quenched with sat. NH_4Cl and the mixture was extracted with EtOAc three times. The combined organic extracts were washed with brine, dried over anhydrous Na_2SO_4, filtered and the solvent was removed with a rotary evaporator under reduced pressure. The residue was subjected to flash column chromatography over silica gel to give the corresponding Moc-protected indole.

Methyl 3-methyl-1*H*-indole-1-carboxylate Moc-**1a**

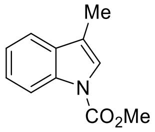

Moc-**1a** was prepared according to the general procedure I with 3-methyl-1*H*-indole **1a** (1.32 g, 10.0 mmol). After purification by flash chromatography (*n*-pentane/EtOAc = 50/1), the desired compound Moc-**1a** was obtained as a colourless oil (1.67 g, 89% yield). The analytical data are in agreement with the reported literature data.[6]

^{1}H NMR (300 MHz, $CDCl_3$) δ 8.14 (s, 1H), 7.53 – 7.48 (m, 1H), 7.37 (d, *J* = 1.6 Hz, 1H), 7.33 (dd, *J* = 8.2, 1.4 Hz, 1H), 7.30 – 7.23 (m, 1H), 4.02 (s, 3H), 2.27 (d, *J* = 1.4 Hz, 3H).

^{13}C NMR (76 MHz, $CDCl_3$) δ 151.6, 135.5, 131.5, 124.6, 122.8, 122.5, 119.1, 117.4, 115.2, 53.7, 9.8.

Methyl 5-methoxy-3-methyl-1*H*-indole-1-carboxylate Moc-**1b**

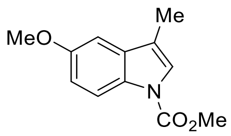

Moc-**1b** was prepared according to the general procedure I with 5-methoxy-3-methyl-1*H*-indole (0.48 g, 3.0 mmol). After purification by flash chromatography (*n*-pentane/EtOAc = 50/1), the desired compound Moc-**1b** was obtained

as a colourless solid (622 mg, 96% yield). The analytical data are in agreement with the reported literature data.[6]

^{1}H NMR (300 MHz, $CDCl_3$) δ 8.02 (s, 1H), 7.34 (s, 1H), 6.98 – 6.89 (m, 2H), 4.00 (s, 3H), 3.88 (s, 3H), 2.24 (d, *J* = 1.3 Hz, 3H).

^{13}C NMR (76 MHz, $CDCl_3$) δ 156.2, 132.2, 123.2, 112.0, 111.6, 117.2, 116.0, 113.0, 102.1, 55.9, 53.6, 9.8.

3,4-Dimethyl-1*H*-indole 1c

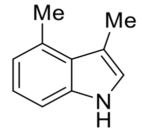

1c was prepared according to a literature procedure[7] with 4-methyl-1*H*-indole-3-carbaldehyde (0.80 g, 5.0 mmol). After purification by flash chromatography (*n*-pentane/EtOAc = 20/1), the desired compound **1c** was obtained as a brown solid (722 mg, 91% yield). The analytical data are in agreement with the reported literature data.[7]

^{1}H NMR (300 MHz, $CDCl_3$) δ 7.80 (s, 1H), 7.18 (d, *J* = 8.1 Hz, 1H), 7.11 – 7.01 (m, 1H), 6.91 (dd, *J* = 2.4, 1.2 Hz, 1H), 6.86 – 6.79 (m, 1H), 2.75 (d, *J* = 1.2 Hz, 3H), 2.54 (d, *J* = 1.2 Hz, 3H).

^{13}C NMR (76 MHz, $CDCl_3$) δ 136.9, 131.4, 126.7, 122.1, 121.9, 120.7, 112.7, 109.1, 20.2, 13.2.

Methyl 3,5-dimethyl-1*H*-indole-1-carboxylate Moc-**1d**

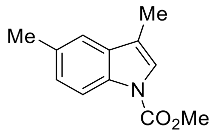

Moc-**1d** was prepared according to the general procedure II with 3,5-dimethyl-1*H*-indole (0.73 g, 5.0 mmol). After purification by flash chromatography (*n*-pentane/EtOAc = 50/1), the desired compound Moc-**1d** was obtained as a white solid (699.3 mg, 69% yield). The analytical data are in agreement with the reported literature data.[8] 3,5-dimethyl-1*H*-indole was synthesized according to a literature procedure with 5-methyl-1*H*-indole-3-carbaldehyde and lithium aluminium hydride.[9]

^{1}H NMR (400 MHz, $CDCl_3$) δ 8.01 (s, 1H), 7.33 (s, 1H), 7.29 (s, 1H), 7.15 (d, *J* = 8.4 Hz, 1H), 4.01 (s, 3H), 2.47 (s, 3H), 2.25 (d, *J* = 1.3 Hz, 3H).

^{13}C NMR (76 MHz, $CDCl_3$) δ 151.7, 133.8, 132.3, 131.7, 125.9, 122.5, 119.1, 117.2, 114.8, 53.6, 21.5, 9.8.

Methyl 5-cyano-3-methyl-1*H*-indole-1-carboxylate Moc-**1e**

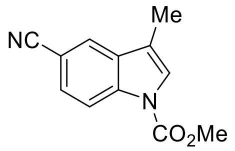

Moc-**1e** was prepared according to the general procedure I with 3-methyl-1*H*-indole-5-carbonitrile (0.32 g, 2.0 mmol). After purification by flash chromatography (*n*-pentane/EtOAc = 25/1), the desired compound Moc-**1e** was obtained as a white solid (339 mg, 80% yield).

TLC: 0.20 (*n*-pentane/EtOAc = 25/1).

^{1}H NMR (300 MHz, $CDCl_3$) δ 8.24 (d, *J* = 8.6 Hz, 1H), 7.83 (dd, *J* = 1.7, 0.7 Hz, 1H), 7.58 (dd, *J* = 8.6, 1.6 Hz, 1H), 7.48 (q, *J* = 1.4 Hz, 1H), 4.05 (s, 3H), 2.29 (d, *J* = 1.3 Hz, 3H).

^{13}C NMR (101 MHz, $CDCl_3$) δ 151.1, 137.5, 131.6, 127.9, 124.6, 124.1, 119.9, 117.2, 116.1, 106.3, 54.3, 9.6.

HRMS (ESI) m/z: [M+Na$^+$] Calculated for $C_{12}H_{10}N_2O_2Na$: 237.06345; Found 237.06329.

IR (neat): 2220 (w), 1275 (m), 1469 (m), 1437 (s), 1387 (m), 1371 (s), 1262 (s), 1227 (m), 1199 (m), 1179 (m), 1083 (s), 1040 (m), 904 (m), 806 (s), 764 (s), 631 (m).

Mp: 146-147 °C.

Methyl 6-(benzyloxy)-3-methyl-1*H*-indole-1-carboxylate Moc-**1f**

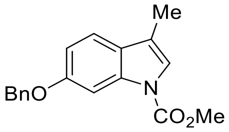

Moc-**1f** was prepared according to the general procedure II with 6-(benzyloxy)-3-methyl-1*H*-indole (0.48 g, 2.0 mmol). After purification by flash chromatography (*n*-pentane/EtOAc = 50/1), the desired compound Moc-**1f** was obtained as a yellow solid (421 mg, 72% yield). 6-(Benzyloxy)-3-methyl-1*H*-indole was synthesized according to a literature procedure with 6-(benzyloxy)-1*H*-indole-3-carbaldehyde and lithium aluminium hydride.[9]

TLC: 0.40 (*n*-pentane/EtOAc = 25/1).

^{1}H NMR (400 MHz, $CDCl_3$) δ 7.86 (s, 1H), 7.50 (d, *J* = 6.9 Hz, 2H), 7.44 – 7.31 (m, 4H), 7.28 – 7.25 (m, 1H), 7.01 – 6.95 (m, 1H), 5.16 (s, 2H), 4.00 (s, 3H), 2.24 (d, *J* = 1.2 Hz, 3H).

^{13}C NMR (101 MHz, $CDCl_3$) δ 157.3, 151.7, 137.3, 136.6, 128.7, 128.1, 127.7, 125.6, 121.3, 119.6, 117.3, 112.5, 101.0, 70.7, 53.7, 9.8.

HRMS (ESI) m/z: [M+Na$^+$] Calculated for $C_{18}H_{17}NO_3Na$: 318.11006; Found 318.11010.

IR (neat): 1733 (m), 1456 (m), 1437 (s), 1394 (m), 1374 (m), 1259 (s), 1211 (s), 1093 (m), 1031 (m), 1003 (s), 954 (m), 899 (m), 847 (m), 810 (s), 756 (s).

Mp: 77-78 °C.

***tert*-Butyl 5-bromo-6-chloro-3-methyl-1*H*-indole-1-carboxylate** Boc-**1g**

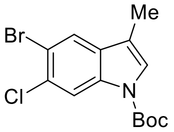

Boc-**1g** was prepared according to literature reported procedures[6,9] from *tert*-butyl 5-bromo-6-chloro-3-formyl-1*H*-indole-1-carboxylate (0.90 g, 3.48 mmol) in two steps. After purification by flash chromatography (*n*-pentane), the desired compound Boc-**1g** was obtained as a white solid (920 mg, 78% yield in two steps).

TLC: 0.50 (*n*-pentane/EtOAc = 100/1).

^{1}H NMR (400 MHz, $CDCl_3$, containing tautomers) δ 8.27 – 8.15 (m, 1H), 7.79 – 7.19 (m, 2H), 2.26 – 2.15 (m, 3H),

1.66 (s, 9H).

^{13}C NMR (101 MHz, $CDCl_3$, containing tautomers) δ 149.3, 131.6, 130.4, 130.0, 124.5, 123.5, 123.4, 122.9, 119.8, 117.0, 116.3, 115.9, 115.6, 115.6, 84.3, 83.9, 28.3, 28.3, 9.7, 9.6.

HRMS (ESI) m/z: [M+Na$^+$] Calculated for $C_{14}H_{15}NO_2BrClNa$: 365.98669; found: 365.98672.

IR (neat): 1725 (s), 1456 (s), 1447 (s), 1387 (s), 1363 (m), 1273 (w), 1157 (s), 1115 (w), 1089 (s), 1078 (s), 877 (w), 851 (w), 791 (s), 765 (s).

Mp: 98-100 °C.

Methyl 3-isobutyl-1*H*-indole-1-carboxylate Moc-**1h**

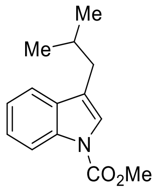

Moc-**1h** was prepared according to the general procedure II with 3-isobutyl-1*H*-indole (0.69 g, 4.0 mmol). After purification by flash chromatography (*n*-pentane/EtOAc = 50/1), the desired compound Moc-**1h** was obtained as a yellow oil (878 mg, 95% yield). 3-Isobutyl-1*H*-indole was synthesized according to a literature procedure.[10]

TLC: 0.64 (*n*-pentane/EtOAc = 25/1).

^{1}H NMR (400 MHz, $CDCl_3$) δ 8.17 (s, 1H), 7.56 – 7.51 (m, 1H), 7.39 – 7.30 (m, 2H), 7.28 – 7.20 (m, 1H), 4.03 (d, J = 1.5 Hz, 3H), 2.56 (dt, J = 7.0, 1.2 Hz, 2H), 2.09 – 1.94 (m, 1H), 0.97 (dd, J = 6.6, 1.5 Hz, 6H).

^{13}C NMR (101 MHz, $CDCl_3$) δ 151.7, 135.7, 131.3, 124.5, 122.7, 121.2, 119.4, 115.3, 53.7, 34.4, 28.5, 22.8. (one carbon was not detected).

HRMS (ESI) m/z: [M+Na$^+$] Calculated for $C_{14}H_{17}NO_2Na$: 254.11515; Found 254.11537.

IR (neat): 1732 (s), 1455 (s), 1440 (s), 1363 (s), 1250 (s), 1218 (m), 1153 (w), 1101 (m), 1075 (s), 1020 (w), 792 (w), 763 (m), 742 (s).

3-Cyclohexyl-1*H*-indole 1i

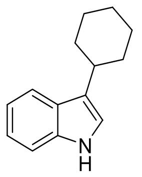

1i was prepared according to a literature procedure[11] with 1*H*-indole (1.17 g, 10.0 mmol) and cyclohexanone (1.12 mL, 11.0 mmol). After purification by flash chromatography (*n*-pentane/EtOAc = 20/1), the desired compound **1i** was obtained as a brown solid (1.60 g, 81% yield). The analytic data are in agreement with the reported literature data.[11]

^{1}H NMR (300 MHz, $CDCl_3$) δ 7.90 – 7.85 (m, 1H), 7.72 – 7.65 (m, 1H), 7.39 – 7.31 (m, 1H), 7.23 – 7.16 (m, 1H), 7.16 – 7.08 (m, 1H), 6.95 (dd, J = 2.4, 0.9 Hz, 1H), 2.93 – 2.78 (m, 1H), 2.21 – 2.04 (m, 2H), 1.92 – 1.74 (m, 3H), 1.58 – 1.40 (m, 4H), 1.38 – 1.22 (m, 1H).

^{13}C NMR (76 MHz, $CDCl_3$) δ 136.5, 126.9, 123.4, 121.9, 119.49, 119.47, 119.1, 111.2, 35.6, 34.2, 27.1, 26.7.

3-(Adamantan-2-yl)-1*H*-indole 1j

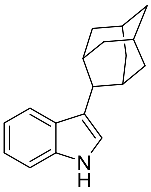

1j was prepared according to a literature procedure[11] with 1*H*-indole (1.17 g, 10.0 mmol) and 2-adamantanone (1.65 g, 11.0 mmol). After purification by flash chromatography (*n*-pentane/EtOAc = 20/1), the desired compound **1j** was obtained as a brown solid (1.75 g, 70% yield). The analytic data are in agreement with the reported literature data.[11]

^{1}H NMR (300 MHz, $CDCl_3$) δ 7.95 (s, 1H), 7.63 (dd, *J* = 7.9, 1.2 Hz, 1H), 7.36 (d, *J* = 8.0 Hz, 1H), 7.22 – 7.03 (m, 3H), 3.38 (s, 1H), 2.37 (s, 2H), 2.15 – 1.92 (m, 8H), 1.89 – 1.77 (m, 2H), 1.70 – 1.56 (m, 2H).

^{13}C NMR (76 MHz, $CDCl_3$) δ 136.3, 127.6, 121.8, 121.3, 120.4, 119.8, 119.0, 111.1, 42.6, 39.6, 38.3, 32.8, 32.4, 28.4, 28.2.

3-(2-Phenylpropan-2-yl)-1*H*-indole 1k

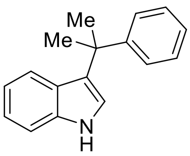

1k was prepared according to a literature procedure[12] with 1*H*-indole (0.56 g, 5.0 mmol) and prop-1-en-2-ylbenzene (1.47 mL, 10.0 mmol). After purification by flash chromatography (*n*-pentane/EtOAc = 50/1), the desired compound **1k** was obtained as a yellow oil (0.83 g, 75% yield). The analytic data are in agreement with the reported literature data.[12]

^{1}H NMR (300 MHz, $CDCl_3$) δ 7.94 (s, 1H), 7.39 – 7.33 (m, 3H), 7.29 – 7.22 (m, 2H), 7.19 – 7.15 (m, 1H), 7.13 (d, *J* = 2.4 Hz, 1H), 7.11 – 7.07 (m, 1H), 7.06 – 7.02 (m, 1H), 6.92 – 6.83 (m, 1H), 1.78 (s, 6H).

^{13}C NMR (76 MHz, $CDCl_3$) δ 150.0, 137.2, 128.1, 126.8, 126.6, 126.5, 125.7, 121.8, 121.4, 120.7, 119.0, 111.2, 39.0, 30.8.

3-Phenyl-1*H*-indole 1l

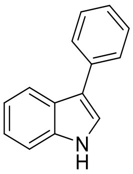

1l was prepared according to a literature procedure[11] with 2-phenylacetaldehyde (2.89 mL, 20.0 mmol) and phenylhydrazine hydrochloride (2.89g, 20.0 mmol). After purification by flash chromatography (*n*-pentane/EtOAc = 20/1), the desired compound **1l** was obtained as a brown solid (1.58 g, 41% yield). The analytical data are in agreement with the reported literature data.[11]

^{1}H NMR (300 MHz, $CDCl_3$) δ 8.22 (s, 1H), 8.01 – 7.94 (m, 1H), 7.72 – 7.66 (m, 2H), 7.51 – 7.42 (m, 3H), 7.38 (d, *J* = 2.5 Hz, 1H), 7.34 – 7.27 (m, 2H), 7.25 – 7.17 (m, 1H).

^{13}C NMR (76 MHz, $CDCl_3$) δ 136.8, 135.7, 128.9, 127.6, 126.1, 125.9, 122.6, 121.9, 120.5, 120.0, 118.5, 111.5.

3-Methyl-2-(trifluoromethyl)-1*H*-indole 1o

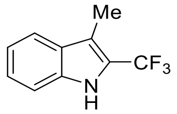

1o was prepared according to a literature procedure[13] with 3-methylindole (0.66g, 5.0 mmol). After purification by flash chromatography (*n*-pentane/EtOAc = 50/1), the desired compound **1o** was obtained as a brown solid (0.42 g, 43% yield). The analytical data are in agreement with the reported literature data.[13]

^{1}H NMR (300 MHz, $CDCl_3$) δ 8.15 (s, 1H), 7.65 (dd, *J* = 8.0, 1.1 Hz, 1H), 7.41 – 7.36 (m, 1H), 7.36 – 7.29 (m, 1H), 7.20 (ddd, *J* = 8.0, 6.7, 1.4 Hz, 1H), 2.46 (q, *J* = 1.9 Hz, 3H).

^{13}C NMR (76 MHz, $CDCl_3$) δ 135.3, 128.2, 124.9, 122.9 (q, *J* = 268.9 Hz), 121.7 (q, *J* = 37.3 Hz), 120.5, 120.2, 114.2 (q, *J* = 2.9 Hz), 111.7, 8.5.

^{19}F NMR (282 MHz, $CDCl_3$) δ -58.7.

3-Methylbenzofuran-5-yl acetate 4b

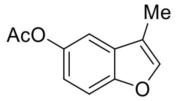

To a solution of 3-methylbenzofuran-5-ol (296 mg, 2.00 mmol, 1.0 equiv.) and acetic anhydride (0.38 mL, 4.0 mmol, 2.0 equiv.) in 10 mL DCM, triethylamine (0.56 mL, 4.0 mmol, 2.0 equiv.) was added. The reaction mixture was stirred at room temperature overnight. After purification by flash chromatography (*n*-pentane/EtOAc = 50/1), the desired compound **4b** was obtained as a colourless solid (341 mg, 90% yield).

TLC: 0.34 (*n*-pentane/EtOAc = 50/1).

^{1}H NMR (300 MHz, $CDCl_3$) δ 7.45 – 7.40 (m, 2H), 7.23 (d, *J* = 2.4 Hz, 1H), 6.98 (dd, *J* = 8.8, 2.4 Hz, 1H), 2.33 (d, *J* = 0.7 Hz, 3H), 2.21 (dd, *J* = 1.4, 0.6 Hz, 3H).

^{13}C NMR (76 MHz, $CDCl_3$) δ 170.3, 153.0, 146.2, 142.9, 129.9, 117.9, 116.1, 112.2, 111.9, 21.3, 8.0.

HRMS (ESI) m/z: [M+Na$^+$] Calculated for $C_{11}H_{10}O_3Na$: 213.05222; Found 213.05219.

IR (neat): 1747 (m), 1455 (m), 1369 (w), 1210 (s), 1166 (s), 1087 (s), 1014 (m), 935 (m), 899 (s), 830 (m), 796 (s), 479 (w).

Mp: 48-49 °C.

5-(But-2-yn-1-yloxy)-3-methylbenzofuran 4c

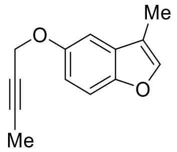

3-Methylbenzofuran-5-ol (350 mg, 2.50 mmol, 1.0 equiv.) was dissolved in acetone (4.5 mL). Then 1-bromobut-2-yne (0.24 mL, 2.75 mmol, 1.1 equiv.) and $CsCO_3$ (896 mg, 2.75 mmol, 1.1 equiv.) were added. The reaction mixture was stirred at room temperature and monitored by TLC. After completion, brine was added and the mixture was extracted with EtOAc three times. The combined organic extracts were washed with brine, dried over anhydrous Na_2SO_4, filtered and the solvent was removed with a rotary evaporator under reduced pressure. The residue was subjected to flash column chromatography (*n*-pentane/EtOAc = 50/1) to give **4c** as a colourless oil (436 mg, 87%).

TLC: 0.64 (*n*-pentane/EtOAc = 50/1).

^{1}H NMR (300 MHz, $CDCl_3$) δ 7.40 – 7.38 (m, 1H), 7.35 (d, *J* = 8.9 Hz, 1H), 7.04 (d, *J* = 2.6 Hz, 1H), 6.95 (dd, *J* = 8.9, 2.6 Hz, 1H), 4.73 – 4.66 (m, 2H), 2.22 (t, *J* = 1.1 Hz, 3H), 1.94 – 1.83 (m, 3H).

^{13}C NMR (76 MHz, $CDCl_3$) δ 154.1, 150.7, 142.4, 129.6, 115.9, 113.6, 111.9, 103.9, 83.7, 74.5, 57.6, 8.1, 3.9.

HRMS (ESI) m/z: [M+Na$^+$] Calculated for $C_{13}H_{12}O_2Na$: 223.07295; Found 223.07296.

IR (neat): 1472 (s), 1454 (s), 1261 (w), 1203 (m), 1178 (s), 1091 (m), 1061 (w), 1008 (s), 831 (w), 784 (s), 753 (w).

3-Methyl-5-((3-methylbut-2-en-1-yl)oxy)benzofuran 4d

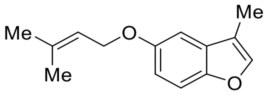

3-Methylbenzofuran-5-ol (350 mg, 2.50 mmol, 1.0 equiv.) was dissolved in acetone (4.5 mL). Then 1-bromo-3-methylbut-2-ene (0.24 mL, 2.75 mmol, 1.1 equiv.) and $CsCO_3$ (896 mg, 2.75 mmol, 1.1 equiv.) were added. The reaction mixture was stirred at room temperature and monitored by TLC. After completion, brine was added and the mixture was extracted by EtOAc three times. The combined organic extracts were washed with brine, dried over anhydrous Na_2SO_4, filtered and the solvent was removed with a rotary evaporator under reduced pressure. The residue was subjected to flash column chromatography (*n*-pentane/EtOAc = 50/1) to give **4d** as a colourless oil (489 mg, 91%).

TLC: 0.74 (*n*-pentane/EtOAc = 50/1).

^{1}H NMR (300 MHz, $CDCl_3$) δ 7.38 (d, *J* = 1.4 Hz, 1H), 7.33 (d, *J* = 8.8 Hz, 1H), 6.99 (d, *J* = 2.6 Hz, 1H), 6.91 (dd, *J* = 8.9, 2.5 Hz, 1H), 5.59 – 5.48 (m, 1H), 4.55 (d, *J* = 6.8 Hz, 2H), 2.22 (d, *J* = 1.3 Hz, 3H), 1.81 (d, *J* = 1.5 Hz, 6H).

^{13}C NMR (76 MHz, $CDCl_3$) δ 155.1, 150.3, 142.3, 138.2, 129.6, 120.1, 115.8, 113.6, 111.8, 103.2, 65.8, 26.0, 18.4, 8.1.

HRMS (ESI) m/z: [M+Na$^+$] Calculated for $C_{14}H_{16}O_2Na$. 239.10425; Found 239.10413.

IR (neat): 1472 (m), 1454 (s), 1260 (w), 1214 (m), 1179 (s), 1092 (m), 1061 (w), 1004 (m), 831 (w), 781 (s).

3-Methylbenzofuran-5-yl trifluoromethanesulfonate 4e

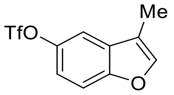

To a vial with a magnetic stirring bar were charged 3-methylbenzofuran-5-ol (361 mg, 2.50 mmol, 1.0 equiv.), *N*-(5-chloropyridin-2-yl)-1,1,1-trifluoro-*N*-((trifluoromethyl)sulfonyl)methanesulfonamide (906.6 mg, 2.75 mmol, 1.1 equiv.) were added. The solids were then suspended in CH_2Cl_2 (3.6 mL). To the reaction was added iPr_2EtN (1.75 mL, 10.0 mmol, 4.0 equiv.). The reaction was stirred at room temperature, and the progress was monitored by TLC. After completion of the reaction, the mixture was concentrated, and the resulting residue was purified by flash column chromatography (*n*-pentane/EtOAc = 50/1) to give **4e** as a colourless oil (230 mg, 33%).

TLC: 0.72 (*n*-pentane/EtOAc = 50/1).

^{1}H NMR (400 MHz, $CDCl_3$) δ 7.51 (d, *J* = 1.4 Hz, 1H), 7.48 (d, *J* = 8.9 Hz, 1H), 7.42 (d, *J* = 2.6 Hz, 1H), 7.18 (dd, *J* = 8.9, 2.6 Hz, 1H), 2.25 (d, *J* = 1.3 Hz, 3H).

^{13}C NMR (101 MHz, $CDCl_3$) δ 154.0, 145.2, 144.1, 130.4, 119.5 (q, *J* = 320.5 Hz), 117.4, 116.3, 112.7, 112.5, 7.9.

^{19}F NMR (376 MHz, $CDCl_3$) δ -72.7.

HRMS (EI) m/z: [M^+] Calculated for $C_{10}H_7O_4SF_3$ 280.00117; Found 280.00139.

IR (neat): 1455 (w), 1419 (s), 1243 (w), 1206 (s), 1138 (s), 1112 (w), 1090 (w), 924 (s), 872 (m), 857 (s), 797 (w), 606 (m), 507 (w).

1-Methylnaphtho[2,1-*b*]furan 4f

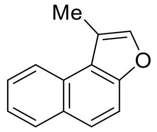

4f was prepared according to a literature[14] procedure with 1-(naphthalen-2-yloxy)propan-2-one (0.60 g, 3.0 mmol, 1.0 equiv.) and titanium tetrachloride (1.14 g, 6.00 mmol, 2.0 equiv.) in dry DCM (30 mL). After purification by flash chromatography (*n*-pentane/EtOAc = 50/1), the desired compound **4f** was obtained as a colourless oil (327 mg, 60% yield). The analytic data are in agreement with the reported literature data.[14]

^{1}H NMR (300 MHz, $CDCl_3$) δ 8.39 (d, J = 8.3 Hz, 1H), 7.97 (dd, J = 8.1, 1.4 Hz, 1H), 7.72 (d, J = 8.9 Hz, 1H), 7.66 – 7.61 (m, 1H), 7.60 – 7.44 (m, 3H), 2.64 (d, J = 1.3 Hz, 3H).

^{13}C NMR (76 MHz, $CDCl_3$) δ 153.5, 141.3, 130.7, 129.2, 129.1, 126.3, 125.4, 124.2, 123.2, 122.2, 117.7, 112.8, 11.5.

***N*-(2-(benzofuran-3-yl)ethyl)-2,2,2-trifluoroacetamide 4g**

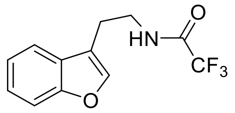

4g was prepared according to a literature[15] procedure with 2-(benzofuran-3-yl)acetonitrile (0.79 g, 5.0 mmol, 1.0 equiv.) and $LiAlH_4$ (250 mg, 6.60 mmol, 1.3 equiv.) in dry Et_2O (10 mL). Then the crude amine was protected using TFAA (0.85 mL, 6.0 mmol, 1.2 equiv.) in dry DCM (30 mL) at 0 °C for 5 h. After purification by flash chromatography (*n*-pentane/EtOAc = 10/1), the desired compound **4g** was obtained as a brown solid (495 mg, 39% yield).

TLC: 0.74 (*n*-pentane/EtOAc = 10/3).

^{1}H NMR (400 MHz, $CDCl_3$) δ 7.56 (dd, J = 7.7, 1.4 Hz, 1H), 7.52 – 7.46 (m, 2H), 7.37 – 7.31 (m, 1H), 7.30 – 7.24 (m, 1H), 6.50 (s, 1H), 3.84 – 3.52 (m, 2H), 3.15 – 2.81 (m, 2H).

^{13}C NMR (101 MHz, $CDCl_3$) δ 157.6 (q, J = 36.6 Hz), 155.7, 142.2, 127.6, 125.0, 123.0, 119.4, 116.5, 116.0 (q, J = 288.0 Hz), 112.0, 39.6, 23.5.

^{19}F NMR (376 MHz, $CDCl_3$) δ -76.0.

HRMS (ESI) m/z: [$M+Na^+$] Calculated for $C_{12}H_{10}NO_2F_3Na$. 280.05558; Found 280.05558.

IR (neat): 1702 (s), 1555 (w), 1453 (m), 1208 (m), 1155 (s), 1096 (m), 857 (w), 743 (s), 726 (w), 689 (w), 520 (w).

Mp: 86-87 °C.

(Benzofuran-3-ylmethoxy)triisopropylsilane 4h

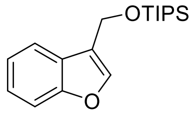

4h was prepared according to a literature[16] procedure with benzofuran-3-carbaldehyde (0.44 g, 3.0 mmol, 1.0 equiv.) and $NaBH_4$ (134 mg, 3.00 mmol, 1.0 equiv.) in dry MeOH (20 mL). The crude alcohol was protected using TIPSCl (0.81 mL, 3.75mmol, 1.25 equiv.) and imidazole (511 mg, 7.50 mmol, 2.5 equiv.) in dry DCM (10 mL) overnight at room temperature. After purification by flash chromatography (*n*-pentane), the desired compound **4h** was obtained as a yellow oil (835 mg, 92% yield).

TLC: 0.90 (*n*-pentane).

^{1}H NMR (400 MHz, $CDCl_3$) δ 7.64 – 7.60 (m, 1H), 7.58 (d, *J* = 1.5 Hz, 1H), 7.48 (d, *J* = 8.1 Hz, 1H), 7.30 (td, *J* = 7.8, 1.5 Hz, 1H), 7.26 – 7.21 (m, 1H), 5.01 – 4.95 (m, 2H), 1.27 – 1.16 (m, 3H), 1.12 (d, *J* = 6.8 Hz, 18H).

^{13}C NMR (101 MHz, $CDCl_3$) δ 155.7, 141.6, 127.0, 124.4, 122.5, 121.4, 120.2, 111.6, 57.6, 18.2, 12.2.

HRMS (ESI) m/z: [M+Na$^+$] Calculated for $C_{18}H_{28}O_2SiNa$: 327.17508; Found 327.17514.

IR (neat): 2942 (w), 2865 (w), 1451 (w), 1104 (w), 1084 (m), 1048 (m), 881 (m), 817 (w), 742 (s), 680 (m), 658 (m).

(Benzofuran-3-ylmethyl)diphenylphosphine oxide 4j

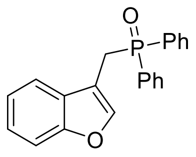

4j was prepared according to a modified literature[17] procedure with 1-iodo-2-(propa-1,2-dien-1-yloxy)benzene (516 mg, 2.0 mmol, 1.0 equiv.), diphenylphosphane (0.7 mL, 4.0 mmol, 2.0 equiv.), LiHMDS (1.3 M in THF, 3.1 mL, 4.0 mmol, 2.0 equiv.) in dry DME (15 mL) at 50 °C for 12 h under argon. Then using H_2O_2 (0.51 mL, 5.0 mmol, 2.5 equiv., 30% wt in water) oxidize. After purification by flash chromatography (*n*-pentane/EtOAc = 1/1), the desired compound **4j** was obtained as a white solid (524 mg, 79% yield). The analytic data are in agreement with the reported literature data.[17]

^{1}H NMR (400 MHz, $CDCl_3$) δ 7.79 – 7.70 (m, 4H), 7.54 (d, *J* = 3.2 Hz, 1H), 7.52 – 7.47 (m, 2H), 7.46 – 7.36 (m, 6H), 7.26 – 7.20 (m, 1H), 7.17 – 7.12 (m, 1H), 3.67 (d, *J* = 13.3 Hz, 2H).

^{13}C NMR (101 MHz, $CDCl_3$) δ 154.9, 143.8 (d, *J* = 7.4 Hz), 132.4 (d, *J* = 99.9 Hz), 132.1 (d, *J* = 2.8 Hz), 131.2 (d, *J* = 9.3 Hz), 128.8 (d, *J* = 11.8 Hz), 128.1 (d, *J* = 5.1 Hz), 124.5, 122.7, 119.6, 111.5, 110.4 (d, *J* = 8.0 Hz), 26.2 (d, *J* = 70.1 Hz).

^{31}P NMR (162 MHz, $CDCl_3$) δ 29.0.

Methyl 3-(3-((2-(benzofuran-3-yl)ethyl)amino)-3-oxopropyl)-1*H*-indole-1-carboxylate 4k

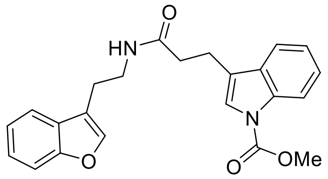

To a solution of commercial 3-(1*H*-indol-3-yl)propanoic acid (568 mg, 2.00 mmol, 1.0 equiv.) in dry DCM (15 mL), 2-(benzofuran-3-yl)ethan-1-amine (323 mg, 2.00 mmol, 1.0 equiv., the amine was synthesized according to the procedure of **4g**), EDCI (422 mg, 2.20 mmol, 1.1 equiv.), DMAP (25 mg, 0.20 mmol, 0.1 equiv.) and TEA (0.31 mL, 2.2 mmol, 1.1 equiv.) were added. The reaction was stirred at r.t. overnight. Then the mixture was diluted with water, extracted with DCM three times. After purification by flash chromatography (*n*-pentane/EtOAc = 1/1), the

unprotected intermediate was obtained. Then **4k** was prepared according to general procedure I. After purification by flash chromatography (*n*-pentane/EtOAc = 1/1), the desired compound **4k** was obtained as a yellow solid (666 mg, 86% yield).

TLC: 0.30 (*n*-pentane/EtOAc = 1/1).

^{1}H NMR (400 MHz, $CDCl_3$) δ 8.16 (d, *J* = 8.2 Hz, 1H), 7.51 (ddd, *J* = 7.6, 5.7, 1.2 Hz, 2H), 7.46 (d, *J* = 8.2 Hz, 1H), 7.38 (s, 1H), 7.36 – 7.31 (m, 1H), 7.31 – 7.27 (m, 2H), 7.26 – 7.19 (m, 2H), 5.53 (s, 1H), 4.00 (s, 3H), 3.56 (q, *J* = 6.5 Hz, 2H), 3.04 (t, *J* = 7.4 Hz, 2H), 2.81 (t, *J* = 6.8 Hz, 2H), 2.51 (t, *J* = 7.4 Hz, 2H).

^{13}C NMR (101 MHz, $CDCl_3$) δ 172.1, 155.5, 151.5, 141.9, 135.7, 130.3, 127.9, 124.9, 124.6, 123.0, 122.7, 122.4, 120.6, 119.5, 119.1, 117.3, 115.4, 111.7, 53.8, 38.9, 36.4, 23.9, 21.0.

HRMS (ESI) m/z: [M+Na$^+$] Calculated for $C_{23}H_{22}N_2O_4Na$. 413.14718; Found 413.14660.

IR (neat): 1730 (m), 1641 (w), 1545 (w), 1453 (m), 1442 (m), 1377 (m), 1308 (w), 1254 (m), 1086 (m), 1044 (w), 742 (s), 700 (w).

Mp: 134-135 °C.

2-Methyl-3-phenylbenzofuran 4m

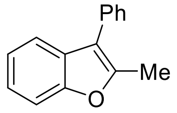

4m was prepared according to a literature procedure[18] with 2-(2-iodophenoxy)-1-phenylpropan-1-one (1.8 g, 5.0 mmol, 1.0 equiv.). After purification by flash chromatography (*n*-pentane/EtOAc = 100/1), the desired compound **4m** was obtained as a yellow oil (0.9 g, 87% yield). The analytic data are in agreement with the reported literature data.[19]

^{1}H NMR (400 MHz, $CDCl_3$) δ 7.60 (dd, *J* = 7.8, 1.5 Hz, 1H), 7.55 – 7.43 (m, 5H), 7.38 (ddt, *J* = 6.8, 5.3, 1.8 Hz, 1H), 7.31 – 7.19 (m, 2H), 2.56 (s, 3H).

^{13}C NMR (101 MHz, $CDCl_3$) δ 154.2, 151.4, 133.0, 129.1, 128.91, 128.89, 127.1, 123.7, 122.8, 119.5, 117.1, 110.9, 13.0.

3-Isopropyl-2-phenylbenzofuran 4n

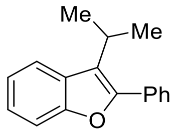

4n was prepared according to a literature procedure[20] with 2-(2-methylprop-1-en-1-yl)phenol (0.60 g, 4.0 mmol, 1.0 equiv.). After purification by flash chromatography (*n*-pentane/EtOAc = 100/1), the desired compound **4n** was obtained as a colourless oil (359 g, 39% yield). The analytic data are in agreement with the reported literature data.[20]

^{1}H NMR (300 MHz, $CDCl_3$) δ 7.76 (ddd, *J* = 7.5, 1.6, 0.7 Hz, 1H), 7.71 – 7.66 (m, 2H), 7.53 – 7.45 (m, 3H), 7.43 – 7.35 (m, 1H), 7.34 – 7.23 (m, 1H), 7.25 – 7.19 (m, 1H), 3.58 – 3.36 (m, 1H), 1.50 (d, *J* = 7.0 Hz, 6H).

^{13}C NMR (76 MHz, $CDCl_3$) δ 154.6, 150.1, 131.5, 128.7, 128.7, 128.4, 128.1, 124.0, 122.1, 121.9, 121.5, 111.5, 25.7, 22.5.

1,2,3,4-Tetrahydrodibenzo[*b*,*d*]furan 4o

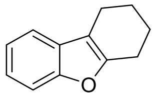

4o was prepared according to a literature[14] procedure with 2-phenoxycyclohexan-1-one (760 mg, 4.0 mmol, 1.0 equiv.). After purification by flash chromatography (*n*-pentane/EtOAc = 100/1), the desired compound **4o** was obtained as a colourless oil (200 mg, 30% yield). The analytic data are in agreement with the reported literature data.[14]

^{1}H NMR (300 MHz, $CDCl_3$) δ 7.45 – 7.37 (m, 2H), 7.23 – 7.15 (m, 2H), 2.79 – 2.71 (m, 2H), 2.66 – 2.57 (m, 2H), 2.01 – 1.78 (m, 4H).

^{13}C NMR (76 MHz, $CDCl_3$) δ 154.4, 154.2, 129.0, 123.1, 122.2, 118.5, 113.0, 110.9, 23.6, 23.1, 22.8, 20.6.

Dimethyl 3-methyl-1*H*-indole-1,5-dicarboxylate Moc-**1ad**

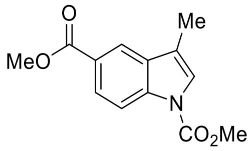

Moc-**1ad** was prepared according to the general procedure II with methyl 3-methyl-1*H*-indole-5-carboxylate (0.28 g, 1.5 mmol). After purification by flash chromatography (*n*-pentane/EtOAc = 25/2), the desired compound Moc-**1ad** was obtained as a white solid (180 mg, 49% yield). Methyl 3-methyl-1*H*-indole-5-carboxylate was synthesized according to a literature procedure with 3-formyl-1*H*-indole-5-carbonitrile.[9]

TLC: 0.36 (*n*-pentane/EtOAc = 100/7).

^{1}H NMR (400 MHz, $CDCl_3$) δ 8.23 (dd, *J* = 1.8, 0.9 Hz, 1H), 8.17 (d, *J* = 8.7 Hz, 1H), 8.06 – 7.99 (m, 1H), 7.41 (s, 1H), 4.04 (s, 3H), 3.95 (s, 3H), 2.30 (d, *J* = 1.3 Hz, 3H).

^{13}C NMR (101 MHz, $CDCl_3$) δ 167.7, 151.4, 138.3, 131.4, 126.0, 124.8, 123.7, 121.5, 117.9, 114.9, 54.0, 52.2, 9.7.

HRMS (ESI) m/z: [M+Na$^+$] Calculated for $C_{13}H_{13}NO_4Na$: 270.07368; Found 270.07357.

IR (neat): 1735 (m), 1714 (s), 1438 (m), 1388 (w), 1369 (m), 1348 (w), 1288 (w), 1270 (w), 1228 (s), 1075 (s), 1038 (w), 763 (s), 745 (m).

Mp: 69-71 °C.

➢ Synthesis of Bioactive compounds

Bioactive compounds

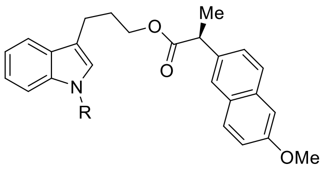

R = H: **1r** from Naproxen
R = CO_2Me: Moc-**1r**

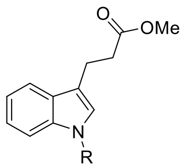

R = H: **1s** from 3-Indolepropionic acid (IPA)
R = Moc: Moc-**1s**

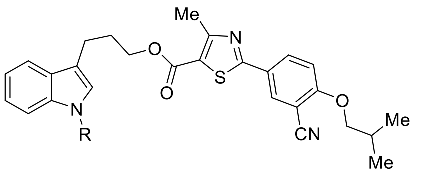

R = H: **1t** from Febuxostat
R = Moc: Moc-**1t**

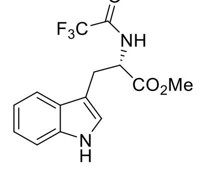

1u from *L*-Tryptophan

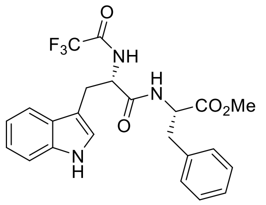

1v from TFA-Trp-Phe-OMe

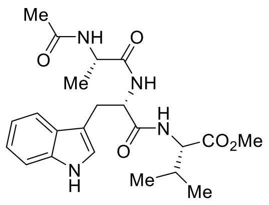

1w from Ac-Ala-Trp-Val-OMe

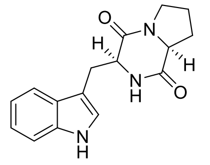

1x from Brevianamide F

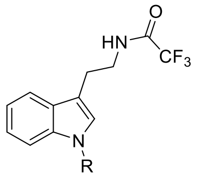

R = H: **1y** from Tryptamine
R = CO_2Et: Eoc-**1y**

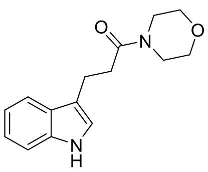

1z inhibitors of 5-Lipoxygenase

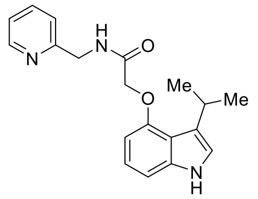

1aa Antibacterial agent

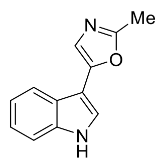

1ab from Pimprinine

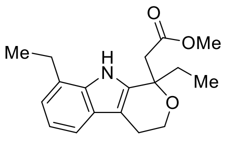

1ac from Etodolac

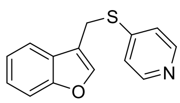

4p Plant antibacterial agent

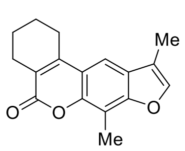

4q Psoralen derivatives

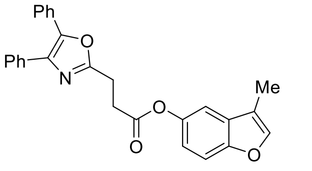

4r from Oxaprozin

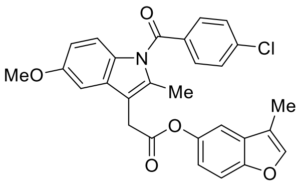

4s from Indomethacin

3-(1*H*-Indol-3-yl)propyl (*S*)-2-(6-methoxynaphthalen-2-yl)propanoate 1r

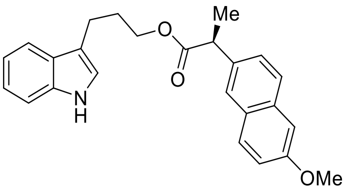

3-(1*H*-Indol-3-yl)propan-1-ol (750 mg, 4.28 mmol) and naproxen (986 mg, 4.28 mmol) were dissolved in anhydrous CH_2Cl_2 (15 mL), and EDCI (821 mg, 4.28 mmol) as well as DMAP (523 mg, 4.28 mmol) were added to the solution. The reaction mixture was stirred at room temperature for 18 h. The solution was concentrated under vacuum. The residue was then taken up in ethyl acetate (30 ml) and washed with 1 M HCl (10 ml), 1M $NaHCO_3$(10 ml), and brine (10 ml). The organic layer was dried over anhydrous Na_2SO_4. After concentration, the residue was purified by flash column chromatography (*n*-pentane/EtOAc = 20/3) to give 3-(1*H*-indol-3-yl)propyl (*S*)-2-(6-methoxynaphthalen-2-yl)propanoate **1r** as white solid (920 mg, 55%).

TLC: 0.75 (*n*-pentane/EtOAc = 20/3).

^{1}H NMR (300 MHz, $CDCl_3$) δ 7.83 (s, 1H), 7.73 – 7.70 (m, 3H), 7.43 (ddd, *J* = 7.8, 2.9, 1.5 Hz, 2H), 7.32 (dt, *J* =

8.2, 1.0 Hz, 1H), 7.22 – 7.09 (m, 3H), 7.03 (ddd, *J* = 8.0, 7.0, 1.1 Hz, 1H), 6.78 (d, *J* = 2.2 Hz, 1H), 4.13 (t, *J* = 6.4 Hz, 2H), 3.91 (s, 3H), 3.91 – 3.84 (q, *J* = 7.0 Hz, 1H), 2.71 (t, *J* = 7.2 Hz, 2H), 1.98 (p, *J* = 6.7 Hz, 2H), 1.59 (d, *J* = 7.1 Hz, 3H).

^{13}C NMR (101 MHz, CDCl3) δ 174.9, 157.8, 136.4, 136.0, 133.8, 129.4, 129.1, 127.4, 127.3, 126.5, 126.1, 122.1, 121.5, 119.3, 119.1, 118.9, 115.4, 111.2, 105.8, 64.4, 55.5, 45.7, 29.0, 21.4, 18.6.

HRMS (ESI) m/z: [M+Na$^+$] Calculated for $C_{25}H_{25}NO_3Na$: 410.17266; found: 410.17263.

IR (neat): 3431 (m), 1733 (m), 1724 (s), 1558 (w), 1506 (w), 1457 (m), 1187 (s), 1172 (m), 1157 (s), 1090 (w), 1026 (m), 863 (m), 820 (m), 794 (w), 484 (m), 472 (m).

Mp: 124-125 °C.

Methyl (*S*)-3-(3-((2-(6-methoxynaphthalen-2-yl)propanoyl)oxy)propyl)-1*H*-indole-1-carboxylate Moc-**1r**

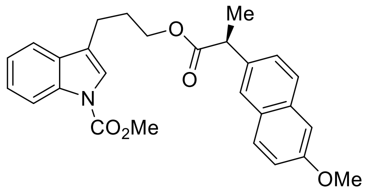

To a solution of NaH (60%, 80 mg, 2.0 mmol) in DMF (2 mL), 3-(1*H*-indol-3-yl)propyl (*S*)-2-(6-methoxynaphthalen-2-yl)propanoate **1r** (0.36 g, 0.93 mmol) in DMF (1 mL) was added under argon at 0 °C. The mixture was stirred at 0 °C for 30 min. Methyl chloroformate (0.16 mL, 2.1 mmol) was added dropwise to the solution. The mixture was stirred at 0 °C for 10 min, and then allowed to warm to room temperature in 2 h. After cooling down to 0 ºC, water (1 mL) was added slowly to quench the reaction. The mixture was extracted with DCM (3 x 10 mL). The combined organic phase was dried over Na_2SO_4. After concentration, the residue was purified by silica-gel column chromatography using *n*-pentane/EtOAc (25:2) as an eluent to obtain Moc-**1r** as colourless oil (320 mg, 77%).

TLC: 0.26 (*n*-pentane/EtOAc = 25/2).

^{1}H NMR (400 MHz, $CDCl_3$) δ 8.13 (s, 1H), 7.72 (d, *J* = 2.8 Hz, 1H), 7.70 (d, *J* = 2.1 Hz, 2H), 7.43 (dd, *J* = 8.5, 1.8 Hz, 1H), 7.34 – 7.27 (m, 3H), 7.18 – 7.12 (m, 2H), 7.10 (d, *J* = 2.5 Hz, 1H), 4.22 – 4.10 (m, 2H), 4.00 (s, 3H), 3.91 (s, 3H), 3.88 (dd, *J* = 14.9, 7.8 Hz, 1H), 2.60 (t, *J* = 7.6 Hz, 2H), 1.98 (dt, *J* = 8.3, 6.5 Hz, 2H), 1.60 (d, *J* = 7.1 Hz, 3H).

^{13}C NMR (101 MHz, $CDCl_3$) δ 174.8, 157.8, 151.5, 135.9, 135.7, 133.8, 130.5, 129.4, 129.1, 127.3, 126.4, 126.1, 124.7, 122.8, 122.1, 120.9, 119.1, 119.0, 115.3, 105.7, 64.2, 55.4, 53.7, 45.7, 28.2, 21.2, 18.5.

HRMS (ESI) m/z: [M+Na$^+$] Calculated for $C_{27}H_{27}NO_5Na$: 468.17814; found: 468.17805.

IR (neat): 1727 (s), 1456 (s), 1440 (m), 1377 (m), 1254 (s), 1175 (m), 1095 (w), 1031 (w), 764 (m), 746 (m).

Mp: 92-93 °C.

Methyl 3-(1*H*-indol-3-yl)propanoate 1s

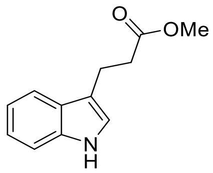

1s was prepared according to a literature procedure[21] with 3-(1*H*-indol-3-yl)propanoic acid (0.95 g, 5.0 mmol, 1.0 equiv.) and MeI (1.6 mL, 25 mmol, 5.0 equiv.) in dry CH_3CN (10 mL). After purification by flash chromatography (*n*-pentane/EtOAc = 10/1), the desired compound **1s** was obtained as a brown oil (914 mg, 90% yield). The analytic data are in agreement with the reported literature data.[22]

^{1}H NMR (300 MHz, $CDCl_3$) δ 7.95 (s, 1H), 7.59 (d, *J* = 7.7 Hz, 1H), 7.33 (dd, *J* = 5.5, 3.5 Hz, 1H), 7.27 – 7.05 (m, 2H), 7.04 – 6.96 (m, 1H), 3.66 (s, 3H), 3.09 (t, *J* = 7.7 Hz, 2H), 2.76 – 2.65 (m, 2H).

^{13}C NMR (76 MHz, $CDCl_3$) δ 173.9, 136.3, 127.2, 122.1, 121.4, 119.4, 118.7, 115.0, 111.2, 51.6, 34.8, 20.7.

Methyl 3-(3-methoxy-3-oxopropyl)-1*H*-indole-1-carboxylate Moc-**1s**

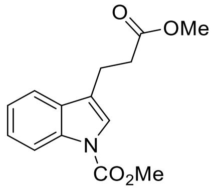

Moc-**1s** was prepared according to the general procedure II with **1s** (0.69 g, 3.0 mmol). After purification by flash chromatography (*n*-pentane/EtOAc = 25/1), the desired compound Moc-**1s** was obtained as a yellow oil (579 mg, 95% yield).

TLC: 0.20 (*n*-pentane/EtOAc = 25/1).

^{1}H NMR (400 MHz, $CDCl_3$) δ 8.16 (s, 1H), 7.56 – 7.50 (m, 1H), 7.40 (s, 1H), 7.37 – 7.32 (m, 1H), 7.27 (tt, *J* = 7.1, 1.3 Hz, 1H), 4.04 – 3.99 (m, 3H), 3.70 (d, *J* = 1.3 Hz, 3H), 3.08 – 3.01 (m, 2H), 2.84 – 2.67 (m, 2H).

^{13}C NMR (101 MHz, $CDCl_3$) δ 173.4, 151.6, 135.7, 130.3, 124.9, 122.9, 122.3, 120.4, 119.0, 115.4, 53.8, 51.9, 33.7, 20.4.

HRMS (ESI) m/z: [$M+Na^+$] Calculated for $C_{14}H_{15}NO_4Na$: 284.089933; Found 284.08905.

IR (neat): 1730 (s), 1455 (m), 1438 (m), 1375 (m), 1308 (w), 1249 (s), 1218 (m), 1196 (m), 1161 (m), 1088 (s), 1054 (w), 1018 (w), 984 (w), 744 (s).

3-(1*H*-Indol-3-yl)propyl 2-(3-cyano-4-isobutoxyphenyl)-4-methylthiazole-5-carboxylate 1t

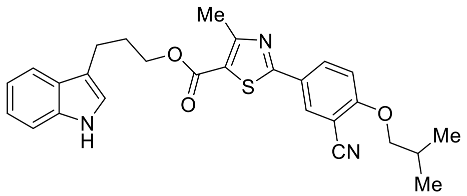

1t was prepared according to a literature procedure[23] with 3-(1*H*-indol-3-yl)propan-1-ol (750 mg, 4.30 mmol, 1.0 equiv.) and febuxostat (1.64 g, 5.20 mmol, 1.2 equiv.). After purification by flash chromatography (*n*-pentane/EtOAc = 4/1), the desired compound **1t** was obtained as a white solid (867 mg, 43% yield).

TLC: 0.32 (*n*-pentane/EtOAc = 4/1).

^{1}H NMR (300 MHz, $CDCl_3$) δ 8.18 (d, *J* = 2.4 Hz, 1H), 8.09 (dd, *J* = 8.7, 2.4 Hz, 1H), 8.03 – 7.98 (m, 1H), 7.66 – 7.55 (m, 1H), 7.45 – 7.33 (m, 1H), 7.21 (ddd, *J* = 8.1, 6.9, 1.3 Hz, 1H), 7.13 (ddd, *J* = 8.1, 6.9, 1.2 Hz, 1H), 7.06 – 6.91 (m, 2H), 4.37 (t, *J* = 6.5 Hz, 2H), 3.90 (d, *J* = 6.5 Hz, 2H), 3.06 – 2.85 (m, 2H), 2.78 (s, 3H), 2.38 – 2.08 (m, 3H), 1.09 (d, *J* = 6.6 Hz, 6H).

^{13}C NMR (76 MHz, $CDCl_3$) δ 167.3, 162.6, 162.2, 161.2, 136.5, 132.7, 132.2, 127.4, 126.1, 122.2, 122.0, 121.6, 119.4, 118.9, 115.6, 115.3, 112.7, 111.3, 103.1, 75.8, 65.1, 29.1, 28.3, 21.8, 19.2, 17.6.

HRMS (ESI) m/z: [M+Na$^+$] Calculated for $C_{27}H_{27}N_3O_3SNa$: 496.16653. Found: 496.16641.

IR (neat): 1685 (s), 1653 (w), 1601 (w), 1558 (w), 1506 (m), 1457 (w), 1430 (m), 1395 (w), 1335 (s), 1287 (s), 1275 (s), 1006 (m), 935 (w), 911 (w), 830 (m), 761 (m), 736 (s).

Mp: 145-146 °C.

3-(1*H*-Indol-3-yl)propyl 2-(3-cyano-4-isobutoxyphenyl)-4-methylthiazole-5-carboxylate Moc-**1t**

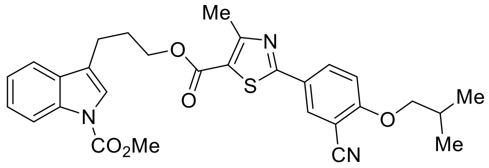

Moc-**1t** was synthesized according to general procedure II with **1t** (0.47 g, 1.0 mmol). After purification by flash chromatography (*n*-pentane/EtOAc = 4/1), the desired compound Moc-**1t** was obtained as a white solid (180.0 mg, 34% yield).

TLC: 0.40 (*n*-pentane/EtOAc = 4/1).

^{1}H NMR (300 MHz, $CDCl_3$) δ 8.16 (d, *J* = 2.4 Hz, 2H), 8.08 (dd, *J* = 8.7, 2.4 Hz, 1H), 7.54 (dd, *J* = 6.9, 0.9 Hz, 1H), 7.42 (s, 1H), 7.39 – 7.31 (m, 1H), 7.31 – 7.23 (m, 1H), 7.01 (d, *J* = 9.0 Hz, 1H), 4.38 (t, *J* = 6.3 Hz, 2H), 4.01 (s, 3H), 3.90 (d, *J* = 6.5 Hz, 2H), 2.86 (t, *J* = 7.4 Hz, 2H), 2.76 (s, 3H), 2.35 – 2.03 (m, 3H), 1.09 (d, *J* = 6.6 Hz, 6H).

^{13}C NMR (76 MHz, $CDCl_3$) δ 167.4, 162.6, 162.1, 161.4, 151.7, 135.8, 132.7, 132.2, 130.5, 126.1, 124.8, 122.9, 122.3, 121.7, 120.7, 119.1, 115.5, 115.4, 112.7, 103.0, 75.8, 64.9, 53.8, 28.3, 28.1, 21.7, 19.2, 17.6.

HRMS (ESI) m/z: [M+Na$^+$] Calculated for $C_{29}H_{29}N_3O_5SNa$: 554.17201. Found: 554.17169.

IR (neat): 1733 (m), 1715 (m), 1456 (m), 1372 (m), 1254 (s), 1093 (m), 1012 (w), 762 (m), 747 (m).

Mp: 99-102 °C.

Methyl (2,2,2-trifluoroacetyl)-*L*-tryptophanate 1u

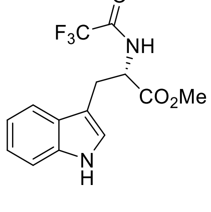

1u was prepared according to a literature procedure[24] with *L*-tryptophan methyl ester hydrochloride (1.27 g, 5.0 mmol, 1.0 equiv.) and ethyl trifluoroacetate (1.2 mL, 10 mmol, 2.0 equiv.) in dry MeOH (15 mL). After purification by flash chromatography (*n*-pentane/EtOAc = 2/1), the desired compound **1u** was obtained as a white solid (1.55 g, 98%

yield). The analytic data are in agreement with the reported literature data.[24]

^{1}H NMR (400 MHz, $CDCl_3$) δ 8.18 (s, 1H), 7.50 (dd, *J* = 7.9, 1.2 Hz, 1H), 7.39 – 7.34 (m, 1H), 7.22 (ddd, *J* = 8.2, 7.0, 1.3 Hz, 1H), 7.14 (ddd, *J* = 8.0, 7.0, 1.1 Hz, 1H), 6.97 (d, *J* = 2.4 Hz, 1H), 6.91 – 6.86 (m, 1H), 5.01 – 4.86 (m, 1H), 3.74 (s, 3H), 3.49 – 3.38 (m, 2H).

^{13}C NMR (101 MHz, $CDCl_3$) δ 170.8, 156.8 (q, *J* = 37.6 Hz), 136.2, 127.4, 123.0, 122.7, 120.1, 118.4, 114.9 (q, *J* = 288.1 Hz), 111.5, 108.9, 53.5, 53.0, 27.2.

^{19}F NMR (376 MHz, $CDCl_3$) δ -75.9.

Methyl (2,2,2-trifluoroacetyl)-*L*-tryptophyl-*L*-phenylalaninate 1v

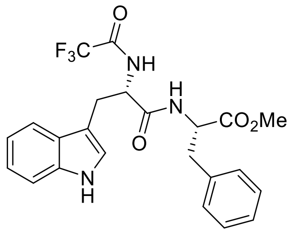

1v was prepared according to a literature procedure[25] with H-*L*-Phe-OMe•HCl (1.08 g, 5.0 mmol, 1.0 equiv.), Cbz-*L*-Trp-OH (1.69 g, 5.0 mmol, 1.0 equiv.). After deprotection by hydrogenation, crude H-*L*-Trp-*L*-Phe-OMe was obtained as a white solid. Under argon, crude H-*L*-Trp-*L*-Phe-OMe (731 mg, 2.00 mmol, 1.0 equiv.) was dissolved in the dry DCM (12 mL) at 0 °C. TFAA (0.34 mL, 2.4 mmol, 1.2 equiv.) was added dropwise. After 5 h at 0 °C, water was added to terminate the reaction. The mixture was extracted with DCM three times and the combined organic phase was dried over Na_2SO_4. After concentration, the residue was purified by silica-gel column chromatography (*n*-pentane/EtOAc = 3/2), the desired compound **1v** was obtained as a white solid (600 mg, 65% yield).

TLC: 0.70 (*n*-pentane/EtOAc = 1/1).

^{1}H NMR (400 MHz, DMSO) δ 10.83 (d, *J* = 2.4 Hz, 1H), 9.55 (d, *J* = 8.4 Hz, 1H), 8.76 (d, *J* = 7.5 Hz, 1H), 7.68 (dd, *J* = 7.6, 1.2 Hz, 1H), 7.37 – 7.32 (m, 1H), 7.30 – 7.18 (m, 5H), 7.13 (d, *J* = 2.4 Hz, 1H), 7.08 (ddd, *J* = 8.1, 6.9, 1.2 Hz, 1H), 7.00 (ddd, *J* = 8.0, 7.0, 1.1 Hz, 1H), 4.67 (ddd, *J* = 10.1, 8.3, 4.6 Hz, 1H), 4.54 (ddd, *J* = 8.8, 7.5, 5.8 Hz, 1H), 3.60 (s, 3H), 3.22 – 2.94 (m, 4H).

^{13}C NMR (101 MHz, DMSO) δ 171.7, 170.2, 156.0 (q, *J* = 36.4 Hz), 137.1, 136.1, 129.1, 128.2, 127.1, 126.6, 123.8, 121.0, 118.5, 118.2, 115.8 (q, *J* = 288.3 Hz), 111.3, 109.5, 53.8, 53.7, 51.9, 36.5, 27.1.

^{19}F NMR (376 MHz, DMSO) δ -74.0.

HRMS (ESI) m/z: [M+Na$^+$] Calculated for $C_{23}H_{22}N_3O_4F_3Na$: 484.14546. Found: 484.14247.

IR (neat): 1718 (m), 1657 (m), 1532 (w), 1454 (m), 1381 (w), 1358 (w), 1257 (w), 1211 (m), 1181 (m), 1091 (w), 742 (s), 701 (w).

Mp: 189-190 °C.

Methyl acetyl-*L*-alanyl-*L*-tryptophyl-*L*-valinate 1w

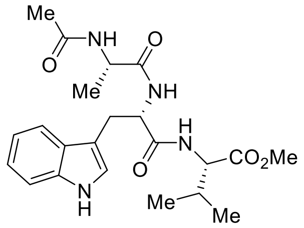

1w was prepared according to a modified literature procedure.[25] A solution of H-*L*-Vla-OMe•HCl (1.68 g, 10.0 mmol, 1.0 equiv.), Cbz-L-Trp-OH (3.39 g, 10.0 mmol, 1.0 equiv.), HOBt (1.48 g, 11.0 mmol, 1.1 equiv.) and NMM (1.90 mL, 17.0 mmol, 1.7 equiv.) in DMF (40 mL) was cooled in an ice bath and subsequently treated with EDCI (2.3 g, 12 mmol, 1.2 equiv.). After 0.5 h at 0 °C, the mixture was allowed to warm to room temperature, and stirred for 2 h. Then, H_2O (60 mL) was added and the mixture was extracted with EtOAc (3 x 60 mL). The organic phase was washed sequentially with 1 M aqueous HCl (2 x 40 mL), saturated aqueous $NaHCO_3$ (2 x 40 mL) and brine (2 x 20 mL). The organic phase was dried over Na_2SO_4, filtered and concentrated in vacuo. The corresponding dipeptide was suspended in a 1/1 mixture of MeOH/EtOAc (40 mL) with Pd/C (10%), and was stirred for 12 h under hydrogen balloon atmosphere at room temperature. The reaction mixture was then filtered through a pad of Celite, and the filtrate was concentrated in vacuo. The corresponding product was mixed with Ac-L-Ala-OH (1.32 g, 10.0 mmol, 1.0 equiv.), HOBt (1.48 g, 11.0 mmol, 1.1 equiv.), and NMM (1.90 mL, 17.0 mmol, 1.7 equiv.) in DMF (40 mL). The mixture was cooled in an ice bath and subsequently treated with EDCI (2.3 g, 12 mmol, 1.2 equiv.). After 0.5 h at 0 °C, the mixture was warmed to room temperature for 2 h. Then H_2O (60 mL) was added and the mixture was extracted with EtOAc (3 x 60 mL). The organic phase was washed sequentially with 1 M aqueous HCl (2 x 40 mL), saturated aqueous $NaHCO_3$ (2 x 40 mL) and brine (2 x 40 mL). The organic phase was dried over Na_2SO_4, filtered and concentrated in vacuo. The resulting residue was purified by column chromatography (EtOAc/MeOH = 20/1) yielding the tripeptide **1w** as a white solid (1.7 g, 40% yield).

TLC: 0.20 (EtOAc/MeOH = 20/1).

^{1}H NMR (400 MHz, DMSO) δ 10.82 (d, *J* = 2.3 Hz, 1H), 8.05 (d, *J* = 8.1 Hz, 1H), 7.99 (d, *J* = 7.3 Hz, 1H), 7.92 (d, *J* = 8.0 Hz, 1H), 7.55 (dd, *J* = 7.7, 1.1 Hz, 1H), 7.34 – 7.28 (m, 1H), 7.12 (d, *J* = 2.3 Hz, 1H), 7.05 (ddd, *J* = 8.1, 7.0, 1.2 Hz, 1H), 6.96 (ddd, *J* = 7.9, 6.9, 1.1 Hz, 1H), 4.63 – 4.53 (m, 1H), 4.28 – 4.21 (m, 1H), 4.20 – 4.13 (m, 1H), 3.62 (s, 3H), 3.12 (dd, *J* = 14.8, 5.2 Hz, 1H), 2.96 (dd, *J* = 14.8, 8.4 Hz, 1H), 2.06 – 1.97 (m, 1H), 1.79 (s, 3H), 1.12 (d, *J* = 7.1 Hz, 3H), 0.86 (dd, *J* = 6.8, 5.7 Hz, 6H).

^{13}C NMR (101 MHz, DMSO) δ 172.2, 171.7, 171.6, 169.1, 136.0, 127.4, 123.5, 120.8, 118.3, 118.1, 111.2, 109.8, 57.4, 53.0, 51.6, 48.1, 29.9, 27.3, 22.4, 18.8, 18.2, 17.9.

HRMS (ESI) m/z: [M+Na$^+$] Calculated for $C_{22}H_{30}N_4O_5Na$: 453.21084. Found: 453.21136.

IR (neat): 3283 (w), 2965 (w), 1739 (w), 1638 (s), 1534 (m), 1437 (w), 1371 (w), 1278 (w), 1212 (w), 742 (m).

Mp: 229-230 °C.

Brevianamide F 1x

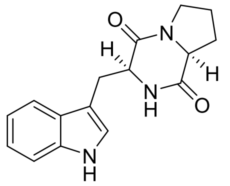

1x was prepared according to a literature procedure[26] with *L*-tryptophan (2.0 g, 10 mmol, 1.0 equiv.). After purification by flash chromatography (EtOAc), the desired compound **1x** was obtained as a white solid (1.05 g, 57% yield). The analytic data are in agreement with the reported literature data.[26]

^{1}H NMR (400 MHz, $CDCl_3$) δ 8.36 (s, 1H), 7.59 (d, *J* = 7.9 Hz, 1H), 7.39 (dd, *J* = 8.2, 2.0 Hz, 1H), 7.26 – 7.20 (m, 1H), 7.17 – 7.11 (m, 1H), 7.10 – 7.06 (m, 1H), 5.75 (s, 1H), 4.46 – 4.32 (m, 1H), 4.15 – 4.03 (m, 1H), 3.81 – 3.71 (m, 1H), 3.71 – 3.52 (m, 2H), 3.04 – 2.89 (m, 1H), 2.38 – 2.28 (m, 1H), 2.11 – 1.96 (m, 2H), 1.95 – 1.83 (m, 1H).

^{13}C NMR (101 MHz, $CDCl_3$) δ 169.5, 165.7, 136.8, 126.8, 123.5, 122.9, 120.1, 118.6, 111.7, 110.0, 59.4, 54.7, 45.6, 28.4, 27.0, 22.8.

***N*-(2-(1*H*-Indol-3-yl)ethyl)-2,2,2-trifluoroacetamide 1y**

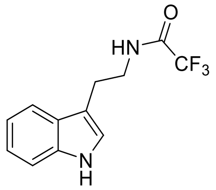

1y was prepared according to a literature procedure[27] with tryptamine (4.0 g, 25 mmol, 1.0 equiv.) and TFAA (4.25 mL, 30.0 mmol, 1.2 equiv.) in dry DCM (150 mL). After purification by flash chromatography (*n*-pentane/DCM = 1/1), the desired compound **1y** was obtained as a yellow solid (5.4 g, 85% yield). The analytic data are in agreement with the reported literature data.[27]

^{1}H NMR (300 MHz, $CDCl_3$) δ 8.17 (s, 1H), 7.66 – 7.56 (m, 1H), 7.44 – 7.35 (m, 1H), 7.25 (ddd, *J* = 8.2, 7.0, 1.3 Hz, 1H), 7.17 (ddd, *J* = 8.0, 7.1, 1.2 Hz, 1H), 7.05 (d, *J* = 2.3 Hz, 1H), 6.45 (s, 1H), 3.69 (q, *J* = 6.5 Hz, 2H), 3.06 (td, *J* = 6.7, 0.8 Hz, 2H).

^{13}C NMR (76 MHz, $CDCl_3$) δ 157.4 (q, *J* = 37.3 Hz), 136.6, 127.0, 122.6, 122.4, 119.9, 118.6, 115.9 (q, *J* = 288.0 Hz), 111.8, 111.6, 40.2, 24.8.

^{19}F NMR (282 MHz, $CDCl_3$) δ -76.0.

Ethyl 3-(2-(2,2,2-trifluoroacetamido)ethyl)-1*H*-indole-1-carboxylate Eoc-1y

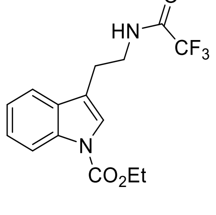

Eoc-1y was prepared according to modified general procedure I with **1y** (0.52g, 2.0 mmol) and ethyl 1*H*-imidazole-1-carboxylate (0.31 g, 2.2 mmol, 1.1 equiv.) in dry CH_3CN (6 mL). After work up, the NMR pure product **Eoc-1y** was obtained as a white solid (649 mg, 99% yield).

TLC: 0.18 (*n*-pentane/EtOAc = 100/7).

^{1}H NMR (400 MHz, $CDCl_3$) δ 8.18 (d, *J* = 8.2 Hz, 1H), 7.55 – 7.51 (m, 1H), 7.46 (s, 1H), 7.36 (ddd, *J* = 8.4, 7.2, 1.3 Hz, 1H), 7.31 – 7.25 (m, 1H), 6.53 (s, 1H), 4.46 (q, *J* = 7.1 Hz, 2H), 3.69 (q, *J* = 6.7 Hz, 2H), 3.00 (td, *J* = 7.0, 1.1 Hz, 2H), 1.46 (t, *J* = 7.1 Hz, 3H).

^{13}C NMR (101 MHz, $CDCl_3$) δ 157.3 (q, *J* = 37.0 Hz), 150.8, 135.7, 129.9, 125.0, 123.1, 123.0, 118.7, 117.2, 115.8 (q, *J* = 286.0 Hz), 115.5, 63.3, 39.5, 24.5, 14.4.

^{19}F NMR (376 MHz, $CDCl_3$) δ -75.9.

HRMS (ESI) m/z: [M+Na$^+$] Calculated for $C_{15}H_{15}N_2O_3F_3Na$: 351.09270; found: 351.09278.

IR (neat): 1732 (m), 1698 (s), 1559 (m), 1456 (m), 1342 (w), 1250 (m), 1202 (m), 1155 (s), 1091 (s), 764 (w), 743 (m).

Mp: 108-109 °C.

3-(1*H*-Indol-3-yl)-1-morpholinopropan-1-one 1z

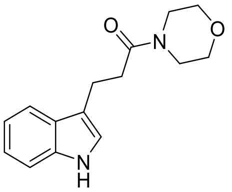

1z was prepared according to a literature procedure[28] with 3-(1*H*-indol-3-yl)propanoic acid (1.89 g, 10.0 mmol, 1.0 equiv.) and morpholine (1.28 mL, 11.0 mmol, 1.1 equiv.) in dry DMF (20 mL). After purification by flash chromatography (EtOAc/MeOH = 100/3), the desired compound **1z** was obtained as a white solid (2.1 g, 82% yield).

TLC: 0.44 (EtOAc/MeOH = 20/1).

^{1}H NMR (300 MHz, $CDCl_3$) δ 8.16 (s, 1H), 7.60 (d, *J* = 7.9 Hz, 1H), 7.36 (d, *J* = 8.1 Hz, 1H), 7.20 (ddd, *J* = 8.1, 7.0, 1.3 Hz, 1H), 7.12 (ddd, *J* = 8.0, 7.0, 1.2 Hz, 1H), 7.03 (d, *J* = 2.1 Hz, 1H), 3.63 – 3.53 (m, 4H), 3.40 – 3.27 (m, 4H), 3.15 (dd, *J* = 8.5, 6.6 Hz, 2H), 2.71 (dd, *J* = 8.4, 6.7 Hz, 2H).

^{13}C NMR (76 MHz, $CDCl_3$) δ 171.7, 136.4, 127.3, 122.2, 121.9, 119.5, 118.8, 115.3, 111.4, 66.9, 66.5, 46.1, 42.0, 33.8, 21.2.

HRMS (ESI) m/z: [M+Na$^+$] Calculated for $C_{15}H_{18}N_2O_2Na$: 281.12605; found: 281.12598.

IR (neat): 1616 (s), 1558 (w), 1457 (m), 1269 (w), 1230 (w), 1112 (m), 1046 (w), 1024 (w), 856 (m), 804 (m), 753 (s), 568 (w), 558 (w).

Mp: 161-162 °C.

2-((3-Isopropyl-1*H*-indol-4-yl)oxy)-*N*-(pyridin-2-ylmethyl)acetamide 1aa

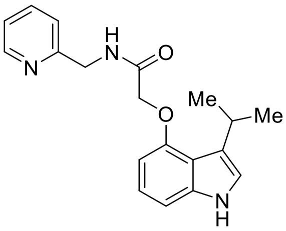

1aa was prepared according to a patent (EP2639220 A1) starting with benzofuran-4-ol (2.67 g, 20.0 mmol, 1.0 equiv.).

After 4 steps and purification by flash chromatography (EtOAc), the desired compound **1aa** was obtained as a brown solid (0.7 g, 11% yield). The analytic data are in agreement with the reported literature data.[11]

^{1}H NMR (300 MHz, $CDCl_3$) δ 8.57 (ddd, *J* = 4.9, 1.8, 1.0 Hz, 1H), 8.21 (s, 1H), 7.81 (s, 1H), 7.68 (td, *J* = 7.7, 1.8 Hz, 1H), 7.32 – 7.27 (m, 1H), 7.25 – 7.19 (m, 1H), 7.11 – 6.97 (m, 2H), 6.90 (dd, *J* = 2.4, 1.0 Hz, 1H), 6.48 (dd, *J* = 7.3, 1.3 Hz, 1H), 4.76 – 4.68 (m, 4H), 3.65 – 3.50 (m, 1H), 1.31 (s, 3H), 1.29 (s, 3H).

^{13}C NMR (76 MHz, $CDCl_3$) δ 168.8, 156.1, 152.1, 149.2, 138.5, 137.0, 124.4, 122.6, 122.1, 118.4, 116.7, 105.9, 100.8, 67.5, 44.3, 38.7, 26.7, 24.2.

5-(1*H*-Indol-3-yl)-2-methyloxazole 1ab

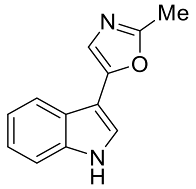

1ab was prepared according to a literature procedure[29] with 1*H*-indole-3-carbaldehyde (1.60 g, 10.0 mmol, 1.0 equiv.). After purification by flash chromatography (EtOAc), the desired compound **1ab** was obtained as a brown solid (380 mg, 20% yield). The analytic data are in agreement with the reported literature data.[29]

^{1}H NMR (400 MHz, $CDCl_3$) δ 8.46 (s, 1H), 7.84 (ddd, *J* = 7.5, 1.6, 0.8 Hz, 1H), 7.51 (d, *J* = 2.6 Hz, 1H), 7.43 (dt, *J* = 8.3, 0.9 Hz, 1H), 7.33 – 7.20 (m, 2H), 7.15 (s, 1H), 2.54 (s, 3H).

^{13}C NMR (101 MHz, $CDCl_3$) δ 159.4, 147.5, 136.4, 124.2, 123.2, 121.6, 121.0, 120.2, 120.1, 111.6, 106.2, 14.2.

Methyl 2-(1,8-diethyl-1,3,4,9-tetrahydropyrano[3,4-*b*]indol-1-yl)acetate 1ac

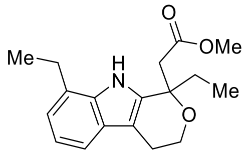

1ac was synthesized according a literature procedure[21] with etodolac (2.87 g, 5.00 mmol, 1.0 equiv.), MeI (1.6 mL, 25 mmol, 5.0 equiv.) and DBU (0.90 mL, 6.0 mmol, 1.2 equiv.) in CH_3CN (10 mL) refluxed for 8 h. After purification by flash chromatography (Pentane/EtOAc = 8/1), the desired compound **1ac** was obtained as a white solid (1.20 g, 80% yield). The analytic data are in agreement with the reported literature data.[30]

^{1}H NMR (300 MHz, $CDCl_3$) δ 9.09 (s, 1H), 7.38 (d, *J* = 7.5 Hz, 1H), 7.16 – 6.94 (m, 2H), 4.23 – 4.01 (m, 1H), 4.02 – 3.91 (m, 1H), 3.74 (s, 3H), 3.29 – 2.60 (m, 6H), 2.26 – 2.10 (m, 1H), 2.11 – 1.91 (m, 1H), 1.39 (t, *J* = 7.5 Hz, 3H), 0.85 (t, *J* = 7.5 Hz, 3H).

^{13}C NMR (76 MHz, $CDCl_3$) δ 173.4, 136.0, 134.6, 126.7, 126.3, 120.5, 119.7, 116.1, 108.5, 74.7, 60.8, 52.1, 42.9, 30.8, 24.3, 22.5, 13.9, 7.7.

4-((Benzofuran-3-ylmethyl)thio)pyridine 4p

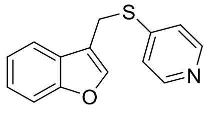

4p was prepared according to a modified literature procedure[17] with 1-iodo-2-(propa-1,2-dien-1-yloxy)benzene (774 mg, 3.00 mmol, 1.5 equiv.), pyridine-4-thiol (222 mg, 2.00 mmol, 1.0 equiv.), LiHMDS (1.3 M in THF, 3.1 mL, 4.0

mmol, 2.0 equiv.) in dry DME (15 mL) at 50 °C for 12 h under argon. After purification by flash chromatography (EtOAc), the desired compound **4p** was obtained as a brown solid (129 mg, 27% yield). The analytic data are in agreement with the reported literature data.[17]

^{1}H NMR (300 MHz, $CDCl_3$) δ 8.46 – 8.37 (m, 2H), 7.70 – 7.63 (m, 1H), 7.63 – 7.59 (m, 1H), 7.53 – 7.47 (m, 1H), 7.38 – 7.28 (m, 2H), 7.18 – 7.12 (m, 2H), 4.30 (s, 2H).

^{13}C NMR (76 MHz, $CDCl_3$) δ 155.7, 149.5, 148.5, 143.3, 125.6, 125.0, 123.0, 121.1, 119.8, 115.7, 111.9, 25.1.

7,10-Dimethyl-1,2,3,4-tetrahydro-5*H*-benzo[*c*]furo[3,2-*g*]chromen-5-one 4q

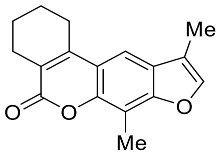

4q was prepared according to a literature[31] procedure starting with 2-methylbenzene-1,3-diol (1.1 g, 8.6 mmol, 1.0 equiv.). Over 4 steps, after purification by flash chromatography (Pentane/EtOAc = 10/1), the desired compound **4q** was obtained as a white solid (550 mg, 24% yield over 4 steps). The analytic data are in agreement with the reported literature data.[32]

^{1}H NMR (400 MHz, $CDCl_3$) δ 7.48 (s, 1H), 7.45 (q, J = 1.4 Hz, 1H), 2.90 – 2.85 (m, 2H), 2.63 – 2.59 (m, 2H), 2.58 (s, 3H), 2.26 (d, J = 1.3 Hz, 3H), 1.93 – 1.86 (m, 2H), 1.86 – 1.79 (m, 2H).

^{13}C NMR (101 MHz, $CDCl_3$) δ 162.5, 155.2, 148.3, 147.9, 142.7, 125.3, 121.4, 116.6, 116.1, 110.4, 109.4, 25.9, 24.3, 22.0, 21.8, 8.7, 8.2.

3-Methylbenzofuran-5-yl 3-(4,5-diphenyloxazol-2-yl)propanoate 4r

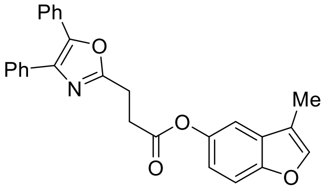

To an oven-dried Schlenk tube equipped with a stir bar, 3-methylbenzofuran-5-ol (327 mg, 1.50 mmol, 1.0 equiv.), oxaprozin (440 mg, 1.50 mmol, 1.0 equiv.), DCC (340 mg, 1.65 mmol, 1.1 equiv.) and DMAP (37 mg, 0.30 mmol, 0.2 equiv.) were dissolved in DCM (7.5 mL) under argon. The reaction mixture was stirred at room temperature overnight. After completion, the reaction mixture was washed with water and extracted with DCM. The organic phase was concentrated and the resulting residue was purified by flash column chromatography (*n*-pentane/EtOAc = 20/1) to give **4r** as a colourless oil (590 mg, 93%).

TLC: 0.18 (*n*-pentane/EtOAc = 10/1).

^{1}H NMR (400 MHz, $CDCl_3$) δ 7.70 – 7.65 (m, 2H), 7.62 – 7.58 (m, 2H), 7.43 – 7.32 (m, 8H), 7.25 (d, J = 2.4 Hz, 1H), 7.00 (dd, J = 8.8, 2.4 Hz, 1H), 3.38 – 3.30 (m, 2H), 3.24 – 3.16 (m, 2H), 2.15 (d, J = 1.3 Hz, 3H).

^{13}C NMR (101 MHz, $CDCl_3$) δ 171.4, 161.7, 153.0, 146.1, 145.7, 142.9, 135.3, 132.6, 129.9, 129.1, 128.8, 128.72, 128.67, 128.2, 128.0, 126.7, 117.9, 116.1, 112.2, 111.9, 31.5, 23.8, 7.9.

HRMS (ESI) m/z: [M+Na$^+$] Calculated for $C_{27}H_{21}NO_4Na$: 446.13628; Found 446.13612.

IR (neat): 1756 (s), 1558 (w), 1506 (w), 1455 (m), 1200 (w), 1166 (s), 1139 (s), 1090 (w), 962 (w), 764 (s), 694 (s).

3-Methylbenzofuran-5-yl 2-(1-(4-chlorobenzoyl)-5-methoxy-2-methyl-1H-indol-3-yl)acetate 4s

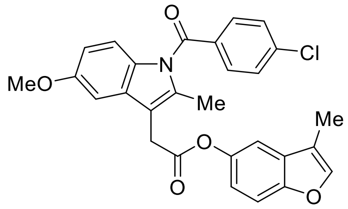

To an oven-dried Schlenk tube equipped with a stir bar, 3-methylbenzofuran-5-ol (327 mg, 1.50 mmol, 1.0 equiv.), indometacin (537 mg, 1.50 mmol, 1.0 equiv.), DCC (340 mg, 1.65 mmol, 1.1 equiv.) and DMAP (37 mg, 0.30 mmol, 0.2 equiv.) were dissolved in DCM (7.5 mL) under argon. The reaction mixture was stirred at room temperature overnight. After completion, the reaction mixture was washed with water and extracted with DCM. The organic phase was concentrated and the resulting residue was purified by flash column chromatography (*n*-pentane/EtOAc = 20/1) to give **4s** as a yellow solid (658 mg, 90%).

TLC: 0.18 (*n*-pentane/EtOAc = 10/1).

^{1}H NMR (400 MHz, $CDCl_3$) δ 7.71 – 7.65 (m, 2H), 7.51 – 7.45 (m, 2H), 7.44 – 7.38 (m, 2H), 7.21 (d, *J* = 2.4 Hz, 1H), 7.09 (d, *J* = 2.5 Hz, 1H), 6.96 – 6.89 (m, 2H), 6.71 (dd, *J* = 9.0, 2.5 Hz, 1H), 3.93 (s, 2H), 3.85 (s, 3H), 2.47 (s, 3H), 2.19 (d, *J* = 1.3 Hz, 3H).

^{13}C NMR (101 MHz, $CDCl_3$) δ 170.1, 168.5, 156.3, 153.0, 146.2, 143.0, 139.5, 136.4, 134.0, 131.4, 131.0, 130.7, 129.9, 129.3, 117.8, 116.1, 115.2, 112.3, 112.1, 112.0, 111.9, 101.4, 55.9, 30.7, 13.6, 8.0.

HRMS (ESI) m/z: [M+Na$^+$] Calculated for $C_{28}H_{22}NO_5ClNa$: 510.10787; Found 510.10743.

IR (neat): 1750 (w), 1684 (m), 1475 (s), 1454 (s), 1356 (m), 1314 (s), 1218 (s), 1163 (s), 1128 (s), 1088 (s), 1066 (m), 926 (w), 833 (w), 803 (w), 754 (m), 737 (w).

Mp: 161-162 °C.

- **Synthesis of other starting materials**

4-Nitrosomorpholine

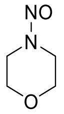

A suspension of $NaNO_2$ (6.90 g, 100 mmol, 2.0 equiv.), oxalic acid dihydrate (12.6 g, 100 mmol, 2.0 equiv.) and morpholine (4.4 mL, 50 mmol, 1.0 equiv.) in DCM (100 mL) was stirred vigorously at room temperature for 5 h. To the reaction mixture, silica gel, anhydrous sodium sulphate and *n*-pentane were added, the undesired precipitate was removed by filtration and washed with *n*-pentane/DCM (100 mL, 1:1). The solvent was evaporated and the residue was subjected to a short column chromatography (*n*-pentane/EtOAc = 5/1) to give 4-nitrosomorpholine as a yellow oil (4.5 g, 78% yield).

^{1}H NMR (300 MHz, $CDCl_3$) δ 4.28 (t, 2H), 3.93-3.80 (m, 4H), 3.65 (t, *J* = 5.3 Hz, 2H).

^{13}C NMR (76 MHz, $CDCl_3$) δ 67.4, 66.0, 50.1, 40.5.

4-(Nitroso-^{15}N)morpholine

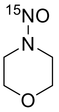

A suspension of $Na^{15}NO_2$ (170 mg, 2.40 mmol, 1.0 equiv.), oxalic acid dihydrate (0.65 g, 4.8 mmol, 2.0 equiv.) and morpholine (0.21 mL, 2.4 mmol, 1.0 equiv.) in DCM (2 mL) was stirred vigorously at room temperature for 5 h. To the reaction mixture, silica gel, anhydrous sodium sulphate and *n*-pentane were added, the undesired precipitate was removed by filtration and washed with *n*-pentane/DCM (100 mL, 1:1). The solvent was evaporated and the residue was subjected to a short column chromatography (*n*-pentane/EtOAc = 3/1) to give 4-(nitroso-^{15}N)morpholine as a yellow oil (170 mg, 64% yield).

TLC: 0.30 (*n*-pentane/EtOAc = 20/7).

^{1}H NMR (300 MHz, $CDCl_3$) δ 4.33 – 4.21 (m, 2H), 3.94 – 3.79 (m, 4H), 3.65 (dd, J = 5.7, 4.5 Hz, 2H).

^{13}C NMR (76 MHz, $CDCl_3$) δ 67.4, 66.0, 50.1 (d, J = 6.1 Hz), 40.4 (d, J = 1.9 Hz).

HRMS (ESI) m/z: [M+Na+] Calculated for $C_4H_8{}^{15}NO_2Na$: 140.04593; Found 140.04484.

IR (neat): 1716 (w), 1699 (m), 1684 (w), 1653 (m), 1558 (s), 1541 (s), 1521 (m), 1507 (s), 1473 (m), 1340 (w), 1280 (w), 1153 (w), 1107 (m), 992 (s).

The ratio of ^{15}N labeling: >95%.

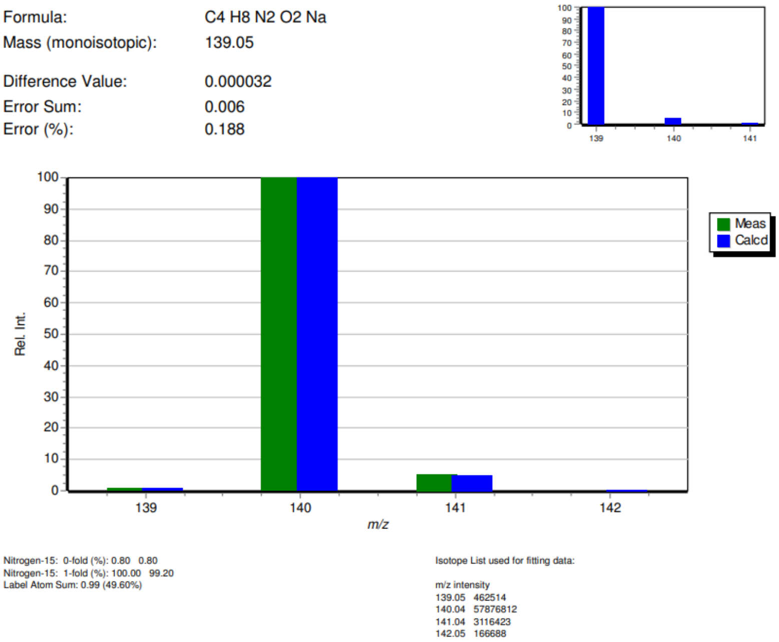

tert-Butyl ((mesitylsulfonyl)oxy)carbamate

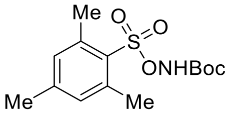

tert-Butyl ((mesitylsulfonyl)oxy)carbamate was prepared according to a literature[33] with *N*-Boc-hydroxylamin (1.33 g, 10.0 mmol, 1.0 equiv.). After purification by recrystallization (toluene/*n*-hexane v/v 1/4), the desired compound was obtained as a colorless solid (1.6 g, 51% yield). The analytical data are in agreement with the reported literature data.[33]

^{1}H NMR (300 MHz, $CDCl_3$) δ 7.61 (s, 1H), 6.99 (s, 2H), 2.67 (s, 6H), 2.32 (s, 3H), 1.31 (s, 9H).

^{13}C NMR (76 MHz, $CDCl_3$) δ 154.3, 144.5, 142.1, 131.8, 128.7, 83.9, 27.9, 23.3, 21.2.

3. Detailed optimization of reaction conditions

Table S1: Light sources screening

To an oven-dried 10 mL Schlenk tube equipped with a stir bar, Boc-**1a** (23 mg, 0.10 mmol, 1.0 equiv.) was dissolved in dimethoxyethane DME (1 mL) under argon. *N*-Nitrosomorphline (35 mg, 0.30 mmol, 3.0 equiv.) and *p*-toluenesulfonic acid monohydrate TsOH•H_2O (38 mg, 0.20 mmol, 2.0 equiv.) were added to the mixture at 0 °C in an ice-water bath. The reaction was irradiated for 48 h at room temperature. Subsequently, saturated $NaHCO_3$ aqueous solution (1 mL) was added and the mixture was extracted with EtOAc (3×5 mL). The combined organic phase was washed with brine and dried over anhydrous Na_2SO_4. Then the solvent was removed under reduced pressure. Dibromomethane (7 μL, 0.1 mmol, 1.0 equiv.) was added as an internal standard, the mixture was dissolved in $CDCl_3$, and a ^{1}H NMR spectrum was recorded. The yield of Boc-**I** was determined by ^{1}H NMR integration relative to the internal standard (standard: δ = 4.93 ppm; typic peak of Boc-**I**: δ = 8.24 (d, *J* = 8.6 Hz, 1H)). Boc-**1a** was synthesized according to a literature procedure and the analytical data are in agreement with the reported literature data.[34]

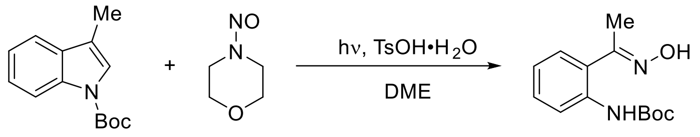

Boc-**1a** Boc-**I**

Entry	Light sources	NMR yield %
1	45 W 456 nm	28%
2	18 W 405 nm	52%
3	40 W 390 nm	38%
4	3 W 415 nm	58%
5	3 W 365 nm	48%

Table S2: Screening of the protecting group

To an oven-dried 10 mL Schlenk tube equipped with a stir bar, protected indole (0.10 mmol, 1.0 equiv.) was dissolved in dimethoxyethane DME (1 mL) under argon. *N*-Nitrosomorphline (35 mg, 0.30 mmol, 3.0 equiv.) and *p*-toluenesulfonic acid monohydrate TsOH•H_2O (38 mg, 0.20 mmol, 2.0 equiv.) were added to the mixture at 0 ºC in an ice-water bath. The reaction was irradiated with 3 W 415 nm LEDs for 48 h at room temperature. Subsequently, saturated aqueous $NaHCO_3$ solution (1 mL) was added and the mixture was extracted with EtOAc (3×5 mL). The combined organic phase was washed with brine and dried over anhydrous Na_2SO_4. Then, the solvent was removed under reduced pressure. Dibromomethane (7 µL, 0.1 mmol, 1.0 equiv.) was added as an internal standard, the mixture was dissolved in $CDCl_3$, and a ^1H NMR spectrum was recorded.

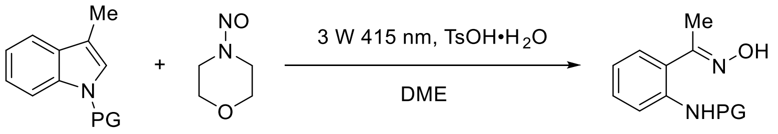

Entry	Protecting group	NMR yield %
1	Moc (methoxycarbonyl)	66%
2	Fmoc (fluorenylmethoxycarbonyl)	58%
3	Acetyl	54%
4	H	n.d.

Table S3: Acid screening, stoichiometry, and solvent screening

To an oven-dried 10 mL Schlenk tube equipped with a stir bar, Moc-**1a** (19 mg, 0.10 mmol, 1.0 equiv.) was dissolved in the given solvent (1 mL) under argon. *N*-Nitrosomorphline and acid were added to the mixture at 0 °C in an ice-water bath. The reaction was irradiated with 3 W 415 nm LEDs for 48 h at room temperature. Subsequently, saturated $NaHCO_3$ aqueous solution (1 mL) was added and the mixture was extracted with EtOAc (3×5 mL). The combined organic phase was washed with brine and dried over anhydrous Na_2SO_4. Then, the solvent was removed under reduced pressure. Dibromomethane (7 μL, 0.1 mmol, 1.0 equiv.) was added as an internal standard. The mixture was dissolved in $CDCl_3$, and a ^1H NMR spectrum was recorded.

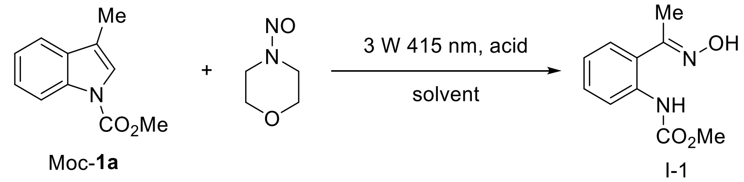

Entry	Acid	Solvent	Equivalents of *N*-nitrosomorphline	NMR yield %
1	MsOH (2.0 equiv.)	DME	3.0	53
2	TFE (2.0 equiv.)	DME	3.0	29
3	oxalic acid (2.0 equiv.)	DME	3.0	44
4	HCl (2.0 equiv.)	DME	3.0	53
5	TfOH (2.0 equiv.)	DME	3.0	18
6	TsOH•H_2O (2.5 equiv.)	DME	3.0	44
7	TsOH•H_2O (1.5 equiv.)	DME	3.0	77
8	TsOH•H_2O (1.5 equiv.)	DME	2.0	74
9	TsOH•H_2O (1.5 equiv.)	DME	1.5	65
10	TsOH•H_2O (1.5 equiv.)	MeOH	3.0	38
11	TsOH•H_2O (1.5 equiv.)	EtOAc	3.0	78
12	TsOH•H_2O (1.5 equiv.)	DCM	3.0	68
13	TsOH•H_2O (1.5 equiv.)	Acetone	3.0	75
14	TsOH•H_2O (1.5 equiv.)	DMF	3.0	50

4. Reactions with oxime intermediates

➢ **Indazole synthesis from oxime**

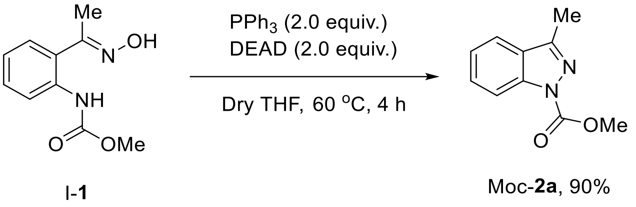

According to a literature procedure[35], oxime I-**1** (63 mg, 0.3 mmol, 1.0 equiv.) and PPh_3 (158 mg, 0.600 mmol, 2.0 equiv.) were dissolved in dry THF (3 mL, 1 M) under argon. The reaction mixture was cooled to 0 °C using an ice-water bath and DEAD (0.24 mL, 0.60 mmol, 2.0 equiv, 40% in toluene) was added dropwise at 0 °C. Afterwards, the mixture was heated at 60 °C for 4 h. After completion, the solvent was removed with a rotary evaporator under reduced pressure and the residue was purified by column chromatography (SiO_2, *n*-pentane/EtOAc = 10/1) to give Moc-**2a** as a white solid (51.3 mg, 90%).

➢ **Benzimidazole synthesis from oxime**

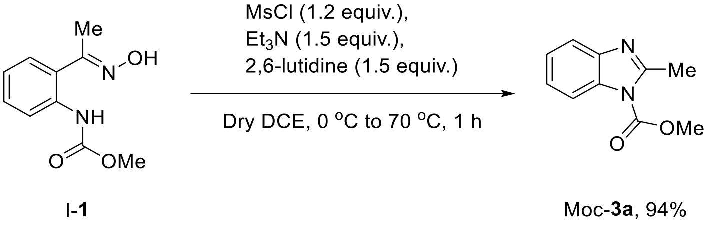

The oxime I-**1** (42 mg, 0.2 mmol, 1.0 equiv.) was dissolved in dry DCE (1 mL) under argon. Triethylamine (41.5 µL, 0.300 mmol, 1.5 equiv.) was added and the mixture was stirred at room temperature for 10 min. Then, MsCl (18.5 µL, 0.240 mmol, 1.2 equiv.) in 1 mL DCE was added to the solution dropwise at 0 °C using an ice-water bath. The reaction mixture was stirred at 0 °C for 30 min followed by the addition of 2,6-lutidine (35.0 µL, 0.300 mmol, 1.5 equiv.). Subsequently, the reaction was heated at 70 °C for 1 h. After completion, the solvent was removed with a rotary evaporator under reduced pressure and the residue was purified by column chromatography (SiO_2, *n*-pentane/EtOAc = 7/1) to give benzimidazoles as a yellow solid (35.8 mg, 94%).

➢ **Benzisoxazole synthesis from oxime**

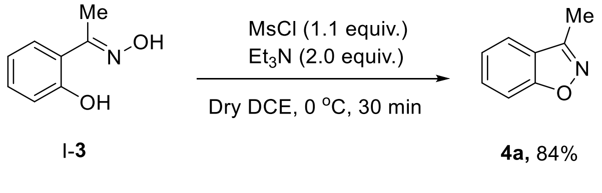

The oxime I-**3** (45.3 mg, 0.300 mmol, 1.0 equiv.) was dissolved in dry DCE (2 mL) under argon. Triethylamine (83.4 µL, 0.600 mmol, 2.0 equiv.) was added and the mixture was stirred at room temperature for 10 min. Then, MsCl (25.5 µL 0.33 mmol, 1.1 equiv.) in 1 mL DCE was added to the solution dropwise at 0 °C using an ice-water bath and the reaction mixture was stirred at 0 °C for 30 min. After completion, the solvent was removed with a rotary evaporator under reduced pressure and the residue was purified by column chromatography (SiO_2, *n*-pentane/Et_2O = 20/1) to give **4a** as a colourless oil (33.5 mg, 84%, **4a** is volatile).

- **Benzoxazole synthesis from oxime**

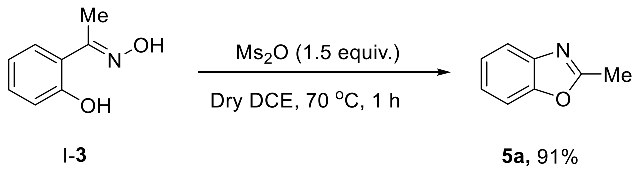

The oxime I-**3** (45.3 mg, 0.300 mmol, 1.0 equiv.) was dissolved in dry DCE (2 mL) under argon. Methanesulfonic anhydride (78.4 mg, 0.450 mmol, 1.5 equiv.) in 1 mL DCE was added to the solution dropwise at 0 °C using an ice-water bath. Then the reaction mixture was heated at 70 °C for 1 h. After completion, 5 mL saturated $NaHCO_3$ aqueous solution was added and the mixture was extracted with DCM (3×10 mL). The combined organic phase was washed with brine (10 mL) and dried over anhydrous Na_2SO_4. The solvent was removed with a rotary evaporator under reduced pressure and the residue was purified by column chromatography (SiO_2, *n*-pentane/Et_2O = 20/1) to give **5a** as a colourless oil (36.3 mg, 91%, **5a** is volatile).

5. General procedure

5.1 General procedure III: skeletal editing of indoles to give indazoles through radical pathway (A)

Step 1: Under argon, Moc-indole (1.0 equiv.) was dissolved in EtOAc (0.1 M) in an oven-dried 10 mL Schlenk tube equipped with a stir bar. The reaction mixture was cooled to 0 °C using an ice-water bath. *N*-nitrosomorpholine (3.0 equiv.) and TsOH•H_2O (1.5 equiv.) were added sequentially. Then, the reaction mixture was irradiated with 3 W 415 nm LEDs for 48 h at room temperature. After completion, saturated $NaHCO_3$ aqueous solution was added and the mixture was extracted with EtOAc (3 times). The combined organic phase was washed with brine and dried over anhydrous Na_2SO_4. After filtration, the solvent was removed under reduced pressure and the residue was passed through a short column (silica, ca. 5 cm, *n*-pentane/EtOAc) to give the crude or pure oxime intermediate. If it is crude, dibromomethane (7 µL, 0.1 mmol, 1.0 equiv.) was added as an internal standard. The mixture was dissolved in $CDCl_3$, and a ^1H NMR spectrum was recorded. The yield of oxime intermediate was determined by ^1H NMR integration relative to the internal standard.

Step 2: The condensed oxime intermediate and PPh_3 (2.0 equiv. based on the yield of oxime intermediate) were dissolved in dry THF (0.1 M) under argon. The reaction mixture was cooled to 0 °C using an ice-water bath and DEAD (2.0 equiv. 40 wt.% in toluene, based on the yield of oxime intermediate) was added dropwise at 0 °C. Afterwards, the mixture was heated at 60 °C for 4 h. After completion, the solvent was removed with a rotary evaporator under reduced pressure and the residue was purified by column chromatography (SiO_2, *n*-pentane/EtOAc) to give the indazoles. If necessary, further purification was accomplished by preparative TLC plates or MPLC.

5.2 General procedure IV: skeletal editing of indoles to give benzimidazoles through radical pathway (A)

Step 1: The oxime intermediate was prepared according to the first step of general procedure III.

Step 2: The oxime intermediate was dissolved in dry DCE under argon. Triethylamine (1.5 equiv. based on the yield of oxime intermediate) was added and the mixture was stirred at room temperature for 10 min. Then MsCl (1.2 equiv. based on the yield of oxime intermediate) in DCE (1 mL) was added to the solution dropwise at 0 °C using an ice-water bath. The reaction mixture was stirred at 0 °C for 30 min followed by the addition of 2,6-lutidine (1.5 equiv. based on the yield of oxime intermediate). Subsequently, the reaction was heated at 70 °C for 1 h. After completion, the solvent was removed with a rotary evaporator under reduced pressure and the residue was purified by column chromatography (SiO_2, *n*-pentane/EtOAc) to give the benzimidazole.

5.3 General procedure V: skeletal editing of benzofurans to give benzisoxazoles through radical pathway (A)

Step 1: Under argon, benzofuran (1.0 equiv.) was dissolved in EtOAc (0.1 M) in an oven-dried 10 mL Schlenk tube equipped with a stir bar. The reaction mixture was cooled to 0 °C using an ice-water bath. *N*-nitrosomorpholine (3.0 equiv.) and TsOH•H_2O (1.5 equiv.) were added sequentially. Then, the reaction was irradiated with 3 W 415 nm LEDs for 3 h at room temperature. After completion, saturated $NaHCO_3$ aqueous solution was added and the mixture was extracted with EtOAc (3 times). The combined organic phase was washed with brine and dried over anhydrous Na_2SO_4. After filtration, the solvent was removed under reduced pressure and the residue was directly used for the next step.

Step 2: The oxime intermediate was dissolved in dry DCE under argon. Triethylamine (2.0 equiv.) was added and the

mixture was stirred at room temperature for 10 min. Then, MsCl (1.1 equiv.) in DCE was added to the solution dropwise at 0 °C using an ice-water bath and the reaction mixture was stirred at 0 °C for 30 min. After completion, the solvent was removed with a rotary evaporator under reduced pressure and the residue was purified by column chromatography (SiO_2, *n*-pentane/EtOAc) to give the benzisoxazole.

5.4 General procedure VI: skeletal editing of benzofurans to give benzoxazoles through radical pathway (A)

Step 1: The oxime intermediate was prepared according to the first step of general procedure V.

Step 2: The oxime intermediate was dissolved in dry DCE under argon. Methanesulfonic anhydride (1.5 equiv.) in DCE was added to the solution dropwise at 0 °C using an ice-water bath. Then, the reaction mixture was heated at 70 °C for 1 h. After completion, saturated $NaHCO_3$ aqueous solution was added and the mixture was extracted with DCM (3 times). The combined organic phase was washed with brine and dried over anhydrous Na_2SO_4. After filtration, the solvent was removed with a rotary evaporator under reduced pressure and the residue was purified by column chromatography (SiO_2, *n*-pentane/EtOAc) to give the benzoxazole.

5.5 General procedure VII: skeletal editing of indoles to give indazoles through ionic pathway (B)

Step 1: Under air, a solution of indole (1.0 equiv.) in MeOH (0.05 M-0.2 M) was added dropwise to a solution of sodium metaperiodate $NaIO_4$ (2.0-4.0 equiv.) in water (0.05 M-0.2 M) at 0 °C using an ice-water bath. (*Other oxidants were also used for oxidative cleavage. For example, mCPBA performed in indoles* ***1j*** *and* ***1k****. Oxone were applied for indole* ***1o****. Detailed procedure and conditions see part* ***6*** *below*). Then, the mixture was stirred at room temperature and monitored by TLC. After consumption of the starting material, the mixture was poured into water and extracted with DCM (3 times). The combined organic phase was washed with brine and dried over anhydrous Na_2SO_4. After filtration, the solvent was removed with a rotary evaporator under reduced pressure and the residue was dissolved in MeOH. *Deformylation under the acidic condition:* conc. HCl (1.5 equiv.) was dropped into the reaction mixture and refluxed at 70 °C for 1 h (***Note***: for indole **1r**, **1t**, **1v** and **1w**, the reaction mixture was stirred at room temperature for 24 h). After cooling down to room temperature, the reaction solution is neutralized with 1 M K_2CO_3 solution to PH = 7. The residue was poured into the water and extracted with DCM three times. The organic phase was washed with water, brine and dried over anhydrous Na_2SO_4 and filtered. The solvent was removed and the residue was purified by column chromatography (SiO_2, *n*-pentane/EtOAc) to give the 2-aminophenone. *Deformylation under the basic condition:* 10% NaOH aqueous solution was added and the mixture was heated at 70 °C for 1 h. After cooling to room temperature, the reaction mixture was extracted with DCM three times and washed with water, brine and dried over anhydrous Na_2SO_4 and filtered. The solvent was removed and the residue was purified by column chromatography (SiO_2, *n*-pentane/EtOAc) to give the 2-aminophenone.

Step 2: To a mixture of the 2-aminophenone (1.0 equiv.) and *tert*-butyl ((mesitylsulfonyl)oxy)carbamate (1.5 equiv.), hexafluoroisopropanol HFIP (0.2 M) was added, and the resulting reaction mixture was stirred at 45 °C for 15 h. After cooling down to room temperature, the solvent was removed and the residue was dissolved in EtOAc and washed with saturated aqueous $NaHCO_3$ solution. The organic phase was collected, concentrated and the residue was purified by column chromatography (SiO_2, *n*-pentane/EtOAc) to give the indazole.

5.6 General procedure VIII: skeletal editing of indoles to give benzimidazoles through ionic pathway (B)

Step 1: 2-Aminophenones were prepared according to the step 1 of general procedure VII.

Step 2: To a solution of 2-aminophenone (1 equiv.) in DCM (0.1 M), pyridine (7.0 equiv.) and DMAP (0.2 equiv.) were added followed by TsCl (1.5 equiv.) at 0 °C. The reaction mixture was then stirred at room temperature for 12 h. Then, the reaction was quenched through addition of water and extracted with DCM three times. The collected organic phase was washed with 1 N HCl and dried over Na_2SO_4 and filtered. After removing the solvent, the Ts-protected aminoaryl ketone was directly used for next step without further purification. *Ketimine as intermediate for benzimidazole synthesis:* An oven-dried vial was charged with the aminoaryl ketone and a solution of 7 M NH_3 in MeOH (0.25 M) was added. The vial was capped and the reaction mixture was stirred at room temperature for 24 h. The solvent was removed to give the ketimine as a yellow solid. The crude ketimine was dissolved in methanol (0.5 M) and $PhI(OAc)_2$ (1.5 equiv.) was added to the mixture. After stirring at room temperature for 1 h, the solvent was removed and the residue was purified by column chromatography (SiO_2, *n*-pentane/EtOAc) to give the benzimidazole. *Oxime as intermediate for benzimidazole synthesis:* To a solution of aminoaryl ketone in methanol (0.02 M), NH_2OH •HCl (10 equiv.) and NaOAc (12 equiv.) were added. Then the reaction mixture was refluxed for 10 h. After cooling to room temperature, water was added to quench the reaction. The mixture was extracted with DCM three times and the combined organic layer was washed with brine, dried over Na_2SO_4, and concentrated under reduced pressure to get crude oxime intermediate. The approximate yield was determined *via* weighing the crude product. Based on this result, respective equivalent of the reaction will be chosen. The oxime intermediate was dissolved in dry DCE under argon. Triethylamine (1.5 equiv.) was added and the mixture was stirred at room temperature for 10 min. Then MsCl (1.2 equiv.) in DCE was added to the solution dropwise at 0 °C using an ice-water bath. The reaction mixture was stirred at 0 °C for 30 min followed by the addition of 2,6-lutidine (1.5 equiv.). Subsequently, the reaction was heated at 70 °C for 1 h. After completion, the solvent was removed with a rotary evaporator under reduced pressure and the residue was purified by column chromatography (SiO_2, *n*-pentane/EtOAc) to give the benzimidazole.

5.7 General procedure IX: skeletal editing of benzofurans to give benzisoxazoles through ionic pathway (B)

Step 1: A solution of 2,3-disubstitued benzofuran (1.0 equiv.) in dry DCM (0.02 M) was cooled to 0 °C using an ice-water bath. Silica gel (2.4 g/mmol) and PCC (5.4 equiv.) were then added sequentially. Then, the obtained dark mixture was allowed to warm to room temperature and stirring was continued for 48 h (monitored by TLC). The reaction mixture was then filtered through a pad of silica, washed with DCM and Et_2O, and the filtrate was concentrated in vacuo. The corresponding product was dissolved in 7 M NH_3 in MeOH (0.2 M) and the mixture was stirred at room temperature or heating at 70 °C for 24 h. Then, the solvent was removed with a rotary evaporator under reduced pressure to give the crude *o*-hydroxyl ketimine.

Step 2: A mixture of the crude *o*-hydroxyl ketimine, NCS (1.5 equiv.) and base (2.0 equiv. K_2CO_3 or ^{t}BuOK) in THF (0.2 M) was stirred at ambient temperature for 24 h. Analysis by TLC of the reaction mixture showed completion of the reaction. Et_2O and water was added to the reaction mixture and the organic layer was separated, over anhydrous Na_2SO_4 and filtered. The organic phase was collected, concentrated and the residue was purified by column chromatography (SiO_2, *n*-pentane/EtOAc) to give the benzisoxazoles.

5.8 General procedure X: skeletal editing of benzofurans to give benzoxazoles through ionic pathway (B)

Step 1: *o*-Hydroxyl ketimines were prepared according to the step 1 of general procedure IX.

Step 2: A solution of crude *o*-Hydroxyl ketimines in isopropyl alcohol (0.2 M) was cooled to 0 °C using an ice-water

bath. The aqueous 10% NaOCl (3.0 equiv.) was added dropwise keeping the reaction temperature below 10 ^oC. The yellow colour faded and the reaction was monitored by TLC (around 1 h). The mixture was allowed to warm to ambient temperature and was then extracted with DCM three times. The organic phase was washed with water, brine and dried over anhydrous Na_2SO_4 and filtered. The solvent was removed and the residue was purified by column chromatography (SiO_2, *n*-pentane/EtOAc) to give the benzoxazoles.

6. Spectral data of products

Methyl (*E*)-(2-(1-(hydroxyimino)ethyl)phenyl)carbamate (I-1)

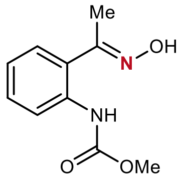

Oxime intermediate I-**1** could be isolated in the first step of general procedure III using Moc-**1a** (56.8 mg, 0.300 mmol, 1.0 equiv.), *N*-nitrosomorpholine (105 mg, 0.900 mmol, 3.0 equiv.) and TsOH•H_2O (86 mg, 0.45 mmol, 1.5 equiv.) in EtOAc (3 mL). After purification by flash chromatography (*n*-pentane/EtOAc = 10/1), I-**1** was obtained as a white solid (46.8 mg, 75%).

TLC: 0.60 (*n*-pentane/EtOAc = 10/1).

^{1}H NMR (300 MHz, $CDCl_3$) δ 10.21 (s, 1H), 8.33 – 8.22 (m, 1H), 7.44 (dd, *J* = 8.0, 1.6 Hz, 1H), 7.34 (ddd, *J* = 8.6, 7.3, 1.6 Hz, 1H), 7.07 (ddd, *J* = 8.0, 7.3, 1.3 Hz, 1H), 3.76 (s, 3H), 2.33 (s, 3H). (N-H was not detected).

^{13}C NMR (76 MHz, $CDCl_3$) δ 158.1, 154.6, 137.0, 130.0, 128.6, 122.8, 122.5, 119.9, 52.3, 13.3.

HRMS (ESI) m/z: [M+Na^+] Calculated for $C_{10}H_{12}N_2O_3Na$ 231.07401; Found 231.07376.

IR (neat): 1719 (m), 1652 (m), 1588 (m), 1532 (s), 1507 (m), 1457 (s), 1355 (w), 1315 (w), 1241 (s), 1213 (s), 1063 (s), 743 (s), 715 (m), 601 (w), 518 (w).

Mp: 115-116 °C.

1-(2-Aminophenyl)ethan-1-one (I-2)

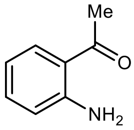

2-Aminophenone intermediate I-**2** could be isolated in the first step of general procedure VII using **1a** (65.6 mg, 0.500 mmol, 1.0 equiv.) in MeOH (2.5 mL) with $NaIO_4$ (214 mg, 1.00 mmol, 2.0 equiv.) in water (2.5 mL) for oxidative cleavage. Deformylation was conducted under basic conditions using 10% NaOH aqueous solution (1 mL) in MeOH (5 mL) at 70 °C for 1 h. After purification by flash chromatography (*n*-pentane/EtOAc = 10/1), I-**2** was obtained as a yellow oil (50.7 mg, 75%).

^{1}H NMR (400 MHz, $CDCl_3$) δ 7.79 – 7.60 (m, 1H), 7.31 – 7.15 (m, 1H), 6.66 – 6.60 (m, 2H), 6.27 (s, 2H), 2.57 (s, 3H).

^{13}C NMR (101 MHz, $CDCl_3$) δ 200.9, 150.4, 134.5, 132.2, 118.4, 117.3, 115.9, 28.0.

Analytic data are in agreement with those reported in the literature.[36]

(*E*)-1-(2-Hydroxyphenyl)ethan-1-one oxime (I-3)

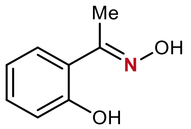

Oxime intermediate I-**3** could be isolated in the first step of general procedure V using **4a** (39.6 mg, 0.300 mmol, 1.0 equiv.), *N*-nitrosomorpholine (105 mg, 0.900 mmol, 3.0 equiv.) and TsOH•H_2O (86 mg, 0.45 mmol, 1.5 equiv.) in EtOAc (3 mL). After purification by flash chromatography (*n*-pentane/EtOAc = 20/1), I-**3** was obtained as a white solid (36.5 mg, 92%).

^{1}H NMR (400 MHz, $CDCl_3$) δ 11.51 (s, 1H), 7.79 (s, 1H), 7.45 (dd, *J* = 8.0, 1.7 Hz, 1H), 7.31 – 7.25 (m, 1H), 7.02 – 6.97 (m, 1H), 6.95 – 6.88 (m, 1H), 2.37 (s, 3H).

^{13}C NMR (101 MHz, $CDCl_3$) δ 159.8, 157.6, 131.0, 127.8, 119.4, 118.7, 117.4, 10.9.

Analytic data are in agreement with those reported in the literature.[37]

2-(Imino(phenyl)methyl)phenol (I-4)

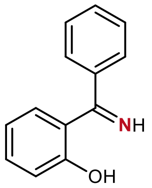

Ketimine intermediate I-**4** generated from the first step of general procedure IX using **4m** (208 mg, 1 mmol, 1.0 equiv.), silica gel (2.4 g), PCC (1.17 g, 5.4 mmol, 5.4 equiv.) in DCM (50 mL) at r.t. for 48 h and subsequent aminolysis with NH_3 (5 mL, 7 M in MeOH) at r.t. for 24 h. I-**4** could be identified by crude **^{1}H** and **^{13}C** NMR without isolation to avoid loss in the purification process. And analytic data are in agreement with those reported in the literature.[38]

^{1}H NMR (300 MHz, $CDCl_3$, crude) δ 14.65 (s, 1H), 9.36 (s, 1H), 7.51 – 7.43 (m, 3H), 7.41 – 7.34 (m, 2H), 7.34 – 7.29 (m, 1H), 7.18 (dd, *J* = 8.0, 1.7 Hz, 1H), 7.01 (dd, *J* = 8.4, 1.2 Hz, 1H), 6.76 – 6.65 (m, 1H).

^{13}C NMR (76 MHz, $CDCl_3$, crude) δ 181.2, 164.3, 138.9, 133.7, 132.3, 130.1, 128.8, 127.4, 118.7, 118.2, 117.6.

Methyl 3-methyl-1*H*-indazole-1-carboxylate Moc-**2a**

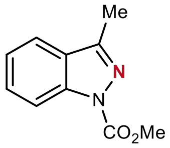

Moc-**2a** was prepared according to the general procedure III using Moc-**1a** (56.8 mg, 0.300 mmol, 1.0 equiv.), *N*-nitrosomorpholine (105 mg, 0.900 mmol, 3.0 equiv.), TsOH•H_2O (86 mg, 0.45 mmol, 1.5 equiv.) in EtOAc (3 mL) and subsequent ring closure with PPh_3 (121 mg, 0.460 mmol), DEAD (0.20 mL, 0.46 mmol, 40 wt.% in toluene) in dry THF (3 mL). After purification by flash chromatography (*n*-pentane/EtOAc = 10/1), Moc-**2a** was obtained as a white solid (36.5 mg, 64%). The NMR yield of oxime intermediate I-**1** was 76% (0.23 mmol).

TLC: 0.38 (*n*-pentane/EtOAc = 5/1).

^{1}H NMR (300 MHz, $CDCl_3$) δ 8.19 (d, *J* = 8.4 Hz, 1H), 7.72 – 7.61 (m, 1H), 7.58 – 7.48 (m, 1H), 7.36 – 7.28 (m, 1H), 4.10 (s, 3H), 2.59 (s, 3H).

^{13}C NMR (76 MHz, $CDCl_3$) δ 151.2, 149.5, 140.5, 129.3, 126.0, 123.8, 120.5, 114.6, 54.4, 12.3.

HRMS (ESI) m/z: [M+Na$^+$] Calculated for $C_{10}H_{10}N_2O_2Na$ 213.06345; Found 213.06348.

IR (neat): 1731 (s), 1443 (s), 1399 (m), 1737 (s), 1353 (w), 1339 (w), 1288 (w), 1241 (s), 1154 (w), 1083 (s), 1041 (w), 760 (s).

Mp: 75-77 °C.

Methyl 2-methyl-1*H*-benzo[*d*]imidazole-1-carboxylate Moc-**3a**

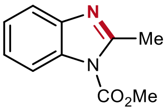

Moc-**3a** was prepared according to the general procedure IV using Moc-**1a** (56.8 mg, 0.300 mmol, 1.0 equiv.), *N*-nitrosomorpholine (105 mg, 0.900 mmol, 3.0 equiv.), $TsOH•H_2O$ (86 mg, 0.45 mmol, 1.5 equiv.) in EtOAc (3 mL) and subsequent ring closure with Et_3N (50.0 µL, 0.460 mmol), 2,6-lutidine (40.1 µL, 0.460 mmol) in dry DCE (2 mL) as well as MsCl (21.5 µL, 0.276 mmol) in dry DCE (1 mL). After purification by flash chromatography (*n*-pentane/EtOAc = 7/1), Moc-**3a** was obtained as a yellow solid (34.8 mg, 61%). The NMR yield of oxime intermediate I-**1** was 76% (0.23 mmol).

TLC: 0.30 (*n*-pentane/EtOAc = 10/3).

^{1}H NMR (300 MHz, $CDCl_3$) δ 7.91 – 7.84 (m, 1H), 7.68 – 7.61 (m, 1H), 7.34 – 7.27 (m, 2H), 4.08 (s, 3H), 2.81 (s, 3H).

^{13}C NMR (76 MHz, $CDCl_3$) δ 153.2, 151.2, 142.3, 132.9, 124.5, 124.5, 119.5, 114.8, 54.3, 18.3.

HRMS (ESI) m/z: [M+Na$^+$] Calculated for $C_{10}H_{10}N_2O_2Na$ 213.06345; Found 213.06337.

IR (neat): 1750 (m), 1558 (m), 1541 (m), 1507 (m), 1457 (s), 1437 (w), 1353 (w), 1336 (w), 1209 (w), 763 (w).

Mp: 57-58 °C.

3-Methyl-1*H*-indazole 2a

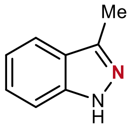

2a was prepared according to the general procedure VII using **1a** (65.6 mg, 0.500 mmol, 1.0 equiv.) in MeOH (2.5 mL) with $NaIO_4$ (214 mg, 1.00 mmol, 2.0 equiv.) in water (2.5 mL) for oxidative cleavage. Deformylation was conducted under basic conditions using 10% NaOH aqueous solution (1 mL) in MeOH (5 mL) at 70 °C for 1 h. The crude 2-aminophenone obtained can be directly used for the second step with $Me_3C_6H_2SO_2ONHBoc$ (236 mg, 0.75 mmol) in HFIP (2.5 mL). After second step's purification by flash chromatography (*n*-pentane/EtOAc = 5/1), **2a** was obtained as a light white solid (39.5 mg, 60%).

^{1}H NMR (300 MHz, $CDCl_3$) δ 10.13 (s, 1H), 7.75 – 7.65 (m, 1H), 7.48 – 7.33 (m, 2H), 7.15 (ddd, *J* = 7.9, 6.7, 1.1 Hz, 1H), 2.64 (s, 3H).

^{13}C NMR (76 MHz, $CDCl_3$) δ 143.4, 141.2, 126.8, 122.8, 120.3, 120.3, 109.9, 12.1.

Analytic data are in agreement with those reported in the literature.[33]

2-Methyl-1-tosyl-1*H*-benzo[*d*]imidazole Ts-**3a**

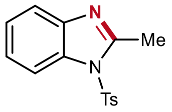

Ts-**3a** was prepared according to the general procedure VIII using **1a** (65.6 mg, 0.500 mmol, 1.0 equiv.) in MeOH (2.5 mL) with $NaIO_4$ (214 mg, 1.00 mmol, 2.0 equiv.) in water (2.5 mL) for oxidative cleavage. Deformylation was conducted under basic conditions use 10% NaOH aqueous solution (1 mL) in MeOH (5 mL) at 70 °C for 1 h. The crude 2-aminophenone obtained can be directly used for the second step *via* ketimine as intermediate with NH_3 (2 mL, 7 M in MeOH) and ring closure by $PhI(OAc)_2$ (242 mg, 0.75 mmol) in MeOH (1 mL). After second step's purification by flash chromatography (*n*-pentane/EtOAc = 10/1), Ts-**3a** was obtained as a light white solid (82.5 mg, 58%).

^{1}H NMR (300 MHz, $CDCl_3$) δ 8.07 – 8.02 (m, 1H), 7.85 – 7.80 (m, 2H), 7.68 – 7.60 (m, 1H), 7.39 – 7.26 (m, 4H), 2.83 (s, 3H), 2.39 (s, 3H).

^{13}C NMR (76 MHz, $CDCl_3$) δ 151.5, 146.1, 141.9, 135.5, 133.3, 130.4, 126.9, 124.8, 124.7, 119.7, 113.5, 21.7, 17.0. Analytic data are in agreement with those reported in the literature.[39]

Methyl 5-methoxy-3-methyl-1*H*-indazole-1-carboxylate Moc-**2b**

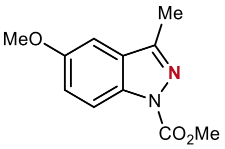

Moc-**2b** was prepared according to the general procedure III using Moc-**1b** (65.8 mg, 0.300 mmol, 1.0 equiv.), *N*-nitrosomorpholine (105 mg, 0.900 mmol, 3.0 equiv.), TsOH•H_2O (86 mg, 0.45 mmol, 1.5 equiv.) in EtOAc (3 mL) and subsequent ring closure with PPh_3 (78.7 mg, 0.300 mmol), DEAD (0.13 mL, 0.30 mmol, 40 wt.% in toluene) in dry THF (3 mL). After purification by flash chromatography (*n*-pentane/EtOAc = 6/1), Moc-**2b** was obtained as a light white solid (30.1 mg, 46%). The NMR yield of oxime intermediate was 50% (0.15 mmol).

TLC: 0.30 (*n*-pentane/EtOAc = 10/3).

^{1}H NMR (300 MHz, $CDCl_3$) δ 8.08 (d, *J* = 9.1 Hz, 1H), 7.17 (dd, *J* = 9.1, 2.5 Hz, 1H), 7.03 – 6.97 (m, 1H), 4.09 (s, 3H), 3.88 (s, 3H), 2.57 (s, 3H).

^{13}C NMR (76 MHz, $CDCl_3$) δ 156.9, 151.3, 149.1, 135.9, 126.9, 119.9, 115.6, 101.1, 56.0, 54.5, 12.5.

HRMS (ESI) m/z: [$M+Na^+$] Calculated for $C_{11}H_{12}N_2O_3Na$ 243.07401; Found 243.07391.

IR (neat): 1728 (m), 1440 (m), 1350 (m), 1330 (w), 1248 (s), 1218 (w), 1075 (s), 1021 (w), 809 (w), 759 (m).

Mp: 102-103 °C.

Methyl 5-methoxy-2-methyl-1H-benzo[d]imidazole-1-carboxylate Moc-**3b**

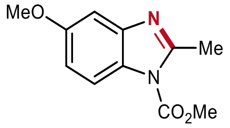

Moc-**3b** was prepared according to the general procedure IV using Moc-**1b** (65.8 mg, 0.300 mmol, 1.0 equiv.), *N*-nitrosomorpholine (105 mg, 0.900 mmol, 3.0 equiv.), TsOH•H_2O (86 mg, 0.45 mmol, 1.5 equiv.) in EtOAc (3 mL) and subsequent ring closure with Et_3N (31.5 µL, 0.23 mmol), 2,6-lutidine (26.8 µL, 0.23 mmol) in dry DCE (2 mL) as well as MsCl (14 µL, 0.18 mmol) in dry DCE (1 mL). After purification by flash chromatography (*n*-pentane/EtOAc = 5/1), Moc-**3b** was obtained as a brown solid (24.6 mg, 38%). The NMR yield of oxime intermediate was 51% (0.15 mmol).

TLC: 0.18 (*n*-pentane/EtOAc = 10/3).

^{1}H NMR (300 MHz, $CDCl_3$) δ 7.75 (d, *J* = 9.0 Hz, 1H), 7.14 (d, *J* = 2.5 Hz, 1H), 6.91 (dd, *J* = 9.0, 2.5 Hz, 1H), 4.08 (s, 3H), 3.85 (s, 3H), 2.81 (s, 3H).

^{13}C NMR (76 MHz, $CDCl_3$) δ 157.4, 153.8, 151.2, 143.3, 127.1, 115.3, 113.1, 102.5, 55.8, 54.3, 18.4.

HRMS (ESI) m/z: [M+Na$^+$] Calculated for $C_{11}H_{12}N_2O_3Na$ 243.07401; Found 243.07368.

IR (neat): 1743 (m), 1541 (m), 1486 (m), 1437 (m), 1359 (m), 1323 (m), 1285 (m), 1198 (m), 1148 (s), 1122 (m), 1018 (m), 943 (w), 846 (w), 817 (m), 765 (m).

Mp: 111-112 °C.

3,4-Dimethyl-1*H*-indazole 2c

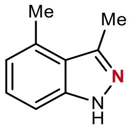

2c was prepared according to the general procedure VII using **1c** (87.1 mg, 0.600 mmol, 1.0 equiv.) in MeOH (3 mL) with $NaIO_4$ (257 mg, 1.20 mmol, 2.0 equiv.) in water (3 mL) for oxidative cleavage. Deformylation was conducted under acidic conditions use conc. HCl (75 µL) in MeOH (6 mL) at 70 °C for 1 h to give 2-aminophenone (60 mg, 67%, 0.4 mmol). The ring closure step was conducted with 2-aminophenone (30 mg, 0.2 mmol) with $Me_3C_6H_2SO_2ONHBoc$ (95 mg, 0.30 mmol) in HFIP (1 mL). After purification by flash chromatography (*n*-pentane/EtOAc = 5/1), **2c** was obtained as a brown solid (7.9 mg, 27%).

TLC: 0.30 (*n*-pentane/EtOAc = 10/3).

^{1}H NMR (300 MHz, $CDCl_3$) δ 7.80 (s, 1H), 7.34 – 7.27 (m, 2H), 7.00 – 6.80 (m, 1H), 2.81 (s, 3H), 2.76 (d, *J* = 1.0 Hz, 3H).

^{13}C NMR (76 MHz, $CDCl_3$) δ 143.6, 141.8, 132.5, 127.4, 121.9, 121.4, 107.7, 19.8, 15.1.

HRMS (ESI) m/z: [M+Na$^+$] Calculated for $C_{13}H_{13}NO_4Na$ 270.07368; Found 270.07353.

IR (neat): 3168 (m), 2958 (w), 1699 (m), 1653 (m), 1558 (m), 1541 (m), 1521 (m), 1507 (m), 1457 (m), 1343 (s), 1162 (w), 1099 (w), 850 (w), 762 (m), 740 (s).

Mp: 123-124 °C.

2,4-Dimethyl-1-tosyl-1*H*-benzo[*d*]imidazole Ts-**3c**

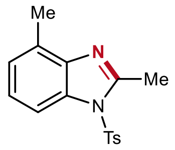

Ts-**3c** was prepared according to the general procedure VIII using **1c** (87.1 mg, 0.600 mmol, 1.0 equiv.), in MeOH (3 mL) with $NaIO_4$ (257 mg, 1.20 mmol, 2.0 equiv.) in water (3 mL) for oxidative cleavage. Deformylation was conducted under acidic conditions use conc. HCl (75 µL) in MeOH (6 mL) at 70 °C for 1 h to give 2-aminophenone (60 mg, 67%, 0.4 mmol). The ring closure step was conducted with 2-aminophenone (30 mg, 0.2 mmol) *via* ketimine as intermediate. After second step's purification by flash chromatography (*n*-pentane/EtOAc = 10/1), Ts-**3c** was obtained as a brown solid (23 mg, 26%).

TLC: 0.30 (*n*-pentane/EtOAc = 10/3).

^{1}H NMR (400 MHz, $CDCl_3$) δ 7.84 (d, *J* = 8.2 Hz, 1H), 7.81 – 7.76 (m, 2H), 7.30 – 7.25 (m, 2H), 7.23 (t, *J* = 7.8 Hz, 1H), 7.14 – 7.10 (m, 1H), 2.82 (s, 3H), 2.57 (s, 3H), 2.38 (s, 3H).

^{13}C NMR (101 MHz, $CDCl_3$) δ 150.6, 146.0, 141.1, 135.7, 133.0, 130.3, 129.8, 126.9, 125.3, 124.7, 111.1, 21.8, 17.1, 16.6.

HRMS (ESI) m/z: [M+Na$^+$] Calculated for $C_{16}H_{16}N_2O_2SNa$ 323.08357; Found 323.08250.

IR (neat): 1598 (w), 1551 (w), 1369 (s), 1282 (w), 1262 (m), 1179 (s), 1163 (s), 1108 (s), 1001 (m), 811 (m), 758 (m), 703 (w), 678 (s), 647 (w), 578 (s), 543 (s).

Mp: 103-104 °C.

Methyl 3,5-dimethyl-1*H*-indazole-1-carboxylate Moc-**2d**

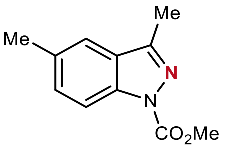

Moc-**2d** was prepared according to the general procedure III using Moc-**1d** (61.0 mg, 0.300 mmol, 1.0 equiv.), *N*-nitrosomorpholine (105 mg, 0.900 mmol, 3.0 equiv.), $TsOH•H_2O$ (86 mg, 0.45 mmol, 1.5 equiv.) in EtOAc (3 mL) and subsequent ring closure with PPh_3 (94.5 mg, 0.360 mmol), DEAD (0.16 mL, 0.36 mmol, 40 wt.% in toluene) in dry THF (3 mL). After purification by flash chromatography (*n*-pentane/EtOAc = 3/1), Moc-**2d** was obtained as a white solid (36.7 mg, 60%). The yield of oxime intermediate was 61% (40.5 mg, 0.18 mmol).

TLC: 0.36 (*n*-pentane/EtOAc = 5/1).

^{1}H NMR (400 MHz, $CDCl_3$) δ 8.06 (d, *J* = 8.4 Hz, 1H), 7.43 (dt, *J* = 1.8, 0.8 Hz, 1H), 7.37 (dd, *J* = 8.4, 1.6 Hz, 1H), 4.10 (s, 3H), 2.57 (s, 3H), 2.48 (s, 3H).

^{13}C NMR (101 MHz, $CDCl_3$) δ 151.3, 149.2, 139.0, 133.6, 131.1, 126.4, 120.0, 114.2, 54.3, 21.4, 12.4.

HRMS (ESI) m/z: [M+Na$^+$] Calculated for $C_{11}H_{12}N_2O_2Na$ 227.07910; Found 227.07888.

IR (neat): 1722 (s), 1534 (w), 1457 (s), 1399 (s), 1380 (s), 1347 (m), 1328 (w), 1281 (m), 1249 (s), 1205 (m), 1083 (s), 1038 (w), 803 (m), 762 (m).

Mp: 106-107 °C.

Methyl 2,5-dimethyl-1*H*-benzo[*d*]imidazole-1-carboxylate Moc-**3d**

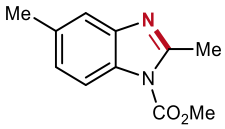

Moc-**3d** was prepared according to the general procedure IV using Moc-**1d** (61.0 mg, 0.300 mmol, 1.0 equiv.), *N*-nitrosomorpholine (105 mg, 0.900 mmol, 3.0 equiv.), TsOH•H_2O (86 mg, 0.45 mmol, 1.5 equiv.) in EtOAc (3 mL) and subsequent ring closure with Et_3N (37.5 µL, 0.27 mmol), 2,6-lutidine (28.0 µL, 0.27 mmol) in dry DCE (2 mL) as well as MsCl (16.7 µL, 0.276 mmol) in dry DCE (1 mL). After purification by flash chromatography (*n*-pentane/EtOAc = 3/1), Moc-**3d** was obtained as a white solid (28.3 mg, 46%). The yield of oxime intermediate was 60% (39.7 mg, 0.18 mmol).

TLC: 0.30 (*n*-pentane/EtOAc = 10/3).

^{1}H NMR (300 MHz, $CDCl_3$) δ 7.74 (d, *J* = 8.4 Hz, 1H), 7.43 (s, 1H), 7.11 (dd, *J* = 8.4, 1.0 Hz, 1H), 4.09 (s, 3H), 2.81 (s, 3H), 2.45 (s, 3H).

^{13}C NMR (76 MHz, $CDCl_3$) δ 153.2, 151.3, 142.6, 134.3, 130.8, 125.7, 119.5, 114.4, 54.3, 21.5, 18.4.

HRMS (ESI) m/z: [M+Na$^+$] Calculated for $C_{11}H_{12}N_2O_2Na$ 227.07910; Found 227.07886.

IR (neat): 1747 (s), 1557 (w), 1439 (m), 1347 (s), 1327 (s), 1269 (w), 1206 (s), 1140 (s), 1097 (s), 1025 (w), 960 (w), 937 (w), 801 (m), 766 (m), 596 (w).

Mp: 86-87 °C.

Ethyl 5-cyano-3-methyl-1*H*-indazole-1-carboxylate 2e

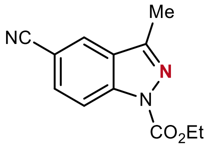

2e was prepared according to the general procedure III using Moc-**1e** (64.0 mg, 0.300 mmol, 1.0 equiv.), *N*-nitrosomorpholine (105 mg, 0.900 mmol, 3.0 equiv.), TsOH•H_2O (86 mg, 0.45 mmol, 1.5 equiv.) in EtOAc (3 mL) and subsequent ring closure with PPh_3 (28 mg, 0.11 mmol), DEAD (0.05 mL, 0.11 mmol, 40 wt.% in toluene) in dry THF (1 mL). After purification by flash chromatography (*n*-pentane/EtOAc = 5/1), **2e** was obtained as a white solid (7 mg, 10%). The yield of oxime intermediate was determined by **^{1}H NMR** with dibromomethane as internal standard (18%, 0.054 mmol). In this case, indole Moc-**1e** was recovered (44.5 mg, 65%).

TLC: 0.24 (*n*-pentane/EtOAc = 10/3).

^{1}H NMR (400 MHz, $CDCl_3$) δ 8.33 (d, *J* = 8.7 Hz, 1H), 8.05 (t, *J* = 1.2 Hz, 1H), 7.77 (dq, *J* = 8.7, 1.3 Hz, 1H), 4.61 (q, *J* = 7.1 Hz, 1H), 2.64 (t, *J* = 1.2 Hz, 3H), 1.53 (t, *J* = 7.1 Hz, 1H).

^{13}C NMR (101 MHz, $CDCl_3$) δ 150.4, 149.1, 142.0, 131.8, 126.1, 126.1, 118.8, 116.0, 107.6, 64.7, 14.6, 12.3.

HRMS (ESI) m/z: [M+Na$^+$] Calculated for $C_{12}H_{11}N_3O_2Na$: 252.07544; found: 252.07430.

IR (neat): 1735 (s), 1558 (m), 1540 (m), 1507 (m), 1456 (m), 1417 (s), 1384 (m), 1353 (w), 1281 (m), 1252 (s), 1190 (m), 1074 (s), 1037 (m), 826 (s), 762 (s).

Mp: 191-192 °C.

Methyl 6-(benzyloxy)-2-methyl-1*H*-benzo[*d*]imidazole-1-carboxylate Moc-**3f**

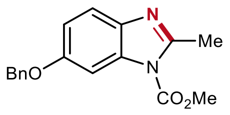

Moc-**3f** was prepared according to the general procedure IV using Moc-**1f** (89.0 mg, 0.300 mmol, 1.0 equiv.), *N*-nitrosomorpholine (105 mg, 0.900 mmol, 3.0 equiv.), TsOH•H_2O (86 mg, 0.45 mmol, 1.5 equiv.) in EtOAc (3 mL) and subsequent ring closure with Et_3N (28 µL, 0.20 mmol), 2,6-lutidine (21 µL, 0.20 mmol) in dry DCE (1 mL) as well as MsCl (12.5 µL, 0.16 mmol) in dry DCE (1 mL). After purification by flash chromatography (*n*-pentane/EtOAc = 4/1), Moc-**3f** was obtained as a yellow solid (35 mg, 40%). The yield of oxime intermediate was determined by ^1H NMR with dibromomethane as internal standard (44%, 0.132 mmol).

TLC: 0.20 (*n*-pentane/EtOAc = 5/2).

^{1}H NMR (400 MHz, $CDCl_3$) δ 7.59 – 7.50 (m, 2H), 7.51 – 7.45 (m, 2H), 7.43 – 7.36 (m, 2H), 7.36 – 7.30 (m, 1H), 7.01 (dd, *J* = 8.7, 2.5 Hz, 1H), 5.12 (s, 2H), 4.08 (s, 3H), 2.79 (s, 3H).

^{13}C NMR (101 MHz, $CDCl_3$) δ 156.7, 152.1, 151.2, 137.1, 136.8, 133.7, 128.7, 128.2, 127.7, 119.8, 113.2, 101.3, 71.0, 54.4, 18.4.

HRMS (ESI) m/z: [M+Na$^+$] Calculated for $C_{17}H_{16}N_2O_3Na$: 319.10531; found: 319.10561.

IR (neat): 1750 (s), 1480 (m), 1443 (s), 1351 (s), 1280 (m), 1202 (s), 1178 (s), 1090 (m), 1023 (m), 766 (m).

Mp: 83-84 °C.

***tert*-Butyl 5-bromo-6-chloro-3-methyl-1*H*-indazole-1-carboxylate** Boc-**2g**

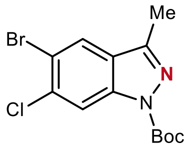

Boc-**2g** was prepared according to the general procedure III using Boc-**1g** (103 mg, 0.300 mmol, 1.0 equiv.), *N*-nitrosomorpholine (105 mg, 0.900 mmol, 3.0 equiv.), TsOH•H_2O (86 mg, 0.45 mmol, 1.5 equiv.) in EtOAc (3 mL) and subsequent ring closure with PPh_3 (35 mg, 0.14 mmol), DEAD (60 µL, 0.14 mmol, 40 wt.% in toluene) in dry THF (1 mL). After purification by flash chromatography (*n*-pentane/EtOAc = 20/1), Boc-**2g** was obtained as a white solid (9 mg, 9%). The yield of oxime intermediate was determined by ^1H NMR with dibromomethane as internal standard (22%, 0.066 mmol). In this case, indole Moc-**1g** was recovered (36.0 mg, 35%)

TLC: 0.62 (*n*-pentane/EtOAc = 10/3).

^{1}H NMR (300 MHz, $CDCl_3$) δ 8.29 (s, 1H), 7.91 (s, 1H), 2.56 (d, *J* = 0.7 Hz, 3H), 1.71 (s, 9H).

^{13}C NMR (101 MHz, $CDCl_3$) δ 148.9, 147.5, 139.6, 135.4, 126.0, 124.9, 117.2, 116.4, 85.6, 28.3, 12.4.

HRMS (ESI) m/z: [M+Na$^+$] Calculated for $C_{13}H_{14}N_2O_2BrClNa$: 366.98194; found: 366.98183.

IR (neat): 1760 (w), 1732 (m), 1412 (w), 1393 (s), 1367 (s), 1331 (s), 1301 (w), 1248 (s), 1151 (s), 1115 (s), 1075 (s), 1027 (m), 848 (w), 792 (s), 761 (m).

Mp: 132-133 °C.

Methyl 3-isobutyl-1*H*-indazole-1-carboxylate Moc-**2h**

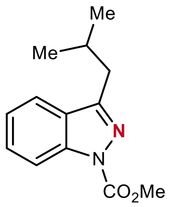

Moc-**2h** was prepared according to the general procedure III using Moc-**1h** (69.4 mg, 0.300 mmol, 1.0 equiv.), *N*-nitrosomorpholine (105 mg, 0.900 mmol, 3.0 equiv.), TsOH•H_2O (86 mg, 0.45 mmol, 1.5 equiv.) in EtOAc (3 mL) and subsequent ring closure with PPh_3 (36 mg, 0.14 mmol), DEAD (60 µL, 0.14 mmol, 40 wt.% in toluene) in dry THF (1 mL). After purification by flash chromatography (*n*-pentane/EtOAc = 7/1), Moc-**2h** was obtained as a colourless oil (11.5 mg, 17%). The yield of oxime intermediate was 23% (16.9 mg, 0.0680 mmol). In this case, indole Moc-**1h** was recovered (33.0 mg, 48%).

TLC: 0.62 (*n*-pentane/EtOAc = 10/3).

^{1}H NMR (400 MHz, $CDCl_3$) δ 8.23 (d, *J* = 8.4 Hz, 1H), 7.69 (dd, *J* = 8.0, 1.2 Hz, 1H), 7.55 (dd, *J* = 8.4, 7.2 Hz, 1H), 7.33 (dd, *J* = 8.0, 6.8 Hz, 1H), 4.12 (s, 3H), 2.87 (d, *J* = 7.2 Hz, 2H), 2.22 (dt, *J* = 13.6, 6.8 Hz, 1H), 0.99 (d, *J* = 6.4 Hz, 6H).

^{13}C NMR (76 MHz, $CDCl_3$) δ 152.7, 151.4, 140.8, 129.2, 126.0, 123.8, 120.8, 114.8, 54.5, 36.2, 28.8, 22.9.

HRMS (ESI) m/z: [M+Na$^+$] Calculated for $C_{13}H_{16}N_2O_2Na$: 255.11040; Found 255.10999.

IR (neat): 1743 (s), 1521 (w), 1444 (s), 1395 (m), 1372 (m), 1340 (w), 1290 (w), 1244 (s), 1075 (s), 762 (s).

Methyl 2-isobutyl-1*H*-benzo[*d*]imidazole-1-carboxylate Moc-**3h**

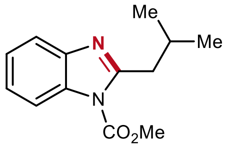

Moc-**3h** was prepared according to the general procedure IV using Moc-**1h** (69.4 mg, 0.300 mmol, 1.0 equiv.), *N*-nitrosomorpholine (105 mg, 0.900 mmol, 3.0 equiv.), TsOH•H_2O (86 mg, 0.45 mmol, 1.5 equiv.) in EtOAc (3 mL) and subsequent ring closure with Et_3N (15 µL, 0.11 mmol), 2,6-lutidine (11 µL, 0.11mmol) in dry DCE (1 mL) as well as MsCl (6.5 µL, 0.084 mmol) in dry DCE (1 mL). After purification by flash chromatography (*n*-pentane/EtOAc = 7/1), Moc-**3h** was obtained as a colourless oil (11.7 mg, 17%). The yield of oxime intermediate was 23% (17.5 mg, 0.070 mmol). In this case, indole Moc-**1h** was recovered (35.6 mg, 51%).

TLC: 0.60 (*n*-pentane/EtOAc = 10/3).

^{1}H NMR (400 MHz, $CDCl_3$) δ 7.96 – 7.81 (m, 1H), 7.79 – 7.66 (m, 1H), 7.32 (t, *J* = 3.6 Hz, 2H), 4.11 (s, 3H), 3.10 (d, *J* = 6.8 Hz, 2H), 2.27 (dt, *J* = 13.6, 7.2 Hz, 1H), 1.04 (d, *J* = 6.8 Hz, 6H).

^{13}C NMR (76 MHz, $CDCl_3$) δ 156.1, 151.2, 142.4, 132.9, 124.5, 124.5, 119.7, 115.0, 54.4, 39.7, 27.7, 22.7.

HRMS (ESI) m/z: [M+Na$^+$] Calculated for $C_{13}H_{16}N_2O_2Na$: 255.11040; Found 255.11040.

IR (neat): 1751 (s), 1541 (w), 1455 (s), 1440 (m), 1347 (s), 1325 (s), 1257 (w), 1190 (s), 1122 (w), 1090 (m), 764 (s), 746 (m).

3-Cyclohexyl-1*H*-indazole 2i

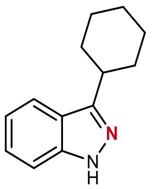

2i was prepared according to the general procedure VII using **1i** (99.6 mg, 0.500 mmol, 1.0 equiv.) in MeOH (10 mL) with $NaIO_4$ (428 mg, 2.00 mmol, 4.0 equiv.) in water (5 mL) for oxidative cleavage. Deformylation was conducted under basic conditions using 10% NaOH aqueous solution (1 mL) in MeOH (5 mL) at 70 °C for 1 h to give the aminophenone intermediate (85.3 mg, 0.420 mmol, 84%). The ring closure step was conducted with 2-aminophenone (85.3 mg, 0.420 mmol) with $Me_3C_6H_2SO_2ONHBoc$ (199 mg, 0.630 mmol) in HFIP (2 mL). After purification by flash chromatography (*n*-pentane/EtOAc = 5/1), **2i** was obtained as a brown solid (64.0 mg, 64%).

^{1}H NMR (300 MHz, $CDCl_3$) δ 10.63 (s, 1H), 7.79 (dt, *J* = 8.2, 1.0 Hz, 1H), 7.49 – 7.30 (m, 2H), 7.18 – 7.07 (m, 1H), 3.11 (tt, *J* = 11.9, 3.5 Hz, 1H), 2.17 – 2.07 (m, 2H), 1.95 – 1.85 (m, 2H), 1.83 – 1.72 (m, 3H), 1.57 – 1.33 (m, 3H).

^{13}C NMR (76 MHz, $CDCl_3$) δ 151.8, 141.4, 126.6, 121.4, 120.8, 120.0, 110.0, 37.7, 32.7, 26.8, 26.4.

Analytic data are in agreement with those reported in the literature.[40]

2-Cyclohexyl-1-tosyl-1*H*-benzo[*d*]imidazole Ts-**3i**

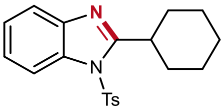

Ts-**3i** was prepared according to the general procedure VIII using **1i** (99.6 mg, 0.500 mmol, 1.0 equiv.) in MeOH (10 mL) with $NaIO_4$ (428 mg, 2.00 mmol, 4.0 equiv.) in water (5 mL) for oxidative cleavage. Deformylation was conducted under basic conditions using 10% NaOH aqueous solution (1 mL) in MeOH (5 mL) at 70 °C for 1 h to give the 2-aminophenone intermediate (85.3 mg, 0.420 mmol, 84%). The ring closure step was conducted with 2-aminophenone (85.3 mg, 0.420 mmol) *via* ketimine. The corresponding ketimine was synthesized at 50 °C for 20 h. After purification by flash chromatography (*n*-pentane/EtOAc = 10/1), Ts-**3i** was obtained as a white solid (40.8 mg, 23%).

TLC: 0.70 (*n*-pentane/EtOAc = 10/3).

^{1}H NMR (300 MHz, $CDCl_3$) δ 8.11 – 7.98 (m, 1H), 7.79 – 7.72 (m, 2H), 7.69 – 7.65 (m, 1H), 7.34 – 7.30 (m, 2H), 7.29 – 7.24 (m, 2H), 3.57 – 3.34 (m, 1H), 2.38 (s, 3H), 1.99 – 1.81 (m, 3H), 1.80 – 1.59 (m, 4H), 1.51 – 1.27 (m, 3H).

^{13}C NMR (76 MHz, $CDCl_3$) δ 159.8, 145.9, 142.2, 136.0, 133.0, 130.3, 126.8, 124.8, 124.7, 119.9, 114.1, 38.3, 32.7, 26.4, 25.9, 21.8.

HRMS (ESI) m/z: [M+Na$^+$] Calculated for $C_{20}H_{22}N_2O_2SNa$: 377.12942; Found 377.12933.

IR (neat): 1449 (w), 1372 (m), 1198 (w), 1166 (m), 1120 (w), 1088 (m), 1047 (m), 811 (w), 766 (w), 742 (m), 703 (w), 665 (m), 643 (w), 575 (s), 542 (m).

Mp: 121-122 °C.

3-(Adamantan-2-yl)-1*H*-indazole 2j

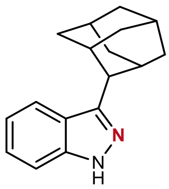

2j was prepared according to the general procedure VII using **1j** (101 mg, 0.400 mmol, 1.0 equiv.) with *m*-CPBA for oxidative cleavage. To a solution of **1j** in dry DCM (8 mL), *m*-CPBA (215 mg, 0.800 mmol, 2.0 equiv., ≤77%) was added slowly at 0 °C. Then the reaction was stirred at 0 °C and monitored by TLC. After completion of the reaction (around 30 min. to 1 h), sat. $NaHCO_3$ aqueous solution was added and the mixture was extracted with DCM (×3). The combined organic phase was washed with brine and dried over anhydrous Na_2SO_4. The solvent was removed with a rotary evaporator under reduced pressure and the residue was directly used for deformylation. Deformylation was conducted under acidic conditions use conc. HCl (50 µL) in MeOH at 70 °C for 1 h to give the aminophenone intermediate (63.8 mg, 0.250 mmol, 63%). The ring closure step was conducted with 2-aminophenone (63.8 mg, 0.250 mmol) with $Me_3C_6H_2SO_2ONHBoc$ (118 mg, 0.375 mmol) in HFIP (1.5 mL). After purification by flash chromatography (*n*-pentane/EtOAc = 6/1), **2j** was obtained as a white solid (56.5 mg, 56%).

TLC: 0.76 (*n*-pentane/EtOAc = 10/3).

^{1}H NMR (300 MHz, $CDCl_3$) δ 8.92 (s, 1H), 7.78 – 7.70 (m, 1H), 7.49 – 7.42 (m, 1H), 7.40 – 7.32 (m, 1H), 7.16 – 7.07 (m, 1H), 3.51 (s, 1H), 2.55 (s, 2H), 2.27 (d, *J* = 12.4 Hz, 2H), 2.04 (d, *J* = 2.6 Hz, 5H), 1.92 – 1.86 (m, 1H), 1.85 – 1.78 (m, 2H), 1.72 – 1.61 (m, 2H).

^{13}C NMR (76 MHz, $CDCl_3$) δ 150.1, 141.1, 126.5, 122.2, 121.0, 120.0, 109.8, 44.0, 39.3, 38.1, 32.8, 31.8, 28.3, 28.1.

HRMS (ESI) m/z: [M+H$^+$] Calculated for $C_{17}H_{20}N_2H$: 253.16993; Found 253.16982.

IR (neat): 3154 (w), 2899 (s), 2847 (m), 1621 (w), 1493 (w), 1448 (w), 1346 (w), 1319 (w), 1230 (w), 1096 (w), 1033 (w), 965 (w), 738 (s).

Mp: 186-187 °C.

3-(2-Phenylpropan-2-yl)-1*H*-indazole 2k

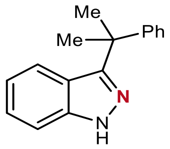

2k was prepared according to the general procedure VII using **1k** (141 mg, 0.600 mmol, 1.0 equiv.) with *m*-CPBA for oxidative cleavage. To a solution of **1k** in dry DCM (12 mL), *m*-CPBA (274 mg, 1.5 mmol, 2.5 equiv., ≤77%) was added slowly by two potions at 0 °C (The second potion of 0.5 mmol *m*-CPBA was added after 30 min.). Then the reaction was stirred at 0 °C and monitored by TLC. After completion of the reaction (around 1 h), sat. $NaHCO_3$ aqueous solution was added and the mixture was extracted with DCM (×3). The combined organic phase was washed with brine and dried over anhydrous Na_2SO_4. The solvent was removed with a rotary evaporator under reduced pressure and the residue was directly used for deformylation. Deformylation was conducted under acidic conditions using conc. HCl (75 µL) in MeOH at 70 °C for 1 h to give the 2-aminophenone intermediate (100 mg, 0.420 mmol, 70%). The ring closure step was conducted with 2-aminophenone (23.9 mg, 0.1 mmol) with $Me_3C_6H_2SO_2ONHBoc$ (48 mg, 0.15 mmol) in HFIP (0.5 mL). After purification by flash chromatography (*n*-pentane/EtOAc = 4/1), **2k** was

obtained as a brown solid (12.8 mg, 38% overall yield).

TLC: 0.44 (*n*-pentane/EtOAc = 10/3).

^{1}H NMR (300 MHz, $CDCl_3$) δ 10.00 (s, 1H), 7.41 (dt, *J* = 8.4, 0.9 Hz, 1H), 7.35 – 7.26 (m, 5H), 7.23 – 7.16 (m, 1H), 7.08 – 6.99 (m, 1H), 6.89 (ddd, *J* = 8.3, 6.8, 1.0 Hz, 1H), 1.88 (s, 6H).

^{13}C NMR (76 MHz, $CDCl_3$) δ 154.3, 148.7, 142.0, 128.3, 126.5, 126.4, 126.2, 122.2, 121.0, 120.1, 109.8, 41.2, 29.7.

HRMS (ESI) m/z: [M+H$^+$] Calculated for $C_{16}H_{16}N_2H$: 237.13863; Found 237.13856.

IR (neat): 2972 (w), 2933 (w), 1493 (w), 1339 (w), 1048 (w), 746 (s), 700 (m), 590 (w), 457 (w).

Mp: 150-151 °C.

2-(2-Phenylpropan-2-yl)-1-tosyl-1*H*-benzo[*d*]imidazole Ts-**3k**

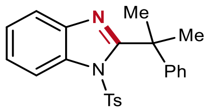

Ts-**3k** was prepared according to the general procedure VII using **1k** (141 mg, 0.600 mmol, 1.0 equiv.) with *m*-CPBA for oxidative cleavage. 2-Aminophenone intermediate obtained from the first step of **2k** was directly used. The ring closure step was conducted with 2-aminophenone intermediate (75 mg, 0.3 mmol) *via* ketimine. After second step's purification by flash chromatography (*n*-pentane/EtOAc = 10/1), Ts-**3k** was obtained as a brown solid (16 mg, 10% overall yield).

TLC: 0.82 (*n*-pentane/EtOAc = 10/3).

^{1}H NMR (400 MHz, $CDCl_3$) δ 7.96 – 7.90 (m, 1H), 7.81 – 7.74 (m, 1H), 7.37 – 7.29 (m, 2H), 7.22 (s, 5H), 6.95 (q, *J* = 8.6 Hz, 4H), 2.29 (s, 3H), 2.00 (s, 6H).

^{13}C NMR (101 MHz, $CDCl_3$) δ 160.9, 146.0, 145.0, 141.0, 135.7, 134.6, 129.6, 128.3, 127.2, 126.8, 126.3, 125.1, 124.6, 120.4, 114.6, 44.5, 31.4, 21.6.

HRMS (ESI) m/z: [M+Na$^+$] Calculated for $C_{23}H_{22}N_2O_2SNa$: 413.12942; Found 413.12952.

IR (neat): 1453 (w), 1377 (m), 1263 (w), 1178 (m), 1085 (w), 748 (s), 702 (m), 678 (s), 654 (w), 577 (s), 547 (m).

Mp: 140-142 °C.

3-Phenyl-1*H*-indazole 2l

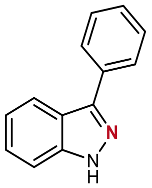

2l was prepared according to the general procedure VII using **1l** (99.5 mg, 0.500 mmol, 1.0 equiv.) in MeOH (4 mL) with $NaIO_4$ (428 mg, 2.00 mmol, 4.0 equiv.) in water (4 mL) at 60 °C for 12 h for oxidative cleavage. Deformylation was conducted under basic conditions using 10% NaOH aqueous solution (1 mL) in MeOH (5 mL) at 70 °C for 1 h to give the 2-aminophenone intermediate (78.8 mg, 0.410 mmol, 82%). The ring closure step was conducted with 2-aminophenone (78.8 mg, 0.410 mmol) with $Me_3C_6H_2SO_2ONHBoc$ (194 mg, 0.62 mmol) in HFIP (2 mL). After

purification by flash chromatography (*n*-pentane/EtOAc = 5/1), **2l** was obtained as a brown solid (66.0 mg, 68%).

^{1}H NMR (300 MHz, $CDCl_3$) δ 10.75 (s, 1H), 8.07 – 7.98 (m, 3H), 7.59 – 7.52 (m, 2H), 7.50 – 7.43 (m, 1H), 7.39 – 7.32 (m, 1H), 7.26 – 7.19 (m, 2H).

^{13}C NMR (76 MHz, $CDCl_3$) δ 145.7, 141.8, 133.6, 129.1, 128.3, 127.9, 126.9, 121.5, 121.2, 121.0, 110.5.

Analytic data are in agreement with those reported in the literature.[33]

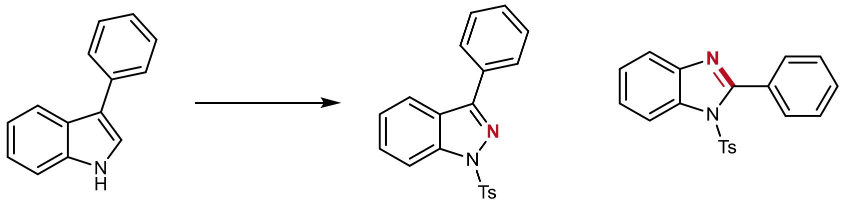

1l Ts-**2l** 33% Ts-**3l** 30%

Ts-**3l** was prepared according to the general procedure VIII using **1l** (99.5 mg, 0.500 mmol, 1.0 equiv.) in MeOH (4 mL) with $NaIO_4$ (428 mg, 2.00 mmol, 4.0 equiv.) in water (4 mL) at 60 °C for 12 h for oxidative cleavage. Deformylation was conducted under basic conditions using 10% NaOH aqueous solution (1 mL) in MeOH (5 mL) at 70 °C for 1 h to give the 2-aminophenone (78.0 mg, 0.410 mmol, 82%). The ring closure step was conducted with 2-aminophenone (78.8 mg, 0.410 mmol) *via* ketimine. The corresponding ketimine was synthesized at 50 °C for 20 h. After purification by flash chromatography (*n*-pentane/EtOAc = 5/1), Ts-**3l** was obtained as a colourless solid (52.2 mg, 30%), Ts-**2l** was obtained as a colourless solid (52.2 mg, 33%).

3-Phenyl-1-tosyl-1*H*-indazole Ts-2l

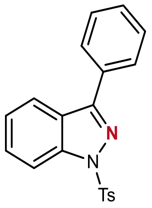

^{1}H NMR (400 MHz, $CDCl_3$) δ 8.28 (d, *J* = 8.5 Hz, 1H), 7.97 – 7.89 (m, 5H), 7.63 – 7.54 (m, 1H), 7.54 – 7.45 (m, 3H), 7.37 (dd, *J* = 8.2, 6.9 Hz, 1H), 7.23 (d, *J* = 8.1 Hz, 2H), 2.34 (s, 3H).

^{13}C NMR (101 MHz, $CDCl_3$) δ 151.8, 145.4, 142.0, 134.8, 131.5, 129.9, 129.7, 129.2, 128.9, 128.4, 127.7, 124.6, 124.4, 121.8, 113.7, 21.7.

Analytic data are in agreement with those reported in the literature.[41]

2-Phenyl-1-tosyl-1*H*-benzo[*d*]imidazole Ts-3l

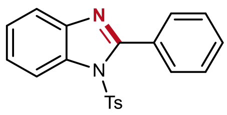

^{1}H NMR (400 MHz, $CDCl_3$) δ 8.25 – 8.16 (m, 1H), 7.75 – 7.70 (m, 1H), 7.63 – 7.58 (m, 2H), 7.58 – 7.51 (m, 1H),

7.50 – 7.36 (m, 4H), 7.35 – 7.29 (m, 2H), 7.09 (d, J = 7.9 Hz, 2H), 2.32 (s, 3H).

^{13}C NMR (101 MHz, $CDCl_3$) δ 154.2, 145.8, 142.7, 135.1, 134.0, 131.0, 130.6, 130.2, 129.8, 127.8, 127.1, 125.6, 125.4, 120.5, 115.3, 21.7.

Analytic data are in agreement with those reported in the literature.[41]

1*H*-Indazole 2m

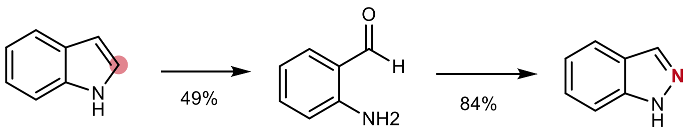

1m **2m**

Step 1: the *Witkop-Winterfeldt* oxidation of indole **1m** was conducted according to a literature procedure[42]: Ozone (around 0.35 mmol/min) in O_2 (1 L/min) was bubbled about 10 min through a solution of indole (175 mg, 1.50 mmol) in 10 mL DCM and 5 mL methanol in a Schlenk tube at room temperature until the starting material disappeared as checked by TLC. The residual ozone was flushed with O_2. Then, PPh_3 (525 mg, 2.00 mmol) was added to the solution and the mixture was stirred at room temperature overnight. The solvent was removed by rotary vacuum evaporation and the residue was dissolved in methanol again. Afterwards, 2 mL 10% NaOH aqueous solution was added and the mixture was heated at 70 °C for 1 h. After completion, methanol was removed and the residue was extracted with DCM for three times. The combined organic phase was washed with brine and dried over anhydrous Na_2SO_4. After filtration, the solvent was removed and the residue was purified by column chromatography (SiO_2, *n*-pentane/EtOAc) to give 2-aminobenzaldehyde as a yellow oil (89.0 mg, 49%).

Step 2: 1*H*-Indazole was synthesized according to a literature procedure[33]: to a mixture of 2-aminobenzaldehyde (24.9 mg, 0.200 mmol, 1.0 equiv.) and hydroxylamine-*O*-sulfonic acid (25 mg, 0.22 mmol, 1.1 equiv.) was added trifluoroethanol. The resulting reaction mixture was stirred at 60 °C for 15 h. After cooling to room temperature, the solvent was removed and the crude products were dissolved in EtOAc and washed with saturated aqueous $NaHCO_3$ solution. The organic phase was collected, concentrated and the residue was purified by column chromatography (SiO_2, *n*-pentane/EtOAc = 10/1) to give 1*H*-indazole **2m** as a light white solid (19.8 mg, 41% over two steps).

^{1}H NMR (300 MHz, $CDCl_3$) δ 8.24 (s, 1H), 7.21 – 7.07 (m, 2H), 6.81 – 6.65 (m, 2H), 5.41 (s, 1H).

^{13}C NMR (76 MHz, $CDCl_3$) δ 154.0, 146.4, 132.4, 130.6, 117.1, 115.8, 114.6.

Analytic data are in agreement with those reported in the literature.[33]

- *The skeletal editing of C2,C3-disubstituted indoles **1n** and **1o***

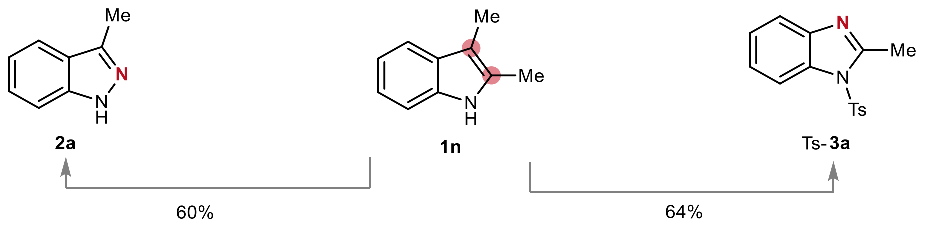

2a and Ts-**3a** were prepared from 2,3-dimethyl-1*H*-indole **1n** according to the general procedure VII and VIII using **1n** (146 mg, 1.00 mmol, 1.0 equiv.) in MeOH (4 mL) and $NaIO_4$ (534 mg, 2.50 mmol, 2.5 equiv.) in H_2O (4 mL) at

room temperature for 24 h. Deformylation was conducted under acidic conditions unsing conc. HCl (0.13 mL) in MeOH (10 mL) to give the 1-(2-aminophenyl)ethan-1-one as a yellow oil (116 mg, 0.860 mmol, 86%). Then, the intermediate was divided equally for the diversification synthesis (58 mg, 0.43 mmol). Through purification of step 2 by flash chromatography (*n*-pentane/EtOAc = 10/1), **2a** was obtained as a light white solid (39.6 mg, 60%, general procedure VII) and Ts-**3a** was obtained as a colourless solid (91.5 mg, 64%, general procedure VIII *via* ketimine intermediate).

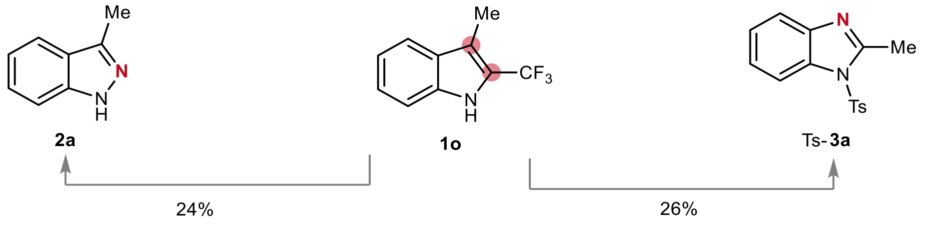

2a and Ts-**3a** were prepared from 3-methyl-2-(trifluoromethyl)-1*H*-indole **1o** according to the general procedure VII and VIII using **1o** with oxone as oxidants. The oxidative cleavage was according to a literature procedure.[43] To a solution of indole **1o** (200 mg, 1.00 mmol, 1.0 equiv.) and KCl (7.4 mg, 0.1 mmol, 0.1 equiv.) in HFIP/H_2O (10 mL, 10/1, 0.1 M) at r.t. was added oxone (368 mg, 1.20 mmol, MW = 307) in one batch. The resulting solution was stirred at r.t. for 24 h. and then diluted with EtOAc. The reaction mixture passed through a short pad of silica gel and washed with EtOAc. The resulting solution was concentrated under reduced pressure and the residue was directly deformylation under acidic conditions using conc. HCl (0.05 mL) in MeOH (10 mL) to give the 1-(2-aminophenyl)ethan-1-one as a yellow oil (40.5 mg, 30%). Through purification of step 2 by flash chromatography (*n*-pentane/EtOAc = 10/1), **2a** was obtained as a light white solid (32 mg, 24% overall yield, general procedure VII) and Ts-**3a** was obtained as a colourless solid (74.4 mg, 26%, general procedure VIII *via* ketimine intermediate).

7,8,9,10-Tetrahydrobenzo[4,5]imidazo[1,2-*a*]azocin-11(6*H*)-one 3p’

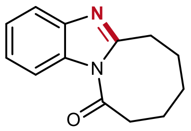

Under air, a solution of indole **1p** (74.2 mg, 0.400 mmol, 1.0 equiv.) in MeOH (2 mL) was added dropwise to a solution of sodium metaperiodate $NaIO_4$ (214 mg, 1.00 mmol, 2.5 equiv.) in water (1 mL) at 0 °C using an ice-water bath. Then, the reaction mixture was allowed to warm to r.t. and was stirred overnight. After consumption of the starting material (as monitored by TLC), the mixture was poured into the water and extracted with DCM (3 times). The combined organic phase was washed with brine and dried over anhydrous Na_2SO_4. After filtration, the solvent was removed with a rotary evaporator under reduced pressure and the residue was dissolved in dry CH_3CN (2 mL) under argon. Then the reaction mixture was cooled to 0 °C using an ice/water bath and $TMSN_3$ (66 µL, 0.50 mmol, 1.25 equiv.) as well as TfOH (53 µL, 0.60 mmol, 1.5 equiv.) were added subsequently. Afterwards, the reaction mixture was stirred at room temperature overnight. After completion, the solvent was removed with a rotary evaporator under reduced pressure and the residue was purified by column chromatography (SiO_2, CH_2Cl_2/EtOAc = 5/1) to give **3p’** as a colorless oil (20.4 mg, 24%).

TLC: 0.35 (*n*-pentane/EtOAc = 1/2).

^{1}H NMR (400 MHz, $CDCl_3$) δ 8.39 – 8.32 (m, 1H), 7.71 – 7.65 (m, 1H), 7.40 – 7.32 (m, 2H), 3.49 (t, J = 7.2 Hz, 2H), 3.20 (t, J = 7.3 Hz, 2H), 2.09 – 1.94 (m, 4H), 1.57 – 1.51 (m, 2H).

^{13}C NMR (101 MHz, $CDCl_3$) δ 172.4, 153.9, 142.2, 134.4, 125.2, 125.1, 119.4, 116.8, 37.9, 30.8, 26.0, 24.5, 23.5.

HRMS (ESI) m/z: [M+H$^+$] Calculated for $C_{13}H_{14}N_2OH$: 215.11789; Found 215.11783.

IR (neat): 1711 (m), 1542 (w), 1454 (m), 1347 (m), 1323 (s), 1266 (m), 1130 (w), 1048 (w), 752 (s), 589 (w).

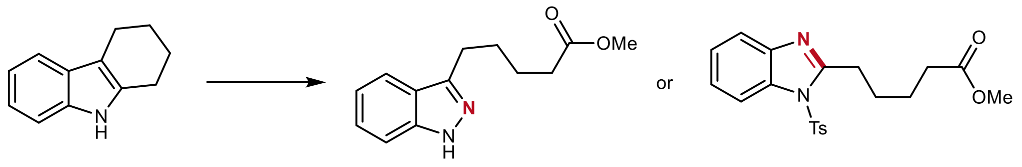

1q **2q** 67% (B) Ts-**3q** 38% (B, Ts-**2q** 15%)

Under air, a solution of indole **1q** (86 mg, 0.50 mmol, 1.0 equiv.) in MeOH (2.5 mL) was added dropwise to a solution of sodium metaperiodate $NaIO_4$ (268 mg, 1.25 mmol, 2.5 equiv.) in water (2 mL) at 0 °C using an ice-water bath. Then, the reaction mixture was allowed to warm to r.t. and stirred for 4 h. After consumption of the starting material (monitored by TLC), the mixture was poured into the water and extracted with DCM (3 times). The combined organic phase was washed with brine and dried over anhydrous Na_2SO_4. After filtration, the solvent was removed with a rotary evaporator under reduced pressure and the residue was dissolved in MeOH (10 mL). Then, conc. HCl (0.06 mL) was added slowly and the reaction mixture was heated at 70 °C for 2 h to break the ring. After work up according to the general procedure VII, methyl 6-(2-aminophenyl)-6-oxohexanoate was obtained (121.5 mg, quantitative yield).

Methyl 5-(1*H*-indazol-3-yl)pentanoate 2q

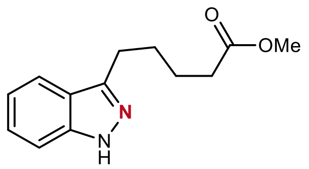

2q was prepared according to the general procedure VII using the ring-opened intermediate methyl 6-(2-aminophenyl)-6-oxohexanoate (47.1 mg, 0.200 mmol, 1.0 equiv.) with $Me_3C_6H_2SO_2ONHBoc$ (95 mg, 0.30 mmol) in HFIP (1 mL). After purification by flash chromatography (n-pentane/EtOAc = 4/1), **2q** was obtained as a colourless oil (31 mg, 67%).

TLC: 0.24 (n-pentane/EtOAc = 5/2).

^{1}H NMR (400 MHz, $CDCl_3$) δ 10.46 (s, 1H), 7.72 – 7.66 (m, 1H), 7.45 – 7.40 (m, 1H), 7.39 – 7.32 (m, 1H), 7.16 – 7.09 (m, 1H), 3.65 (s, 3H), 3.03 (t, J = 7.5 Hz, 2H), 2.37 (t, J = 7.4 Hz, 2H), 1.95 – 1.83 (m, 2H), 1.81 – 1.70 (m, 2H).

^{13}C NMR (101 MHz, $CDCl_3$) δ 174.2, 147.0, 141.3, 126.8, 122.2, 120.29, 120.27, 109.9, 51.6, 34.0, 28.6, 26.8, 24.9.

HRMS (ESI) m/z: [M+Na$^+$] Calculated for $C_{13}H_{16}N_2O_2Na$: 255.11040; Found 255.11026.

IR (neat): 1732 (s), 1621 (w), 1499 (w), 1436 (m), 1345 (m), 1249 (m), 1198 (m), 1174 (m), 1005 (w), 744 (s).

Methyl 5-(1-tosyl-1*H*-benzo[d]imidazol-2-yl)pentanoate Ts-3q

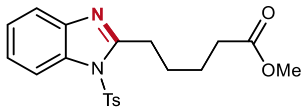

Ts-**3q** was prepared according to the general procedure VIII using the ring-opened intermediate methyl 6-(2-aminophenyl)-6-oxohexanoate (70.6 mg, 0.300 mmol, 1.0 equiv.) *via* oxime as intermediate. After purification by flash chromatography (*n*-pentane/EtOAc = 10/1 to 4/1), Ts-**3q** was obtained as a brown solid (43.2 mg, 38%), Ts-**2q** was obtained as a yellow solid (16.7 mg, 15%).

TLC: 0.42 (*n*-pentane/EtOAc = 5/2).

^{1}H NMR (400 MHz, $CDCl_3$) δ 8.06 – 7.96 (m, 1H), 7.81 – 7.73 (m, 2H), 7.67 – 7.59 (m, 1H), 7.36 – 7.30 (m, 2H), 7.29 – 7.24 (m, 2H), 3.67 (s, 3H), 3.23 – 3.09 (m, 2H), 2.41 – 2.38 (m, 2H), 2.37 (s, 3H), 1.99 – 1.89 (m, 2H), 1.85 – 1.73 (m, 2H).

^{13}C NMR (101 MHz, $CDCl_3$) δ 173.9, 154.7, 146.0, 142.0, 135.7, 133.3, 130.4, 126.8, 124.9, 124.7, 119.9, 113.7, 51.7, 33.9, 29.7, 27.2, 24.7, 21.8.

HRMS (ESI) m/z: [M+Na^{+}] Calculated for $C_{20}H_{22}N_2O_4SNa$: 409.11925; Found 409.11894.

IR (neat): 1732 (m), 1451 (w), 1371 (m), 1166 (s), 1089 (w), 1041 (w), 765 (w), 746 (m), 664 (s), 576 (s), 543 (s).

Mp: 69-71 °C.

Methyl 5-(1-tosyl-1*H*-indazol-3-yl)pentanoate Ts-2q

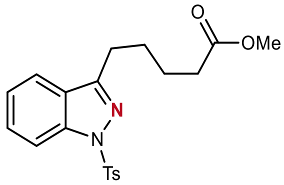

TLC: 0.30 (*n*-pentane/EtOAc = 10/3).

^{1}H NMR (400 MHz, $CDCl_3$) δ 8.19 – 8.14 (m, 1H), 7.85 – 7.77 (m, 2H), 7.64 – 7.58 (m, 1H), 7.56 – 7.49 (m, 1H), 7.33 – 7.27 (m, 1H), 7.23 – 7.17 (m, 2H), 3.65 (s, 3H), 2.92 (t, *J* = 7.5 Hz, 2H), 2.34 (s, 3H), 2.31 (t, *J* = 7.4 Hz, 2H), 1.80 (tt, *J* = 8.3, 7.2 Hz, 2H), 1.69 – 1.59 (m, 2H).

^{13}C NMR (101 MHz, $CDCl_3$) δ 173.9, 154.2, 145.1, 141.5, 134.8, 129.8, 129.2, 127.6, 125.8, 124.0, 120.6, 113.7, 51.6, 33.8, 27.6, 26.8, 24.6, 21.7.

HRMS (ESI) m/z: [M+Na^{+}] Calculated for $C_{20}H_{22}N_2O_4SNa$: 409.11925; Found 409.11913

IR (neat): 1733 (m), 1598 (w), 1518 (w), 1435 (w), 1374 (m), 1254 (w), 1188 (m), 1174 (s), 1123 (w), 813 (w), 750 (m), 704 (w), 672 (m), 574 (m), 540 (m).

Mp: 57-59 °C.

6,7,8,9-Tetrahydro-10*H*-benzo[4,5]imidazo[1,2-*a*]azepin-10-one 3q'

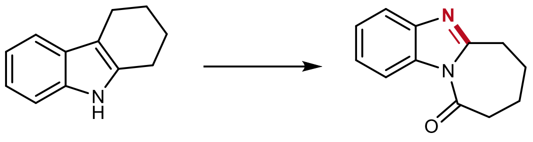

1q **3q'** 54% (B)

Under air, a solution of indole **1q** (69 mg, 0.40 mmol, 1.0 equiv.) in MeOH (2 mL) was added dropwise to a solution of sodium metaperiodate $NaIO_4$ (214 mg, 1.00 mmol, 2.5 equiv.) in water (1 mL) at 0 °C using an ice-water bath. Then the reaction mixture was allowe to warm to r.t. and was stirred for 4 h. After consumption of the starting material (as monitored by TLC), the mixture was poured into the water and extracted with DCM (3 times). The combined organic phase was washed with brine and dried over anhydrous Na_2SO_4. After filtration, the solvent was removed with a rotary evaporator under reduced pressure and the residue was dissolved in dry CH_3CN (2 mL) under argon. Then, the reaction mixture was cooled to 0 °C using an ice/water bath and $TMSN_3$ (66 µL, 0.50 mmol, 1.25 equiv.) as well as TfOH (53 µL, 0.60 mmol, 1.5 equiv.) were added subsequently. Afterwards, the reaction mixture was stirred at room temperature for 4 h. After completion, the solvent was removed with a rotary evaporator under reduced pressure and the residue was purified by column chromatography (SiO_2, CH_2Cl_2/EtOAc = 5/1) to give **3q'** as a yellow oil (43.3 mg, 54%).

TLC: 0.33 (*n*-pentane/EtOAc = 1/2).

^{1}H NMR (300 MHz, $CDCl_3$) δ 8.36 – 8.06 (m, 1H), 7.94 – 7.54 (m, 1H), 7.42 – 7.15 (m, 2H), 3.40 – 3.13 (m, 2H), 3.09 – 2.90 (m, 2H), 2.07 – 1.77 (m, 4H).

^{13}C NMR (76 MHz, $CDCl_3$) δ 172.6, 154.8, 142.5, 133.7, 125.02, 125.00, 119.3, 115.7, 35.9, 27.7, 22.0, 20.5.

HRMS (ESI) m/z: [M+H$^+$] Calculated for $C_{12}H_{12}N_2OH$: 201.10224; Found 201.10230.

IR (neat): 1709 (s), 1608 (w), 1545 (m), 1452 (s), 1348 (s), 1325 (s), 1284 (w), 1260 (w), 1167 (m), 1148 (s), 1108 (w), 939 (m), 768 (m), 747 (s), 570 (m).

3-Methylbenzo[*d*]isoxazole 5a

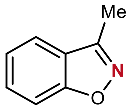

5a was prepared according to the general procedure V using **4a** (37.9 µL, 0.300 mmol, 1.0 equiv.), *N*-nitrosomorpholine (105 mg, 0.900 mmol, 3.0 equiv.), $TsOH{\bullet}H_2O$ (86 mg, 0.45 mmol, 1.5 equiv.) in EtOAc (3 mL) and subsequent ring closure with Et_3N (83.4 µL, 0.60 mmol, 2.0 equiv.) in dry DCE (2 mL) as well as MsCl (25.5 µL, 0.33 mmol, 1.1 equiv.) in dry DCE (1 mL). After purification by flash chromatography (*n*-pentane/Et_2O = 50/1), **5a** was obtained as a colourless oil (29 mg, 73%).

^{1}H NMR (300 MHz, $CDCl_3$) δ 7.63 (dt, *J* = 7.9, 1.0 Hz, 1H), 7.56 – 7.51 (m, 2H), 7.34 – 7.27 (m, 1H), 2.59 (s, 3H).

^{13}C NMR (76 MHz, $CDCl_3$) δ 162.9, 155.1, 129.8, 123.3, 122.3, 121.3, 109.9, 10.2.

Analytic data are in agreement with those reported in the literature.[44]

2-Methylbenzo[*d*]oxazole 6a

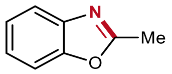

6a was prepared according to the general procedure VI using **4a** (37.9 µL, 0.300 mmol, 1.0 equiv.), *N*-nitrosomorpholine (105 mg, 0.900 mmol, 3.0 equiv.), TsOH•H_2O (86 mg, 0.45 mmol, 1.5 equiv.) in EtOAc (3 mL) and subsequent ring closure with Ms_2O (78.4 mg, 0.45 mmol, 1.5 equiv.). After purification by flash chromatography (*n*-pentane/Et_2O = 50/1), **6a** was obtained as a colourless oil (25.1 mg, 63%).

^{1}H NMR (300 MHz, $CDCl_3$) δ 7.69 – 7.59 (m, 1H), 7.50 – 7.42 (m, 1H), 7.33 – 7.26 (m, 2H), 2.64 (s, 3H).

^{13}C NMR (101 MHz, $CDCl_3$) δ 164.0, 151.1, 141.6, 124.6, 124.2, 119.5, 110.3, 14.6.

Analytic data are in agreement with those reported in the literature.[45]

3-Methylbenzo[*d*]isoxazol-5-yl acetate 5b

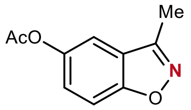

5b was prepared according to the general procedure V using **4b** (57.1 mg, 0.300 mmol, 1.0 equiv.), *N*-nitrosomorpholine (105 mg, 0.900 mmol, 3.0 equiv.), TsOH•H_2O (86 mg, 0.45 mmol, 1.5 equiv.) in EtOAc (3 mL) and subsequent ring closure with Et_3N (83.4 µL, 0.60 mmol, 2.0 equiv.) in dry DCE (2 mL) as well as MsCl (25.5 µL, 0.33 mmol, 1.1 equiv.) in dry DCE (1 mL). After purification by flash chromatography (*n*-pentane/EtOAc = 10/1), **5b** was obtained as a white solid (42.1 mg, 74%).

TLC: 0.20 (*n*-pentane/EtOAc = 10/3).

^{1}H NMR (400 MHz, $CDCl_3$) δ 7.52 (dd, *J* = 8.9, 0.6 Hz, 1H), 7.36 (dd, *J* = 2.3, 0.6 Hz, 1H), 7.25 (dd, *J* = 8.9, 2.3 Hz, 1H), 2.55 (s, 3H), 2.33 (s, 3H).

^{13}C NMR (101 MHz, $CDCl_3$) δ 169.8, 160.7, 155.3, 146.5, 124.3, 122.9, 113.5, 110.5, 21.1, 10.2.

HRMS (ESI) m/z: [M+Na^+] Calculated for $C_{10}H_9N_1O_3Na$ 214.04746; Found 214.04736.

IR (neat): 1756 (m), 1449 (w), 1368 (w), 1202 (s), 1174 (s), 1011 (w), 904 (w), 879 (w), 825 (w), 572 (w), 481 (w).

Mp: 62-63 °C.

2-Methylbenzo[*d*]oxazol-5-yl acetate 6b

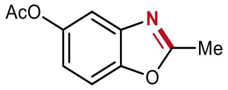

6b was prepared according to the general procedure VI using **4b** (57.1 mg, 0.300 mmol, 1.0 equiv.), *N*-nitrosomorpholine (105 mg, 0.900 mmol, 3.0 equiv.), TsOH•H_2O (86 mg, 0.45 mmol, 1.5 equiv.) in EtOAc (3 mL) and subsequent ring closure with Ms_2O (78.4 mg, 0.45 mmol, 1.5 equiv.). After purification by flash chromatography (*n*-pentane/EtOAc = 10/1), **6b** was obtained as a white solid (46.0 mg, 80%).

TLC: 0.18 (*n*-pentane/EtOAc = 10/3).

^{1}H NMR (300 MHz, $CDCl_3$) δ 7.47 – 7.40 (m, 1H), 7.36 (d, *J* = 2.3 Hz, 1H), 7.01 (dd, *J* = 8.8, 2.3 Hz, 1H), 2.62 (s,

3H), 2.31 (s, 3H).

^{13}C NMR (76 MHz, $CDCl_3$) δ 169.9, 165.4, 148.8, 147.4, 142.3, 118.4, 112.8, 110.4, 21.2, 14.7.

HRMS (ESI) m/z: [M+Na$^+$] Calculated for $C_{10}H_9N_1O_3Na$: 214.04746; Found 214.04735.

IR (neat): 1756 (m), 1576 (w), 1472 (w), 1368 (w), 1266 (w), 1201 (s), 1167 (s), 1128 (s), 1011 (w), 953 (w), 903 (m), 845 (w), 815 (w).

Mp: 67-68 °C.

5-(But-2-yn-1-yloxy)-3-methylbenzo[*d*]isoxazole 5c

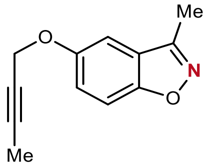

5c was prepared according to the general procedure V using **4c** (60.1 mg, 0.300 mmol, 1.0 equiv.), *N*-nitrosomorpholine (105 mg, 0.900 mmol, 3.0 equiv.), TsOH•H_2O (86 mg, 0.45 mmol, 1.5 equiv.) in EtOAc (3 mL) and subsequent ring closure with Et_3N (83.4 µL, 0.60 mmol, 2.0 equiv.) in dry DCE (2 mL) as well as MsCl (25.5 µL, 0.33 mmol, 1.1 equiv.) in dry DCE (1 mL). After purification by flash chromatography (*n*-pentane/EtOAc = 20/1), **5c** was obtained as a white solid (37.5 mg, 63%).

TLC: 0.56 (*n*-pentane/EtOAc = 20/3).

^{1}H NMR (400 MHz, $CDCl_3$) δ 7.43 (dd, *J* = 9.1, 0.6 Hz, 1H), 7.20 (dd, *J* = 9.0, 2.5 Hz, 1H), 7.06 (dd, *J* = 2.5, 0.6 Hz, 1H), 4.69 (q, *J* = 2.3 Hz, 2H), 2.55 (s, 3H), 1.86 (t, *J* = 2.3 Hz, 3H).

^{13}C NMR (101 MHz, $CDCl_3$) δ 158.6, 155.0, 154.3, 122.5, 120.6, 110.5, 103.4, 84.2, 73.7, 57.3, 10.2, 3.7.

HRMS (ESI) m/z: [M+Na$^+$] Calculated for $C_{12}H_{11}NO_2Na$ 224.06820; Found 224.06803.

IR (neat): 1526(w), 11475 (m), 1450 (s), 1319 (m), 1199 (s), 1072 (w), 1003 (s), 882 (w), 814 (m), 758 (w), 679 (w), 578 (w).

Mp: 60-61 °C.

5-(But-2-yn-1-yloxy)-2-methylbenzo[*d*]oxazole 6c

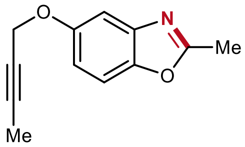

6c was prepared according to the general procedure VI using **4c** (60.1 mg, 0.300 mmol, 1.0 equiv.), *N*-nitrosomorpholine (105 mg, 0.900 mmol, 3.0 equiv.), TsOH•H_2O (86 mg, 0.45 mmol, 1.5 equiv.) in EtOAc (3 mL) and subsequent ring closure with Ms_2O (78.4 mg, 0.45 mmol, 1.5 equiv.). After purification by flash chromatography (*n*-pentane/EtOAc = 20/1), **6c** was obtained as a white solid (37.0 mg, 62%).

TLC: 0.30 (*n*-pentane/EtOAc = 20/3).

^{1}H NMR (400 MHz, $CDCl_3$) δ 7.33 (d, *J* = 8.8 Hz, 1H), 7.24 (d, *J* = 2.6 Hz, 1H), 6.92 (dd, *J* = 8.8, 2.5 Hz, 1H), 4.66

(q, J = 2.3 Hz, 2H), 2.60 (s, 3H), 1.84 (t, J = 2.3 Hz, 3H).

^{13}C NMR (101 MHz, $CDCl_3$) δ 164.8, 155.3, 146.1, 142.3, 113.6, 110.4, 104.5, 84.0, 74.0, 57.4, 14.7, 3.8.

HRMS (ESI) m/z: [M+Na$^+$] Calculated for $C_{12}H_{11}NO_2Na$ 224.06820; Found 224.06811.

IR (neat): 1572 (w), 1559 (w), 1473 (s), 1394 (w), 1272 (s), 1180 (s), 1158 (s), 1146 (m), 1010 (s), 930 (m), 866 (w), 850 (m), 812 (s).

Mp: 82-83 °C.

3-Methyl-5-((3-methylbut-2-en-1-yl)oxy)benzo[*d*]isoxazole 5d

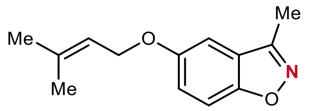

5d was prepared according to the general procedure V using **4d** (64.9 mg, 0.300 mmol, 1.0 equiv.), *N*-nitrosomorpholine (105 mg, 0.900 mmol, 3.0 equiv.), TsOH•H_2O (86 mg, 0.45 mmol, 1.5 equiv.) in EtOAc (3 mL) and subsequent ring closure with Et_3N (83.4 µL, 0.60 mmol, 2.0 equiv.) in dry DCE (2 mL) as well as MsCl (25.5 µL, 0.33 mmol, 1.1 equiv.) in dry DCE (1 mL). After purification by flash chromatography (*n*-pentane/EtOAc = 20/1), **5d** was obtained as a yellow oil (15.6 mg, 24%).

TLC: 0.70 (*n*-pentane/EtOAc = 10/3).

^{1}H NMR (400 MHz, $CDCl_3$) δ 7.33 (dd, J = 9.0, 0.7 Hz, 1H), 7.07 (dd, J = 9.0, 2.4 Hz, 1H), 6.88 (d, J = 2.5 Hz, 1H), 5.42 (ddt, J = 6.8, 5.4, 1.4 Hz, 1H), 4.45 (d, J = 6.8 Hz, 2H), 2.45 (s, 3H), 1.72 (d, J = 1.5 Hz, 3H), 1.67 (d, J = 1.4 Hz, 3H).

^{13}C NMR (101 MHz, $CDCl_3$) δ 158.5, 155.6, 155.1, 138.8, 122.6, 120.8, 119.5, 110.6, 102.7, 65.8, 26.0, 18.4, 10.3.

HRMS (ESI) m/z: [M+Na$^+$] Calculated for $C_{13}H_{15}NO_2Na$ 240.09950; Found 240.09928.

IR (neat): 1524 (w), 1507 (m), 1473 (w), 1450 (s), 1387 (w), 1317 (w), 1267 (w), 1244 (w), 1212 (s), 1071 (w), 999 (m), 813 (w).

3-Methylbenzo[*d*]isoxazol-5-yl trifluoromethanesulfonate 5e

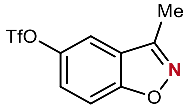

5e was prepared according to the general procedure V using **4e** (84.0 mg, 0.300 mmol, 1.0 equiv.), *N*-nitrosomorpholine (105 mg, 0.900 mmol, 3.0 equiv.), TsOH•H_2O (86 mg, 0.45 mmol, 1.5 equiv.) in EtOAc (3 mL) and subsequent ring closure with Et_3N (83.4 µL, 0.60 mmol, 2.0 equiv.) in dry DCE (2 mL) as well as MsCl (25.5 µL, 0.33 mmol, 1.1 equiv.) in dry DCE (1 mL). After purification by flash chromatography (*n*-pentane/EtOAc = 20/1), **5e** was obtained as a yellow oil (53.0 mg, 66%).

TLC: 0.74 (*n*-pentane/EtOAc = 10/3).

^{1}H NMR (400 MHz, $CDCl_3$) δ 7.62 (dd, J = 9.0, 0.6 Hz, 1H), 7.56 (dd, J = 2.5, 0.6 Hz, 1H), 7.45 (dd, J = 9.0, 2.4 Hz, 1H), 2.61 (s, 3H).

^{13}C NMR (101 MHz, $CDCl_3$) δ 161.64, 155.49, 145.28, 123.49, 123.39, 118.88 (q, J = 320.8 Hz), 114.25, 111.56,

10.21.

^{19}F NMR (282 MHz, $CDCl_3$) δ -72.6.

HRMS (EI) m/z: [M $^+$] Calculated for $C_9H_6NO_4SF_3$ 280.99641; Found 280.99627.

IR (neat): 1419 (m), 1249 (w), 1203 (s), 1137 (s), 1121 (m), 931 (m), 889 (m), 855 (s), 816 (m), 750 (w), 609 (m), 570 (w), 505 (m).

2-Methylbenzo[*d*]oxazol-5-yl trifluoromethanesulfonate 6e

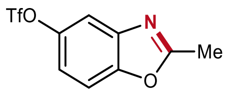

6e was prepared according to the general procedure VI using **4e** (84.0 mg, 0.300 mmol, 1.0 equiv.), *N*-nitrosomorpholine (105 mg, 0.900 mmol, 3.0 equiv.), TsOH•H_2O (86 mg, 0.45 mmol, 1.5 equiv.) in EtOAc (3 mL) and subsequent ring closure with Ms_2O (78.4 mg, 0.45 mmol, 1.5 equiv.). After purification by flash chromatography (*n*-pentane/EtOAc = 20/1), **6e** was obtained as a white solid (40.0 mg, 50%).

TLC: 0.50 (*n*-pentane/EtOAc = 10/3).

^{1}H NMR (300 MHz, $CDCl_3$) δ 7.58 (d, *J* = 2.5 Hz, 1H), 7.52 (dd, *J* = 8.9, 0.5 Hz, 1H), 7.23 (dd, *J* = 8.9, 2.5 Hz, 1H), 2.67 (s, 3H).

^{13}C NMR (76 MHz, $CDCl_3$) δ 166.7, 150.2, 146.1, 142.7, 118.9 (q, *J* = 320.9 Hz), 118.1, 113.0, 111.2, 14.8.

^{19}F NMR (282 MHz, $CDCl_3$) δ -72.6.

HRMS (ESI) m/z: [M+H$^+$] Calculated for $C_9H_6NO_4SF_3H$ 282.00424; Found 282.00419.

IR (neat): 1419 (s), 1235 (w), 1203 (s), 1171 (w), 1138 (s), 1094 (s), 945 (s), 921 (w), 876 (s), 838 (w), 811 (w), 607 (m), 496 (w).

Mp: 55-56 °C.

2-Methylbenzo[d]oxazol-5-yl trifluoromethanesulfonate 5f

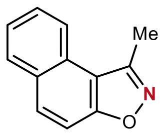

5f was prepared according to the general procedure V using **4f** (54.7 mg, 0.300 mmol, 1.0 equiv.), *N*-nitrosomorpholine (105 mg, 0.900 mmol, 3.0 equiv.), TsOH•H_2O (86 mg, 0.45 mmol, 1.5 equiv.) in EtOAc (3 mL) and subsequent ring closure with Et_3N (83.4 µL, 0.60 mmol, 2.0 equiv.) in dry DCE (2 mL) as well as MsCl (25.5 µL, 0.33 mmol, 1.1 equiv.) in dry DCE (1 mL). After purification by flash chromatography (*n*-pentane/EtOAc = 20/1), **5f** was obtained as a colourless oil (33.0 mg, 61%).

TLC: 0.50 (*n*-pentane/EtOAc = 10/3).

^{1}H NMR (400 MHz, $CDCl_3$) δ 8.51 – 8.38 (m, 1H), 7.95 (dt, *J* = 8.4, 1.0 Hz, 1H), 7.75 (d, *J* = 8.9 Hz, 1H), 7.67 – 7.61 (m, 2H), 7.52 (ddd, *J* = 8.2, 6.9, 1.3 Hz, 1H), 2.74 (s, 3H).

^{13}C NMR (101 MHz, $CDCl_3$) δ 163.0, 148.2, 136.7, 131.1, 128.6, 127.0, 126.4, 125.4, 125.2, 122.0, 110.8, 14.8.

HRMS (EI) m/z: [M+H$^+$] Calculated for $C_{12}H_9NOH$ 184.07569; Found 184.07571.

IR (neat): 1589 (m), 1570 (w), 1380 (m), 1371 (m), 1285 (w), 1271 (m), 1224 (m), 1172 (w), 1005 (m), 924 (w), 879 (w), 800 (s), 747 (m).

2-Methylnaphtho[1,2-*d*]oxazole 6f

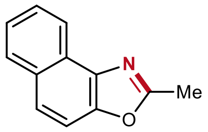

6f was prepared according to the general procedure VI using **4f** (54.7 mg, 0.300 mmol, 1.0 equiv.), *N*-nitrosomorpholine (105 mg, 0.900 mmol, 3.0 equiv.), TsOH•H_2O (86 mg, 0.45 mmol, 1.5 equiv.) in EtOAc (3 mL) and subsequent ring closure with Ms_2O (78.4 mg, 0.45 mmol, 1.5 equiv.). After purification by flash chromatography (*n*-pentane/EtOAc = 20/1), **6f** was obtained as colourless oil (37.5 mg, 69%).

TLC: 0.50 (*n*-pentane/EtOAc = 10/3).

^{1}H NMR (400 MHz, $CDCl_3$) δ 8.48 – 8.41 (m, 1H), 7.94 (ddd, *J* = 8.2, 1.2, 0.8 Hz, 1H), 7.74 (d, *J* = 8.8 Hz, 1H), 7.68 – 7.59 (m, 2H), 7.52 (ddd, *J* = 8.2, 6.8, 1.3 Hz, 1H), 2.74 (s, 3H).

^{13}C NMR (101 MHz, $CDCl_3$) δ 163.0, 148.2, 136.7, 131.1, 128.6, 127.0, 126.4, 125.4, 125.2, 122.0, 110.8, 14.8.

HRMS (ESI) m/z: [M+H$^+$] Calculated for $C_{12}H_9NOH$: 184.07569; Found 184.07569.

IR (neat): 1589 (w), 1371 (w), 1285 (w), 1271 (m), 1224 (m), 1172 (w), 1005 (w), 923 (w), 879 (w), 799 (s), 745 (w), 696 (m), 560 (w).

***N*-(2-(Benzo[*d*]isoxazol-3-yl)ethyl)-2,2,2-trifluoroacetamide 5g**

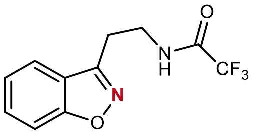

5g was prepared according to the general procedure V using **4g** (77.2 mg, 0.300 mmol, 1.0 equiv.), *N*-nitrosomorpholine (105 mg, 0.900 mmol, 3.0 equiv.), TsOH•H_2O (86 mg, 0.45 mmol, 1.5 equiv.) in EtOAc (3 mL) and subsequent ring closure with Et_3N (83.4 µL, 0.60 mmol, 2.0 equiv.) in dry DCE (2 mL) as well as MsCl (25.5 µL, 0.33 mmol, 1.1 equiv.) in dry DCE (1 mL). After purification by flash chromatography (*n*-pentane/EtOAc = 10/1), **5g** was obtained as a white solid (17.0 mg, 23%).

TLC: 0.48 (*n*-pentane/EtOAc = 10/3).

^{1}H NMR (400 MHz, $CDCl_3$) δ 7.68 – 7.63 (m, 1H), 7.61 – 7.57 (m, 2H), 7.39 – 7.30 (m, 1H), 7.18 – 7.12 (m, 1H), 3.95 (q, *J* = 6.2 Hz, 2H), 3.27 (t, *J* = 6.2 Hz, 2H).

^{13}C NMR (101 MHz, $CDCl_3$) δ 163.2, 157.6 (q, *J* = 37.3 Hz), 156.2, 130.6, 123.9, 121.4, 121.0, 114.5 (q, *J* = 289.0 Hz), 110.2, 37.1, 25.2.

^{19}F NMR (376 MHz, $CDCl_3$) δ -76.0.

HRMS (ESI) m/z: [M+H$^+$] Calculated for $C_{11}H_9N_2O_2F_3Na$: 281.05083; Found 281.05074

IR (neat): 1701 (s), 1611 (w), 1561 (m), 1519 (w), 1441 (w), 1208 (s), 1180 (s), 1155 (s), 876 (w), 749 (s).

Mp: 89-90 °C.

_N_-(2-(Benzo[_d_]oxazol-2-yl)ethyl)-2,2,2-trifluoroacetamide 6g

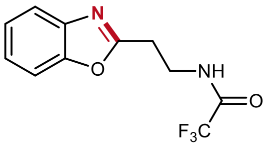

6g was prepared according to the general procedure V using **4g** (77.2 mg, 0.300 mmol, 1.0 equiv.), *N*-nitrosomorpholine (105 mg, 0.900 mmol, 3.0 equiv.), TsOH•H_2O (86 mg, 0.45 mmol, 1.5 equiv.) in EtOAc (3 mL) and subsequent ring closure with Ms_2O (78.4 mg, 0.45 mmol, 1.5 equiv.). After purification by flash chromatography (*n*-pentane/EtOAc = 8/1), **6g** was obtained as a white solid (63.5 mg, 82%).

TLC: 0.20 (*n*-pentane/EtOAc = 10/3).

^{1}H NMR (300 MHz, $CDCl_3$) δ 7.78 – 7.62 (m, 2H), 7.55 – 7.46 (m, 1H), 7.39 – 7.30 (m, 2H), 3.92 (q, *J* = 6.0 Hz, 2H), 3.24 – 3.14 (m, 2H).

^{13}C NMR (76 MHz, $CDCl_3$) δ 164.4, 157.4 (q, *J* = 36.9 Hz), 150.9, 140.8, 125.3, 124.7, 119.8, 116.2 (q, *J* = 289.7 Hz), 110.7, 36.2, 27.9.

^{19}F NMR (282 MHz, $CDCl_3$) δ -76.1.

HRMS (ESI) m/z: [M+H $^+$] Calculated for $C_{11}H_9N_2O_2F_3Na$: 281.05083; Found 281.05074

IR (neat): 1726 (m), 1565 (w), 1458 (w), 1222 (w), 1157 (s), 982 (w), 761 (m), 746 (s), 724 (m).

Mp: 141-143 °C.

3-(((Triisopropylsilyl)oxy)methyl)benzo[_d_]isoxazole 5h

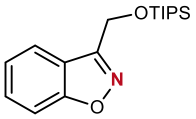

5h was prepared according to the general procedure V using **4h** (91.3 mg, 0.300 mmol, 1.0 equiv.), *N*-nitrosomorpholine (105 mg, 0.900 mmol, 3.0 equiv.), TsOH•H_2O (86 mg, 0.45 mmol, 1.5 equiv.) in EtOAc (3 mL) and subsequent ring closure with Et_3N (83.4 µL, 0.60 mmol, 2.0 equiv.) in dry DCE (2 mL) as well as MsCl (25.5 µL, 0.33 mmol, 1.1 equiv.) in dry DCE (1 mL). After purification by flash chromatography (*n*-pentane/Et_2O = 40/1), **5h** was obtained as a colourless oil (56.8 mg, 62%).

TLC: 0.80 (*n*-pentane/Et_2O = 20/1).

^{1}H NMR (400 MHz, $CDCl_3$) δ 7.95 – 7.87 (m, 1H), 7.60 – 7.50 (m, 2H), 7.31 (ddd, *J* = 8.0, 5.9, 2.0 Hz, 1H), 5.19 (s, 2H), 1.28 – 1.15 (m, 3H), 1.09 (d, *J* = 7.1 Hz, 18H).

^{13}C NMR (101 MHz, $CDCl_3$) δ 163.4, 158.2, 129.9, 123.5, 122.9, 121.3, 109.8, 58.4, 18.1, 12.0.

HRMS (ESI) m/z: [M+Na$^+$] Calculated for $C_{17}H_{27}NO_2SiNa$: 328.17033; Found 328.17042.

IR (neat): 2943 (w), 2866 (w), 1131 (w), 1103 (m), 1066 (w), 1009 (w), 881 (m), 866 (w), 806 (m), 747 (s), 682 (s), 660 (m).

2-(5-Aminoisoxazol-3-yl)phenol 5i

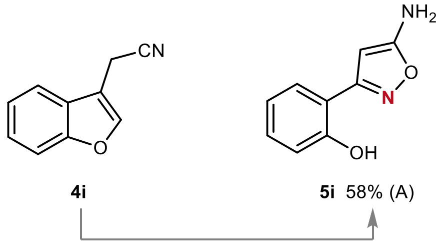

4i 5i 58% (A)

5i was prepared according to the general procedure V using **4i** (47.2 mg, 0.300 mmol, 1.0 equiv.), *N*-nitrosomorpholine (105 mg, 0.900 mmol, 3.0 equiv.), TsOH•H_2O (86 mg, 0.45 mmol, 1.5 equiv.) in EtOAc (3 mL). After purification by flash chromatography (*n*-pentane/EtOAc = 5/1), **5i** was obtained as a colourless solid (30.7 mg, 58%).

TLC: 0.24 (*n*-pentane/EtOAc= 10/3).

^{1}H NMR (400 MHz, $CDCl_3$) δ 9.57 (s, 1H), 7.39 (dd, *J* = 7.8, 1.7 Hz, 1H), 7.34 – 7.29 (m, 1H), 7.04 (dd, *J* = 8.4, 1.2 Hz, 1H), 6.97 – 6.90 (m, 1H), 5.51 (s, 1H), 4.61 (s, 2H).

^{13}C NMR (101 MHz, $CDCl_3$) δ 168.2, 164.4, 156.8, 131.5, 128.2, 119.8, 117.5, 114.1, 77.8.

HRMS (ESI) m/z: [M+H $^+$] Calculated for $C_9H_8N_2O_2Na$: 199.04780; Found 199.04777.

IR (neat): 1634 (s), 1577 (m), 1496 (s), 1463 (w), 1437 (m), 1388 (w), 1296 (w), 1248 (m), 752 (s), 646 (w).

2943 (w), 2866 (w), 1131 (w), 1103 (m), 1066 (w), 1009 (w), 881 (m), 866 (w), 806 (m), 747 (s), 682 (s), 660 (m).

Mp: 94-95 °C.

(Benzo[*d*]oxazol-2-ylmethyl)diphenylphosphine oxide 6j

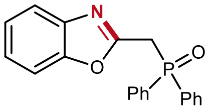

6j was prepared according to the general procedure VI using **4j** (99.7 mg, 0.300 mmol, 1.0 equiv.), *N*-nitrosomorpholine (105 mg, 0.900 mmol, 3.0 equiv.), TsOH•H_2O (86 mg, 0.45 mmol, 1.5 equiv.) in EtOAc (3 mL) and subsequent ring closure with Ms_2O (78.4 mg, 0.45 mmol, 1.5 equiv.). After purification by flash chromatography (EtOAc), **6j** was obtained as a yellow solid (68.0 mg, 68%).

TLC: 0.12 (*n*-pentane/EtOAc = 1/2).

^{1}H NMR (400 MHz, $CDCl_3$) δ 7.85 – 7.76 (m, 4H), 7.63 – 7.57 (m, 1H), 7.53 (ddt, *J* = 8.3, 4.6, 1.5 Hz, 2H), 7.49 – 7.42 (m, 4H), 7.41 – 7.36 (m, 1H), 7.28 – 7.22 (m, 2H), 4.07 (d, *J* = 14.5 Hz, 2H).

^{13}C NMR (101 MHz, $CDCl_3$) δ 158.9 (d, *J* = 8.7 Hz), 151.3, 141.3 (d, *J* = 1.7 Hz), 132.6 (d, *J* = 2.9 Hz), 132.1, 131.3 (d, *J* = 9.7 Hz), 128.9 (d, *J* = 12.3 Hz), 125.1, 124.5, 119.9, 110.8, 33.2 (d, *J* = 63.2 Hz).

^{31}P NMR (162 MHz, $CDCl_3$) δ 27.2.

HRMS (ESI) m/z: [M+Na $^+$] Calculated for $C_{20}H_{16}NO_2PNa$: 356.08109; Found 356.08074.

IR (neat): 1611 (w), 1565 (m), 1454 (m), 1438 (m), 1242 (w), 1200 (s), 1121 (m), 944 (w), 732 (s), 695 (s), 545 (s), 516 (m).

Mp: 153-155 °C.

Methyl 3-(3-((2-(benzo[*d*]oxazol-2-yl)ethyl)amino)-3-oxopropyl)-1*H*-indole-1-carboxylate 6k

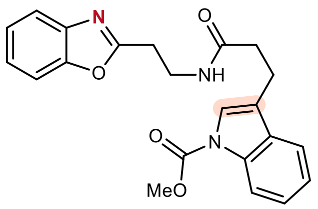

6k was prepared according to the general procedure VI using **4k** (117 mg, 0.300 mmol, 1.0 equiv.), *N*-nitrosomorpholine (105 mg, 0.900 mmol, 3.0 equiv.), TsOH•H_2O (86 mg, 0.45 mmol, 1.5 equiv.) in EtOAc (3 mL) and subsequent ring closure with Ms_2O (78.4 mg, 0.45 mmol, 1.5 equiv.). After purification by flash chromatography (EtOAc) crude **6k** was obtained, which was further purified by reversed-phase MPLC (MeCN/water, v/v ratio was increased gradient from 20:80 to 65:35 over 9 min, then kept at 65:35 for 3 min, then increased from 65:35 to 90:10 over 5 min and finally kept at 90:10 for 5 min) to give **6k** as a brown solid (58.7 mg, 50%).

TLC: 0.12 (*n*-pentane/EtOAc = 1/2).

^{1}H NMR (400 MHz, $CDCl_3$) δ 8.01 (d, J = 7.7 Hz, 1H), 7.60 – 7.54 (m, 1H), 7.51 – 7.48 (m, 1H), 7.47 – 7.42 (m, 1H), 7.36 (s, 1H), 7.33 – 7.28 (m, 2H), 7.30 – 7.21 (m, 1H), 7.23 – 7.18 (m, 1H), 6.41 (t, J = 6.2 Hz, 1H), 3.95 (s, 3H), 3.75 (q, J = 6.1 Hz, 2H), 3.09 – 2.94 (m, 4H), 2.56 (t, J = 7.5 Hz, 2H).

^{13}C NMR (101 MHz, $CDCl_3$) δ 172.3, 165.3, 151.5, 150.9, 141.1, 135.6, 130.4, 125.0, 124.8, 124.5, 122.9, 122.4, 120.6, 119.7, 119.1, 115.3, 110.7, 53.8, 36.5, 35.9, 28.8, 21.1.

HRMS (ESI) m/z: [M+Na$^+$] Calculated for $C_{22}H_{21}N_3O_4Na$: 414.14243; Found 414.14224.

IR (neat): 1731 (m), 1647 (m), 1544 (w), 1454 (s), 1377 (s), 1308 (w), 1253 (m), 1154 (w), 1087 (m), 743 (s).

Mp: 102-103 °C.

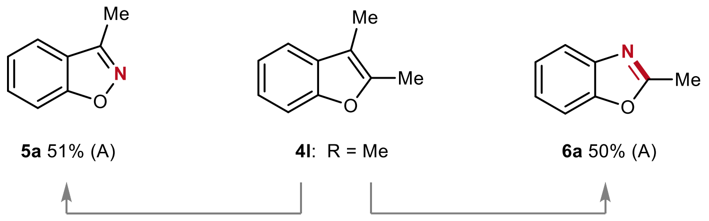

5a was prepared according to the general procedure V using **4l** (43.8 mg, 0.300 mmol, 1.0 equiv.), *N*-nitrosomorpholine (105 mg, 0.900 mmol, 3.0 equiv.), TsOH•H_2O (86 mg, 0.45 mmol, 1.5 equiv.) in EtOAc (3 mL) and subsequent ring closure with Et_3N (83.4 µL, 0.60 mmol, 2.0 equiv.) in dry DCE (2 mL) as well as MsCl (25.5 µL, 0.33 mmol, 1.1 equiv.) in dry DCE (1 mL). After purification by flash chromatography (*n*-pentane/EtOAc = 50/1), **5a** was obtained as a colourless oil (21.0 mg, 51%).

6a was prepared according to the general procedure V using **4l** (43.8 mg, 0.300 mmol, 1.0 equiv.), *N*-nitrosomorpholine (105 mg, 0.900 mmol, 3.0 equiv.), TsOH•H_2O (86 mg, 0.45 mmol, 1.5 equiv.) in EtOAc (3 mL) and subsequent ring closure with Ms_2O (78.4 mg, 0.45 mmol, 1.5 equiv.). After purification by flash chromatography (*n*-pentane/EtOAc = 50/1), **6a** was obtained as a colourless oil (20.0 mg, 50%).

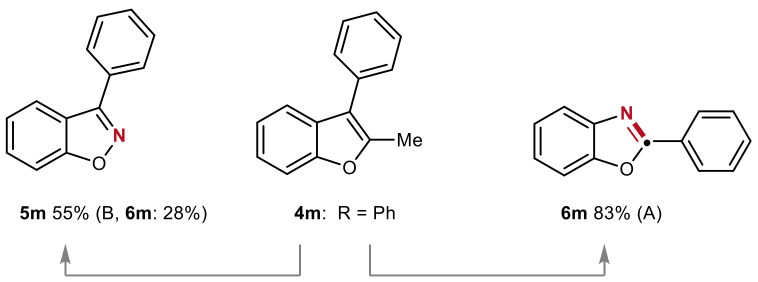

5m and **6m** were prepared according to the general procedures IX and X using **4m** (208 mg, 1.0 mmol, 1.0 equiv.) with silica gel (2.4 g) and PCC (1.12 g, 5.4 mmol, 5.4 equiv.) in DCM (50 mL) for oxidative cleavage. Subsequent aminolysis afforded upon treatment with NH_3 (5 mL, 7 M in MeOH) at room temperature the crude ketimine (260.3 mg) that was dircetyl used for the synthesis of **5m** and **6m**, see below.

3-Phenylbenzo[*d*]isoxazole 5m

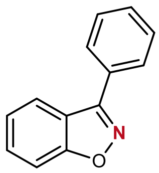

5m was prepared according to the general procedure IX using crude ketimine (52.06 mg, 0.2000 mmol based on **4m**), NCS (40 mg, 0.30 mmol, 1.5 equiv.) and K_2CO_3 (55.3 mg, 0.400 mmol, 2.0 equiv.) in THF (1 mL). After purification by flash chromatography (*n*-pentane/EtOAc = 20/1), **5m** was obtained as a colourless oil (21.1 mg, 55%) along with **6m** that was isolated as a yellow solid (10.7 mg, 28%).

^{1}H NMR (300 MHz, $CDCl_3$) δ 8.05 – 7.89 (m, 3H), 7.70 – 7.52 (m, 5H), 7.44 – 7.35 (m, 1H).

^{13}C NMR (76 MHz, $CDCl_3$) δ 164.0, 157.4, 130.4, 129.9, 129.3, 129.1, 128.2, 124.0, 122.3, 120.6, 110.3.

Analytic data are in agreement with those reported in the literature.[44]

2-Phenylbenzo[*d*]oxazole 6m

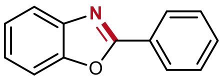

6m was prepared according to the general procedure X using crude ketimine (52.06 mg, 0.2000 mmol based on **4m**), NaClO (0.8 mL) in isopropyl alcohol (1 mL). After purification by flash chromatography (*n*-pentane/EtOAc = 20/1), **6m** was obtained as a yellow solid (32.4 mg, 83%).

^{1}H NMR (300 MHz, $CDCl_3$) δ 8.32 – 8.20 (m, 2H), 7.82 – 7.75 (m, 1H), 7.62 – 7.57 (m, 1H), 7.56 – 7.47 (m, 3H), 7.40 – 7.31 (m, 2H).

^{13}C NMR (76 MHz, $CDCl_3$) δ 163.1, 150.8, 142.1, 131.5, 128.9, 127.6, 127.2, 125.1, 124.6, 120.0, 110.6.

Analytic data are in agreement with those reported in the literature.[45]

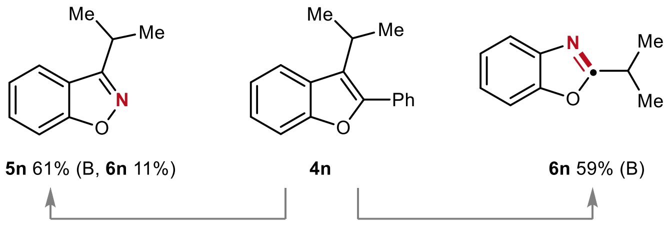

5n and **6n** were prepared according to the general procedures IX and X using **4n** (180 mg, 0.7 mmol, 1.0 equiv.) with silica gel (1.8 g) and PCC (0.90 g, 2.7 mmol, 5.4 equiv.) in DCM (40 mL) for oxidative cleavage. Subsequent aminolysis upon treatment with NH_3 (3.5 mL, 7 M in MeOH) at 70 °C afforded the crude ketimine (177.6 mg) that was used without further purification used for the synthesis of **5n** and **6n**.

3-Isopropylbenzo[*d*]isoxazole 5n

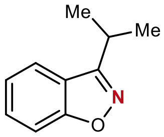

5n was prepared according to the general procedure IX using crude ketimine (50.8 mg, 0.200 mmol based on **4n**), NCS (40 mg, 0.30 mmol, 1.5 equiv.), ^{t}BuOK (44.9 mg, 0.400 mmol, 2.0 equiv.) and 18-crown-6 (53.0 mg, 0.200 mmol, 1.0 equiv.) in THF (1 mL). After purification by flash chromatography (*n*-pentane/Et_2O = 25/1), **5n** was obtained as a colourless oil (19.6 mg, 61%) along with **6n** that was isolated as a yellow oil (3.5 mg, 11%).

^{1}H NMR (400 MHz, $CDCl_3$) δ 7.72 (dd, J = 8.0, 1.1 Hz, 1H), 7.58 – 7.49 (m, 2H), 7.32 – 7.27 (m, 1H), 3.46 – 3.36 (m, 1H), 1.50 (d, J = 7.0 Hz, 6H).

^{13}C NMR (101 MHz, $CDCl_3$) δ 163.3, 163.3, 129.7, 123.1, 121.8, 121.0, 110.2, 27.1, 21.3.

Analytic data are in agreement with those reported in the literature.[46]

2-Isopropylbenzo[*d*]oxazole 6n

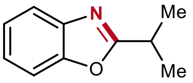

6n was prepared according to the general procedure X using crude ketimine (50.8 mg, 0.200 mmol based on **4n**), NaClO (0.8 mL) in isopropyl alcohol (1 mL). After purification by flash chromatography (*n*-pentane/EtOAc = 20/1), **6n** was obtained as a yellow oil (19.0 mg, 59%).

^{1}H NMR (300 MHz, $CDCl_3$) δ 7.74 – 7.65 (m, 1H), 7.52 – 7.42 (m, 1H), 7.35 – 7.26 (m, 2H), 3.33 – 3.13 (m, 1H), 1.46 (dd, J = 7.0, 0.7 Hz, 6H).

^{13}C NMR (76 MHz, $CDCl_3$) δ 171.4, 150.8, 141.3, 124.5, 124.1, 119.7, 110.4, 29.0, 20.4.

Analytic data are in agreement with those reported in the literature.[47]

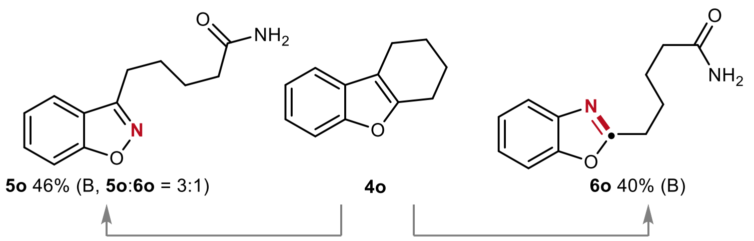

5o and **6o** were prepared according to the general procedures IX and X using **4o** (172 mg, 1.00 mmol, 1.0 equiv.) with silica gel (2.4 g) and PCC (1.17 g, 5.40 mmol, 5.4 equiv.) in DCM (50 mL) for oxidative cleavage. Subsequent aminolysis by treatment with NH_3 (5 mL, 7 M in MeOH) at 70 °C afforded the crude ketimine (180 mg) that was used without any further purification for the synthesis of **5o** and **6o**.

5-(Benzo[*d*]isoxazol-3-yl)pentanamide 5o

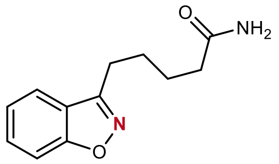

5o was prepared according to the general procedure IX using crude ketimine (90 mg, 0.50 mmol based on **4o**), NCS (100 mg, 0.750 mmol, 1.5 equiv.), K_2CO_3 (122 mg, 1.00 mmol, 2.0 equiv.) and 18-crown-6 (132 mg, 0.500 mmol, 1.0 equiv.) in THF (2.5 mL). After purification by flash chromatography (EtOAc) crude **5o** was obtained, which was further purified by reversed-phase MPLC (MeCN/water, v/v ratio was increased gradient from 20:80 to 65:35 over 9 min, then kept at 65:35 for 3 min, then increased from 65:35 to 90:10 over 5 min and finally kept at 90:10 for 5 min) to give **5o** as a white solid (37.9 mg, 35%) along with **5o** that was isolated as a white solid (12.3 mg, 11%).

TLC: 0.70 (*n*-pentane/EtOAc = 1/1).

^{1}H NMR (300 MHz, $CDCl_3$) δ 7.71 – 7.60 (m, 1H), 7.57 – 7.47 (m, 2H), 7.35 – 7.27 (m, 1H), 5.72 (d, J = 25.1 Hz, 2H), 3.12 – 2.91 (m, 2H), 2.36 – 2.17 (m, 2H), 2.00 – 1.86 (m, 2H), 1.84 – 1.69 (m, 2H).

^{13}C NMR (76 MHz, $CDCl_3$) δ 175.3, 163.1, 158.4, 130.0, 123.4, 121.8, 121.5, 110.1, 35.5, 27.1, 25.20, 25.17.

HRMS (ESI) m/z: [M+Na$^+$] Calculated for $C_{12}H_{14}N_2O_2Na$: 241.09475; Found 241.09462.

IR (neat): 1631 (s), 1462 (m), 1423 (s), 1327 (m), 1263 (w), 859 (w), 774 (w), 745 (s), 698 (m).

Mp: 115-116 °C.

5-(Benzo[*d*]oxazol-2-yl)pentanamide 6o

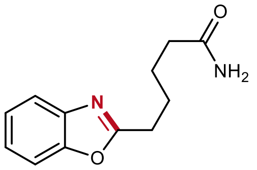

6o was prepared according to the general procedure X using crude ketimine (90 mg, 0.50 mmol based on **4o**), NaClO (2 mL) in isopropyl alcohol (2.5 mL). After purification by flash chromatography (*n*-pentane/EtOAc = 20/1), **6o** was obtained as a white solid (43.6 mg, 40%).

TLC: 0.54 (*n*-pentane/EtOAc = 1/1).

^{1}H NMR (400 MHz, $CDCl_3$) δ 7.67 – 7.60 (m, 1H), 7.48 – 7.40 (m, 1H), 7.32 – 7.25 (m, 2H), 4.64 – 4.57 (m, 1H), 3.22 (q, J = 6.6 Hz, 2H), 2.94 (t, J = 7.4 Hz, 2H), 1.96 – 1.85 (m, 2H), 1.70 – 1.56 (m, 2H). (One N-H is not detected.)

^{13}C NMR (101 MHz, $CDCl_3$) δ 167.0, 158.4, 150.9, 141.4, 124.7, 124.3, 119.6, 110.5, 40.1, 29.8, 28.3, 24.0.

HRMS (ESI) m/z: [M+Na$^+$] Calculated for $C_{12}H_{14}N_2O_2Na$: 241.09475; Found 241.09473.

IR (neat): 3336 (w), 2934 (w), 1613 (s), 1568 (sm), 1455 (m), 1242 (m), 1153 (w), 946 (w), 837 (w), 742 (m).

Mp: 179-180 °C.

Methyl (*S*)-3-(3-((2-(6-methoxynaphthalen-2-yl)propanoyl)oxy)propyl)-1*H*-indazole-1-carboxylate (Moc-**2r)**

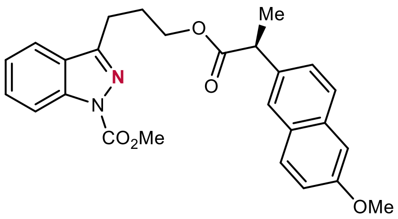

Moc-**2r** was prepared according to the general procedure III using Moc-**1r** (66 mg, 0.15 mmol, 1.0 equiv.), *N*-nitrosomorpholine (52.5 mg, 0.450 mmol, 3.0 equiv.), TsOH•H_2O (43 mg, 0.23 mmol, 1.5 equiv.) in EtOAc (2 mL) and subsequent ring closure with PPh_3 (13.0 mg, 0.048 mmol), DEAD (0.020 mL, 0.048 mmol, 40 wt.% in toluene) in dry THF (0.5 mL). After purification by flash chromatography (*n*-pentane/EtOAc = 10/1), Moc-**2r** was obtained as colourless oil (10 mg, 15%). The yield of oxime intermediate was determined by ^{1}H NMR with dibromomethane as internal standard (16%). In this case, indole Moc-**1r** was recovered (28.5 mg, 49%).

TLC: 0.40 (*n*-pentane/EtOAc = 5/2).

^{1}H NMR (400 MHz, $CDCl_3$) δ 8.18 (d, J = 8.4 Hz, 1H), 7.75 – 7.65 (m, 3H), 7.53 – 7.47 (m, 1H), 7.41 (dd, J = 8.5, 2.1 Hz, 1H), 7.32 – 7.24 (m, 1H), 7.18 – 7.06 (m, 3H), 4.25 – 4.14 (m, 2H), 4.10 (s, 3H), 3.91 (s, 3H), 3.84 (q, J = 7.1 Hz, 1H), 2.96 – 2.85 (m, 2H), 2.17 – 2.04 (m, 2H), 1.57 (s, 3H).

^{13}C NMR (101 MHz, $CDCl_3$) δ 174.7, 157.8, 152.2, 151.3, 140.7, 135.9, 133.9, 129.37, 129.08, 129.1, 127.3, 126.4, 126.1, 125.4, 123.8, 120.3, 119.2, 114.7, 105.7, 64.1, 55.5, 54.5, 45.6, 27.7, 23.6, 18.5.

HRMS (ESI) m/z: [M+Na$^+$] Calculated for $C_{26}H_{26}N_2O_5Na$: 469.17339; found: 469.17329.

IR (neat): 1731 (s), 1606 (w), 1506 (w), 1445 (m), 1395 (w), 1374 (m), 1244 (m), 1231 (m), 1089 (w), 1031 (w), 761 (m).

Methyl (*S*)-2-(3-((2-(6-methoxynaphthalen-2-yl)propanoyl)oxy)propyl)-1*H*-benzo[d]imidazole-1-carboxylate (Moc-**3r)**

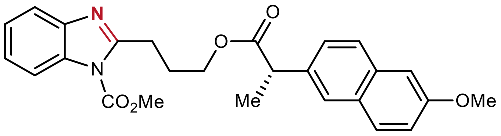

Moc-**3r** was prepared according to the general procedure IV using Moc-**1r** (66 mg, 0.15 mmol, 1.0 equiv.), *N*-nitrosomorpholine (52.5 mg, 0.450 mmol, 3.0 equiv.), TsOH•H_2O (43 mg, 0.23 mmol, 1.5 equiv.) in EtOAc (2 mL)

and subsequent ring closure with Et_3N (5.0 µL, 0.04 mmol), 2,6-lutidine (3.0 µL, 0.04 mmol) in dry DCE (0.5 mL) as well as MsCl (2.5 µL, 0.03 mmol) in dry DCE (0.5 mL). After purification by flash chromatography (*n*-pentane/EtOAc = 10/1), Moc-**3r** was obtained as colourless oil (10 mg, 15%). The yield of oxime intermediate was determined by ^1H NMR with dibromomethane as internal standard (16%). In this case, indole Moc-**1r** was recovered (30.2 mg, 52%).

TLC: 0.40 (*n*-pentane/EtOAc = 5/2).

^{1}H NMR (599 MHz, $CDCl_3$) δ 7.90 – 7.86 (m, 1H), 7.69 – 7.62 (m, 4H), 7.38 (dd, *J* = 8.4, 1.9 Hz, 1H), 7.33 – 7.31 (m, 2H), 7.10 (dd, *J* = 8.9, 2.5 Hz, 1H), 7.06 (d, *J* = 2.6 Hz, 1H), 4.26 (t, *J* = 6.2 Hz, 2H), 3.99 (s, 3H), 3.90 (s, 3H), 3.82 (q, *J* = 7.1 Hz, 1H), 3.21 – 3.08 (m, 2H), 2.26 – 2.18 (m, 2H), 1.57 (s, 3H).

^{13}C NMR (151 MHz, $CDCl_3$) δ 174.8, 157.7, 155.6, 151.0, 142.3, 135.9, 133.8, 132.9, 129.4, 129.1, 127.2, 126.4, 126.1, 124.7, 124.6, 119.8, 119.0, 115.0, 105.7, 64.2, 55.4, 54.3, 45.6, 27.8, 26.3, 18.6.

HRMS (ESI) m/z: [M+Na$^+$] Calculated for $C_{26}H_{26}N_2O_5Na$: 469.17339; found: 469.17311.

IR (neat): 1751 (m), 1733 (s), 1456 (s), 1439 (m), 1351 (m), 1261 (m), 1177 (s), 1121 (w), 1089 (w), 1030 (w), 764 (m), 746 (w).

3-(1*H*-Indazol-3-yl)propyl (*S*)-2-(6-methoxynaphthalen-2-yl)propanoate 2r

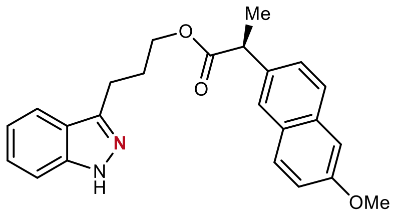

2r was prepared according to the general procedure VII using **1r** (140 mg, 0.360 mmol, 1.0 equiv.) in CH_3CN (6 mL) with $NaIO_4$ (336 mg, 1.45 mmol, 4.0 equiv.) in water (6 mL) heating at 60 °C for oxidative cleavage. Deformylation was conducted under acidic conditions using conc. HCl (45 µL) in MeOH (10 mL) at r.t. for 24 h to give 2-aminophenone (100 mg, 67%, 0.250 mmol). The ring closure step was conducted with 2-aminophenone (37.7 mg, 0.100 mmol) and $Me_3C_6H_2SO_2ONHBoc$ (47.3 mg, 0.150 mmol) in HFIP (1 mL). After purification by flash chromatography (*n*-pentane/EtOAc = 4/1), **2r** was obtained as a brown solid (30.1 mg, 52% overall yield).

TLC: 0.22 (*n*-pentane/EtOAc = 2/1).

^{1}H NMR (400 MHz, $CDCl_3$) δ 9.98 (s, 1H), 7.71 (d, *J* = 2.9 Hz, 1H), 7.69 (d, *J* = 2.2 Hz, 2H), 7.45 – 7.36 (m, 3H), 7.35 – 7.29 (m, 1H), 7.14 (dd, *J* = 8.9, 2.5 Hz, 1H), 7.11 (d, *J* = 2.5 Hz, 1H), 7.05 – 6.97 (m, 1H), 4.20 (t, *J* = 6.3 Hz, 2H), 3.91 (s, 3H), 3.86 (q, *J* = 7.1 Hz, 1H), 3.00 – 2.91 (m, 2H), 2.19 – 2.08 (m, 2H), 1.59 (d, *J* = 7.1 Hz, 3H).

^{13}C NMR (101 MHz, $CDCl_3$) δ 174.8, 157.8, 146.3, 141.2, 135.9, 133.8, 129.4, 129.1, 127.3, 126.8, 126.4, 126.1, 122.3, 120.4, 120.1, 119.1, 109.8, 105.8, 64.3, 55.4, 45.7, 28.0, 23.4, 18.6.

HRMS (ESI) m/z: [M+Na$^+$] Calculated for $C_{24}H_{24}N_2O_3Na$: 411.16791; found: 411.16779.

IR (neat): 1727 (m), 1605 (m), 1504 (m), 1485 (m), 1452 (m), 1391 (m), 1263 (m), 1230 (m), 1175 (m), 1030 (m), 853 (m), 811 (m), 744 (m), 475 (m).

Mp: 87-89 °C.

3-(1-Tosyl-1*H*-benzo[d]imidazol-2-yl)propyl (*S*)-2-(6-methoxynaphthalen-2-yl)propanoate Ts-3r

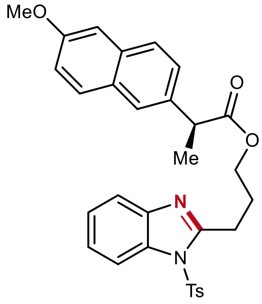

Ts-3r was prepared according to the general procedure VIII using 2-aminophenone intermediate from the first step of **2r**. The ring closure step was conducted with 2-aminophenone (60 mg, 0.15 mmol, 1.0 equiv.) *via* oxime as intermediate. After second step's purification by flash chromatography (*n*-pentane/EtOAc = 5/1), Ts-**3r** was obtained as a colourless oil (37.4 mg, 32% overall yield). Ts-**2r** was obtained as a colourless oil (17.4 mg, 15% overall yield).

TLC: 0.44 (*n*-pentane/EtOAc = 2/1).

^{1}H NMR (400 MHz, $CDCl_3$) δ 8.03 – 7.96 (m, 1H), 7.73 – 7.66 (m, 5H), 7.64 – 7.60 (m, 1H), 7.45 – 7.39 (m, 1H), 7.36 – 7.28 (m, 2H), 7.17 – 7.05 (m, 4H), 4.33 – 4.17 (m, 2H), 3.90 (s, 3H), 3.89 – 3.82 (m, 1H), 3.25 – 3.08 (m, 2H), 2.33 (s, 3H), 2.27 – 2.19 (m, 2H), 1.59 (d, *J* = 7.1 Hz, 3H).

^{13}C NMR (101 MHz, $CDCl_3$) δ 174.8, 157.7, 154.1, 146.0, 142.0, 135.8, 135.5, 133.8, 133.3, 130.3, 129.4, 129.1, 127.3, 126.8, 126.4, 126.0, 125.0, 124.8, 120.0, 119.1, 113.7, 105.7, 64.1, 55.4, 45.6, 26.63, 26.60, 21.7, 18.7.

HRMS (ESI) m/z: [M+Na$^+$] Calculated for $C_{31}H_{30}N_2O_5SNa$: 565.17676; found: 565.17632.

IR (neat): 1728 (m), 1605 (w), 1451 (w), 1373 (m), 1263 (w), 1166 (s), 1088 (m), 1030 (m), 853 (w), 742 (m), 703 (w), 665 (s), 642 (w), 576 (s), 543 (s).

3-(1-Tosyl-1*H*-indazol-3-yl)propyl (*S*)-2-(6-methoxynaphthalen-2-yl)propanoate Ts-2r

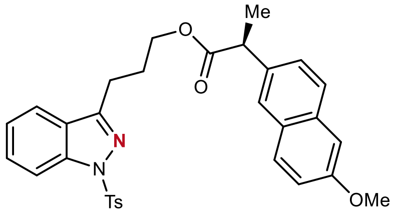

TLC: 0.26 (*n*-pentane/EtOAc = 10/3).

^{1}H NMR (400 MHz, $CDCl_3$) δ 8.12 (dd, *J* = 8.5, 1.0 Hz, 1H), 7.82 – 7.76 (m, 2H), 7.71 – 7.64 (m, 3H), 7.50 – 7.45 (m, 1H), 7.39 (dd, *J* = 8.4, 1.9 Hz, 1H), 7.21 – 7.10 (m, 5H), 7.09 – 7.07 (m, 1H), 4.13 – 4.00 (m, 2H), 3.90 (s, 3H), 3.83 (q, *J* = 7.2 Hz, 1H), 2.85 – 2.74 (m, 2H), 2.30 (s, 3H), 2.10 – 1.96 (m, 2H), 1.58 (s, 3H).

^{13}C NMR (101 MHz, $CDCl_3$) δ 174.7, 157.8, 153.5, 145.2, 141.4, 135.9, 134.7, 133.8, 129.8, 129.4, 129.2, 129.1, 127.5, 127.3, 126.4, 126.1, 125.6, 124.0, 120.4, 119.2, 113.6, 105.8, 63.8, 55.5, 45.6, 27.1, 23.4, 21.7, 18.5.

HRMS (ESI) m/z: [M+Na$^+$] Calculated for $C_{31}H_{30}N_2O_5SNa$: 565.17676; found: 565.17642.

IR (neat): 1729 (m), 1605 (m), 1375 (m), 1259 (w), 1174 (s), 1031 (w), 812 (w), 749 (w), 672 (m), 575 (m), 540 (m).

Methyl 3-(3-methoxy-3-oxopropyl)-1*H*-indazole-1-carboxylate Moc-**2s**

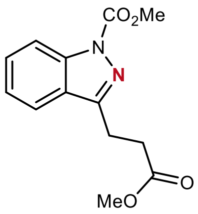

Moc-**2s** was synthesized according general procedure III from Moc-**1s** (78.3 mg, 0.300 mmol, 1.0 equiv.), *N*-nitrosomorpholine (105 mg, 0.900 mmol, 3.0 equiv.), TsOH•H_2O (86 mg, 0.45 mmol, 1.5 equiv.) in EtOAc (3 mL) and subsequent ring closure with PPh_3 (26.8 mg, 0.110 mmol), DEAD (0.05 mL, 0.10 mmol, 40 wt.% in toluene) in dry THF (0.5 mL). After purification by flash chromatography (*n*-pentane/EtOAc = 3/1), the desired compound Moc-**2s** was obtained as a white solid (9.5 mg, 12% yield). The yield of oxime intermediate was 17% (0.051 mmol). In this case, indole Moc-**1s** was recovered (33.1 mg, 42%).

TLC: 0.50 (*n*-pentane/EtOAc = 5/2).

^{1}H NMR (300 MHz, $CDCl_3$) δ 8.21 (d, *J* = 8.4 Hz, 1H), 7.73 (d, *J* = 7.8 Hz, 1H), 7.56 (ddd, *J* = 8.4, 7.2, 1.2 Hz, 1H), 7.48 – 7.34 (m, 1H), 4.12 (s, 3H), 3.69 (s, 3H), 3.32 (t, *J* = 8.1 Hz, 2H), 2.90 (t, *J* = 8.1 Hz, 2H).

^{13}C NMR (100 MHz, $CDCl_3$) δ 173.1, 151.5, 151.3, 140.8, 129.5, 125.3, 124.0, 120.5, 114.8, 54.5, 52.0, 32.5, 22.4.

HRMS (ESI) m/z: [M+Na$^+$] Calculated for $C_{13}H_{14}N_2O_4Na$: 285.08458. Found: 285.08440.

IR (neat): 1733 (s), 1443 (m), 1374 (m), 1340 (w), 1239 (m), 1152 (w), 1084 (m), 760 (m).

Mp: 100-101 °C.

Methyl 2-(3-methoxy-3-oxopropyl)-1H-benzo[*d*]imidazole-1-carboxylate Moc-**3s**

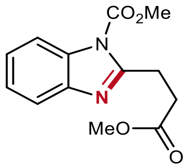

Moc-**3s** was synthesized according general procedure IV from Moc-**1s** (78.3 mg, 0.300 mmol, 1.0 equiv.), *N*-nitrosomorpholine (105 mg, 0.900 mmol, 3.0 equiv.), TsOH•H_2O (86 mg, 0.45 mmol, 1.5 equiv.) in EtOAc (3 mL) and subsequent ring closure with Et_3N (11 µL, 0.077 mmol), 2,6-lutidine (7.3 µL, 0.077 mmol) in dry DCE (0.5 mL) as well as MsCl (4.8 µL, 0.07 mmol) in dry DCE (0.5 mL). After purification by flash chromatography (*n*-pentane/EtOAc = 3/1), the desired compound Moc-**3s** was obtained as a white solid (7.9 mg, 10% yield). The yield of oxime intermediate was 17% (0.051 mmol). In this case, indole Moc-**1s** was recovered (39.4 mg, 50%).

TLC: 0.42 (*n*-pentane/EtOAc = 5/2).

^{1}H NMR (300 MHz, $CDCl_3$) δ 8.01 – 7.82 (m, 1H), 7.80 – 7.60 (m, 1H), 7.32 (dd, *J* = 6.0, 3.3 Hz, 2H), 4.12 (s, 3H), 3.72 (s, 3H), 3.53 (t, *J* = 7.2 Hz, 2H), 2.99 (t, *J* = 7.2 Hz, 2H).

^{13}C NMR (100 MHz, $CDCl_3$) δ 173.2, 154.9, 151.2, 142.3, 133.0, 124.7, 124.6, 119.9, 115.0, 54.5, 52.0, 31.1, 26.5.

HRMS (ESI) m/z: [M+Na$^+$] Calculated for $C_{13}H_{14}N_2O_4Na$: 285.08458. Found: 285.08428.

IR (neat): 1735 (s), 1456 (s), 1438 (m), 1350 (s), 1254 (w), 1170 (s), 1120 (m), 1092 (m), 764 (w), 747 (m).

Mp: 68-69 °C.

Methyl 3-(1*H*-indazol-3-yl)propanoate 2s

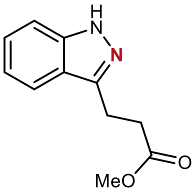

2s was prepared according to the general procedure VII using **1s** (204 mg, 1.00 mmol, 1.0 equiv.) in MeOH (20 mL) and $NaIO_4$ (855.5 mg, 1.250 mmol, 2.5 equiv.) in H_2O (20 mL) at room temperature for 24 h. After deformylation with conc. HCl (0.15 mL) at 70 °C for 1 h, 2-aminophenone was obtained after column chromatography (SiO_2, *n*-pentane/EtOAc = 10/3) as a yellow oil (180 mg, 87%). 2-Aminophenone (0.20 mmol) was used for the second step with $Me_3C_6H_2SO_2ONHBoc$ (95 mg, 0.30 mmol) in HFIP (1 mL). Through purification of step 2 by flash chromatography (*n*-pentane/EtOAc = 10/3), **2s** was obtained as a light white solid (31.3 mg, 67% overall yield).

TLC: 0.30 (*n*-pentane/EtOAc = 5/2).

^{1}H NMR (400 MHz, $CDCl_3$) δ 7.72 (dd, *J* = 8.1, 1.0 Hz, 1H), 7.48 – 7.43 (m, 1H), 7.40 – 7.34 (m, 1H), 7.18 – 7.12 (m, 1H), 3.69 (s, 3H), 3.40 – 3.31 (m, 2H), 2.91 (t, *J* = 7.6 Hz, 2H). (N-H was not detected)

^{13}C NMR (76 MHz, $CDCl_3$) δ 173.7, 145.4, 141.2, 126.9, 122.1, 120.5, 120.1, 110.0, 51.9, 32.9, 22.3.

HRMS (ESI) m/z: [$M+Na^+$] Calculated for $C_{11}H_{12}N_2O_2Na$: 227.0791. Found: 227.07870.

IR (neat): 1733 (s), 1717 (s), 1437 (m), 1362 (w), 1343 (m), 1200 (m), 1166 (s), 1076 (w), 1006 (w), 743 (s).

Mp: 45-46 °C.

Methyl 3-(1-tosyl-1H-benzo[*d*]imidazol-2-yl)propanoate Ts-**3s**

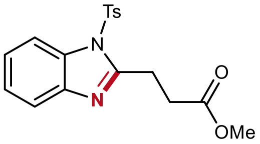

Ts-3s was prepared according to the general procedure VIII using **1s** (204 mg, 1.00 mmol, 1.0 equiv.) in MeOH (20 mL) and $NaIO_4$ (855.5 mg, 1.250 mmol, 2.5 equiv.) in H_2O (20 mL) at room temperature for 24 h. After deformylation with conc. HCl (0.15 mL) at 70 °C for 1 h, 2-aminophenone was obtained after column chromatography (SiO_2, *n*-pentane/EtOAc = 10/3) as a yellow oil (180 mg, 87%). 2-Aminophenone (0.20 mmol) was used for the second step *via* oxime as intermediate. Through purification by flash chromatography (DCM/EtOAc = 20/1), Ts-**3s** was obtained as a white solid (27.9 mg, 39% over two steps) and by-product Ts-**2s** was also obtained as a white solid (10.8 mg, 15% overall yield).

TLC: 0.54 (*n*-pentane/EtOAc = 5/2).

^{1}H NMR (300 MHz, $CDCl_3$) δ 8.04 – 7.96 (m, 1H), 7.88 – 7.80 (m, 2H), 7.66 – 7.59 (m, 1H), 7.37 – 7.25 (m, 4H), 3.70 (s, 3H), 3.51 (t, *J* = 7.2 Hz, 2H), 3.00 (t, *J* = 7.2 Hz, 2H), 2.39 (s, 3H).

^{13}C NMR (76 MHz, $CDCl_3$) δ 173.0, 153.4, 146.1, 142.0, 135.4, 133.3, 130.4, 127.0, 125.0, 124.7, 120.1, 113.6, 52.0, 31.0, 25.3, 21.8.

HRMS (ESI) m/z: [$M+Na^+$] Calculated for $C_{18}H_{18}N_2O_4SNa$: 381.08795. Found: 381.08769.

IR (neat): 1734 (m), 1453 (w), 1437 (w), 1363 (m), 1231 (w), 1165 (s), 1148 (m), 1089 (w), 1048 (w), 766 (w), 746

(w), 664 (m), 576 (s), 543 (s).

Mp: 123-124 °C.

Methyl 3-(1-tosyl-1*H*-indazol-3-yl)propanoate Ts-**2s**

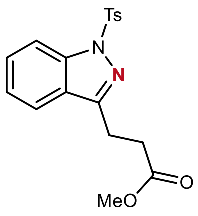

TLC: 0.64 (*n*-pentane/EtOAc = 5/2).

^{1}H NMR (300 MHz, $CDCl_3$) δ 8.20 – 8.12 (m, 1H), 7.86 – 7.76 (m, 2H), 7.64 (dt, *J* = 8.0, 1.0 Hz, 1H), 7.54 (ddd, *J* = 8.4, 7.1, 1.1 Hz, 1H), 7.31 (ddd, *J* = 8.0, 7.1, 0.9 Hz, 1H), 7.24 – 7.16 (m, 2H), 3.65 (s, 3H), 3.28 – 3.16 (m, 2H), 2.92 – 2.81 (m, 2H), 2.34 (s, 3H).

^{13}C NMR (76 MHz, $CDCl_3$) δ 173.1, 152.8, 145.2, 141.5, 134.7, 129.8, 129.4, 127.6, 125.5, 124.1, 120.5, 113.7, 51.9, 31.9, 22.3, 21.8.

HRMS (ESI) m/z: [M+Na$^+$] Calculated for $C_{18}H_{18}N_2O_4SNa$: 381.08795. Found: 381.08769.

IR (neat): 1734 (m), 1437 (w), 1373 (m), 1253 (w), 1188 (m), 1173 (s), 1124 (w), 814 (w), 752 (m), 670 (m), 567 (m), 539 (s).

Mp: 139-141 °C.

3-(1-(Methoxycarbonyl)-1*H*-indazol-3-yl)propyl 2-(3-cyano-4-isobutoxyphenyl)-4-methylthiazole-5-carboxylate Moc-**2t**

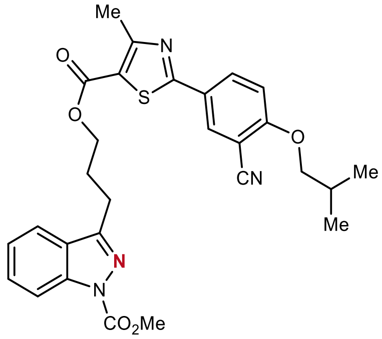

Moc-**2t** was synthesized according to the general procedure III from Moc-**1t** (53.1 mg, 0.100 mmol, 1.0 equiv.), *N*-nitrosomorpholine (35 mg, 0.30 mmol, 3.0 equiv.), TsOH•H_2O (29 mg, 0.15 mmol, 1.5 equiv.) in EtOAc (1 mL) and subsequent ring closure with PPh_3 (6.90 mg, 0.026 mmol), DEAD (12.0 µL, 0.026 mmol, 40 wt.% in toluene) in dry THF (0.5 mL). After purification by flash chromatography (*n*-pentane/EtOAc = 1/1), the desired compound Moc-**2t** was obtained as a white solid (7.5 mg, 14% yield). The yield of oxime intermediate was 13% (0.013 mmol). In this case, indole Moc-**1t** was recovered (27.1 mg, 51%).

TLC: 0.18 (*n*-pentane/EtOAc = 5/2).

^{1}H NMR (400 MHz, $CDCl_3$) δ 8.22 (d, *J* = 8.4 Hz, 1H), 8.15 (s, 1H), 8.08 (d, *J* = 8.8 Hz, 1H), 7.71 (d, *J* = 7.8 Hz,

1H), 7.60 – 7.51 (m, 1H), 7.35 (t, J = 7.6 Hz, 1H), 7.02 (d, J = 9.6 Hz, 1H), 4.61 – 4.38 (m, 2H), 4.11 (s, 3H), 3.90 (d, J = 5.2 Hz, 2H), 3.17 (t, J = 7.6 Hz, 2H), 2.74 (s, 3H), 2.34 (p, J = 6.8 Hz, 2H), 2.28 – 2.14 (m, 1H), 1.09 (d, J = 5.2 Hz, 6H).

^{13}C NMR (125 MHz, $CDCl_3$) δ 167.4, 162.7, 162.0, 161.6, 152.0, 151.3, 132.7, 132.3, 129.5, 126.1, 125.4, 124.0, 121.5, 120.3, 115.5, 114.9, 112.8, 103.2, 75.9, 64.9, 54.6, 28.3, 27.6, 24.3, 19.2, 17.6.

HRMS (ESI) m/z: [M+Na$^+$] Calculated for $C_{28}H_{28}N_4O_5SNa$: 555.16726. Found: 555.16760.

IR (neat): 1733 (s), 1716 (s), 1521 (m), 1507 (m), 1446 (s), 1372 (s), 1328 (w), 1259 (s), 1088 (m), 1013 (w), 761 (m).

Mp: 133-135 °C.

3-(1*H*-Indazol-3-yl)propyl 2-(3-cyano-4-isobutoxyphenyl)-4-methylthiazole-5-carboxylate 2t

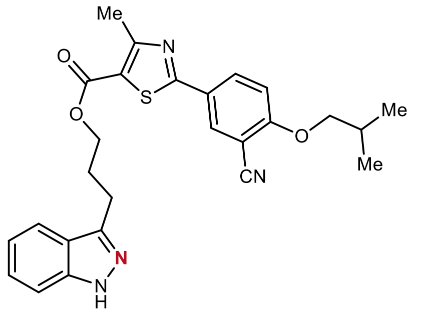

2t was prepared according to the general procedure VII using **1t** (142 mg, 0.300 mmol, 1.0 equiv.) in CH_3CN (6 mL) and $NaIO_4$ (257 mg, 1.20 mmol, 4.0 equiv.) in H_2O (6 mL) at 60 °C for 12 h. After deformylation with conc. HCl (0.04 mL) at room temperature for 24 h, 2-aminophenone was obtained after column chromatography (SiO_2, *n*-pentane/EtOAc = 10/3) as a yellow oil (87 mg, 61%, 0.183 mmol). 2-Aminophenone was used for the second step with $Me_3C_6H_2SO_2ONHBoc$ (86.5 mg, 0.275 mmol) in HFIP (1 mL). Through purification of step 2 by flash chromatography (*n*-pentane/EtOAc = 5/1), **2t** was obtained as a light white solid (65.4 mg, 46% overall yield).

TLC: 0.36 (*n*-pentane/EtOAc = 1/1).

^{1}H NMR (400 MHz, $CDCl_3$) δ 10.33 (s, 1H), 8.14 (d, J = 2.3 Hz, 1H), 8.06 (dd, J = 8.8, 2.3 Hz, 1H), 7.75 – 7.69 (m, 1H), 7.46 – 7.42 (m, 1H), 7.37 (ddd, J = 8.3, 6.8, 1.1 Hz, 1H), 7.20 – 7.13 (m, 1H), 6.99 (d, J = 8.9 Hz, 1H), 4.41 (t, J = 6.3 Hz, 2H), 3.89 (d, J = 6.5 Hz, 2H), 3.16 (t, J = 7.4 Hz, 2H), 2.74 (s, 3H), 2.38 – 2.26 (m, 2H), 2.25 – 2.14 (m, 1H), 1.08 (d, J = 6.8 Hz, 6H).

^{13}C NMR (101 MHz, $CDCl_3$) δ 167.3, 162.6, 162.1, 161.3, 146.0, 141.3, 132.7, 132.2, 126.9, 126.1, 122.2, 121.9, 120.5, 120.1, 115.5, 112.7, 110.0, 103.0, 75.8, 65.0, 28.3, 27.9, 23.7, 19.2, 17.6.

HRMS (ESI) m/z: [M+Na$^+$] Calculated for $C_{26}H_{26}N_4O_3SNa$: 497.16178. Found: 497.16183.

IR (neat): 1708 (w), 1604 (w), 1506 (w), 1431 (w), 1371 (w), 1326 (w), 1258 (s), 1099 (m), 1044 (w), 1009 (m), 820 (w), 735 (s), 703 (m).

Mp: 128-130 °C.

Methyl (*S*)-3-(1*H*-indazol-3-yl)-2-(2,2,2-trifluoroacetamido)propanoate 2u

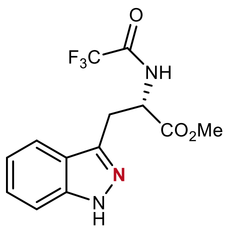

2u was prepared according to the general procedure VII using **1u** (157 mg, 0.500 mmol, 1.0 equiv.) in MeOH (10 mL) and $NaIO_4$ (428 mg, 2.00 mmol, 4.0 equiv.) in H_2O (10 mL) at r.t. for 24 h. After deformylation with conc. HCl (0.04 mL) at room temperature for 48 h, 2-aminophenone was obtained after column chromatography (SiO_2, *n*-pentane/EtOAc = 1/1) as a yellow oil (133.1 mg, 84%, 0.420 mmol). 2-Aminophenone (63.7 mg, 0.200 mmol) was used for the second step with $Me_3C_6H_2SO_2ONHBoc$ (95 mg, 0.30 mmol) in HFIP (1 mL). Through purification of step 2 by flash chromatography (*n*-pentane/EtOAc = 1/1), **2u** was obtained as a light white solid (44 mg, 58% overall yield).

TLC: 0.48 (*n*-pentane/EtOAc = 1/1).

^{1}H NMR (400 MHz, $CDCl_3$) δ 10.43 (s, 1H), 8.17 (d, *J* = 8.3 Hz, 1H), 7.68 – 7.62 (m, 1H), 7.46 – 7.36 (m, 2H), 7.21 – 7.14 (m, 1H), 5.19 – 5.07 (m, 1H), 3.78 – 3.71 (m, 1H), 3.69 (s, 3H), 3.55 – 3.48 (m, 1H).

^{13}C NMR (101 MHz, $CDCl_3$) δ 170.7, 157.3 (q, *J* = 37.7 Hz), 141.4, 141.0, 127.5, 122.2, 121.2, 119.5, 115.8 (q, *J* = 287.6 Hz), 110.3, 53.1, 51.6, 28.0.

^{19}F NMR (376 MHz, $CDCl_3$) δ -75.9.

HRMS (ESI) m/z: [M+Na$^+$] Calculated for $C_{13}H_{12}N_3O_3F_3Na$: 338.07230. Found: 338.07238.

IR (neat): 1713 (s), 1623 (w), 1550 (m), 1500 (w), 1440 (w), 1349 (w), 1211 (s), 1164 (s), 746 (m).

Mp: 128-130 °C.

Methyl ((*S*)-3-(1H-indazol-3-yl)-2-(2,2,2-trifluoroacetamido)propanoyl)-*L*-phenylalaninate 2v

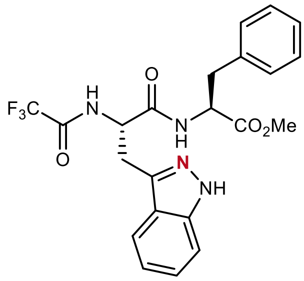

2v was prepared according to the general procedure VII using **1v** (461 mg, 1.00 mmol, 1.0 equiv.) in CH_3CN (10 mL) and $NaIO_4$ (856 mg, 4.00 mmol, 4.0 equiv.) in H_2O (10 mL) at 60 °C for 12 h for oxidative cleavage. Another portion of $NaIO_4$ (856 mg, 4.00 mmol, 4.0 equiv.) in H_2O (10 mL) was added to force complete consumption of **1v**. The reaction mixture was heating at 60 °C for another 12 h. After deformylation with conc. HCl (0.125 mL) at room temperature for 24 h, 2-aminophenone was obtained after column chromatography (SiO_2, *n*-pentane/EtOAc = 1/1) as a yellow oil (304.8 mg, 66%, 0.660 mmol). 2-Aminophenone (93 mg, 0.20 mmol) was used for the second step with $Me_3C_6H_2SO_2ONHBoc$ (95 mg, 0.30 mmol) in HFIP (1.5 mL). Through purification of step 2 by flash chromatography (*n*-pentane/EtOAc = 1/1), **2v** was obtained as a light white solid (54.7 mg, 40% overall yield).

TLC: 0.36 (*n*-pentane/EtOAc = 1/1).

^{1}H NMR (400 MHz, $CDCl_3$) δ 10.32 (s, 1H), 7.95 (d, *J* = 7.4 Hz, 1H), 7.70 (d, *J* = 8.2 Hz, 1H), 7.55 (dd, *J* = 8.2, 1.0 Hz, 1H), 7.33 – 7.25 (m, 2H), 7.16 – 7.09 (m, 3H), 7.05 – 7.00 (m, 1H), 6.96 – 6.90 (m, 2H), 5.07 – 4.95 (m, 1H), 4.77 – 4.65 (m, 1H), 3.52 (dd, *J* = 15.3, 4.9 Hz, 1H), 3.46 (s, 3H), 3.28 (dd, *J* = 15.2, 8.5 Hz, 1H), 2.92 (d, *J* = 6.1 Hz, 2H).

^{13}C NMR (101 MHz, $CDCl_3$) δ 171.8, 169.6, 157.0 (q, *J* = 37.7 Hz), 141.8, 140.8, 135.7, 129.4, 128.6, 127.3, 127.2, 122.3, 121.0, 120.0, 115.9 (q, *J* = 289.2 Hz), 110.0, 53.7, 52.5, 52.4, 38.1, 29.3.

^{19}F NMR (376 MHz, $CDCl_3$) δ -75.7.

HRMS (ESI) m/z: [M+Na$^+$] Calculated for $C_{22}H_{21}N_4O_4F_3Na$: 485.14071. Found: 485.14136.

IR (neat): 1720 (m), 1660 (s), 1547 (m), 1439 (w), 1351 (w), 1276 (w), 1211 (s), 1155 (s), 1078 (w), 1031 (w), 746 (s), 700 (m).

Mp: 177-180 °C.

Methyl ((*S*)-2-((*S*)-2-acetamidopropanamido)-3-(1*H*-indazol-3-yl)propanoyl)-*L*-valinate 2w

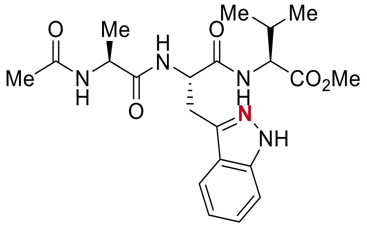

2w was prepared according to the general procedure VII using **1w** (430 mg, 1.00 mmol, 1.0 equiv.) in CH_3CN (10 mL) and $NaIO_4$ (856 mg, 4.00 mmol, 4.0 equiv.) in H_2O (10 mL) at 60 °C for 12 h for oxidative cleavage. After deformylation with conc. HCl (0.125 mL) at room temperature for 24 h, 2-aminophenone was obtained after column chromatography (SiO_2, EtOAc/ MeOH = 20/1) as a yellow oil (232.8 mg, 54%, 0.540 mmol). 2-Aminophenone (44 mg, 0.10 mmol) was used for the second step with $Me_3C_6H_2SO_2ONHBoc$ (48 mg, 0.15 mmol) in HFIP (1.0 mL). Through purification of step 2 by flash chromatography (DCM/ MeOH = 20/1) crude **2w** was obtained, which was further purified by reversed-phase MPLC (MeCN/water, v/v ratio was increased gradient from 20:80 to 65:35 over 9 min, then kept at 65:35 for 3 min, then increased from 65:35 to 90:10 over 5 min and finally kept at 90:10 for 5 min) to give **2w** as a white solid (20 mg, 25% overall yield).

TLC: 0.10 (EtOAc/MeOH = 20/1).

^{1}H NMR (400 MHz, MeOD) δ 7.76 – 7.67 (m, 1H), 7.48 – 7.43 (m, 1H), 7.37 – 7.32 (m, 1H), 7.13 – 7.08 (m, 1H), 4.30 – 4.21 (m, 2H), 4.14 (d, *J* = 6.3 Hz, 1H), 3.60 (s, 3H), 3.49 (dd, *J* = 14.7, 6.7 Hz, 1H), 3.39 (dd, *J* = 14.7, 6.2 Hz, 1H), 2.02 – 1.98 (m, 1H), 1.98 (s, 3H), 1.28 (d, *J* = 7.2 Hz, 3H), 0.74 (dd, *J* = 9.9, 6.8 Hz, 6H).

^{13}C NMR (101 MHz, MeOD) δ 173.4, 172.1, 171.8, 171.6, 141.5, 141.2, 126.4, 122.0, 120.1, 119.7, 109.7, 58.0, 52.8, 51.0, 49.5, 30.4, 28.2, 21.1, 17.8, 17.0, 16.1.

HRMS (ESI) m/z: [M+Na$^+$] Calculated for $C_{21}H_{29}N_5O_5Na$: 454.20609. Found: 454.20684

IR (neat): 1740 (w), 1642 (s), 1530 (m), 1435 (w), 1372 (w), 1209 (w), 1153 (w), 1005 (w), 744 (m).

Mp: 180-181 °C.

(3*S*,8a*S*)-3-((1*H*-Indazol-3-yl)methyl)hexahydropyrrolo[1,2-*a*]pyrazine-1,4-dione 2x

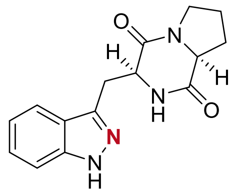

2x was prepared according to the general procedure VII using **1x** (142 mg, 0.500 mmol, 1.0 equiv.) in MeOH (10 mL) and $NaIO_4$ (428 mg, 2.00 mmol, 4.0 equiv.) in H_2O (10 mL) at r.t. for 24 h for oxidative cleavage. After deformylation with conc. HCl (0.07 mL) at 70 °C for 1 h, 2-aminophenone was obtained after column chromatography (SiO_2, EtOAc/ MeOH = 100/3) as a yellow oil (83.5 mg, 59%, 0.290 mmol). 2-Aminophenone (83.5 mg, 0.290 mmol) was used for the second step with $Me_3C_6H_2SO_2ONHBoc$ (137 mg, 0.435 mmol) in HFIP (1.5 mL). Through purification of step 2 by flash chromatography (EtOAc/ MeOH = 100/3), **2x** was obtained as a white solid (46.2 mg, 33% overall yield).

TLC: 0.16 (EtOAc/MeOH = 20/1).

^{1}H NMR (400 MHz, DMSO) δ 12.82 (s, 1H), 7.84 (s, 1H), 7.77 (d, *J* = 8.2 Hz, 1H), 7.47 (d, *J* = 8.4 Hz, 1H), 7.38 – 7.29 (m, 1H), 7.16 – 7.04 (m, 1H), 4.59 (t, *J* = 6.1 Hz, 1H), 4.19 (t, *J* = 8.0 Hz, 1H), 3.56 (dd, *J* = 15.6, 4.9 Hz, 1H), 3.47 – 3.37 (m, 1H), 3.33 – 3.26 (m, 1H), 3.18 (dd, *J* = 15.6, 7.0 Hz, 1H), 2.13 – 2.04 (m, 1H), 1.80 – 1.62 (m, 3H).

^{13}C NMR (101 MHz, DMSO) δ 169.2, 165.3, 141.5, 140.7, 126.1, 121.9, 120.2, 119.6, 109.9, 58.5, 53.7, 44.9, 27.7, 27.0, 22.1.

HRMS (ESI) m/z: [M+Na$^+$] Calculated for $C_{15}H_{16}N_4O_2Na$: 307.11655. Found: 307.11652.

IR (neat): 3245 (w), 2954 (w), 1658 (s), 1424 (m), 1303 (w), 1264 (w), 1060 (w), 732 (s), 699 (m).

Mp: 219-220 °C.

(3*S*,8a*S*)-3-((1-Tosyl-1*H*-benzo[*d*]imidazol-2-yl)methyl)hexahydropyrrolo[1,2-*a*]pyrazine-1,4-dione Ts-3x

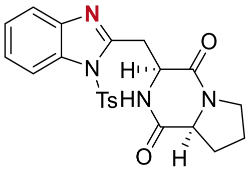

Ts-3x was prepared according to the general procedure VIII using **1x** (142 mg, 0.500 mmol, 1.0 equiv.) in MeOH (10 mL) and $NaIO_4$ (428 mg, 2.00 mmol, 4.0 equiv.) in H_2O (10 mL) at r.t. for 24 h for oxidative cleavage. After deformylation with conc. HCl (0.07 mL) at 70 °C for 1 h, 2-aminophenone was obtained after column chromatography (SiO_2, EtOAc/ MeOH = 100/3) as a yellow oil (83.5 mg, 59%, 0.290 mmol). 2-Aminophenone (57.5 mg, 0.200 mmol) was used for the second step through oxime as the intermediate. After purification of step 2 by flash chromatography (EtOAc/ MeOH = 100/3) crude **Ts-3x** and **Ts-2x** were obtained, which were further purified by reversed-phase MPLC (MeCN/water, v/v ratio was increased gradient from 20:80 to 65:35 over 9 min, then kept at 65:35 for 3 min, then increased from 65:35 to 90:10 over 5 min and finally kept at 90:10 for 5 min) to give **Ts-3x** as a colourless oil (28 mg, 26% overall yield) as well as **Ts-2x** as a white solid (8.8 mg, 10% overall yield).

TLC: 0.26 (EtOAc/MeOH = 20/1).

^{1}H NMR (400 MHz, $CDCl_3$) δ 8.10 – 8.03 (m, 1H), 7.93 – 7.82 (m, 2H), 7.71 – 7.58 (m, 1H), 7.41 – 7.32 (m, 2H),

7.31 (d, J = 8.2 Hz, 2H), 7.13 (s, 1H), 4.74 – 4.61 (m, 1H), 4.26 (dd, J = 17.7, 2.6 Hz, 1H), 4.18 – 4.10 (m, 1H), 3.70 (d, J = 8.0 Hz, 1H), 3.63 – 3.55 (m, 1H), 3.26 (dd, J = 17.7, 11.0 Hz, 1H), 2.46 – 2.33 (m, 4H), 2.17 – 2.04 (m, 2H), 2.00 – 1.85 (m, 1H).

^{13}C NMR (101 MHz, $CDCl_3$) δ 169.4, 164.4, 151.1, 146.6, 141.6, 135.3, 133.3, 130.7, 127.2, 125.6, 125.2, 120.2, 113.8, 59.4, 53.6, 45.9, 31.6, 28.8, 22.7, 21.9.

HRMS (ESI) m/z: [M+Na$^+$] Calculated for $C_{22}H_{22}N_4O_4SNa$: 461.12540. Found: 461.12499.

IR (neat): 1667 (s), 1451 (w), 1373 (m), 1170 (m), 1089 (w), 1052 (w), 744 (w), 675 (m), 578 (s), 543 (m).

(3*S*,8a*S*)-3-((1-Tosyl-1*H*-indazol-3-yl)methyl)hexahydropyrrolo[1,2-*a*]pyrazine-1,4-dione Ts-2x

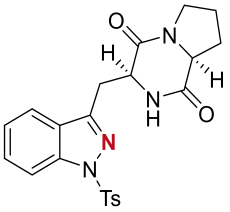

TLC: 0.20 (EtOAc/MeOH = 20/1).

^{1}H NMR (400 MHz, $CDCl_3$) δ 8.18 (d, J = 8.5 Hz, 1H), 7.89 – 7.81 (m, 2H), 7.70 – 7.65 (m, 1H), 7.61 – 7.53 (m, 1H), 7.35 (dd, J = 8.1, 7.0 Hz, 1H), 7.26 (d, J = 8.2 Hz, 2H), 6.43 (s, 1H), 4.56 (dt, J = 10.6, 2.5 Hz, 1H), 4.13 (dd, J = 9.3, 6.7 Hz, 1H), 3.87 (dd, J = 16.5, 3.1 Hz, 1H), 3.71 – 3.51 (m, 2H), 3.15 (dd, J = 16.4, 10.5 Hz, 1H), 2.42 – 2.36 (m, 1H), 2.35 (s, 3H), 2.14 – 2.00 (m, 2H), 1.97 – 1.87 (m, 1H).

^{13}C NMR (101 MHz, $CDCl_3$) δ 169.2, 164.7, 150.2, 145.8, 141.5, 134.4, 130.3, 130.0, 127.6, 125.3, 124.5, 120.5, 113.8, 59.3, 53.5, 45.8, 28.6, 28.0, 22.7, 21.8.

HRMS (ESI) m/z: [M+Na$^+$] Calculated for $C_{22}H_{22}N_4O_4SNa$: 461.12540. Found: 461.12543.

IR (neat): 1667 (s), 1433 (w), 1375 (m), 1189 (w), 1175 (m), 749 (w), 671 (m), 575 (m), 539 (m).

Mp: 203-204 °C.

Ethyl 3-(2-(2,2,2-trifluoroacetamido)ethyl)-1*H*-indazole-1-carboxylate Eoc-**2y**

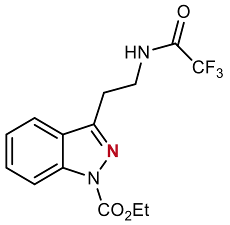

Eoc-**2y** was synthesized according to the general procedure III from Eoc-**1y** (98.5 mg, 0.300 mmol, 1.0 equiv.), *N*-nitrosomorpholine (105 mg, 0.900 mmol, 3.0 equiv.), $TsOH•H_2O$ (86 mg, 0.45 mmol, 1.5 equiv.) in EtOAc (3 mL) and subsequent ring closure with PPh_3 (63 mg, 0.24 mmol), DEAD (0.11 mL, 0.24 mmol, 40 wt.% in toluene) in dry THF (1 mL). After purification by flash chromatography and prepare TLC plates (*n*-pentane/EtOAc = 10/3), the desired compound Eoc-**2y** was obtained as a white solid (23.7 mg, 24% yield). The yield of oxime intermediate was determined by ^{1}H NMR with dibromomethane as internal standard (40%, 0.12 mmol). In this case, indole Eoc-**1y** was recovered (24.2 mg, 25%).

TLC: 0.40 (*n*-pentane/EtOAc = 5/2).

^{1}H NMR (400 MHz, $CDCl_3$) δ 8.22 (d, *J* = 8.5 Hz, 1H), 7.71 – 7.66 (m, 1H), 7.64 – 7.55 (m, 2H), 7.42 – 7.34 (m, 1H), 4.58 (q, *J* = 7.1 Hz, 1H), 3.91 (q, *J* = 6.2 Hz, 2H), 3.32 – 3.17 (m, 2H), 1.51 (t, *J* = 7.1 Hz, 3H).

^{13}C NMR (101 MHz, $CDCl_3$) δ 157.4 (q, *J* = 37.1 Hz), 150.7, 149.7, 140.6, 129.9, 125.2, 124.2, 120.1, 116.0 (q, *J* = 285.4 Hz), 114.9, 64.1, 37.7, 25.9, 14.5.

^{19}F NMR (282 MHz, $CDCl_3$) δ -76.1.

HRMS (ESI) m/z: [M+Na$^+$] Calculated for $C_{14}H_{14}N_3O_3F_3Na$: 352.08795. Found: 352.08756.

IR (neat): 1716 (s), 1558 (w), 1438 (w), 1417 (w), 1379 (w), 1339 (w), 1305 (w), 1209 (s), 1155 (s), 1087 (m), 758 (m).

Mp: 94-96 °C.

Ethyl 2-(2-(2,2,2-trifluoroacetamido)ethyl)-1*H*-benzo[*d*]imidazole-1-carboxylate Eoc-**3y**

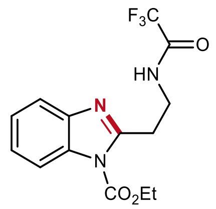

Eoc-**3y** was synthesized according to the general procedure II from Eoc-**1y** (98.5 mg, 0.300 mmol, 1.0 equiv.), *N*-nitrosomorpholine (105 mg, 0.900 mmol, 3.0 equiv.), TsOH•H_2O (86 mg, 0.45 mmol, 1.5 equiv.) in EtOAc (3 mL) and subsequent ring closure with Et_3N (25 µL, 0.18 mmol), 2,6-lutidine (19 µL, 0.18 mmol) in dry DCE (1 mL) as well as MsCl (11.5 µL, 0.144 mmol) in dry DCE (1 mL). After purification by flash chromatography (*n*-pentane/EtOAc = 20/1), the desired compound Eoc-**3y** was obtained as a white solid (24.0 mg, 25% yield). In this case, indole Eoc-**1y** was recovered (23.1 mg, 24%). The yield of oxime intermediate was determined by ^{1}H NMR with dibromomethane as internal standard (39%, 0.12 mmol).

TLC: 0.44 (*n*-pentane/EtOAc = 5/2).

^{1}H NMR (400 MHz, $CDCl_3$) δ 8.03 – 7.90 (m, 2H), 7.73 – 7.64 (m, 1H), 7.40 – 7.32 (m, 2H), 4.57 (q, *J* = 7.2 Hz, 2H), 3.98 (q, *J* = 5.7 Hz, 2H), 3.44 (t, *J* = 5.7 Hz, 2H), 1.53 (t, *J* = 7.2 Hz, 3H).

^{13}C NMR (76 MHz, $CDCl_3$) δ 157.2 (q, *J* = 37.2 Hz), 154.0, 150.4, 141.9, 132.8, 125.2, 124.8, 119.7, 116.0 (q, *J* = 286.0 Hz), 115.2, 64.6, 36.4, 30.7, 14.4.

^{19}F NMR (282 MHz, $CDCl_3$) δ -76.2.

HRMS (ESI) m/z: [M+Na$^+$] Calculated for $C_{14}H_{14}N_3O_3F_3Na$: 352.08795. Found: 352.08757.

IR (neat): 1748 (m), 1716 (s), 1541 (w), 1456 (m), 1375 (m), 1323 (m), 1258 (w), 1152 (s), 1094 (m), 1011 (m), 763 (m), 749 (m).

Mp: 135-136 °C.

N-(2-(1H-Indazol-3-yl)ethyl)-2,2,2-trifluoroacetamide 2y

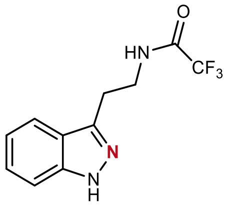

2y was prepared according to the general procedure VII using **1y** (513 mg, 2.00 mmol, 1.0 equiv.) in MeOH (40 mL) and $NaIO_4$ (1.71 g, 8.00 mmol, 4.0 equiv.) in H_2O (40 mL) at room temperature for 24 h. After deformylation with conc. HCl (0.25 mL) at 70 °C for 1 h, 2-aminophenone was obtained after column chromatography (SiO_2, *n*-pentane/DCM = 1/1) as a yellow solid (368 mg, 72%). 2-Aminophenone (0.20 mmol) was conducted for the second step with $Me_3C_6H_2SO_2ONHBoc$ (95 mg, 0.30 mmol) in HFIP (1 mL). Through purification of step 2 by flash chromatography (*n*-pentane/EtOAc = 1/1), **2y** was obtained as a light white solid (43 mg, 60% overall yield).

TLC: 0.20 (*n*-pentane/EtOAc = 5/2).

^{1}H NMR (400 MHz, CD_3CN) δ 11.01 (s, 1H), 7.80 (s, 1H), 7.72 (dt, J = 8.1, 1.0 Hz, 1H), 7.51 (dt, J = 8.5, 0.9 Hz, 1H), 7.37 (ddd, J = 8.4, 6.9, 1.0 Hz, 1H), 7.14 (ddd, J = 8.0, 6.9, 0.9 Hz, 1H), 3.70 (q, J = 6.6 Hz, 2H), 3.20 (t, J = 6.9 Hz, 2H).

^{13}C NMR (101 MHz, CD_3CN) δ 157.7 (q, J = 36.3 Hz), 144.2, 142.2, 127.4, 123.0, 121.2, 120.7, 117.1 (q, J = 287.2 Hz), 111.1, 39.7, 26.7.

^{19}F NMR (376 MHz, CD_3CN) δ -76.8.

HRMS (ESI) m/z: [M+Na$^+$] Calculated for $C_{11}H_{10}N_3OF_3Na$: 280.06682. Found: 280.06682.

IR (neat): 1702 (s), 1685 (w), 1558 (w), 1541 (w), 1506 (w), 1210 (s), 1160 (s), 745 (w).

Mp: 124-125 °C.

2,2,2-Trifluoro-N-(2-(1-tosyl-1H-benzo[*d*]imidazol-2-yl)ethyl)acetamide Ts-**3y**

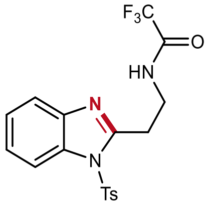

Ts-3y was prepared according to the general procedure VIII using **1y** (513 mg, 2.00 mmol, 1.0 equiv.) in MeOH (40 mL) and $NaIO_4$ (1.71 g, 8.00 mmol, 4.0 equiv.) in H_2O (40 mL) at room temperature for 24 h. After deformylation with conc. HCl (0.25 mL) at 70 °C for 1 h, 2-aminophenone was obtained after column chromatography (SiO_2, *n*-pentane/EtOAc = 10/3) as a yellow solid (368 mg, 72%). 2-Aminophenone (0.2 mmol) was used for the second step *via* oxime as intermediate. Through purification by flash chromatography (DCM/EtOAc = 20/1), Ts-**3y** and Ts-**2y** were obtained as white solids (38.4 mg, 34% overall yield, Ts-**2y**:Ts-**3y** = 1:4). The product ratio was determined by ^{1}H NMR spectroscopy.

TLC: 0.50 (*n*-pentane/EtOAc = 5/2).

^{1}H NMR (400 MHz, $CDCl_3$) δ 8.08 – 8.03 (m, 1H), 7.85 (s, 1H), 7.83 – 7.77 (m, 2H), 7.66 (dd, J = 8.0, 1.5 Hz, 1H),

7.43 – 7.34 (m, 2H), 7.31 (d, J = 8.3 Hz, 2H), 3.95 (q, J = 5.9 Hz, 2H), 3.46 – 3.35 (m, 2H), 2.40 (s, 3H).

^{13}C NMR (101 MHz, $CDCl_3$) δ 157.2 (q, J = 36.9 Hz), 152.3, 146.6, 141.5, 135.2, 133.1, 130.6, 127.0, 125.5, 125.1, 120.0, 116.0 (q, J = 287.8 Hz), 113.7, 36.5, 29.3, 21.8.

^{19}F NMR (376 MHz, $CDCl_3$) δ -76.2.

HRMS (ESI) m/z: [M+Na$^+$] Calculated for $C_{18}H_{16}N_3O_3SF_3Na$: 434.07567. Found: 434.07553.

IR (neat): 1716 (m), 1558 (w), 1541 (w), 1455 (w), 1375 (w), 1205 (m), 1169 (s), 1122 (w), 1090 (w), 1055 (w), 746 (w), 576 (m), 543 (m).

Mp: 155-157 °C.

2,2,2-Trifluoro-*N*-(2-(1-tosyl-1*H*-indazol-3-yl)ethyl)acetamide Ts-**2y**

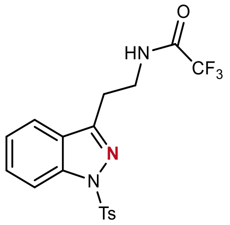

TLC: 0.40 (*n*-pentane/EtOAc = 10/7).

^{1}H NMR (400 MHz, $CDCl_3$) δ 8.25 – 8.17 (m, 1H), 7.85 – 7.79 (m, 2H), 7.62 – 7.54 (m, 2H), 7.35 (td, J = 7.6, 2.1 Hz, 1H), 7.25 – 7.20 (m, 3H), 3.87 (q, J = 6.1 Hz, 2H), 3.16 (t, J = 6.1 Hz, 2H), 2.36 (s, 3H).

^{13}C{^{19}F} NMR (126 MHz, $CDCl_3$) δ 157.3, 151.2, 145.7, 141.3, 134.6, 130.1, 130.0, 127.5, 125.2, 124.4, 120.3, 115.9, 113.7, 37.2, 26.1, 21.8.

^{19}F NMR (470 MHz, cdcl$_3$) δ -76.0.

HRMS (ESI) m/z: [M+Na$^+$] Calculated for $C_{18}H_{16}N_3O_3SF_3Na$: 434.07567. Found: 434.07553.

IR (neat): 1716 (m), 1558 (w), 1541 (w), 1375 (w), 1210 (w), 1174 (s), 1126 (w), 751 (w).

Mp: 137-139 °C.

3-(1*H*-Indazol-3-yl)-1-morpholinopropan-1-one 2z

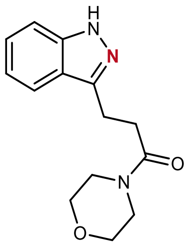

2z was prepared according to the general procedure VII using **1z** (516 mg, 2.00 mmol, 1.0 equiv.) in MeOH (40 mL) and $NaIO_4$ (1.71 g, 8.00 mmol, 4.0 equiv.) in H_2O (40 mL) at room temperature for 24 h. After deformylation with conc. HCl (0.25 mL) at 70 °C for 1 h, 2-aminophenone was obtained after column chromatography (SiO_2, *n*-pentane/DCM = 1/1) as a brown oil (432 mg, 83%). 2-Aminophenone (0.20 mmol) was used for the second step with

$Me_3C_6H_2SO_2ONHBoc$ (95 mg, 0.30 mmol) in HFIP (1 mL). Through purification of step 2 by flash chromatography (EtOAc/MeOH = 100/3), **2z** was obtained as a brown oil (39.5 mg, 67% over two steps).

TLC: 0.30 (EtOAc/MeOH = 20/1).

^{1}H NMR (400 MHz, $CDCl_3$) δ 7.71 (d, *J* = 8.1 Hz, 1H), 7.42 (d, *J* = 8.4 Hz, 1H), 7.35 (t, *J* = 7.6 Hz, 1H), 7.13 (t, *J* = 7.4 Hz, 1H), 3.59 (q, *J* = 3.5 Hz, 4H), 3.49 (t, *J* = 4.8 Hz, 2H), 3.37 (dd, *J* = 9.2, 5.9 Hz, 4H), 2.88 (dd, *J* = 8.8, 6.6 Hz, 2H).

^{13}C NMR (76 MHz, $CDCl_3$) δ 171.1, 145.9, 141.3, 127.0, 122.1, 120.5, 120.2, 110.0, 66.9, 66.5, 45.9, 42.1, 31.8, 22.5.

HRMS (ESI) m/z: [M+Na$^+$] Calculated for $C_{14}H_{17}N_3O_2Na$: 282.12130. Found: 282.12115.

IR (neat): 1653 (m), 1635 (m), 1617 (m), 1558 (w), 1541 (w), 1506 (w), 1472 (w), 1457 (w), 1437 (w), 1271 (w), 1114 (m), 1023 (w), 797 (w), 748 (w).

1-Morpholino-3-(1-tosyl-1*H*-benzo[*d*]imidazol-2-yl)propan-1-one Ts-**3z**

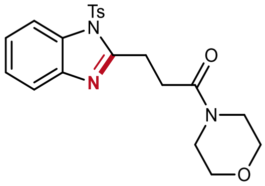

Ts-3z was prepared according to the general procedure VIII using **1z** (513 mg, 2.00 mmol, 1.0 equiv.) in MeOH (40 mL) and $NaIO_4$ (1.71 g, 8.00 mmol, 4.0 equiv.) in H_2O (40 mL) at room temperature for 24 h. After deformylation with conc. HCl (0.25 mL) at 70 °C for 1 h, 2-aminophenone was obtained after column chromatography (SiO_2, *n*-pentane/EtOAc = 10/3) as a brown oil (368 mg, 72%). 2-Aminophenone (0.20 mmol) was conducted for the second step *via* oxime as intermediate. Through purification by flash chromatography (DCM/Acetone = 20/1), Ts-**3z** and Ts-**2z** were obtained as a colourless oil (35.4 mg, 36% over two steps, Ts-**2z**:Ts-**3z** = 1:4). The product ratio was determined by ^{1}H NMR spectroscopy.

TLC: 0.50 (EtOAc/MeOH = 20/1).

^{1}H NMR (400 MHz, $CDCl_3$) δ 8.04 – 7.97 (m, 1H), 7.94 – 7.86 (m, 2H), 7.63 – 7.57 (m, 1H), 7.38 – 7.27 (m, 4H), 3.75 – 3.51 (m, 10H), 3.02 – 2.93 (m, 2H), 2.37 (s, 3H).

^{13}C NMR (101 MHz, $CDCl_3$) δ 170.2, 154.2, 146.1, 142.0, 135.4, 133.4, 130.4, 127.1, 124.9, 124.7, 119.9, 113.6, 67.0, 66.8, 46.0, 42.3, 30.1, 25.6, 21.8.

HRMS (ESI) m/z: [M+Na$^+$] Calculated for $C_{21}H_{23}N_3O_4SNa$: 436.13015. Found: 436.12989.

IR (neat): 1646 (m), 1454 (w), 1437 (w), 1366 (m), 1234 (w), 1166 (s), 1115 (s), 1089 (m), 745 (m), 665 (m), 576 (s), 543 (s).

Mp: 131-132 °C.

2-((3-Isopropyl-1*H*-indazol-4-yl)oxy)-*N*-(pyridin-2-ylmethyl)acetamide 2aa

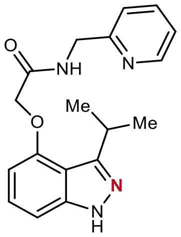

2aa was prepared according to the general procedure VII using **1aa** (97 mg, 0.30 mmol, 1.0 equiv.) in MeOH (6 mL) and $NaIO_4$ (257 g, 1.20 mmol, 4.0 equiv.) in H_2O (6 mL) at room temperature for 24 h. After deformylation with conc. HCl (38 μL) at 70 °C for 1 h, 2-aminophenone was obtained after column chromatography (SiO_2, *n*-pentane/DCM = 1/1) as a brown oil (74.6 mg, 76%, 0.228 mmol). 2-Aminophenone (0.228 mmol) was used for the second step with $Me_3C_6H_2SO_2ONHBoc$ (108 mg, 0.342 mmol) in HFIP (1 mL). Through purification of step 2 by flash chromatography (EtOAc/MeOH = 100/3), **2aa** was obtained as a brown solid (34.1 mg, 36% overall yield).

TLC: 0.50 (EtOAc/MeOH = 20/1).

^{1}H NMR (400 MHz, MeOD) δ 8.49 (ddd, *J* = 4.9, 1.8, 0.9 Hz, 1H), 7.78 (td, *J* = 7.7, 1.8 Hz, 1H), 7.37 (dd, *J* = 7.8, 1.0 Hz, 1H), 7.31 (ddd, *J* = 7.7, 4.9, 1.1 Hz, 1H), 7.24 (dd, *J* = 8.4, 7.6 Hz, 1H), 7.07 (d, *J* = 8.3 Hz, 1H), 6.48 (d, *J* = 7.6 Hz, 1H), 4.78 (s, 2H), 4.61 (s, 2H), 3.82 – 3.67 (m, 1H), 1.37 (d, *J* = 6.9 Hz, 6H).

^{13}C NMR (76 MHz, MeOD) δ 170.9, 158.3, 154.0, 152.8, 149.9, 144.9, 138.8, 128.9, 123.9, 123.1, 113.4, 104.9, 101.5, 68.5, 45.0, 29.3, 22.8.

HRMS (ESI) m/z: [M+H$^+$] Calculated for $C_{18}H_{20}N_4O_2H$: 325.16590. Found: 325.16579.

IR (neat): 1653 (s), 1558 (m), 1540 (m), 1437 (m), 1254 (w), 1119 (w), 1045 (w), 784 (w), 742 (s).

Mp: 158-159 °C.

5-(1*H*-Indazol-3-yl)-2-methyloxazole 2ab

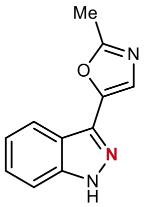

2ab was prepared according to the general procedure VII using **1ab** (60 mg, 0.30 mmol, 1.0 equiv.) in MeOH (6 mL) and $NaIO_4$ (257 g, 1.20 mmol, 4.0 equiv.) in H_2O (6 mL) at room temperature for 12 h. Another portion of $NaIO_4$ (257 g, 1.20 mmol, 4.0 equiv.) in H_2O (6 mL) was added to force complete consumption of **1ab**. The reaction mixture was stirred at r.t. for another 12 h. After deformylation with conc. HCl (38 μL) at r.t. for 24 h, 2-aminophenone was obtained after column chromatography (SiO_2, *n*-pentane/EtOAc = 1/1) as a brown oil (34.0 mg, 57%, 0.171 mmol). This step was repeated for another time to afford more 2-aminophenone. 2-Aminophenone (52.2 mg, 0.260 mmol) was used for the second step with $Me_3C_6H_2SO_2ONHBoc$ (123 mg, 0.390 mmol) in HFIP (1.5 mL). Through purification of step 2 by flash chromatography (*n*-pentane/EtOAc = 1/2), **2ab** was obtained as a brown solid (33.4 mg, 38% overall yield).

TLC: 0.60 (EtOAc/MeOH = 20/1).

^{1}H NMR (400 MHz, MeOD) δ 8.01 (dd, *J* = 8.2, 1.0 Hz, 1H), 7.58 – 7.54 (m, 1H), 7.51 (s, 1H), 7.48 – 7.40 (m, 1H), 7.29 – 7.19 (m, 1H), 2.58 (s, 3H).

^{13}C NMR (101 MHz, MeOD) δ 163.1, 147.8, 142.6, 135.2, 128.4, 123.7, 122.9, 121.5, 121.0, 111.5, 13.6.

HRMS (ESI) m/z: [M+H$^+$] Calculated for $C_{11}H_9N_3ONa$: 222.06378. Found: 222.06373.

IR (neat): 1689 (w), 1623 (w), 1570 (w), 1509 (w), 1346 (w), 1259 (w), 1031 (s), 938 (m), 799 (s), 741 (s), 695 (s), 456 (m).

Mp: 184-185 °C.

Methyl 2-(10-ethyl-2-methyl-1-oxo-1,2,4,5-tetrahydrobenzo[4,5]imidazo[1,2-*d*][1,4]oxazepin-2-yl)acetate 3ac

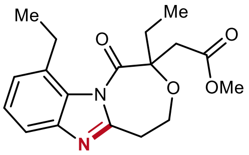

Under air, a solution of indole **3ac** (121 mg, 0.400 mmol, 1.0 equiv.) in MeOH (2 mL) was added dropwise to a solution of sodium metaperiodate $NaIO_4$ (214 mg, 1.00 mmol, 2.5 equiv.) in water (1 mL) at 0 °C using an ice-water bath. Then, the reaction mixture was allowed to warmed to r.t. and was stirred for 4 h. After consumption of the starting material (as monitored by TLC), the mixture was poured into water and extracted with DCM (3 times). The combined organic phase was washed with brine and dried over anhydrous Na_2SO_4. After filtration, the solvent was removed with a rotary evaporator under reduced pressure and the residue was dissolved in dry CH_3CN (2 mL) under argon. Then, the reaction mixture was cooled to 0 °C using an ice/water bath, $TMSN_3$ (66 μL, 0.50 mmol, 1.25 equiv.) and TfOH (53 μL, 0.6 mmol, 1.5 equiv.) were added subsequently. Afterwards, the reaction mixture was stirred at room temperature for 4 h. After completion, the solvent was removed with a rotary evaporator under reduced pressure and the residue was purified by column chromatography (SiO_2, Pentane/EtOAc = 5/1) to give **3ac** as a yellow solid (60.4 mg, 46% yield).

TLC: 0.26 (Pentane/EtOAc = 1/1).

^{1}H NMR (400 MHz, $CDCl_3$) δ 7.52 (d, *J* = 8.0 Hz, 1H), 7.29 (t, *J* = 8.0 Hz, 1H), 7.19 (d, *J* = 7.6 Hz, 1H), 4.32 – 4.06 (m, 2H), 3.60 (s, 3H), 3.42 (t, *J* = 6.4 Hz, 2H), 3.04 (q, *J* = 12.0 Hz, 2H), 2.75 (qd, *J* = 7.6, 2.8 Hz, 2H), 2.17 (q, *J* = 7.6 Hz, 2H), 1.31 (t, *J* = 7.6 Hz, 3H), 1.10 (t, *J* = 7.6 Hz, 3H).

^{13}C NMR (100 MHz, $CDCl_3$) δ 178.0, 169.7, 150.7, 144.0, 132.7, 130.7, 125.4, 124.9, 117.2, 83.3, 61.1, 52.1, 41.0, 30.4, 29.6, 25.1, 14.5, 8.0.

HRMS m/z: $[M+Na]^+$ Calculated for $C_{18}H_{22}N_2O_4Na$: 353.14718. Found: 353.14680.

IR (neat): 1723 (s), 1555 (w), 1436 (w), 1309 (m), 1267 (m), 1203 (s), 1143 (m), 1094 (w), 800 (w), 745 (m).

Mp: 80-81 °C.

2-((Pyridin-4-ylthio)methyl)benzo[*d*]oxazole 6p

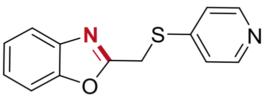

6p was prepared according to the general procedure V using **4p** (24.1 mg, 0.100 mmol, 1.0 equiv.), *N*-

nitrosomorpholine (35.0 mg, 0.300 mmol, 3.0 equiv.), TsOH•H_2O (28.6 mg, 0.150 mmol, 1.5 equiv.) in EtOAc/DCM (3 mL, 1:2) and and subsequent ring closure with Ms_2O (26.2 mg, 0.150 mmol, 1.5 equiv.). After purification by flash chromatography (DCM/MeOH = 50/1), crude **6p** was obtained, which was further purified by reversed-phase MPLC (MeCN/water, v/v ratio was increased gradient from 20:80 to 65:35 over 9 min, then kept at 65:35 for 3 min, then increased from 65:35 to 90:10 over 2 min and finally kept at 90:10 for 7 min) to give **6p** as a colourless oil (14.6 mg, 60%).

TLC: 0.18 (Pentane/EtOAc = 1/1).

^{1}H NMR (400 MHz, $CDCl_3$) δ 8.44 (d, *J* = 5.3 Hz, 2H), 7.74 – 7.68 (m, 1H), 7.54 – 7.47 (m, 1H), 7.39 – 7.28 (m, 4H), 4.42 (s, 2H).

^{13}C NMR (101 MHz, $CDCl_3$) δ 161.9, 151.2, 149.8, 147.0, 141.1, 125.7, 124.9, 121.1, 120.4, 110.9, 28.5.

HRMS (ESI) m/z: [M+Na^+] Calculated for $C_{13}H_{10}N_2OSNa$: 265.04060. Found: 265.04081.

IR (neat): 1573 (s), 1483 (w), 1454 (m), 1409 (w), 1242 (w), 949 (w), 839 (w), 800 (w), 745 (m), 702 (w), 495 (w).

7,10-Dimethyl-1,2,3,4-tetrahydro-5*H*-benzo[3,4]chromeno[6,7-*d*]isoxazol-5-one 5q

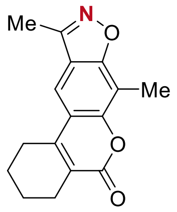

5q was prepared according to the general procedure V using **4q** (53.6 mg, 0.200 mmol, 1.0 equiv.), *N*-nitrosomorpholine (69.8 mg, 0.300 mmol, 3.0 equiv.), camphor-10-sulfonic acid (CSA, 70 mg, 0.30 mmol, 1.5 equiv.) in dry DCE (2 mL) and the reaction was irradiated at 467 nm (45 W) for 3 h. *Afterwards, one-pot process was conducted because of the poor solubility of the oxime intermediate.* After completed irradiation, Et_3N (55.6 µL, 0.400 mmol, 2.0 equiv.) was added and the reaction mixture was stirred at room temperature for 10 min. Then, the reaction mixture was cooled down to 0 °C using an ice/water bath, MsCl (17 µL, 0.22 mmol, 1.1 equiv.) in dry DCE (1 mL) was added dropwise slowly. The mixture was stirred at 0 °C for 30 min. The solvent was removed and after purification by flash chromatography (*n*-pentane/EtOAc = 10/3), **5q** was obtained as a white solid (26.0 mg, 48%) along with **6q** that was isolated as a white solid (9.2 mg, 17%).

TLC: 0.26 (Pentane/EtOAc = 10/3).

^{1}H NMR (400 MHz, $CDCl_3$) δ 7.62 (s, 1H), 2.88 – 2.79 (m, 2H), 2.64 – 2.54 (m, 8H), 1.95 – 1.88 (m, 2H), 1.86 – 1.79 (m, 2H).

^{13}C NMR (101 MHz, $CDCl_3$) δ 162.3, 161.6, 155.8, 151.3, 147.2, 122.6, 118.5, 117.9, 112.8, 107.9, 25.8, 24.2, 21.7, 21.6, 10.4, 9.0.

HRMS (ESI) m/z: [M+Na^+] Calculated for $C_{16}H_{15}NO_3Na$: 292.09441. Found: 292.09426.

IR (neat): 1707 (s), 1620 (w), 1602 (w), 1119 (m), 1018 (w), 811 (w), 762 (w), 735 (m), 703 (w), 627 (w), 524 (w).

Mp: 214-216 °C.

7,9-Dimethyl-1,2,3,4-tetrahydro-5*H*-benzo[3,4]chromeno[6,7-*d*]oxazol-5-one 6q

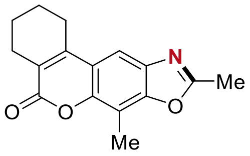

6q was prepared according to the general procedure V using **4q** (53.6 mg, 0.200 mmol, 1.0 equiv.), *N*-nitrosomorpholine (69.8 mg, 0.300 mmol, 3.0 equiv.), camphor-10-sulfonic acid (CSA, 70 mg, 0.30 mmol, 1.5 equiv.) in dry DCE (2 mL) and the reaction was irradiated at 467 nm (45 W) for 3 h. *Afterwards, one-pot process was conducted because of the poor solubility of the oxime intermediate.* After completed irradiation, the reaction mixture was cooled down to 0 °C using an ice/water bath, Ms_2O (52.3 mg, 0.300 mmol, 1.5 equiv.) in dry DCE (1 mL) was added dropwise slowly. The mixture was heated to 70 °C for 1 h. Then sat. $NaHCO_3$ aqueous solution was added and the mixture was extracted with DCM (3 times). The organic phase was washed with brine and dried over Na_2SO_4. After filtration, the solvent was removed with a rotary evaporator under reduced pressure and the residue was purified by flash chromatography (*n*-pentane/EtOAc = 10/3), **6q** was obtained as a white solid (40.9 mg, 76%).

TLC: 0.26 (Pentane/EtOAc = 10/3).

^{1}H NMR (400 MHz, $CDCl_3$) δ 7.62 (s, 1H), 2.87 – 2.78 (m, 2H), 2.65 (s, 3H), 2.60 – 2.56 (m, 2H), 2.55 (s, 3H), 1.91 – 1.84 (m, 2H), 1.83 – 1.77 (m, 2H).

^{13}C NMR (101 MHz, $CDCl_3$) δ 165.1, 161.9, 151.1, 148.0, 147.7, 137.4, 122.2, 117.5, 110.1, 108.9, 25.8, 24.2, 21.7, 21.6, 14.8, 9.2.

HRMS (ESI) m/z: [M+Na$^+$] Calculated for $C_{16}H_{15}NO_3Na$: 292.09441. Found: 292.09468.

IR (neat): 1712 (s), 1631 (w), 1608 (m), 1582 (w), 1431 (w), 1380 (w), 1348 (w), 1313 (w), 1281 (w), 1122 (m), 1091 (w), 1019 (w), 965 (w), 913 (w), 815 (w), 744 (w).

Mp: 217-220°C.

3-Methylbenzo[*d*]isoxazol-5-yl 3-(4,5-diphenyloxazol-2-yl)propanoate 5r

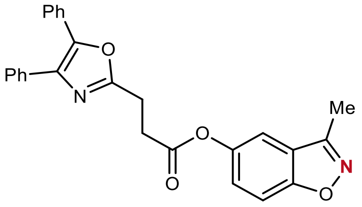

5r was prepared according to the general procedure V using **4r** (63.5 mg, 0.150 mmol, 1.0 equiv.), *N*-nitrosomorpholine (52.5 mg, 0.450 mmol, 3.0 equiv.), TsOH•H_2O (43 mg, 0.23 mmol, 1.5 equiv.) in EtOAc (1.5 mL) and subsequent ring closure with Et_3N (41.7 µL, 0.300 mmol, 2.0 equiv.) in dry DCE (2 mL) as well as MsCl (13 µL, 0.16 mmol, 1.1 equiv.) in dry DCE (1 mL). After purification by flash chromatography (*n*-pentane/EtOAc = 10/1), **5r** was obtained as a white solid (60.6 mg, 81%).

TLC: 0.54 (Pentane/EtOAc = 5/2).

^{1}H NMR (400 MHz, $CDCl_3$) δ 7.71 – 7.65 (m, 2H), 7.61 – 7.56 (m, 2H), 7.50 (dd, *J* = 8.9, 0.7 Hz, 1H), 7.41 – 7.32 (m, 7H), 7.27 (dd, *J* = 9.0, 2.3 Hz, 1H), 3.34 (t, *J* = 7.3 Hz, 2H), 3.20 (t, *J* = 6.9 Hz, 2H), 2.46 (s, 3H).

^{13}C NMR (101 MHz, $CDCl_3$) δ 171.2, 161.5, 160.7, 155.3, 146.6, 145.8, 135.3, 132.5, 128.8, 128.7, 128.65, 128.3,

128.0, 126.7, 124.2, 122.9, 113.6, 110.5, 31.4, 23.7, 10.1.

HRMS (EI) m/z: [M+Na$^+$] Calculated for $C_{26}H_{20}N_2O_4Na$: 447.13153; Found 447.13147.

IR (neat): 1744 (m), 1473 (w), 1264 (w), 1178 (w), 1145 (m), 1124 (w), 766 (m), 738 (s), 698 (m).

Mp: 160-161°C.

2-Methylbenzo[*d*]oxazol-5-yl 3-(4,5-diphenyloxazol-2-yl)propanoate 6r

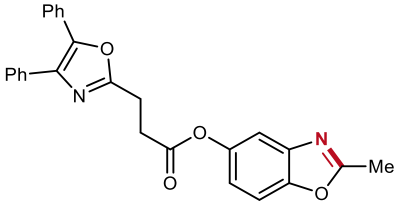

6r was prepared according to the general procedure VI using **4r** (63.5 mg, 0.150 mmol, 1.0 equiv.), *N*-nitrosomorpholine (52.5 mg, 0.450 mmol, 3.0 equiv.), TsOH•H_2O (43 mg, 0.23 mmol, 1.5 equiv.) in EtOAc (1.5 mL) and subsequent ring closure with Ms_2O (78.4 mg, 0.45 mmol, 1.5 equiv.). After purification by flash chromatography (*n*-pentane/EtOAc = 10/1), **6r** was obtained as yellow oil (38.2 mg, 60%).

TLC: 0.26 (Pentane/EtOAc = 5/2).

^{1}H NMR (300 MHz, $CDCl_3$) δ 7.69 – 7.63 (m, 2H), 7.61 – 7.55 (m, 2H), 7.45 – 7.29 (m, 8H), 7.04 (dd, *J* = 8.7, 2.4 Hz, 1H), 3.32 (td, *J* = 7.0, 1.4 Hz, 2H), 3.20 (td, *J* = 7.0, 1.4 Hz, 2H), 2.63 (s, 3H).

^{13}C NMR (76 MHz, $CDCl_3$) δ 171.1, 165.4, 161.5, 148.8, 147.3, 145.7, 142.3, 135.3, 132.5, 129.0, 128.8, 128.7, 128.6, 128.2, 128.0, 126.7, 118.4, 112.7, 110.4, 31.3, 23.6, 14.7.

HRMS (ESI) m/z: [M+H$^+$] Calculated for $C_{26}H_{20}N_2O_4Na$: 447.13153; Found 447.13157.

IR (neat): 1757 (w), 1575 (w), 1471 (w), 1437 (w), 1265 (w), 1219 (w), 1137 (s), 1058 (w), 1024 (w), 961 (w), 920 (w), 846 (w), 763 (s), 736 (w), 693 (s), 674 (m).

3-Methylbenzo[*d*]isoxazol-5-yl 2-(1-(4-chlorobenzoyl)-5-methoxy-2-methyl-1H-indol-3-yl)acetate 5s

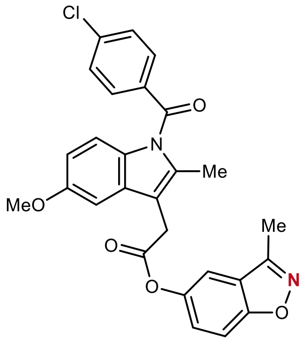

5s was prepared according to the general procedure V using **4s** (73.1 mg, 0.150 mmol, 1.0 equiv.), *N*-nitrosomorpholine (52.5 mg, 0.450 mmol, 3.0 equiv.), TsOH•H_2O (43 mg, 0.23 mmol, 1.5 equiv.) in EtOAc (1.5 mL) and subsequent ring closure with Et_3N (41.7 µL, 0.300 mmol, 2.0 equiv.) in dry DCE (2 mL) as well as MsCl (13 µL, 0.16 mmol, 1.1 equiv.) in dry DCE (1 mL). After purification by flash chromatography (*n*-pentane/EtOAc = 10/1),

5s was obtained as a white solid (58.5 mg, 80%).

TLC: 0.60 (*n*-Pentane/EtOAc = 5/2).

^{1}H NMR (300 MHz, $CDCl_3$) δ 7.72 – 7.64 (m, 2H), 7.53 – 7.44 (m, 3H), 7.35 (dd, *J* = 2.3, 0.6 Hz, 1H), 7.21 (dd, *J* = 8.9, 2.3 Hz, 1H), 7.07 (d, *J* = 2.5 Hz, 1H), 6.90 (d, *J* = 9.0 Hz, 1H), 6.71 (dd, *J* = 9.0, 2.6 Hz, 1H), 3.95 (s, 2H), 3.84 (s, 3H), 2.54 (s, 3H), 2.48 (s, 3H).

^{13}C NMR (76 MHz, $CDCl_3$) δ 169.7, 168.4, 160.7, 156.3, 155.3, 146.6, 139.6, 136.5, 133.9, 131.3, 131.0, 130.6, 129.3, 124.1, 122.9, 115.2, 113.4, 111.84, 111.81, 110.6, 101.4, 55.9, 30.6, 13.6, 10.2.

HRMS (EI) m/z: [M+Na$^+$] Calculated for $C_{27}H_{21}N_2ClO_5Na$: 511.1031; Found 511.1032.

IR (neat): 1753 (w), 1683 (m), 1474 (m), 1455 (m), 1355 (w), 1315 (s), 1218 (m), 1175 (m), 1119 (s), 1088 (m), 1065 (m), 1013 (w), 831 (w), 733 (s).

Mp: 126-127°C.

2-Methylbenzo[*d*]oxazol-5-yl 2-(1-(4-chlorobenzoyl)-5-methoxy-2-methyl-1H-indol-3-yl)acetate 6s

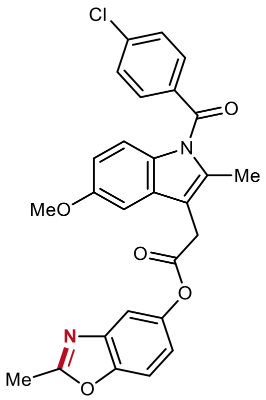

6s was prepared according to the general procedure VI using **4s** (73.1 mg, 0.150 mmol, 1.0 equiv.), *N*-nitrosomorpholine (52.5 mg, 0.450 mmol, 3.0 equiv.), TsOH•H_2O (43 mg, 0.23 mmol, 1.5 equiv.) in EtOAc (1.5 mL) and subsequent ring closure with Ms_2O (78.4 mg, 0.450 mmol, 1.5 equiv.). After purification by flash chromatography (*n*-pentane/EtOAc = 10/3), **6s** was obtained as brown solid (47.6 mg, 65%).

TLC: 0.26 (*n*-Pentane/EtOAc = 5/2).

^{1}H NMR (400 MHz, $CDCl_3$) δ 7.71 – 7.63 (m, 2H), 7.50 – 7.44 (m, 2H), 7.42 (d, *J* = 8.7 Hz, 1H), 7.35 (d, *J* = 2.2 Hz, 1H), 7.07 (d, *J* = 2.5 Hz, 1H), 6.97 (dd, *J* = 8.7, 2.3 Hz, 1H), 6.89 (d, *J* = 9.0 Hz, 1H), 6.70 (dd, *J* = 9.0, 2.5 Hz, 1H), 3.93 (s, 2H), 3.84 (s, 3H), 2.63 (s, 3H), 2.47 (s, 3H).

^{13}C NMR (76 MHz, $CDCl_3$) δ 169.7, 168.4, 165.5, 156.3, 148.8, 147.4, 142.3, 139.5, 136.4, 133.9, 131.3, 131.0, 130.6, 129.3, 118.2, 115.2, 112.6, 112.1, 112.0, 110.4, 101.3, 55.9, 30.6, 14.7, 13.6.

HRMS (EI) m/z: [M+Na$^+$] Calculated for $C_{27}H_{21}N_2ClO_5Na$: 511.1031; Found 511.1031.

IR (neat): 1752 (w), 1684 (m), 1576 (w), 1474 (m), 1437 (w), 1356 (w), 1315 (m), 1219 (m), 1130 (s), 925 (w), 833 (w), 754 (w), 736 (w).

Mp: 161-163°C.

➢ Skeletal editing of 4s as one-pot process starting from morpholine and larger scale synthesis

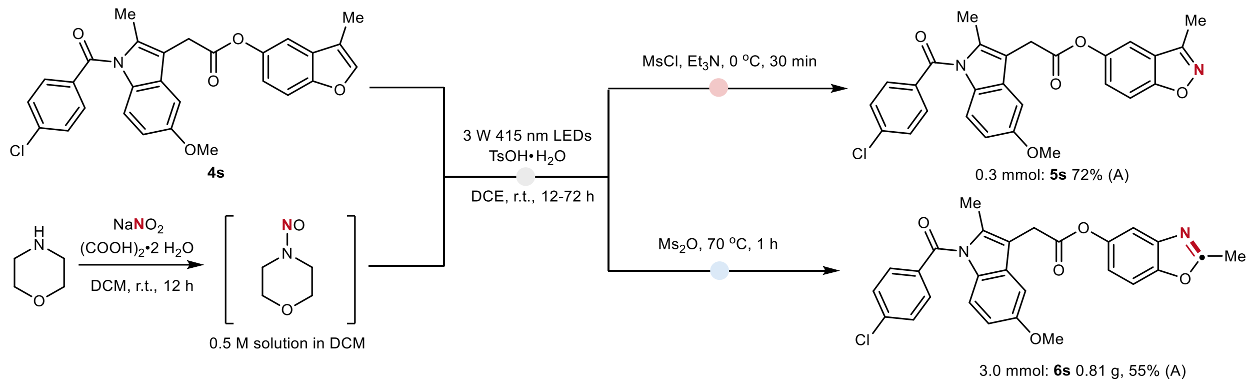

Preparation of 0.5 M 4-nitrosomorpholine solution in DCM

A suspension of $NaNO_2$ (2.76 g, 40.0 mmol, 2.0 equiv.), oxalic acid dihydrate (5.0 g, 40 mmol, 2.0 equiv.) and morpholine (1.75 mL, 20.0 mmol, 1.0 equiv.) in DCM (40 mL) was stirred vigorously at room temperature overnight. To the reaction mixture, anhydrous Na_2SO_4 was added to get a 0.5 M solution.

One-pot process for the preparation of 5s

Under argon, **4s** (147 mg, 0.300 mmol, 1.0 equiv.) was dissolved in dry DCE (1.2 mL). 4-Nitrosomorpholine DCM solution (1.8 mL, 0.5 M in DCM) was added. The reaction mixture was cooled down to 0 °C using an ice/water bath and camphor-10-sulfonic acid (CSA, 105 mg, 0.450 mmol, 1.5 equiv.) was added at 0 °C. The reaction was irradiated at 415 nm (3 W) for 12 h. After completed irradiation, Et_3N (0.17 mL, 1.2 mmol, 4.0 equiv.) was added and the reaction mixture was stirred at room temperature for 10 min. Then, the reaction mixture was cooled down to 0 °C using an ice/water bath, MsCl (25.5 µL, 0.330 mmol, 1.1 equiv.) in dry DCE (1 mL) was added dropwise slowly. The mixture was stirred at 0 °C for 30 min. The solvent was removed and the residue was subjected to a short flash chromatography (*n*-pentane/EtOAc = 2/1) to afford crude **5s** mixed with 4-nitrosomorpholine, which was further purified by reversed-phase MPLC (MeCN/water, v/v ratio was increased gradient from 20:80 to 65:35 over 9 min, then kept at 65:35 for 3 min, then increased from 65:35 to 90:10 over 2 min and finally kept at 90:10 for 7 min) to give **5s** as a white solid (104.8 mg, 72%).

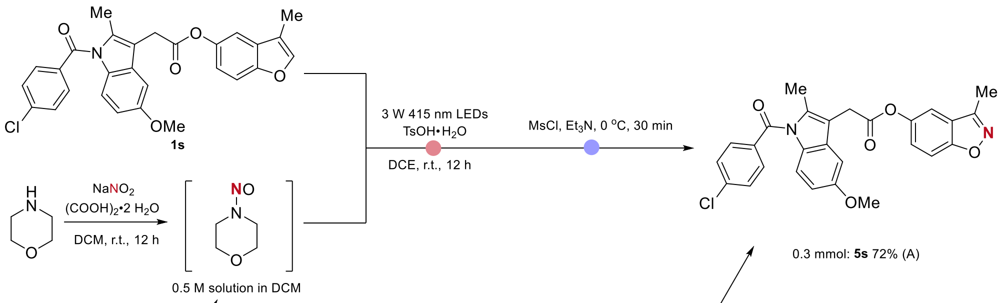

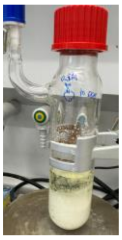

in-situ formed 0.5 M *N*-nitrosomorpholine solution in DCM

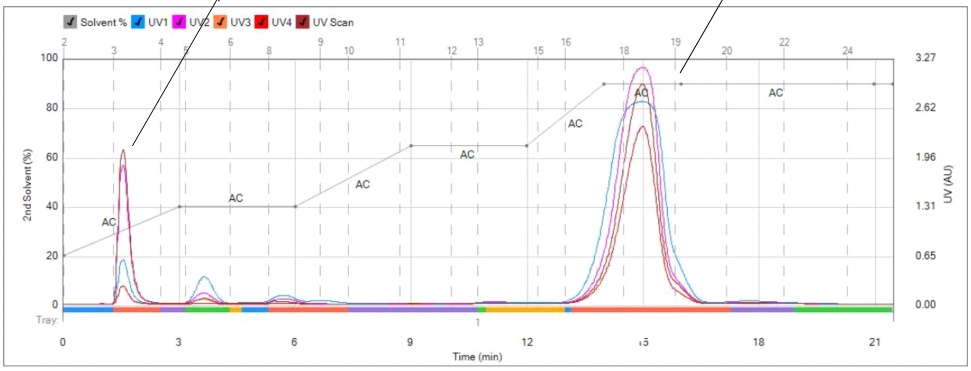

Gradient Table

	Min	Solvents	% 2nd
1	0.0	AC	20
2	3.0	AC	40
3	3.0	AC	40
4	3.0	AC	65
5	3.0	AC	65
6	2.0	AC	90
7	2.0	AC	90
8	5.0	AC	90
9	0.5	AC	90

AC with water
AC: Acetonitrile

Purity test of 5s by HPLC: (XDB-C18, CyH/^{i}PrOH 90:10, 230 nm)

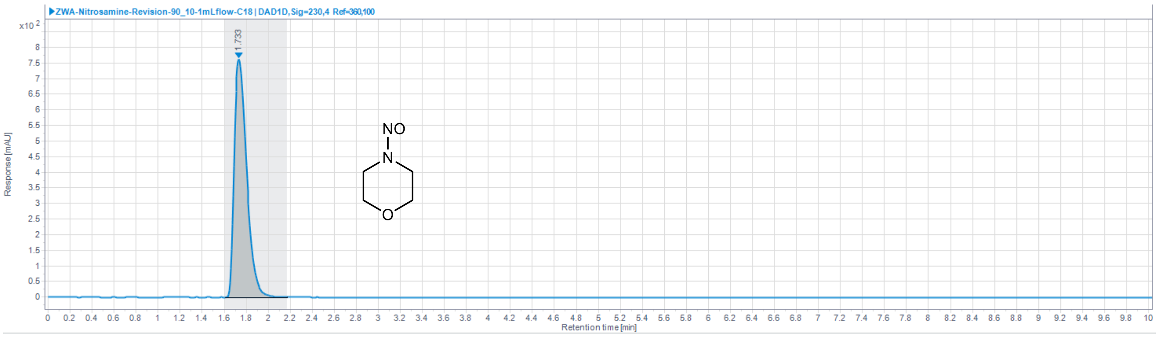

#	Signal description	RT (min)	Area	Area%	Height	Width
1	DAD1D,Sig=230,4 Ref=360,100	1.733	6045.214	100.000	762.955	0.560

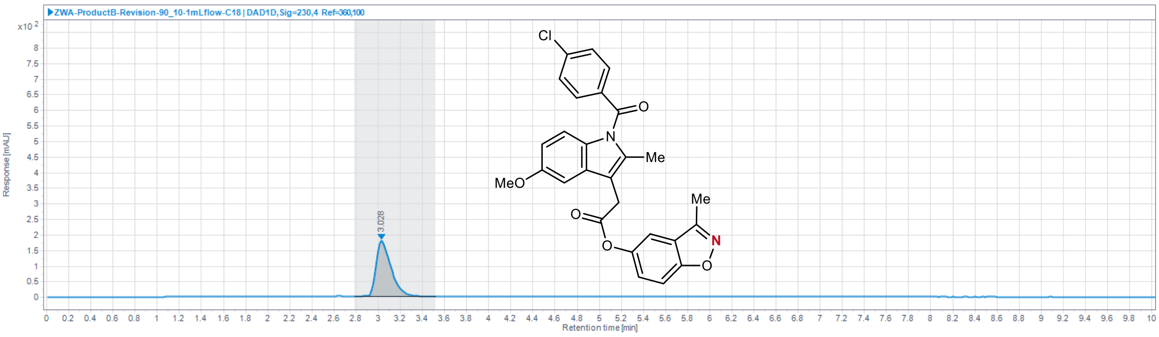

#	Signal description	RT (min)	Area	Area%	Height	Width
1	DAD1D,Sig=230,4 Ref=360,100	3.028	1712.258	100.000	180.085	0.734

Larger scale One-pot process for 6s

Under argon, **4s** (1.47 mg, 3.00 mmol, 1.0 equiv.) was dissolved in dry DCE (12 mL). 4-Nitrosomorpholine DCM solution (18 mL, 0.5 M in DCM) was added. The reaction mixture was cooled down to 0 °C using an ice/water bath and camphor-10-sulfonic acid (CSA, 1.05 mg, 4.50 mmol, 1.5 equiv.) was added at 0 °C. The reaction was irradiated at 415 nm (6 LEDs, 3 W) for 72 h. Aftre completed irradiation, the reaction mixture was cooled down to 0 °C using an ice/water bath, Ms_2O (523 mg, 3.00 mmol, 1.5 equiv.) in dry DCE (10 mL) was added dropwise slowly. The mixture was heated to 70 °C for 1 h. Then, sat. $NaHCO_3$ aqueous solution was added and the mixture was extracted with DCM (3 times). The organic phase was washed with brine and dried over Na_2SO_4. After filtration, the solvent was removed with a rotary evaporator under reduced pressure. The residue was purified by flash chromatography (*n*-pentane/EtOAc = 10/3) and **6s** was obtained as brown solid (0.81 g, 55%).

Methyl 3-methyl-1*H*-indazole-1-carboxylate-2-^{15}N Moc-^{15}N-**2a**

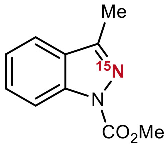

Moc-^{15}N-**2a** was prepared according to the general procedure III using Moc-**1a** (37.8 mg, 0.200 mmol, 1.0 equiv.) with 4-(nitroso-^{15}*N*)morpholine (46.9 mg, 0.400 mmol, 2.0 equiv.) as well as TsOH•H_2O (86 mg, 0.45 mmol, 1.5 equiv.) in EtOAc (3 mL) and subsequent ring closure with PPh_3 (121 mg, 0.460 mmol), DEAD (0.20 mL, 0.46 mmol, 40 wt.% in toluene) in dry THF (3 mL). After purification by flash chromatography (*n*-pentane/EtOAc = 10/1), Moc-^{15}N-**2a** was obtained as white solid (23.0 mg, 60%).

TLC: 0.38 (Pentane/EtOAc = 5/1).

^{1}H NMR (300 MHz, $CDCl_3$) δ 8.20 (d, *J* = 8.4 Hz, 1H), 7.66 (dt, *J* = 7.9, 1.0 Hz, 1H), 7.55 (ddd, *J* = 8.4, 7.1, 1.2 Hz, 1H), 7.33 (ddd, *J* = 8.0, 7.1, 0.9 Hz, 1H), 4.11 (s, 3H), 2.60 (d, *J* = 3.2 Hz, 3H).

^{13}C NMR (76 MHz, $CDCl_3$) δ 151.3 (d, *J* = 4.9 Hz), 149.5 (d, *J* = 1.8 Hz), 140.5, 129.4, 126.0 (d, *J* = 2.1 Hz), 123.8, 120.5 (d, *J* = 1.3 Hz), 114.6, 54.4, 12.4 (d, *J* = 9.0 Hz).

HRMS (ESI) m/z: [M+H$^+$] Calculated for $C_{10}H_{10}N^{15}NO_2H$ 192.07854; Found 192.07842.

IR (neat): 1760 (w), 1729 (s), 1521 (w), 1442 (s), 1396 (w), 1372 (s), 1339 (w), 1287 (w), 1239 (s), 1150 (w), 1078 (s), 1040 (w), 756 (s).

Mp: 64-65°C.

The ratio of ^{15}N labeling: >95%.

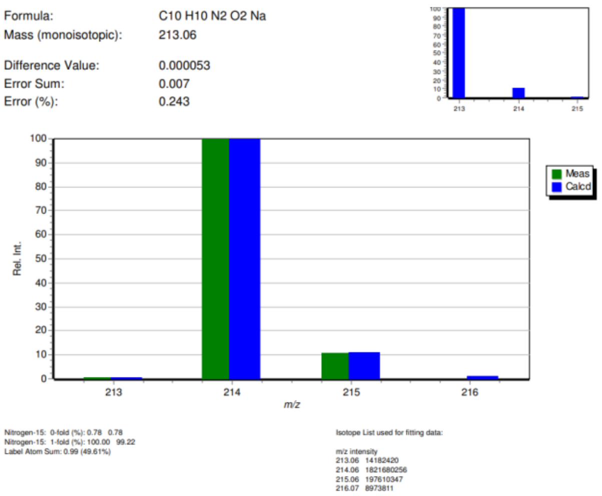

Dimethyl 3-methyl-1*H*-indazole-1,5-dicarboxylate-^{15}N Moc-^{15}N-**2ad**

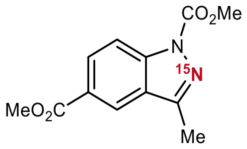

Moc-^{15}N-**2ad** was prepared according to the general procedure III using Moc-**1ad** (74.2 mg, 0.300 mmol, 1.0 equiv.) with 4-(nitroso-^{15}*N*)morpholine (70.3 mg, 0.600 mmol, 2.0 equiv.) as well as TsOH•H_2O (86 mg, 0.45 mmol, 1.5 equiv.) in EtOAc (3 mL) and subsequent ring closure with PPh_3 (72.4 mg, 0.28 mmol), DEAD (0.12 mL, 0.28 mmol, 40 wt.% in toluene) in dry THF (2 mL).After purification by flash chromatography (*n*-pentane/EtOAc = 10/1), Moc-^{15}N-**2ad** was obtained as white solid (18.0 mg, 26%). The yield of oxime intermediate was 46% (36.8 mg, 0.138 mmol). In this case, indole Moc-**1ad** was recovered (28.5 mg, 39%).

TLC: 0.36 (Pentane/EtOAc = 5/2).

^{1}H NMR (599 MHz, $CDCl_3$) δ 8.42 (t, *J* = 1.2 Hz, 1H), 8.25 – 8.23 (m, 2H), 4.14 (s, 3H), 3.97 (s, 3H), 2.65 (d, *J* = 3.3 Hz, 3H).

^{13}C NMR (151 MHz, $CDCl_3$) δ 166.7, 151.1 (d, *J* = 5.0 Hz), 150.2 (d, *J* = 1.8 Hz), 142.8, 130.4, 126.2, 126.1 (d, *J* = 2.0 Hz), 123.3 (d, *J* = 1.4 Hz), 114.5, 54.7, 52.5, 12.4 (d, *J* = 8.8 Hz).

^{15}N NMR (61 MHz, $CDCl_3$) δ -74.5.

HRMS (ESI) m/z: [M+Na^{+}] Calculated for $C_{12}H_{12}N^{15}NO_4Na$: 272.06596; Found 272.06561.

IR (neat): 1717 (s), 1437 (m), 1394 (m), 1373 (m), 1341 (w), 1281 (s), 1232 (s), 1113 (w), 1069 (s), 1034 (w), 768 (m), 748 (m).

Mp: 132-133°C.

The ratio of ^{15}N labeling: >90%.

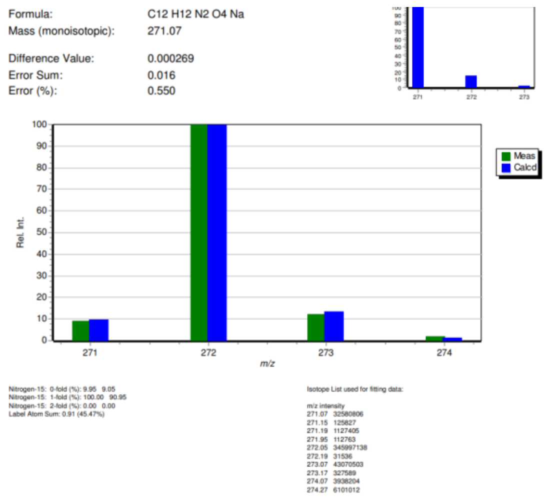

Unsuccessful example:

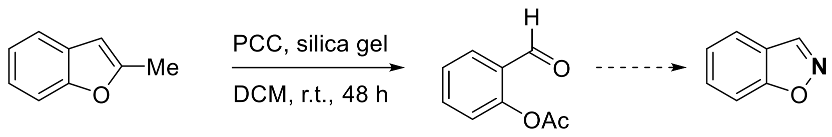

4t trace **5t**

5t was prepared according to the general procedures IX using commercial 2-methylbenzofuran **4t** (0.11 mL, 1.0 mmol, 1.0 equiv.) with silica gel (2.4 g) and PCC (1.12 g, 5.4 mmol, 5.4 equiv.) in DCM (50 mL). However, the oxidative cleavage step failed under current conditions and only a trace amount of 2-formylphenyl acetate intermediates were observed by crude NMR.

7. X-ray crystal structure analysis of 2aa, 5r and 6s

X-ray crystal structure analysis of 2aa (stu10716): A colorless, prism-like specimen of $C_{18}H_{20}N_4O_2$, approximate dimensions 0.045 mm x 0.089 mm x 0.145 mm, was used for the X-ray crystallographic analysis. The X-ray intensity data were measured on a single crystal diffractometer Bruker D8 Venture Photon III system equipped with a micro focus tube Mo ImS (MoKα, λ = 0.71073 Å) and a MX mirror monochromator. A total of 1510 frames were collected. The total exposure time was 14.47 hours. The frames were integrated with the Bruker SAINT software package using a narrow-frame algorithm. The integration of the data using a triclinic unit cell yielded a total of 17747 reflections to a maximum θ angle of 27.56° (0.77 Å resolution), of which 3783 were independent (average redundancy 4.691, completeness = 99.3%, R_{int} = 4.59%, R_{sig} = 3.44%) and 3149 (83.24%) were greater than $2\sigma(F^2)$. The final cell constants of a = 8.6637(2) Å, b = 10.2305(3) Å, c = 10.4771(3) Å, α = 94.730(1)°, β = 104.248(1)°, γ = 111.231(1)°, volume = 823.70(4) $Å^3$, are based upon the refinement of the XYZ-centroids of 7594 reflections above 20 σ(I) with 5.282° < 2θ < 55.05°. Data were corrected for absorption effects using the multi-scan method (SADABS). The ratio of minimum to maximum apparent transmission was 0.854. The calculated minimum and maximum transmission coefficients (based on crystal size) are 0.9870 and 0.9960. The structure was solved and refined using the Bruker SHELXTL Software Package, using the space group *P*-1, with Z = 2 for the formula unit, $C_{18}H_{20}N_4O_2$. The final anisotropic full-matrix least-squares refinement on F^2 with 227 variables converged at R1 = 3.96%, for the observed data and wR2 = 10.60% for all data. The goodness-of-fit was 1.051. The largest peak in the final difference electron density synthesis was 0.286 $e^-/Å^3$ and the largest hole was -0.206 $e^-/Å^3$ with an RMS deviation of 0.043 $e^-/Å^3$. On the basis of the final model, the calculated density was 1.308 g/cm^3 and F(000), 344 e^-. CCDC Nr.: 2402126.

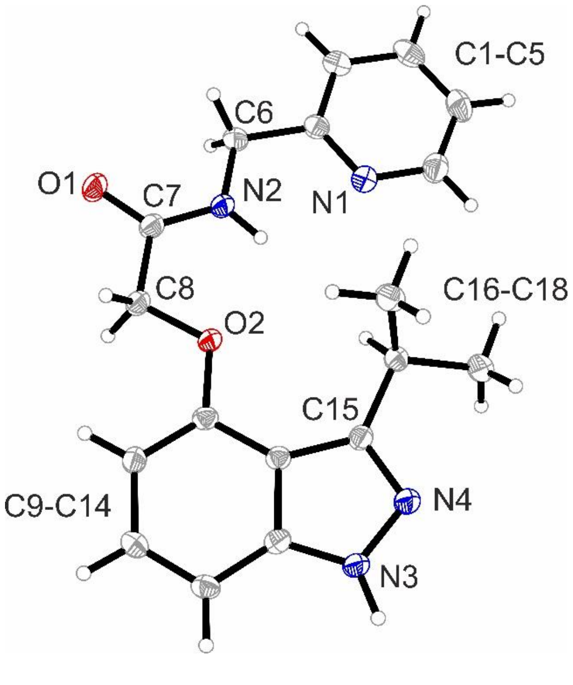

Figure S1: Crystal structure of compound **2aa**.
Thermal ellipsoids are shown at 50% probability.

X-ray crystal structure analysis of 5r (stu10709): A colorless, needle-like specimen of $C_{26}H_{20}N_2O_4$, approximate dimensions 0.057 mm x 0.077 mm x 0.117 mm, was used for the X-ray crystallographic analysis. The X-ray intensity data were measured (λ = 1.54178 Å). The integration of the data using an orthorhombic unit cell yielded a total of 31139 reflections to a maximum θ angle of 66.80° (0.84 Å resolution), of which 3708 were independent (average redundancy 8.398, completeness = 99.7%, R_{int} = 4.61%, R_{sig} = 2.71%) and 3181 (85.79%) were greater than 2σ(F^2). The final cell constants of a = 12.7213(4) Å, b = 7.6522(3) Å, c = 43.0588(13) Å, volume = 4191.6(2) Å^3, are based upon the refinement of the XYZ-centroids of reflections above 20 σ(I). The calculated minimum and maximum transmission coefficients (based on crystal size) are 0.9180 and 0.9590. The final anisotropic full-matrix least-squares refinement on F^2 with 290 variables converged at R1 = 3.43%, for the observed data and wR2 = 8.93% for all data. The goodness-of-fit was 1.035. The largest peak in the final difference electron density synthesis was 0.167 e^-/Å^3 and the largest hole was -0.252 e^-/Å^3 with an RMS deviation of 0.041 e^-/Å^3. On the basis of the final model, the calculated density was 1.345 g/cm^3 and F(000), 1776 e^-. CCDC Nr.: 2392859.

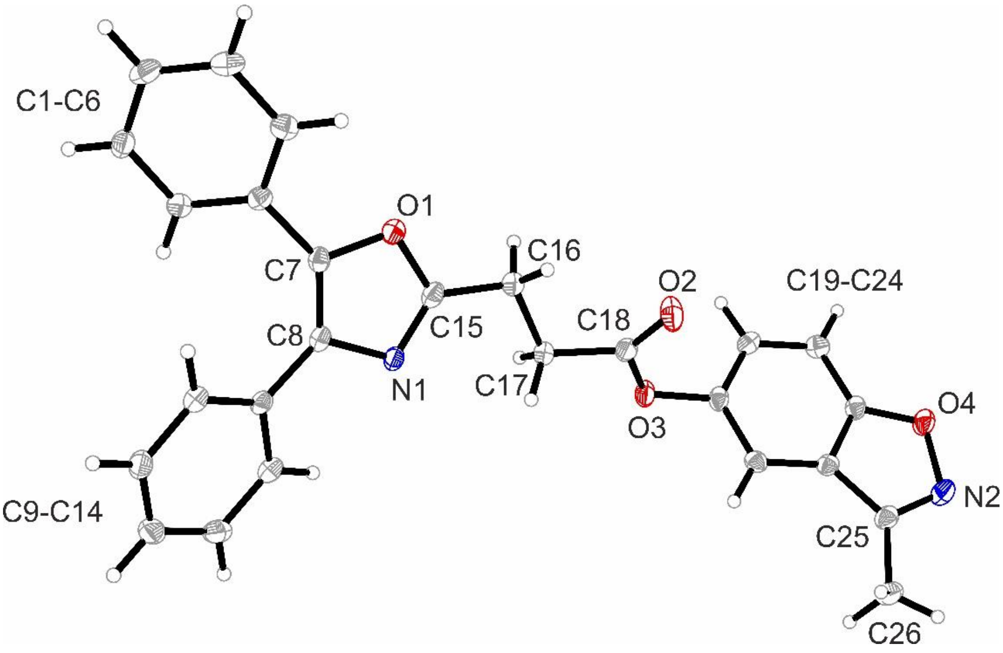

Figure S2: Crystal structure of compound **5r**.
Thermal ellipsoids are shown at 50% probability.

X-ray crystal structure analysis of 6s (stu10742): A colorless, plate-like specimen of $C_{27}H_{21}ClN_2O_5$, approximate dimensions 0.035 mm x 0.044 mm x 0.206 mm, was used for the X-ray crystallographic analysis. The X-ray intensity data were measured on a single crystal Bruker D8 Venture Photon III Diffractometer system equipped with a micro focus tube CuKα (CuKα, λ = 1.54178 Å) and a MX mirror monochromator. The integration of the data using a monoclinic unit cell yielded a total of 28754 reflections to a maximum θ angle of 66.80° (0.84 Å resolution), of which 3903 were independent (average redundancy 7.367, completeness = 98.9%, R_{int} = 5.06%, R_{sig} = 3.38%) and 3676 (94.18%) were greater than 2σ(F^2). The final cell constants of a = 16.6307(14) Å, b = 4.3025(4) Å, c = 31.672(3) Å, β = 100.080(3)°, volume = 2231.3(3) $Å^3$, are based upon the refinement of the XYZ-centroids of reflections above 20 σ(I). Data were corrected for absorption effects using the Multi-Scan method (SADABS). The calculated minimum and maximum transmission coefficients (based on crystal size) are 0.6970 and 0.9370. The structure was solved and refined using the Bruker SHELXTL Software Package, using the space group $P2_1/c$, with Z = 4 for the formula unit, $C_{27}H_{21}ClN_2O_5$. The final anisotropic full-matrix least-squares refinement on F^2 with 319 variables converged at R1 = 5.84%, for the observed data and wR2 = 17.13% for all data. The goodness-of-fit was 1.026. The largest peak in the final difference electron density synthesis was 0.634 $e^-/Å^3$ and the largest hole was -0.458 $e^-/Å^3$ with an RMS deviation of 0.079 $e^-/Å^3$. On the basis of the final model, the calculated density was 1.455 g/cm^3 and F(000), 1016 e^-. CCDC Nr.: 2407817.

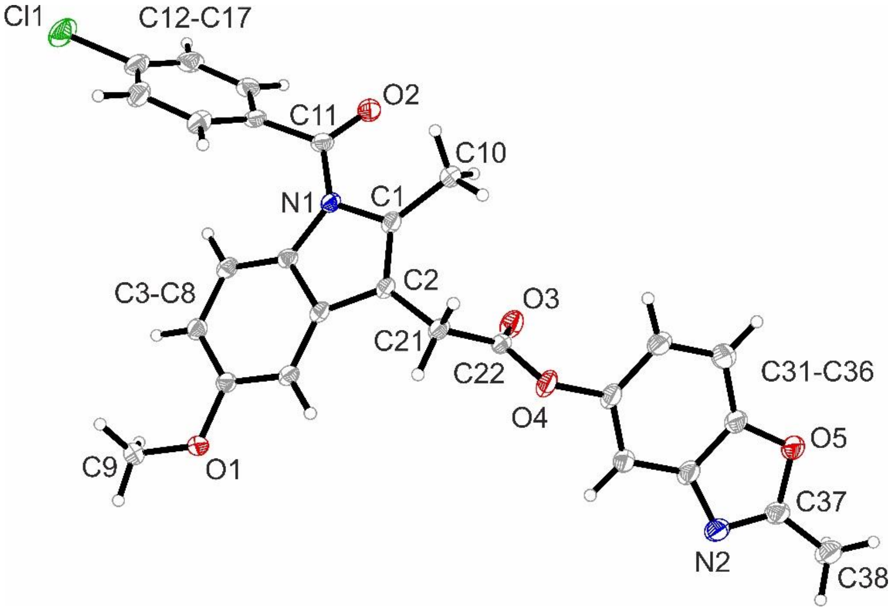

Figure S3: Crystal structure of compound **6s**.
Thermal ellipsoids are shown at 50% probability.

8. References

1. Bruker AXS (**2021**) *APEX4 Version 2021.4-0*, *SAINT Version 8.40B* and *SADABS Bruker AXS area detector scaling and absorption correction Version 2016/2*, Bruker AXS Inc., Madison, Wisconsin, USA.
2. Sheldrick, G. M., *SHELXT – Integrated space-group and crystal-structure determination*, *Acta Cryst.,* **2015**, *A71*, 3-8.
3. Sheldrick, G.M., *Crystal structure refinement with SHELXL*, *Acta Cryst.*, **2015**, *C71 (1)*, 3-8.
4. Bruker AXS (**1998**) *XP – Interactive molecular graphics, Version 5.1,* Bruker AXS Inc., Madison, Wisconsin, USA.
5. Trost, B. M., Zhang, Y. & Zhang, T. Direct N-Carbamoylation of 3-Monosubstituted Oxindoles with Alkyl Imidazole Carboxylates. *J. Org. Chem.* **74**, 5115–5117 (2009).
6. Kumar, N., Maity, A., Gavit, V. R. & Bisai, A. A catalytic *N*-deacylative alkylation approach to hexahydropyrrolo[2,3-*b*]indole alkaloids. *Chem. Commun.* **54**, 9083–9086 (2018).
7. Cheng, H. *et al.* Highly Enantioselective Friedel–Crafts Alkylation/*N*-Hemiacetalization Cascade Reaction with Indoles. *Angew. Chem. Int. Ed.* **52**, 3250–3254 (2013).
8. Siqueira, F. A., Taylor, J. G. & Correia, C. R. D. The first intramolecular Heck–Matsuda reaction and its application in the syntheses of benzofurans and indoles. *Tetrahedron Lett.* **51**, 2102–2105 (2010).
9. Beaud, R., Guillot, R., Kouklovsky, C. & Vincent, G. Regioselective Hydroarylation Reactions of C3 Electrophilic *N* -Acetylindoles Activated by $FeCl_3$: An Entry to 3-(Hetero)arylindolines. *Chem. Eur. J.* **20**, 7492–7500 (2014).
10. Xie, T. *et al.* Cu-Catalyzed Dimerization of Indole Derived Oxime Acetate for Synthesis of Biimidazo[1,2-*a*]indoles. *J. Org. Chem.* **86**, 5518–5529 (2021).
11. Li, S., Liu, X., Tung, C.-H. & Liu, L. Late-Stage Chemo- and Enantioselective Oxidation of Indoles to C3-Monosubstituted Oxindoles. *J. Am. Chem. Soc.* **145**, 27120–27130 (2023).
12. Song, H. *et al.* HFIP as Protonation Reagent and Solvent for Regioselective Alkylation of Indoles with All-Carbon Centers. *J. Org. Chem.* **87**, 1086–1097 (2022).
13. Xie, J.-J., Wang, Z.-Q. & Jiang, G.-F. Metal-free oxidative trifluoromethylation of indoles with CF_3SO_2Na on the C2 position. *RSC Adv.* **9**, 35098–35101 (2019).
14. Zhang, Q. *et al.* Titanium tetrachloride promoted cyclodehydration of aryloxyketones: Facile synthesis of benzofurans and naphthofurans with high regioselectivity. *Tetrahedron Lett.* **60**, 1337–1340 (2019).
15. Huff, J. R. *et al.* *N*-(1,3,4,6,7,12b-hexahydro-2*H*-benzo[*b*]furo[2,3-*a*]quinolizin-2-yl)-*N*-methyl-2-hydroxyethanesulfonamide: a potent and selective α_2-adrenoceptor antagonist. *J. Med. Chem.* **28**, 1756–1759 (1985).
16. Ji, X., Li, Y., Ding, W. & Zhang, Q. Substrate-Tuned Catalysis of the Radical *S* -Adenosyl-L-Methionine Enzyme NosL Involved in Nosiheptide Biosynthesis. *Angew. Chem. Int. Ed.* **54**, 9021–9024 (2015).
17. Jiang, S. *et al.* Facile access to benzofuran derivatives through radical reactions with heteroatom-centered super-electron-donors. *Nat. Commun.* **14**, 7381 (2023).
18. Kraus*, G. A. & Schroeder, J. D. Halogen-Metal Exchange/Cyclization of Iodoketones: A Direct Synthesis of 3-Arylbenzofurans. *Synlett* 2504–2506 (2005) doi:10.1055/s-2005-872690.
19. Zeng, W. *et al.* Facile synthesis of benzofurans *via* copper-catalyzed aerobic oxidative cyclization of phenols and alkynes. *Chem. Commun.* **49**, 6611 (2013).
20. Tan, J. *et al.* Synthesis of 2,3-disubstituted Benzofuran via $HNTf_2$ /TMSOTf Catalyzed Intermolecular Reaction of *o*-alkenyl phenols and Aldehydes. *Adv. Synth. Catal.* **365**, 983–989 (2023).
21. Demange, L. *et al.* Synthesis and Pharmacological in Vitro and in Vivo Evaluations of Novel Triazole Derivatives as Ligands of the Ghrelin Receptor. 1. *J. Med. Chem.* **50**, 1939–1957 (2007).
22. Ma, Y.-Q. & Tian, S.-K. Catalyst-free visible light-promoted defunctionalization of alkyl isocyanides with a hydrosilane through C–N bond cleavage. *Org. Biomol. Chem.* **22**, 2562–2565 (2024).

23. Guo, R. *et al.* Stereodivergent Alkyne Hydrofluorination Using Protic Tetrafluoroborates as Tunable Reagents. *Angew. Chem. Int. Ed.* **59**, 16651–16660 (2020).
24. Reay, A. J., Williams, T. J. & Fairlamb, I. J. S. Unified mild reaction conditions for C2-selective Pd-catalysed tryptophan arylation, including tryptophan-containing peptides. *Org. Biomol. Chem.* **13**, 8298–8309 (2015).
25. Weng, Y., Xu, X., Chen, H., Zhang, Y. & Zhuo, X. Tandem Electrochemical Oxidative Azidation/Heterocyclization of Tryptophan-Containing Peptides under Buffer Conditions. *Angew. Chem. Int. Ed.* **61**, e202206308 (2022).
26. Xu, Z. *et al.* Hydroxylation with Unusual Stereoinversion Catalyzed by an Fe^{II}/2-OG Dependent Oxidase and 3,6-Diene-2,5-diketopiperazine Formation in the Biosynthesis of Brevianamide K. *Angew. Chem. Int. Ed.* **62**, e202216989 (2023).
27. He, T. *et al.* Aggregation-induced emission enhancement of polycyclic aromatic alkaloid derivatives and the crucial role of excited-state proton-transfer. *Chem. Commun.* **47**, 2907 (2011).
28. Zheng, M. *et al.* Indole derivatives as potent inhibitors of 5-lipoxygenase: Design, synthesis, biological evaluation, and molecular modeling. *Bioorg. Med. Chem. Lett.* **17**, 2414–2420 (2007).
29. Xiang, J., Wang, J., Wang, M., Meng, X. & Wu, A. One-pot total synthesis: the first total synthesis of chiral alkaloid pimprinol A and the facile construction of its natural congeners from amino acids. *Tetrahedron* **70**, 7470–7475 (2014).
30. Farizyan, M., Mondal, A., Mal, S., Deufel, F. & Van Gemmeren, M. Palladium-Catalyzed Nondirected Late-Stage C–H Deuteration of Arenes. *J. Am. Chem. Soc.* **143**, 16370–16376 (2021).
31. Dalla Via, L. *et al.* Novel Pyrone Side Tetracyclic Psoralen Derivatives: Synthesis and Photobiological Evaluation. *J. Med. Chem.* **46**, 3800–3810 (2003).
32. Garazd, Ya. L., Garazd, M. M., Shilin, S. V. & Khilya, V. P. Modified Coumarins. 3. Psoralen And Allopsoralen Analogs. *Chem. Nat. Compd.* **37**, 409–420 (2001).
33. Wang, J. *et al.* A Scalable and Metal-Free Synthesis of Indazoles from 2-Aminophenones and In Situ Generated De-Boc-Protected *O* -Mesitylsulfonyl Hydroxylamine Derivatives. *J. Org. Chem.* **88**, 13049–13056 (2023).
34. Kuwano, R. & Kashiwabara, M. Ruthenium-Catalyzed Asymmetric Hydrogenation of *N* -Boc-Indoles. *Org. Lett.* **8**, 2653–2655 (2006).
35. Conlon, I. L., Konsein, K., Morel, Y., Chan, A. & Fletcher, S. Construction of 1*H*-indazoles from ortho-aminobenzoximes by the Mitsunobu reaction. *Tetrahedron Lett.* **60**, 150929 (2019).
36. Hernández-Ruiz, R., Solas, M., Suárez-Pantiga, S., Pedrosa, M. R. & Sanz, R. γ-Terpinene: Biorenewable Reductant for the Molybdenum-Catalyzed Reduction of Sulfoxides, *N*-Oxides and Nitroarenes. *Adv. Synth. Catal.* e202401387 (2025) doi:10.1002/adsc.202401387.
37. Jiang, X. *et al.* A mild system for synthesis of aldoximes and ketoximes in the presence of *N*-hydroxyphthalimide in aqueous system. *Tetrahedron* **74**, 5879–5885 (2018).
38. Kondo, Y. *et al.* Scandium(III) Triflate Catalyzed Direct Synthesis of *N*-Unprotected Ketimines. *Org. Lett.* **22**, 120–125 (2020).
39. Yu, X. *et al.* Tandem Reduction, Ammonolysis, Condensation, and Deamination Reaction for Synthesis of Benzothiadiazines and 1-(Phenylsulfonyl)-1 *H* -benzimidazoles. *J. Org. Chem.* **87**, 14738–14752 (2022).
40. Youngsaye, W. *et al.* ML212: A small-molecule probe for investigating fluconazole resistance mechanisms in *Candida albicans*. *Beilstein J. Org. Chem.* **9**, 1501–1507 (2013).
41. Zhang, X., Huang, R., Marrot, J., Coeffard, V. & Xiong, Y. Hypervalent iodine-mediated synthesis of benzoxazoles and benzimidazoles via an oxidative rearrangement. *Tetrahedron* **71**, 700–708 (2015).
42. Wang, Y., Cacchillo, E. M., Niedzwiedzki, D. M. & Taylor, J. Ability of the Putative Decomposition Products of 2,3-dioxetanes of Indoles to Photosensitize Cyclobutane Pyrimidine Dimer (CPD) Formation and its Implications for the "Dark" (Chemisensitized) Pathway to CPDs in Melanocytes[†]. *J. Photochem. Photobiol. A* **98**, 442–454 (2022).
43. Xu, J., Liang, L., Zheng, H., Chi, Y. R. & Tong, R. Green oxidation of indoles using halide catalysis. *Nat. Commun.*

10, 4754 (2019).

44. Xu, W., Zhao, J., Li, X. & Liu, Y. Selective [5+1] and [5+2] Cycloaddition of Ynamides or Propargyl Esters with Benzo[*d*]isoxazoles via Gold Catalysis. *J. Org. Chem.* **83**, 15470–15485 (2018).
45. Choi, M., Mehra, M. K. & Lee, C. W. Copper-Catalyzed One-Pot Arylation and Cyclization of Diaryliodonium Salts Derived from *o* -Iodoanilines for Indolo[2,3- *b*]indoles Syntheses. *Eur. J. Org. Chem.* **26**, e202201286 (2023).
46. Wu, M., Hu, X., Liu, J., Liao, Y. & Deng, G.-J. Iron-Catalyzed 2-Arylbenzoxazole Formation from *o*-Nitrophenols and Benzylic Alcohols. *Org. Lett.* **14**, 2722–2725 (2012).
47. Gladkov, A. A., Levin, V. V. & Dilman, A. D. Radical Substitution of Polyfluoroarenes and Heteroarenes Promoted by Photoredox Activation of Organozinc Iodides. *Adv. Synth. Catal.* **365**, 3387–3391 (2023).

9. NMR spectra of products

Methyl 5-cyano-3-methyl-1*H*-indole-1-carboxylate Moc-**1e**

^{1}H NMR (300 MHz, $CDCl_3$)

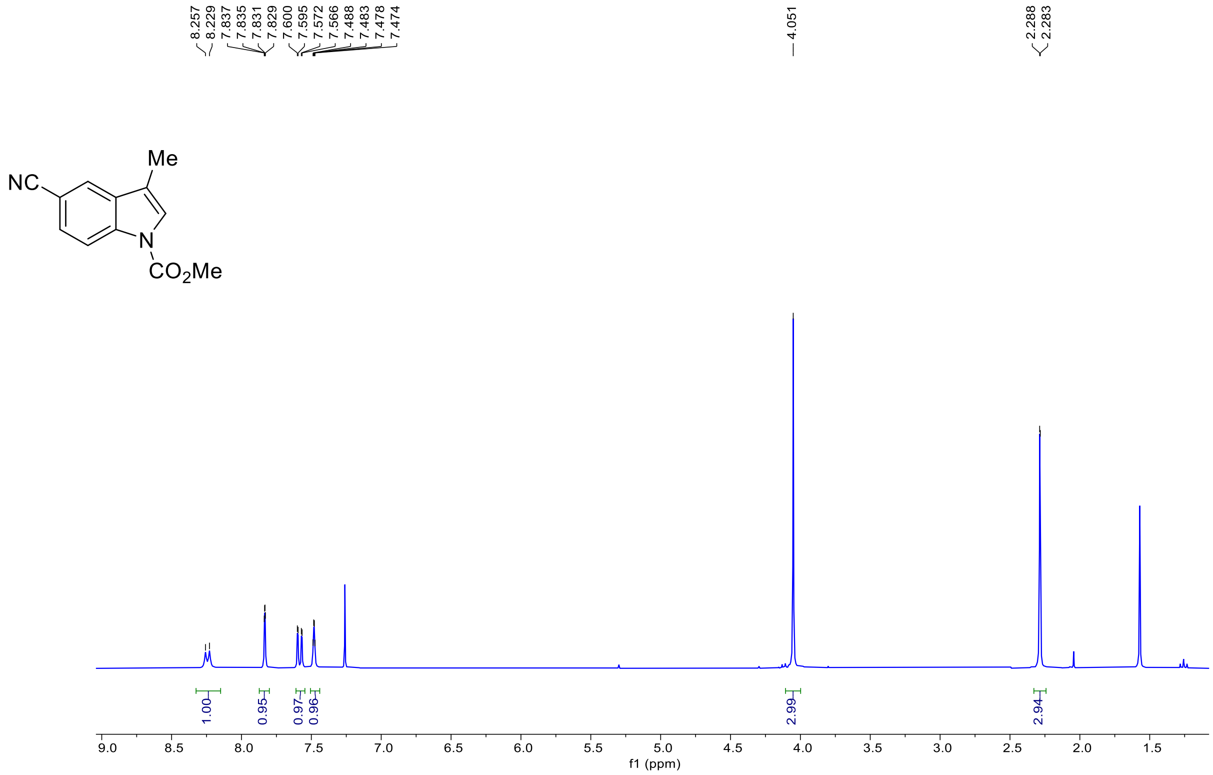

^{13}C NMR (101 MHz, $CDCl_3$)

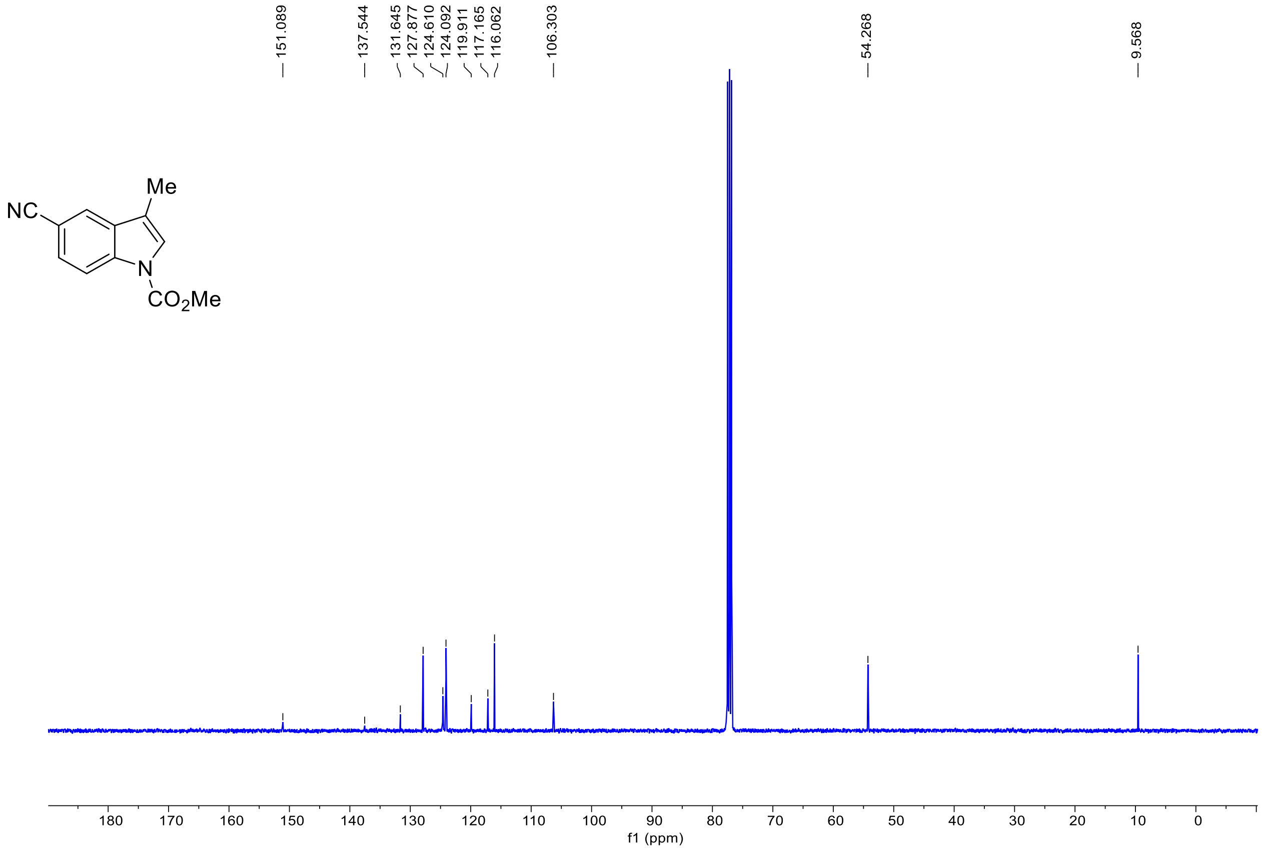

Methyl 6-(benzyloxy)-3-methyl-1*H*-indole-1-carboxylate Moc-**1f**

^{1}H NMR (400 MHz, $CDCl_3$)

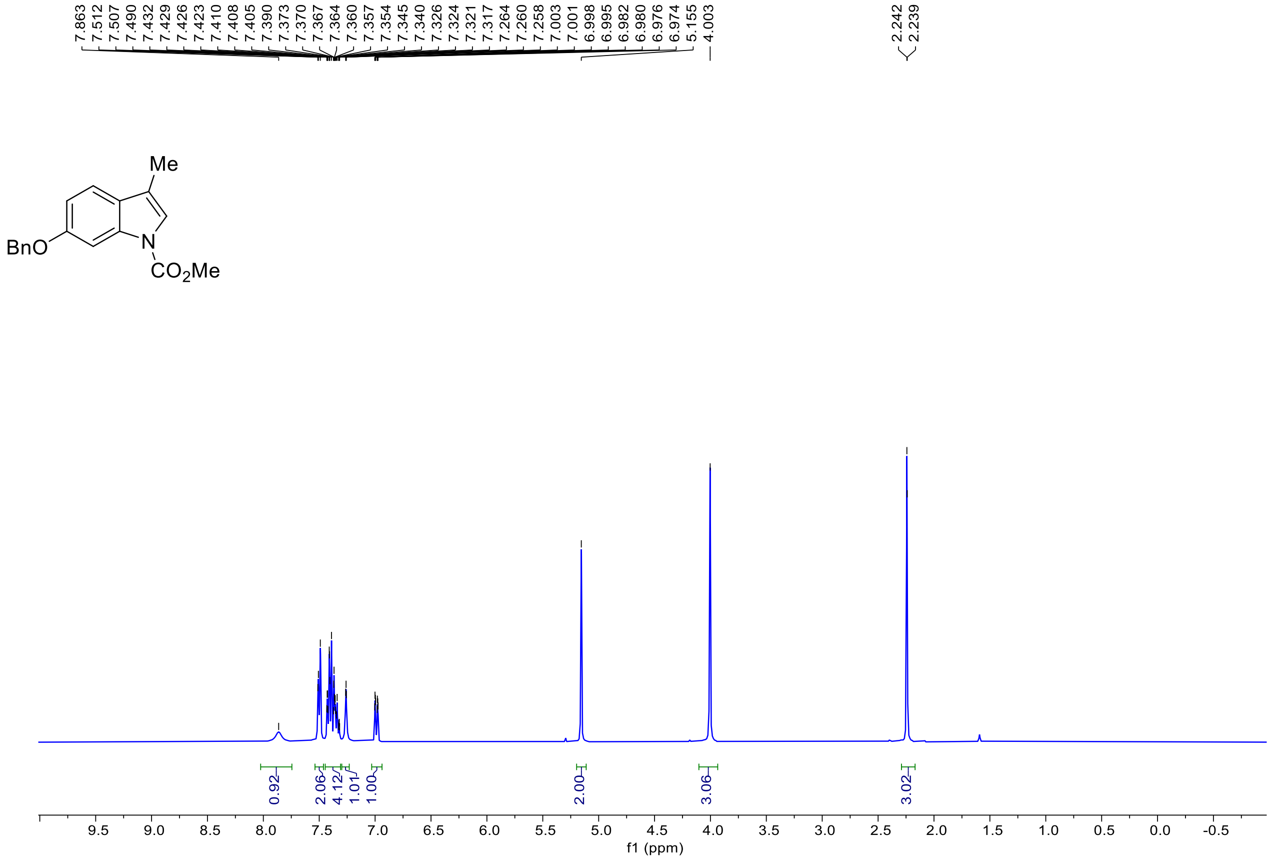

^{13}C NMR (101 MHz, $CDCl_3$)

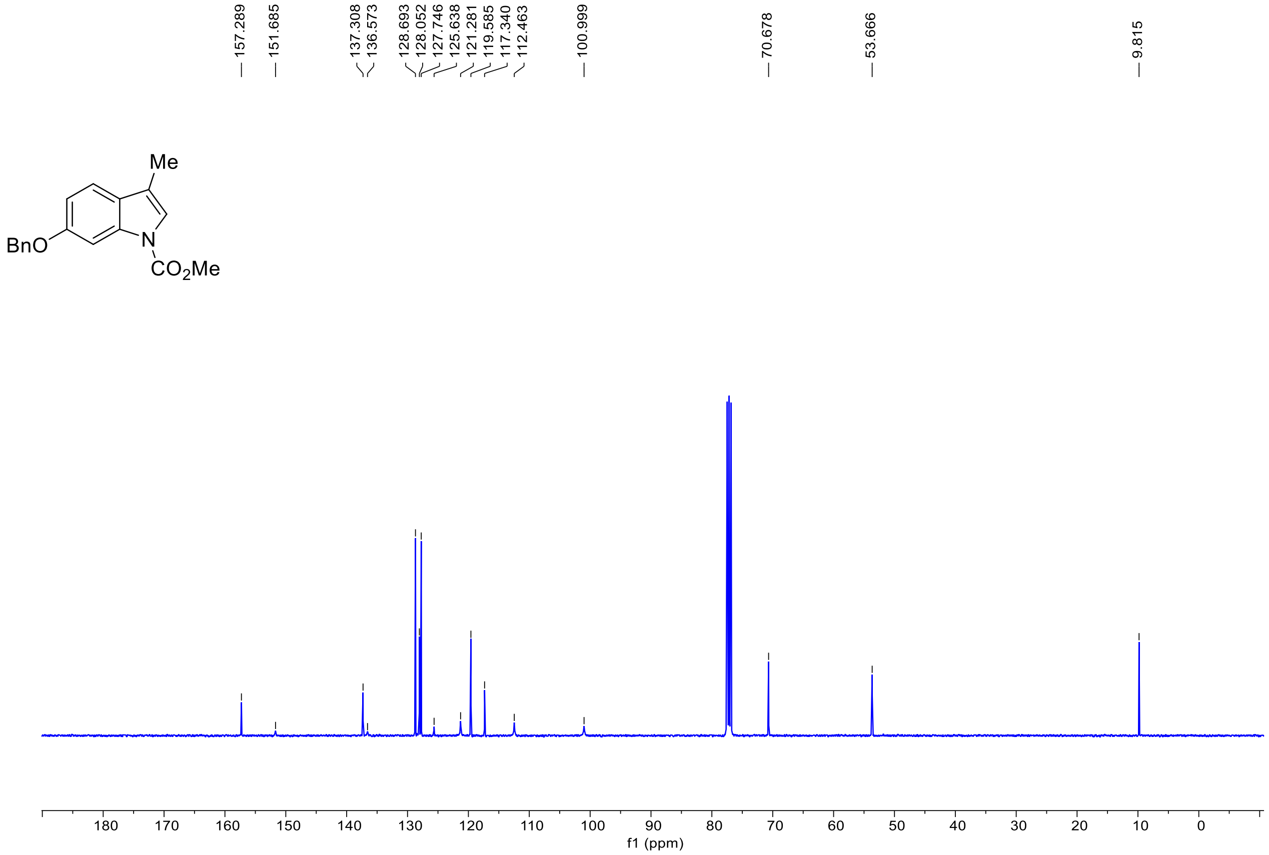

***tert*-Butyl 5-bromo-6-chloro-3-methyl-1*H*-indole-1-carboxylate Boc-1g**

^{1}H NMR (300 MHz, $CDCl_3$, containing tautomers)

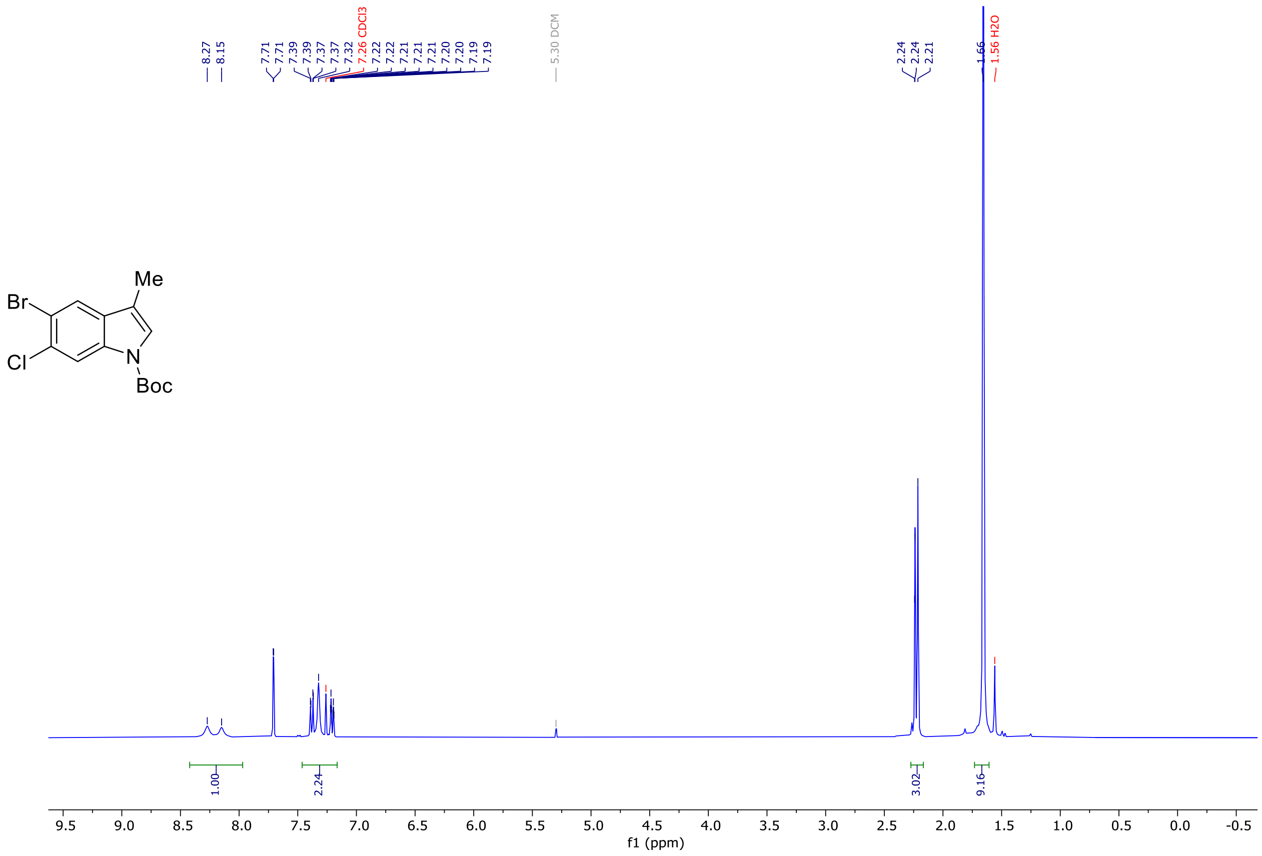

^{13}C NMR (101 MHz, $CDCl_3$, containing tautomers)

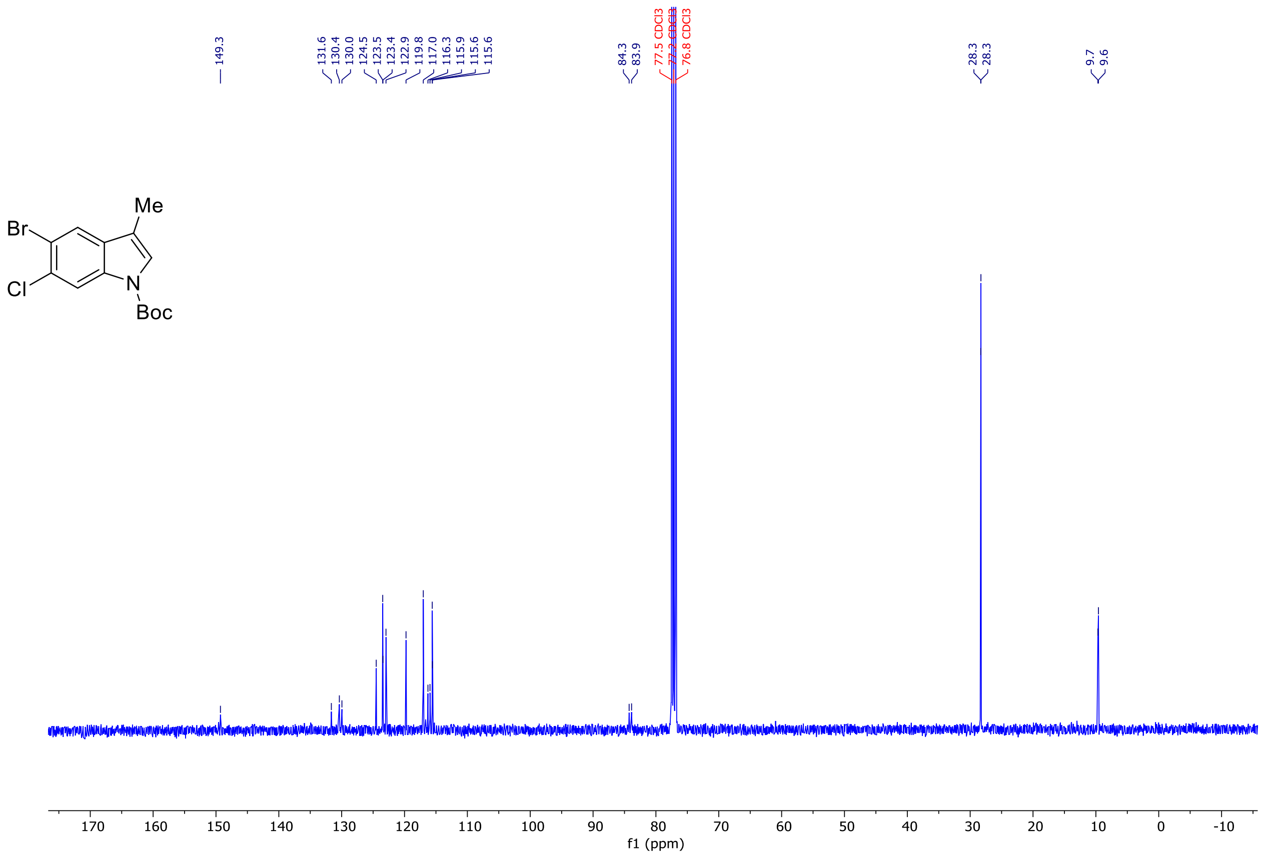

Methyl 3-isobutyl-1H-indole-1-carboxylate Moc-**1h**

^{1}H NMR (400 MHz, $CDCl_3$)

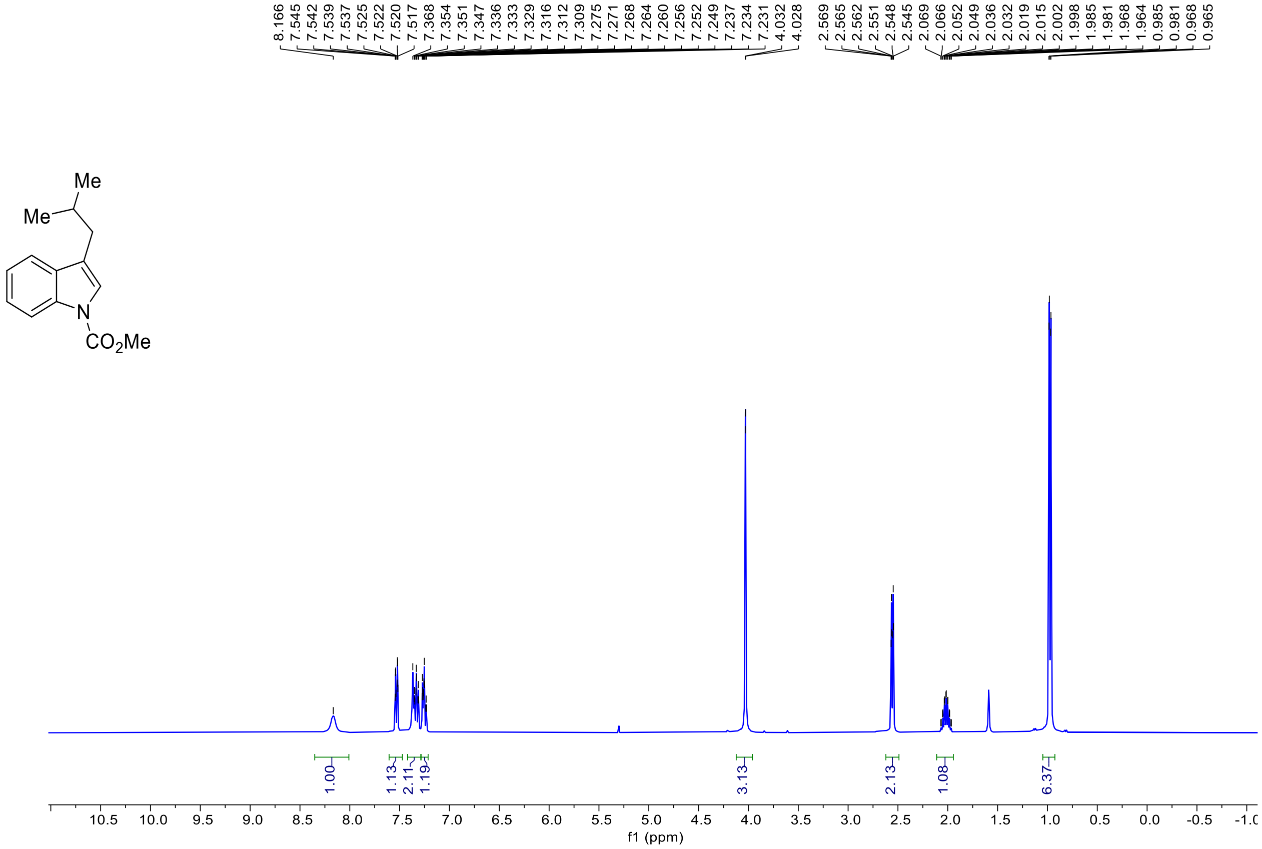

^{13}C NMR (101 MHz, $CDCl_3$)

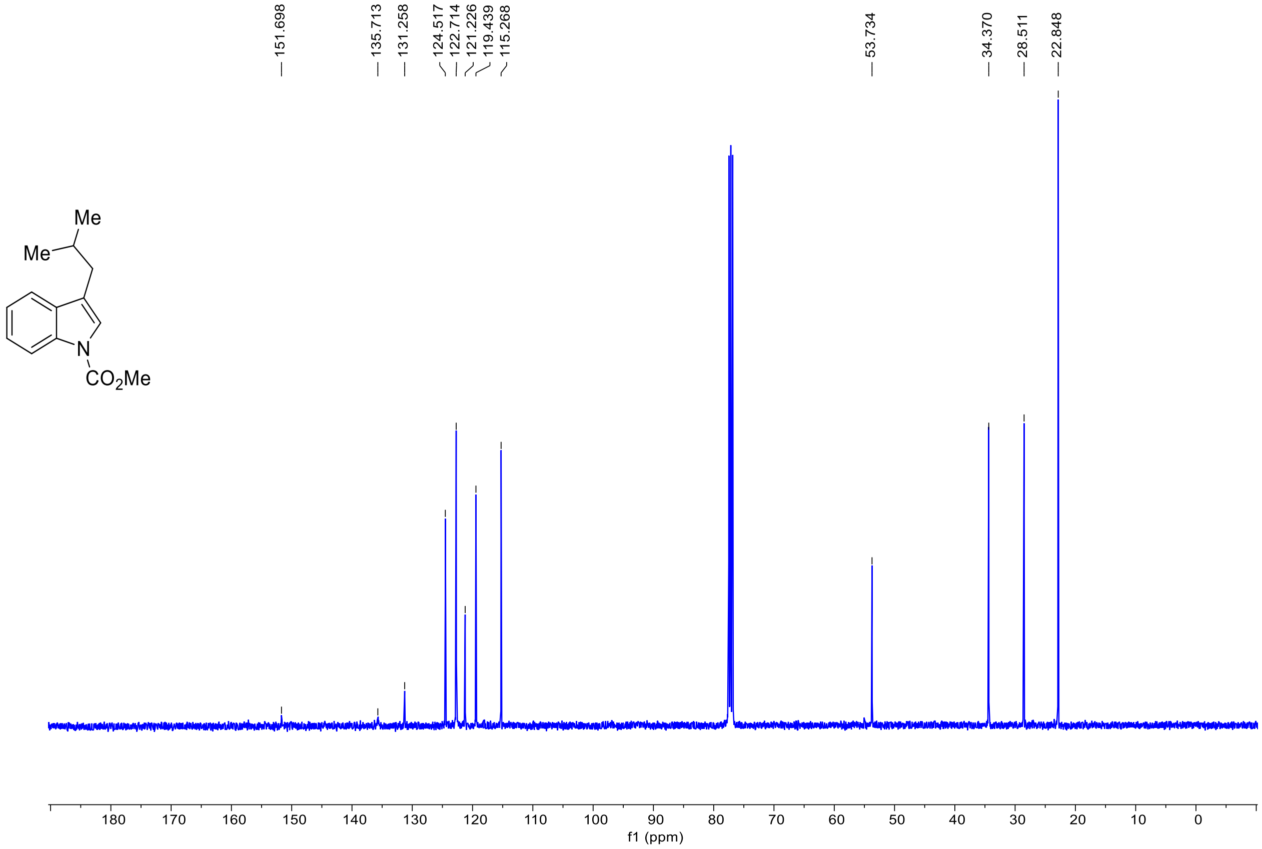

3-Methylbenzofuran-5-yl acetate 4b

^{1}H NMR (300 MHz, $CDCl_3$)

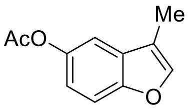

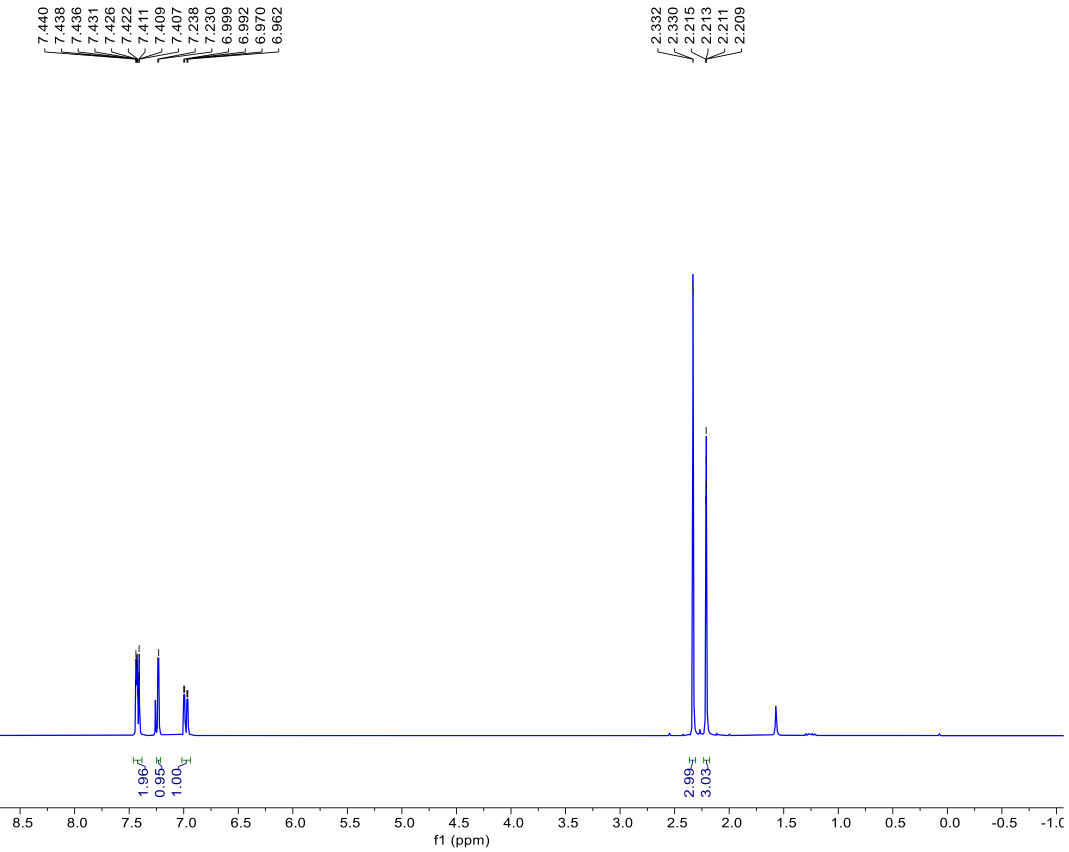

^{13}C NMR (76 MHz, $CDCl_3$)

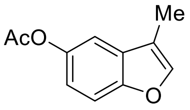

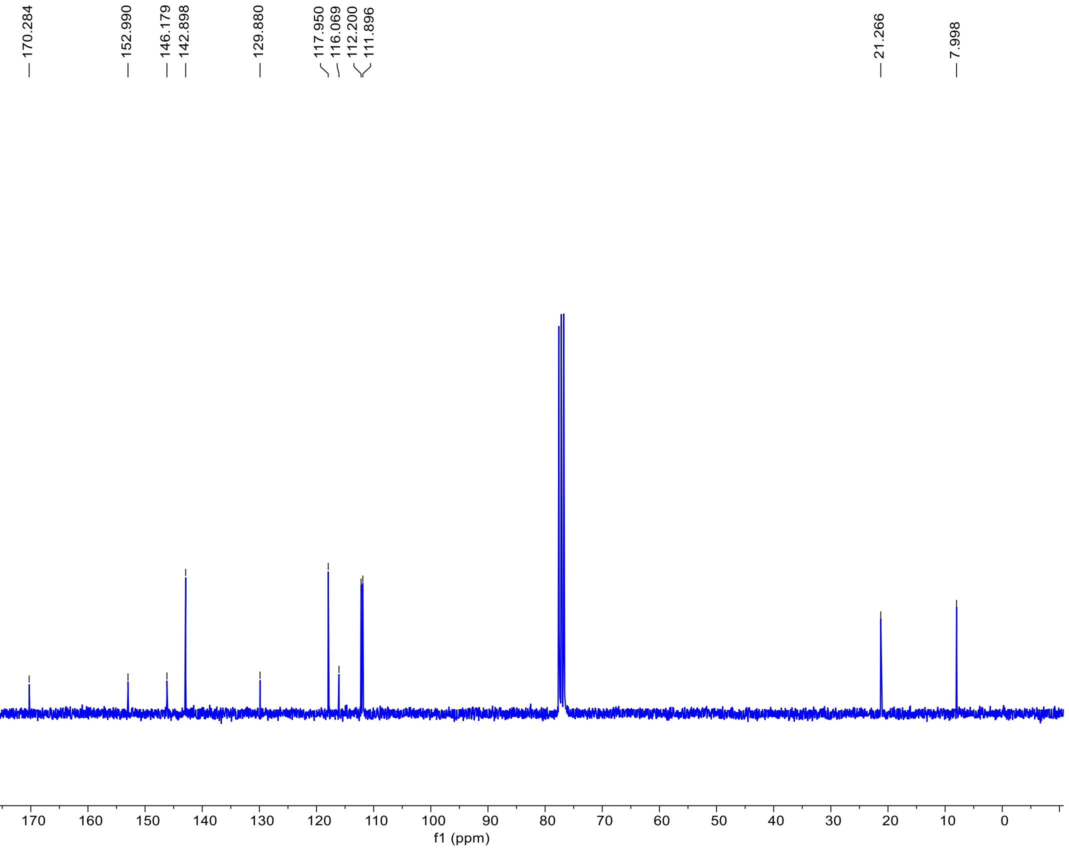

5-(But-2-yn-1-yloxy)-3-methylbenzofuran 4c

^{1}H NMR (300 MHz, $CDCl_3$)

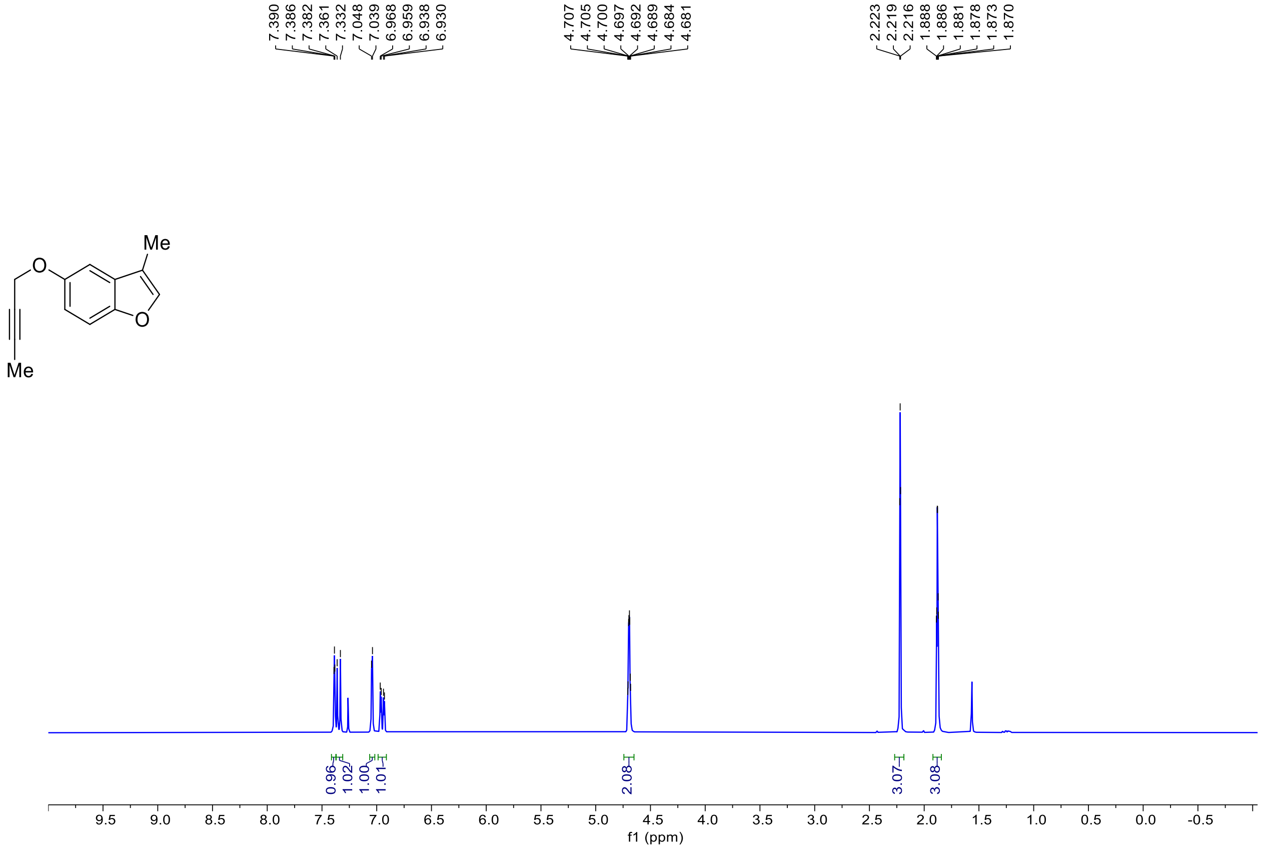

^{13}C NMR (76 MHz, $CDCl_3$)

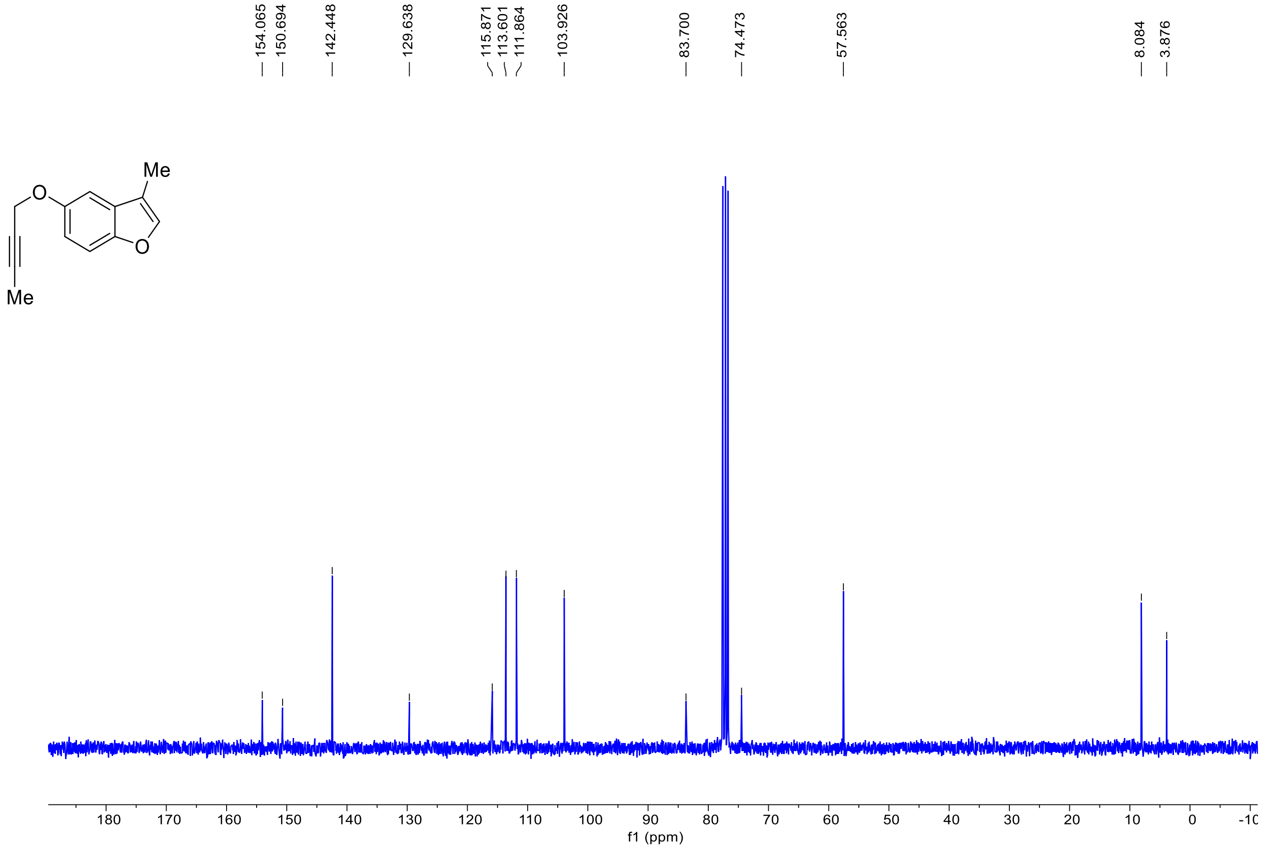

3-Methyl-5-((3-methylbut-2-en-1-yl)oxy)benzofuran 4d

^{1}H NMR (300 MHz, $CDCl_3$)

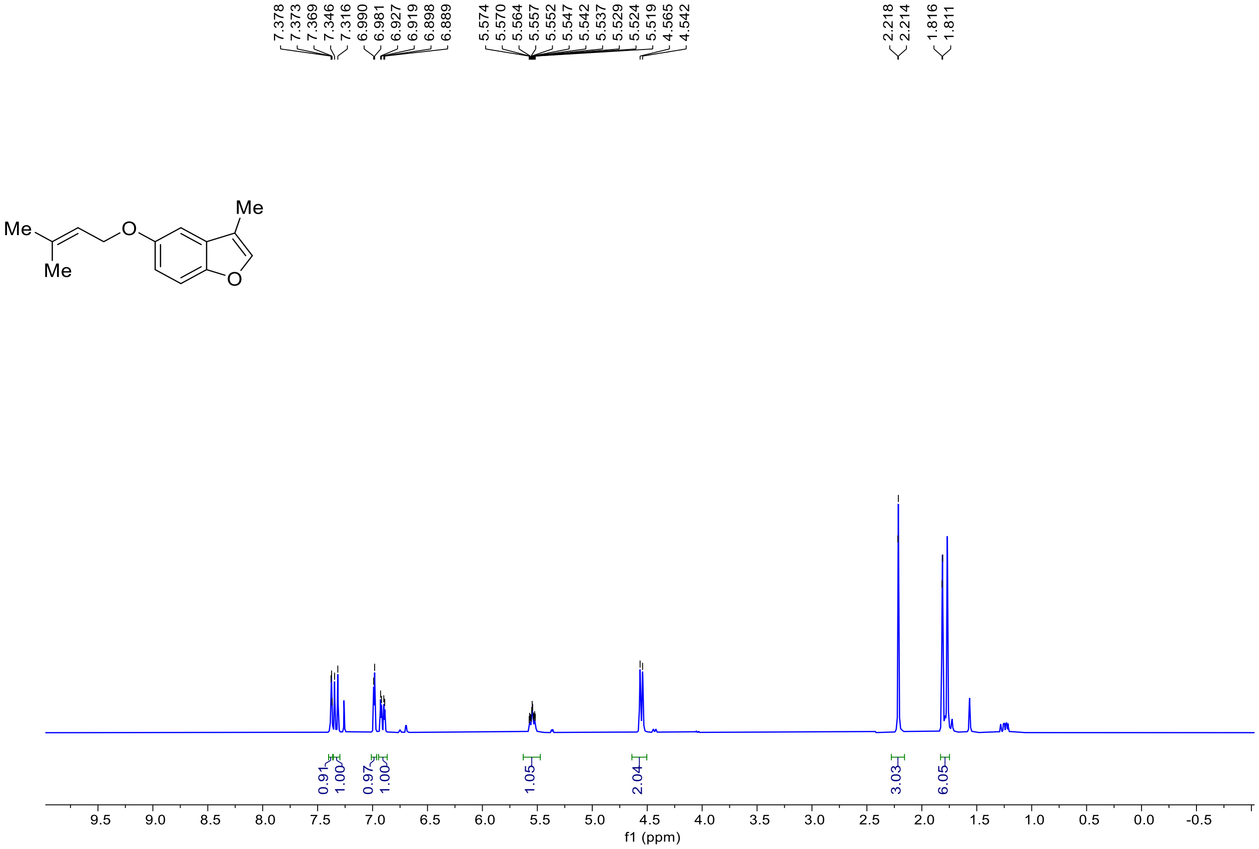

^{13}C NMR (76 MHz, $CDCl_3$)

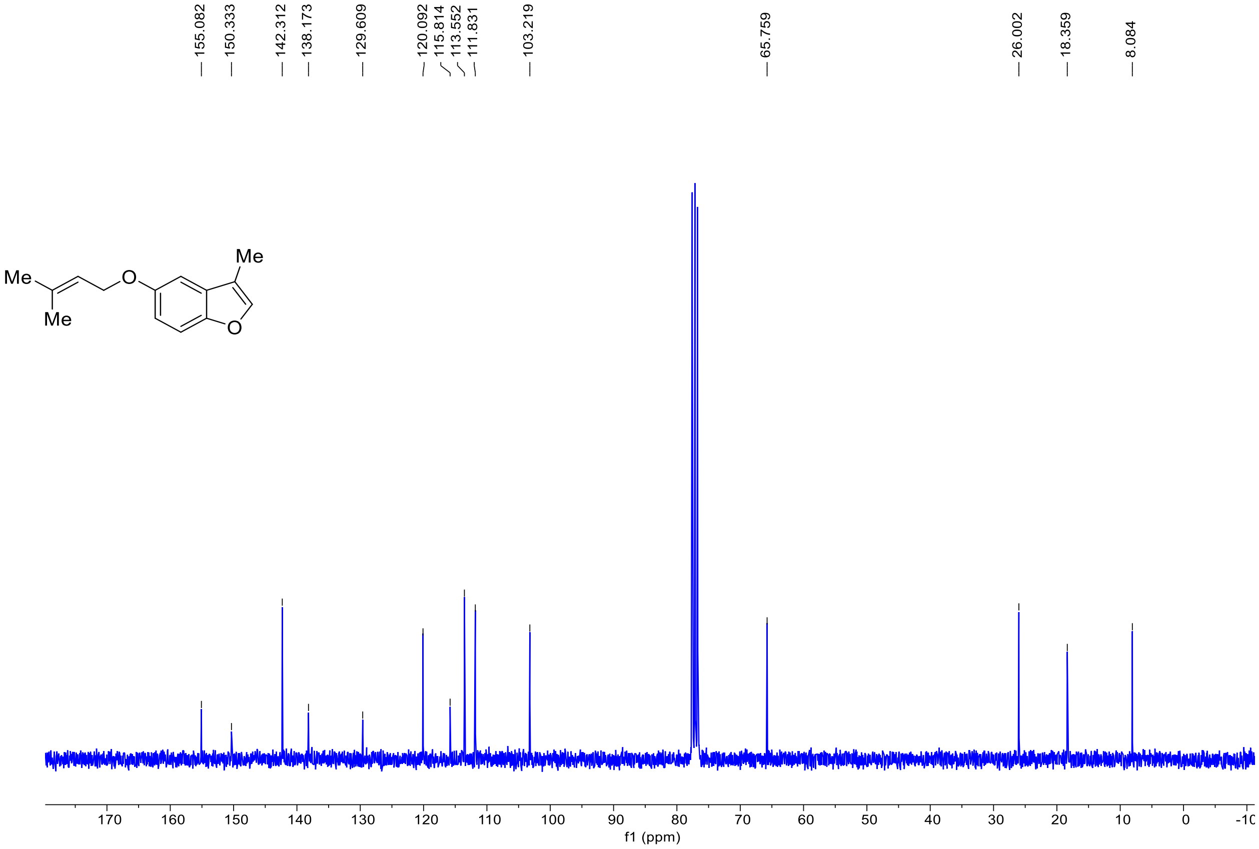

3-Methylbenzofuran-5-yl trifluoromethanesulfonate 4e

^{1}H NMR (400 MHz, $CDCl_3$)

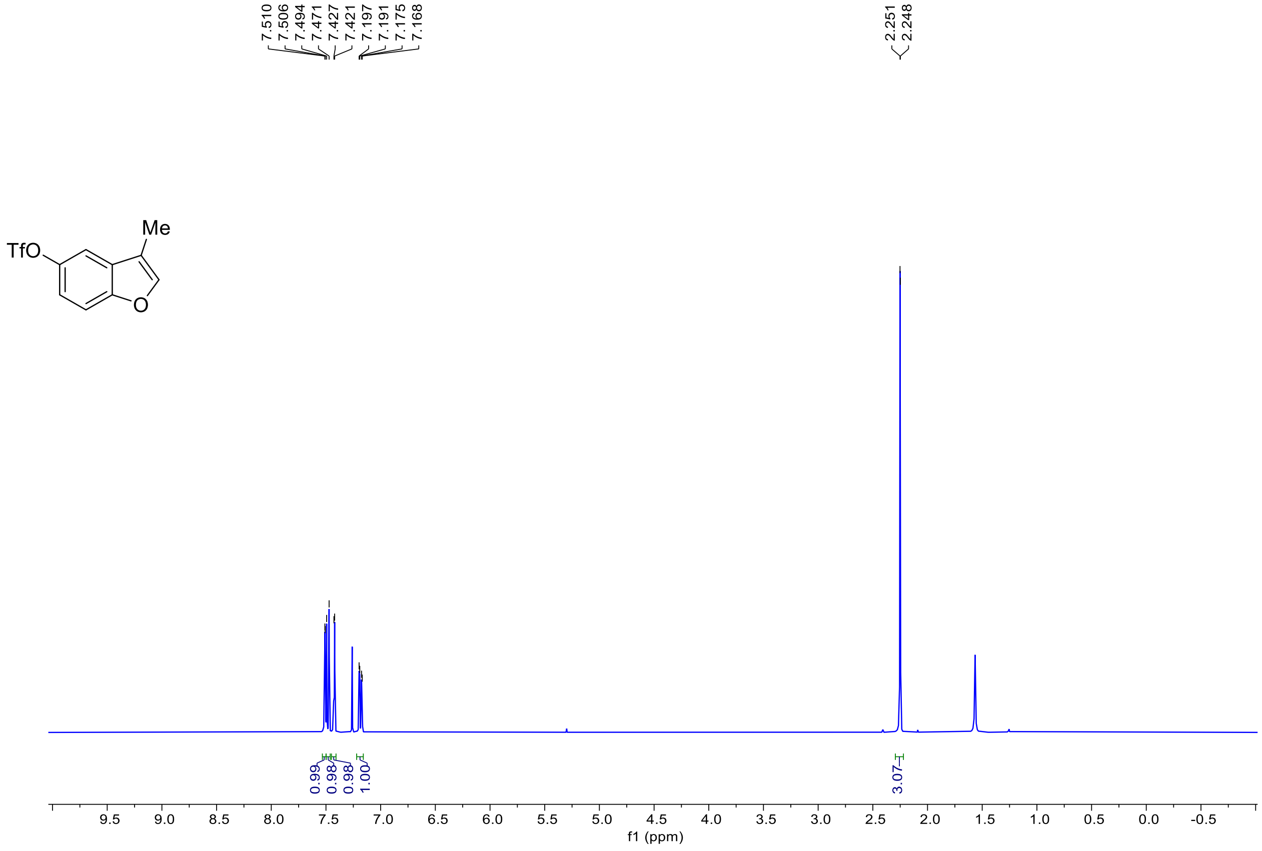

^{13}C NMR (101 MHz, $CDCl_3$)

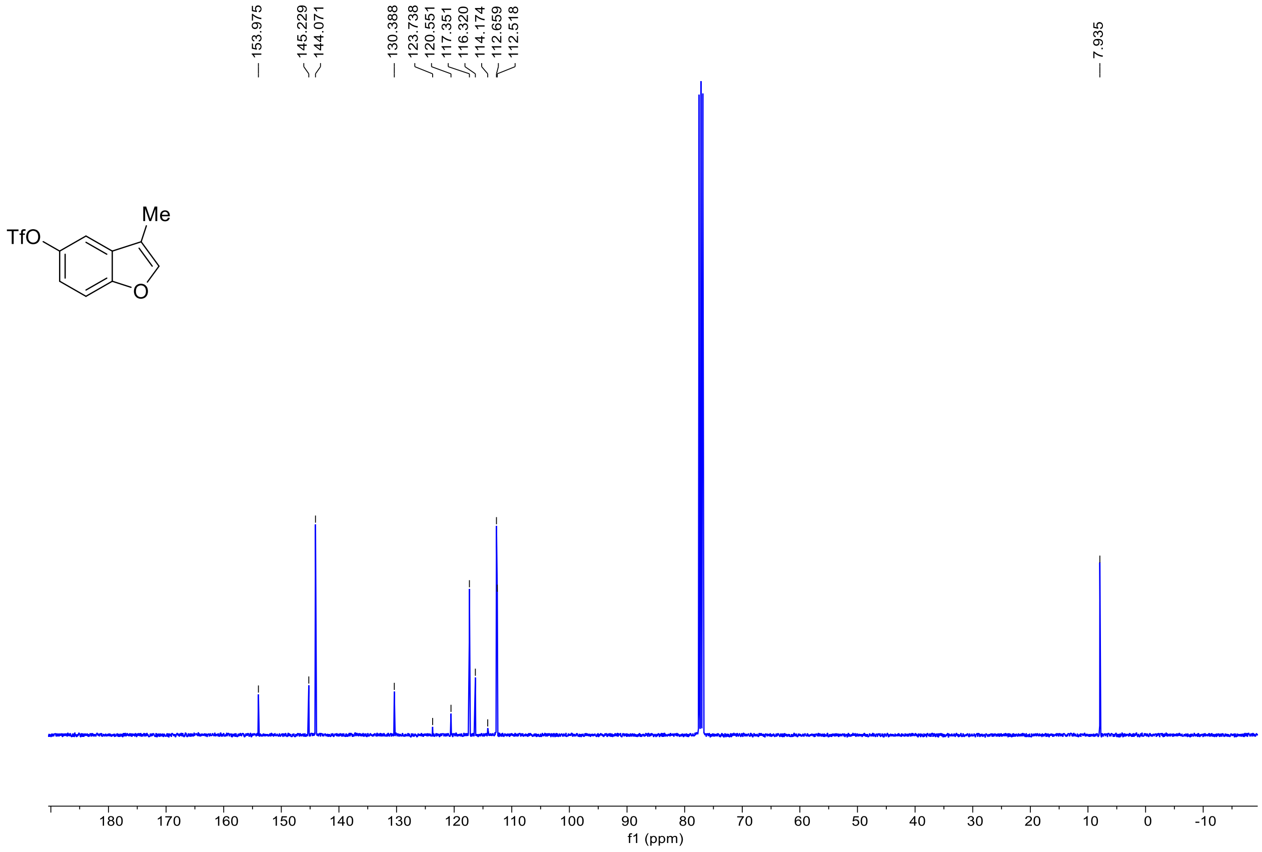

^{19}F NMR (376 MHz, $CDCl_3$)

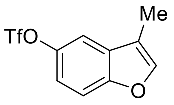

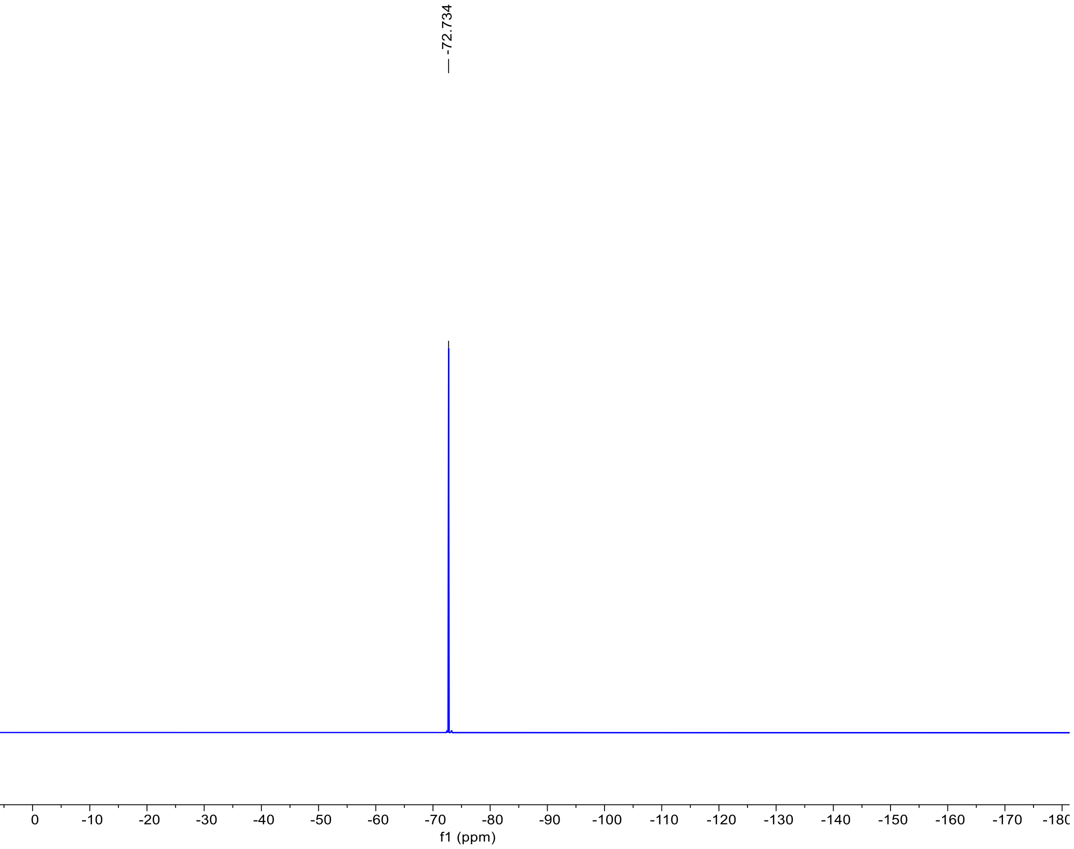

N-(2-(benzofuran-3-yl)ethyl)-2,2,2-trifluoroacetamide 4g

^{1}H NMR (400 MHz, $CDCl_3$)

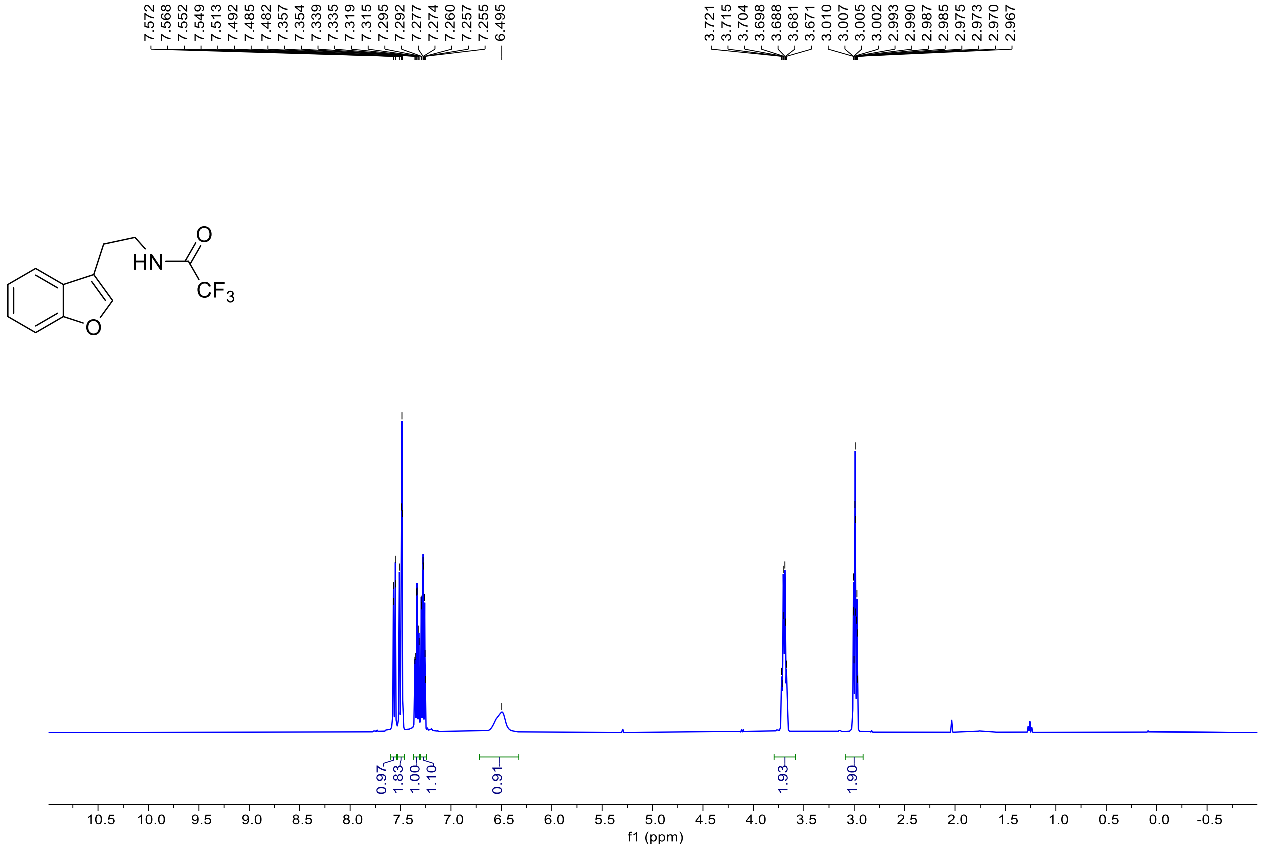

^{13}C NMR (76 MHz, $CDCl_3$)

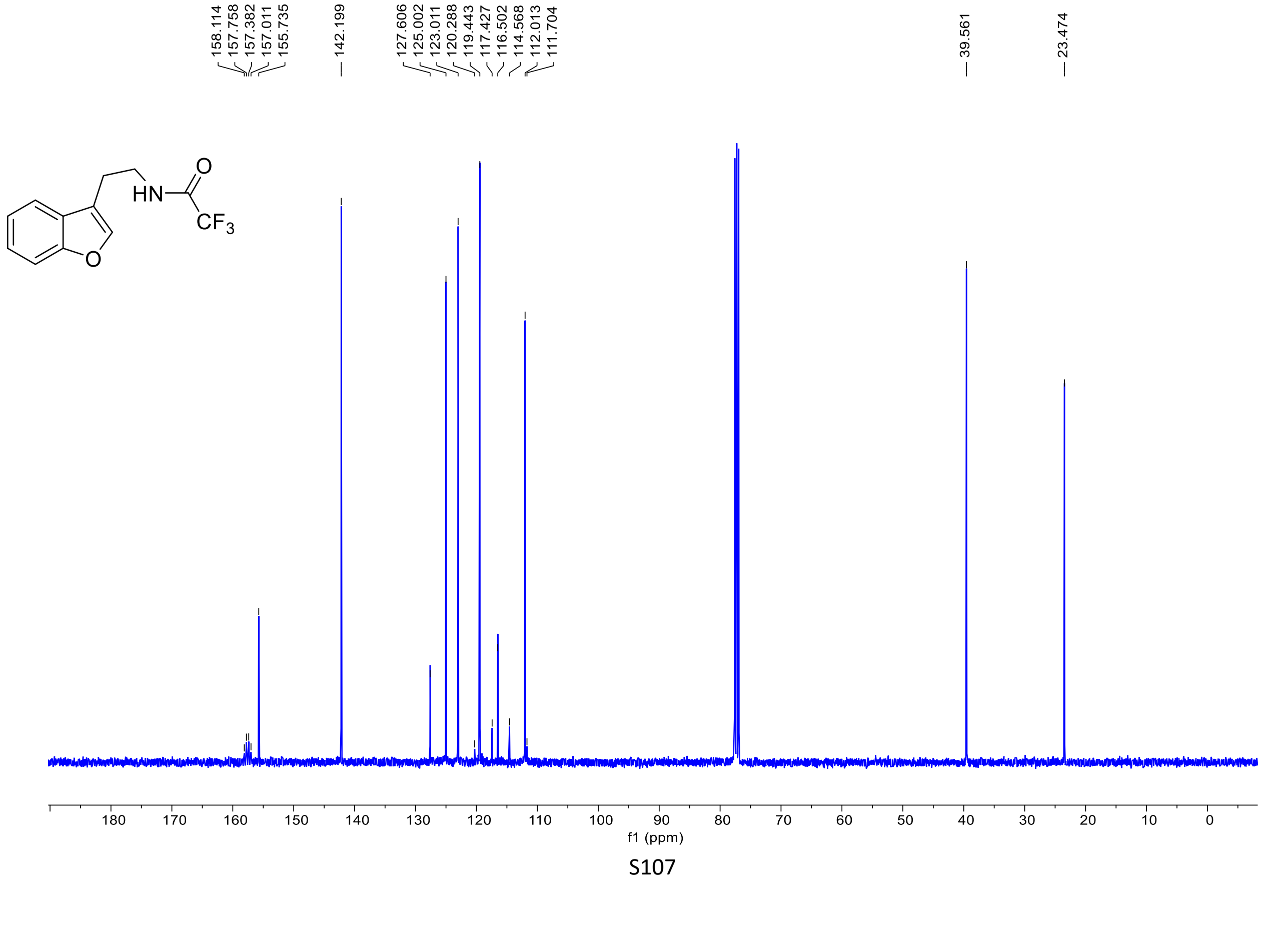

^{19}F NMR (282 MHz, $CDCl_3$)

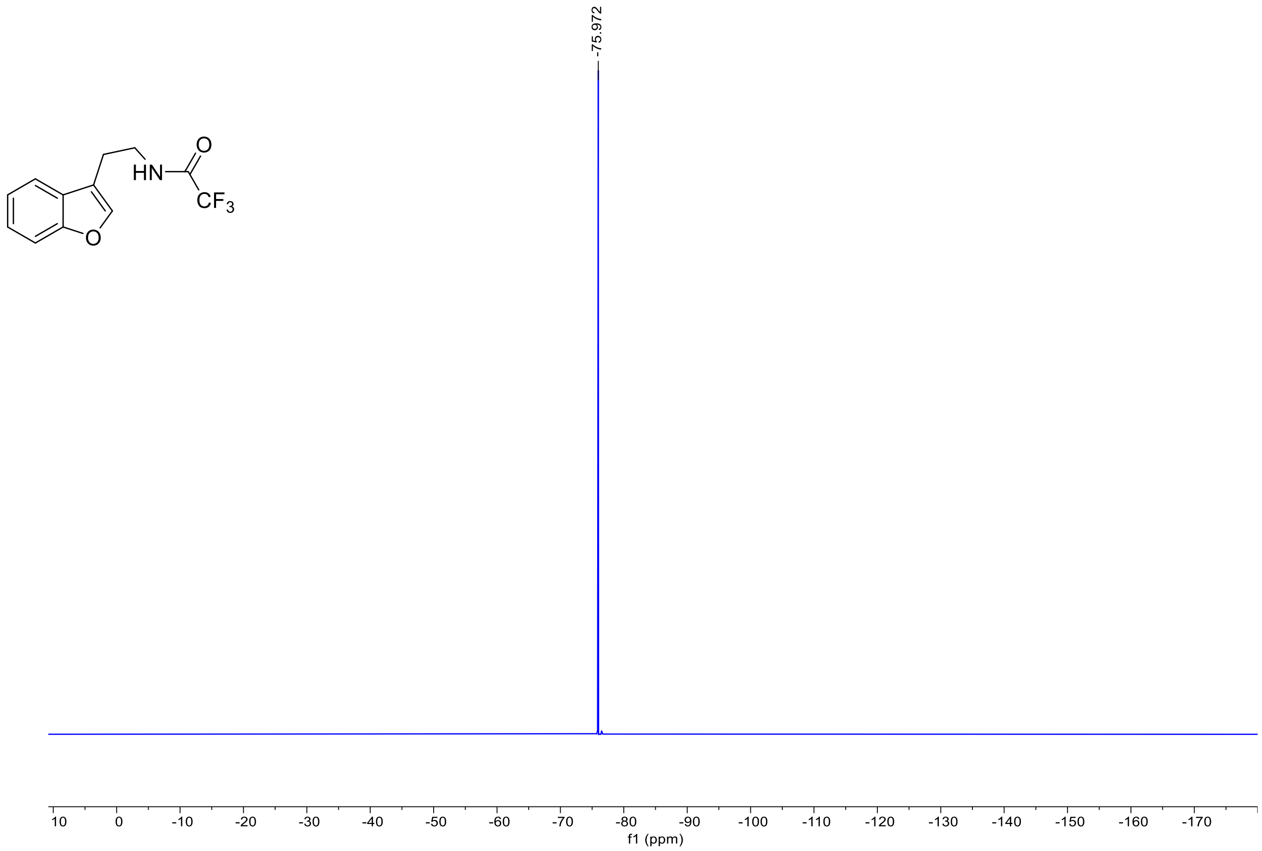

(Benzofuran-3-ylmethoxy)triisopropylsilane 4h

^{1}H NMR (400 MHz, $CDCl_3$)

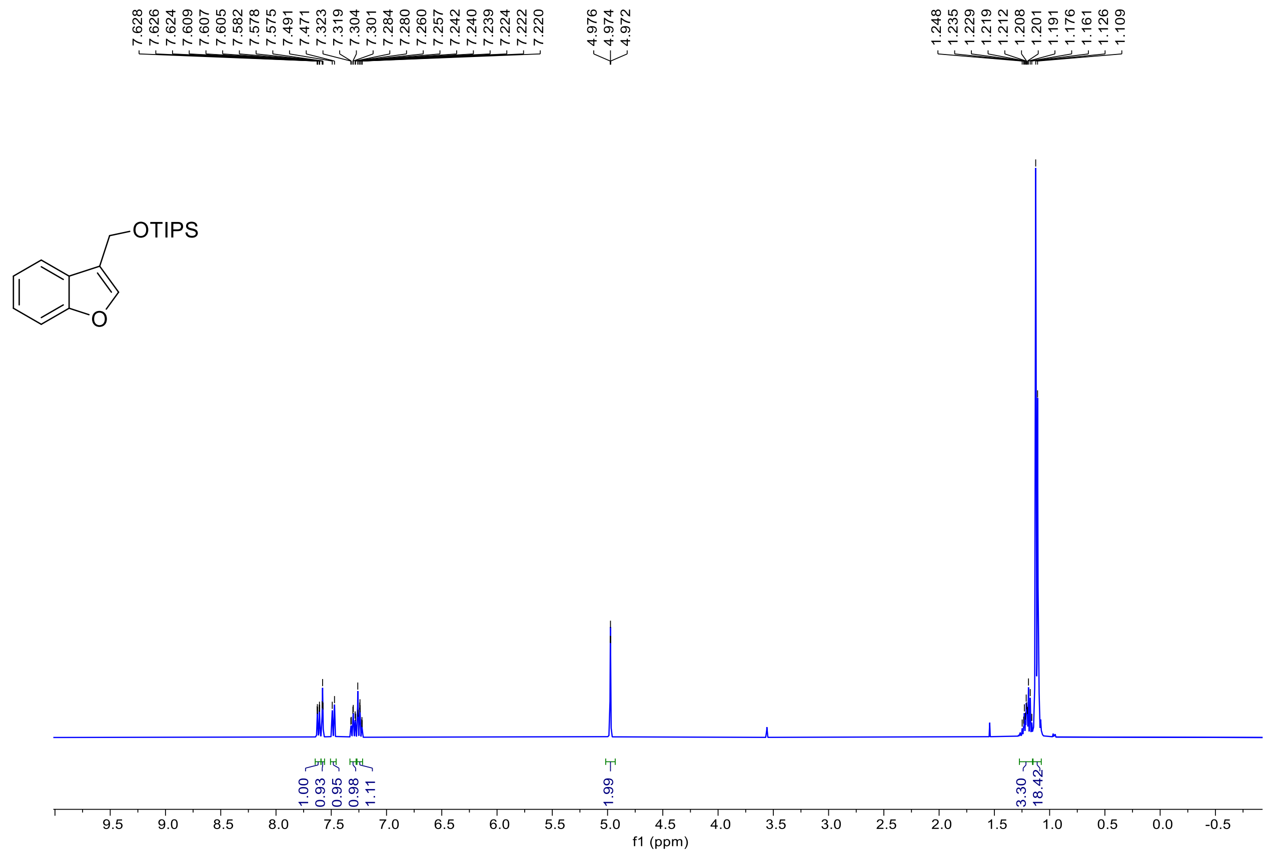

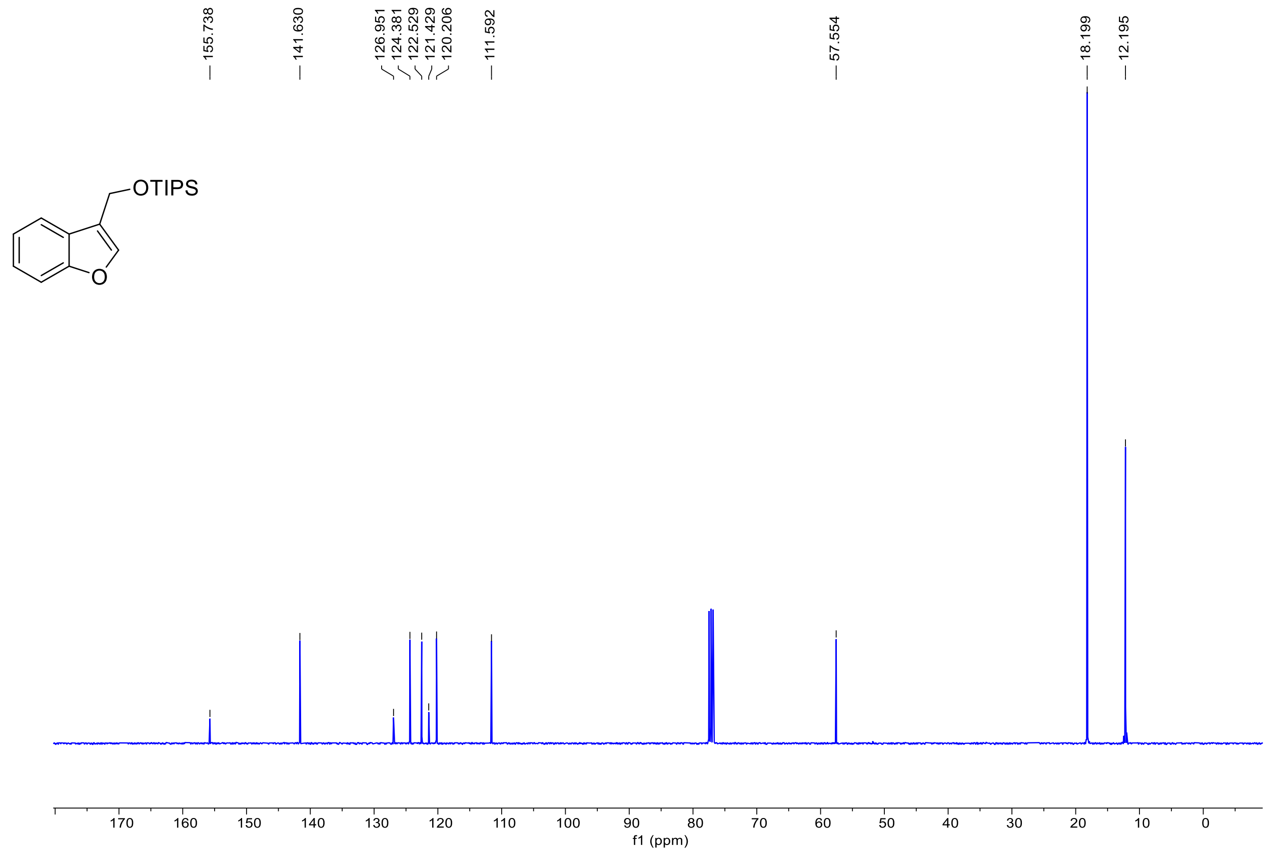

Methyl 3-(3-((2-(benzofuran-3-yl)ethyl)amino)-3-oxopropyl)-1*H*-indole-1-carboxylate 4k

^{1}H NMR (400 MHz, $CDCl_3$)

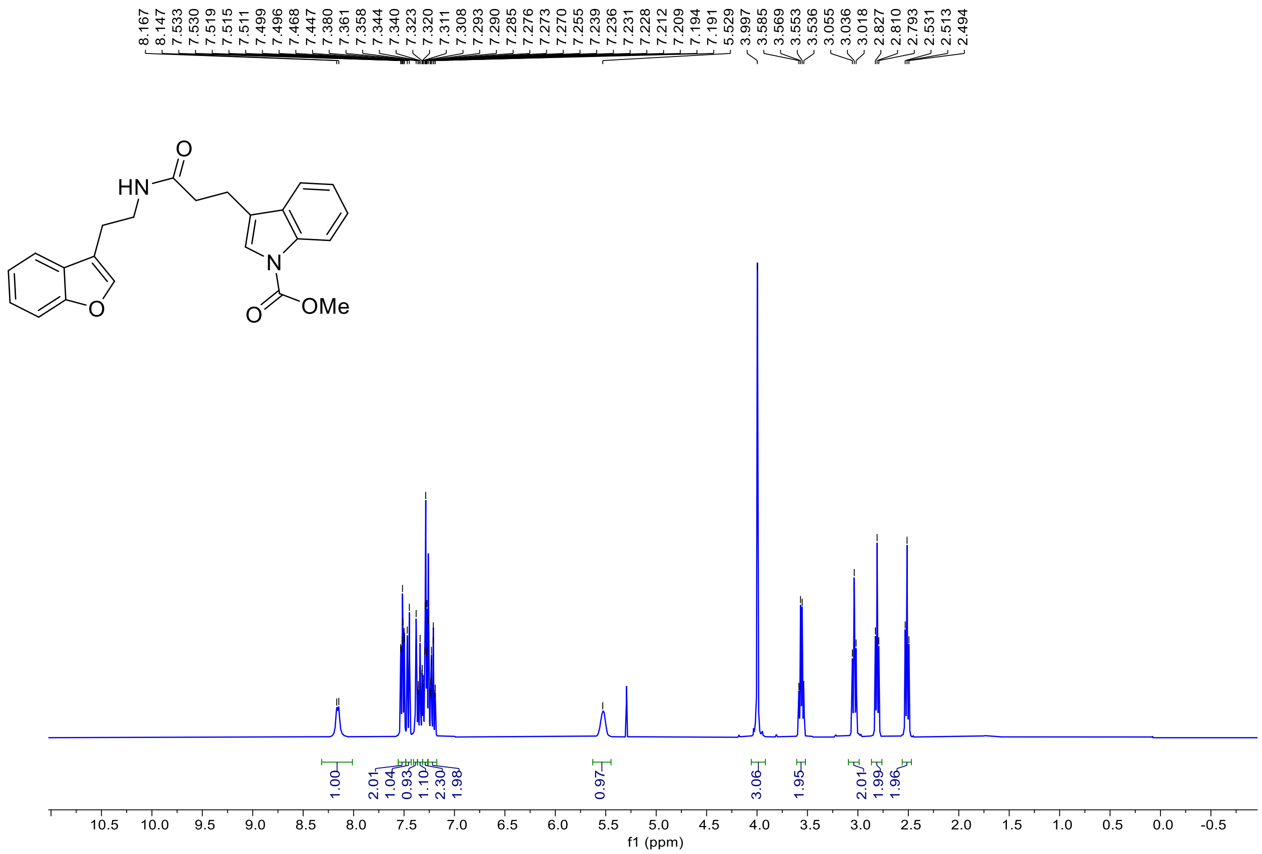

^{13}C NMR (101 MHz, $CDCl_3$)

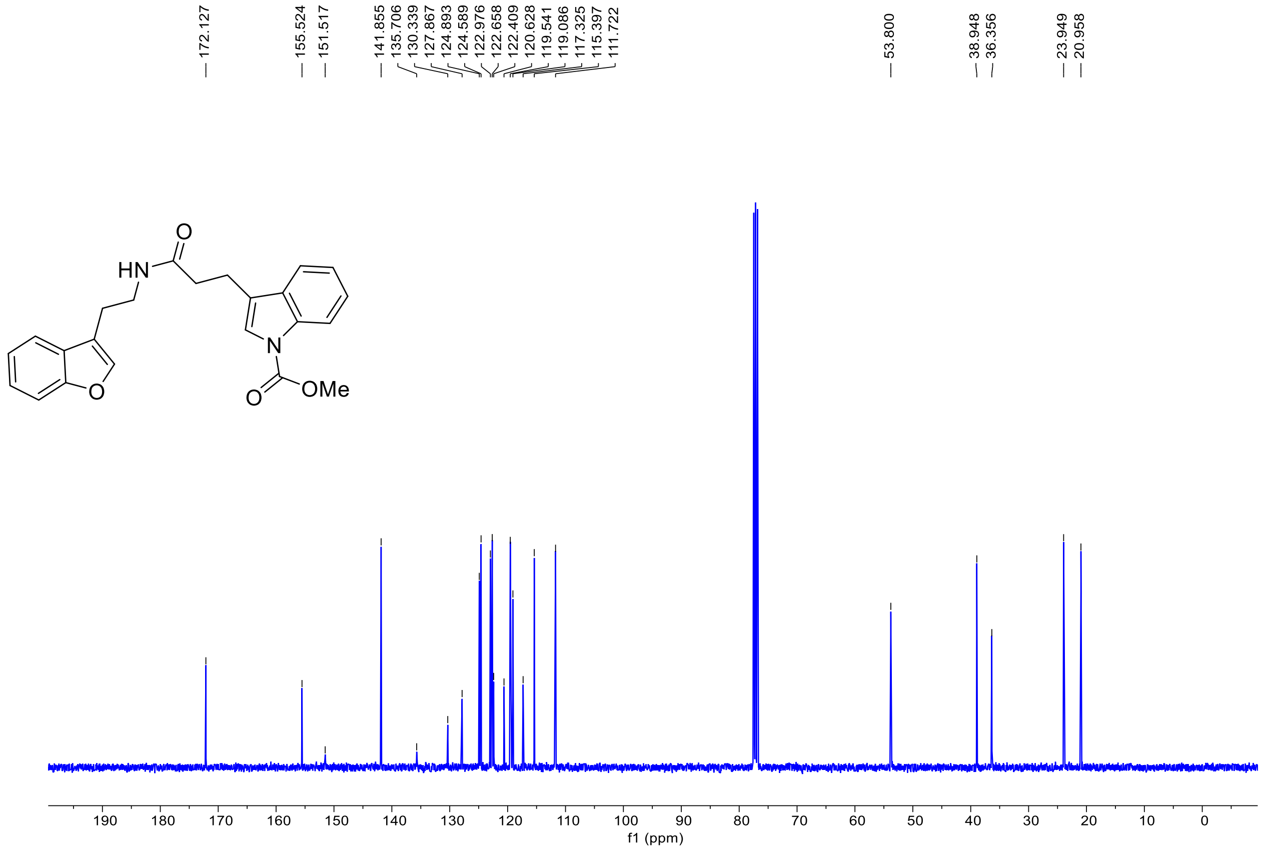

Dimethyl 3-methyl-1*H*-indole-1,5-dicarboxylate Moc-**1ad**

^{1}H NMR (400 MHz, $CDCl_3$)

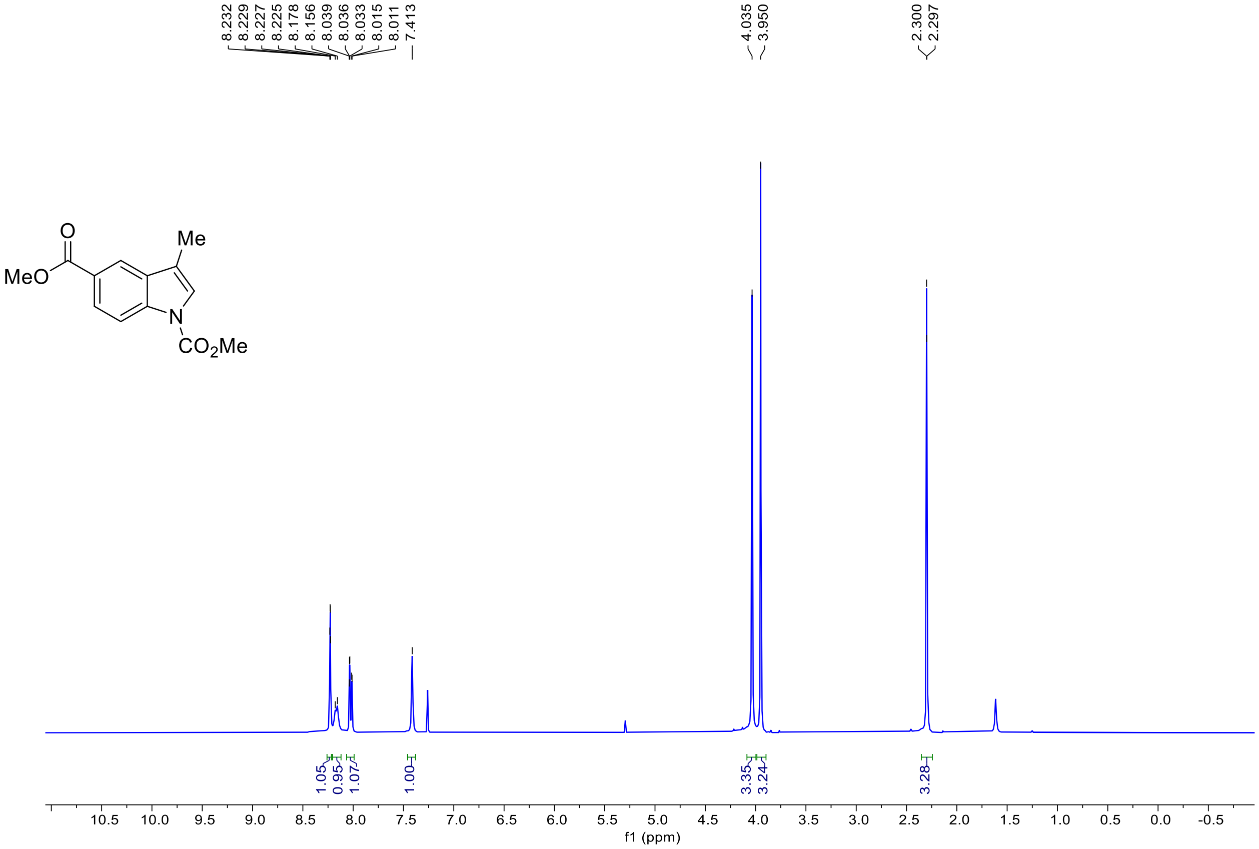

^{13}C NMR (101 MHz, $CDCl_3$)

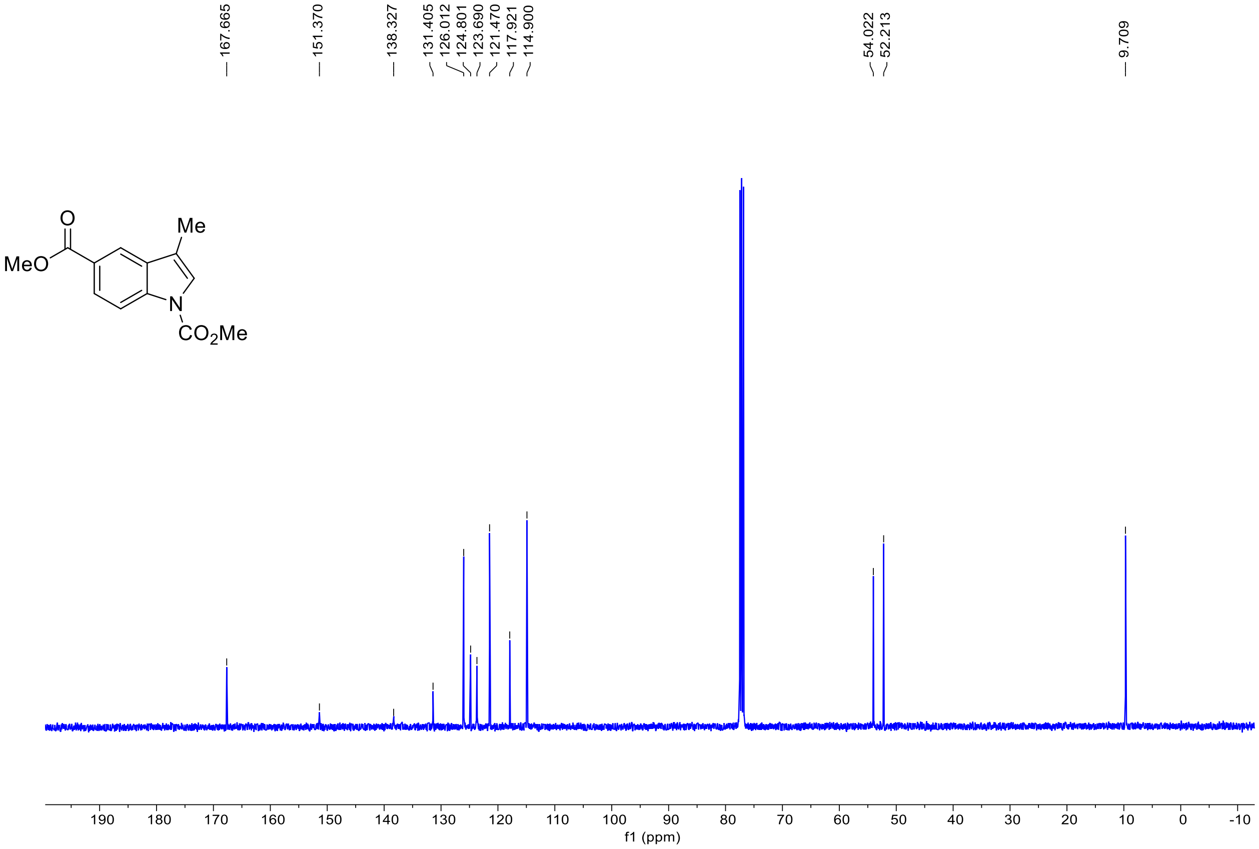

3-(1*H*-indol-3-yl)propyl (*S*)-2-(6-methoxynaphthalen-2-yl)propanoate 1r

^{1}H NMR (400 MHz, $CDCl_3$)

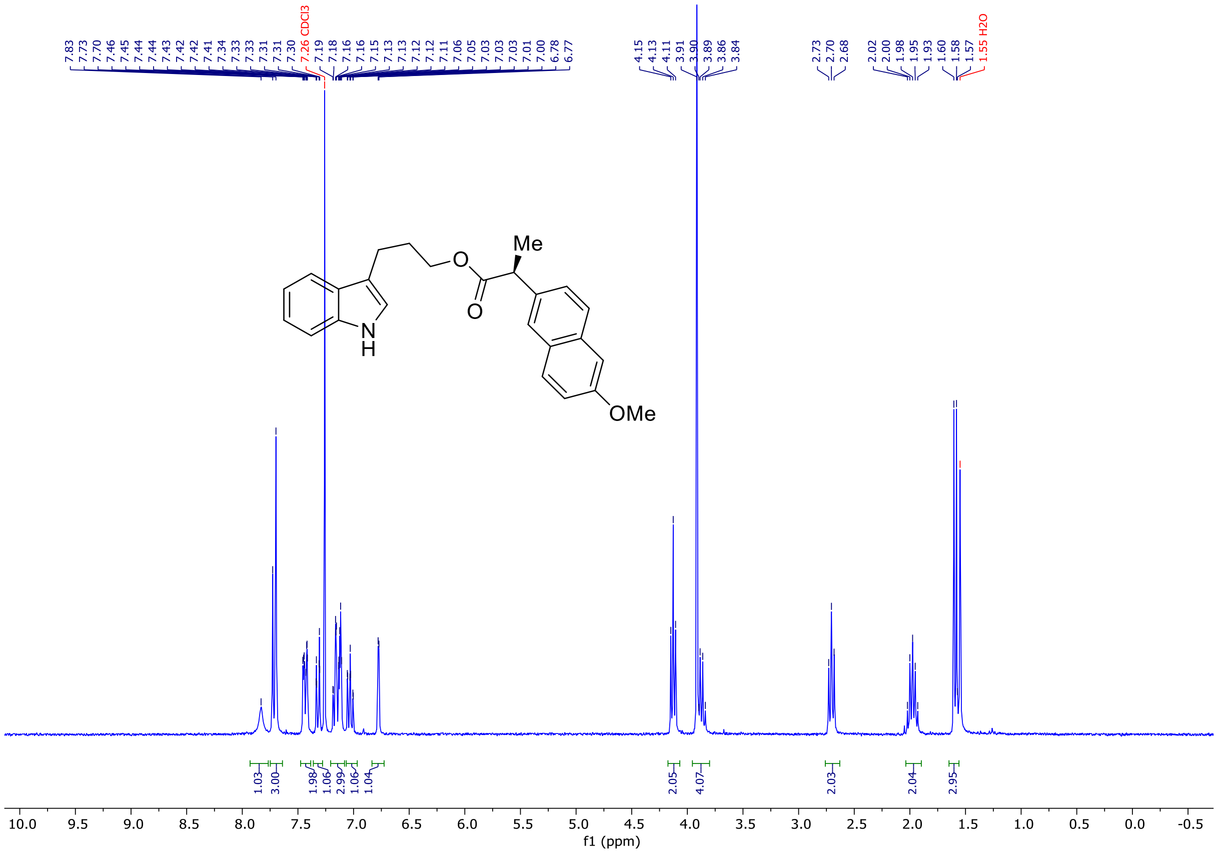

^{13}C NMR (101 MHz, $CDCl_3$)

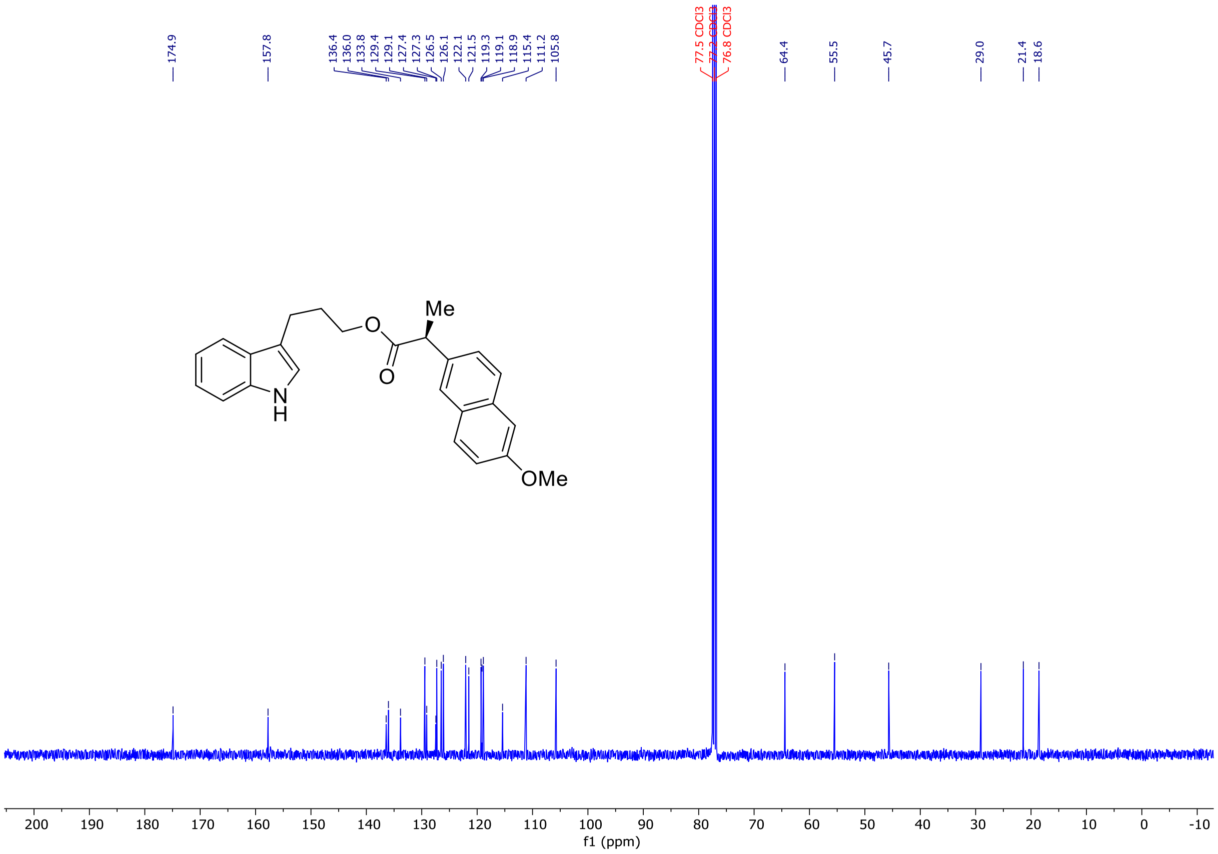

Methyl (*S*)-3-(3-((2-(6-methoxynaphthalen-2-yl)propanoyl)oxy)propyl)-1*H*-indole-1-carboxylate Moc-**1r**

^{1}H NMR (400 MHz, $CDCl_3$)

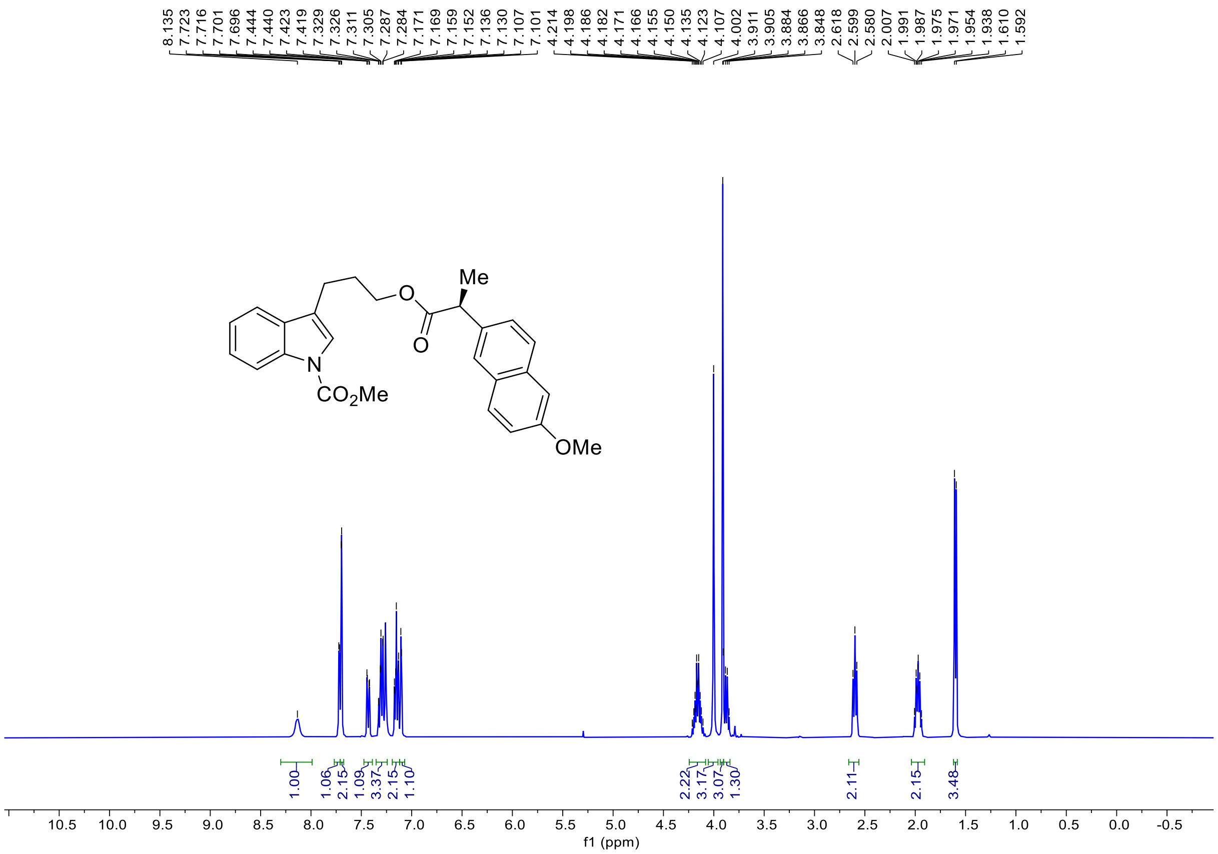

^{13}C NMR (101 MHz, $CDCl_3$)

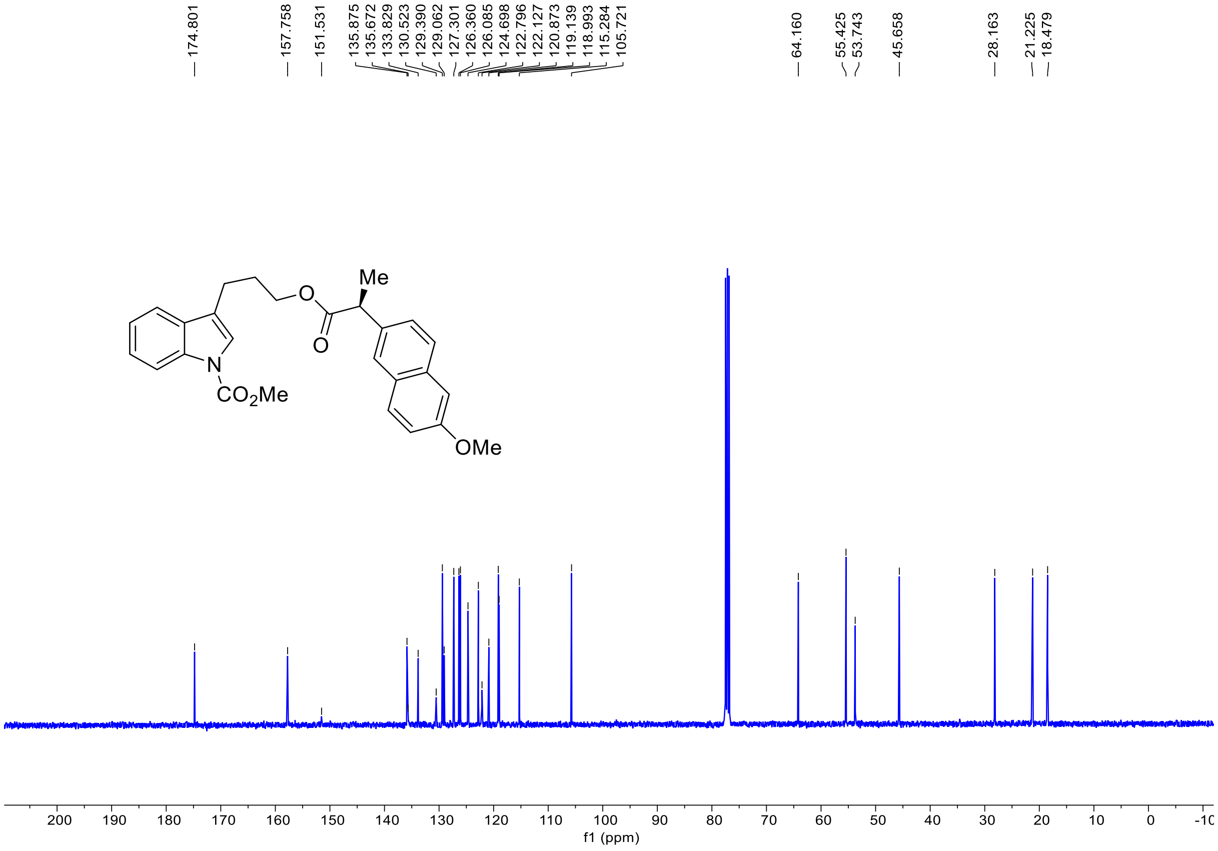

Methyl 3-(3-methoxy-3-oxopropyl)-1*H*-indole-1-carboxylate Moc-**1s**

^{1}H NMR (400 MHz, $CDCl_3$)

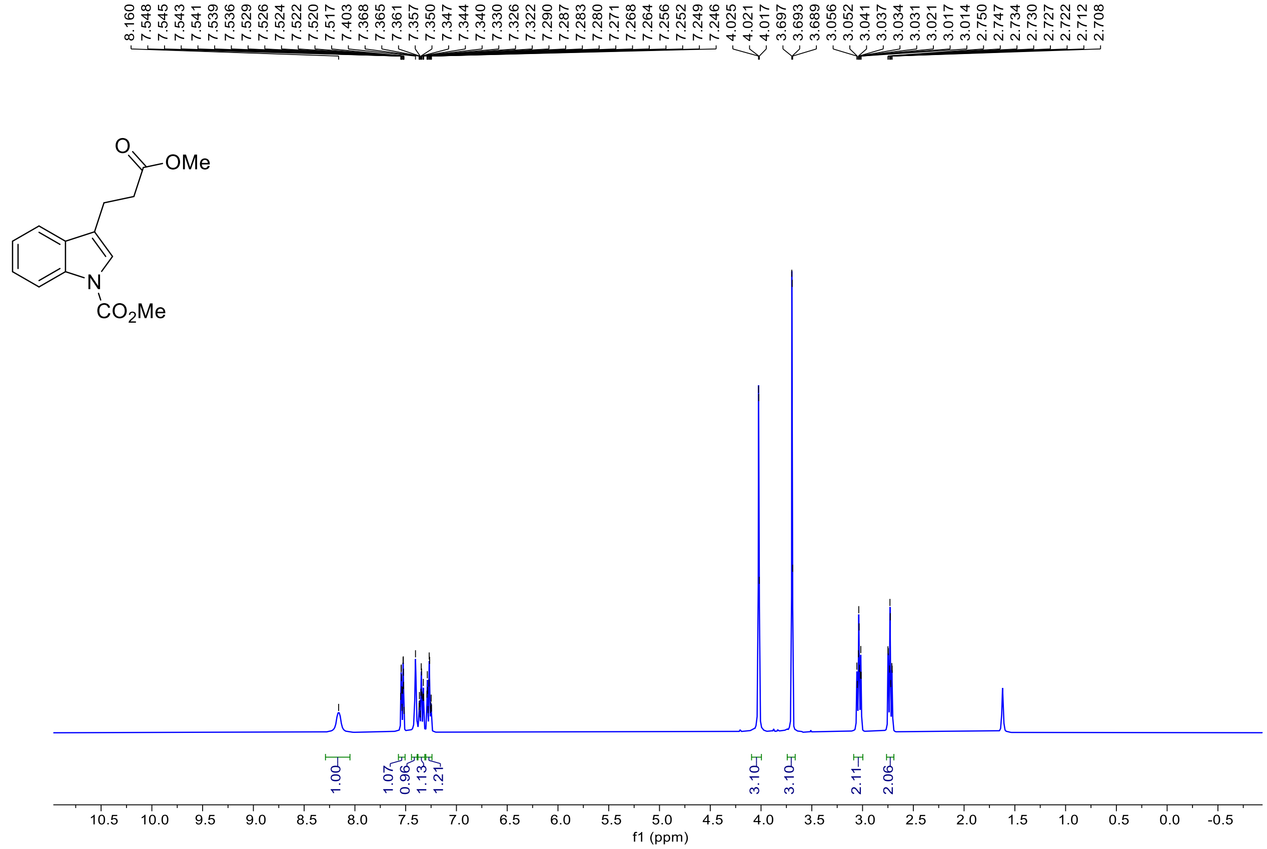

^{13}C NMR (101 MHz, $CDCl_3$)

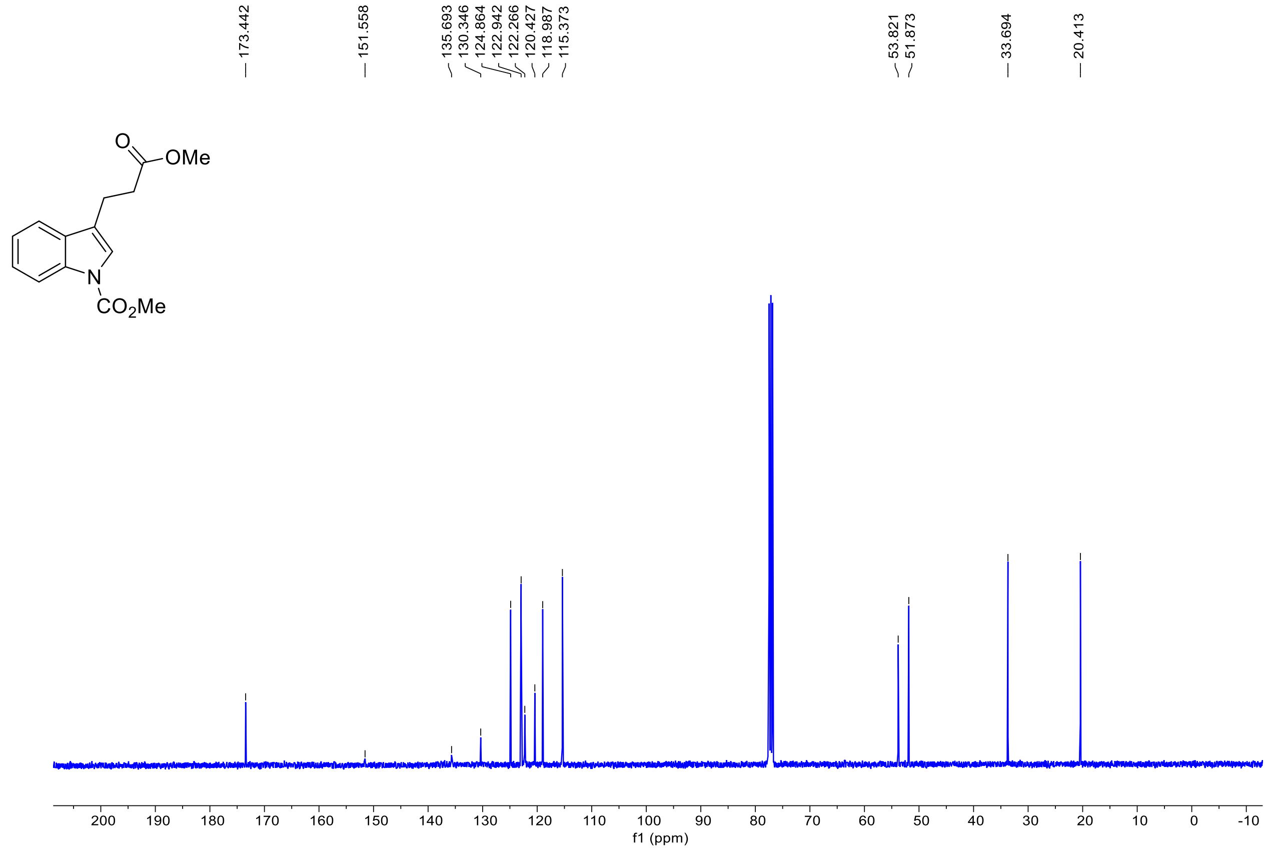

3-(1H-indol-3-yl)propyl 2-(3-cyano-4-isobutoxyphenyl)-4-methylthiazole-5-carboxylate 1t

^{1}H NMR (300 MHz, $CDCl_3$)

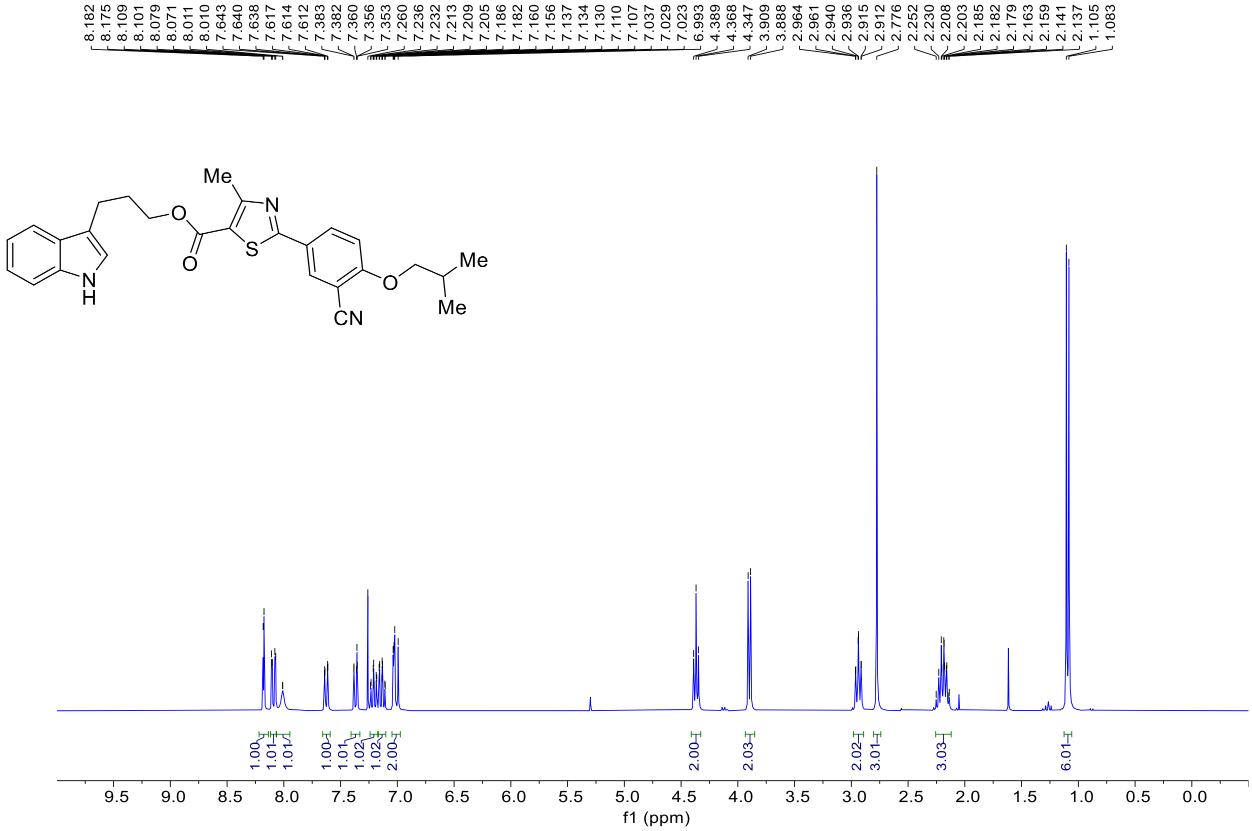

^{13}C NMR (76 MHz, $CDCl_3$)

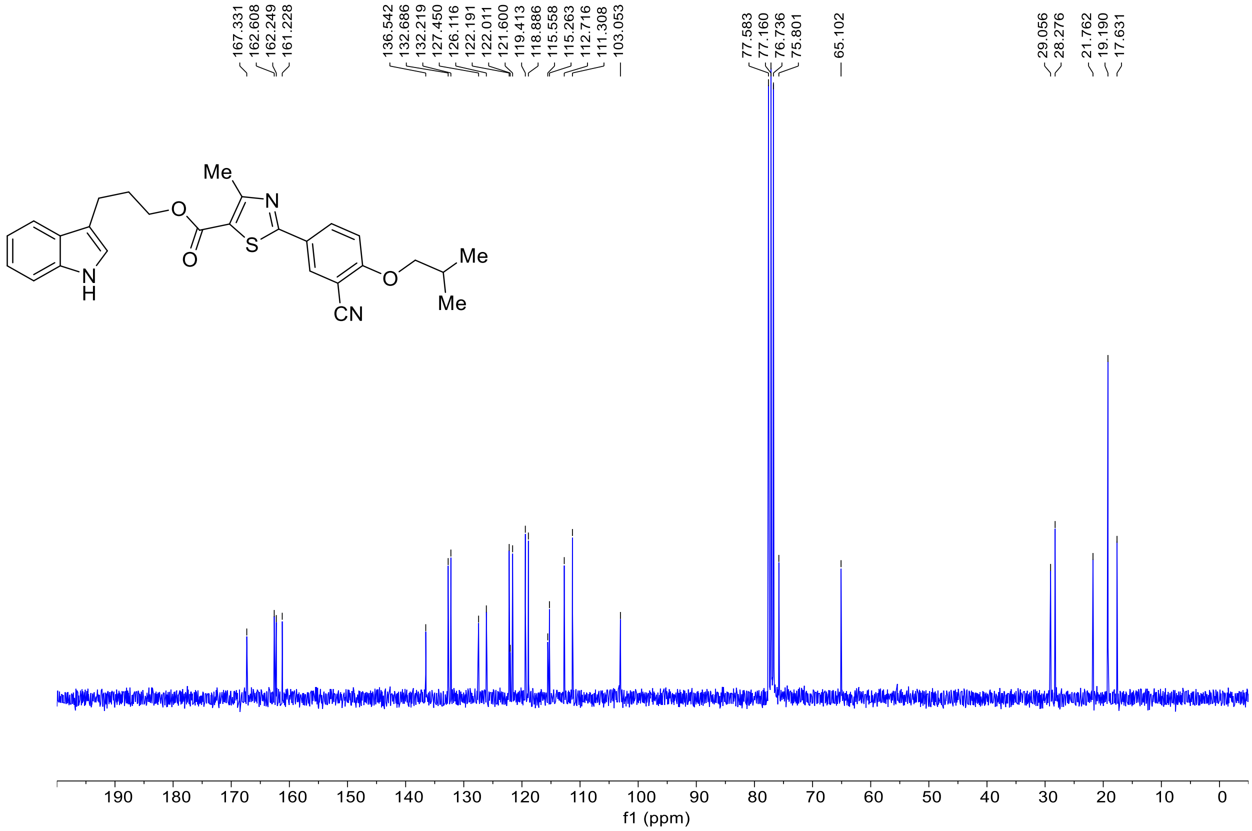

3-(1-(methoxycarbonyl)-1*H*-indol-3-yl)propyl 2-(3-cyano-4-isobutoxyphenyl)-4-methylthiazole-5-carboxylate

Moc-**1t** **^{1}H NMR** (300 MHz, $CDCl_3$)

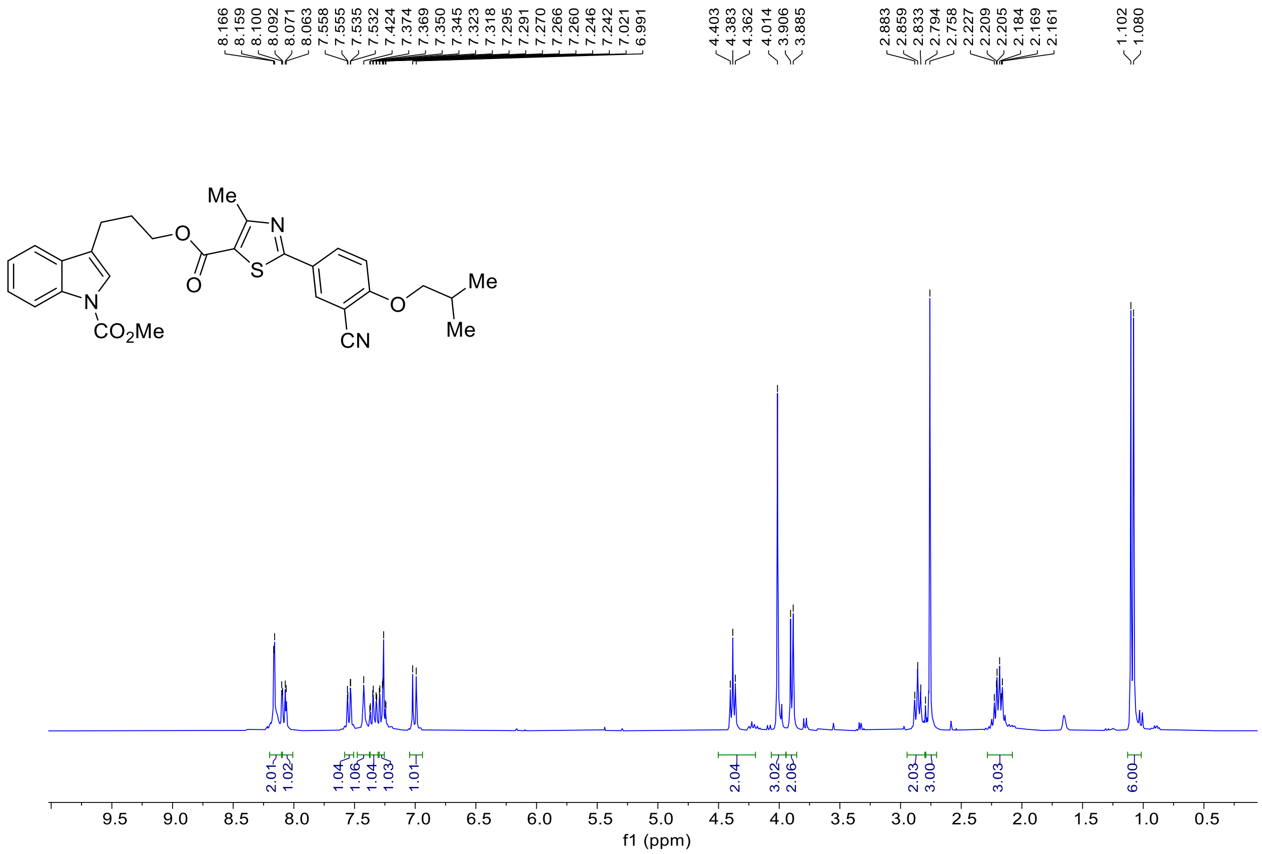

^{13}C NMR (76 MHz, $CDCl_3$)

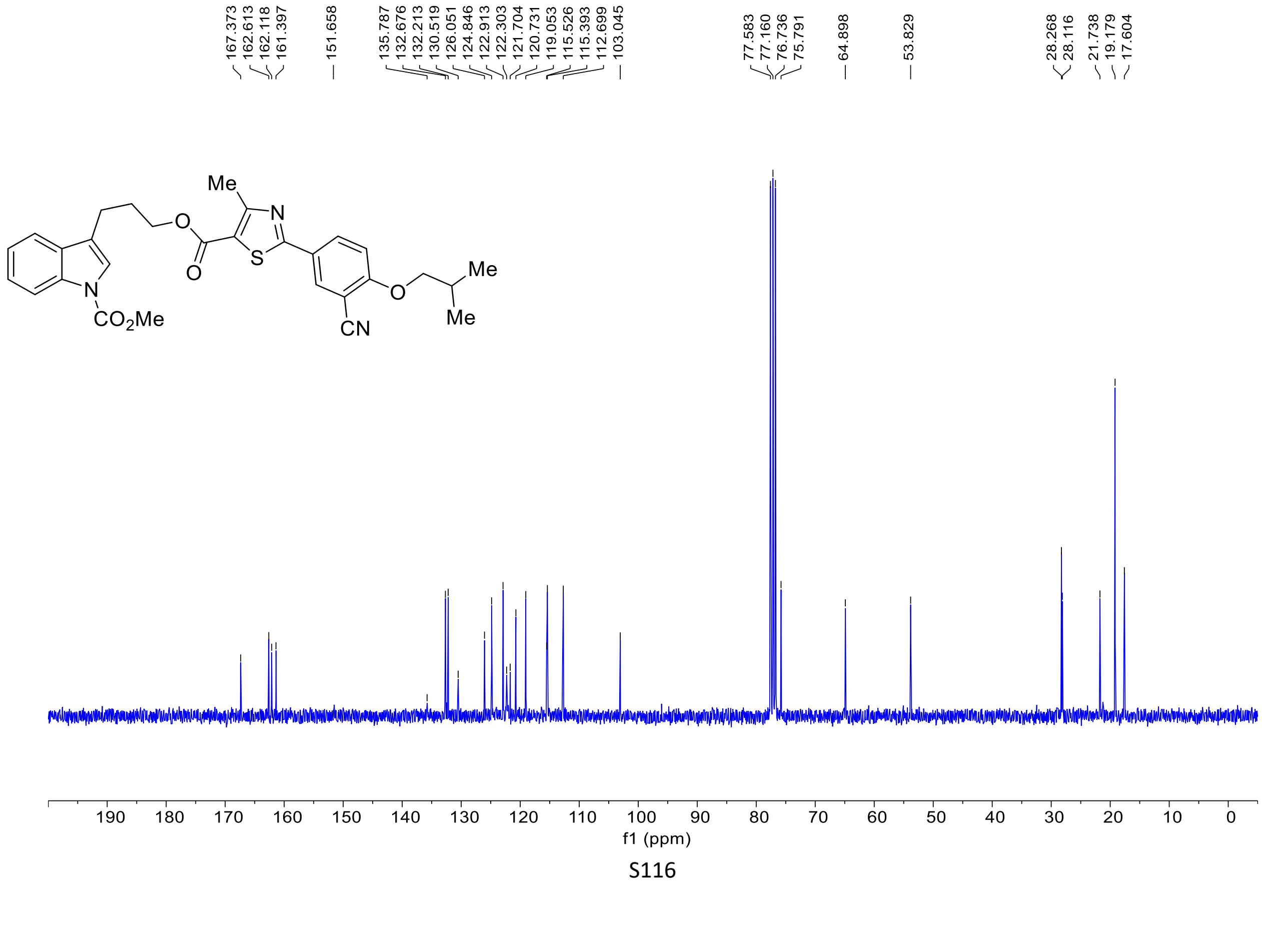

Methyl (2,2,2-trifluoroacetyl)-*L*-tryptophyl-*L*-phenylalaninate 1v

^{1}H NMR (400 MHz, DMSO)

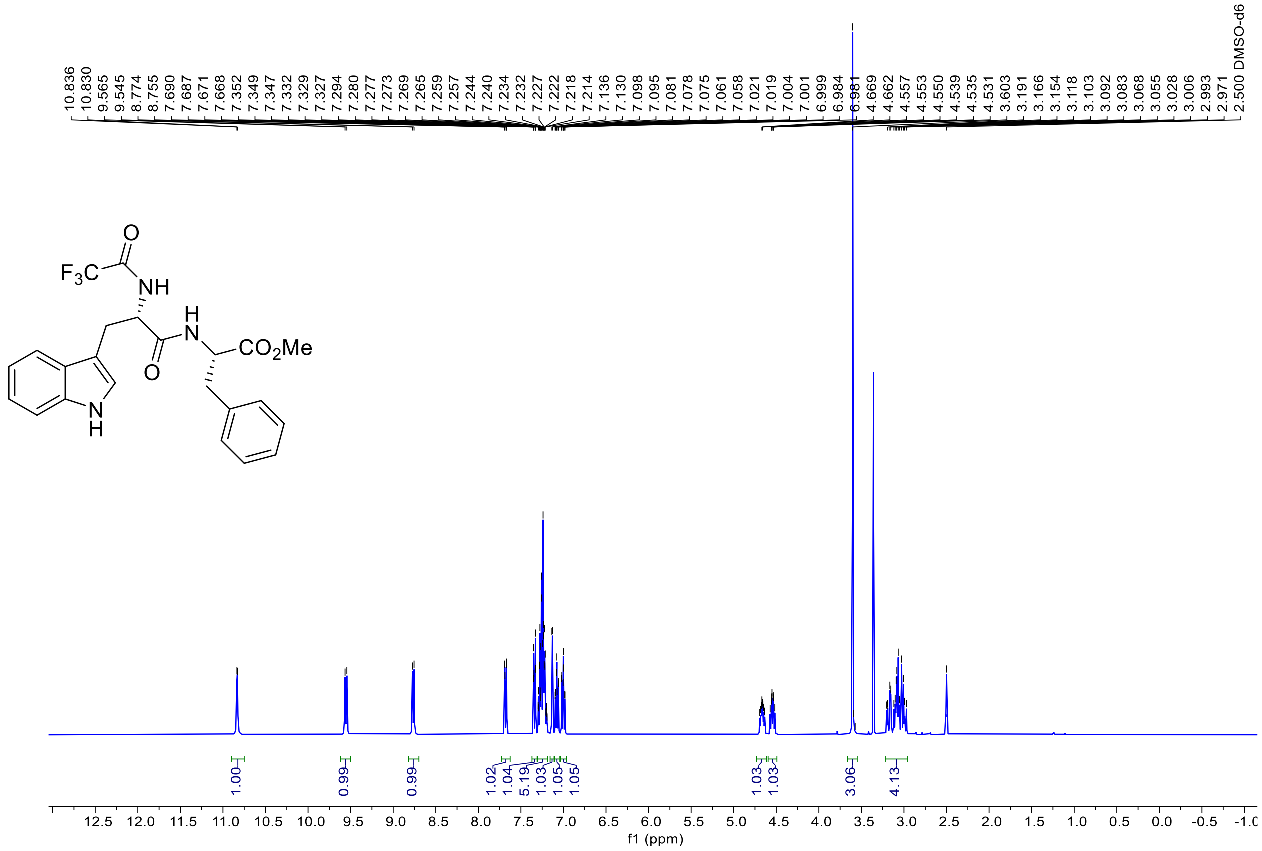

^{13}C NMR (101 MHz, DMSO)

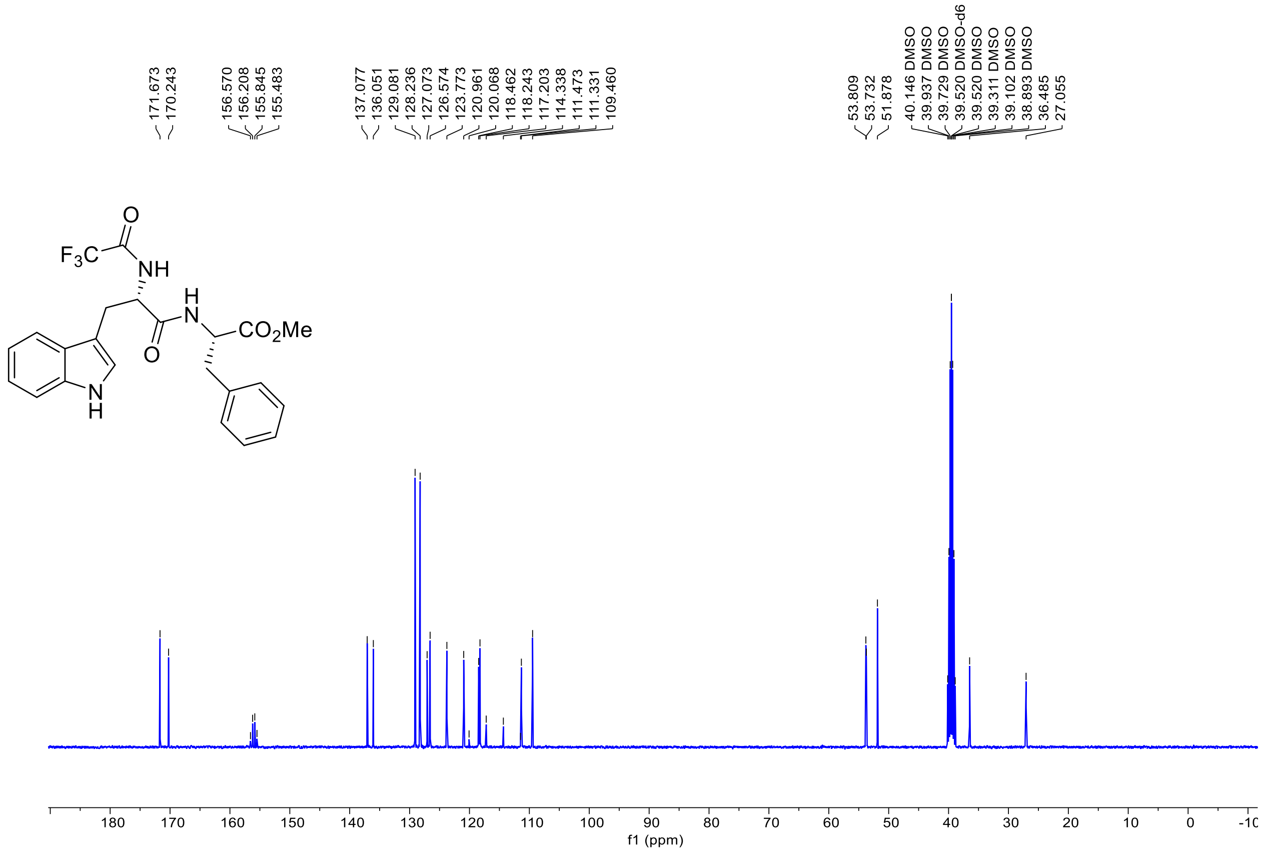

^{19}F NMR (376 MHz, DMSO)

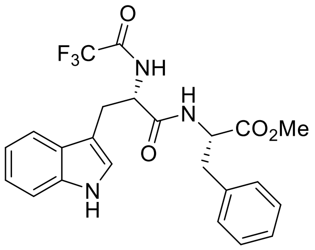

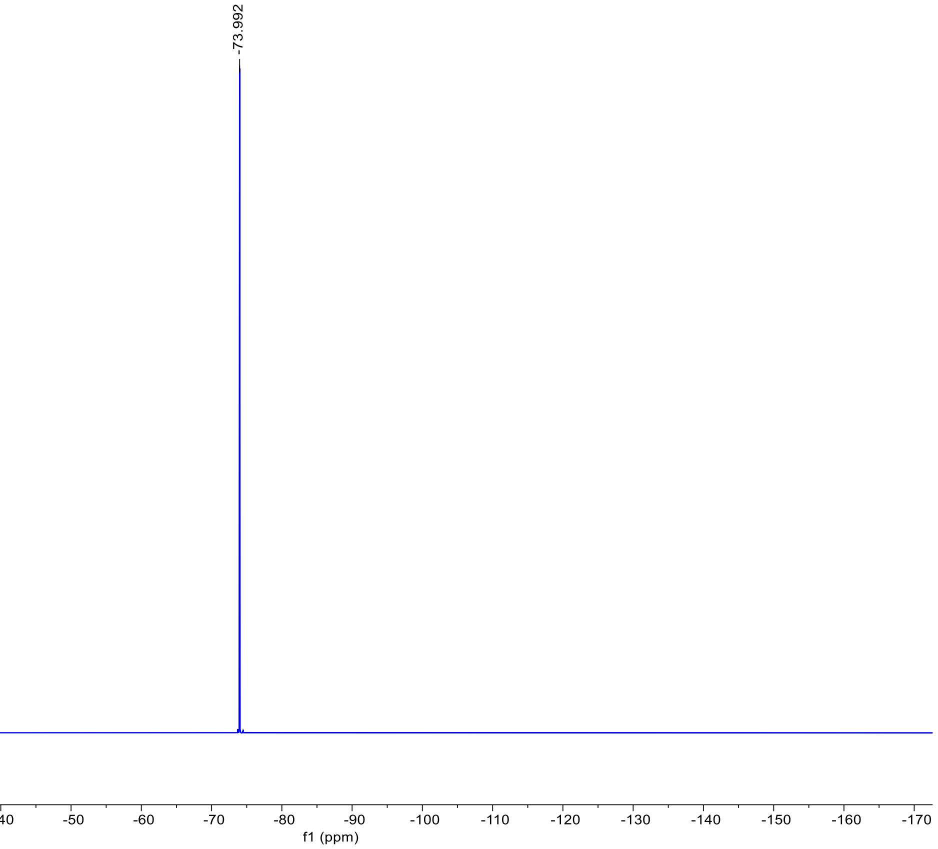

Methyl acetyl-*L*-alanyl-*L*-tryptophyl-*L*-valinate 1w

^{1}H NMR (400 MHz, DMSO)

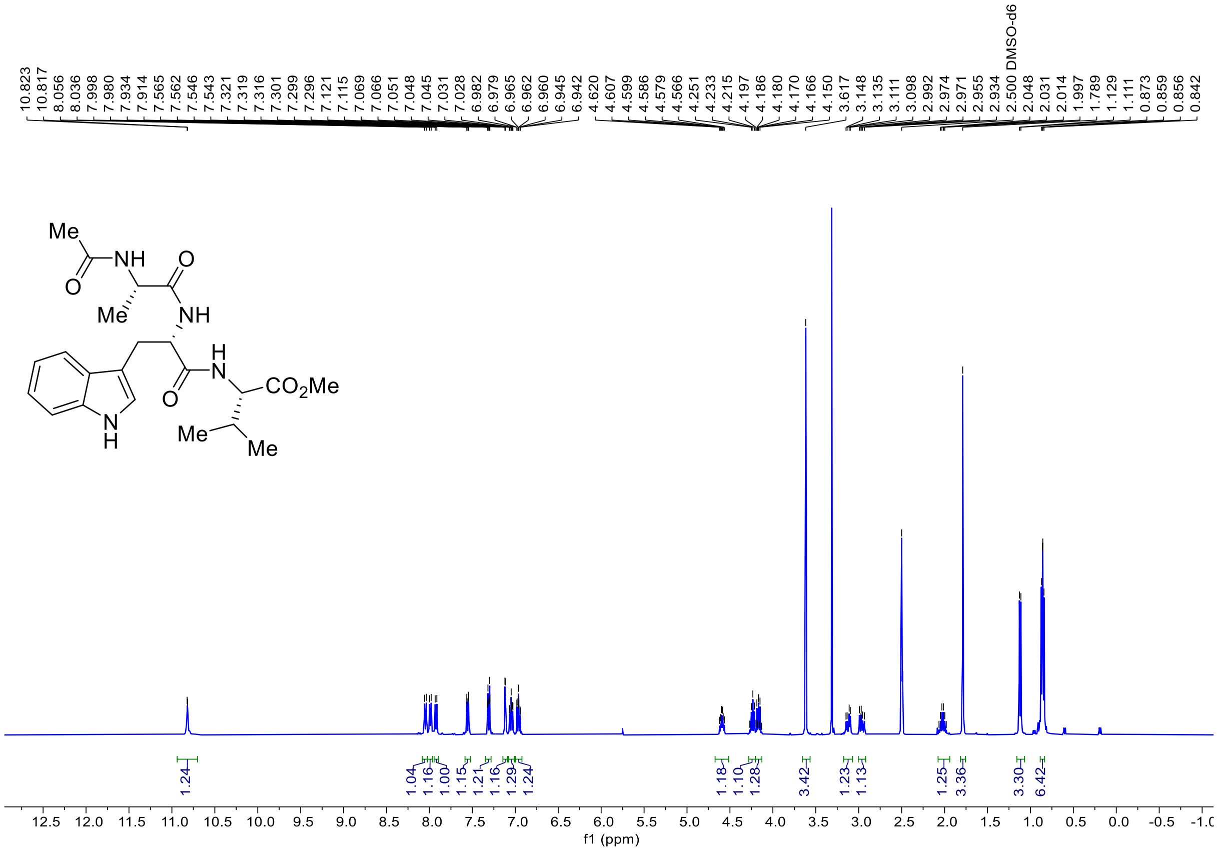

^{13}C NMR (101 MHz, DMSO)

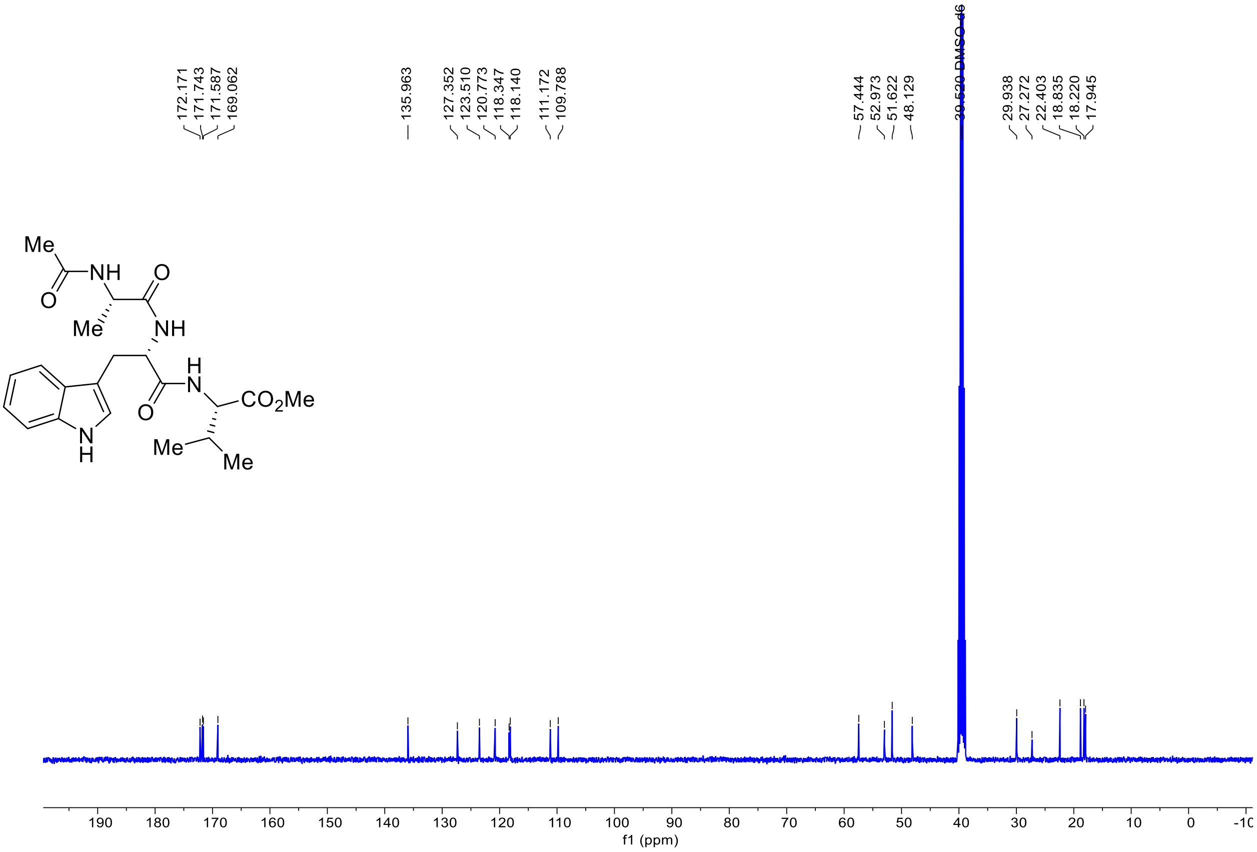

Ethyl 3-(2-(2,2,2-trifluoroacetamido)ethyl)-1*H*-indole-1-carboxylate Eoc-**1y**

^{1}H NMR (400 MHz, $CDCl_3$)

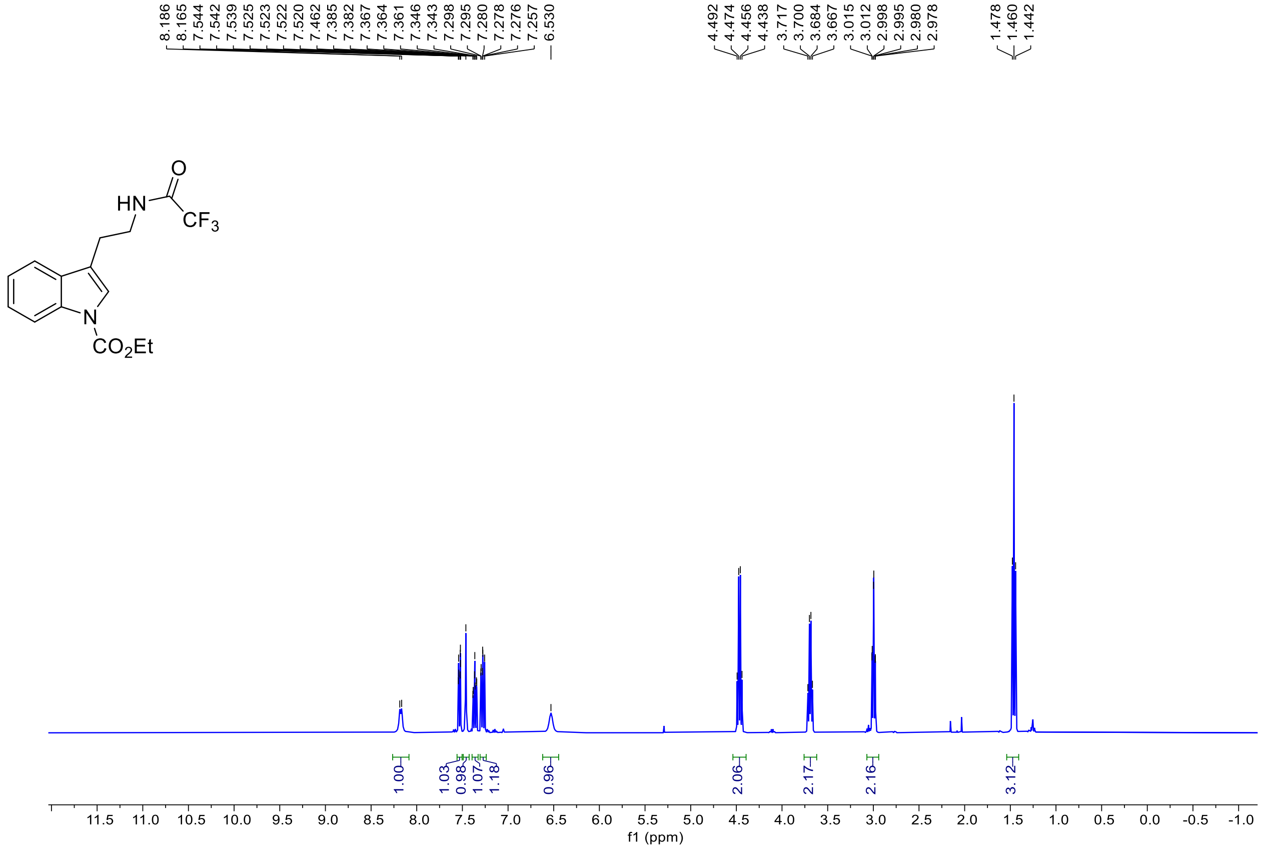

^{13}C NMR (101 MHz, $CDCl_3$)

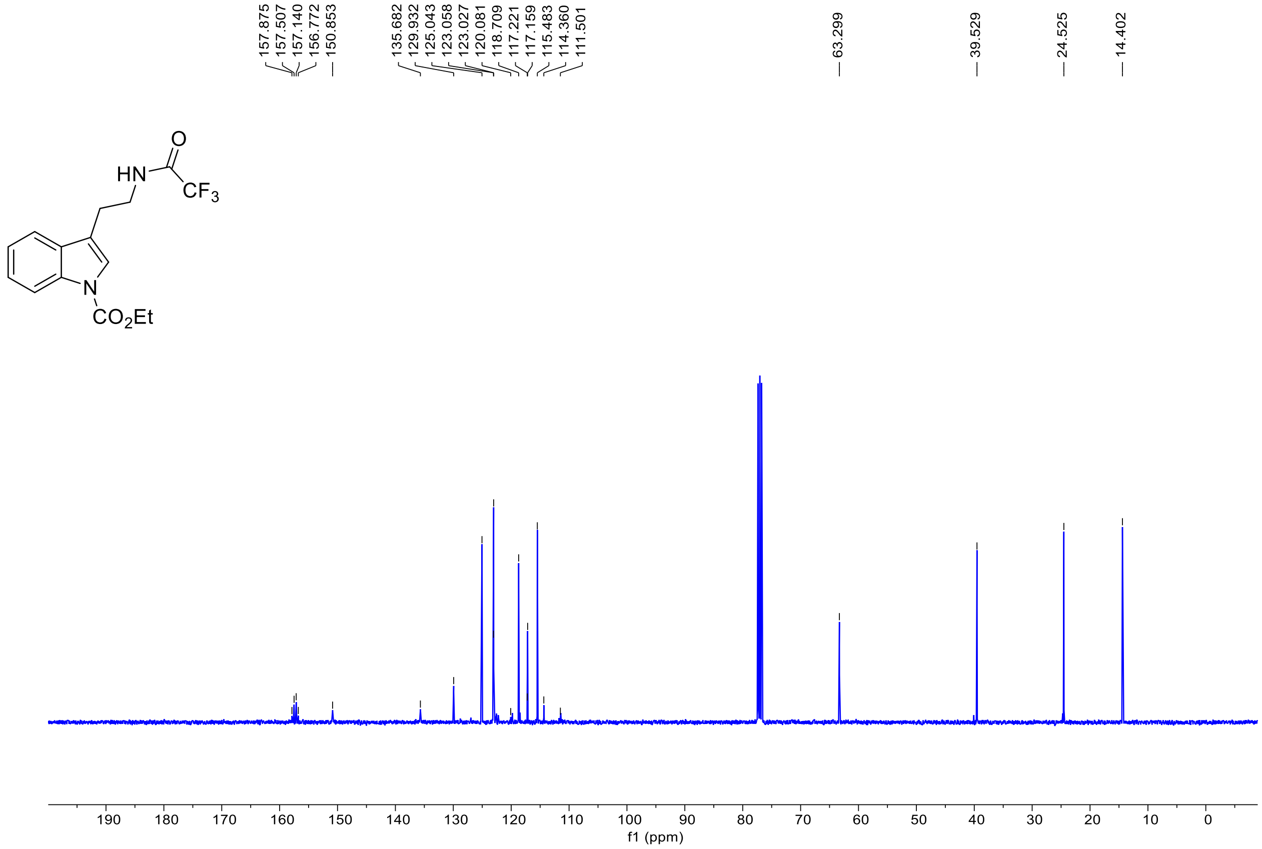

^{19}F NMR (376 MHz, $CDCl_3$)

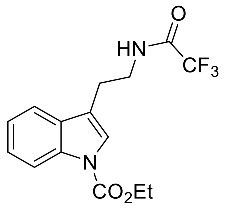

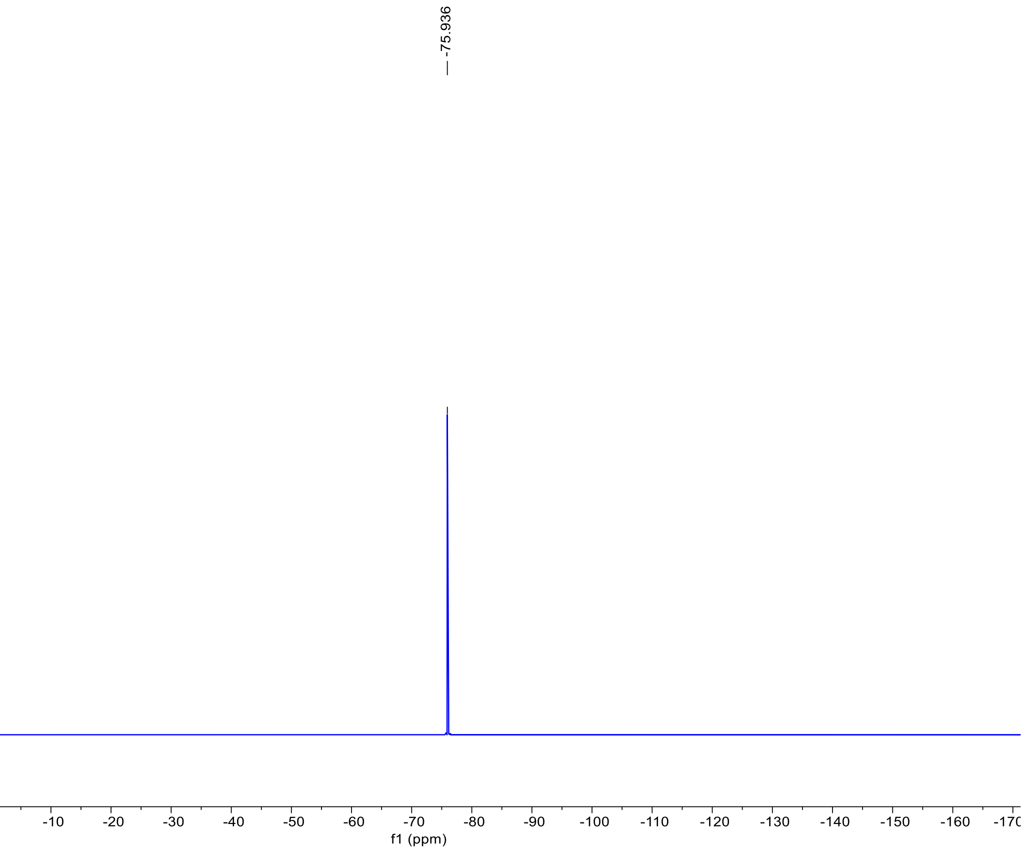

3-(1*H*-indol-3-yl)-1-morpholinopropan-1-one 1z

^{1}H NMR (300 MHz, $CDCl_3$)

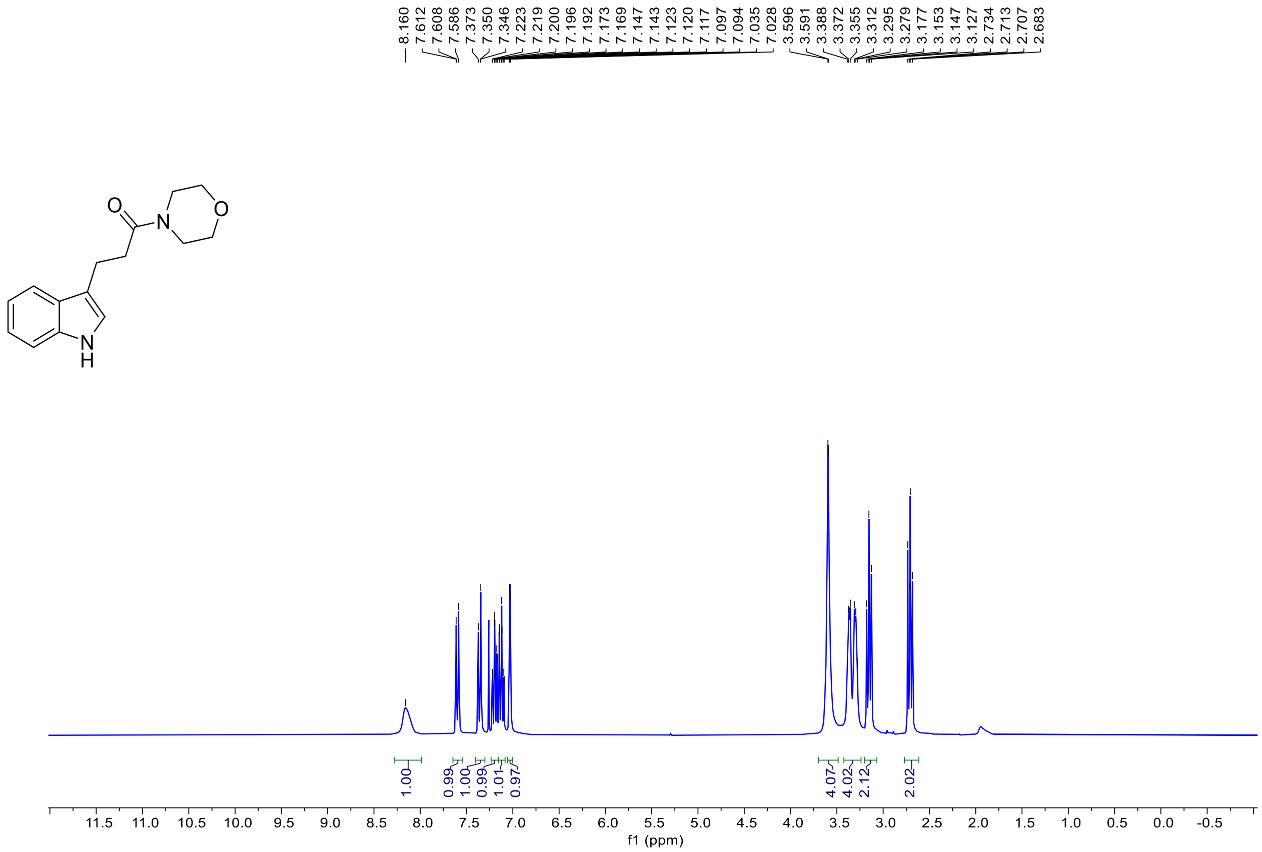

^{13}C NMR (76 MHz, $CDCl_3$)

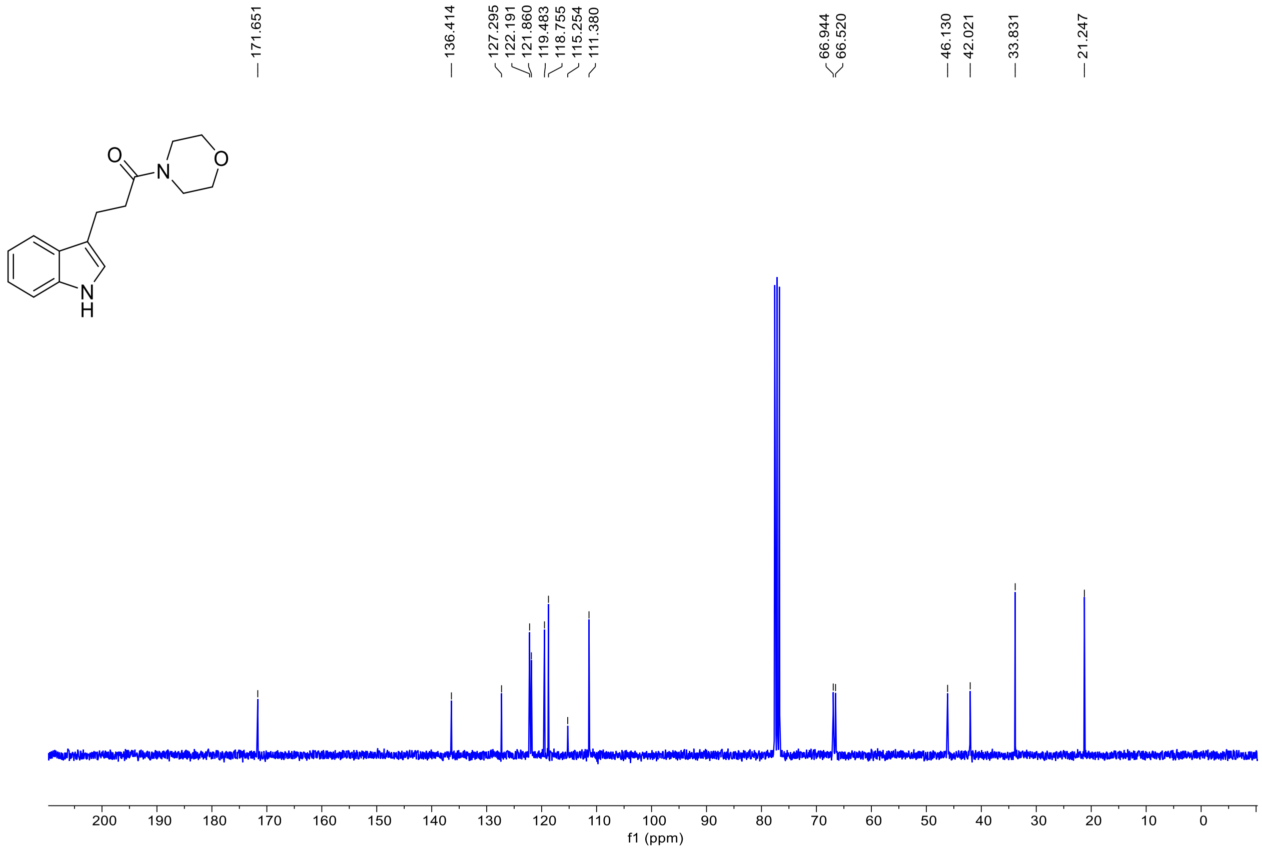

3-Methylbenzofuran-5-yl 3-(4,5-diphenyloxazol-2-yl)propanoate 4r

^{1}H NMR (400 MHz, $CDCl_3$)

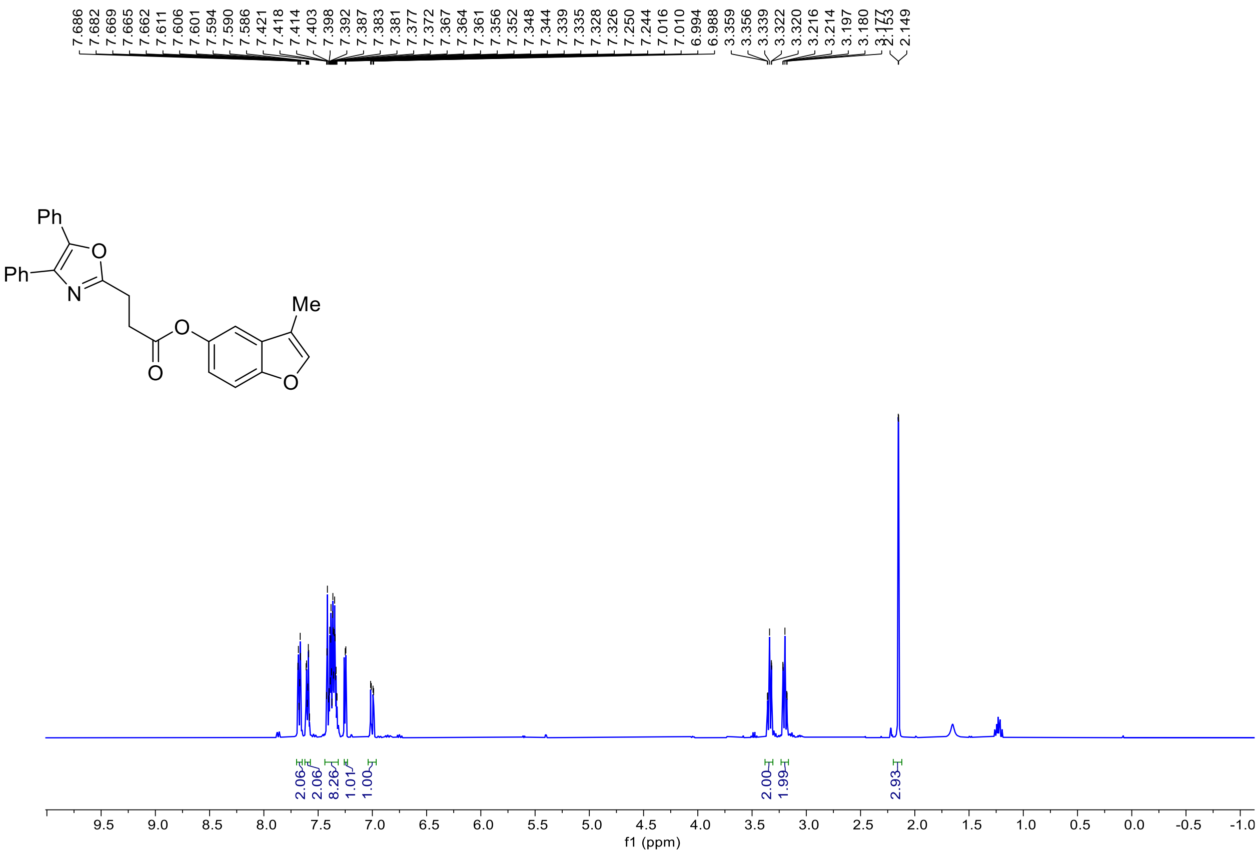

^{13}C NMR (101 MHz, $CDCl_3$)

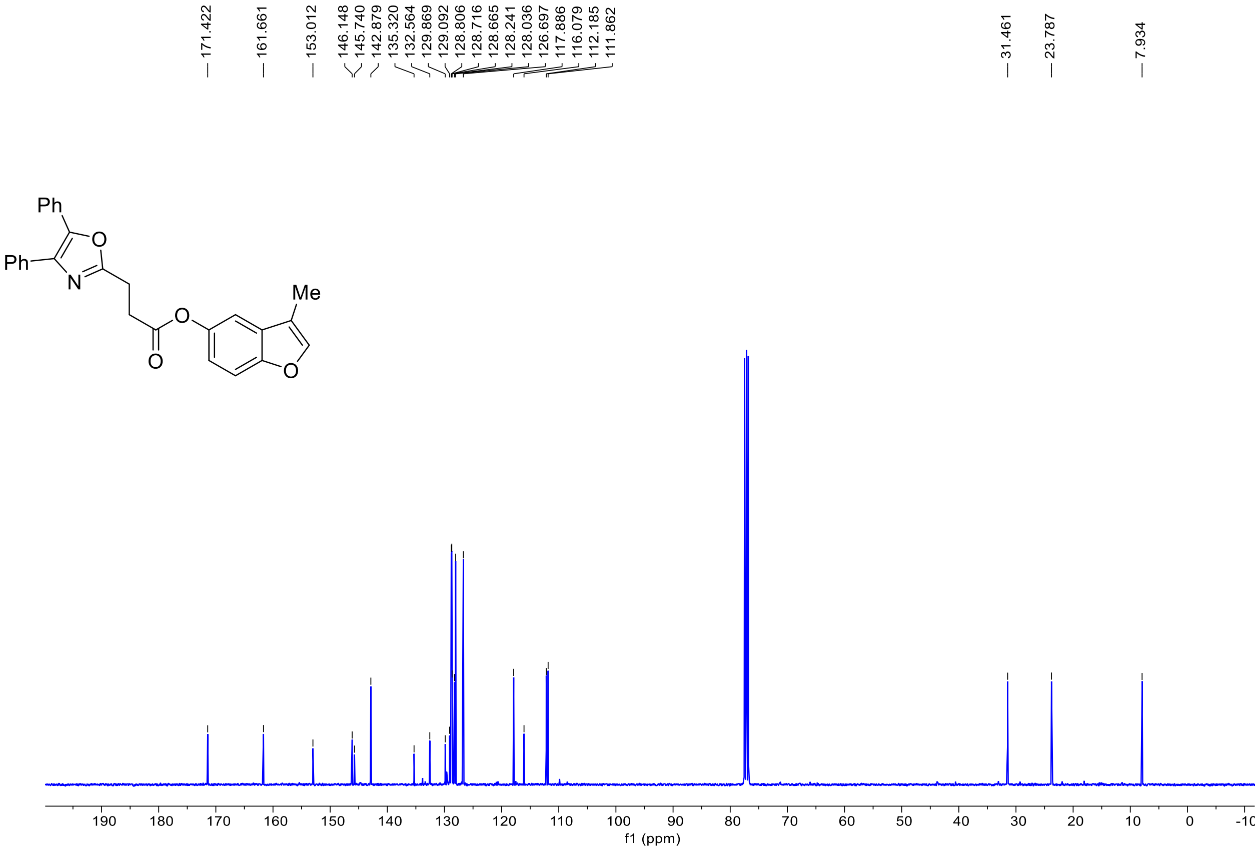

3-Methylbenzofuran-5-yl 2-(1-(4-chlorobenzoyl)-5-methoxy-2-methyl-1H-indol-3-yl)acetate 4s

^{1}H NMR (400 MHz, $CDCl_3$)

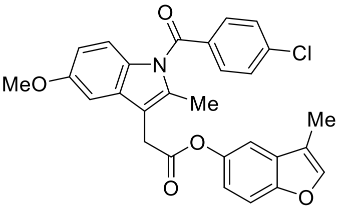

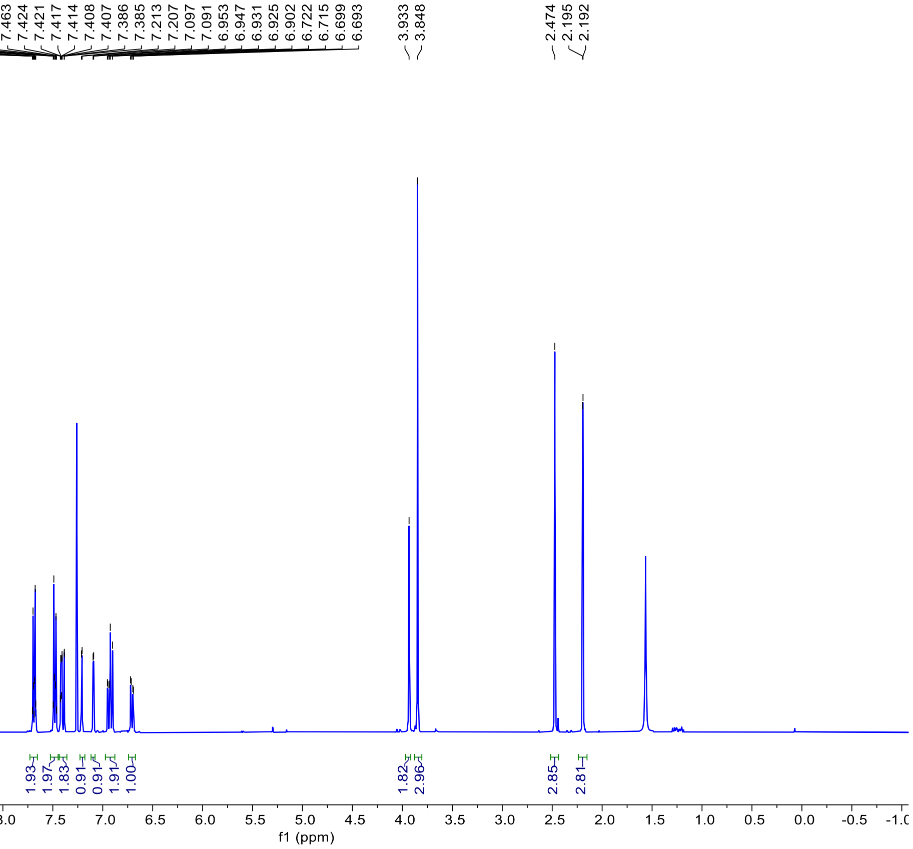

^{13}C NMR (101 MHz, $CDCl_3$)

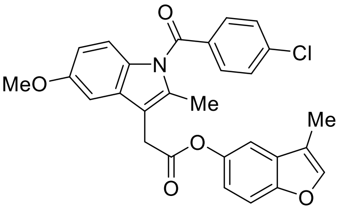

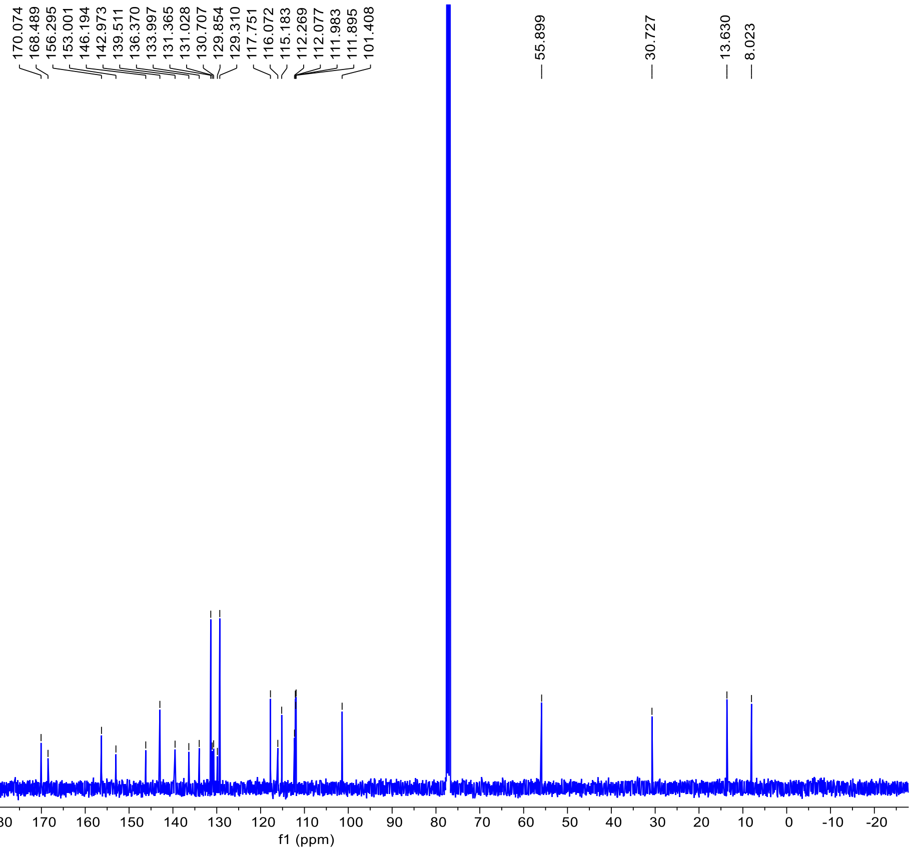

4-(Nitroso-^{15}*N*)morpholine

^{1}H NMR (300 MHz, $CDCl_3$)

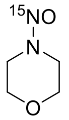

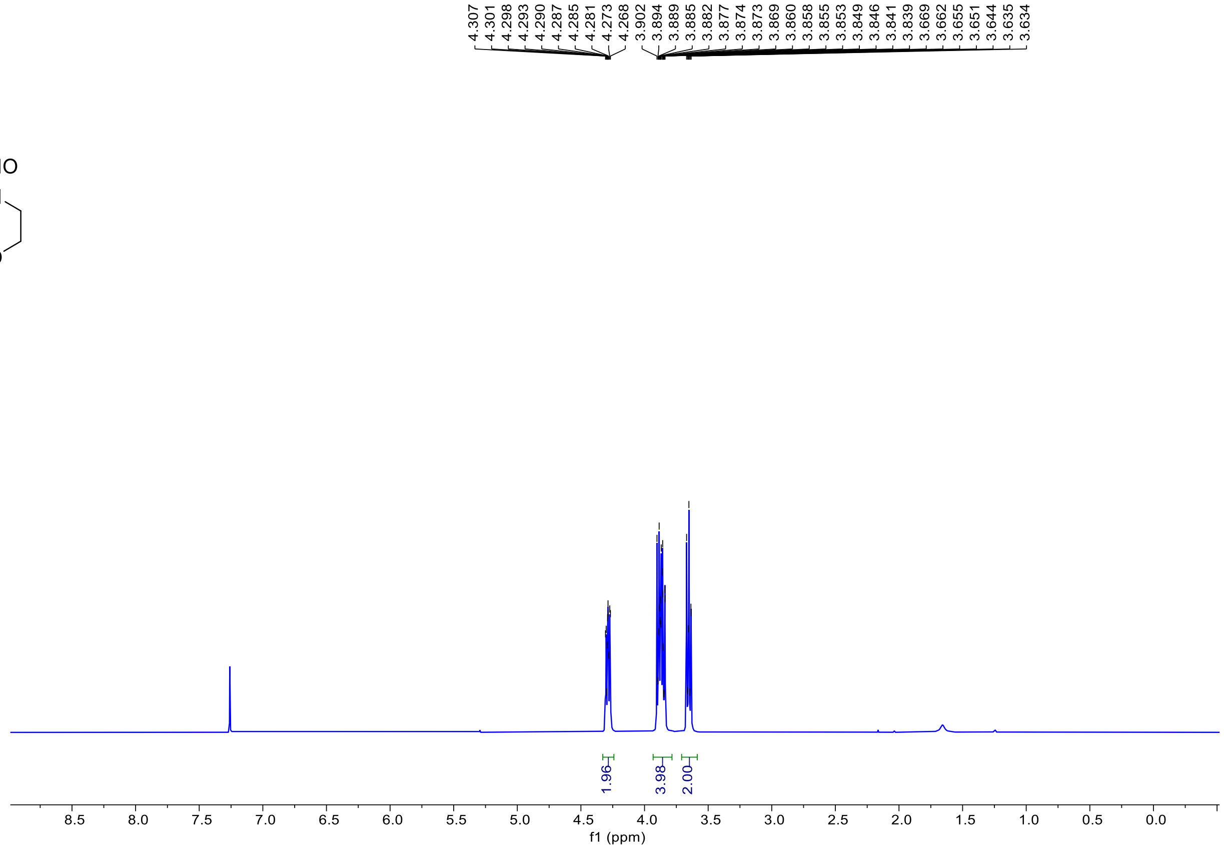

^{13}C NMR (76 MHz, $CDCl_3$)

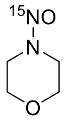

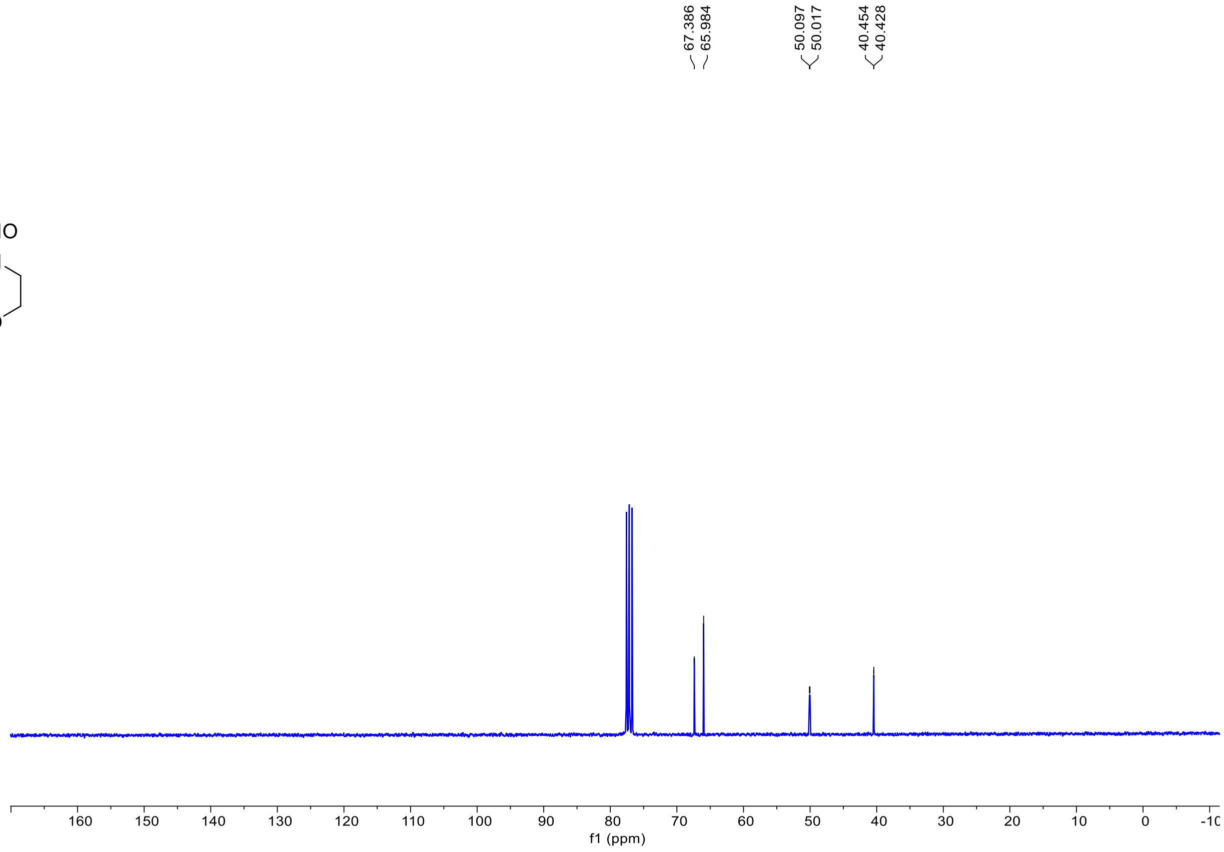

Methyl (*E*)-(2-(1-(hydroxyimino)ethyl)phenyl)carbamate (I-1)

^{1}H NMR (300 MHz, $CDCl_3$)

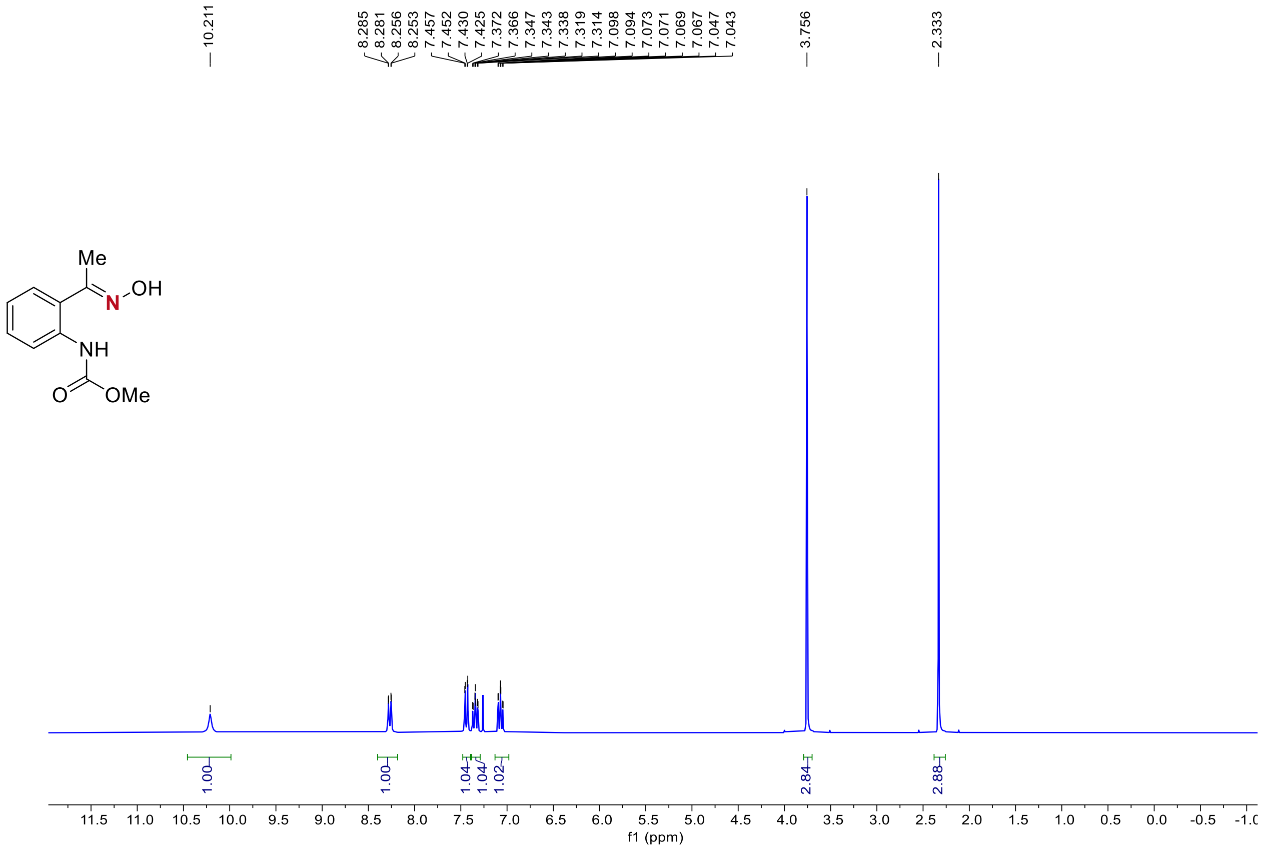

^{13}C NMR (76 MHz, $CDCl_3$)

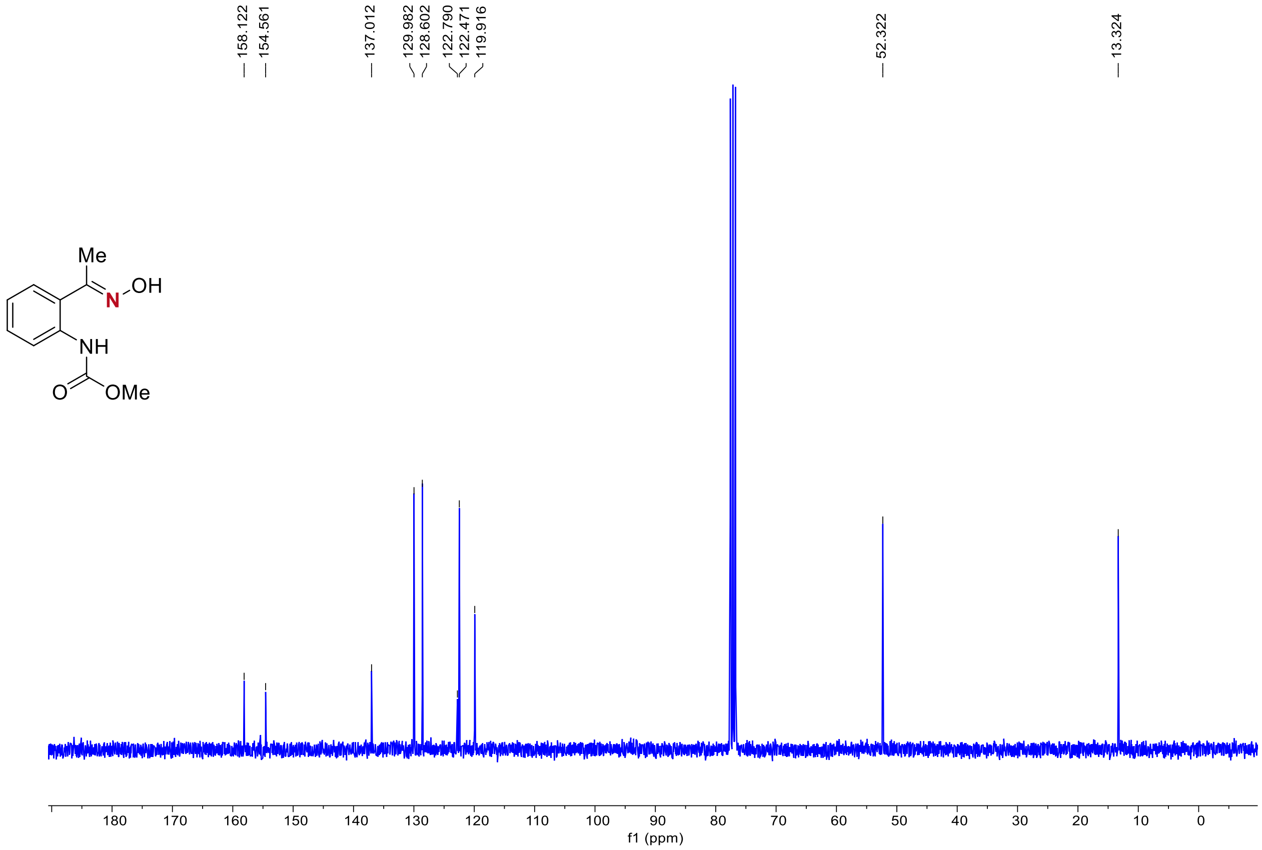

1-(2-Aminophenyl)ethan-1-one (I-2)

^{1}H NMR (400 MHz, $CDCl_3$)

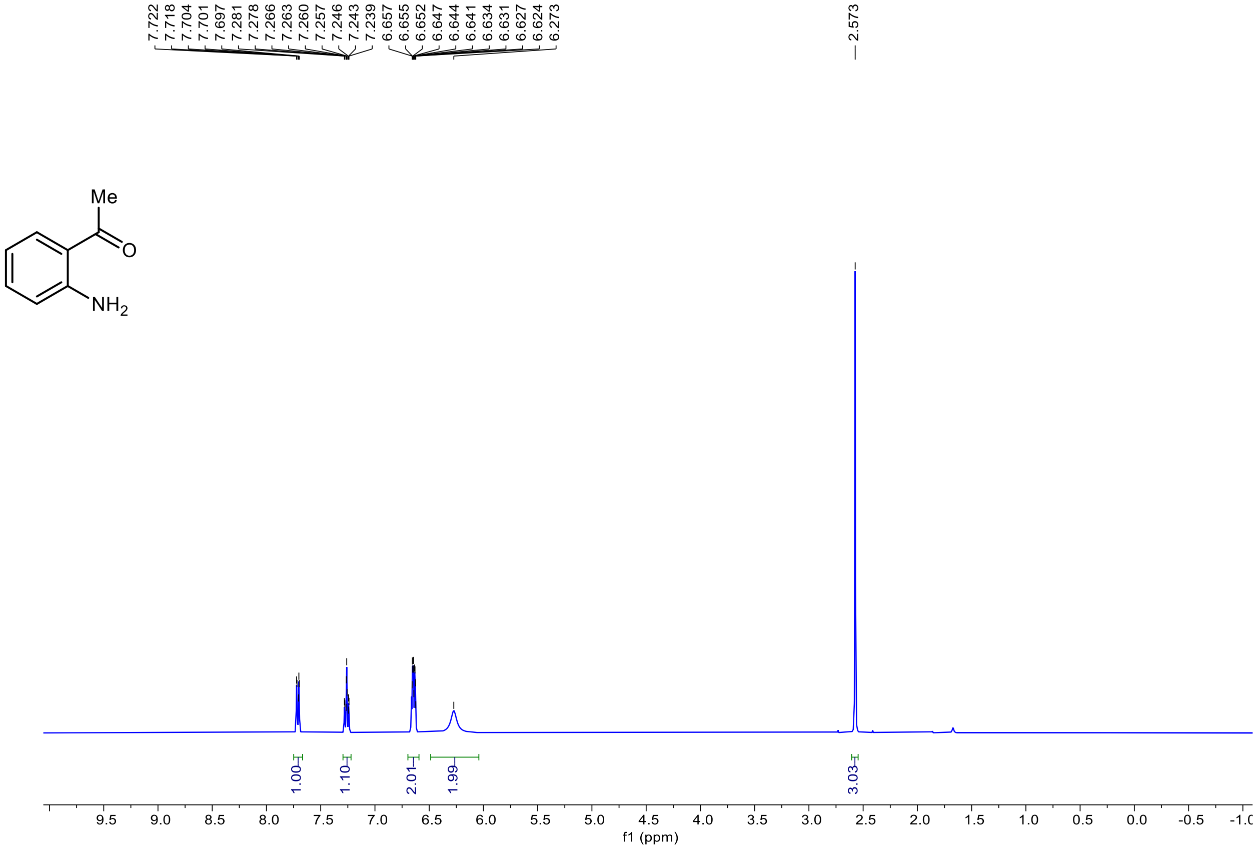

^{13}C NMR (101 MHz, $CDCl_3$)

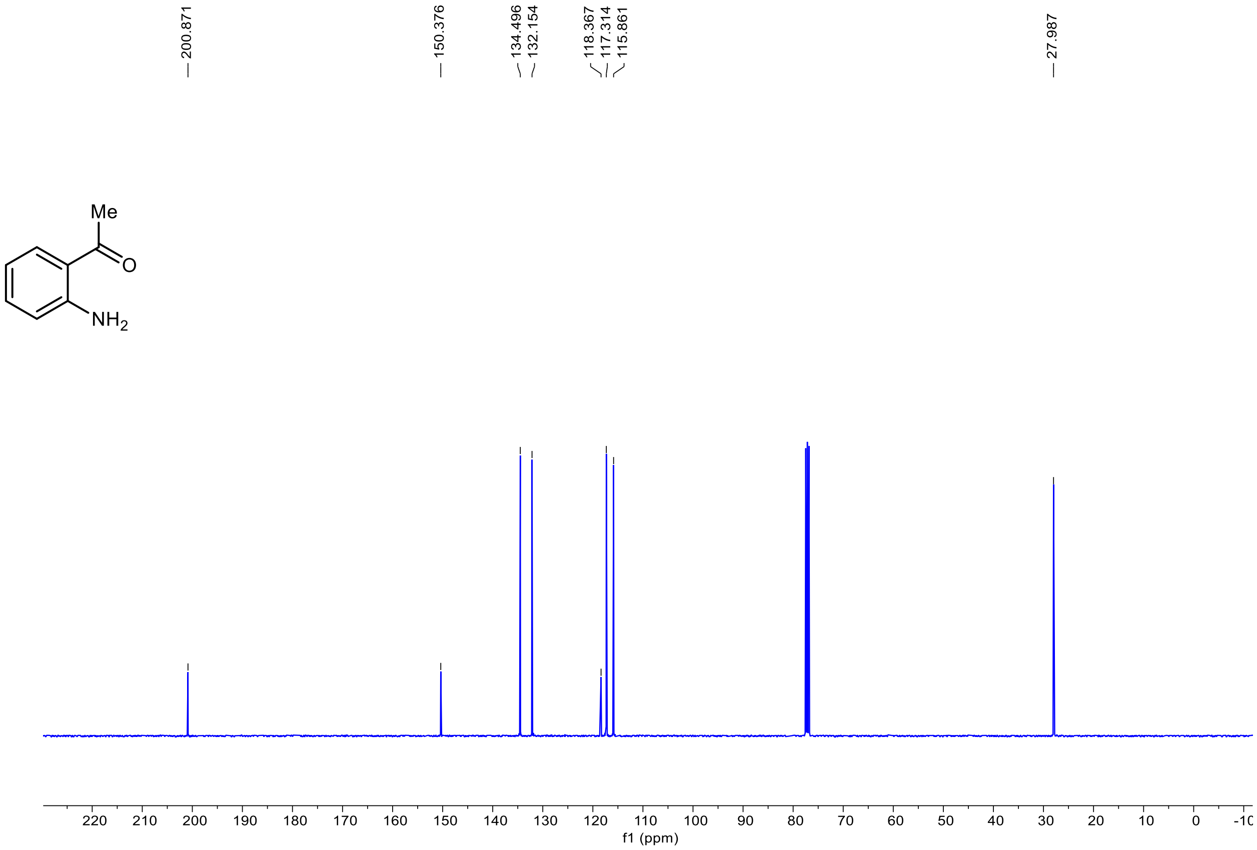

(*E*)-1-(2-hydroxyphenyl)ethan-1-one oxime (I-3)

^{1}H NMR (400 MHz, $CDCl_3$)

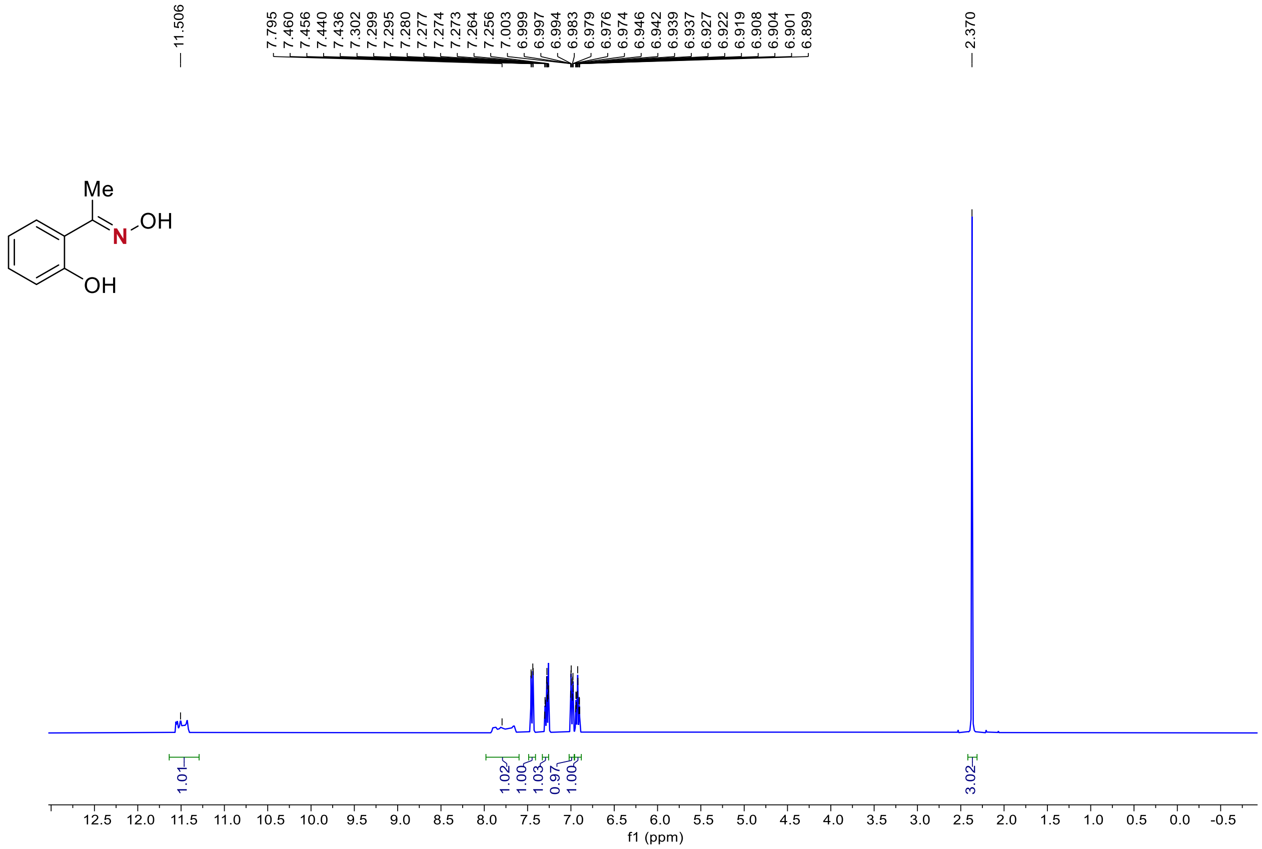

^{13}C NMR (101 MHz, $CDCl_3$)

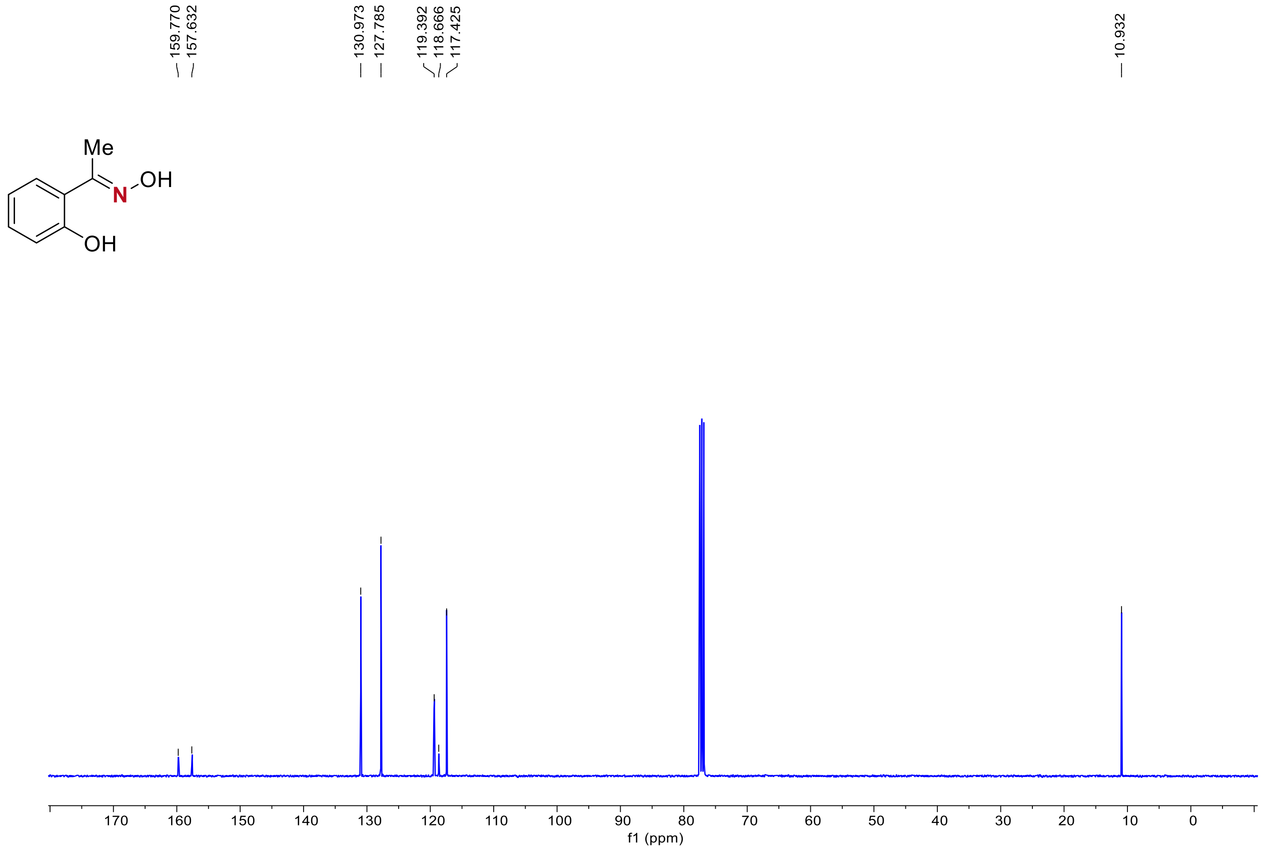

2-(Imino(phenyl)methyl)phenol (I-4)

^{1}H NMR (300 MHz, $CDCl_3$, crude)

Crude imine intermediate I-**4** was identified by crude ^{1}H NMR without purification.

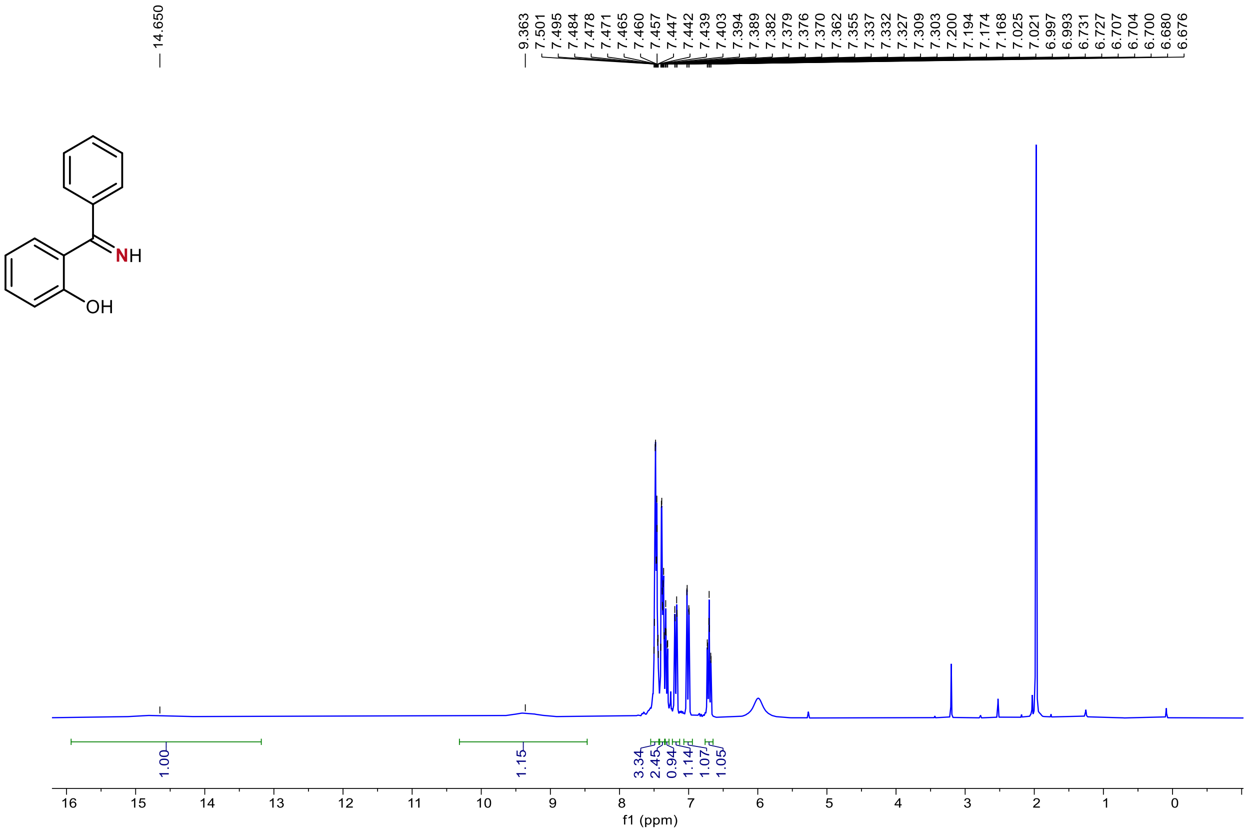

^{13}C NMR (76 MHz, $CDCl_3$)

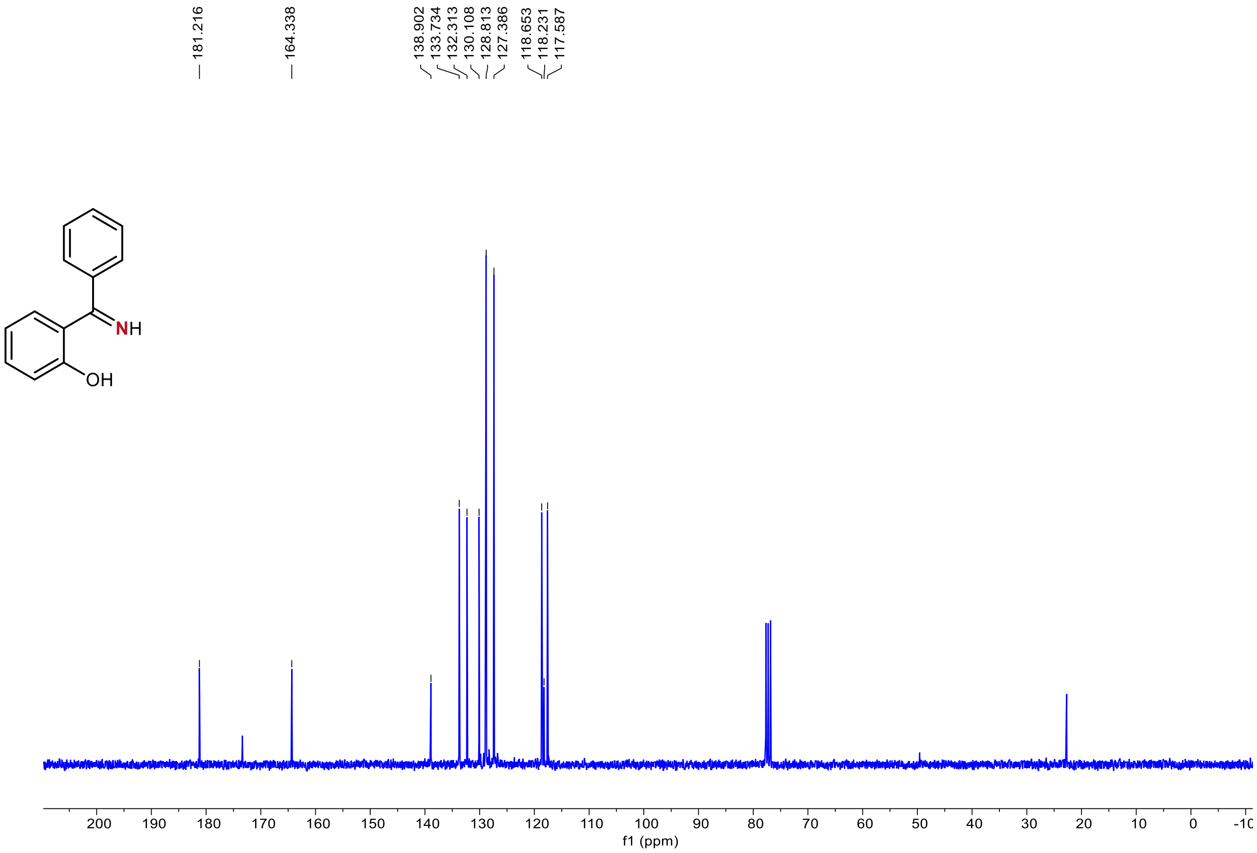

Methyl 3-methyl-1*H*-indazole-1-carboxylate Moc-**2a**

^{1}H NMR (300 MHz, $CDCl_3$)

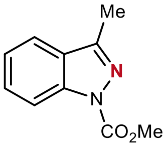

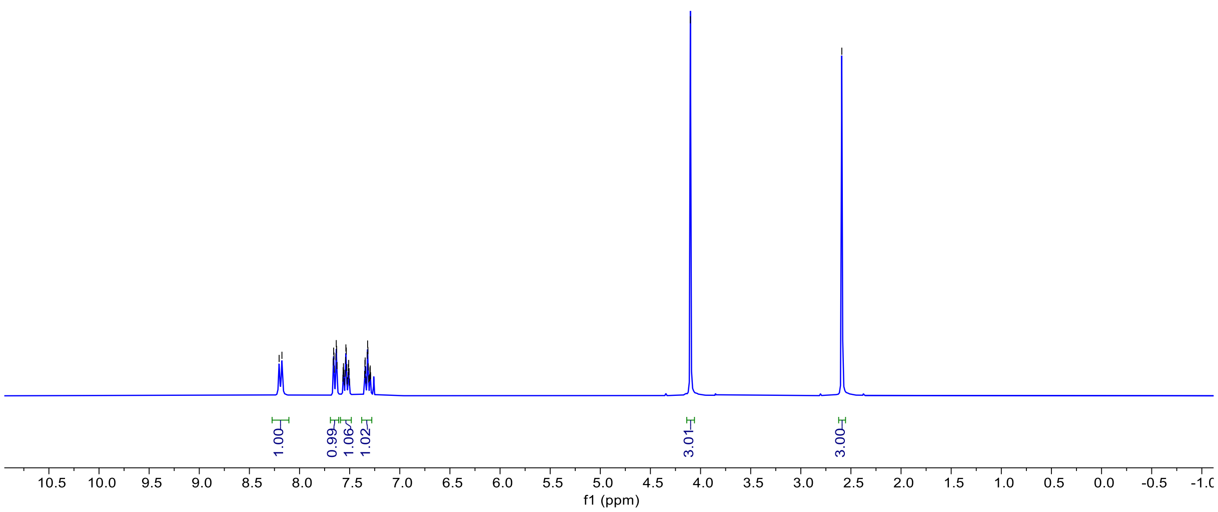

^{13}C NMR (76 MHz, $CDCl_3$)

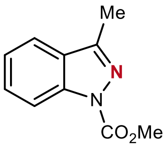

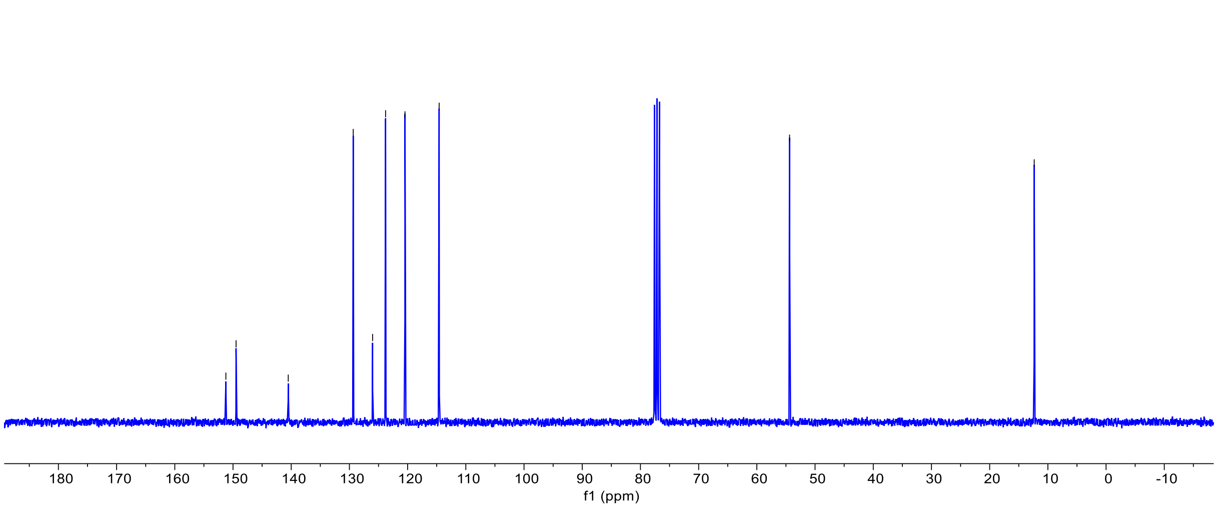

Methyl 2-methyl-1*H*-benzo[*d*]imidazole-1-carboxylate Moc-**3a**

^{1}H NMR (300 MHz, $CDCl_3$)

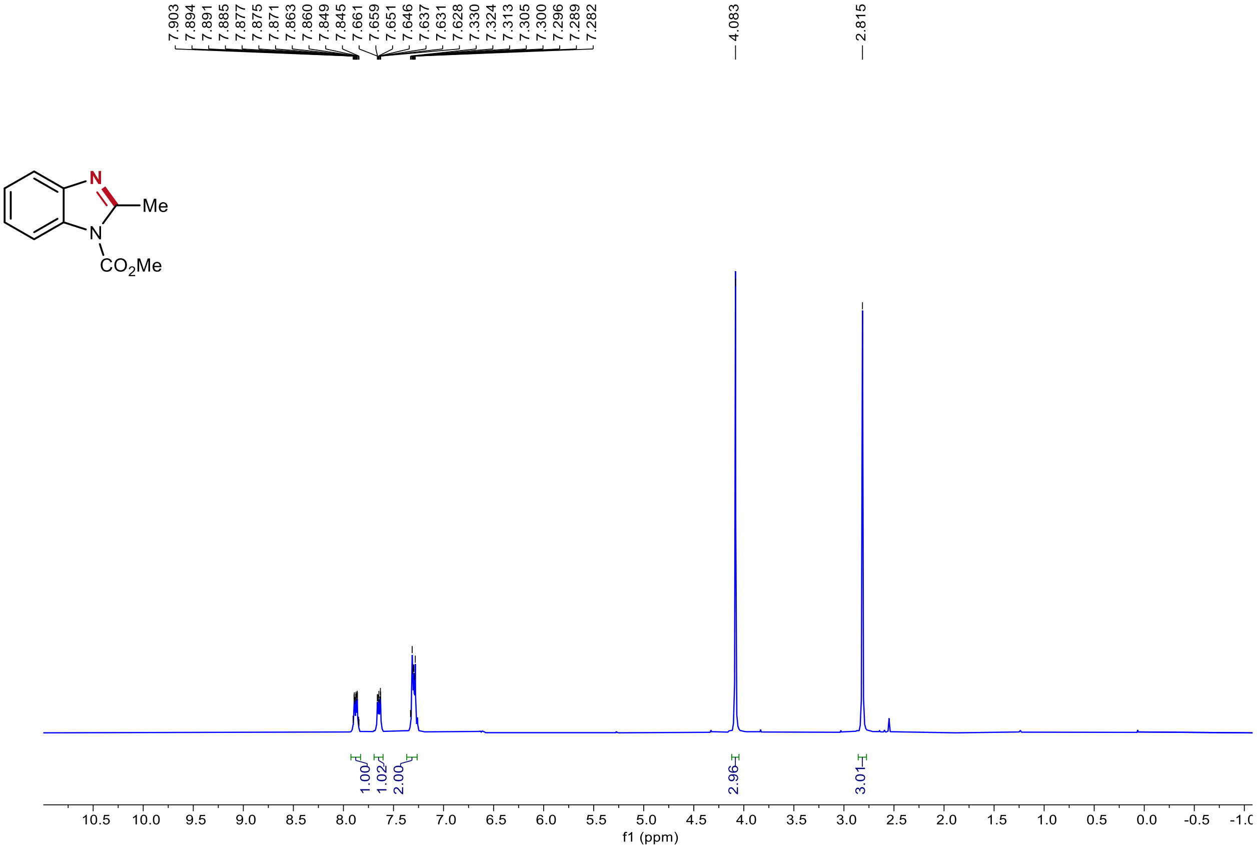

^{13}C NMR (76 MHz, $CDCl_3$)

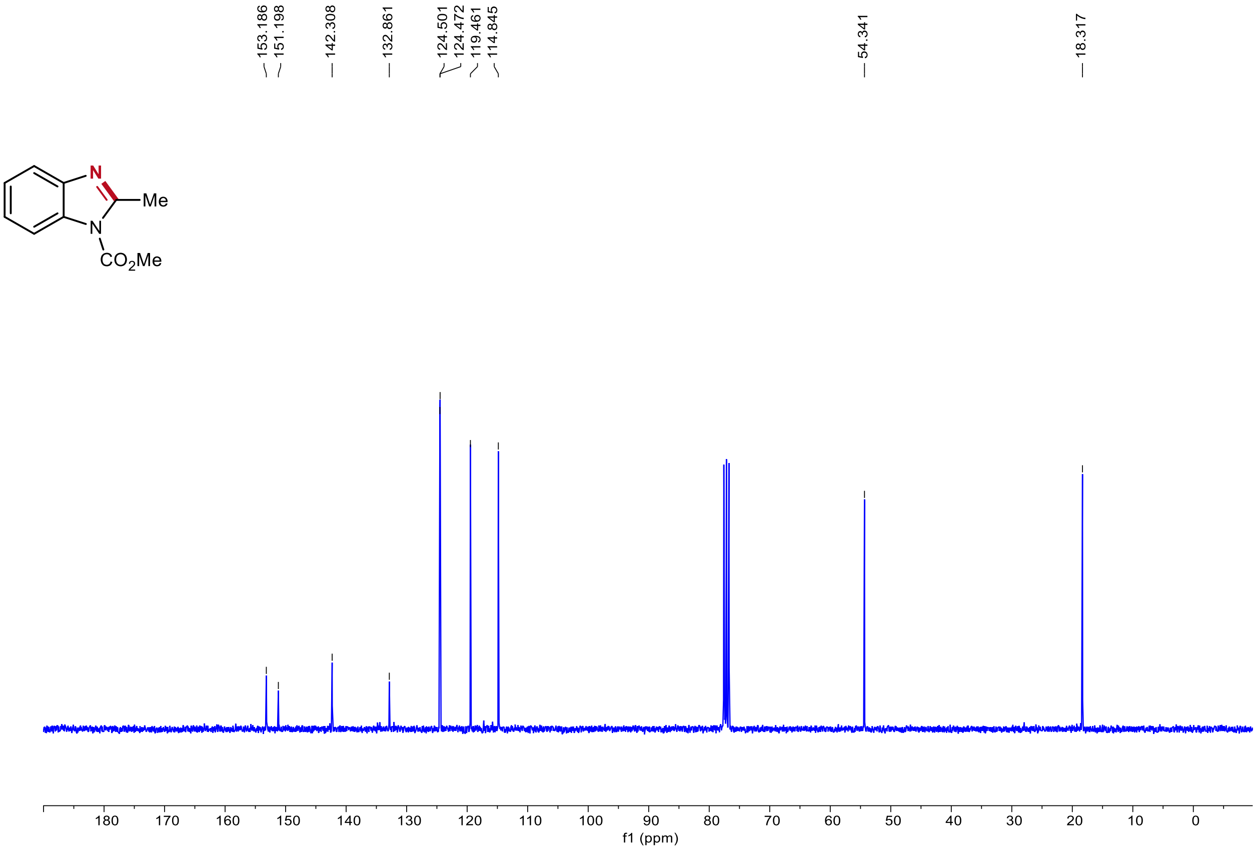

3-methyl-1*H*-indazole 2a

^{1}H NMR (300 MHz, $CDCl_3$)

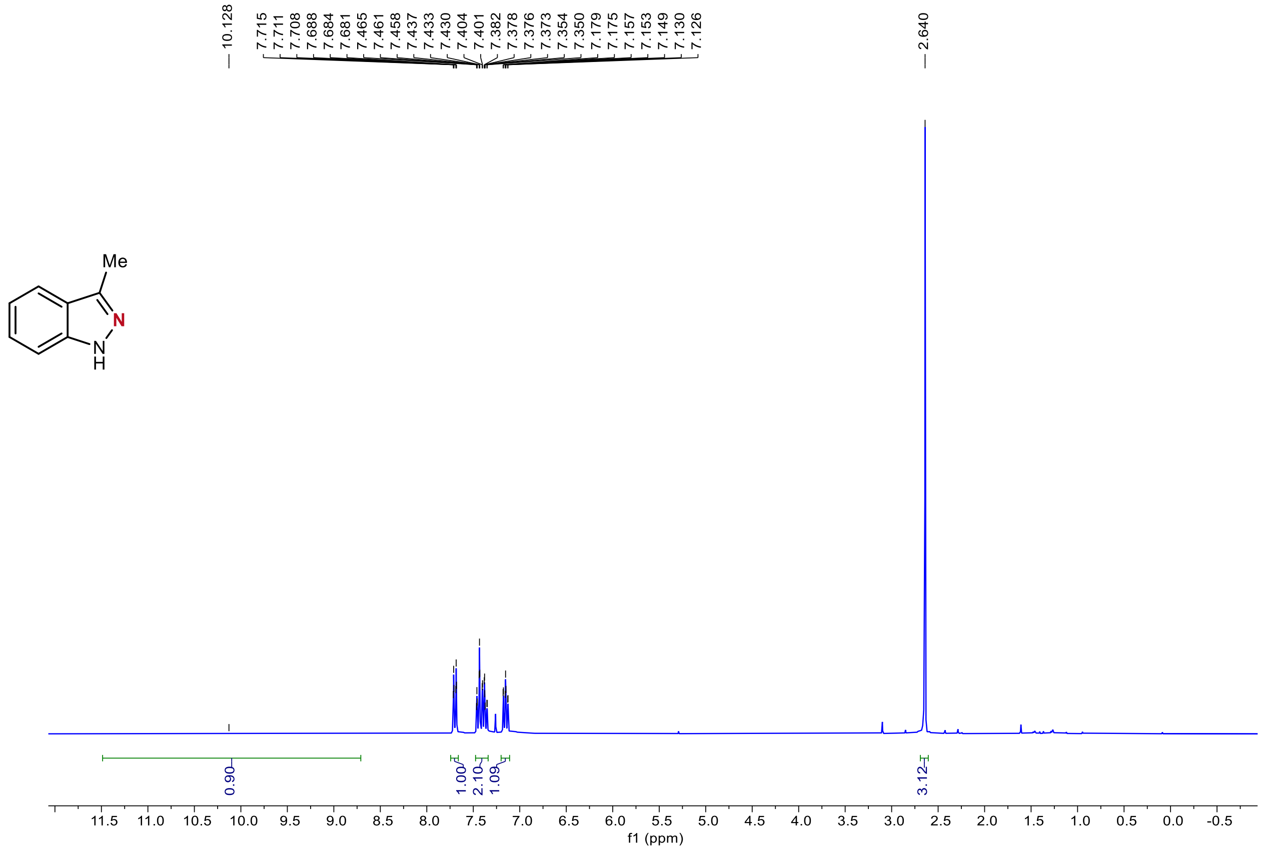

^{13}C NMR (76 MHz, $CDCl_3$)

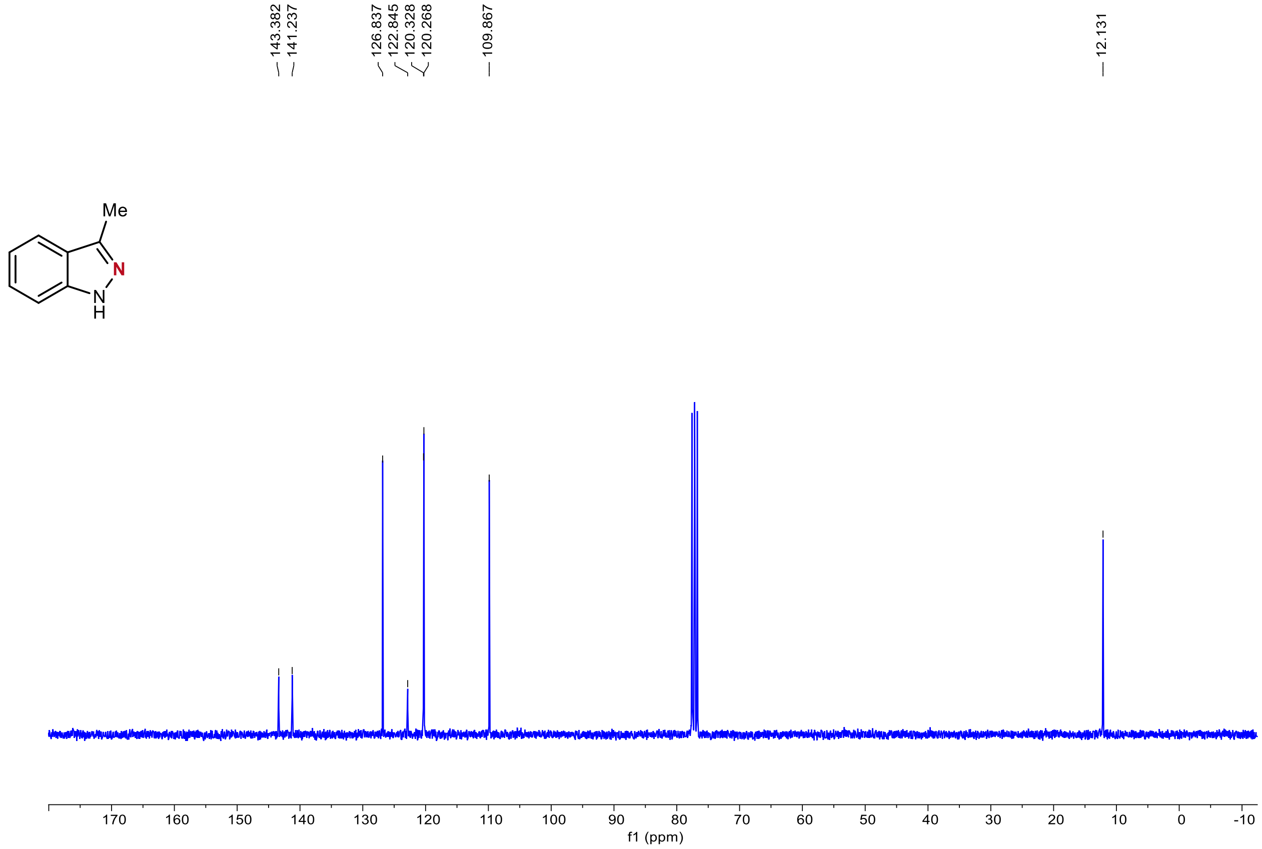

2-Methyl-1-tosyl-1*H*-benzo[*d*]imidazole Ts-**3a**

^{1}H NMR (300 MHz, $CDCl_3$)

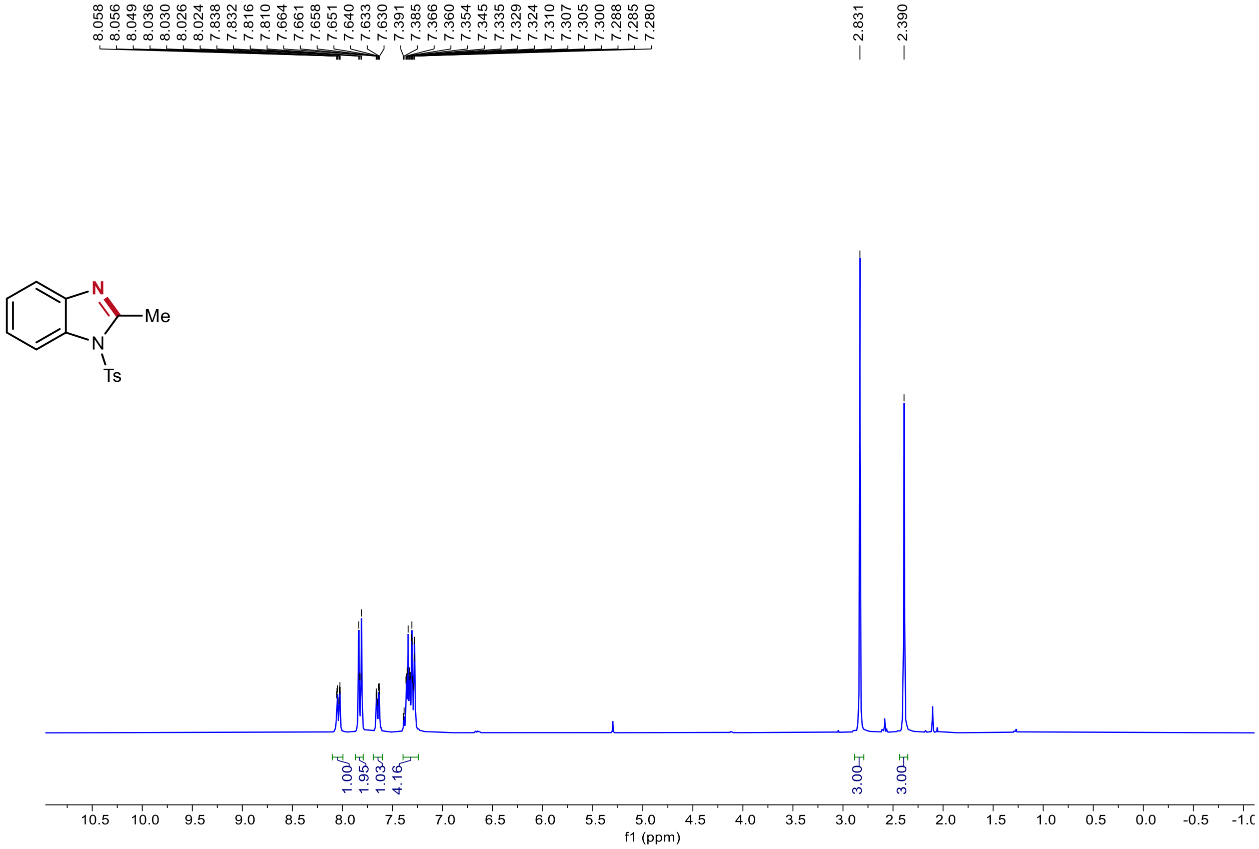

^{13}C NMR (76 MHz, $CDCl_3$)

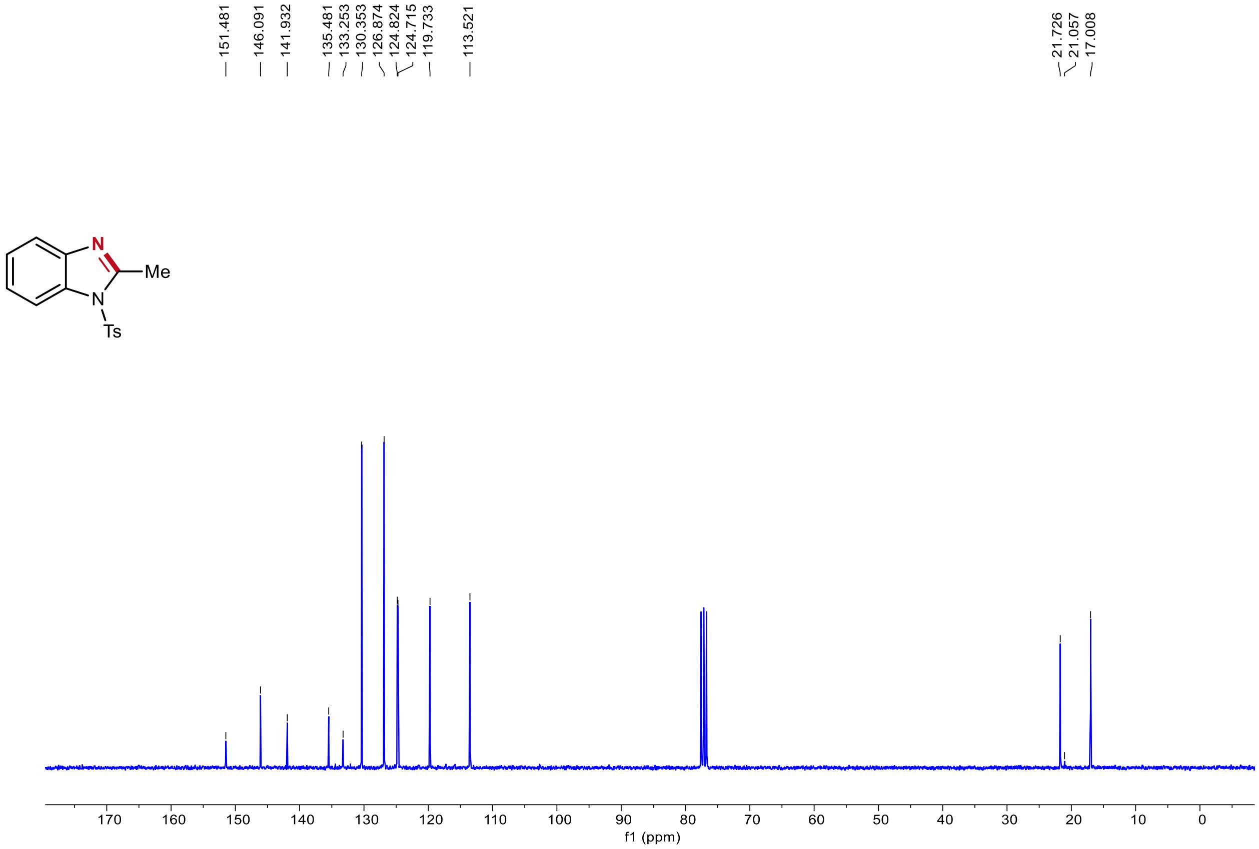

Methyl 5-methoxy-3-methyl-1*H*-indazole-1-carboxylate Moc-**2b**

^{1}H NMR (300 MHz, $CDCl_3$)

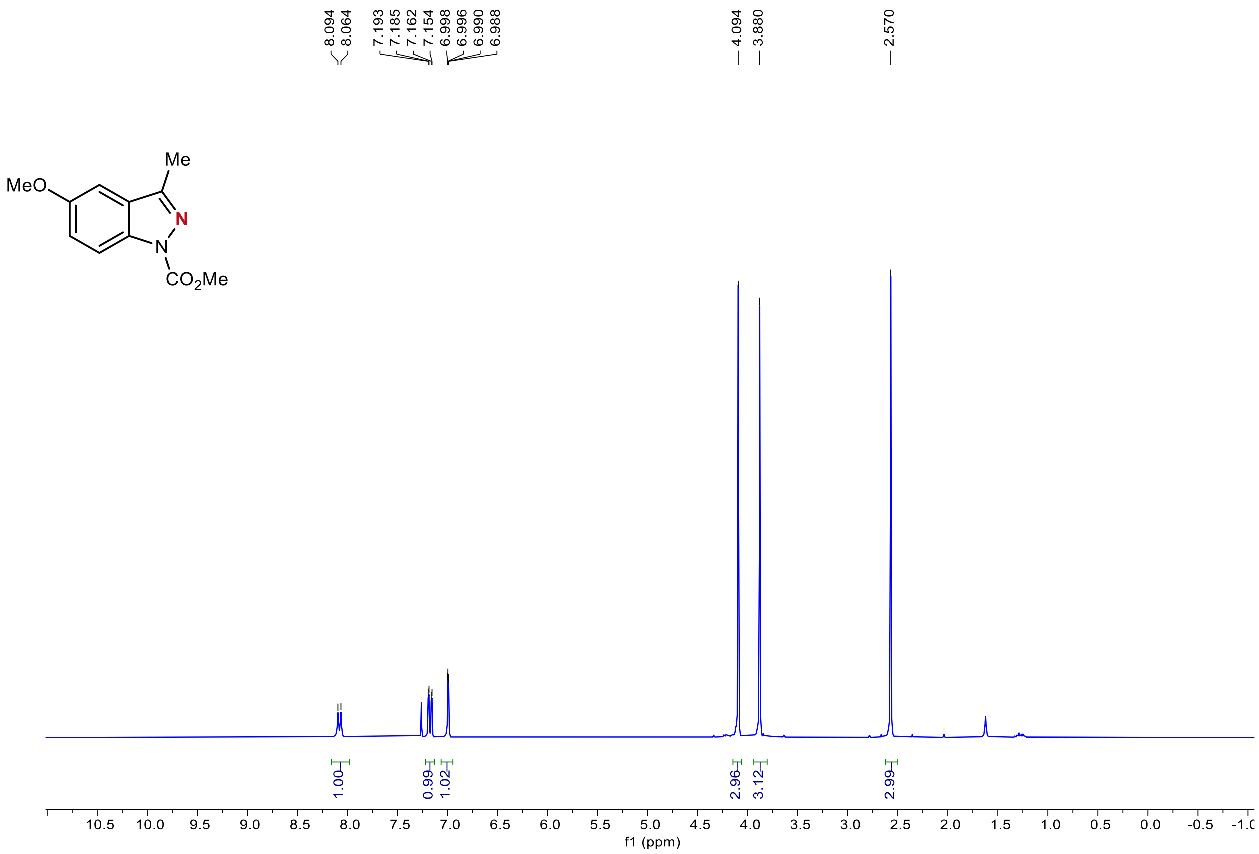

^{13}C NMR (76 MHz, $CDCl_3$)

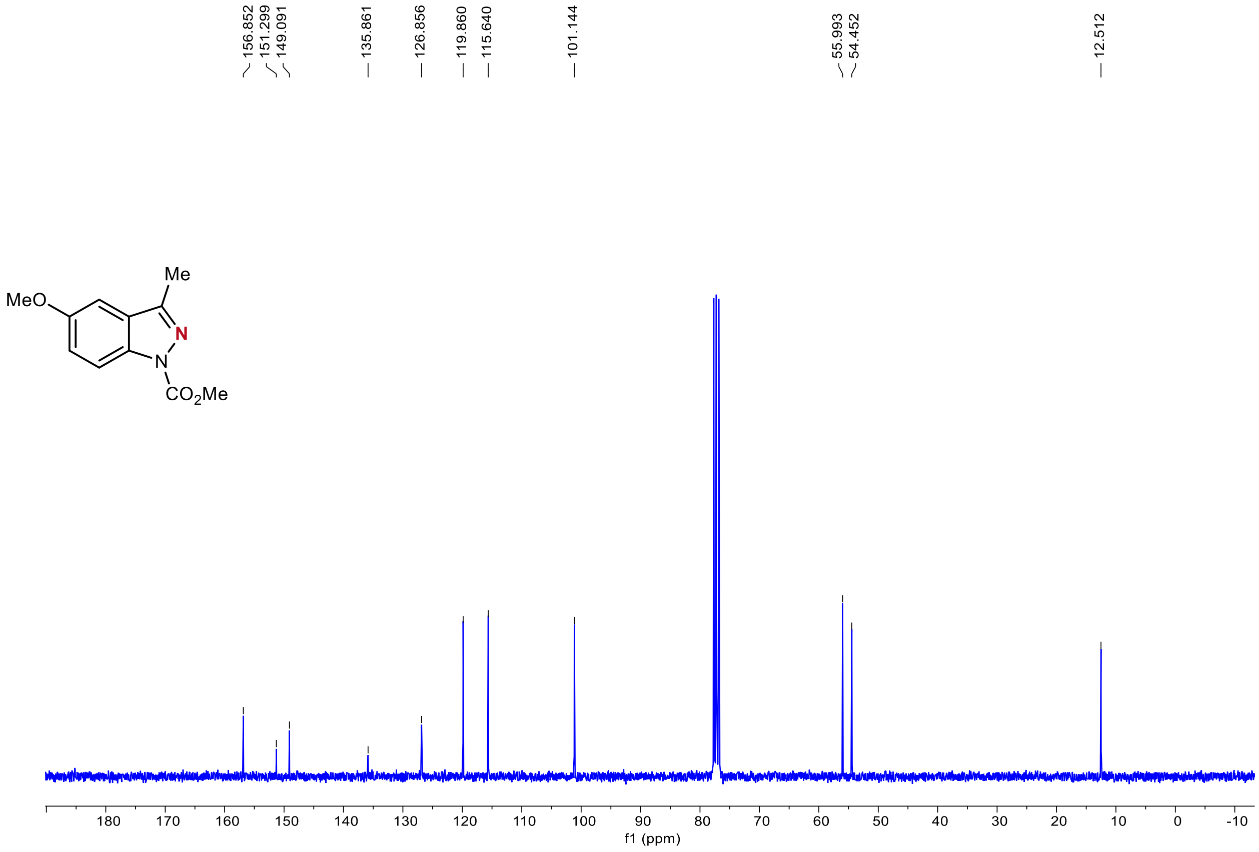

Methyl 5-methoxy-2-methyl-1H-benzo[d]imidazole-1-carboxylate Moc-**3b**

^{1}H NMR (300 MHz, $CDCl_3$)

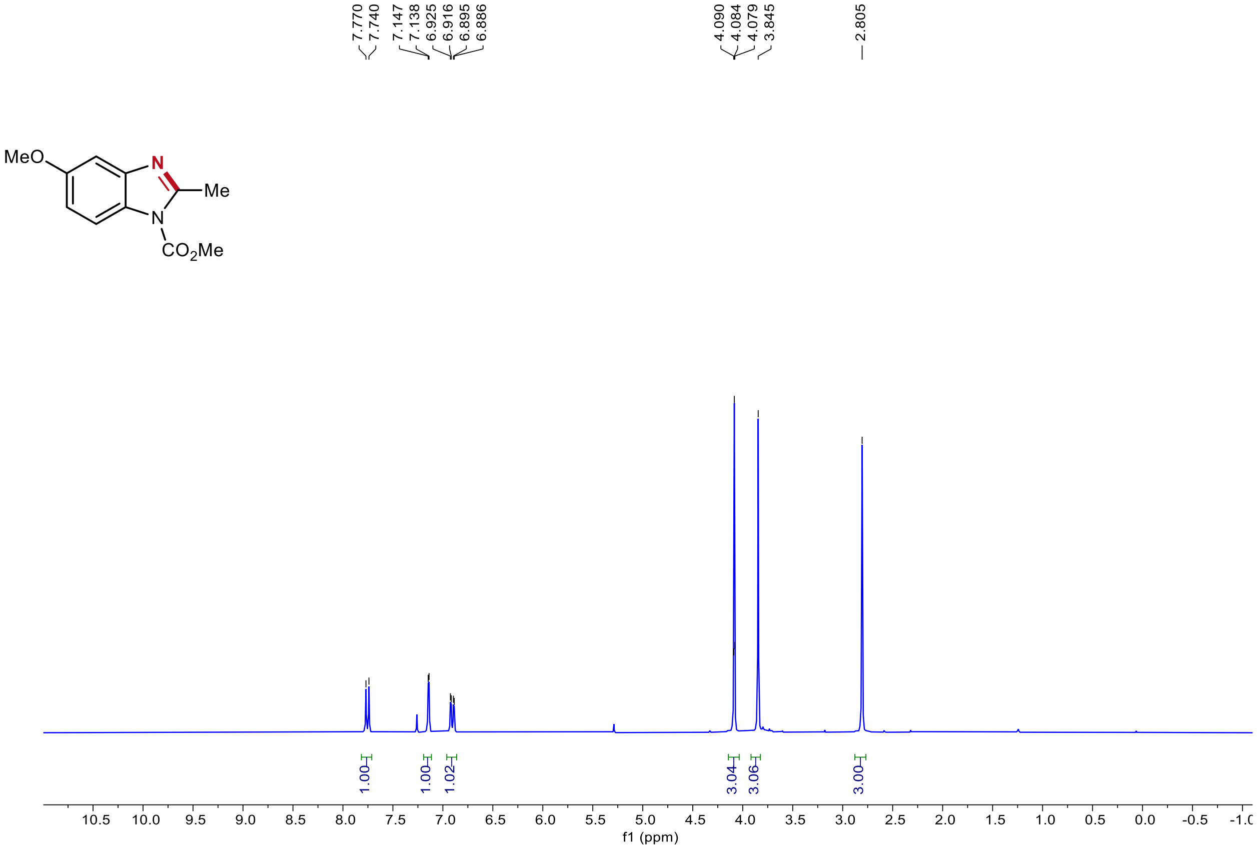

^{13}C NMR (76 MHz, $CDCl_3$)

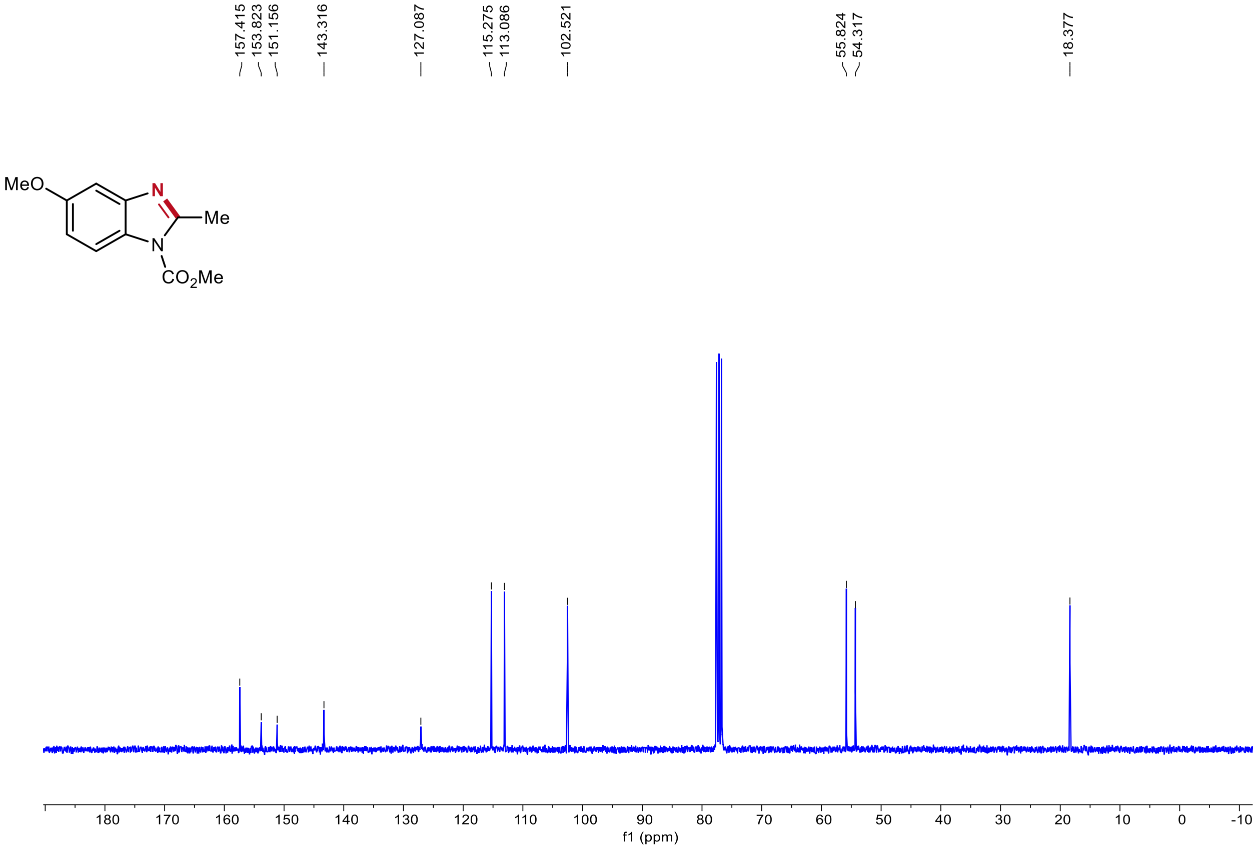

3,4-dimethyl-1*H*-indazole 2c

^{1}H NMR (300 MHz, $CDCl_3$)

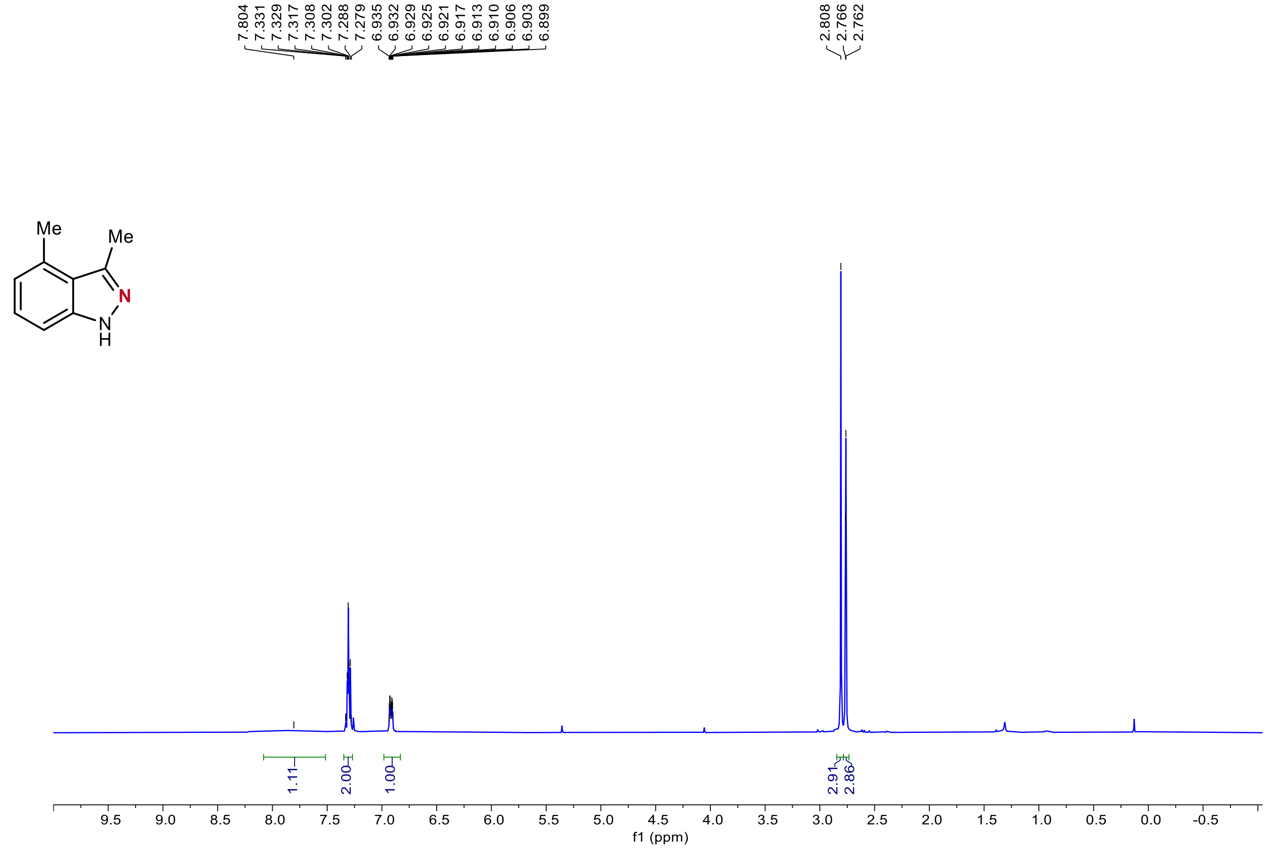

^{13}C NMR (76 MHz, $CDCl_3$)

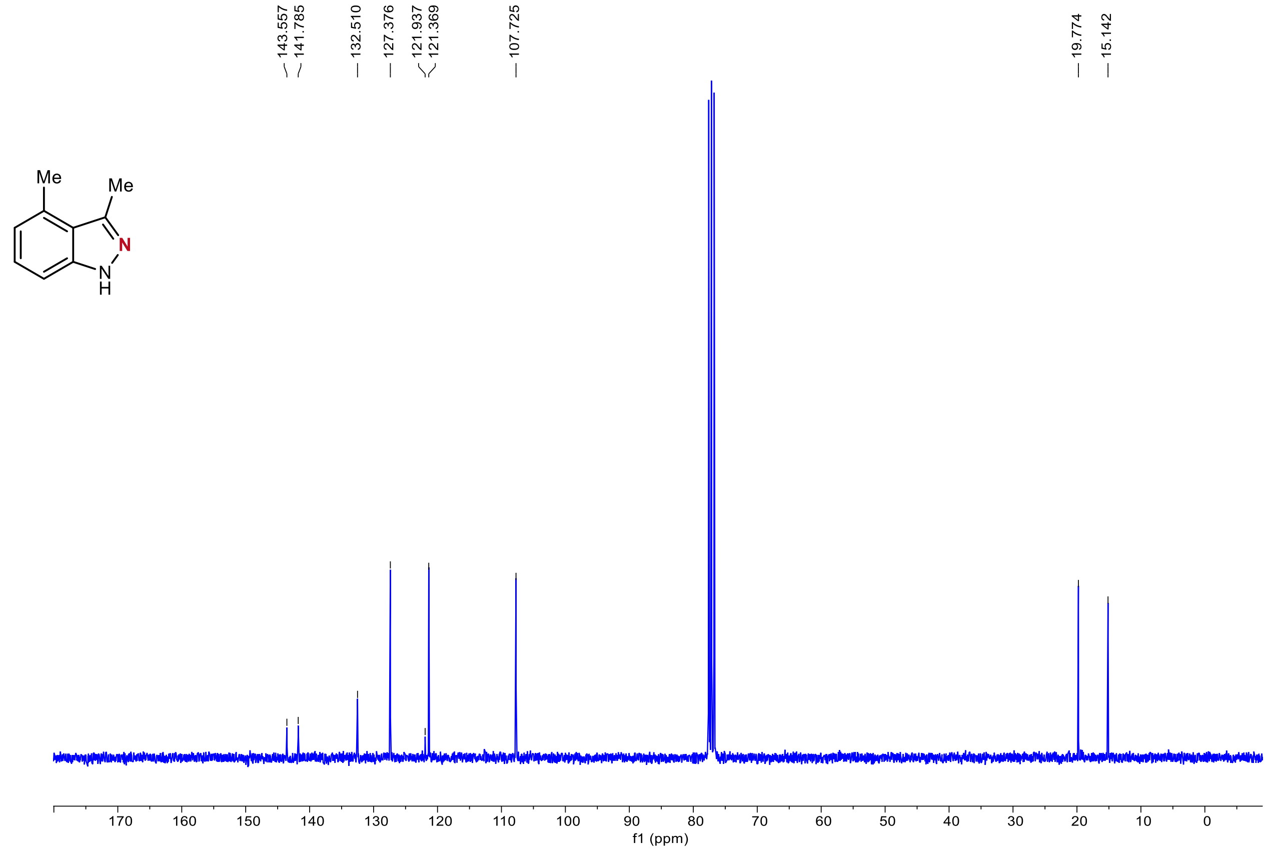

2,4-Dimethyl-1-tosyl-1*H*-benzo[*d*]imidazole Ts-**3c**

^{1}H NMR (400 MHz, $CDCl_3$)

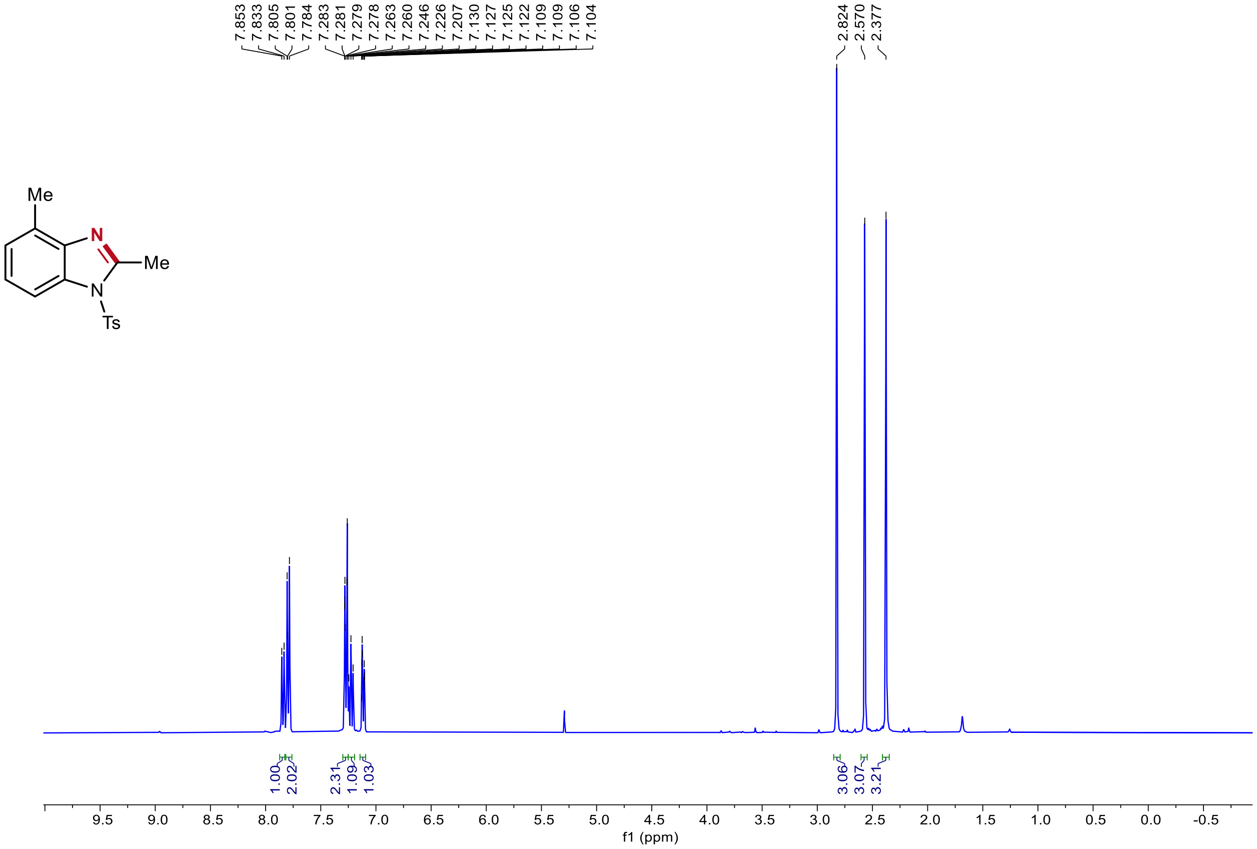

^{13}C NMR (101 MHz, $CDCl_3$)

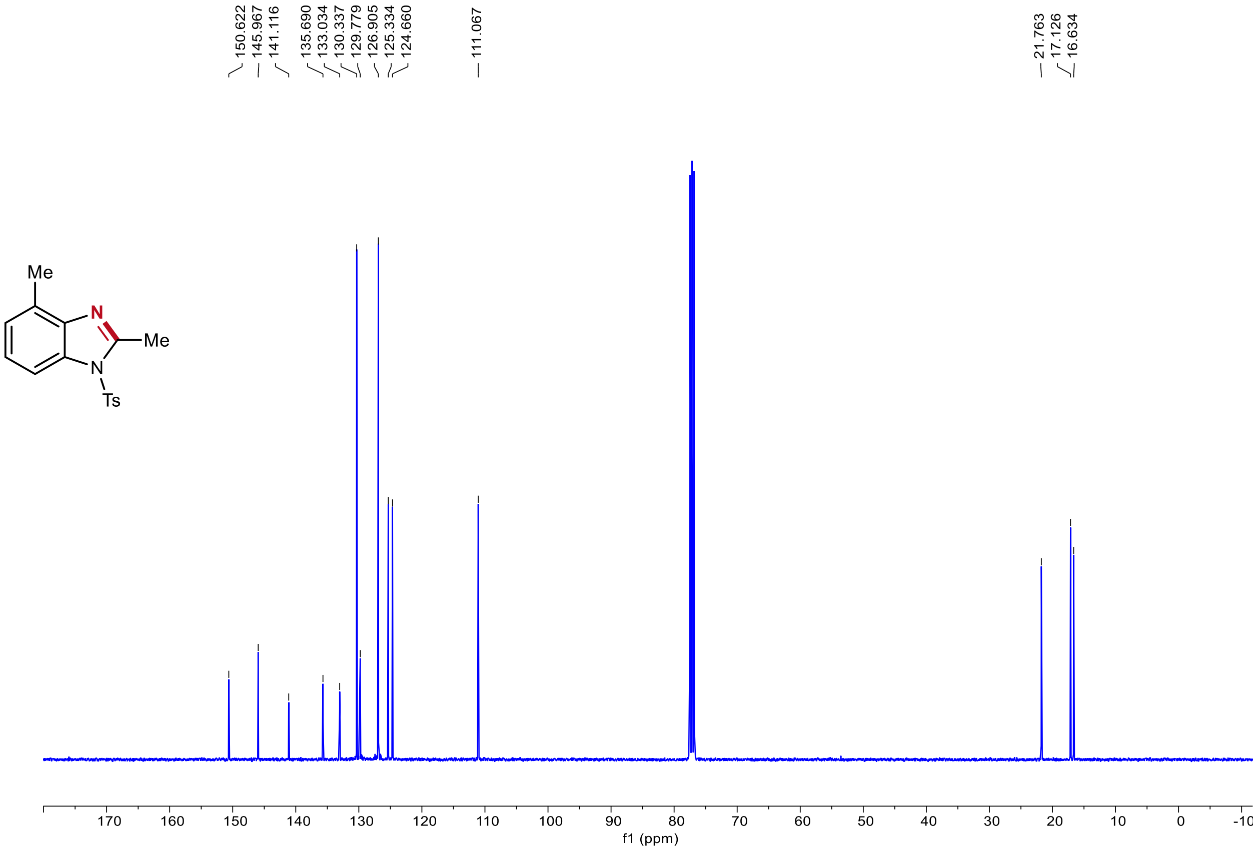

Methyl 3,5-dimethyl-1*H*-indazole-1-carboxylate Moc-**2d**

^{1}H NMR (400 MHz, $CDCl_3$)

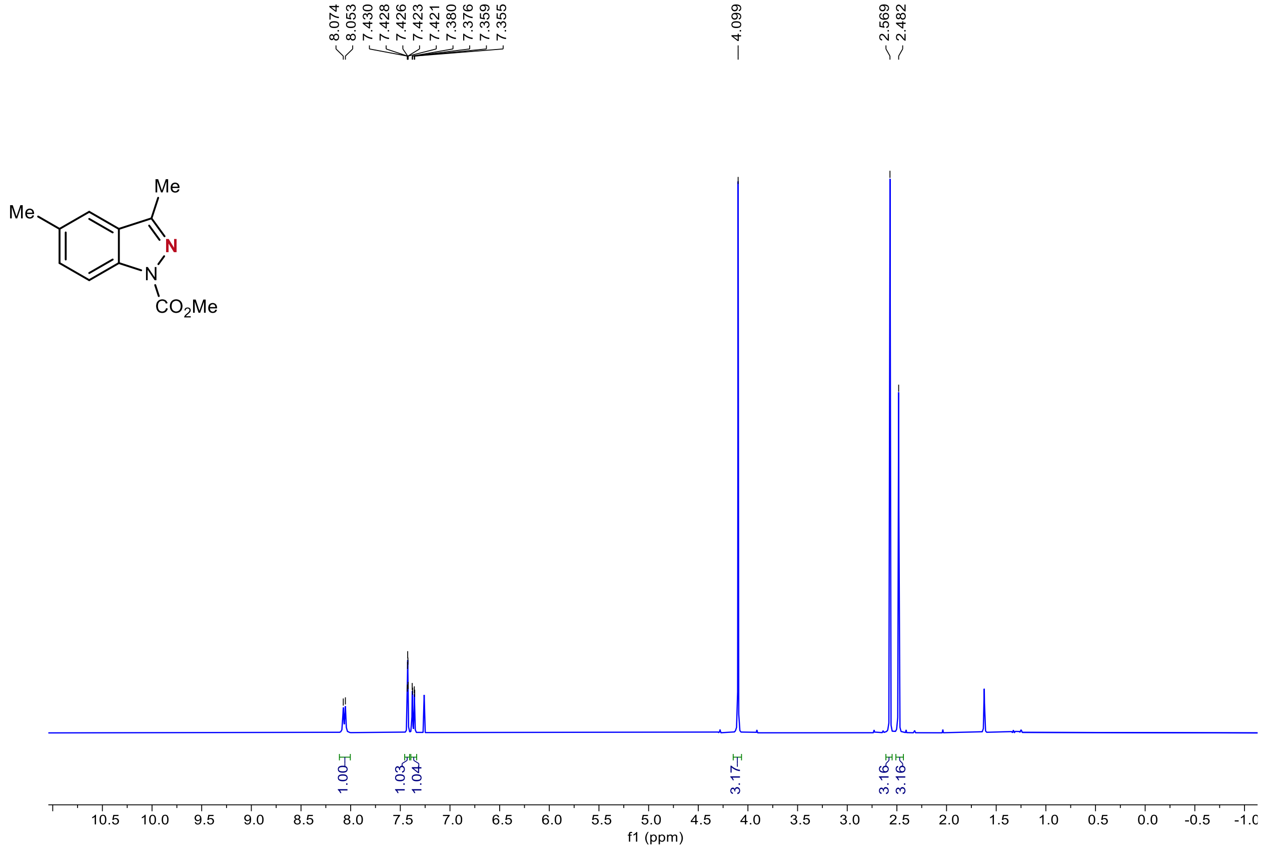

^{13}C NMR (101 MHz, $CDCl_3$)

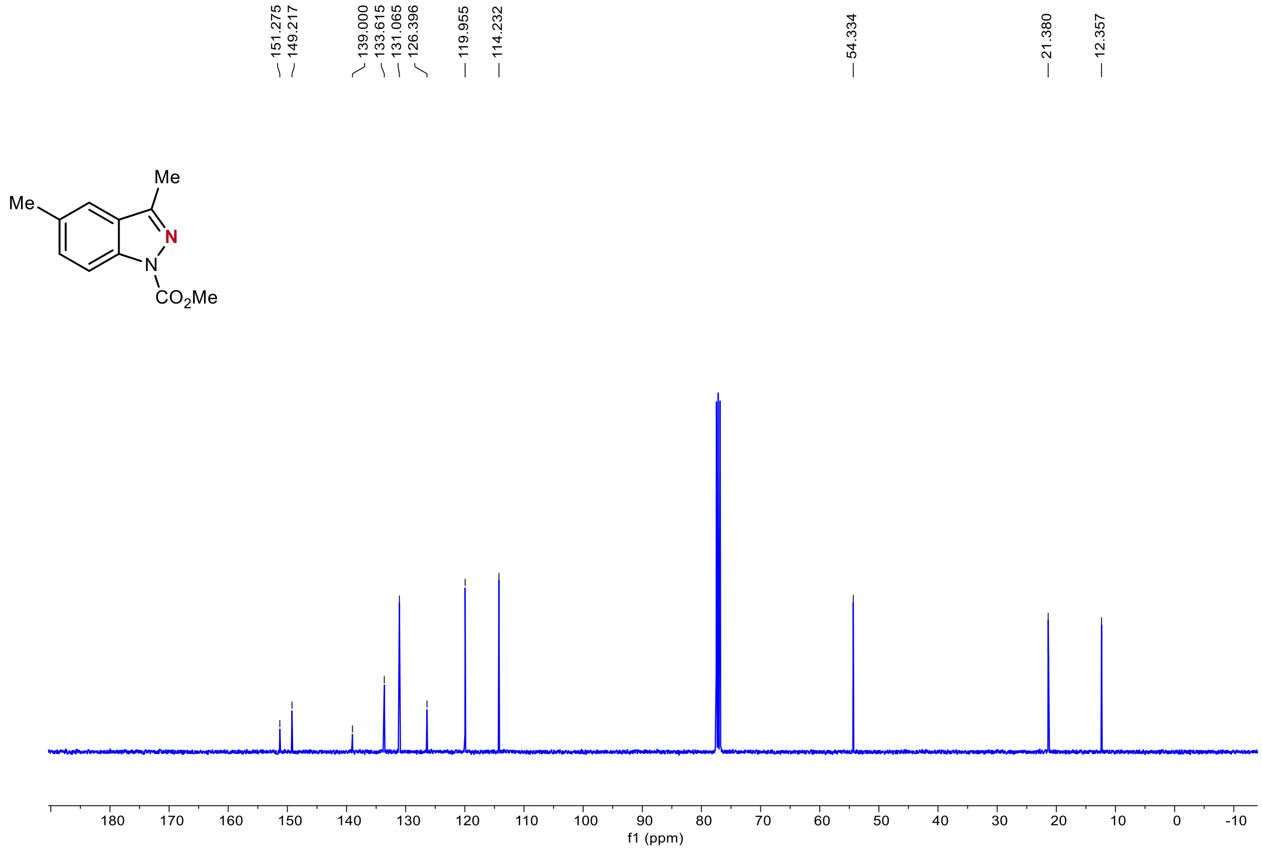

Methyl 2,5-dimethyl-1*H*-benzo[*d*]imidazole-1-carboxylate Moc-**3d**

^{1}H NMR (300 MHz, $CDCl_3$)

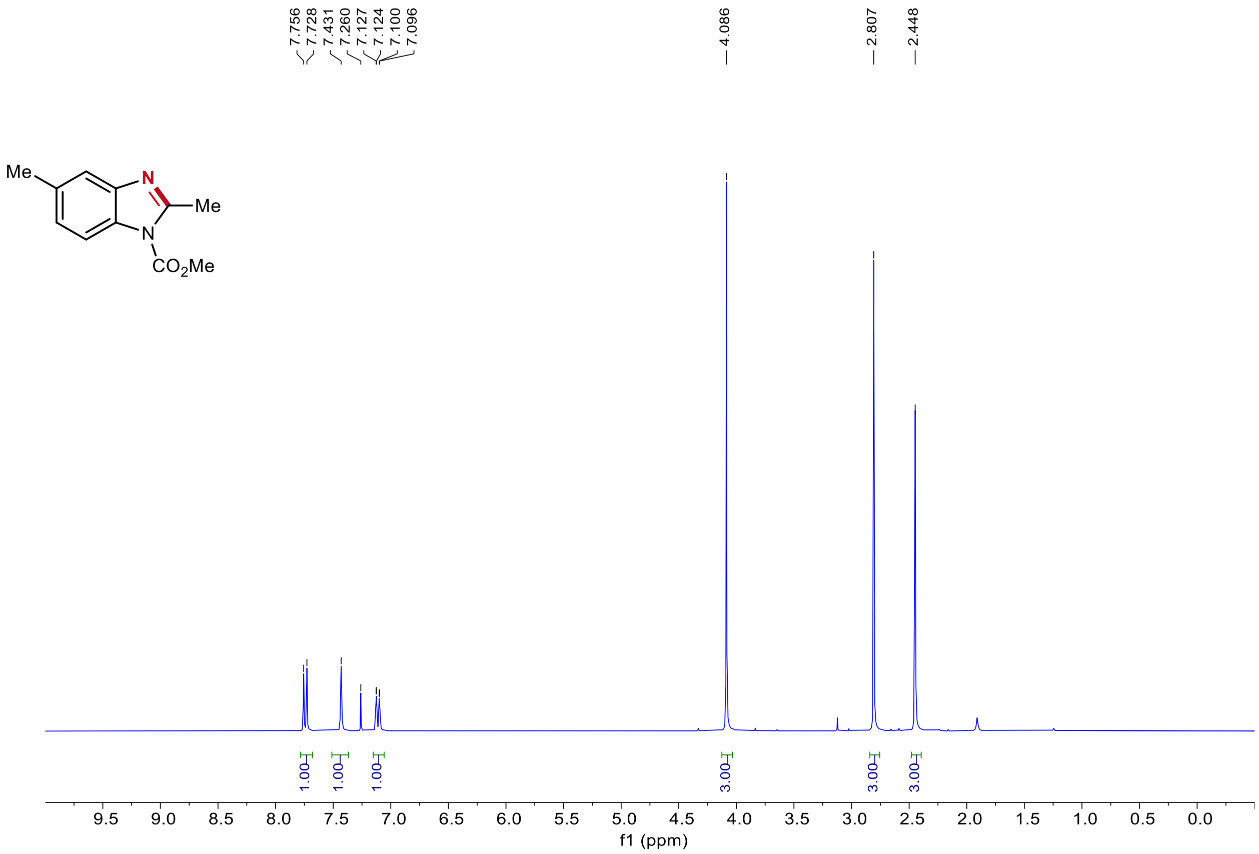

^{13}C NMR (76 MHz, $CDCl_3$)

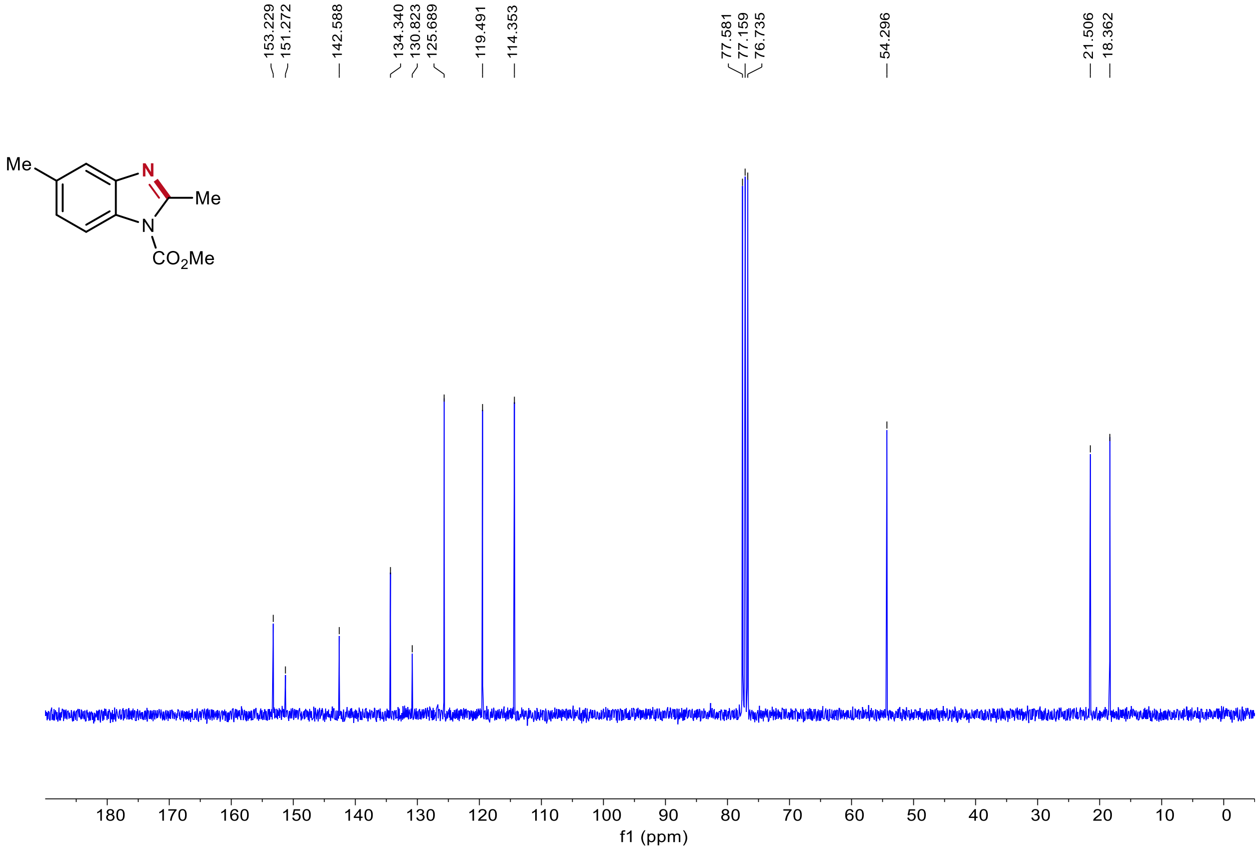

Ethyl 5-cyano-3-methyl-1*H*-indazole-1-carboxylate 2e

^{1}H NMR (400 MHz, $CDCl_3$)

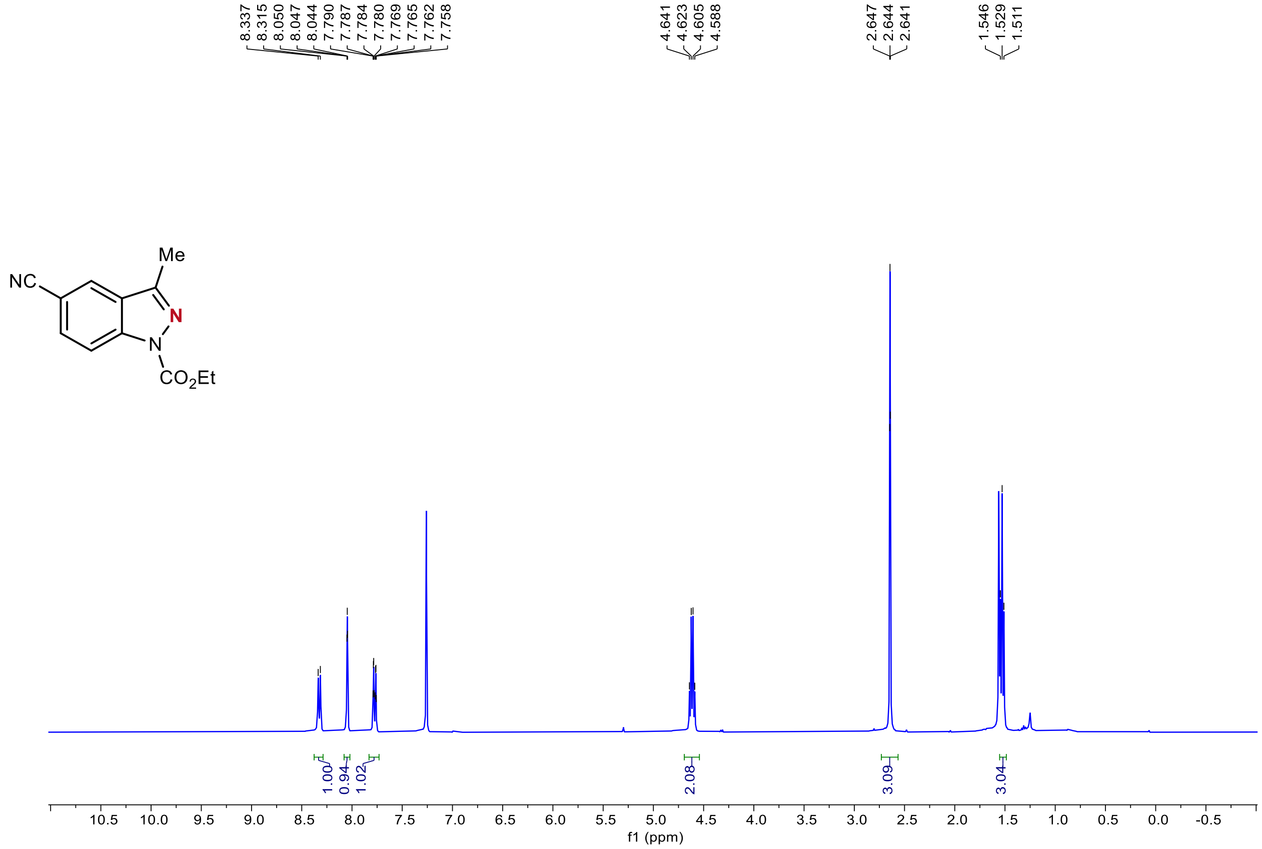

^{13}C NMR (101 MHz, $CDCl_3$)

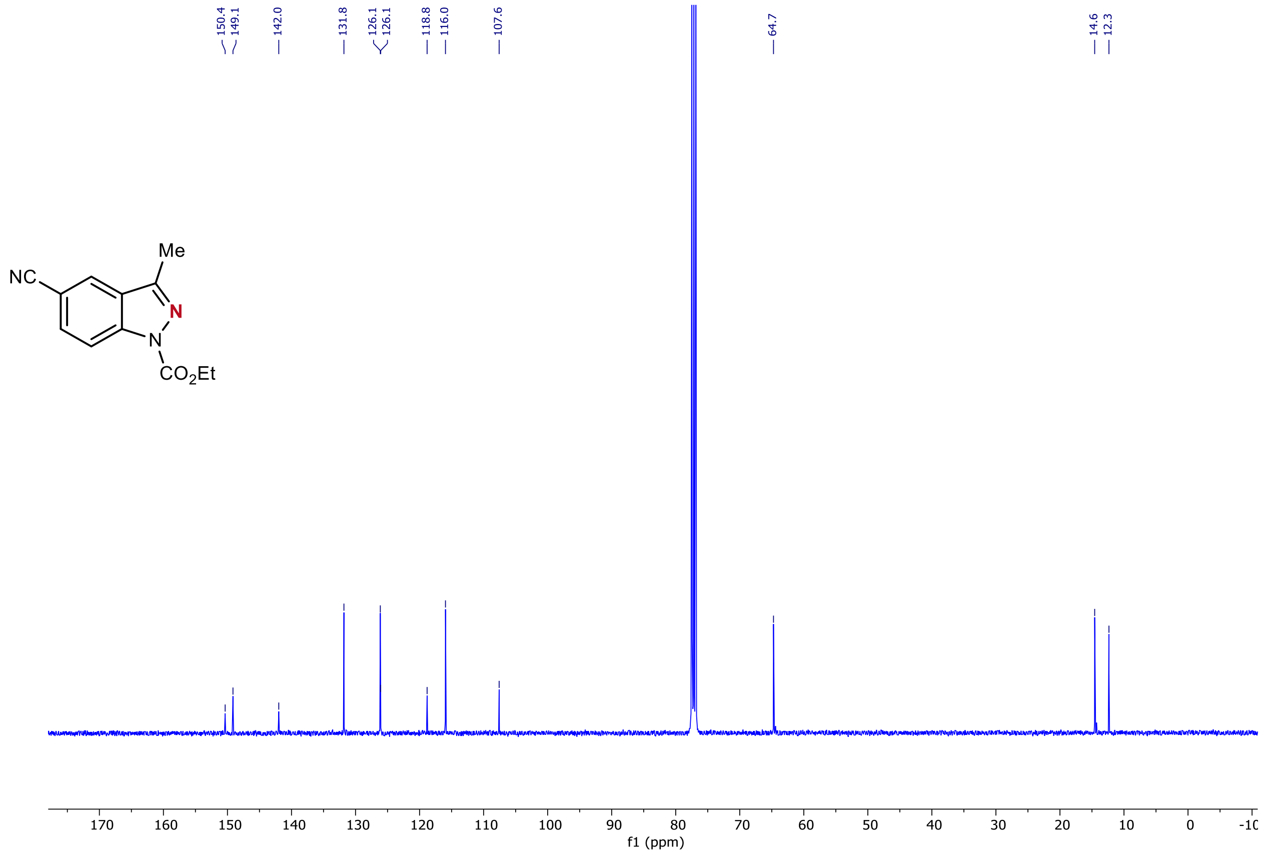

Methyl 6-(benzyloxy)-2-methyl-1*H*-benzo[*d*]imidazole-1-carboxylate Moc-**3f**

^{1}H NMR (400 MHz, $CDCl_3$)

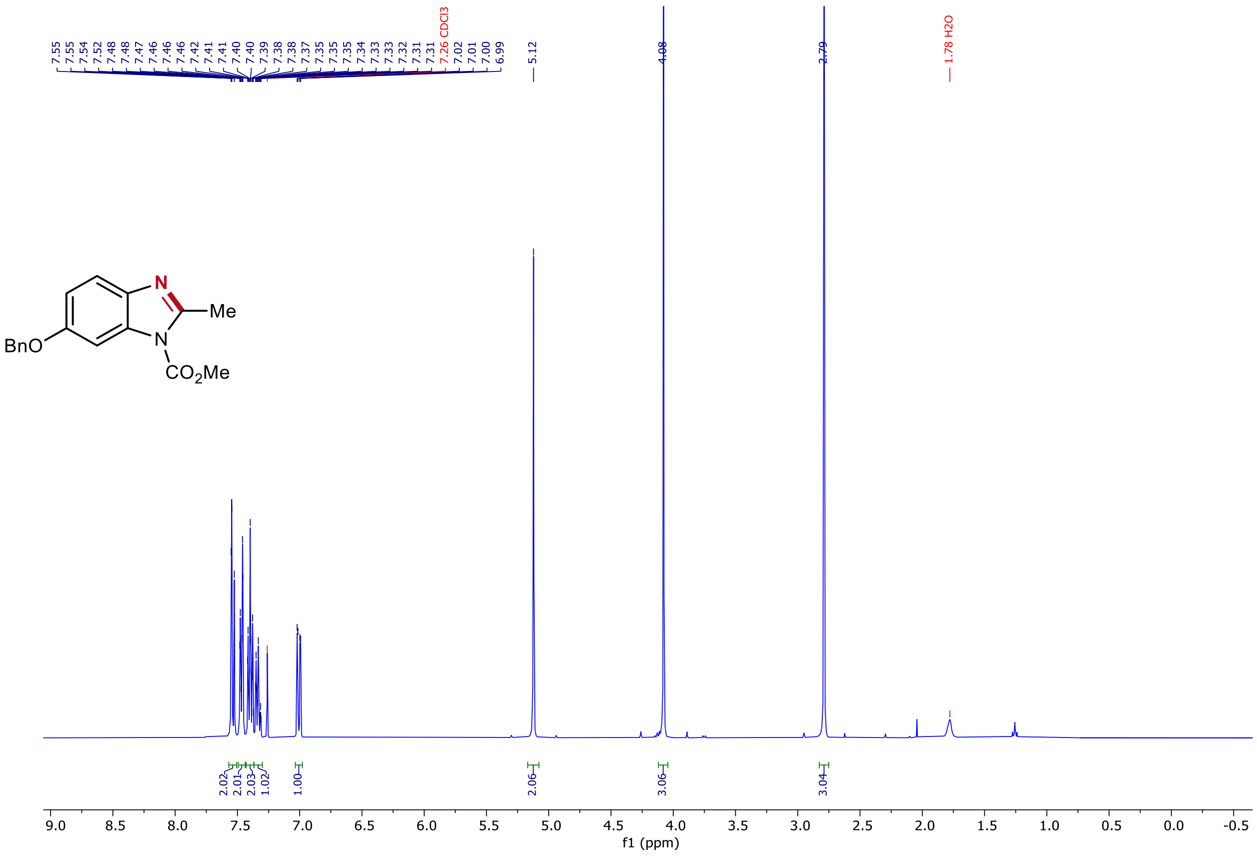

^{13}C NMR (101 MHz, $CDCl_3$)

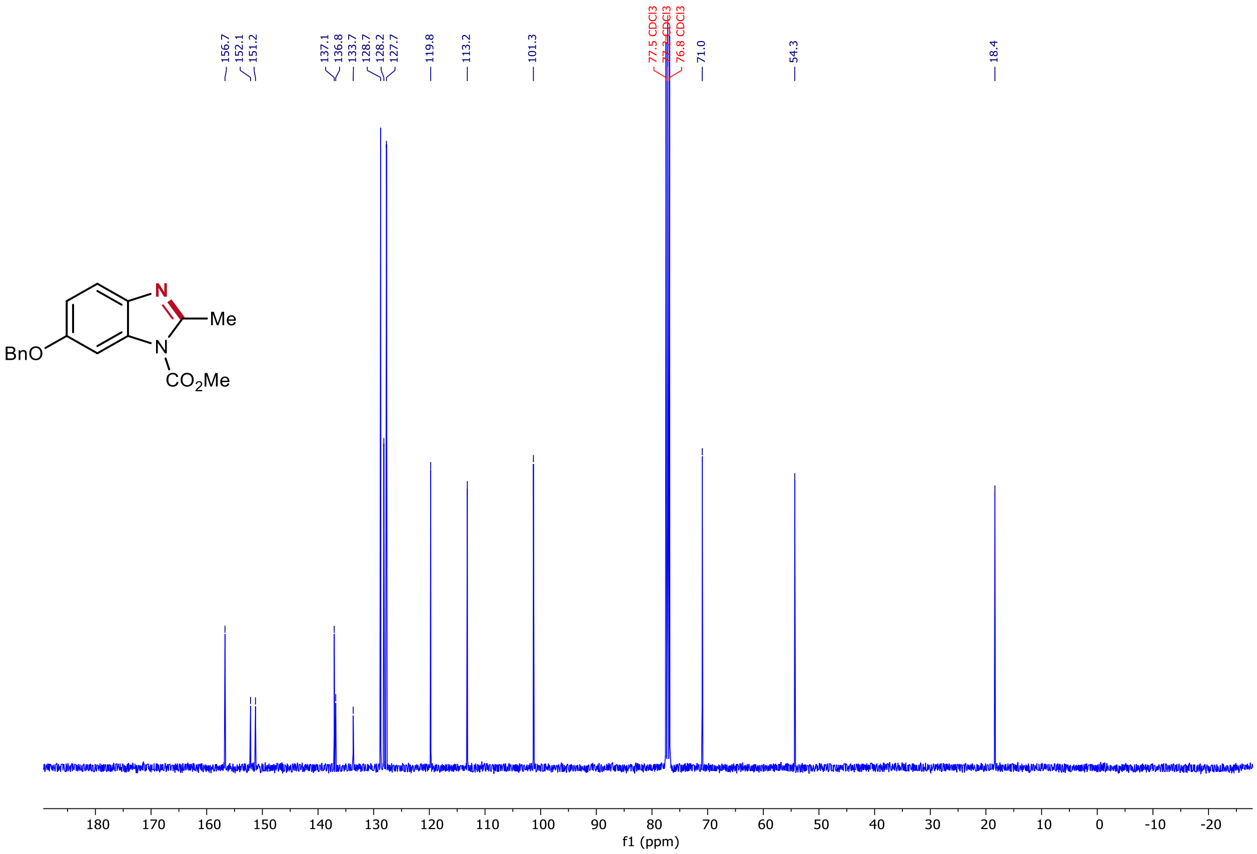

***tert*-Butyl 5-bromo-6-chloro-3-methyl-1*H*-indazole-1-carboxylate** Boc-**2g**

^{1}H NMR (400 MHz, $CDCl_3$)

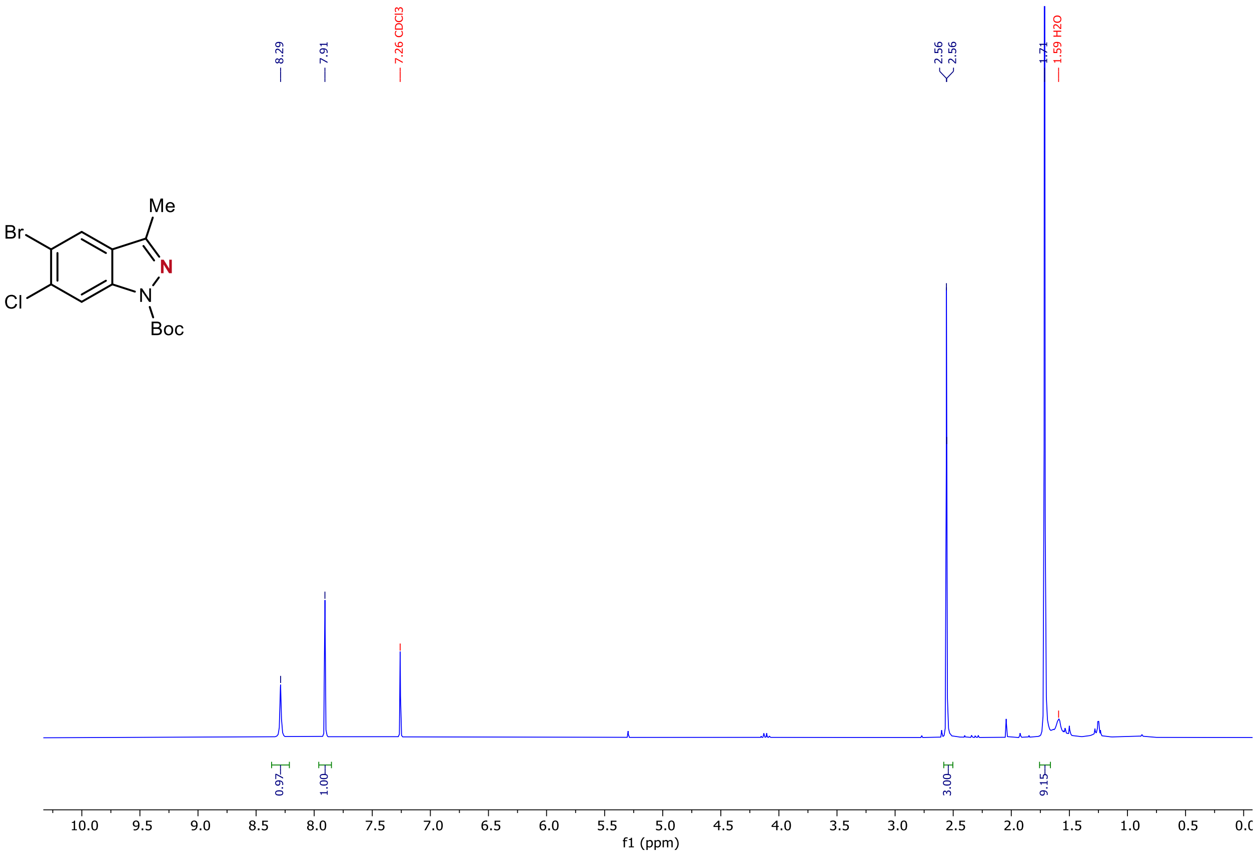

^{13}C NMR (101 MHz, $CDCl_3$)

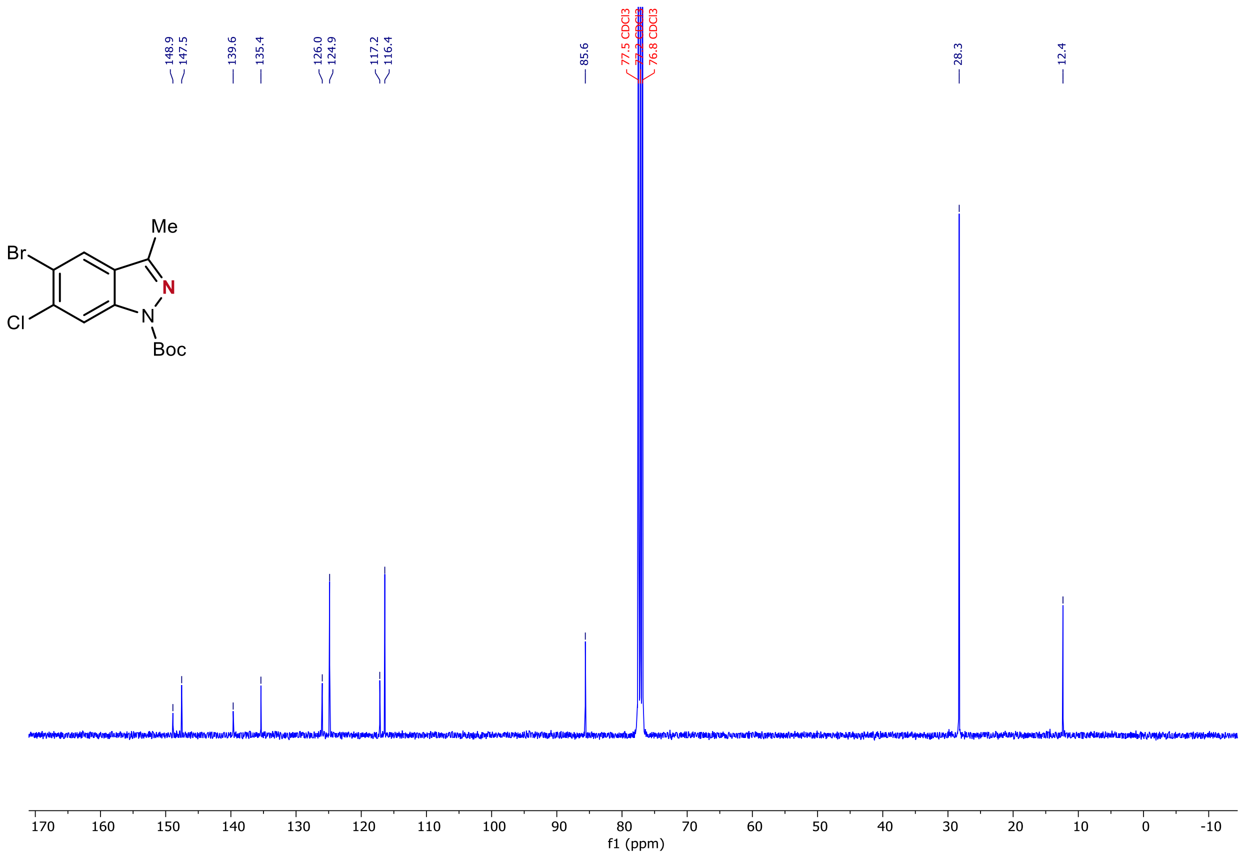

Methyl 3-isobutyl-1*H*-indazole-1-carboxylate Moc-**2h**

^{1}H NMR (400 MHz, $CDCl_3$)

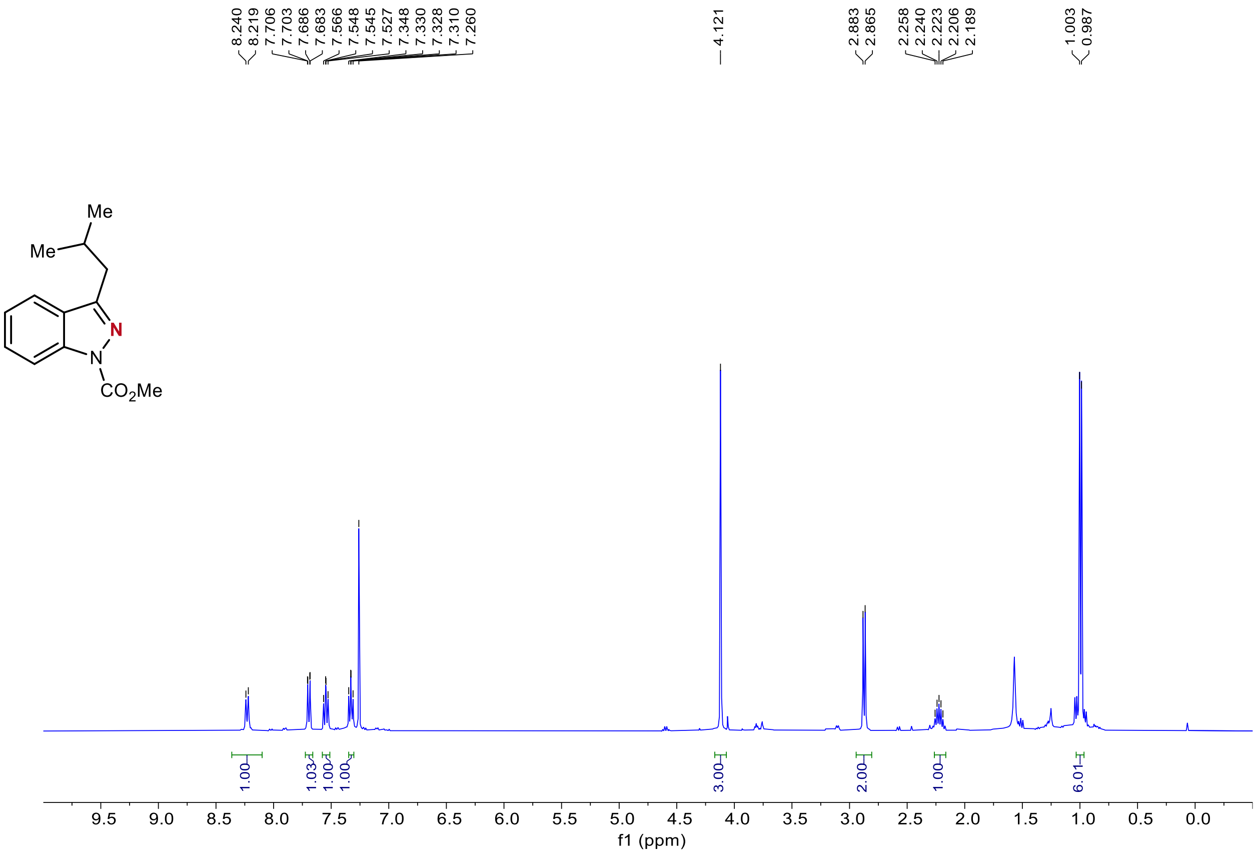

^{13}C NMR (76 MHz, $CDCl_3$)

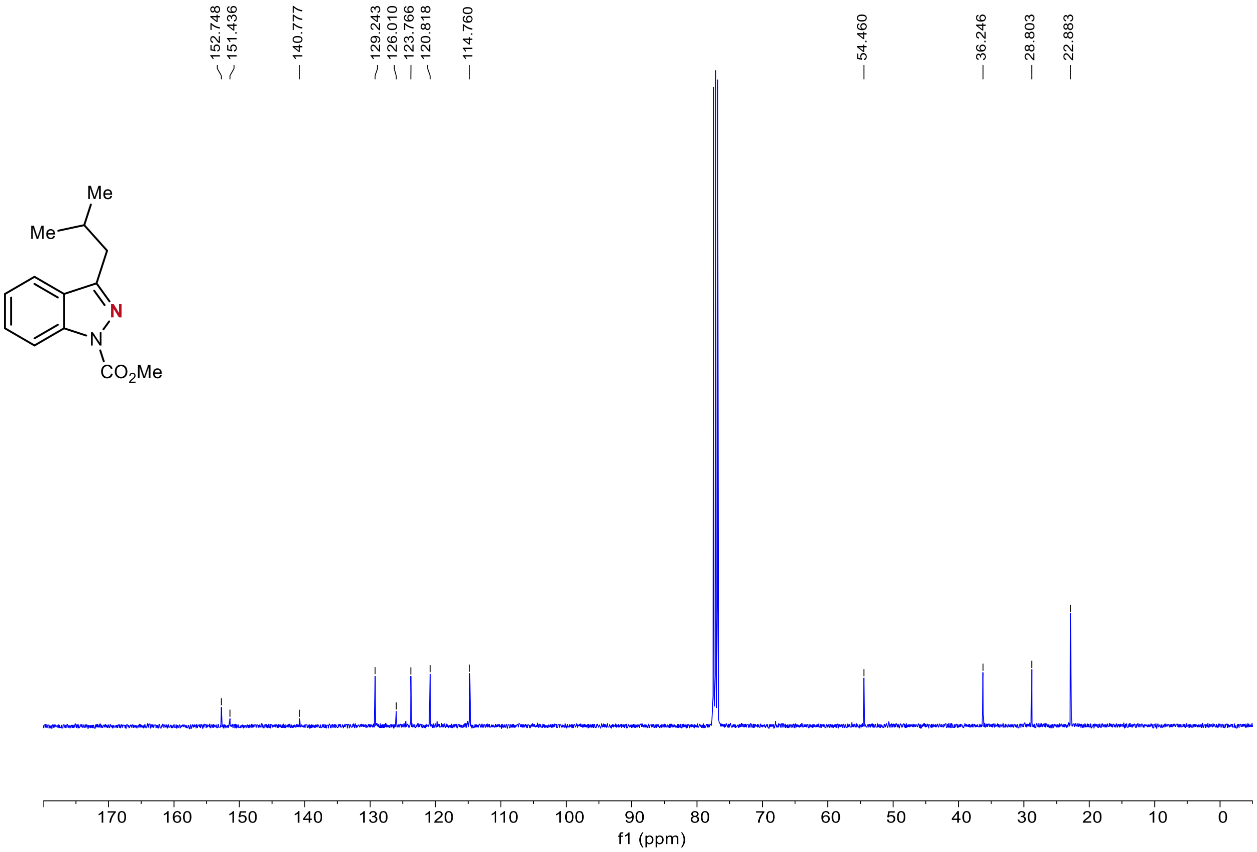

Methyl 2-isobutyl-1*H*-benzo[*d*]imidazole-1-carboxylate Moc-3h

^{1}H NMR (400 MHz, $CDCl_3$)

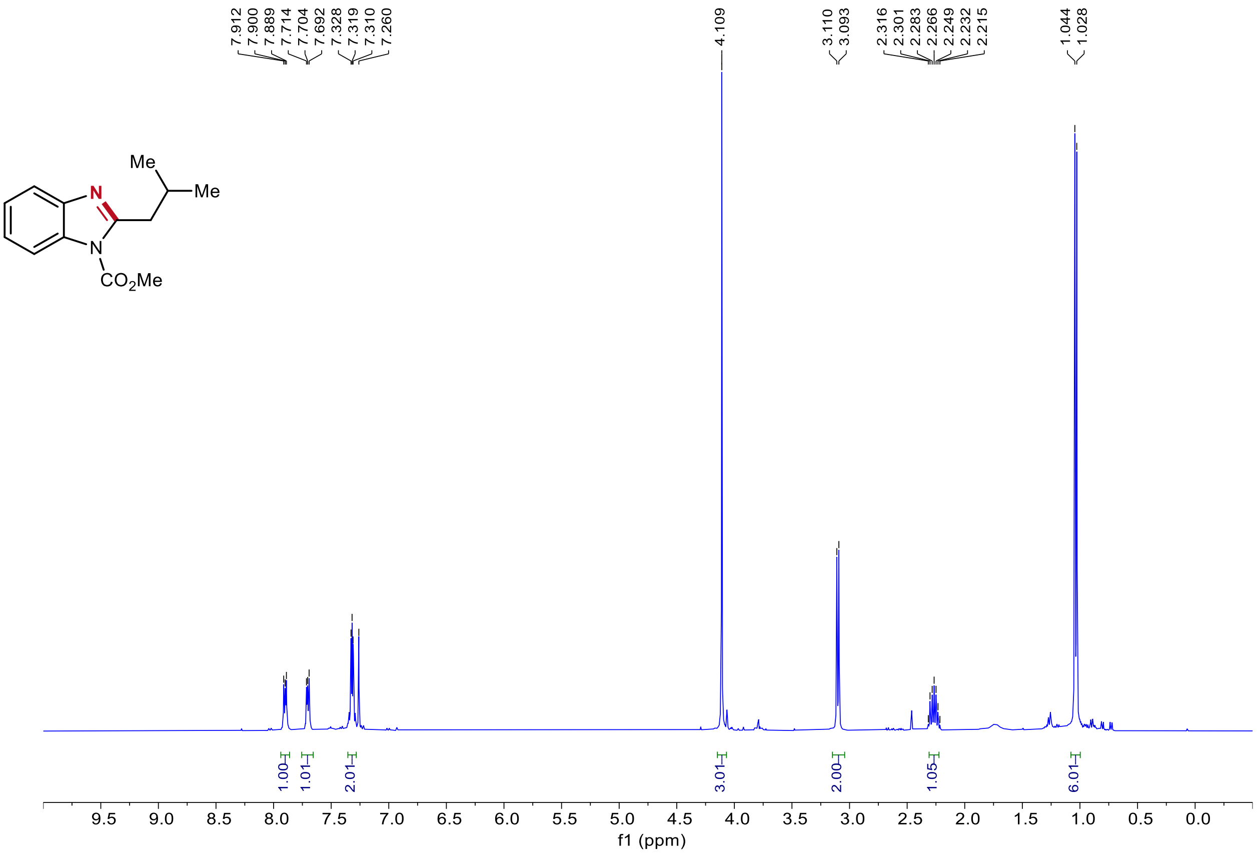

^{13}C NMR (76 MHz, $CDCl_3$)

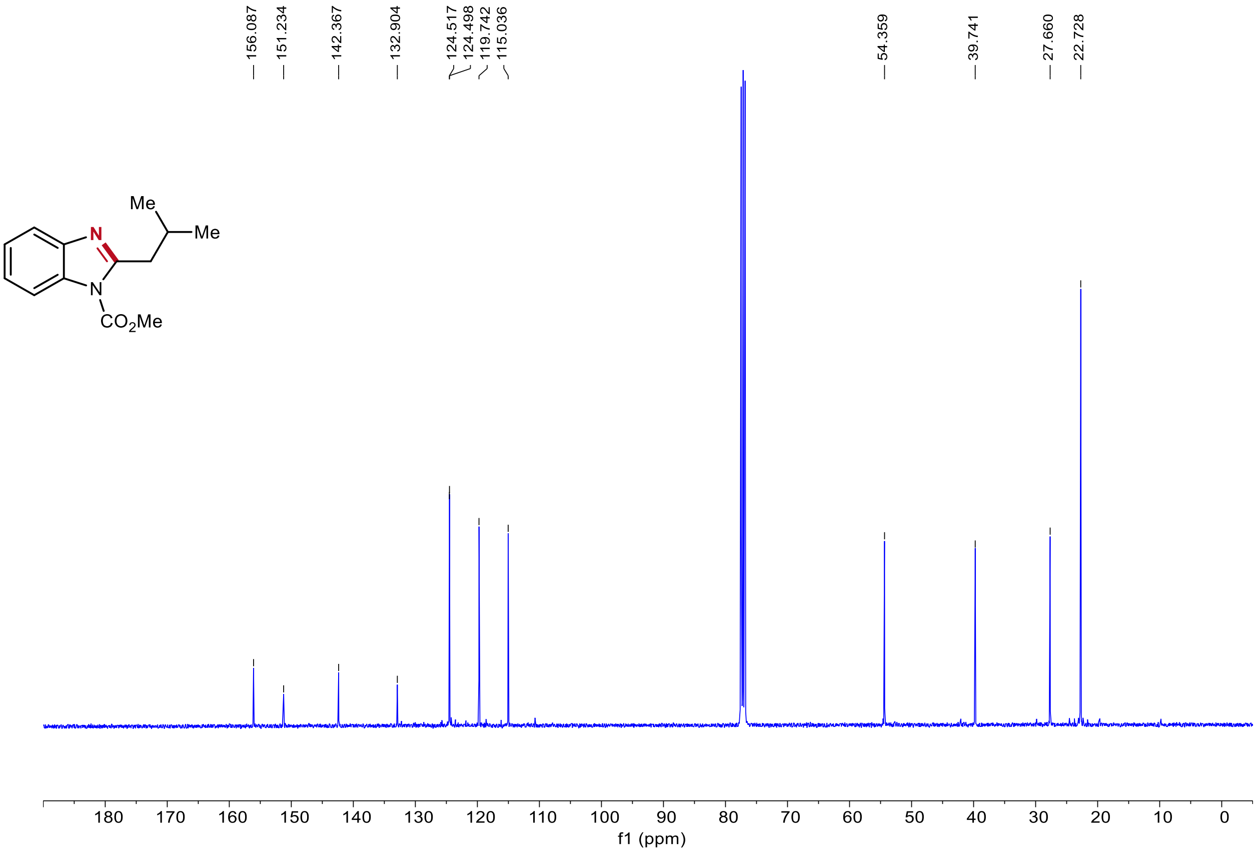

3-Cyclohexyl-1*H*-indazole 2i

^{1}H NMR (300 MHz, $CDCl_3$)

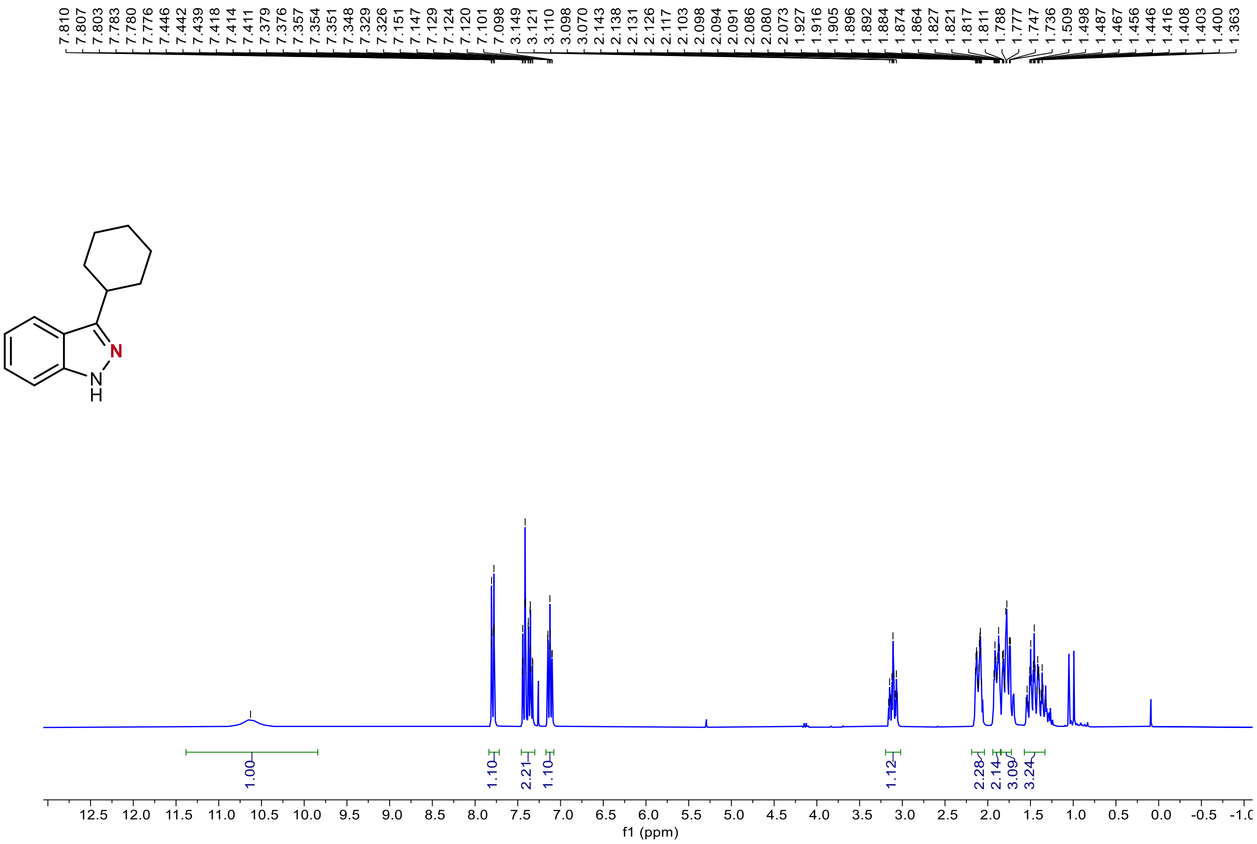

^{13}C NMR (76 MHz, $CDCl_3$)

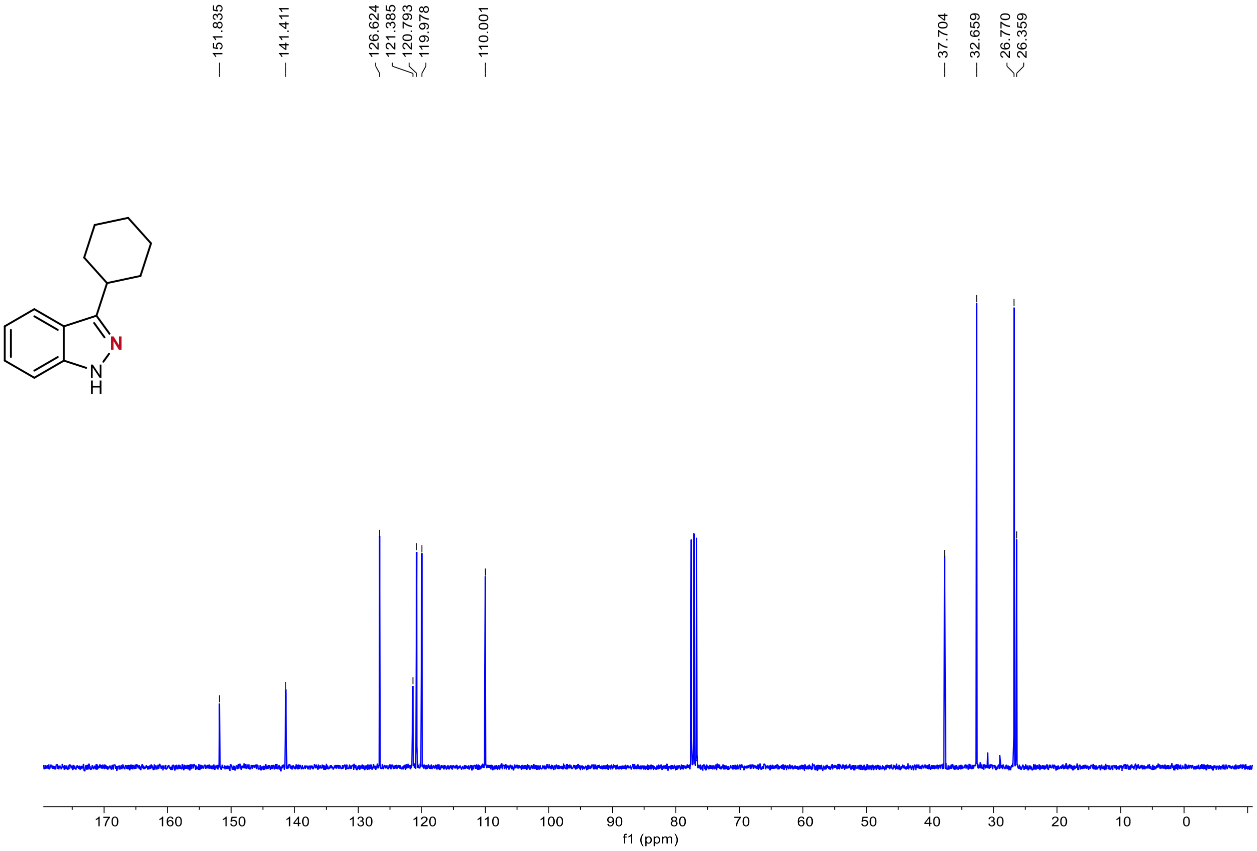

2-Cyclohexyl-1-tosyl-1*H*-benzo[*d*]imidazole Ts-**3i**

^{1}H NMR (300 MHz, $CDCl_3$)

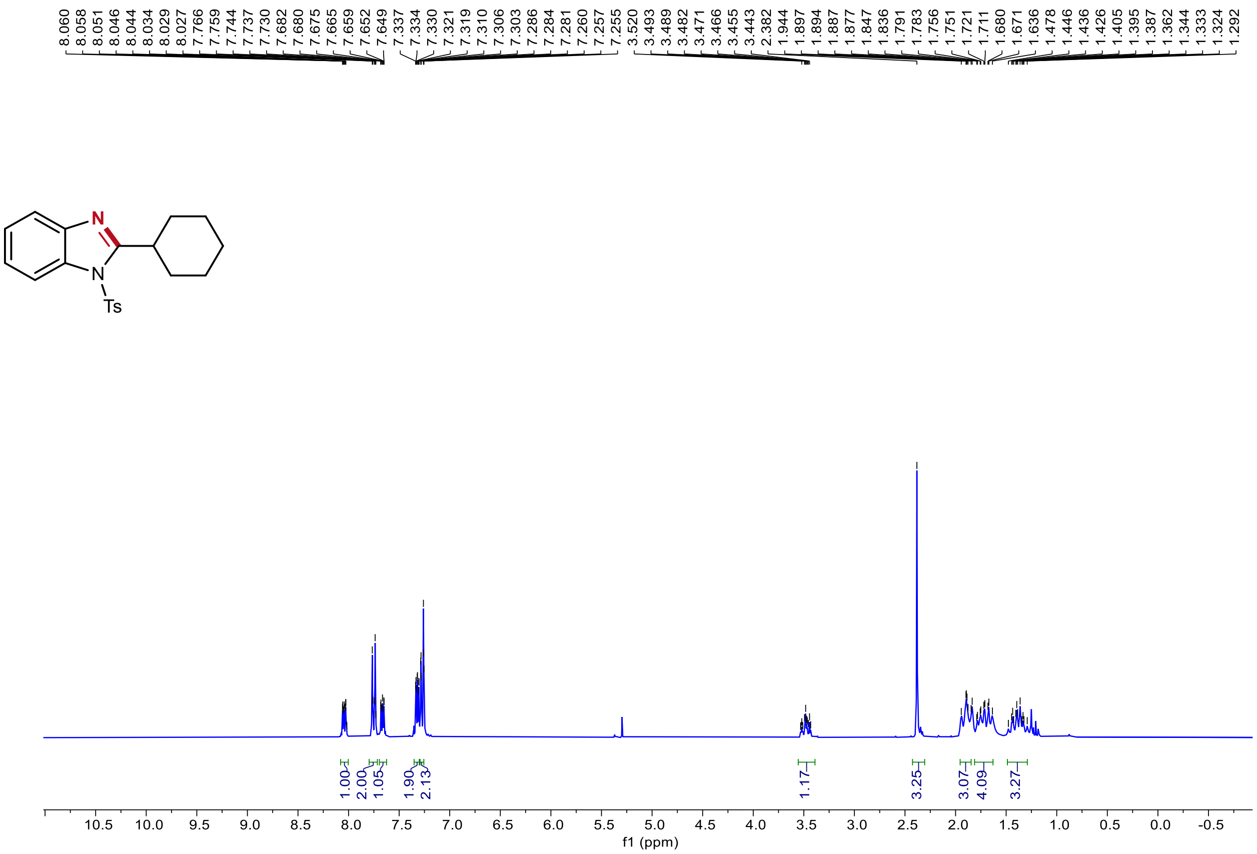

^{13}C NMR (76 MHz, $CDCl_3$)

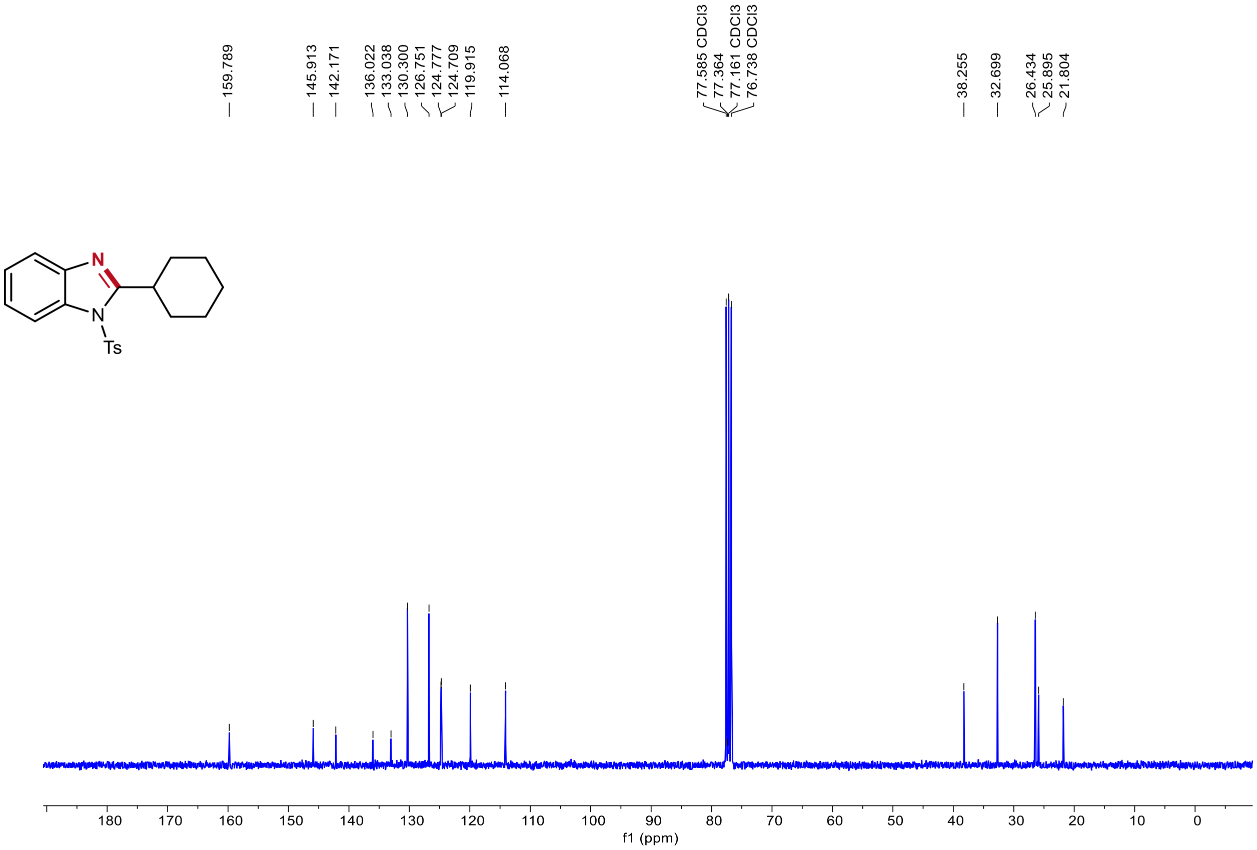

3-(Adamantan-2-yl)-1*H*-indazole 2j

^{1}H NMR (300 MHz, $CDCl_3$)

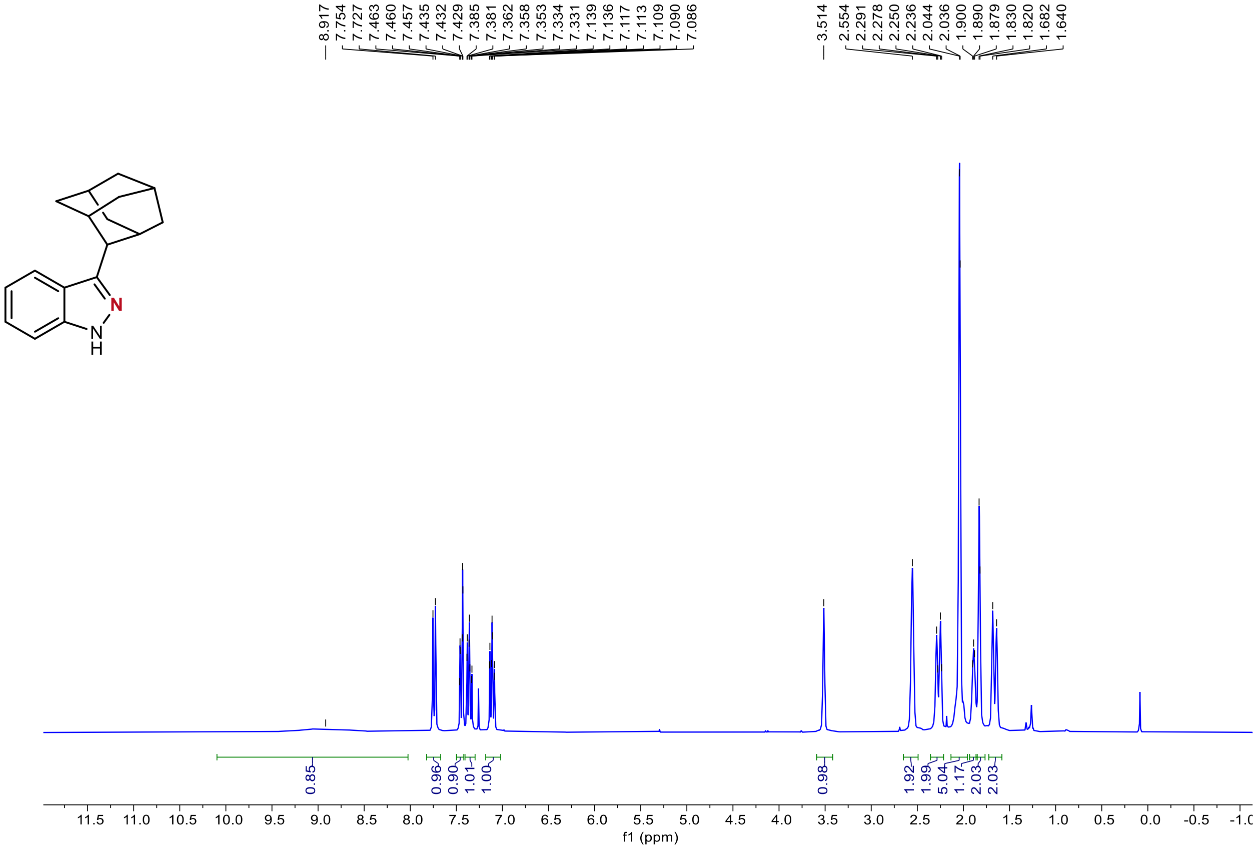

^{13}C NMR (76 MHz, $CDCl_3$)

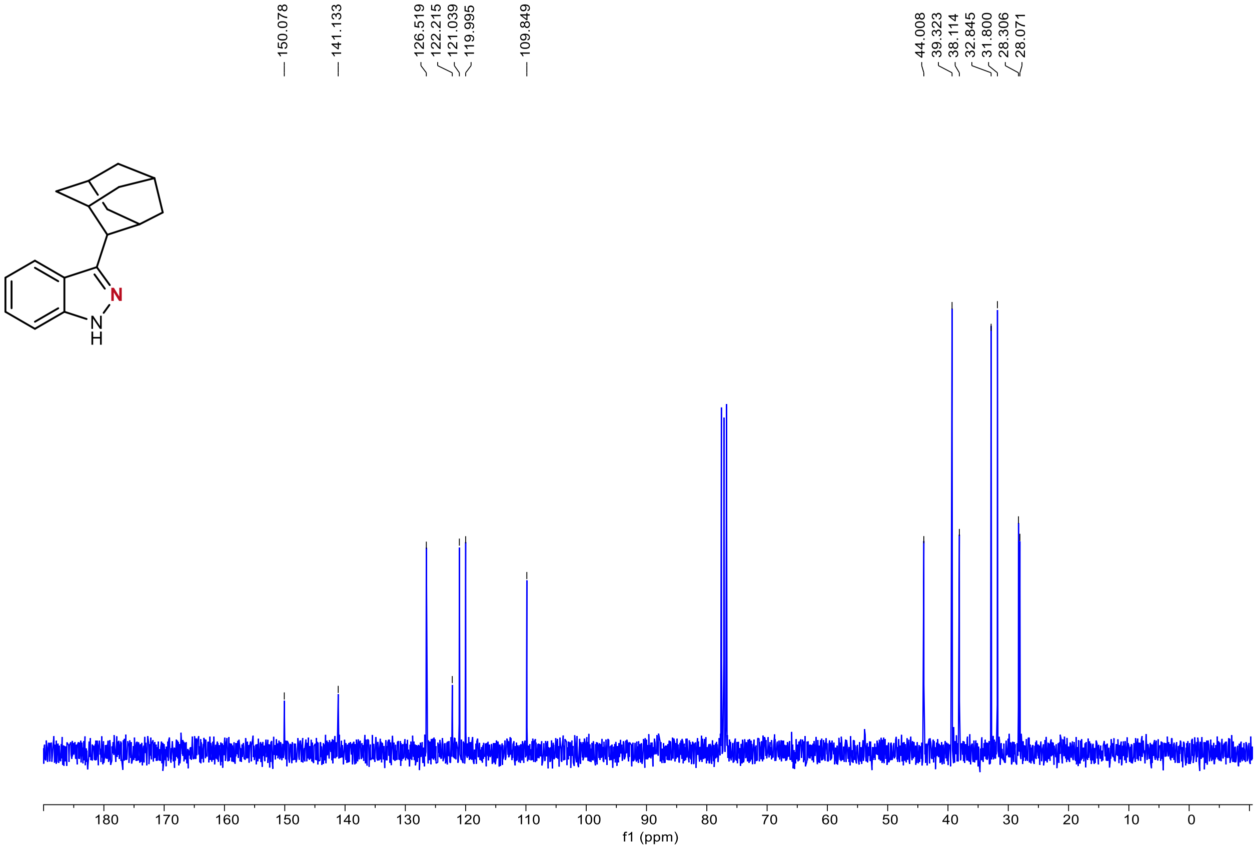

3-(2-Phenylpropan-2-yl)-1*H*-indazole 2k

^{1}H NMR (300 MHz, $CDCl_3$)

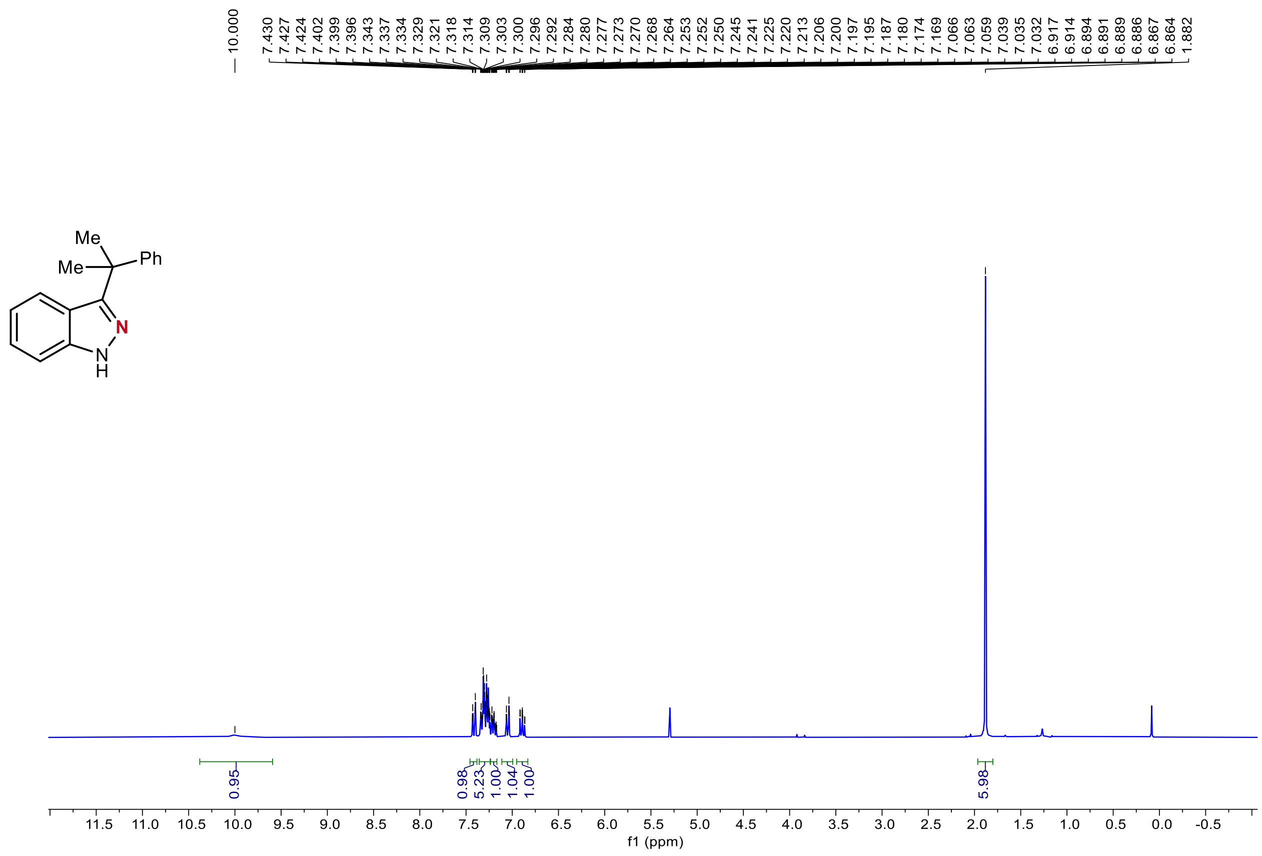

^{13}C NMR (76 MHz, $CDCl_3$)

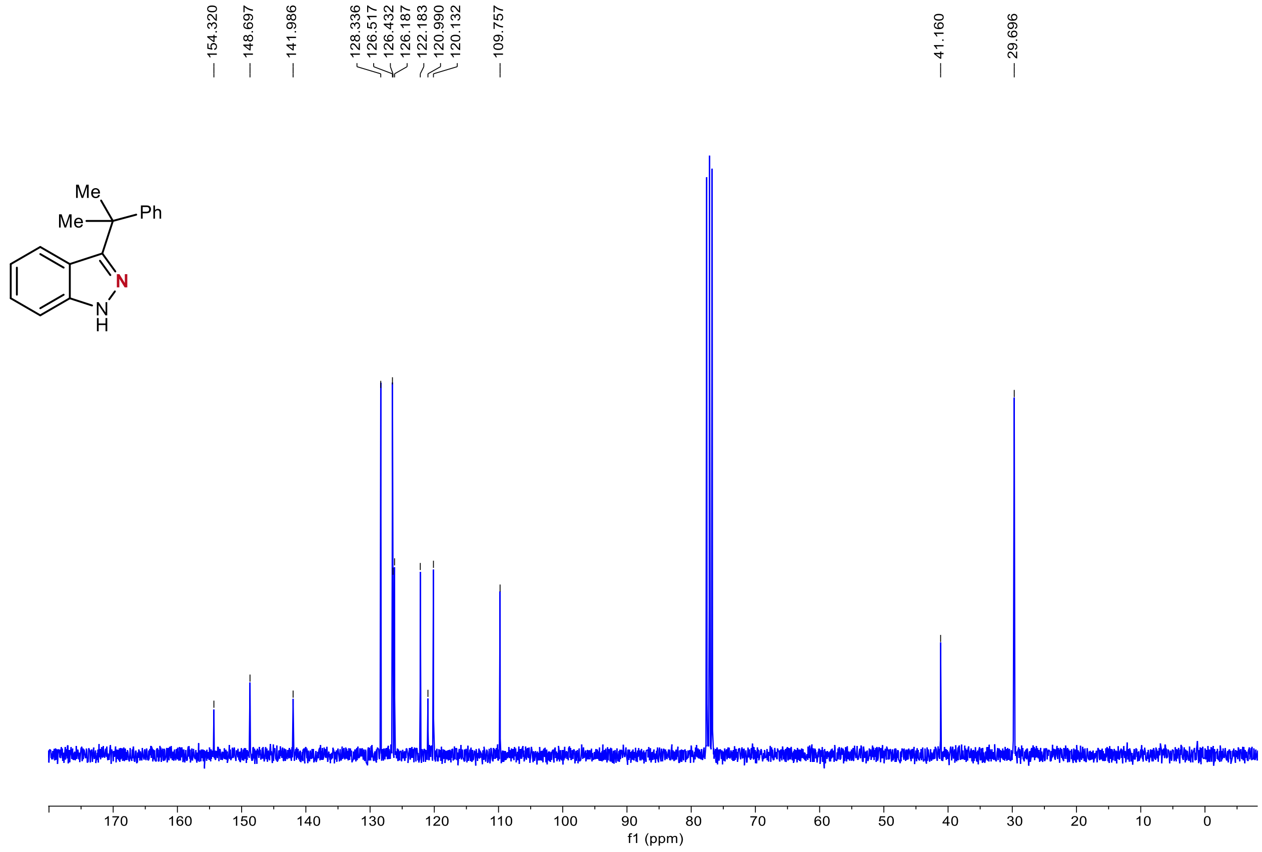

2-(2-Phenylpropan-2-yl)-1-tosyl-1*H*-benzo[*d*]imidazole Ts-3k

^{1}H NMR (400 MHz, $CDCl_3$)

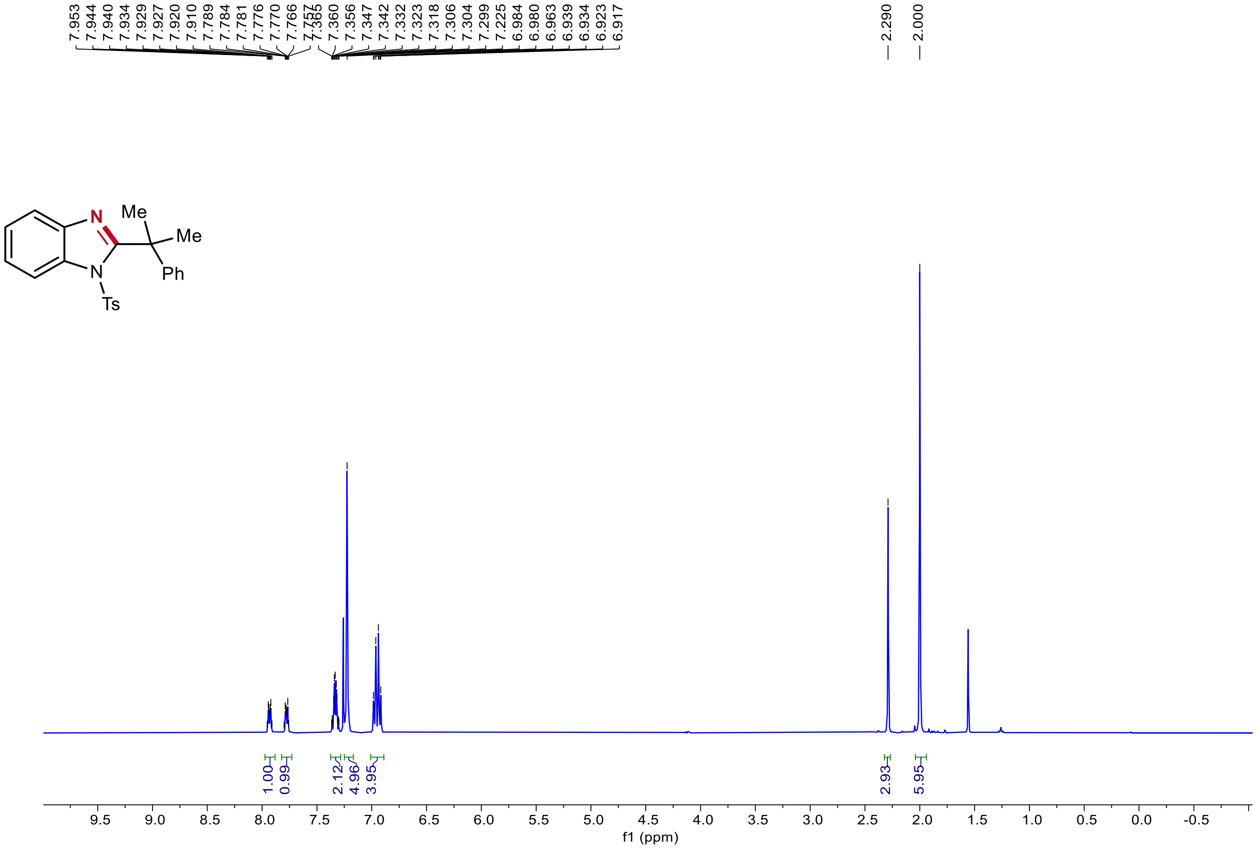

^{13}C NMR (101 MHz, $CDCl_3$)

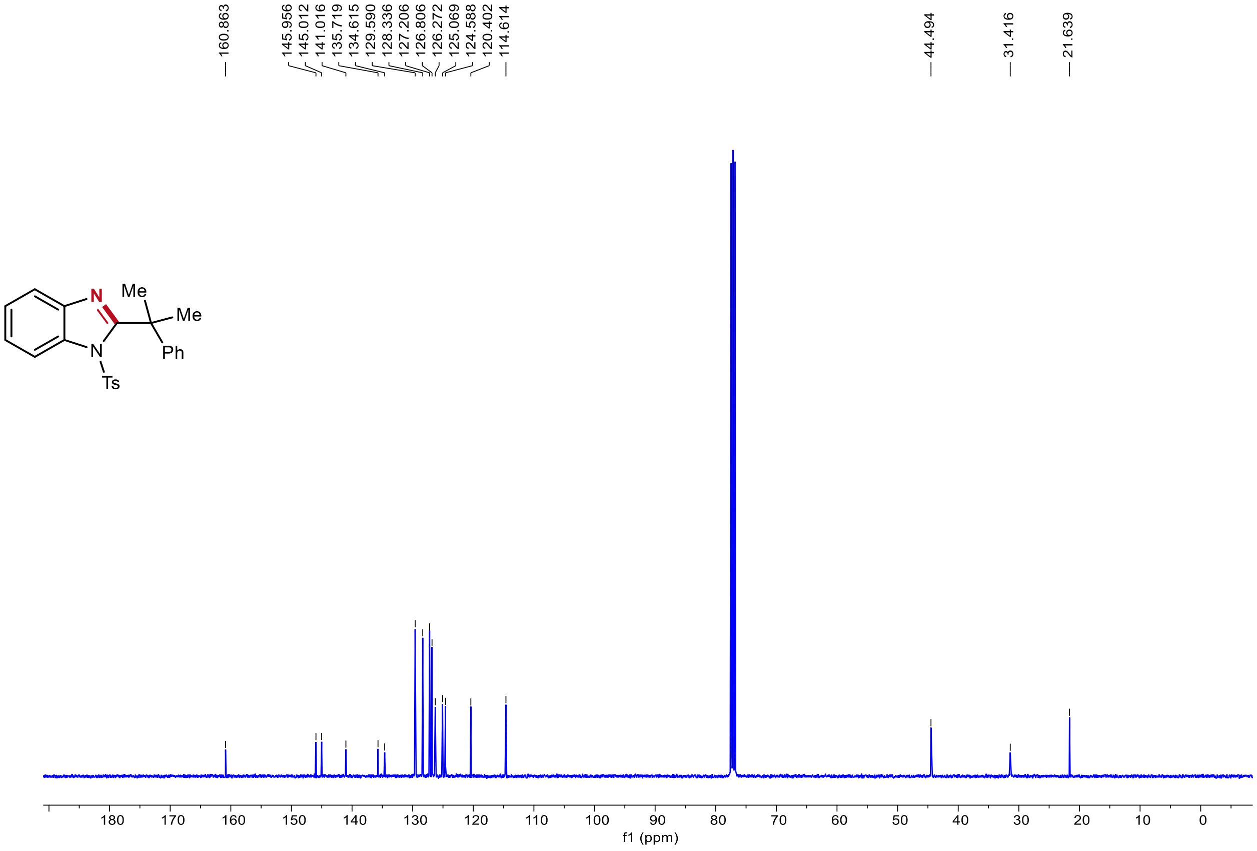

3-Phenyl-1*H*-indazole 2l

^{1}H NMR (300 MHz, $CDCl_3$)

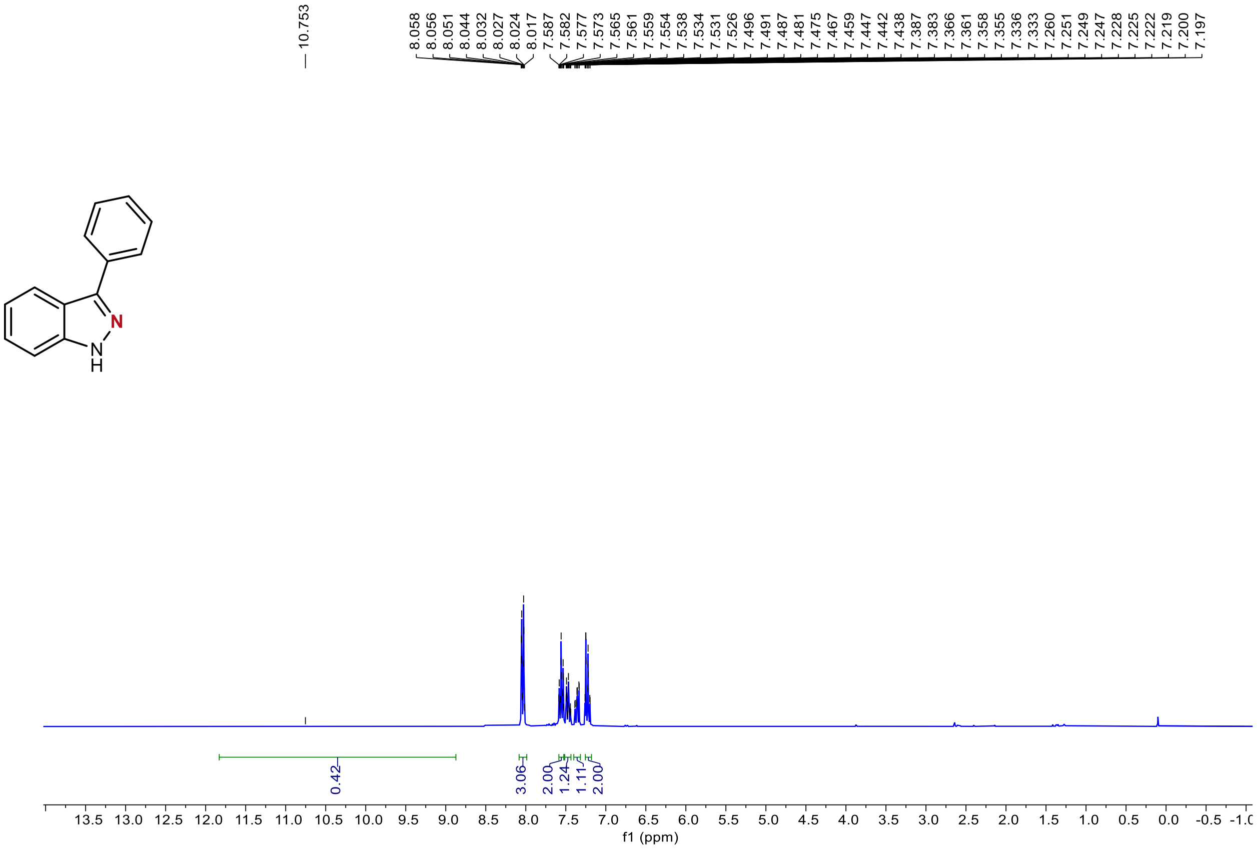

^{13}C NMR (76 MHz, $CDCl_3$)

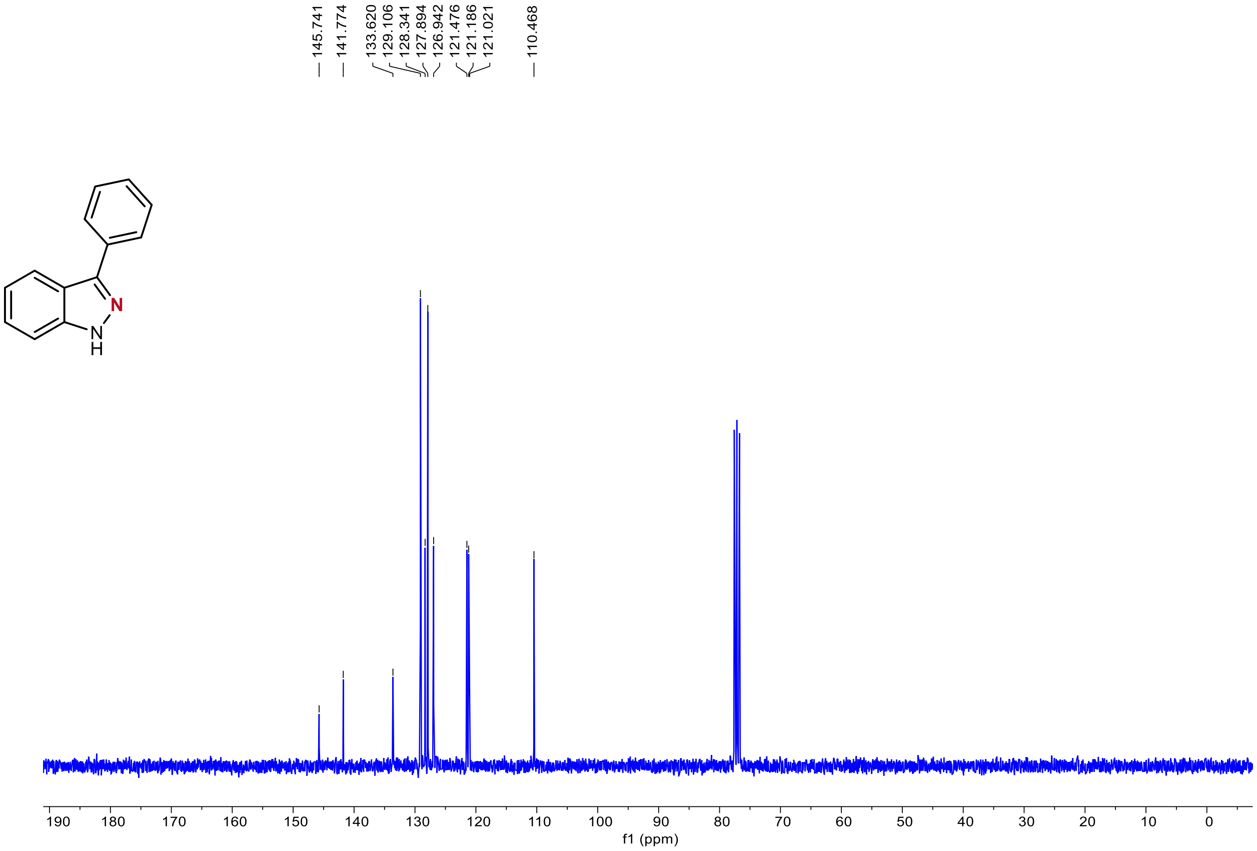

3-Phenyl-1-tosyl-1*H*-indazole Ts-2l

^{1}H NMR (400 MHz, $CDCl_3$)

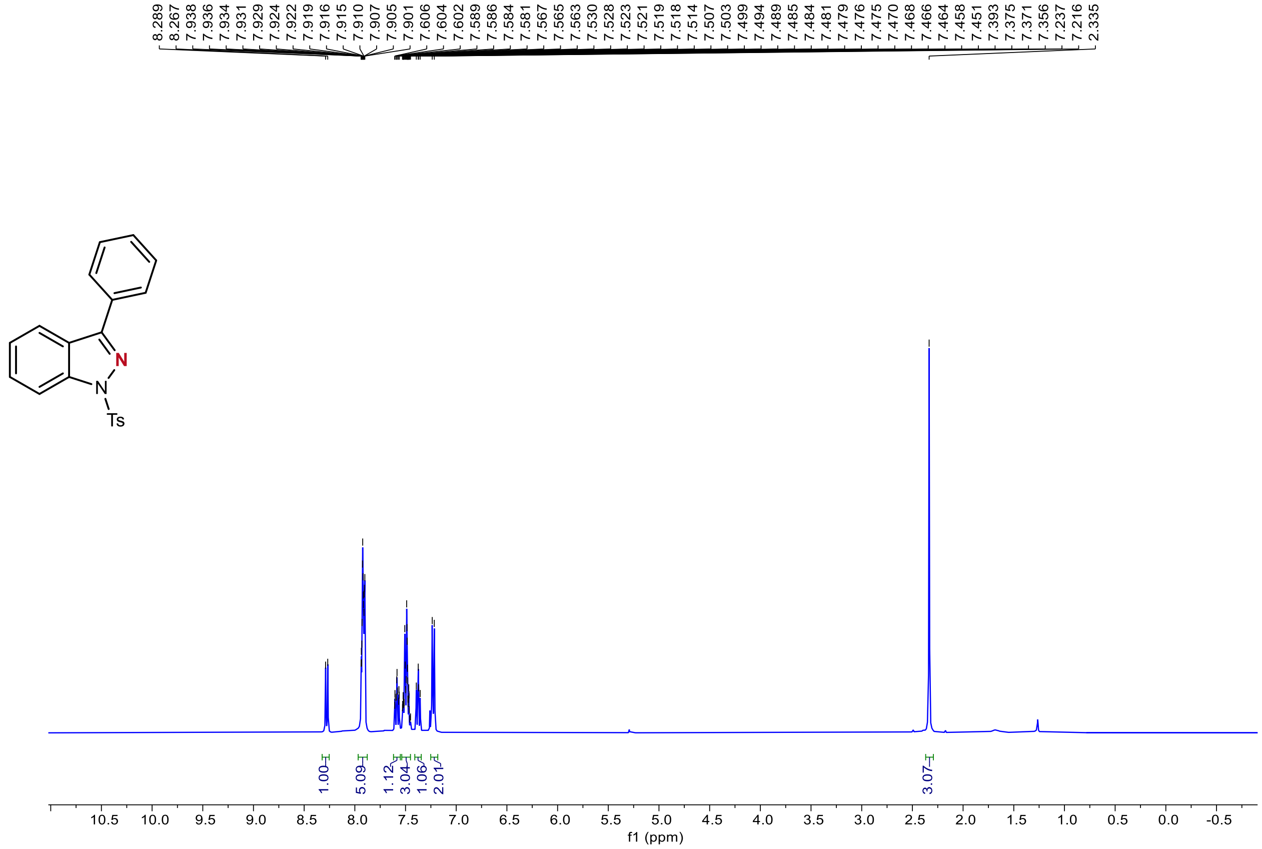

^{13}C NMR (101 MHz, $CDCl_3$)

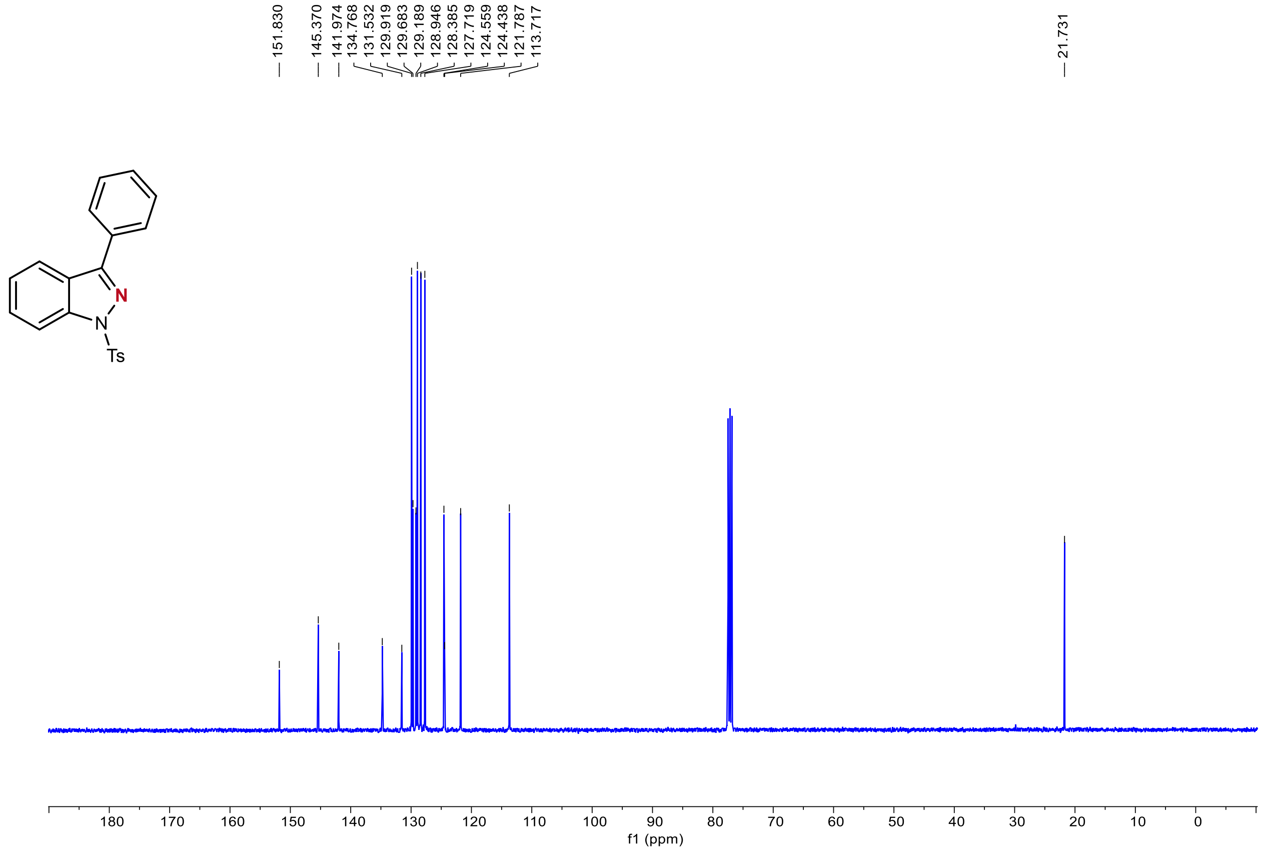

2-Phenyl-1-tosyl-1*H*-benzo[*d*]imidazole Ts-3l

^{1}H NMR (400 MHz, $CDCl_3$)

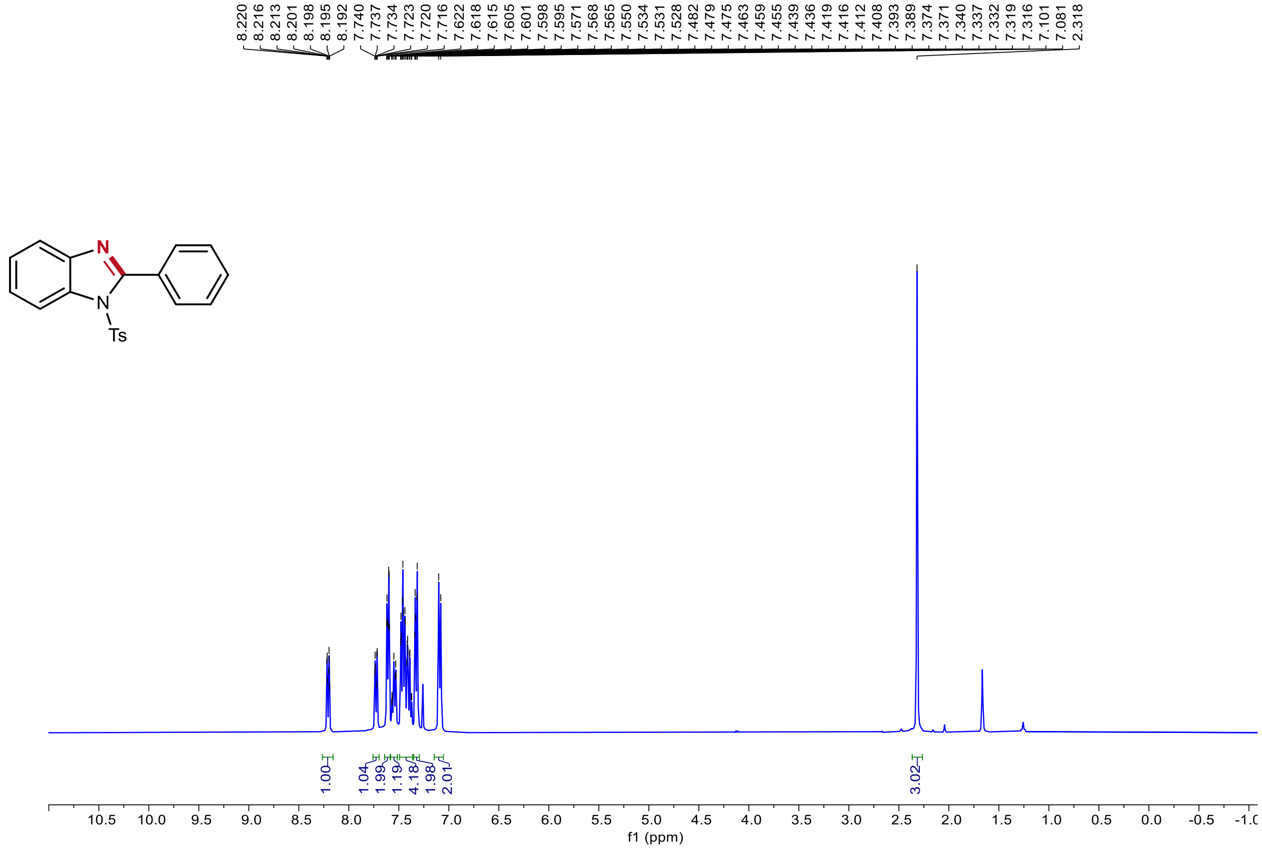

^{13}C NMR (76 MHz, $CDCl_3$)

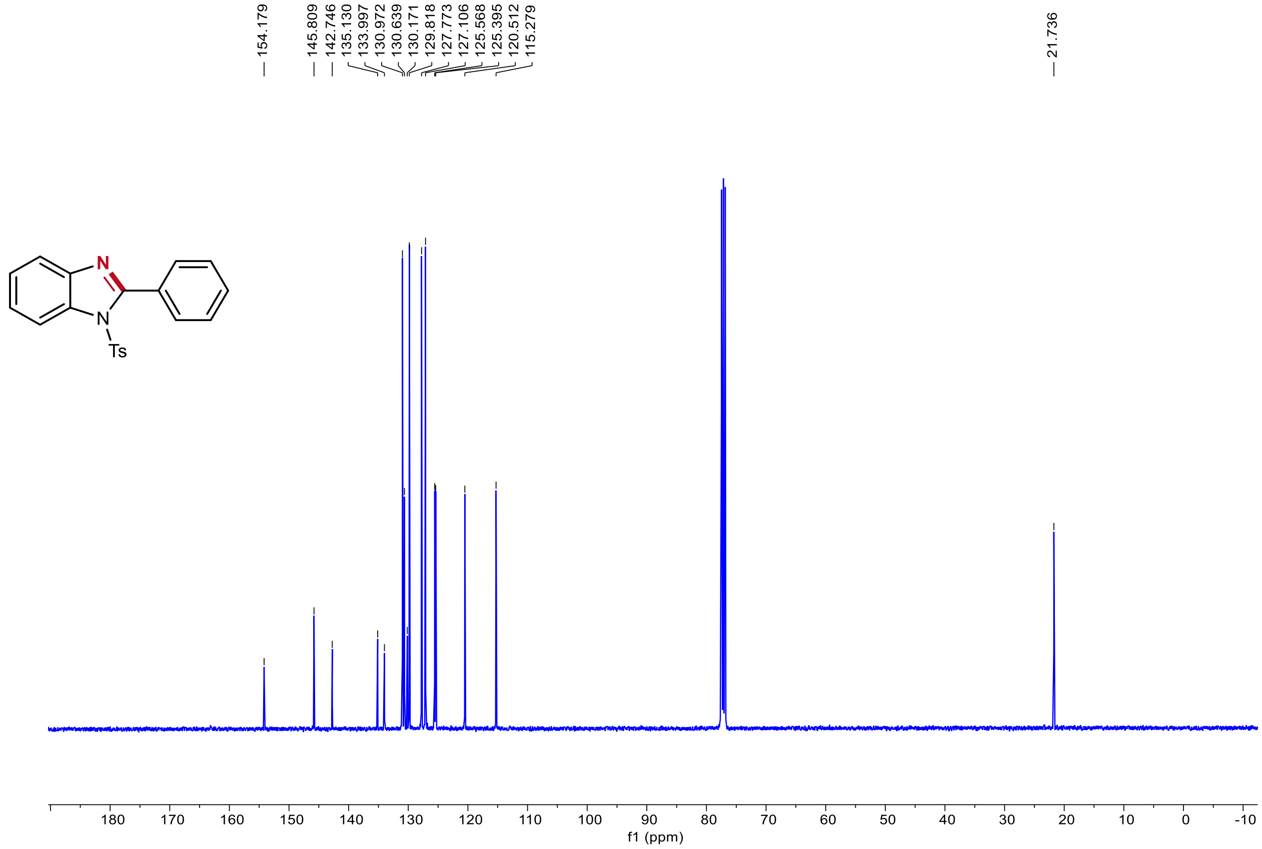

1*H*-indazole 2m

^{1}H NMR (300 MHz, $CDCl_3$)

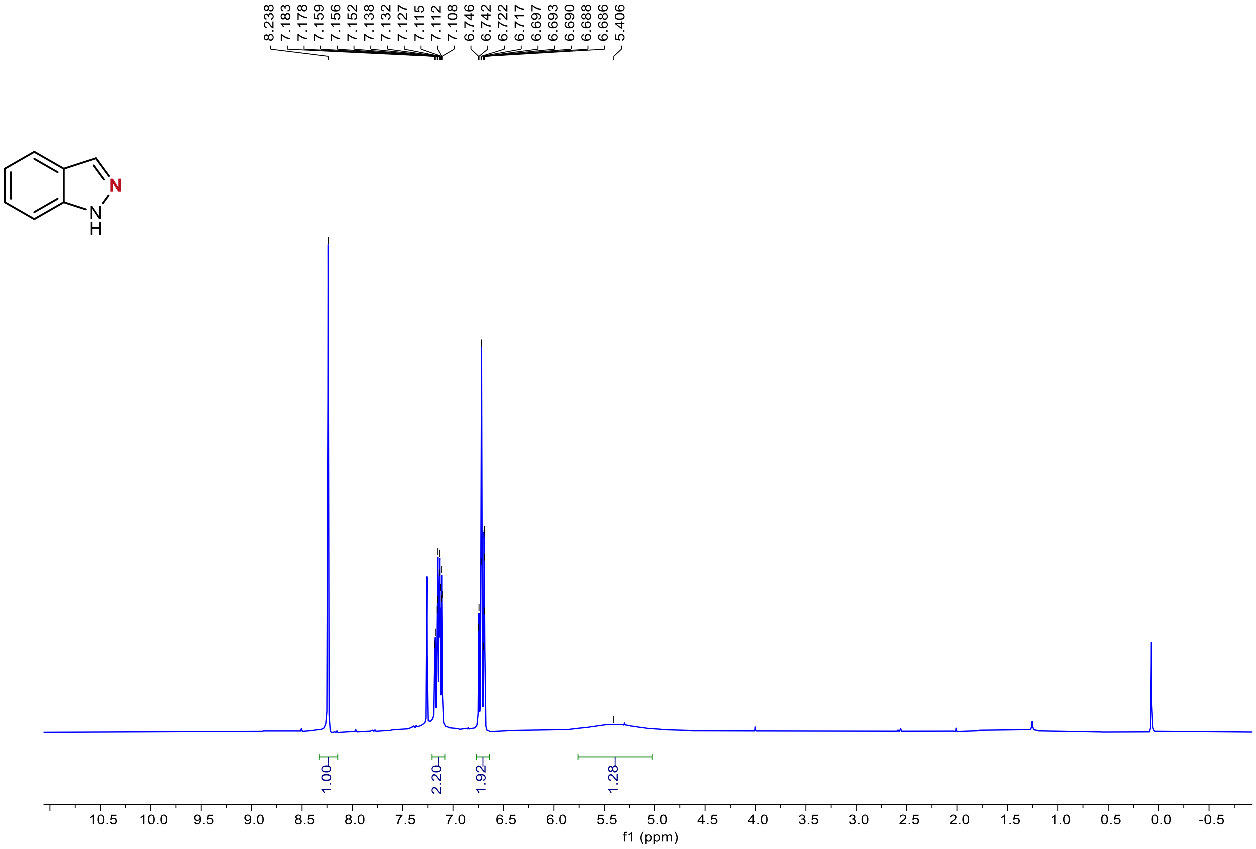

^{13}C NMR (76 MHz, $CDCl_3$)

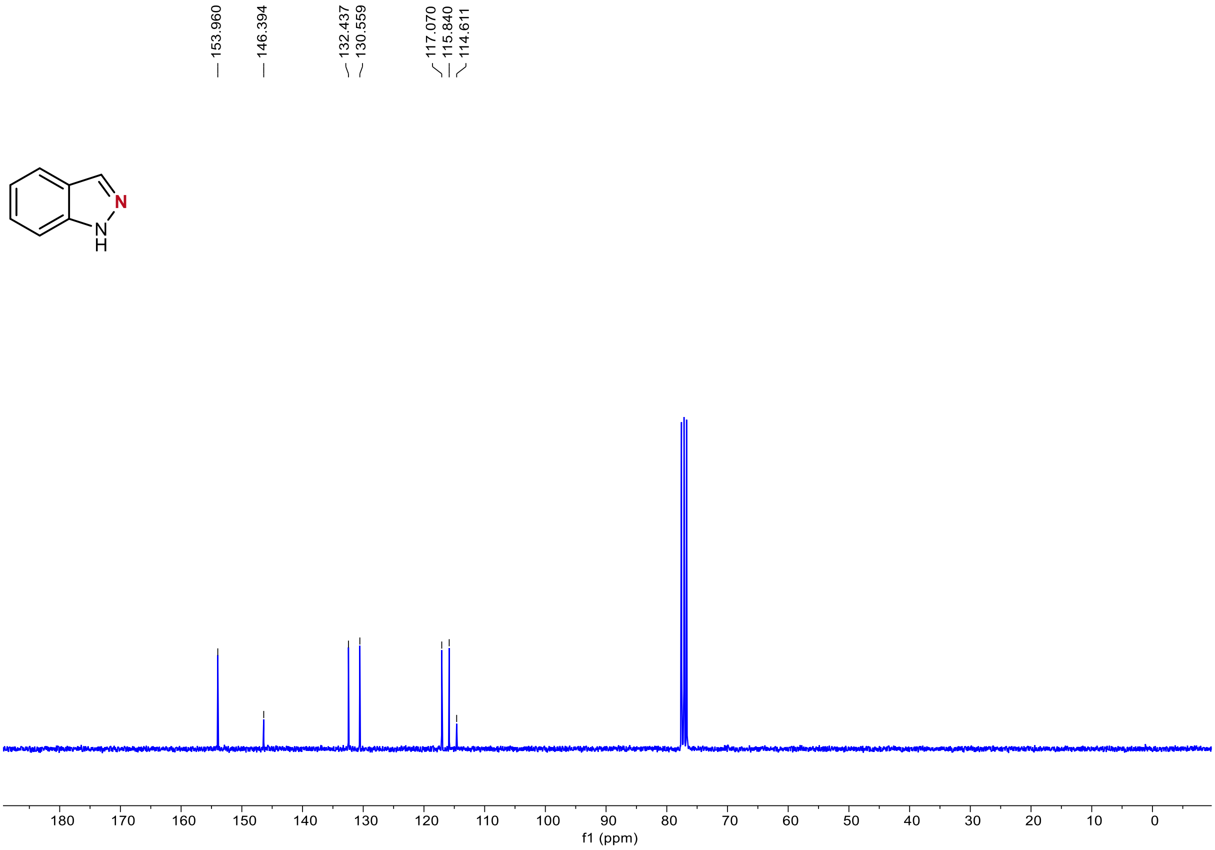

7,8,9,10-Tetrahydrobenzo[4,5]imidazo[1,2-*a*]azocin-11(6*H*)-one 3p’

^{1}H NMR (400 MHz, $CDCl_3$)

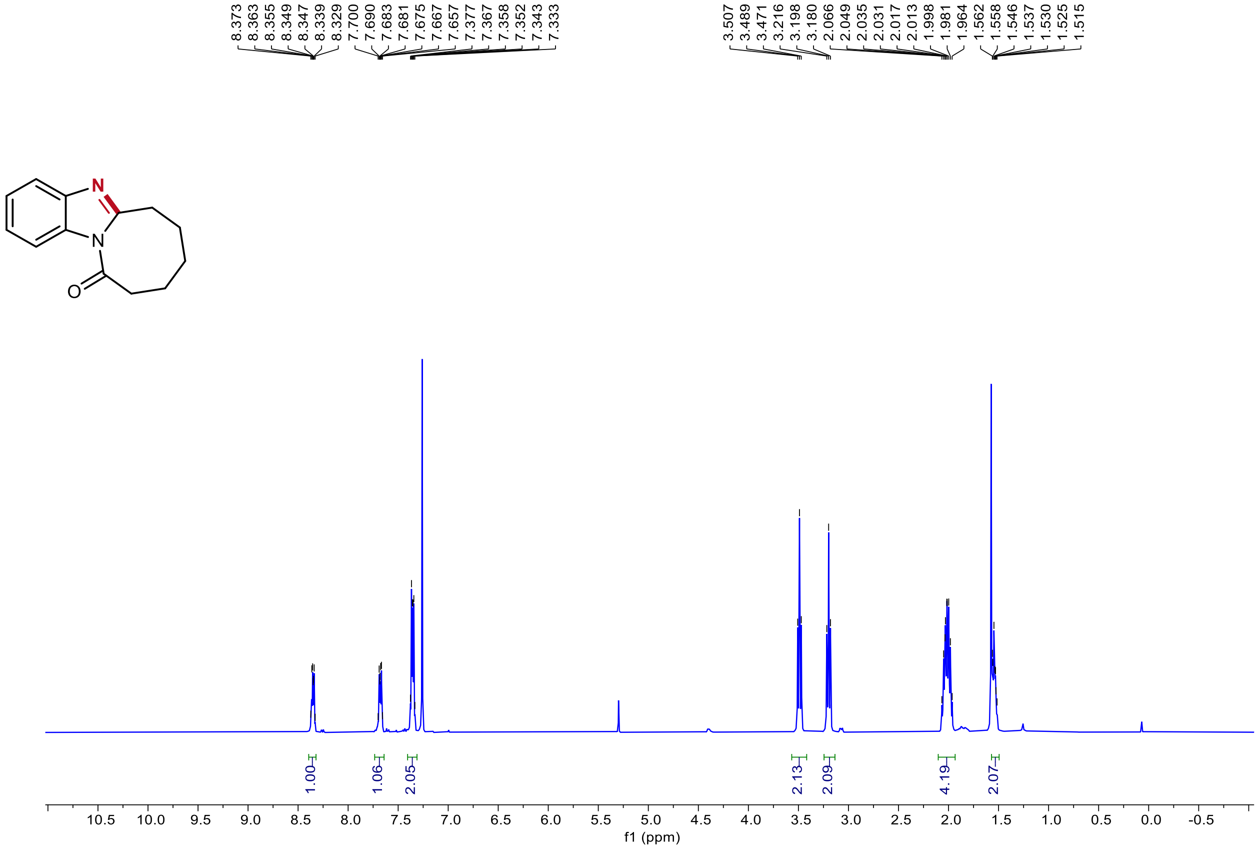

^{13}C NMR (101 MHz, $CDCl_3$)

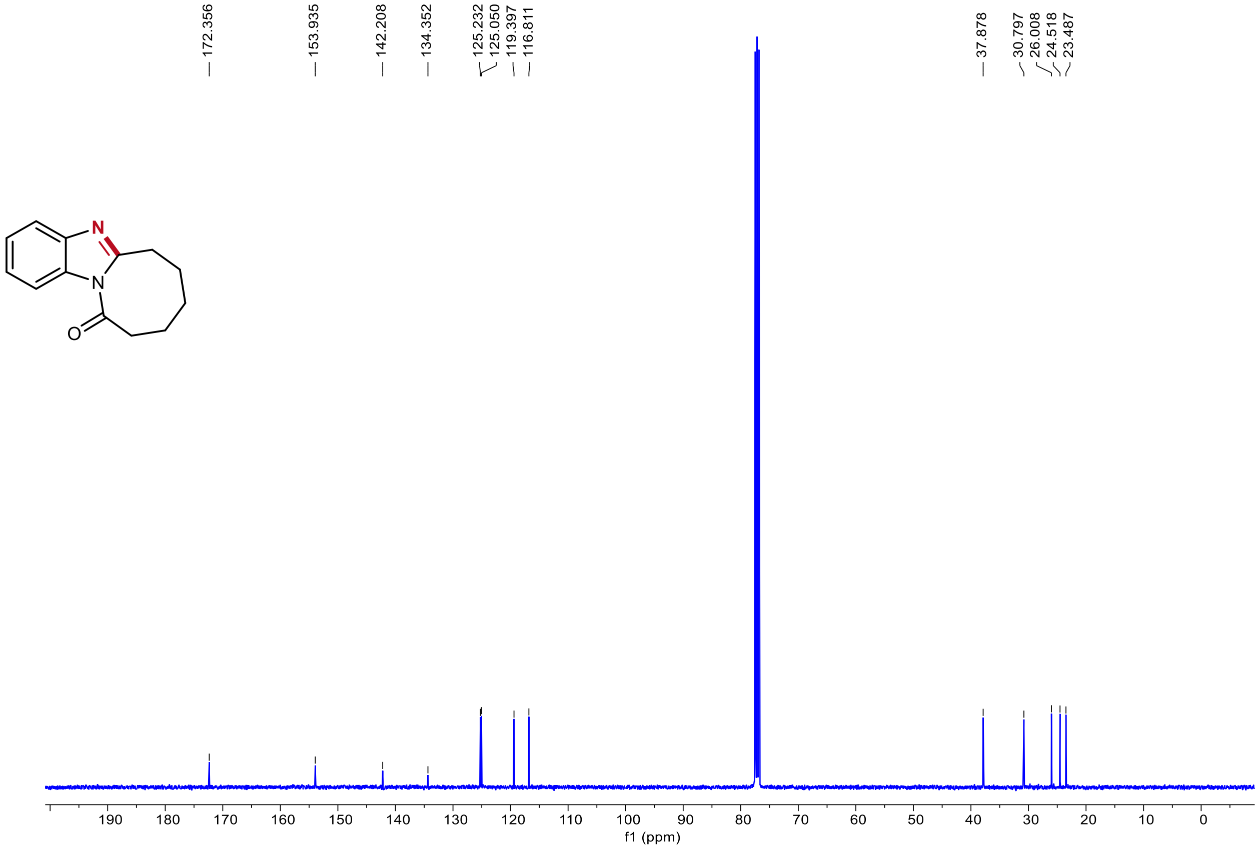

Methyl 5-(1*H*-indazol-3-yl)pentanoate 2q

^{1}H NMR (400 MHz, $CDCl_3$)

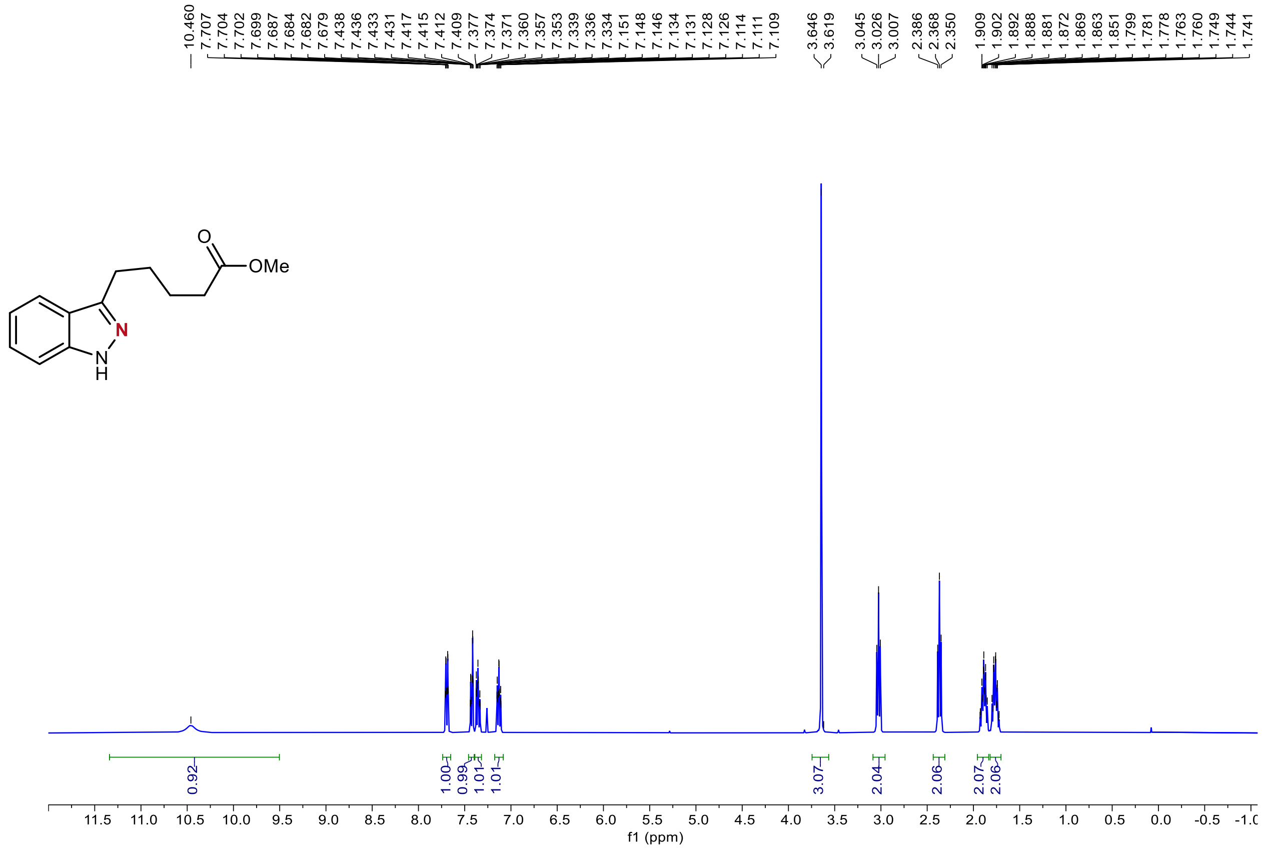

^{13}C NMR (101 MHz, $CDCl_3$)

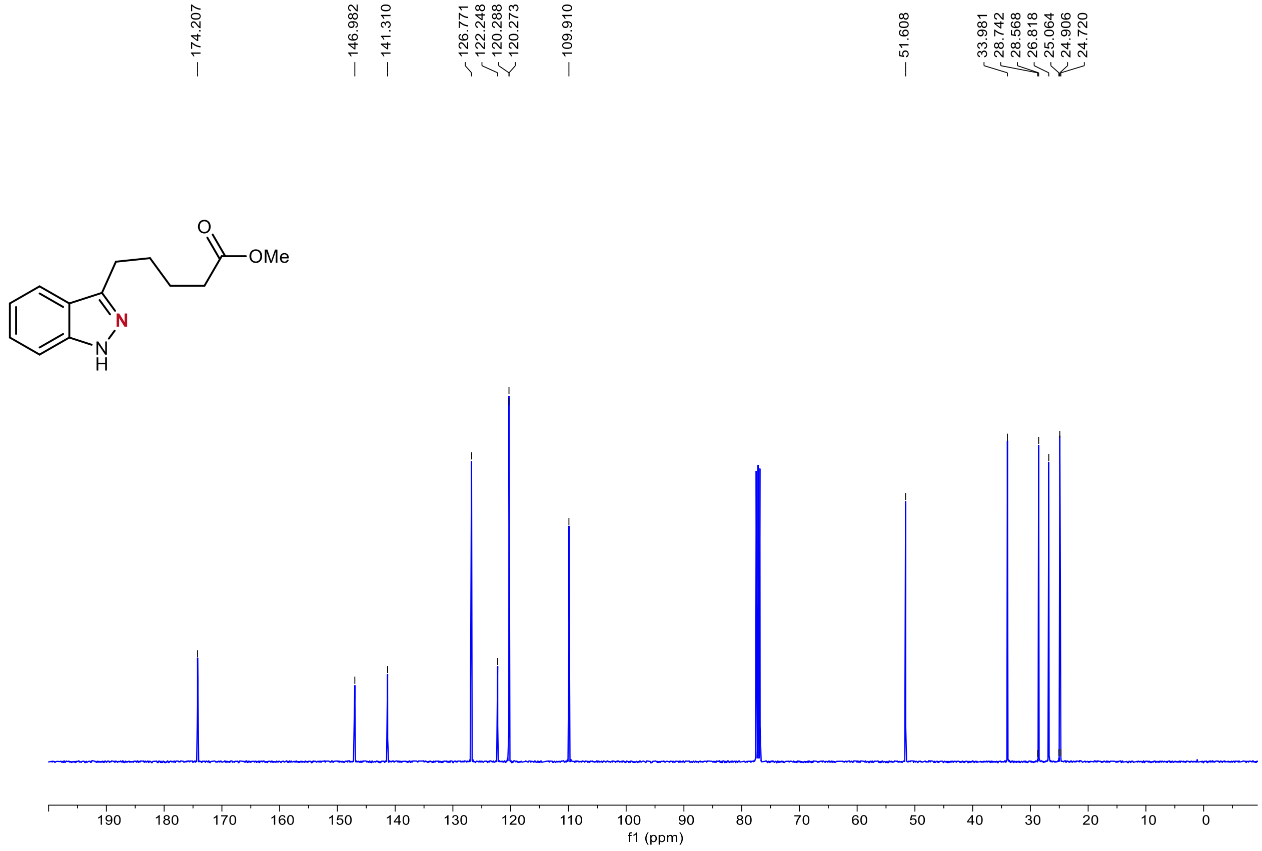

Methyl 5-(1-tosyl-1H-benzo[d]imidazol-2-yl)pentanoate Ts-3q

^{1}H NMR (400 MHz, CDCl$_3$)

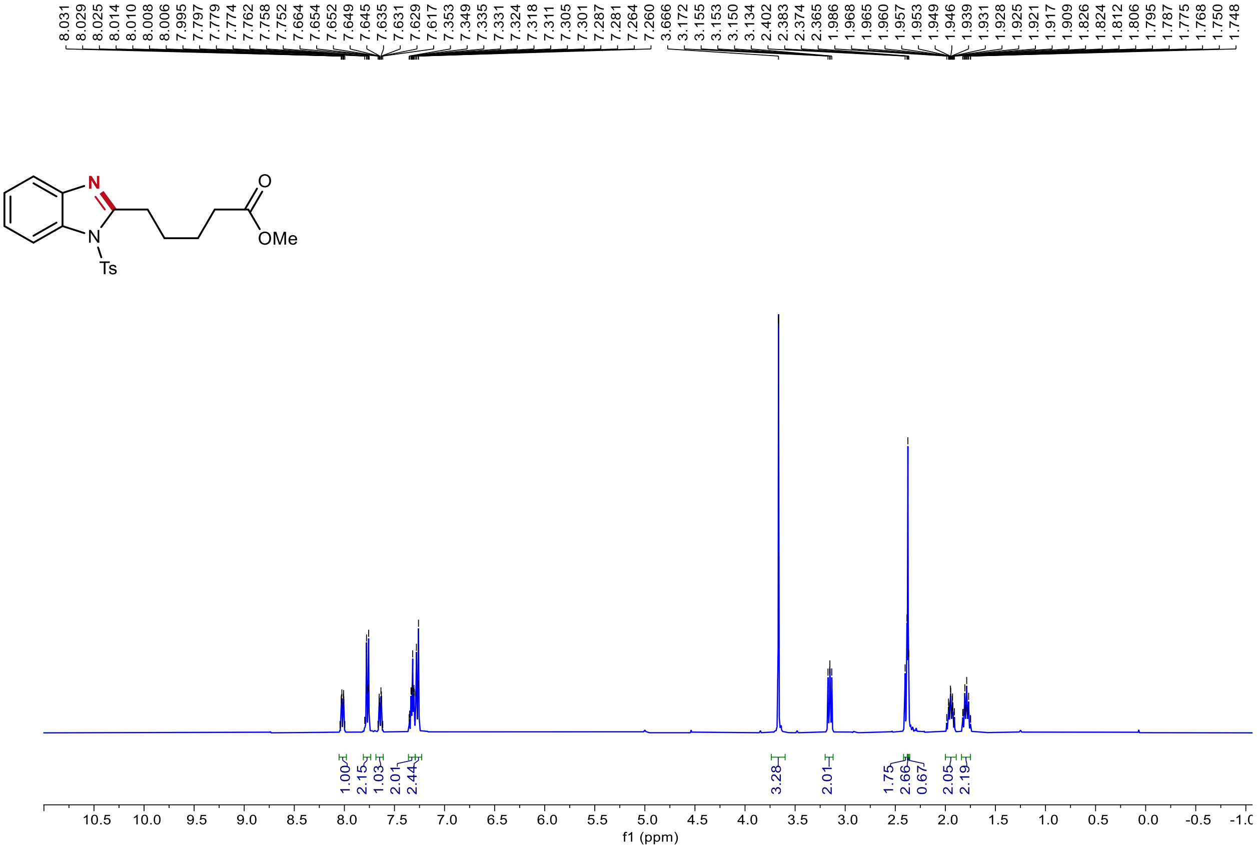

^{13}C NMR (101 MHz, CDCl$_3$)

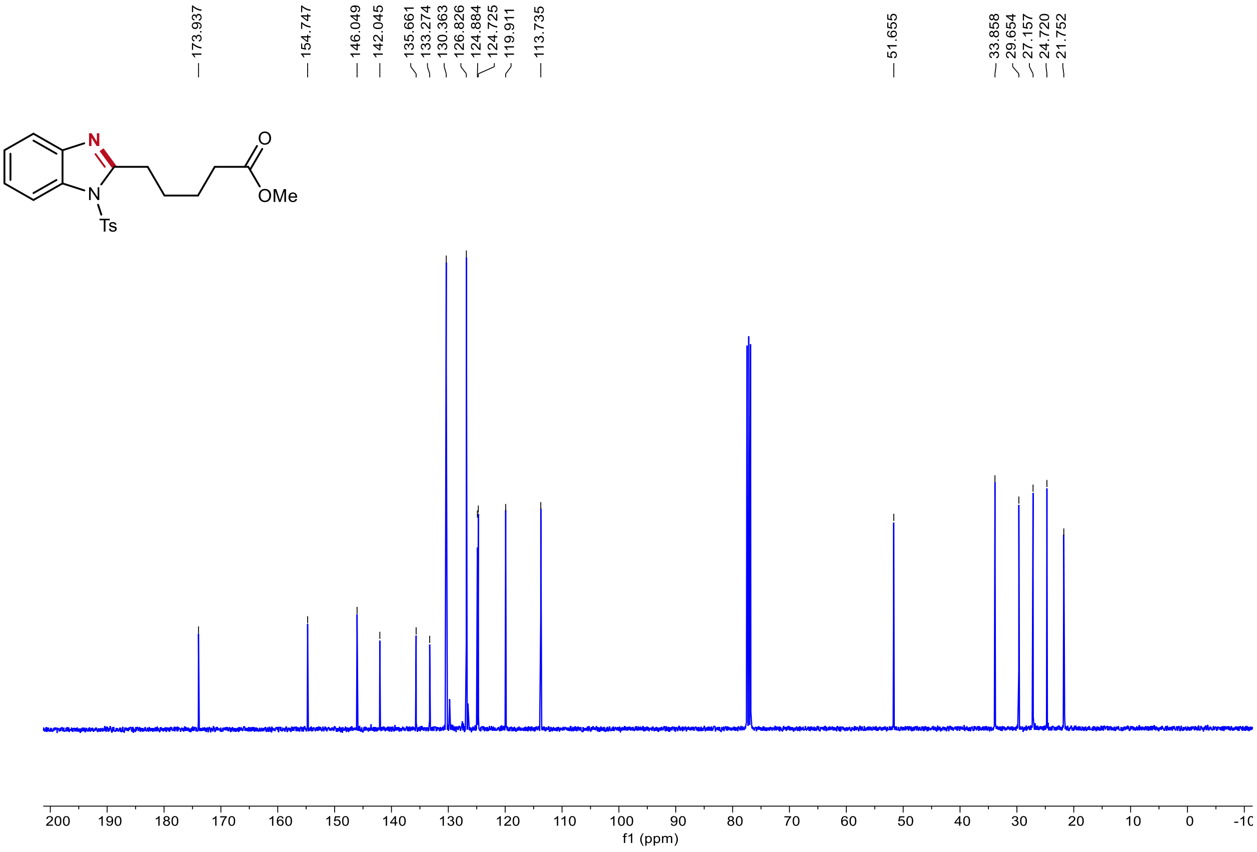

Methyl 5-(1-tosyl-1*H*-indazol-3-yl)pentanoate Ts-2q

^{1}H NMR (400 MHz, $CDCl_3$)

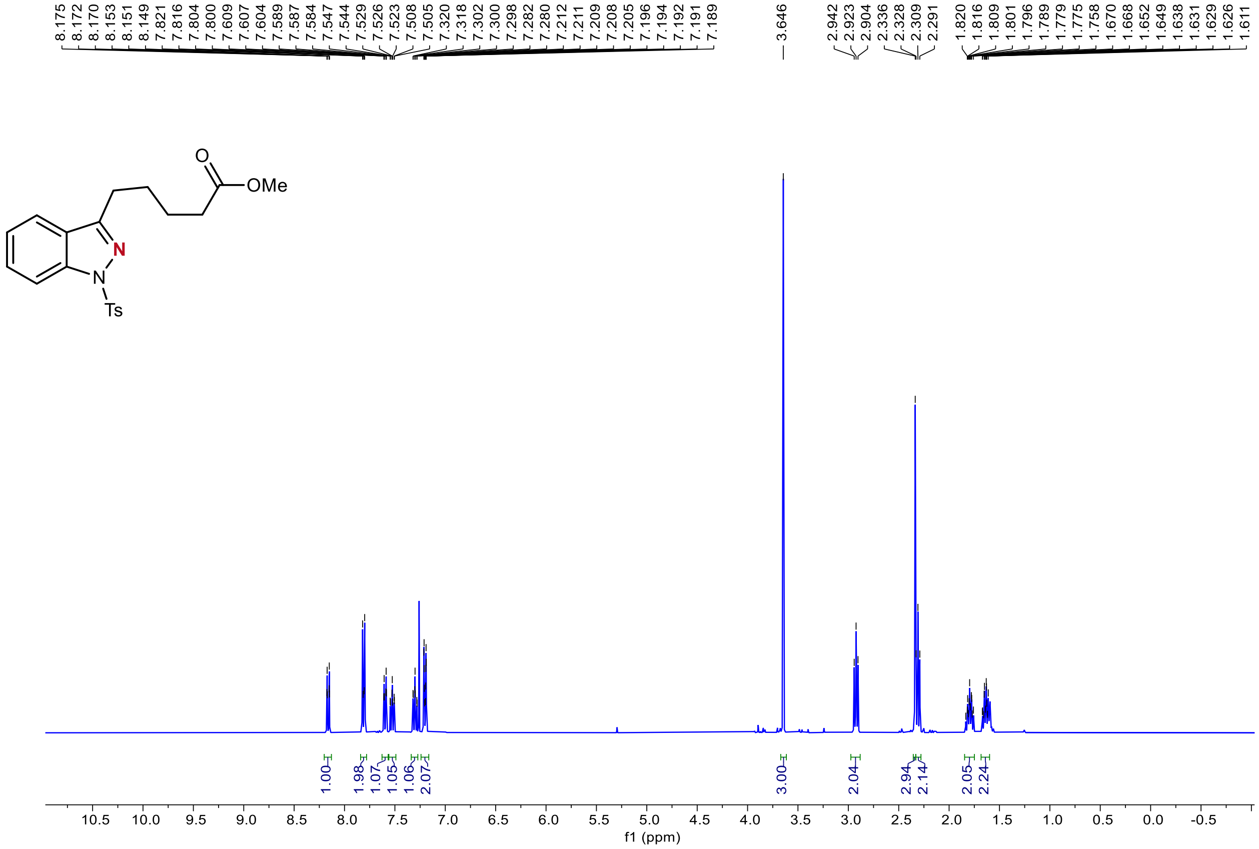

^{13}C NMR (101 MHz, $CDCl_3$)

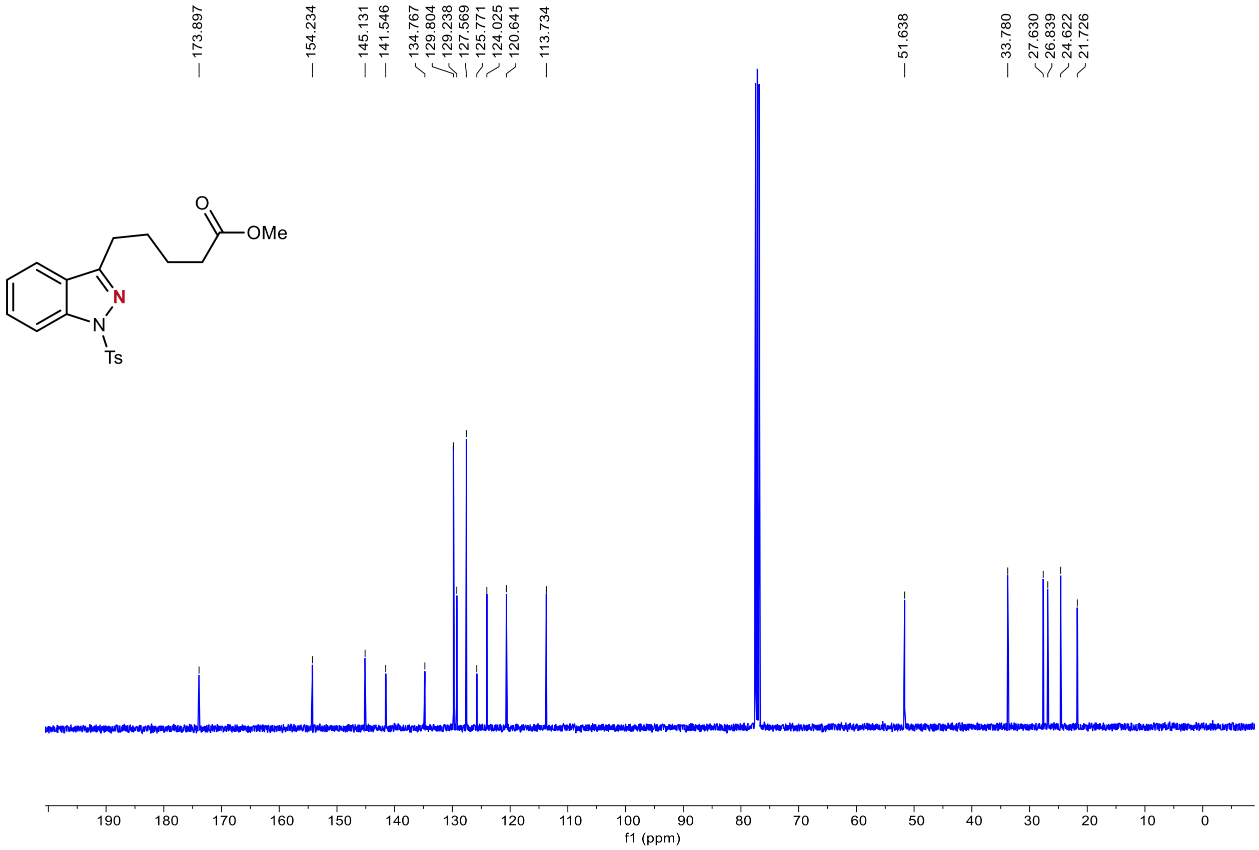

6,7,8,9-Tetrahydro-10*H*-benzo[4,5]imidazo[1,2-*a*]azepin-10-one 3q'

^{1}H NMR (300 MHz, $CDCl_3$)

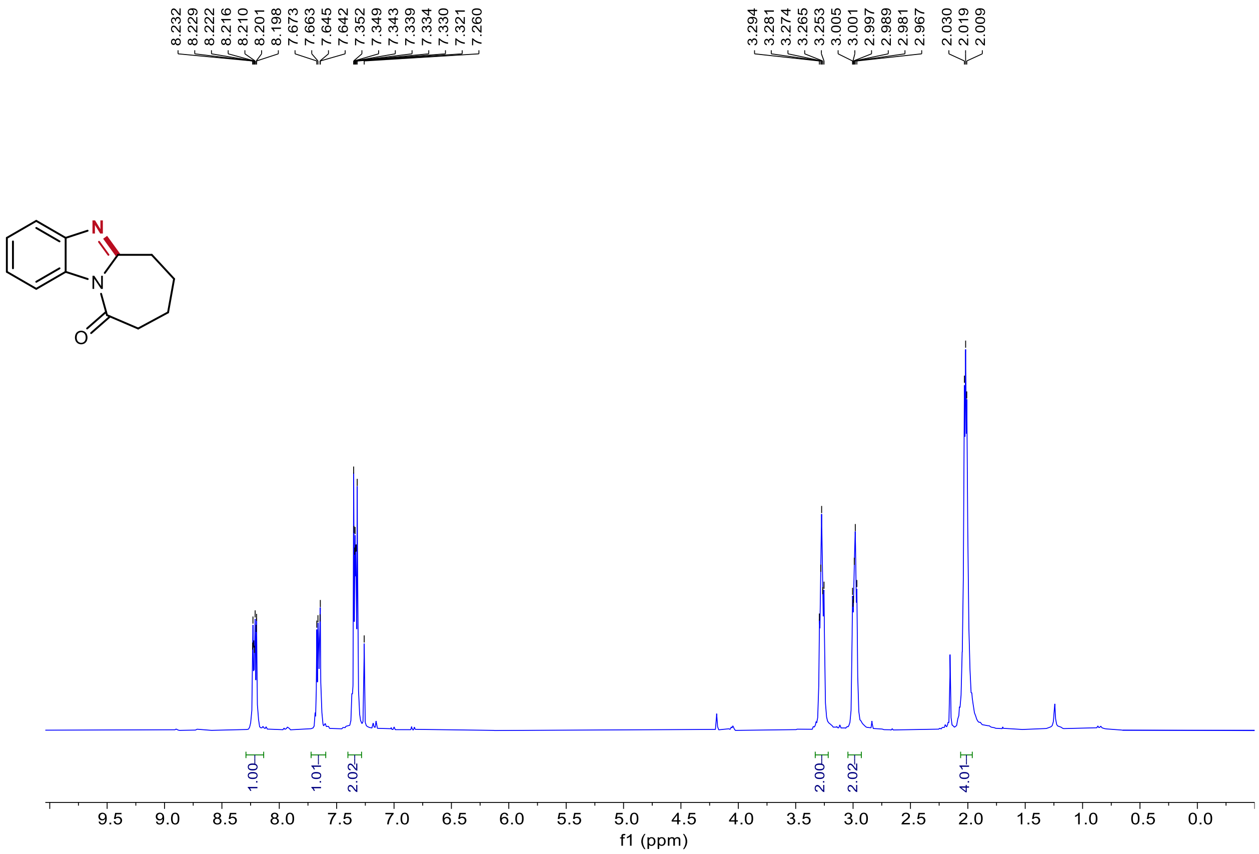

^{13}C NMR (76 MHz, $CDCl_3$)

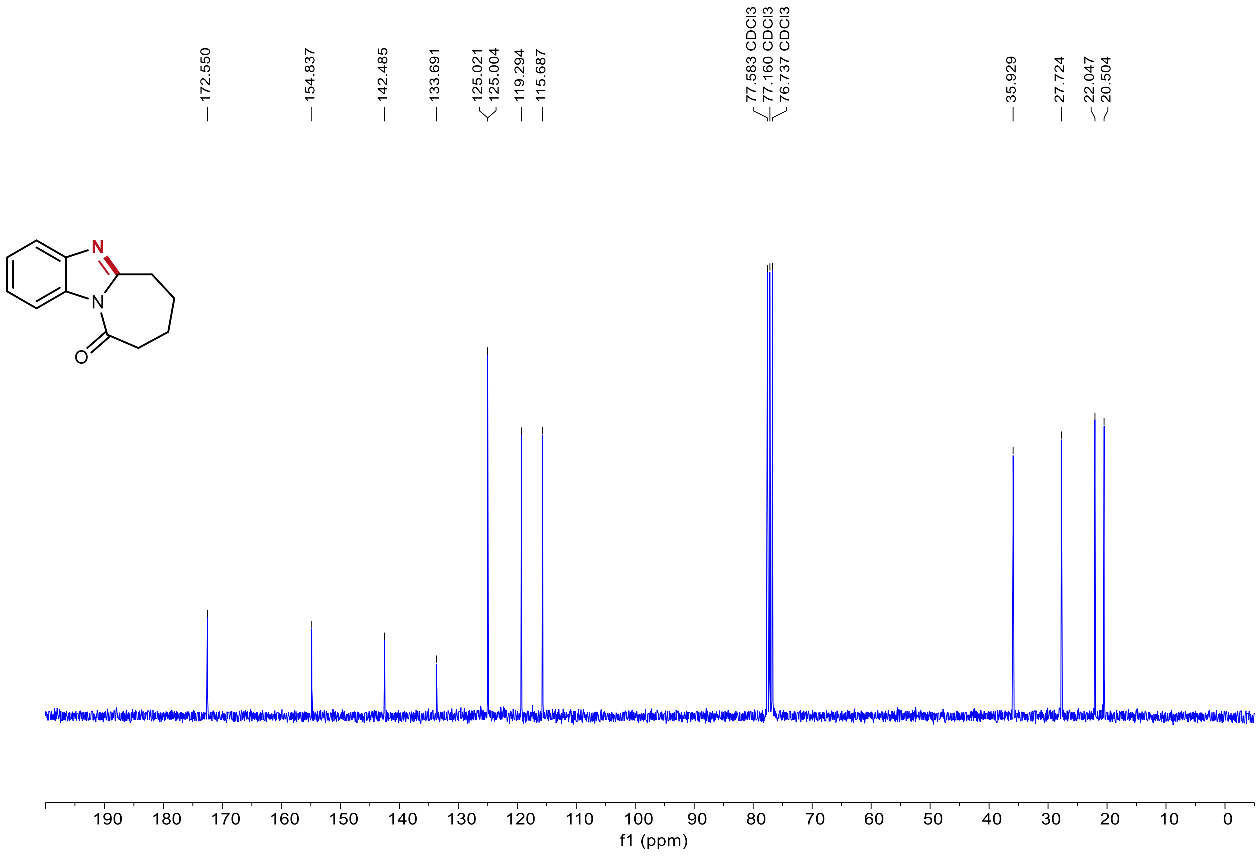

3-Methylbenzo[*d*]isoxazole 5a

^{1}H NMR (300 MHz, $CDCl_3$)

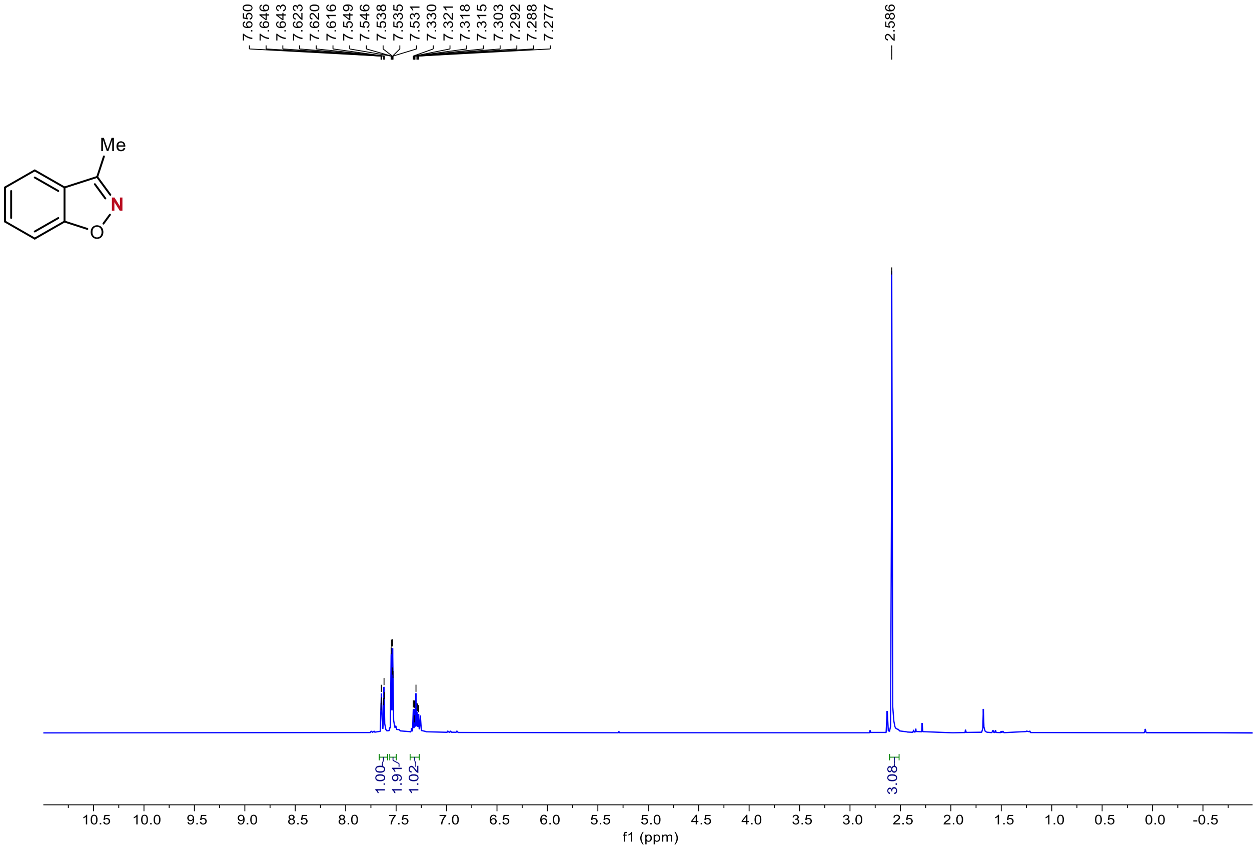

^{13}C NMR (101 MHz, $CDCl_3$)

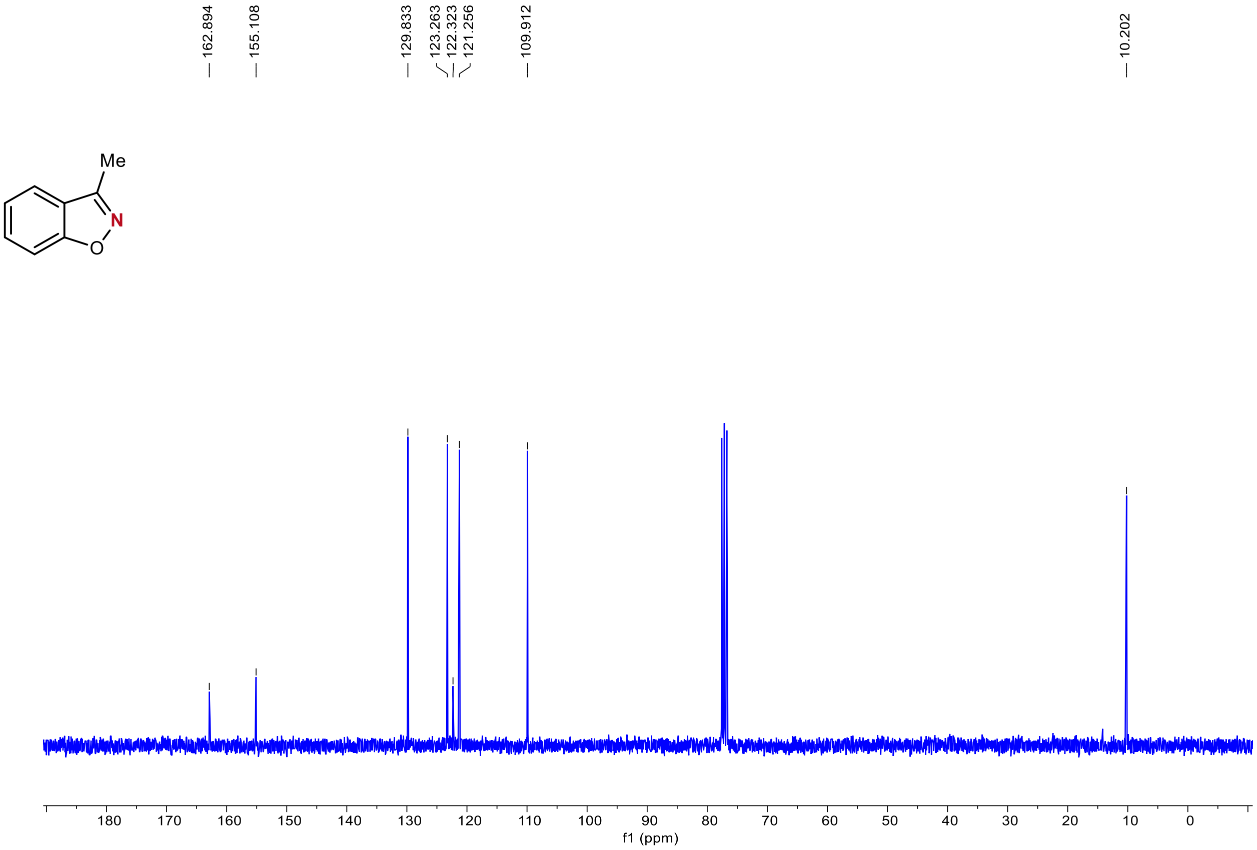

2-Methylbenzo[*d*]oxazole 6a

^{1}H NMR (300 MHz, $CDCl_3$)

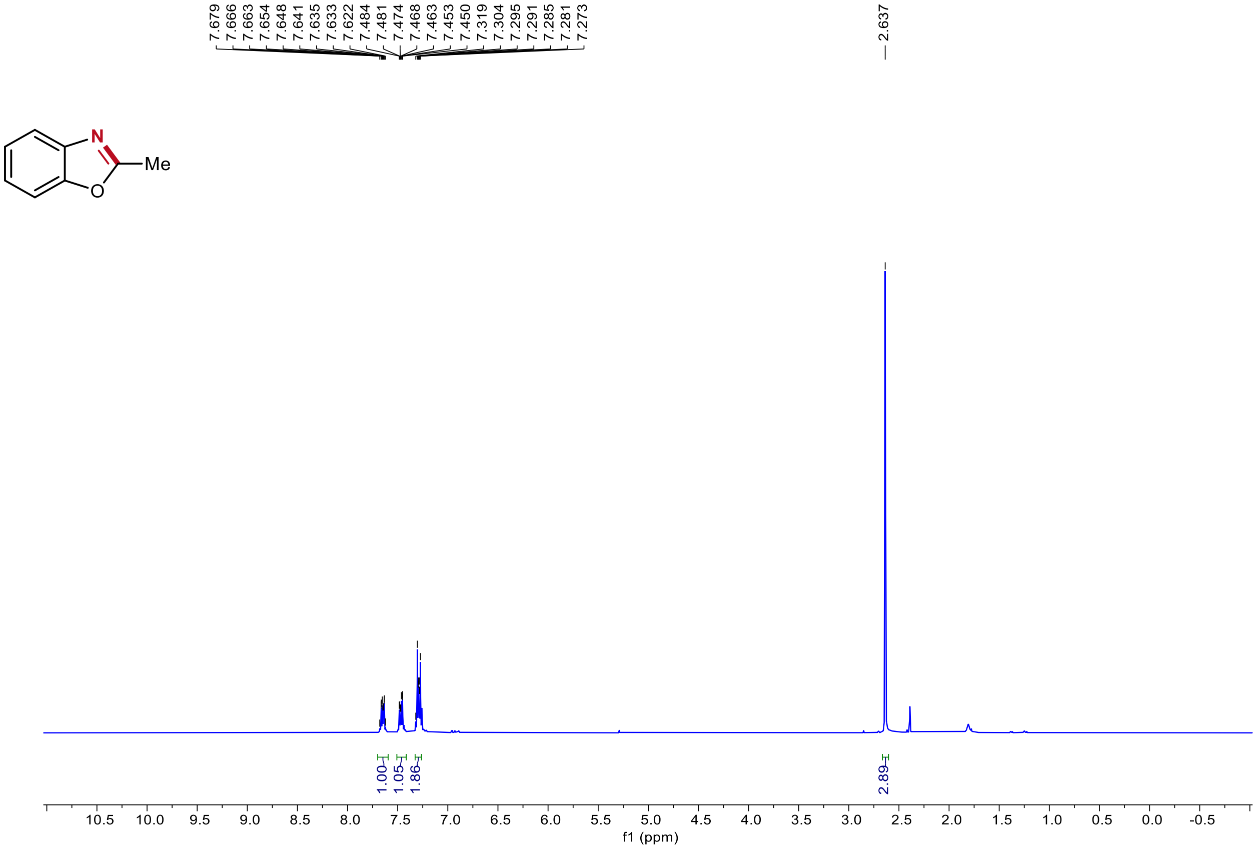

^{13}C NMR (101 MHz, $CDCl_3$)

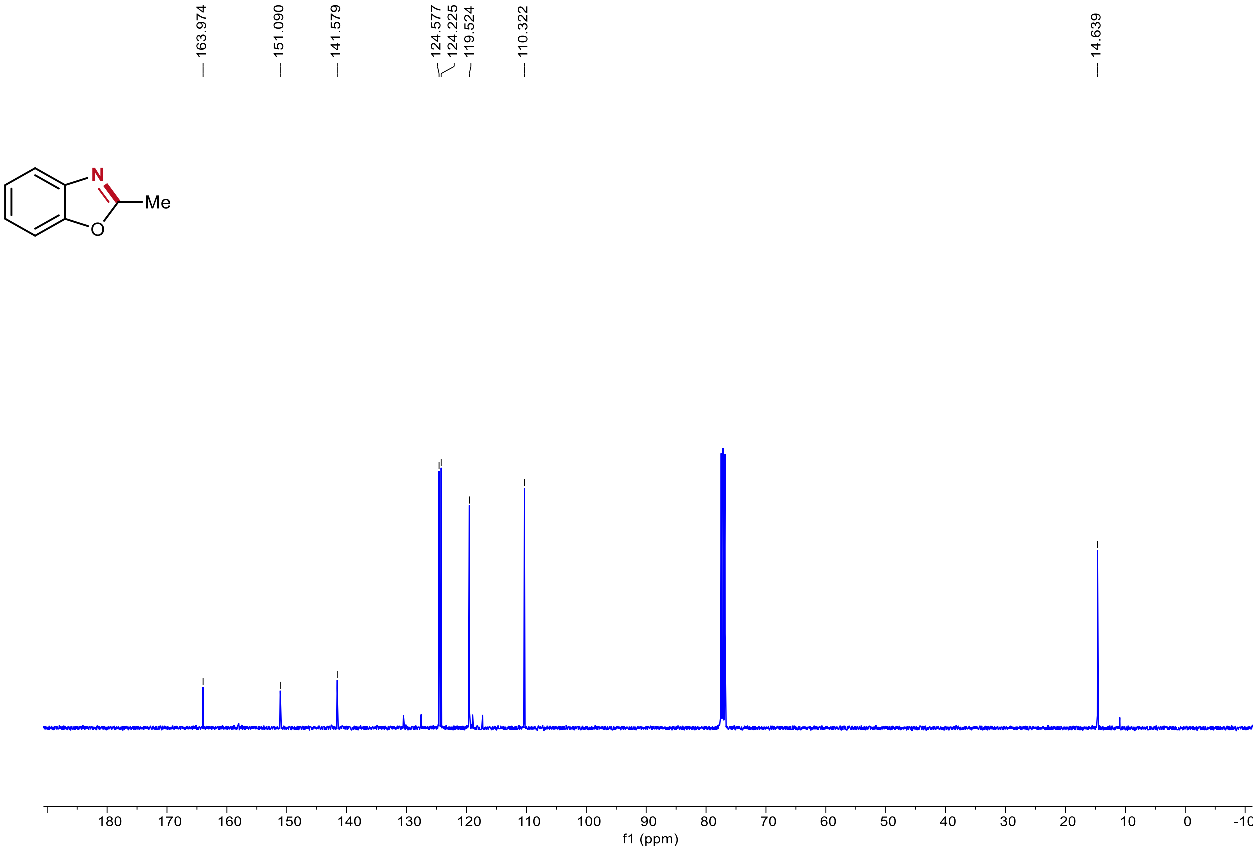

3-Methylbenzo[*d*]isoxazol-5-yl acetate 5b

^{1}H NMR (400 MHz, $CDCl_3$)

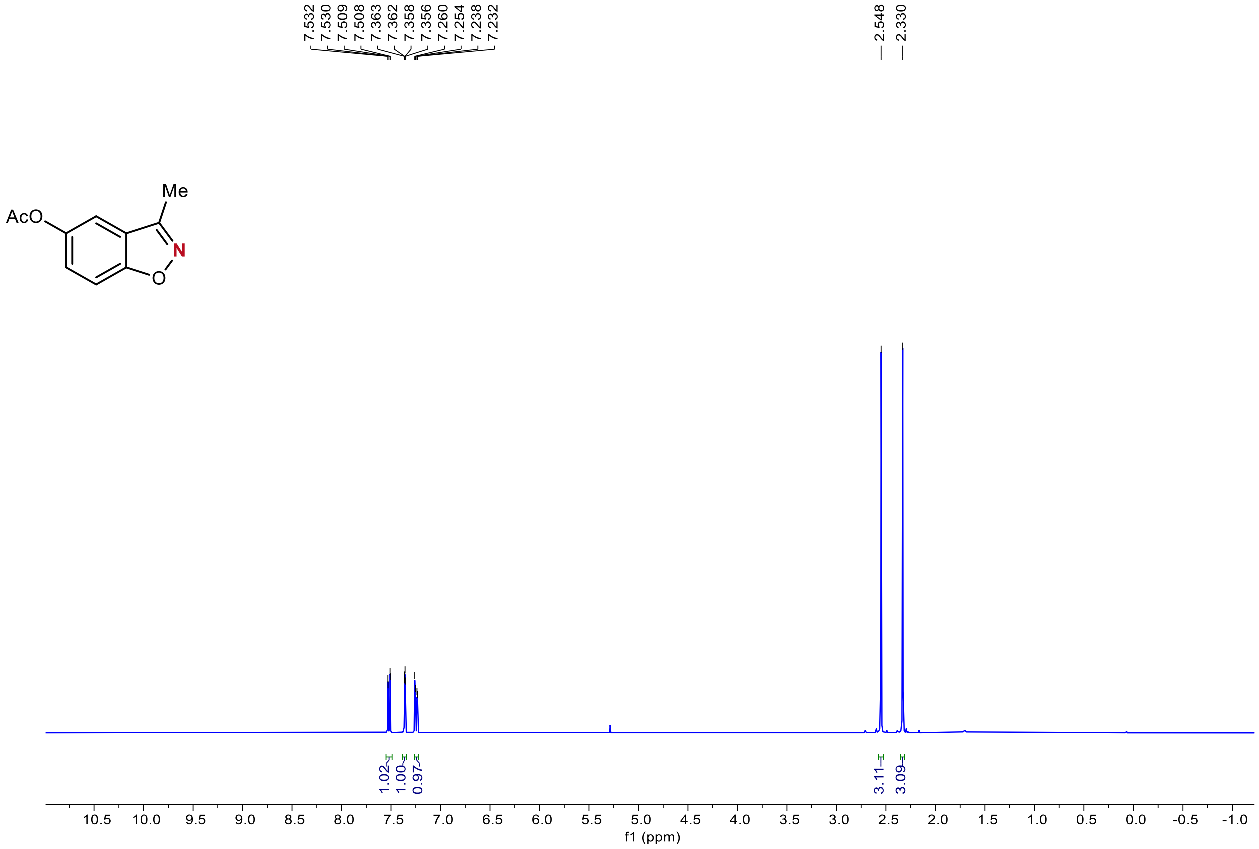

^{13}C NMR (101 MHz, $CDCl_3$)

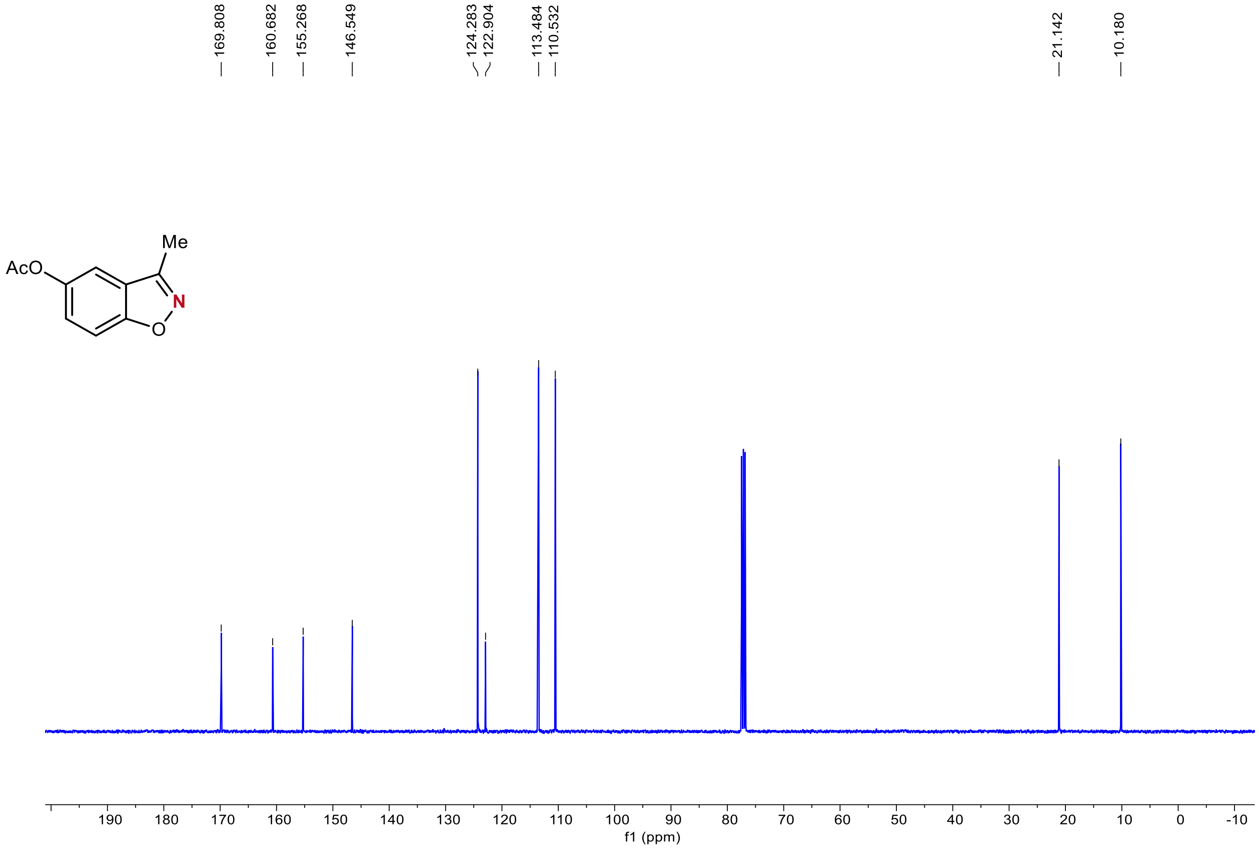

2-Methylbenzo[*d*]oxazol-5-yl acetate 6b

^{1}H NMR (300 MHz, $CDCl_3$)

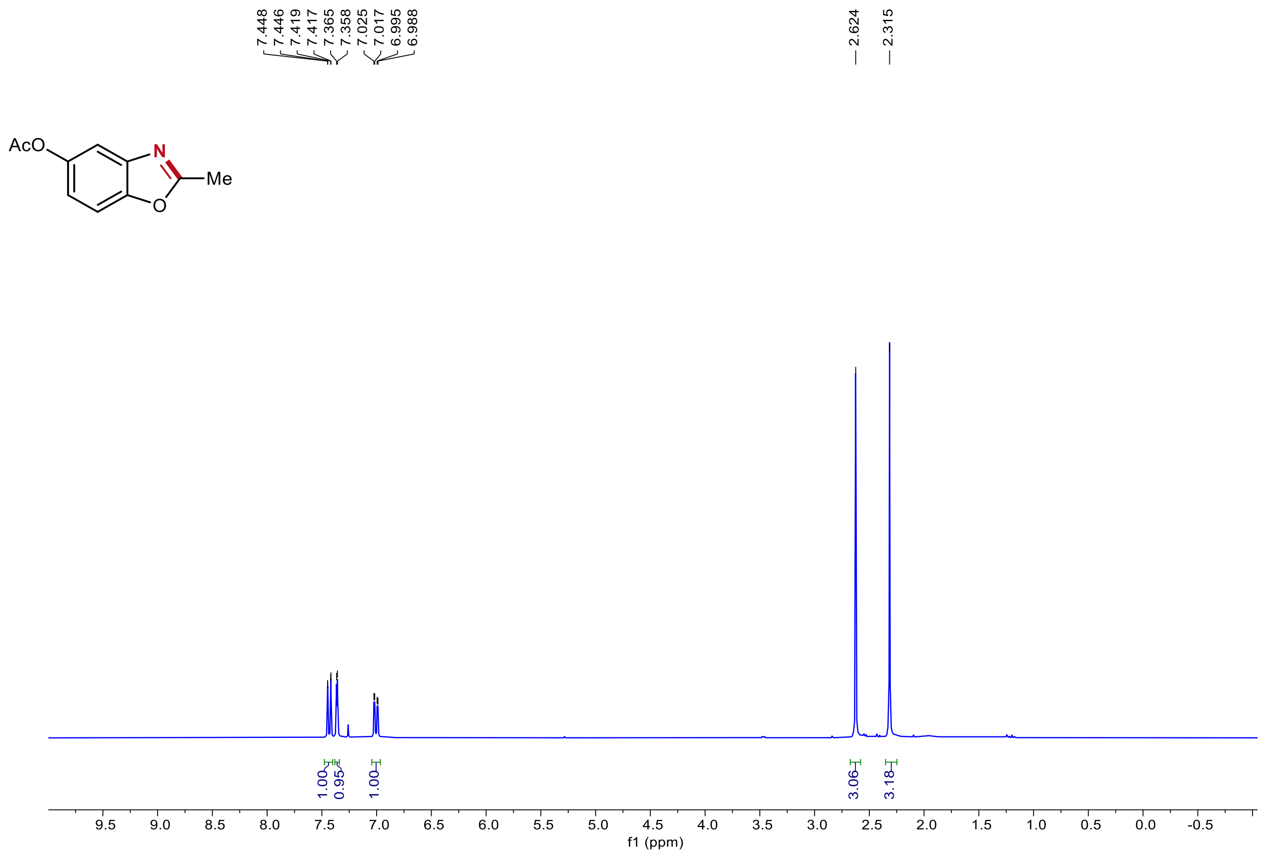

^{13}C NMR (76 MHz, $CDCl_3$)

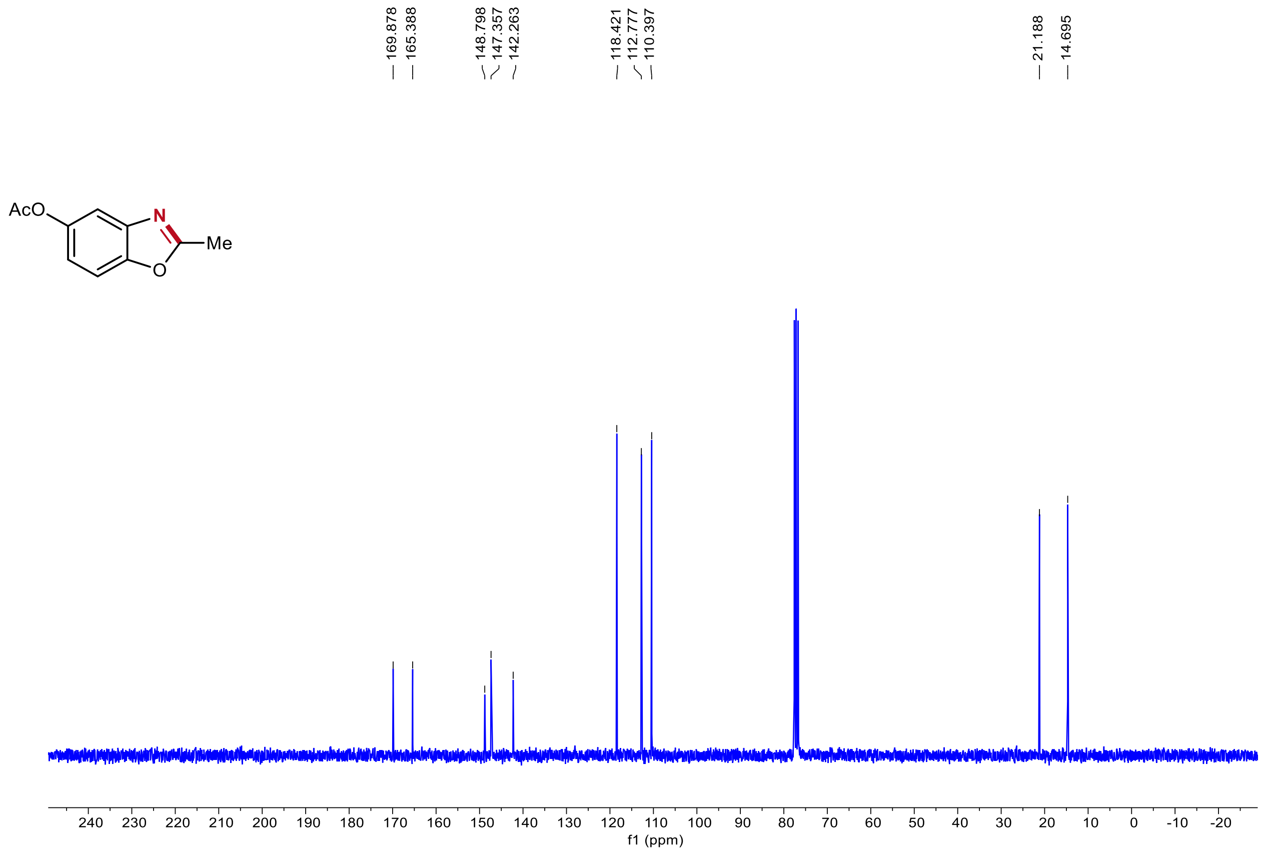

5-(But-2-yn-1-yloxy)-3-methylbenzo[*d*]isoxazole 5c

^{1}H NMR (400 MHz, $CDCl_3$)

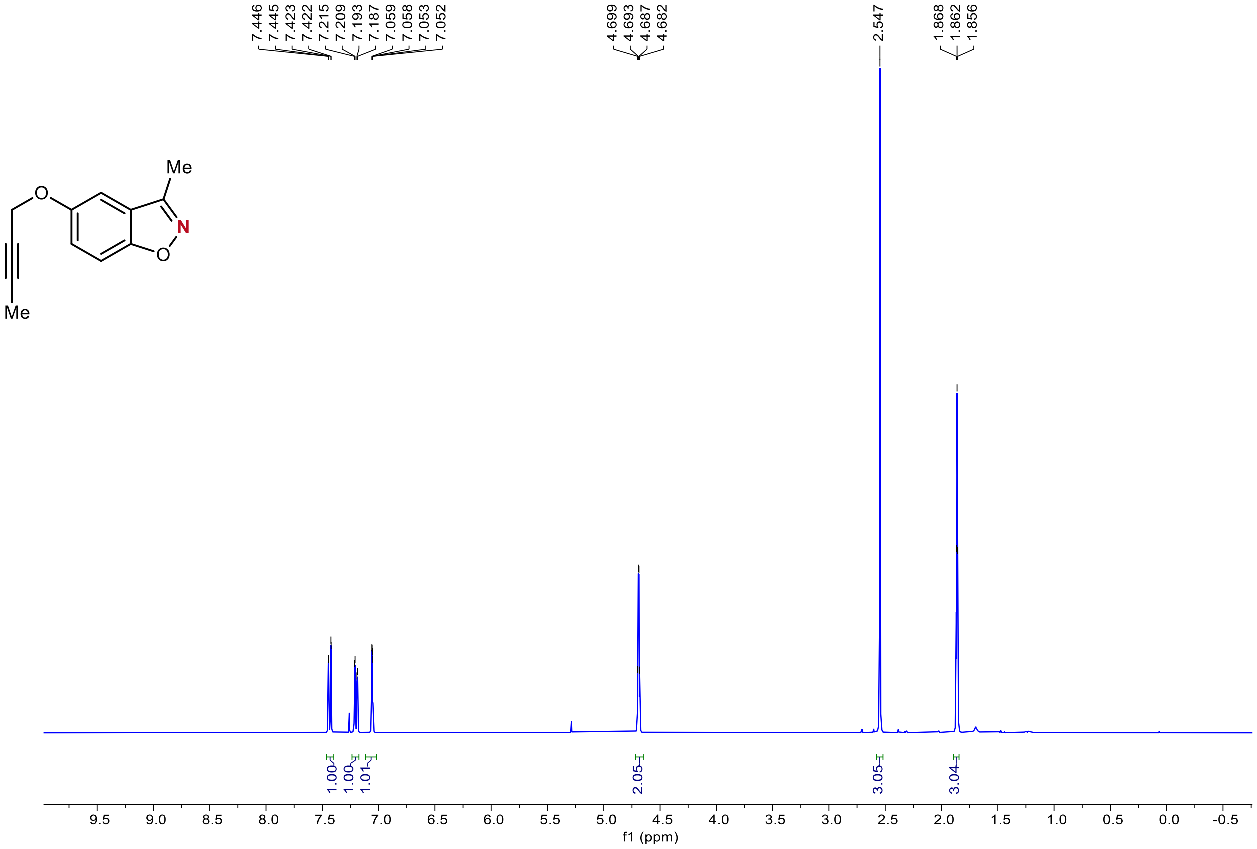

^{13}C NMR (101 MHz, $CDCl_3$)

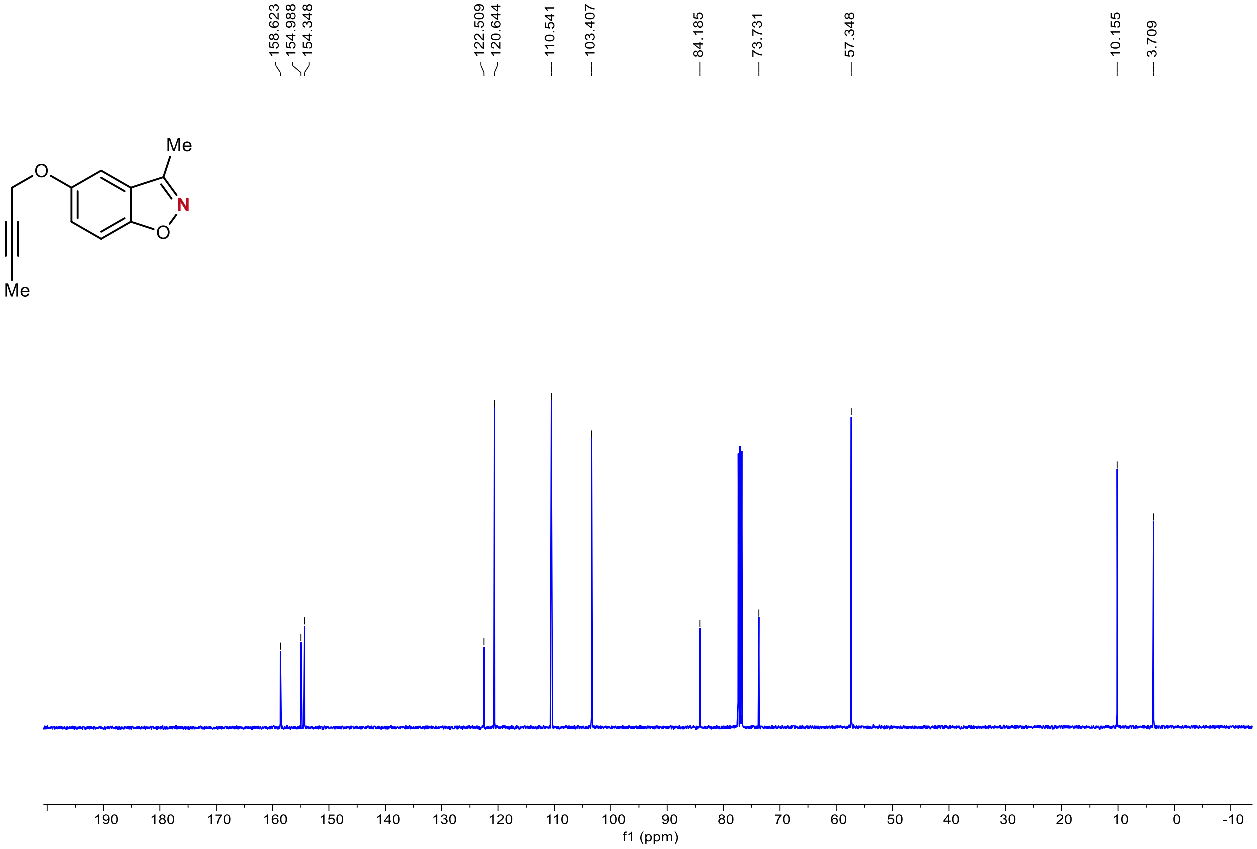

5-(But-2-yn-1-yloxy)-2-methylbenzo[*d*]oxazole 6c

^{1}H NMR (400 MHz, $CDCl_3$)

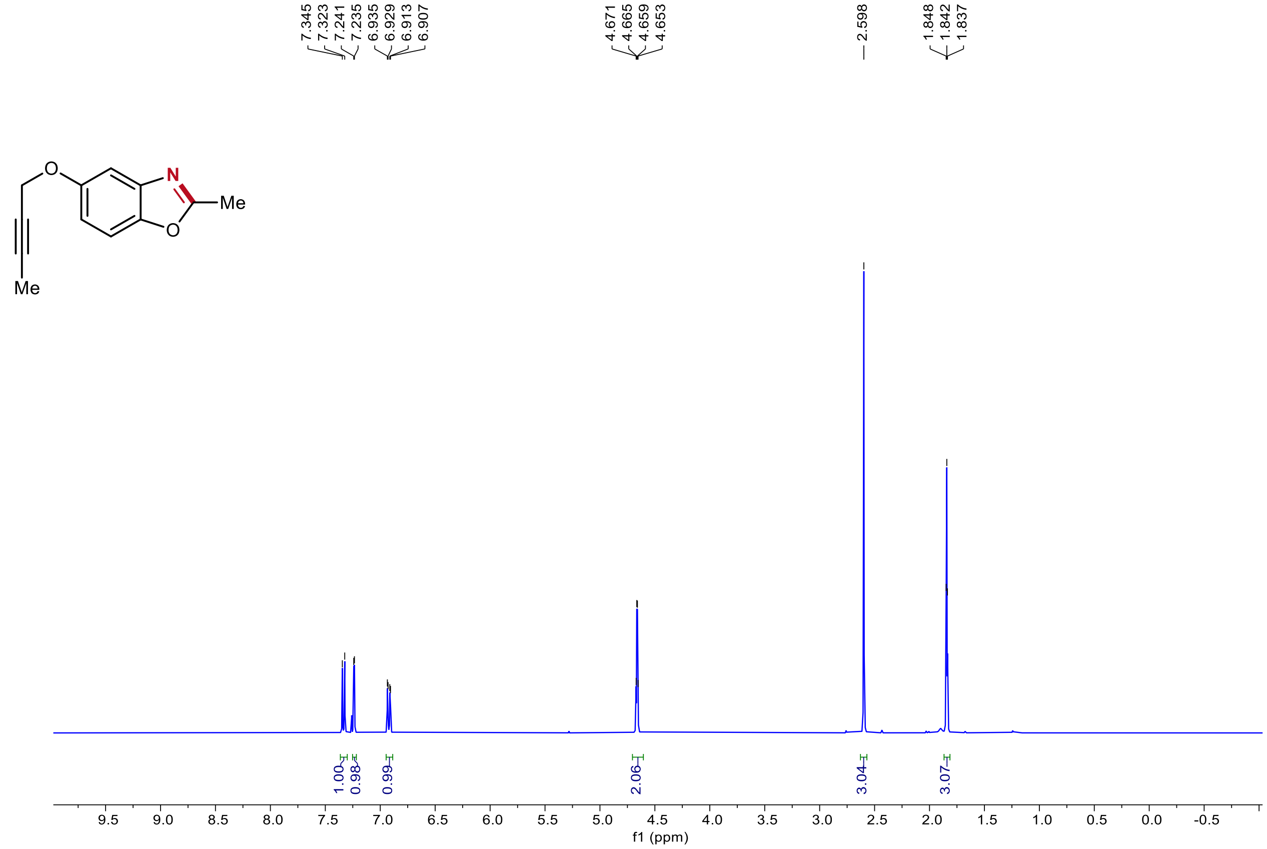

^{13}C NMR (101 MHz, $CDCl_3$)

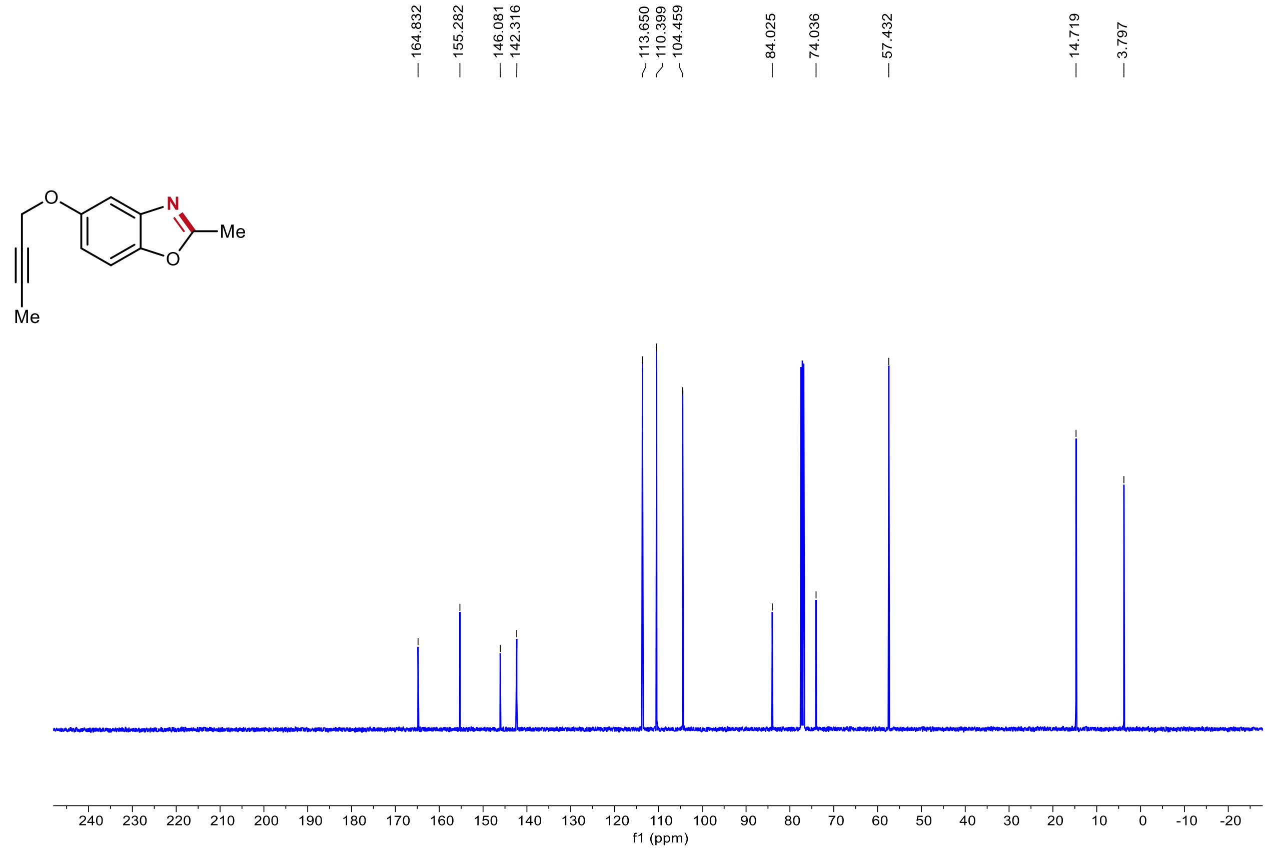

3-Methyl-5-((3-methylbut-2-en-1-yl)oxy)benzo[*d*]isoxazole 5d

^{1}H NMR (400 MHz, $CDCl_3$)

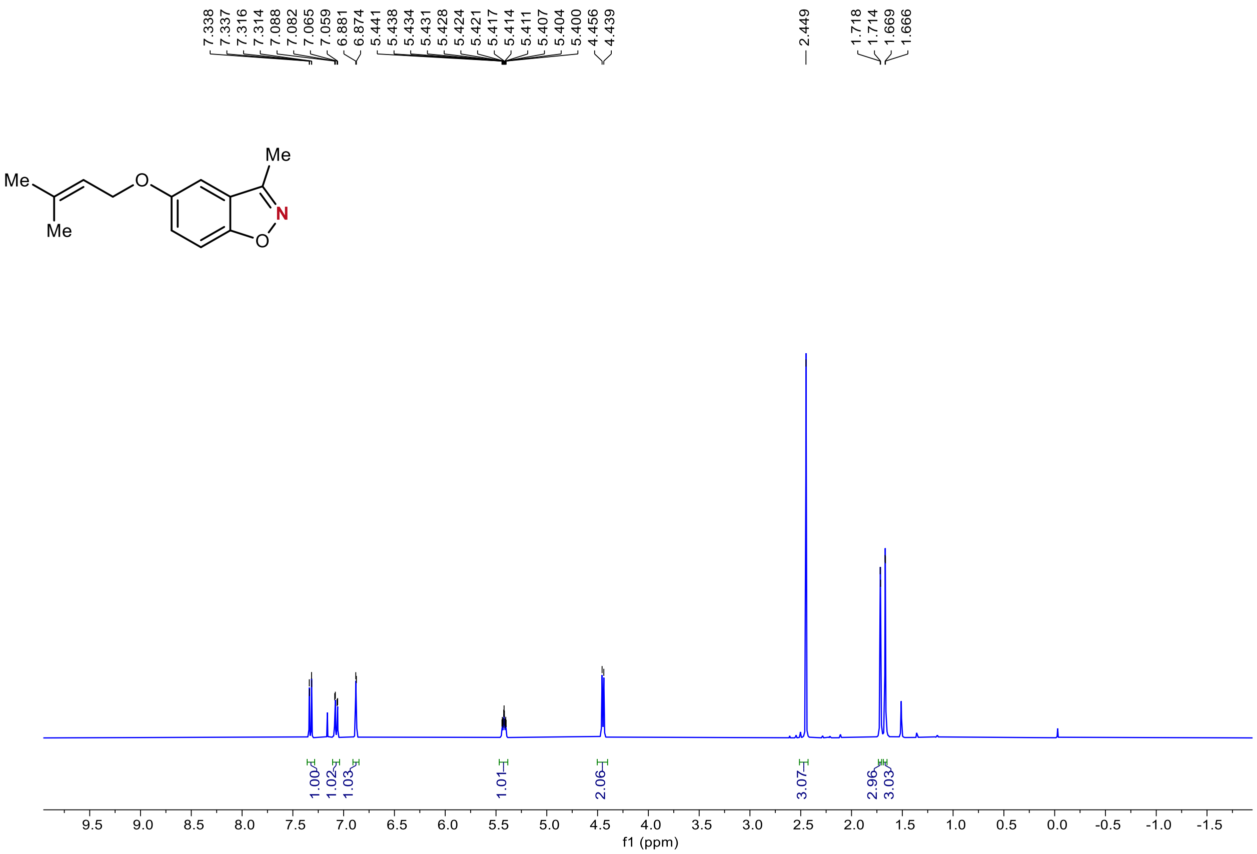

^{13}C NMR (101 MHz, $CDCl_3$)

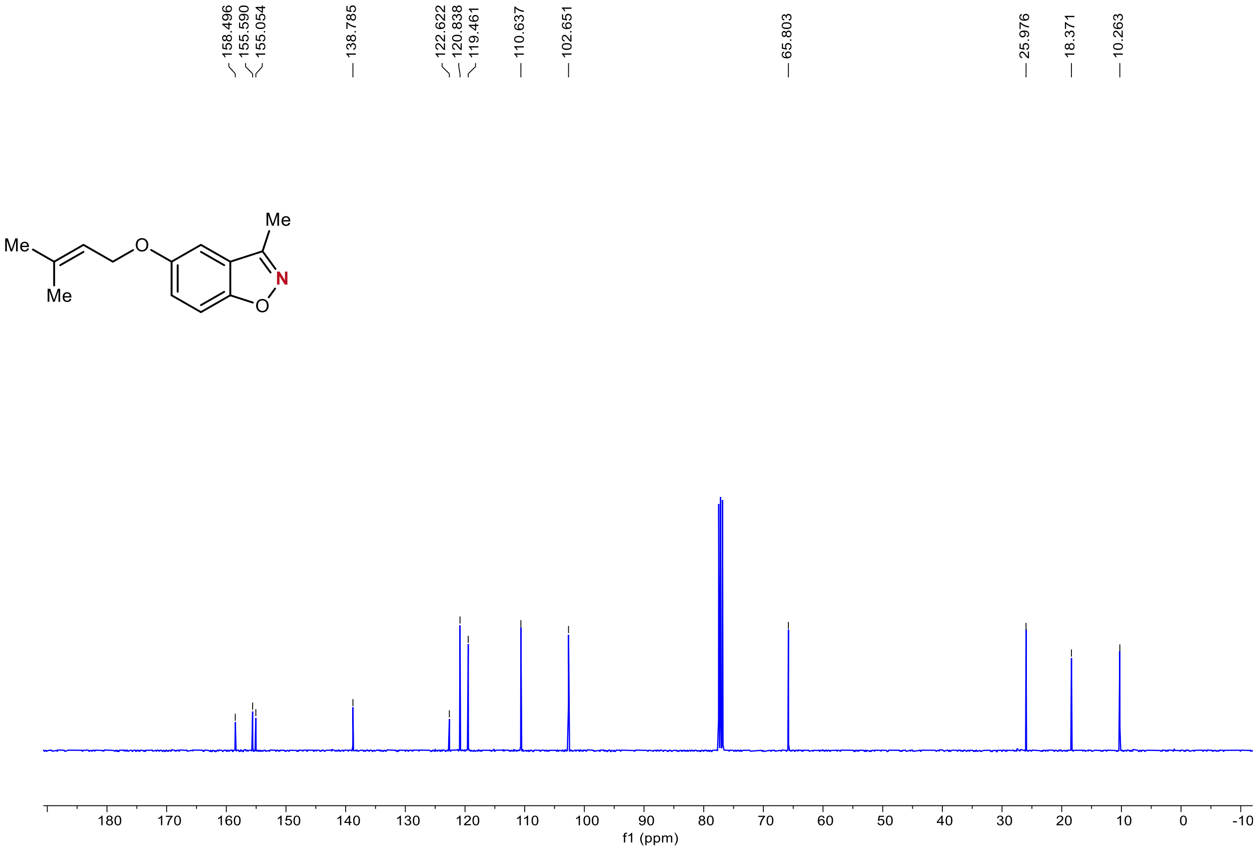

3-Methylbenzo[*d*]isoxazol-5-yl trifluoromethanesulfonate 5e

^{1}H NMR (400 MHz, $CDCl_3$)

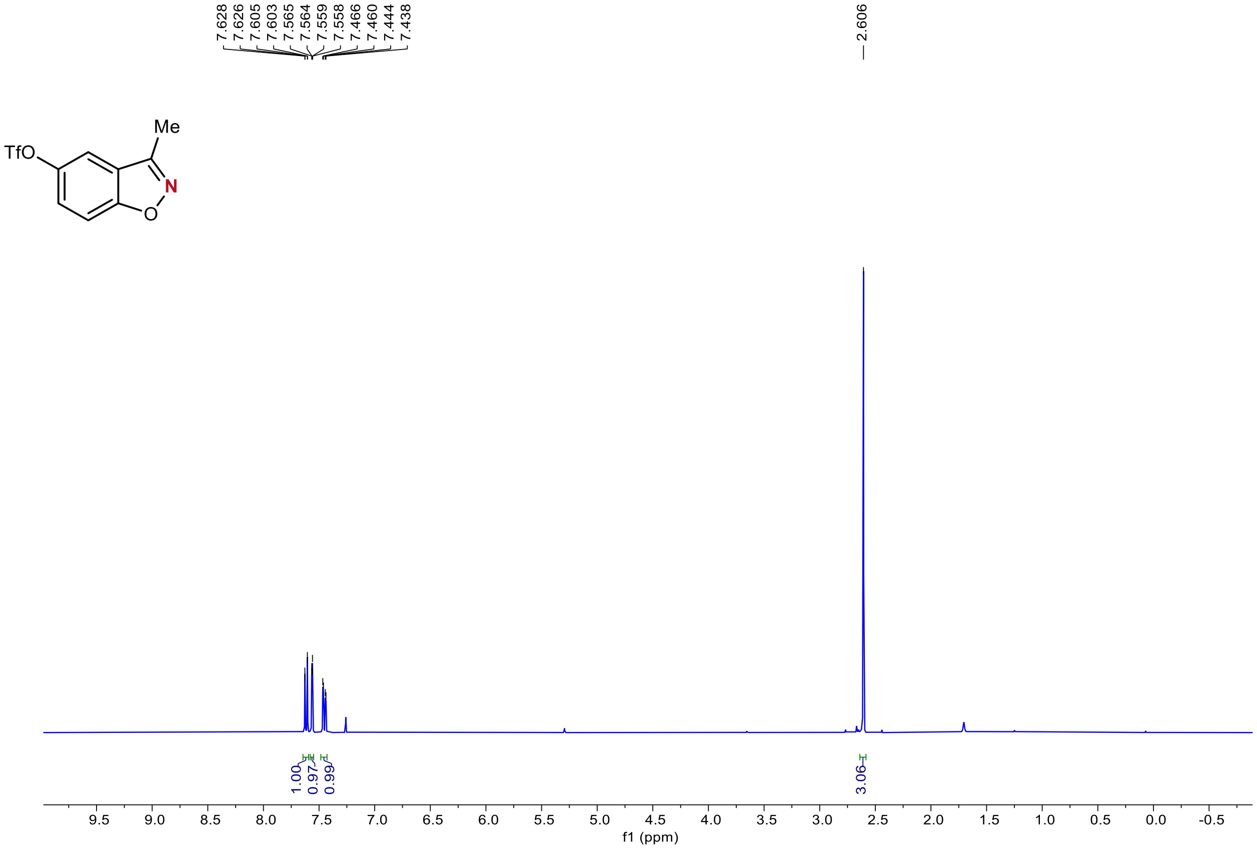

^{13}C NMR (101 MHz, $CDCl_3$)

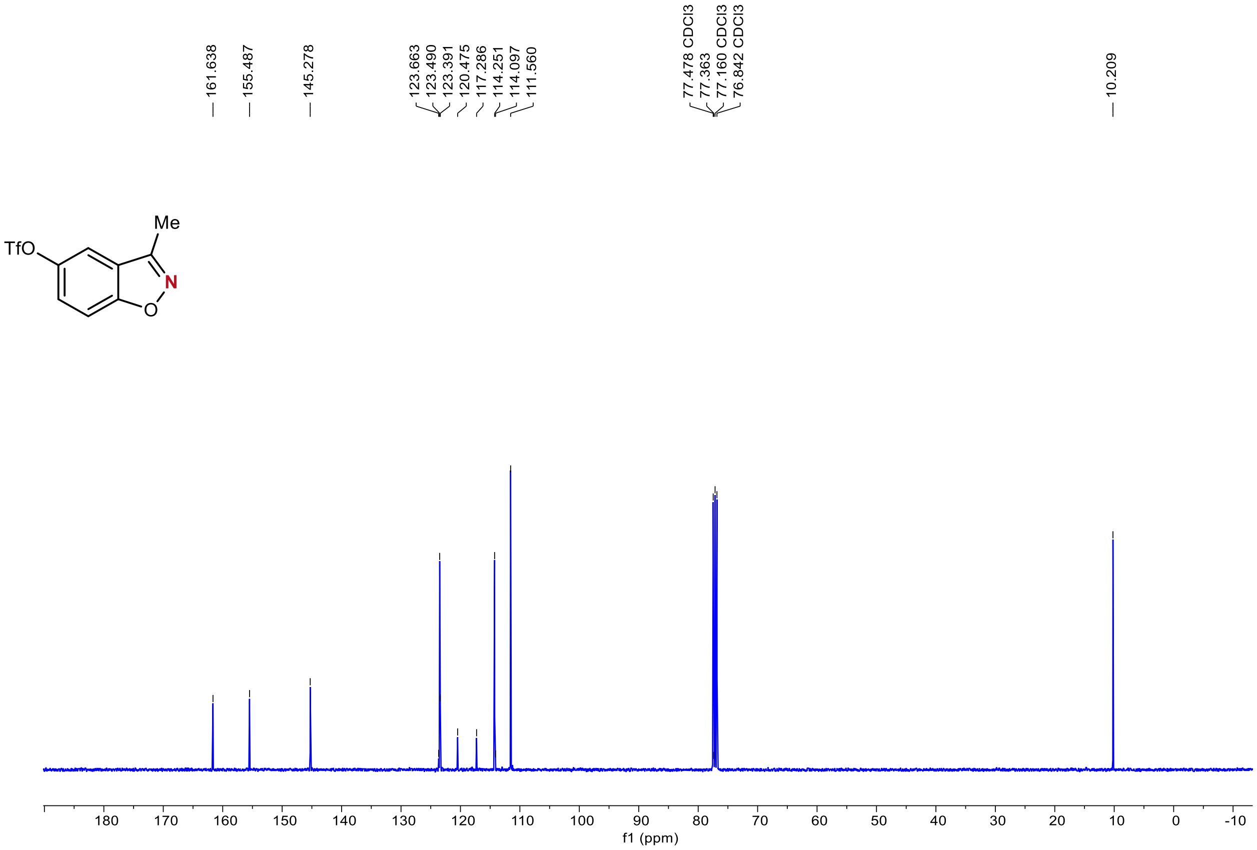

^{19}F NMR (282 MHz, $CDCl_3$)

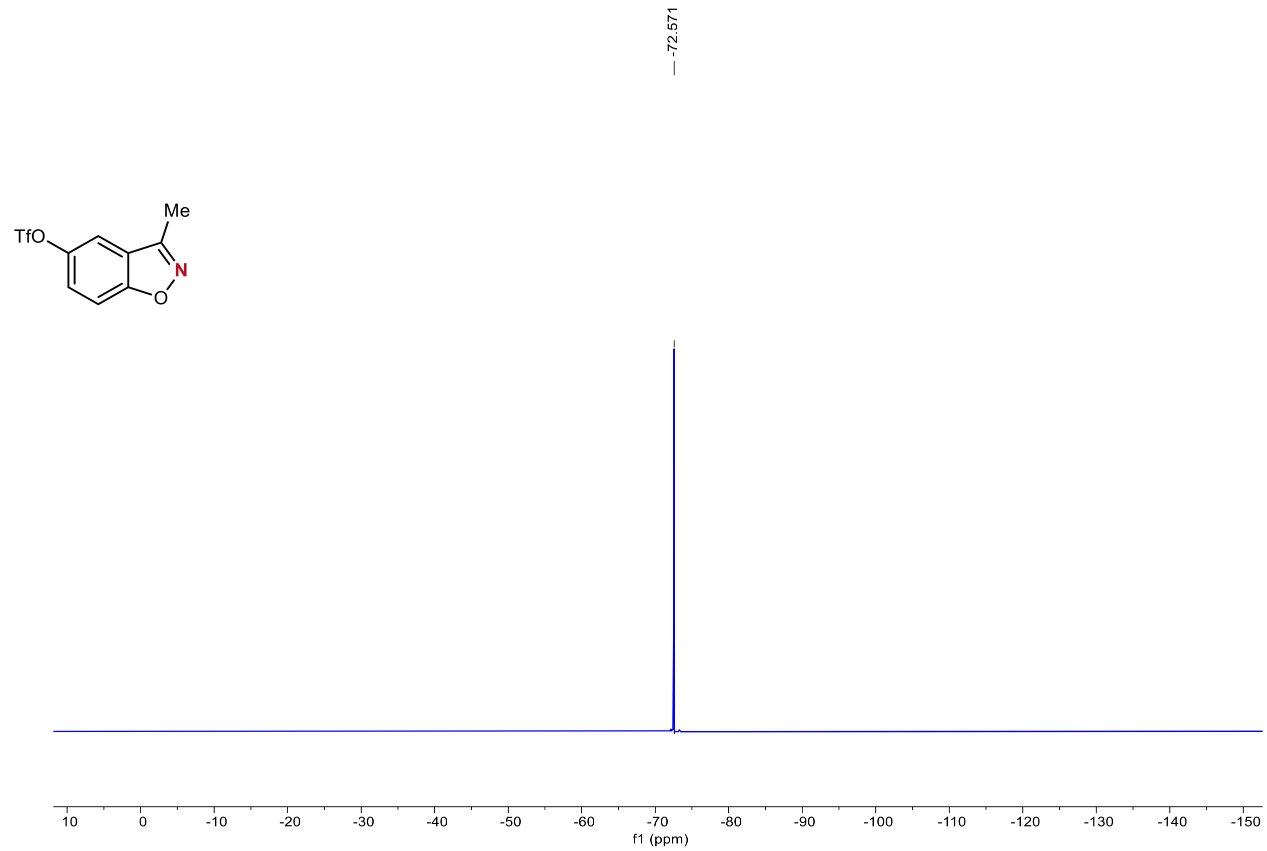

2-Methylbenzo[*d*]oxazol-5-yl trifluoromethanesulfonate 6e

^{1}H NMR (300 MHz, $CDCl_3$)

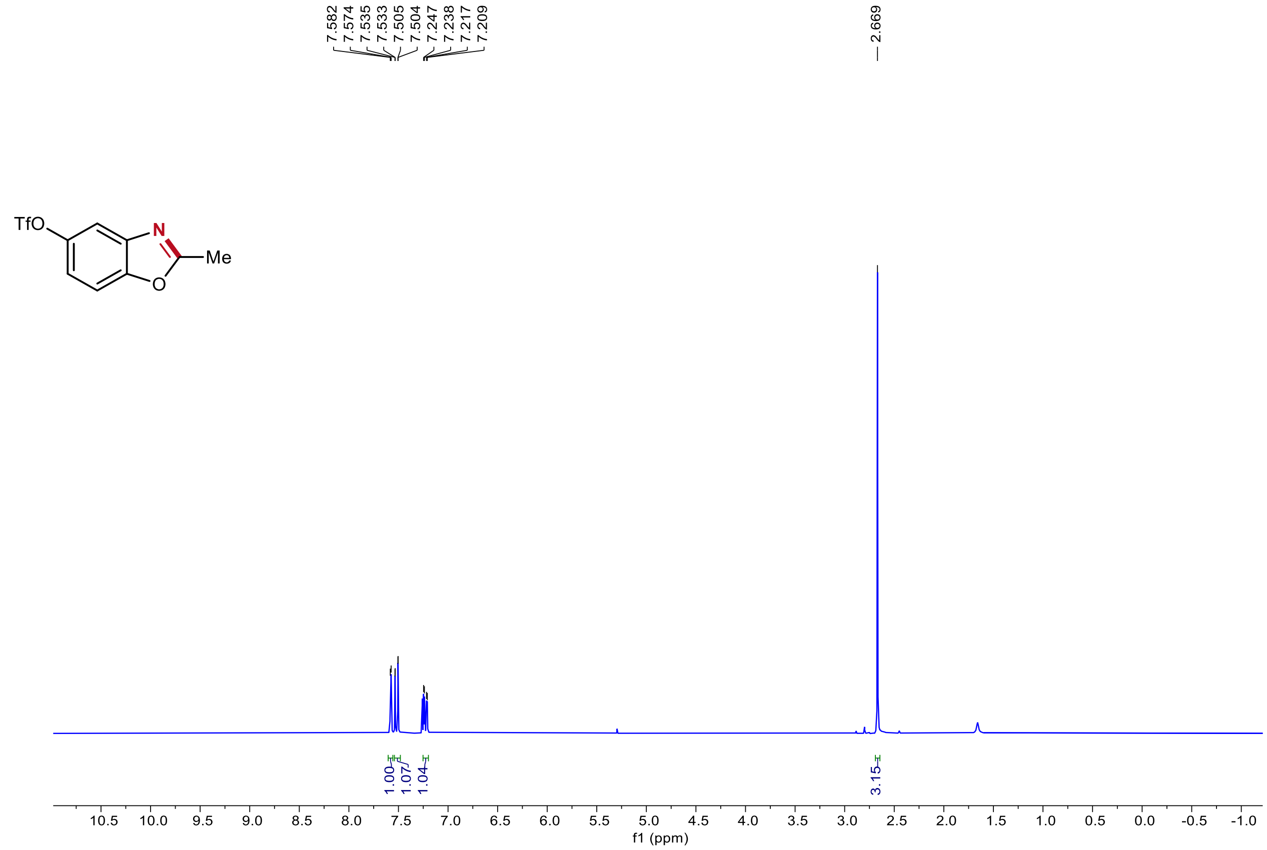

^{13}C NMR (76 MHz, $CDCl_3$)

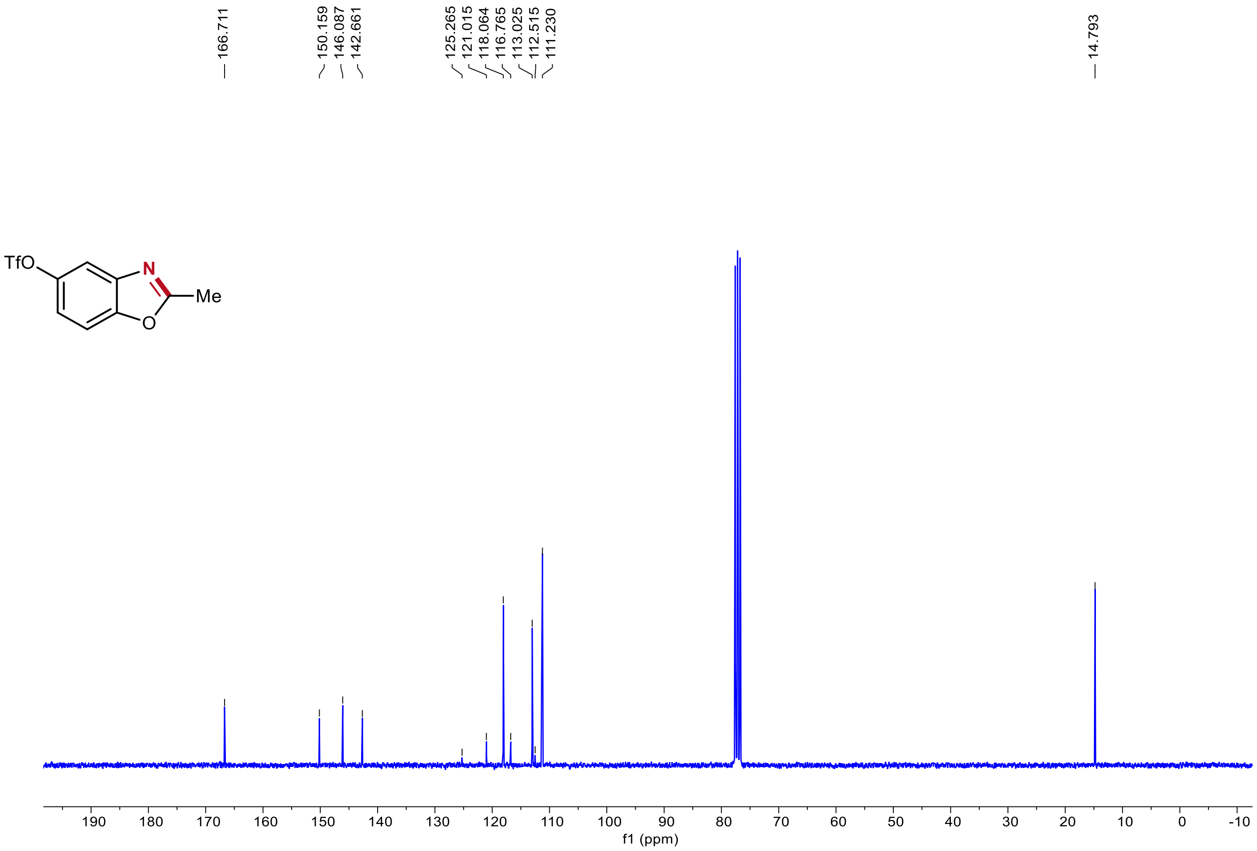

^{19}F NMR (282 MHz, $CDCl_3$)

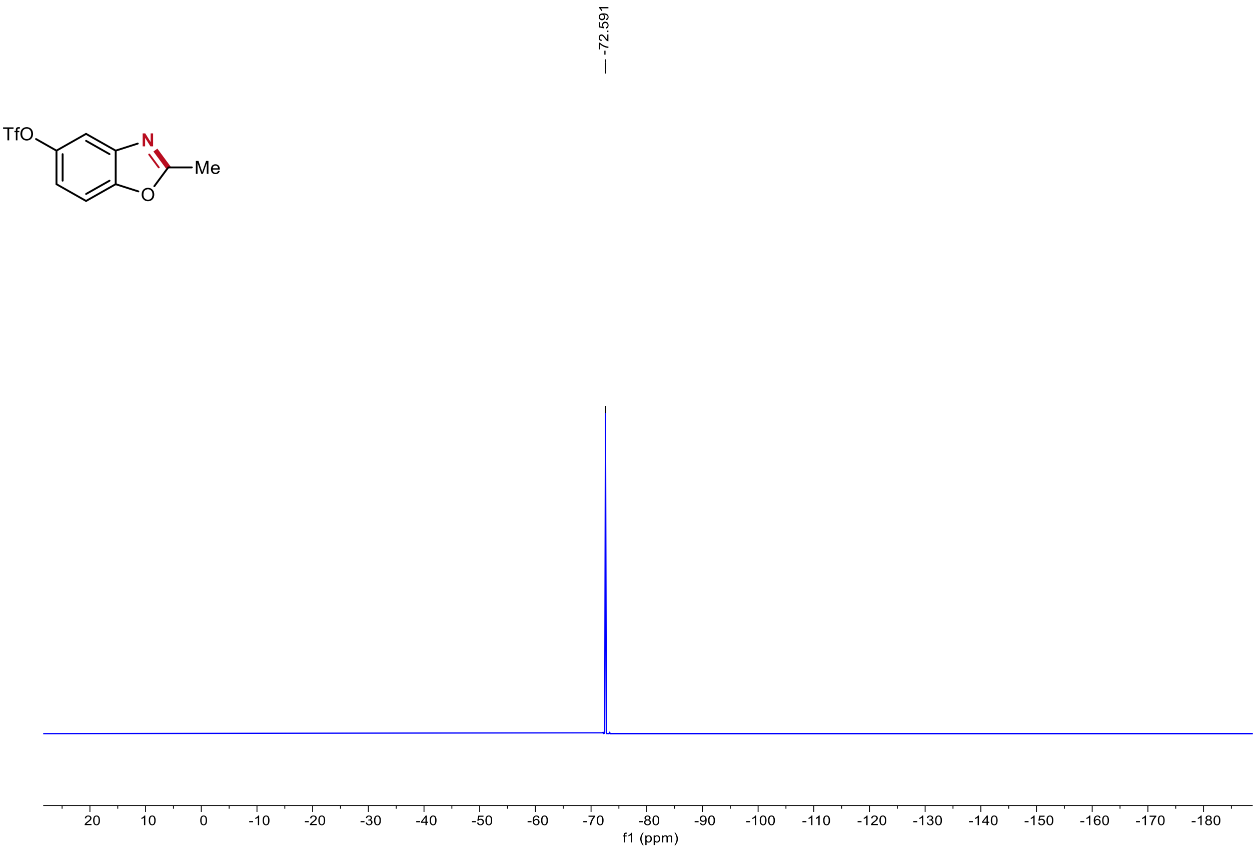

2-Methylbenzo[d]oxazol-5-yl trifluoromethanesulfonate 5f

^{1}H NMR (400 MHz, $CDCl_3$)

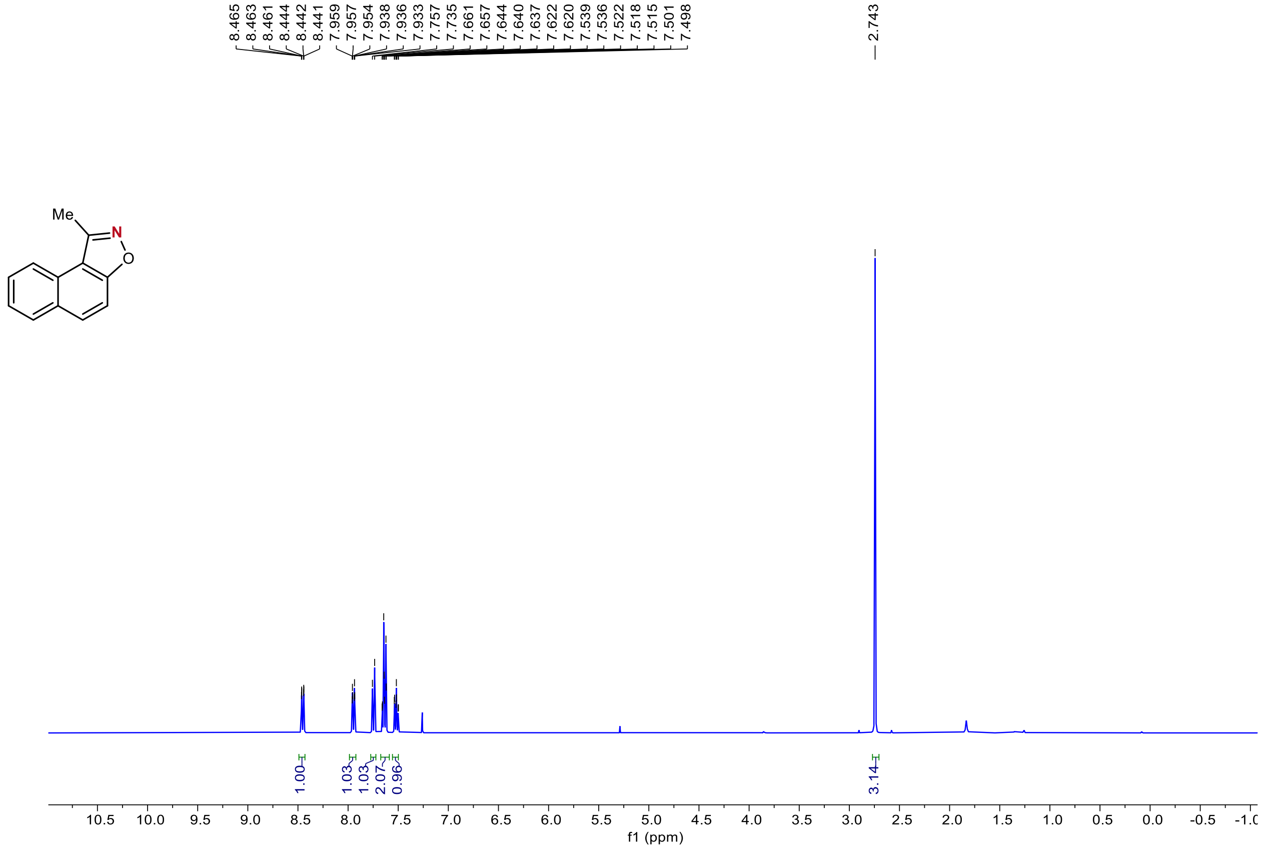

^{13}C NMR (101 MHz, $CDCl_3$)

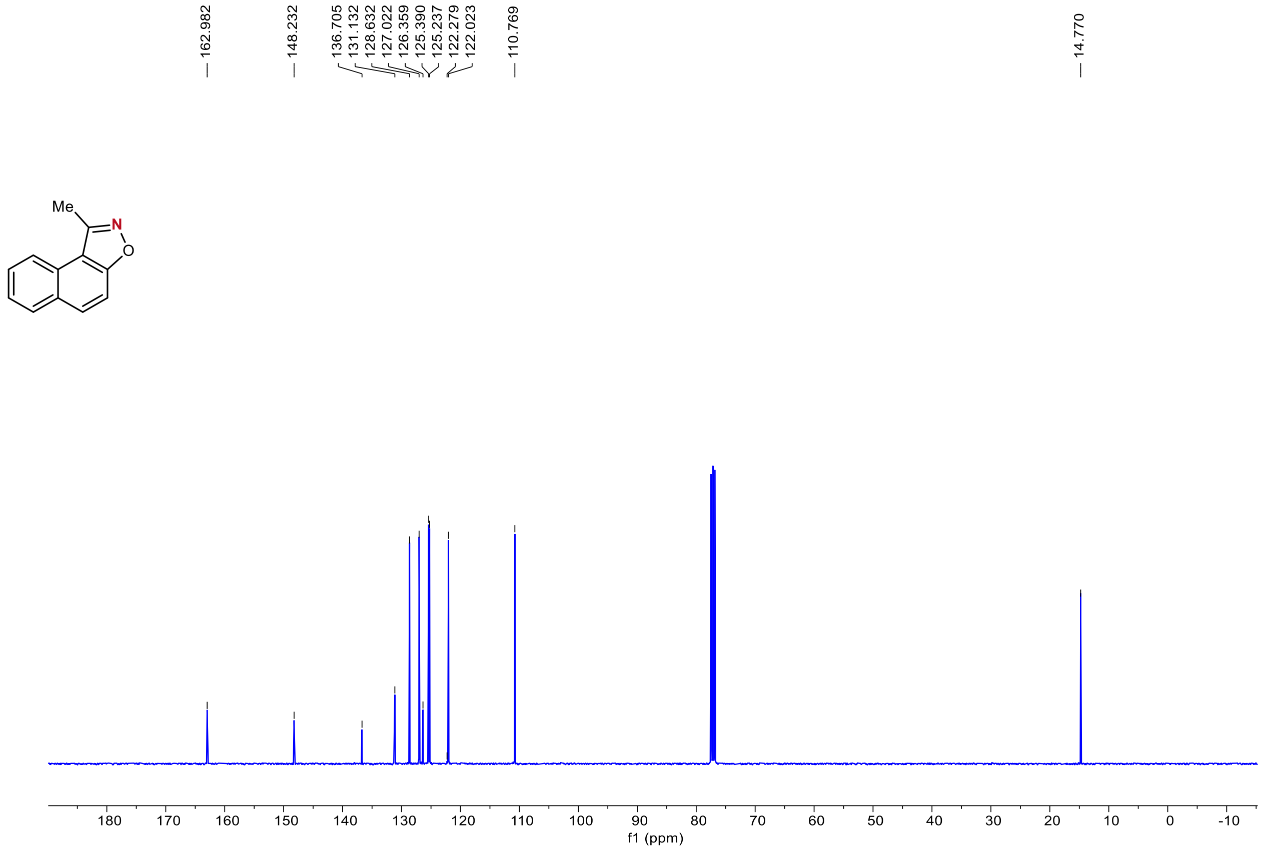

2-Methylnaphtho[1,2-*d*]oxazole 6f

^{1}H NMR (400 MHz, $CDCl_3$)

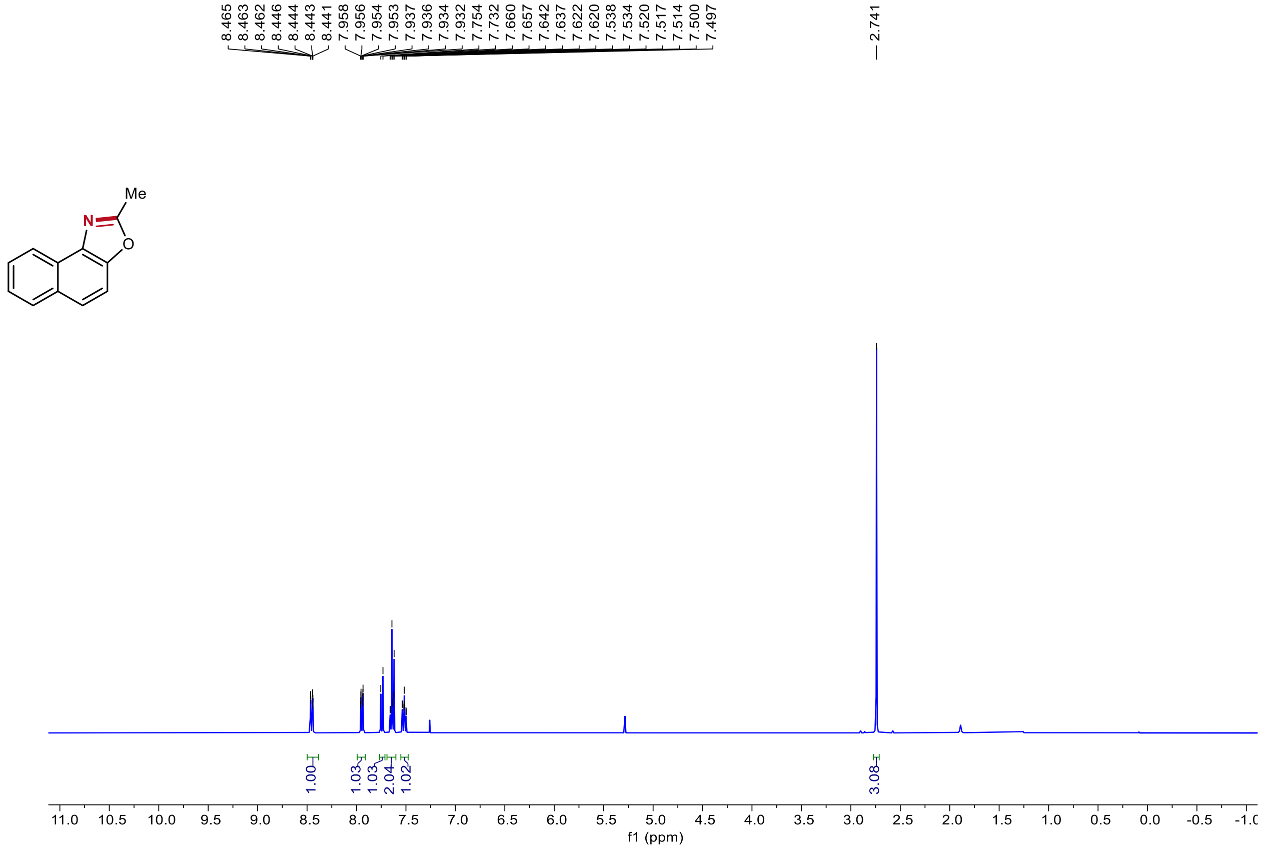

^{13}C NMR (101 MHz, $CDCl_3$)

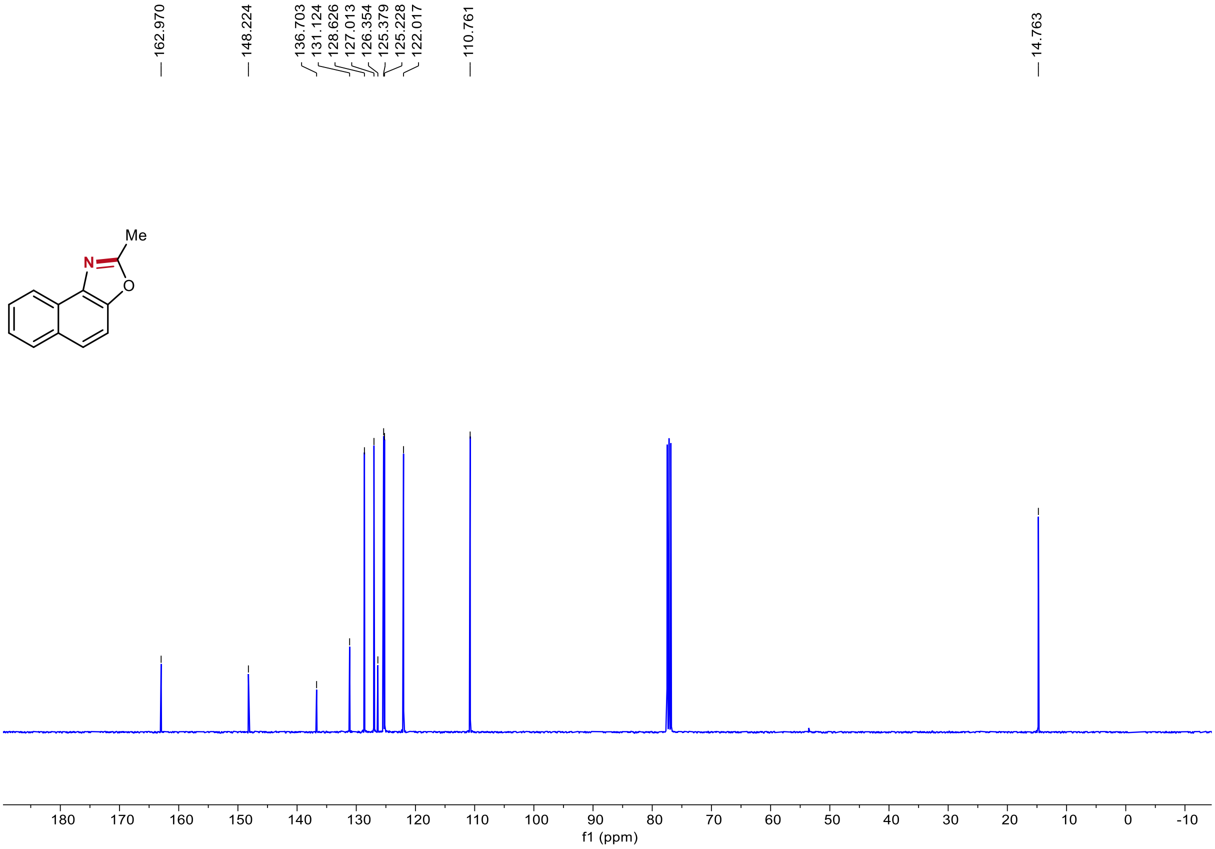

***N*-(2-**

(benzo[*d*]isoxazol-3-yl)ethyl)-2,2,2-trifluoroacetamide 5g

^{1}H NMR (400 MHz, $CDCl_3$)

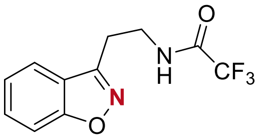

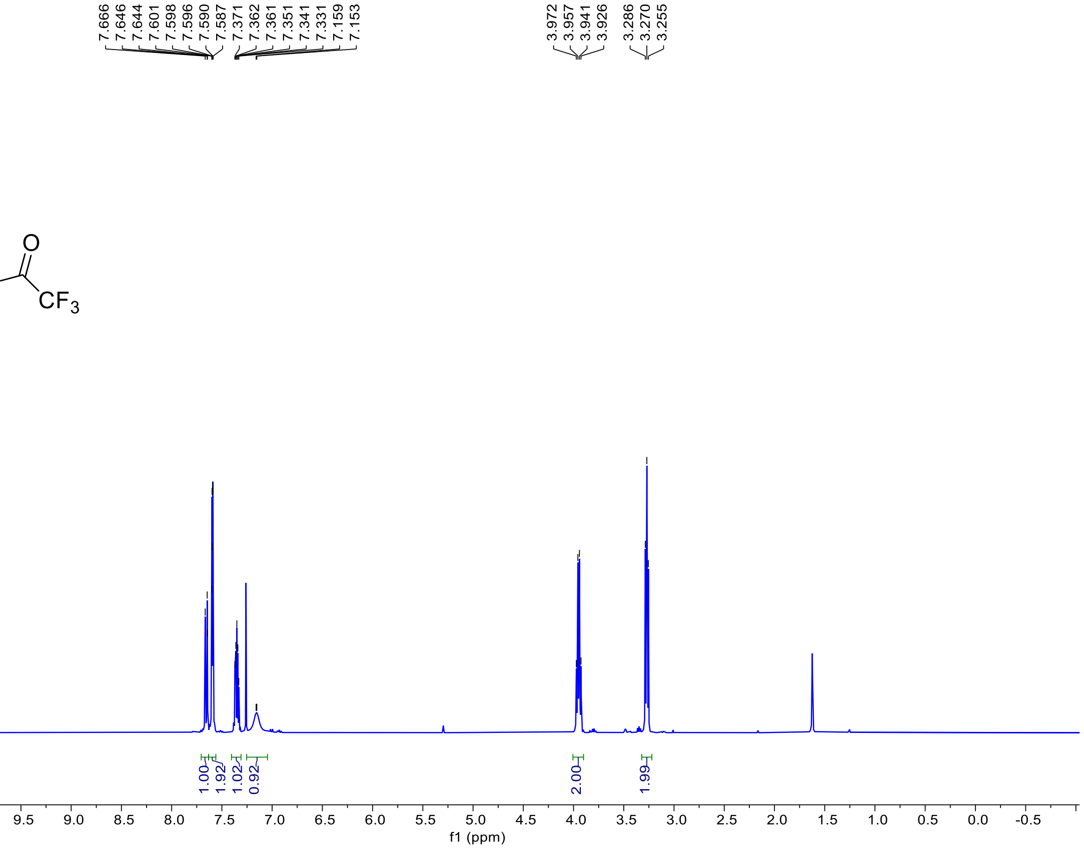

^{13}C NMR (101 MHz, $CDCl_3$)

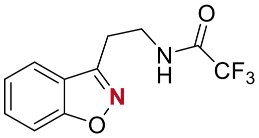

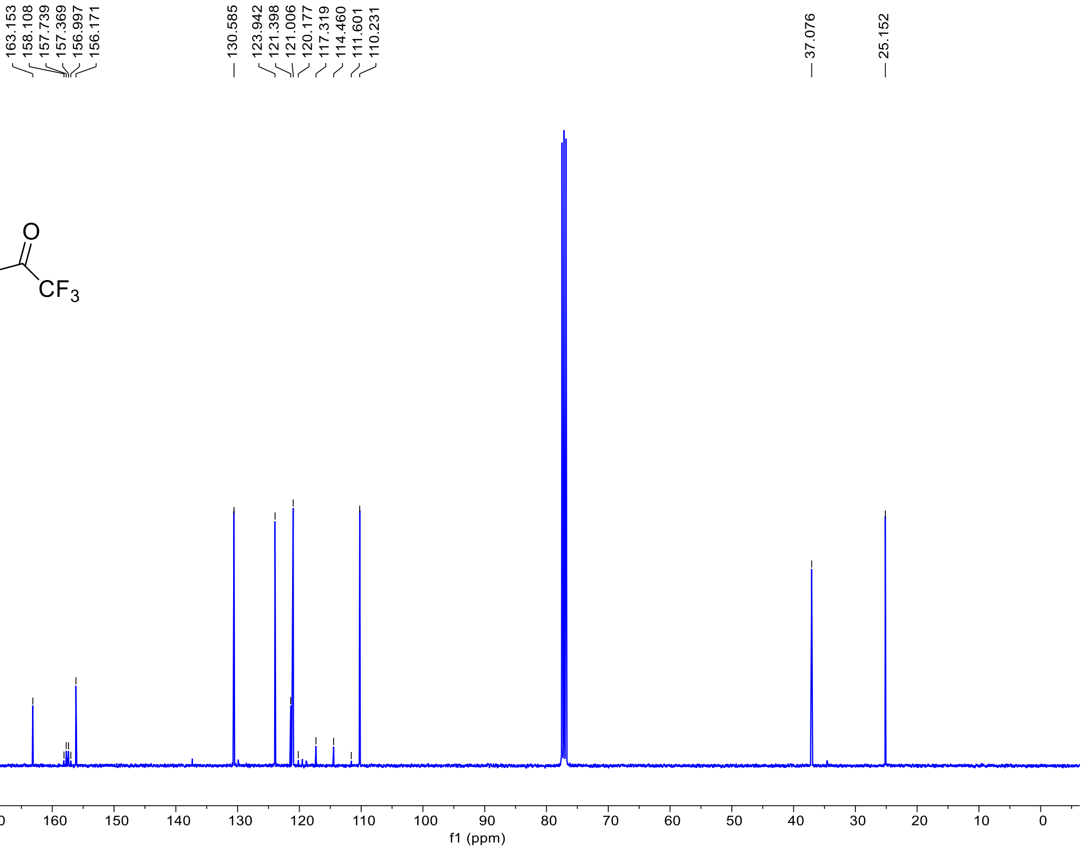

^{19}F NMR (376 MHz, $CDCl_3$)

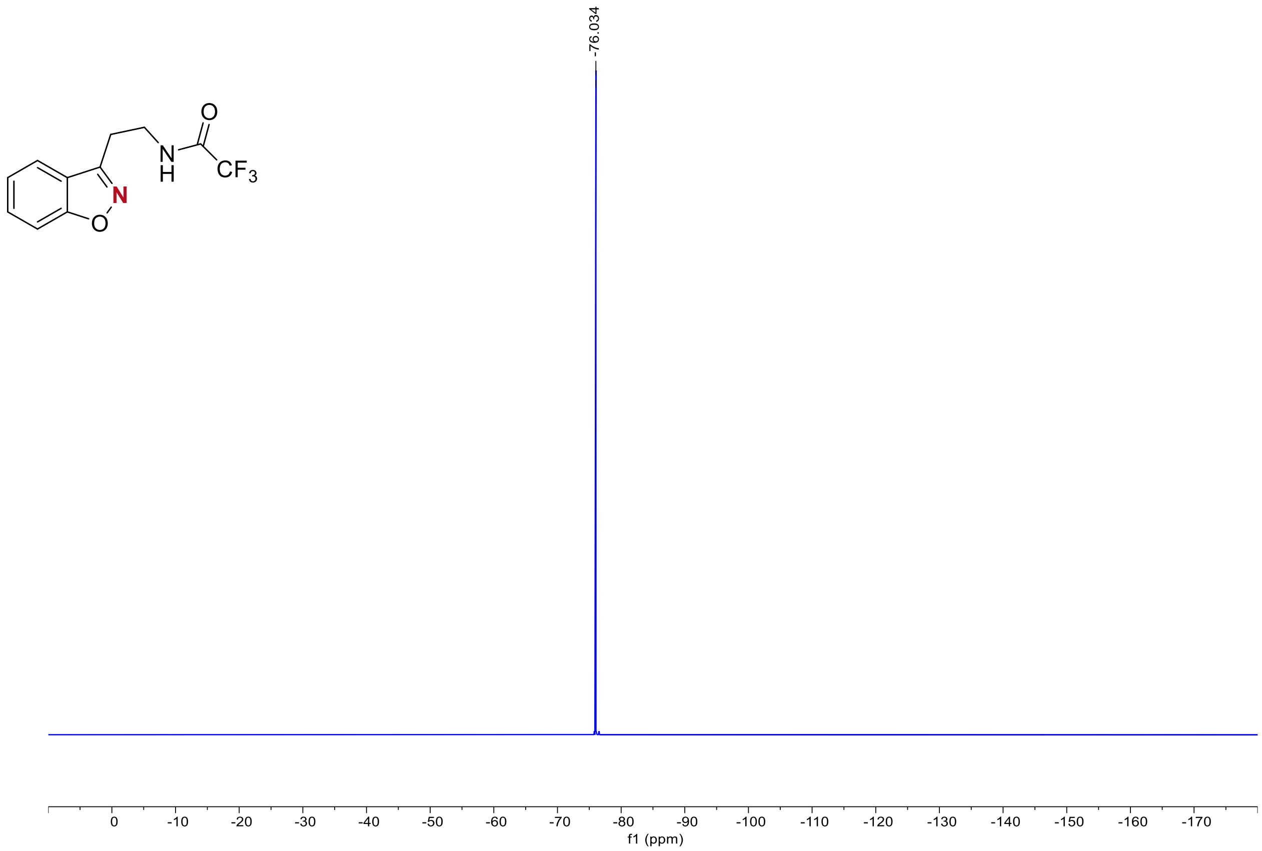

***N*-(2-(Benzo[*d*]oxazol-2-yl)ethyl)-2,2,2-trifluoroacetamide 6g**

^{1}H NMR (300 MHz, $CDCl_3$)

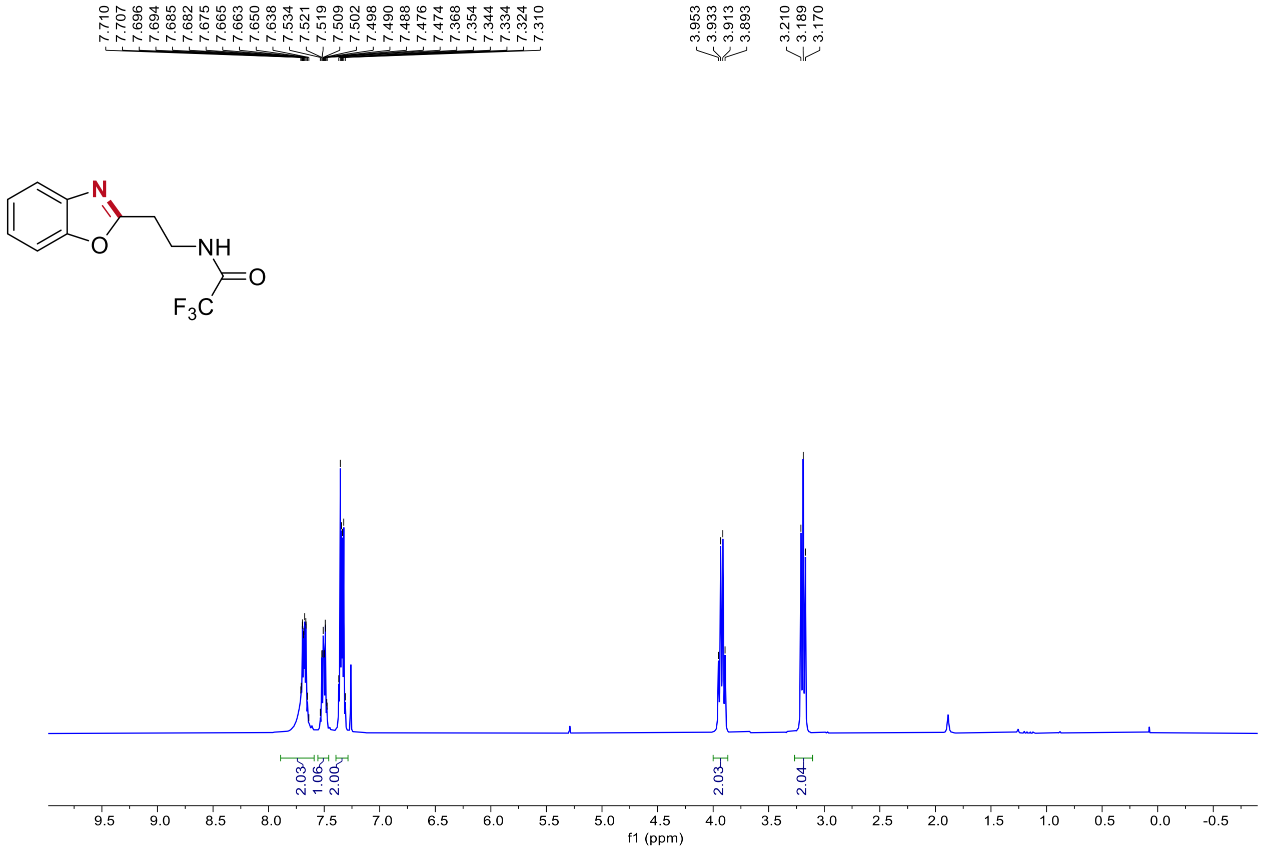

^{13}C NMR (76 MHz, $CDCl_3$)

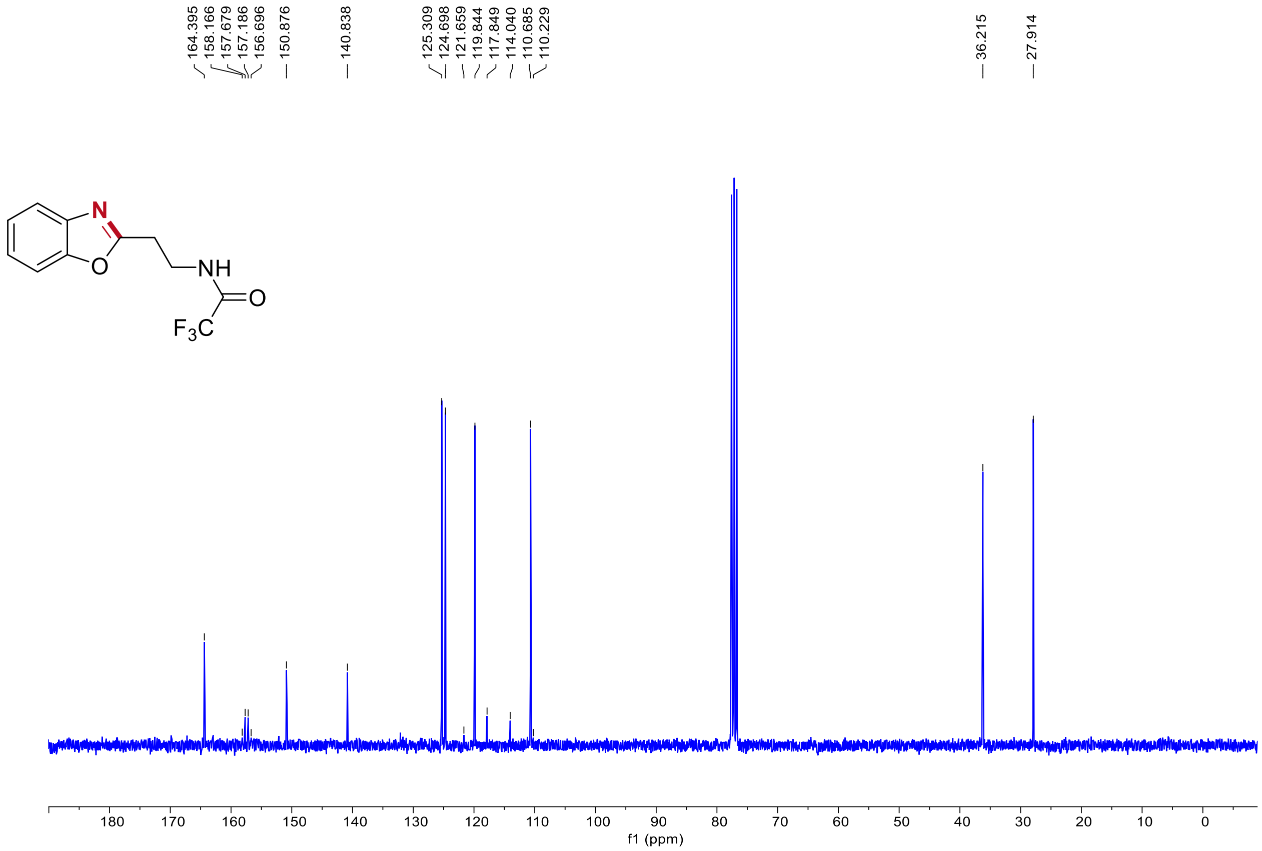

^{19}F NMR (282 MHz, $CDCl_3$)

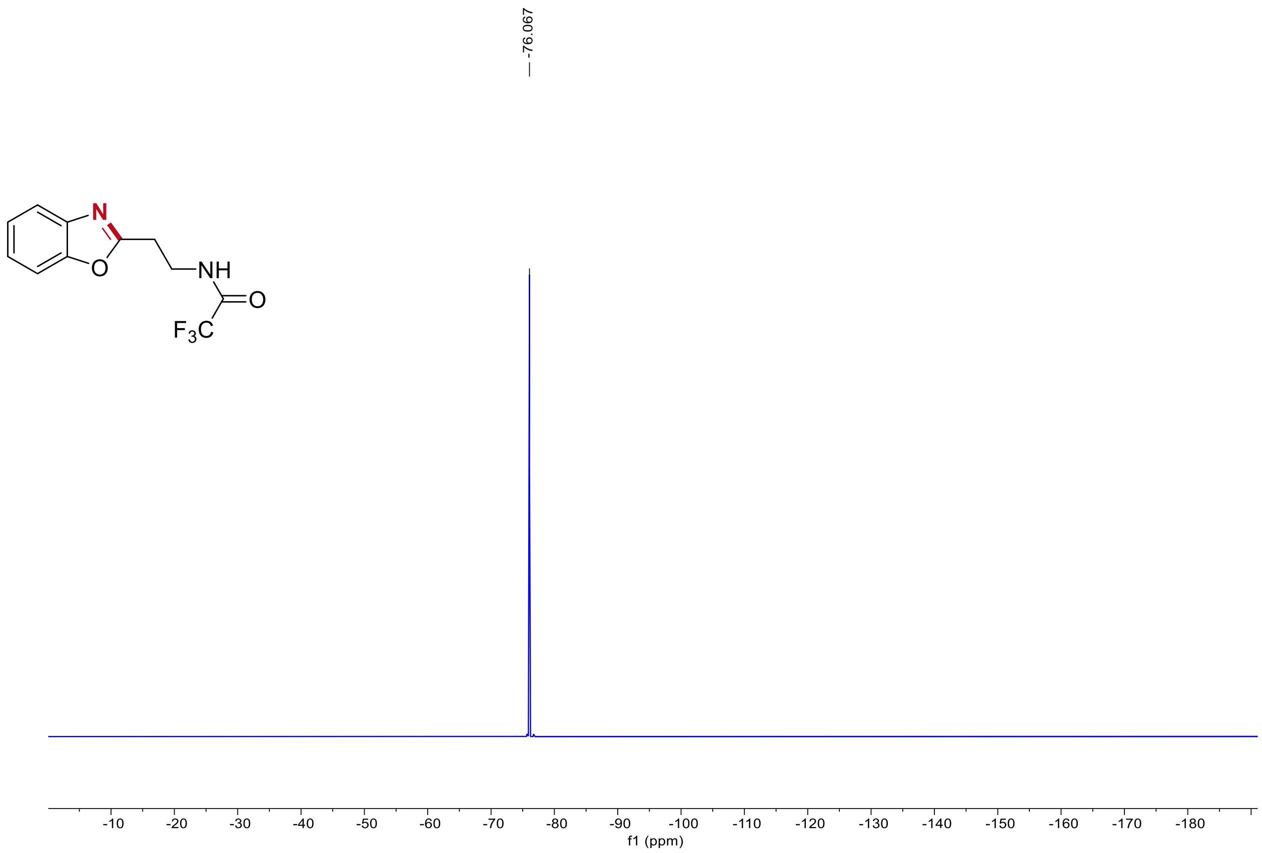

3-(((Triisopropylsilyl)oxy)methyl)benzo[*d*]isoxazole 5h

^{1}H NMR (300 MHz, $CDCl_3$)

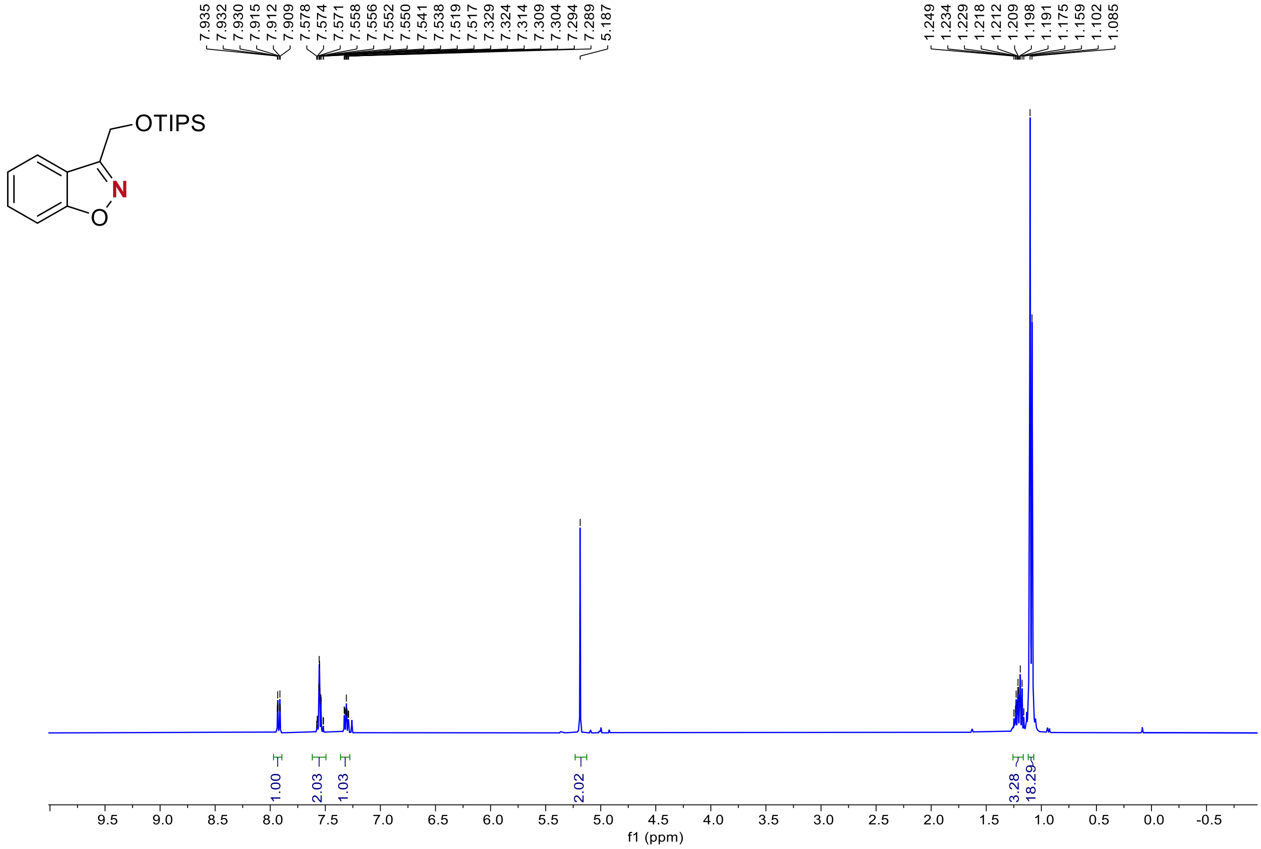

^{13}C NMR (101 MHz, $CDCl_3$)

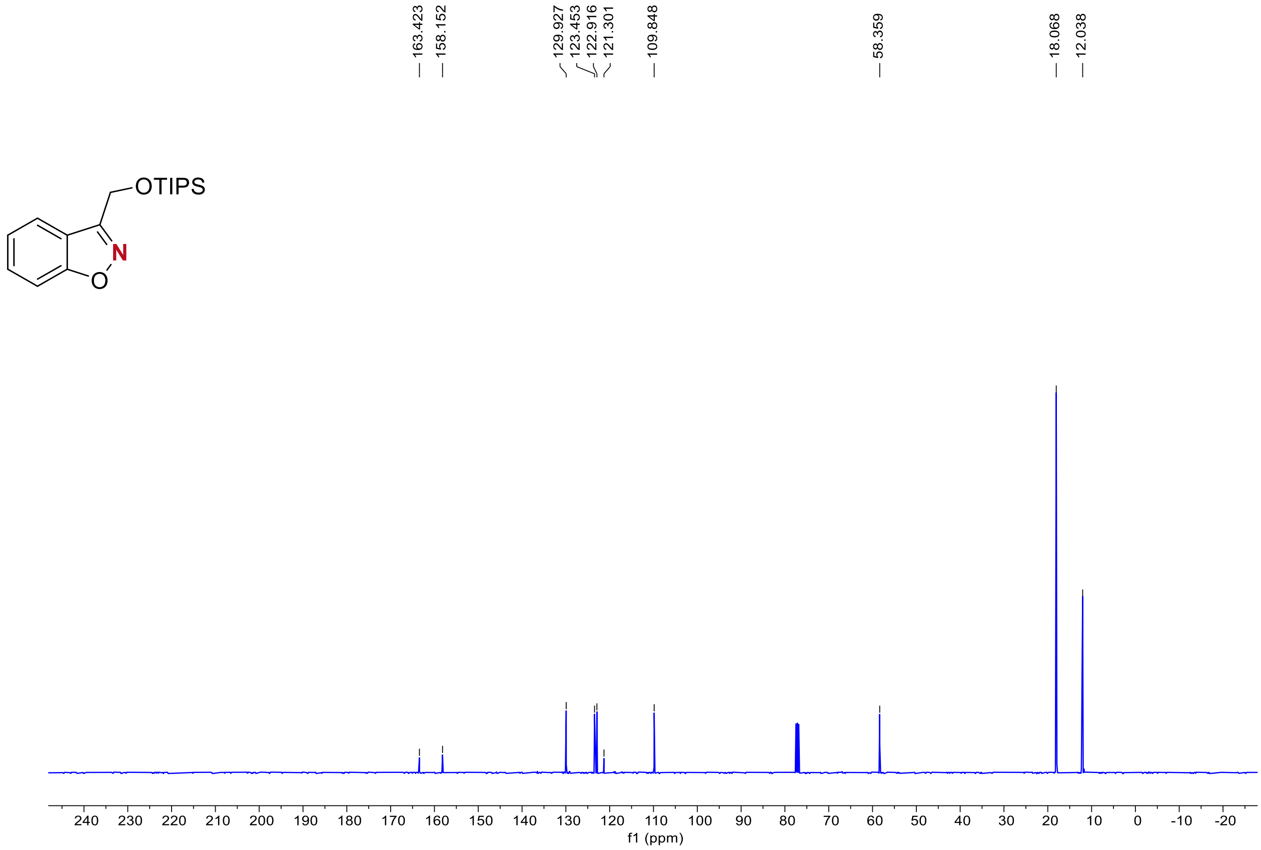

2-(5-Aminoisoxazol-3-yl)phenol 5i

^{1}H NMR (400 MHz, $CDCl_3$)

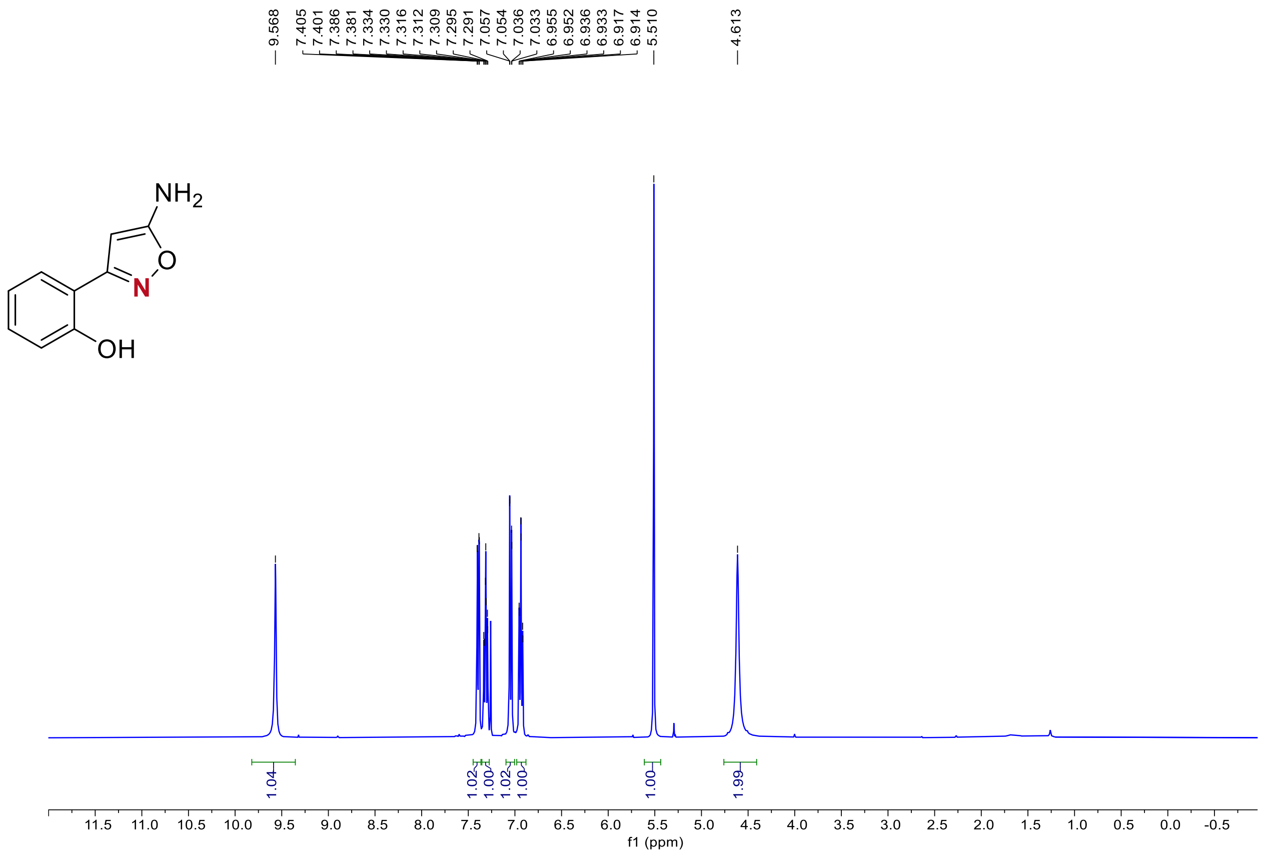

^{13}C NMR (76 MHz, $CDCl_3$)

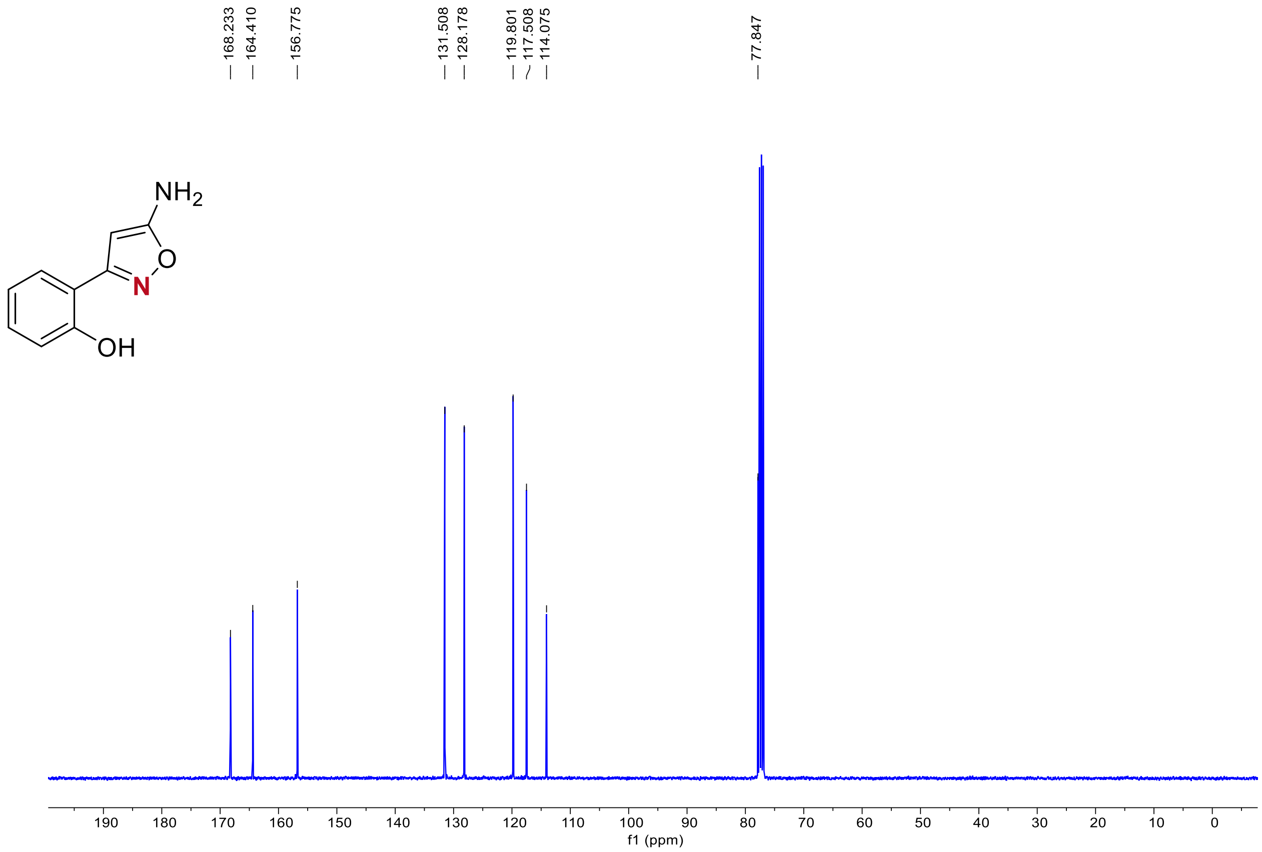

(Benzo[*d*]oxazol-2-ylmethyl)diphenylphosphine oxide 6j

^{1}H NMR (400 MHz, $CDCl_3$)

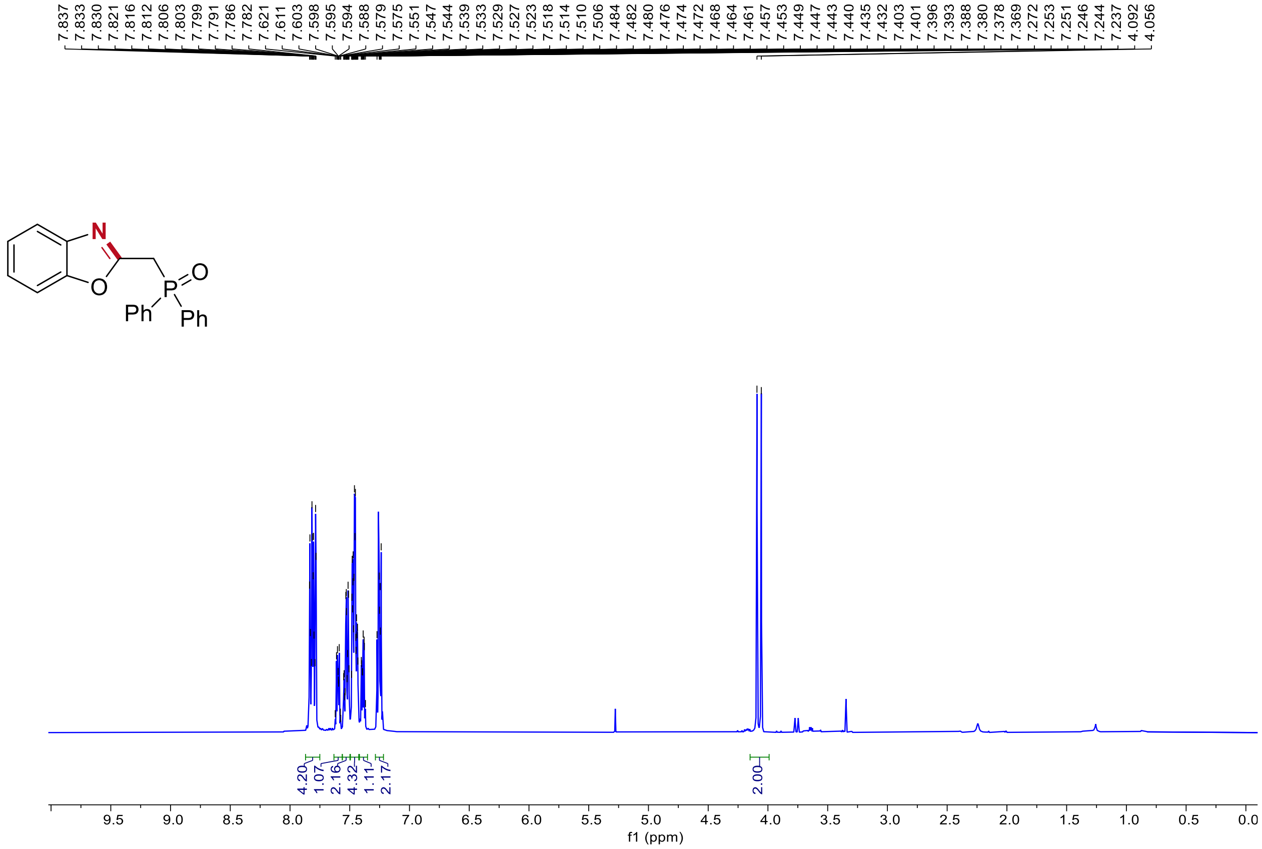

^{13}C NMR (101 MHz, $CDCl_3$)

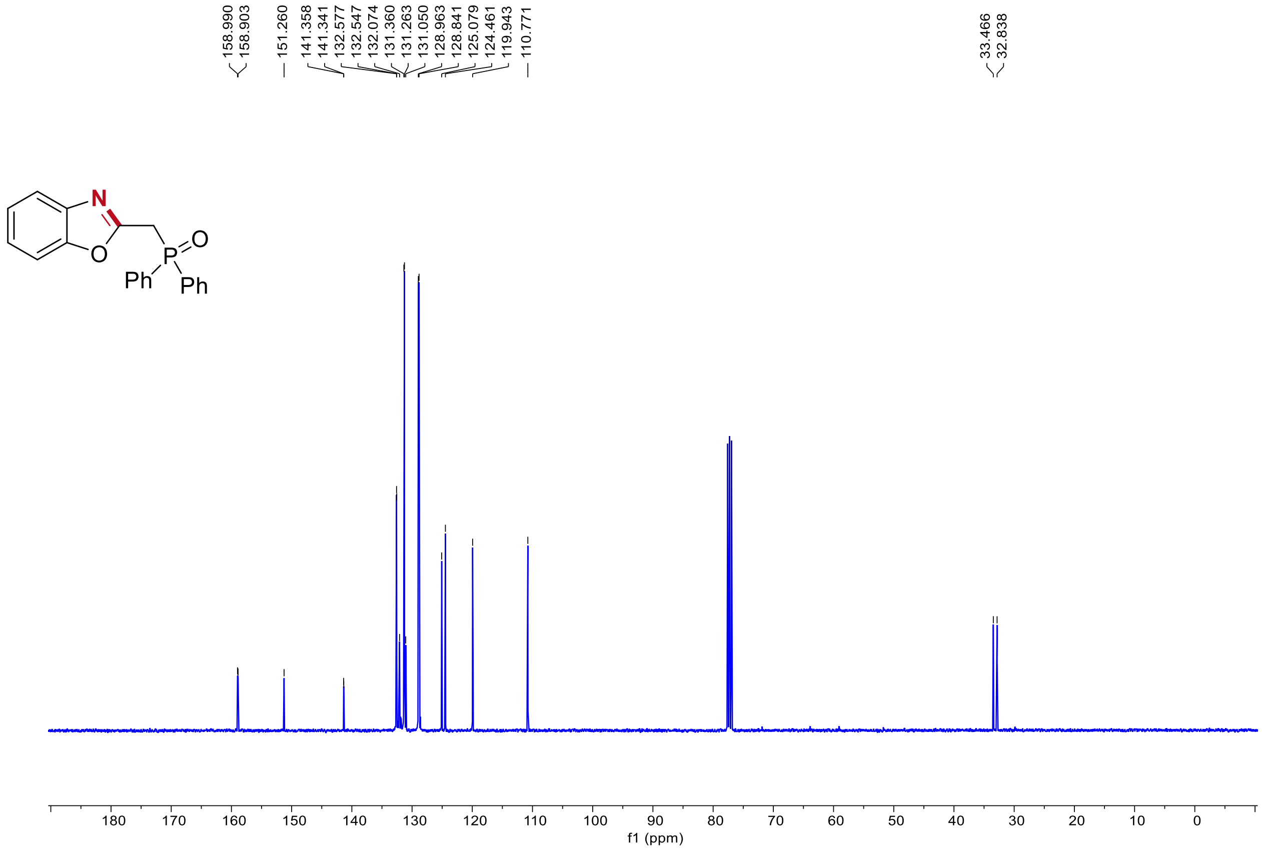

^{31}**P NMR** (162 MHz, $CDCl_3$)

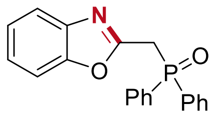

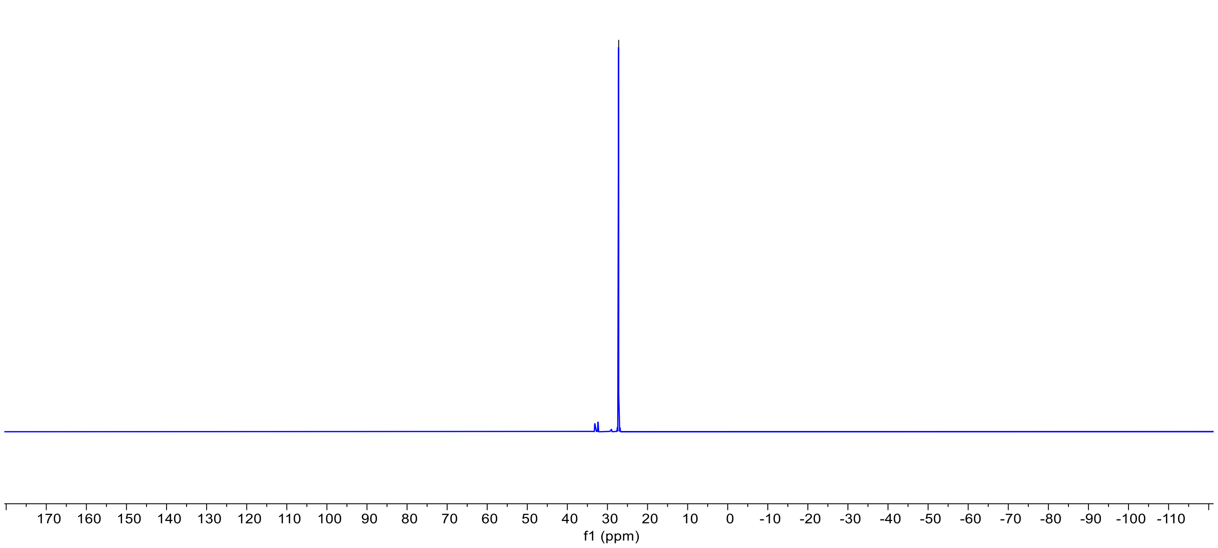

Methyl 3-(3-((2-(benzo[*d*]oxazol-2-yl)ethyl)amino)-3-oxopropyl)-1*H*-indole-1-carboxylate 6k

^{1}H NMR (400 MHz, $CDCl_3$)

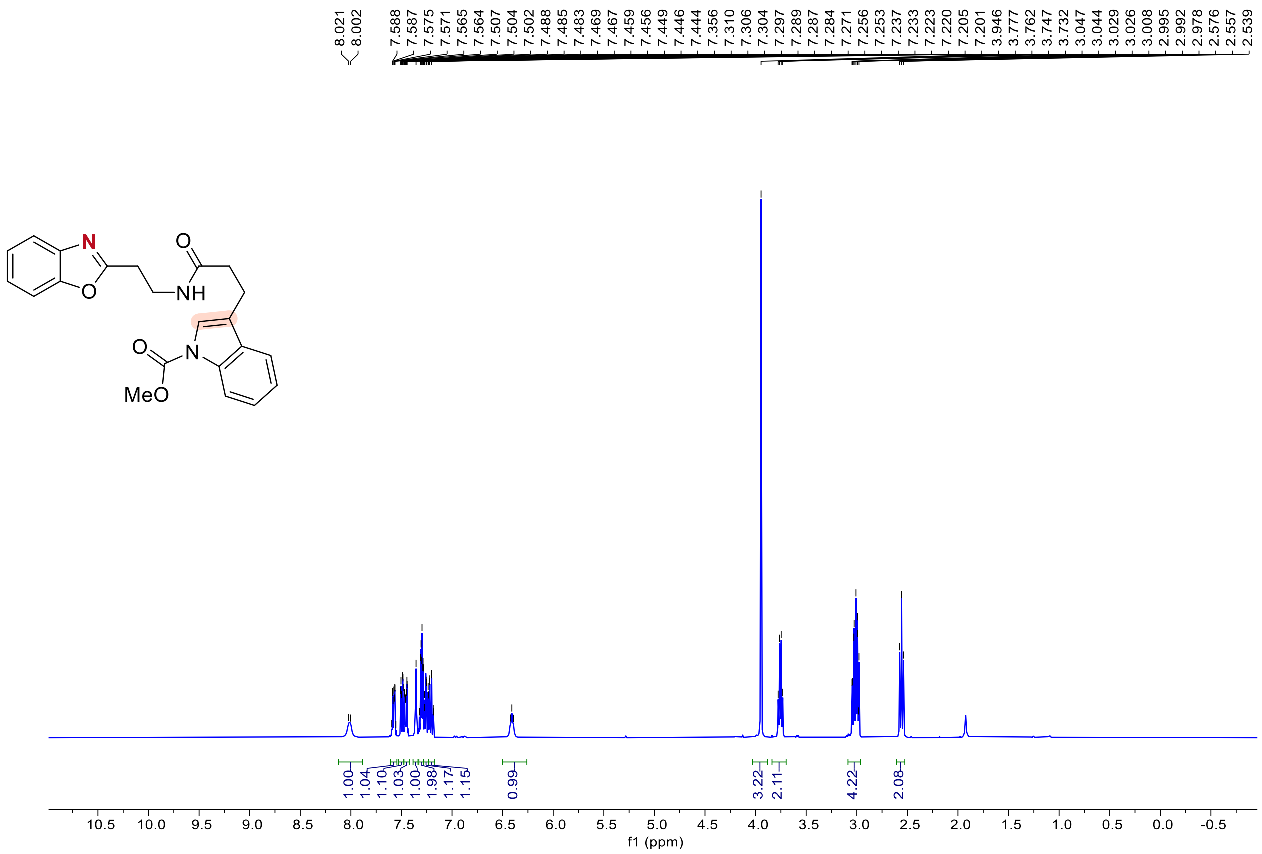

^{13}C NMR (101 MHz, $CDCl_3$)

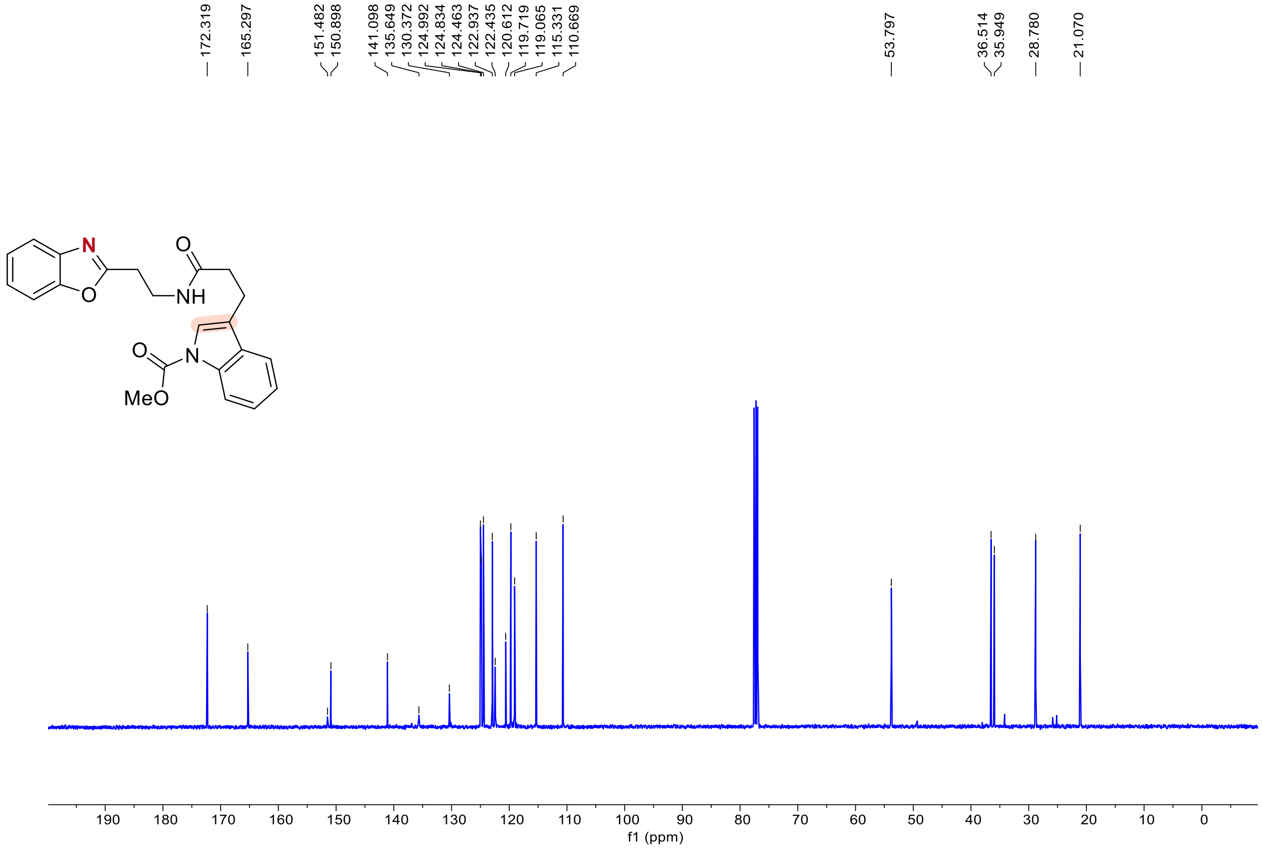

2-Phenylbenzo[*d*]oxazole 6m

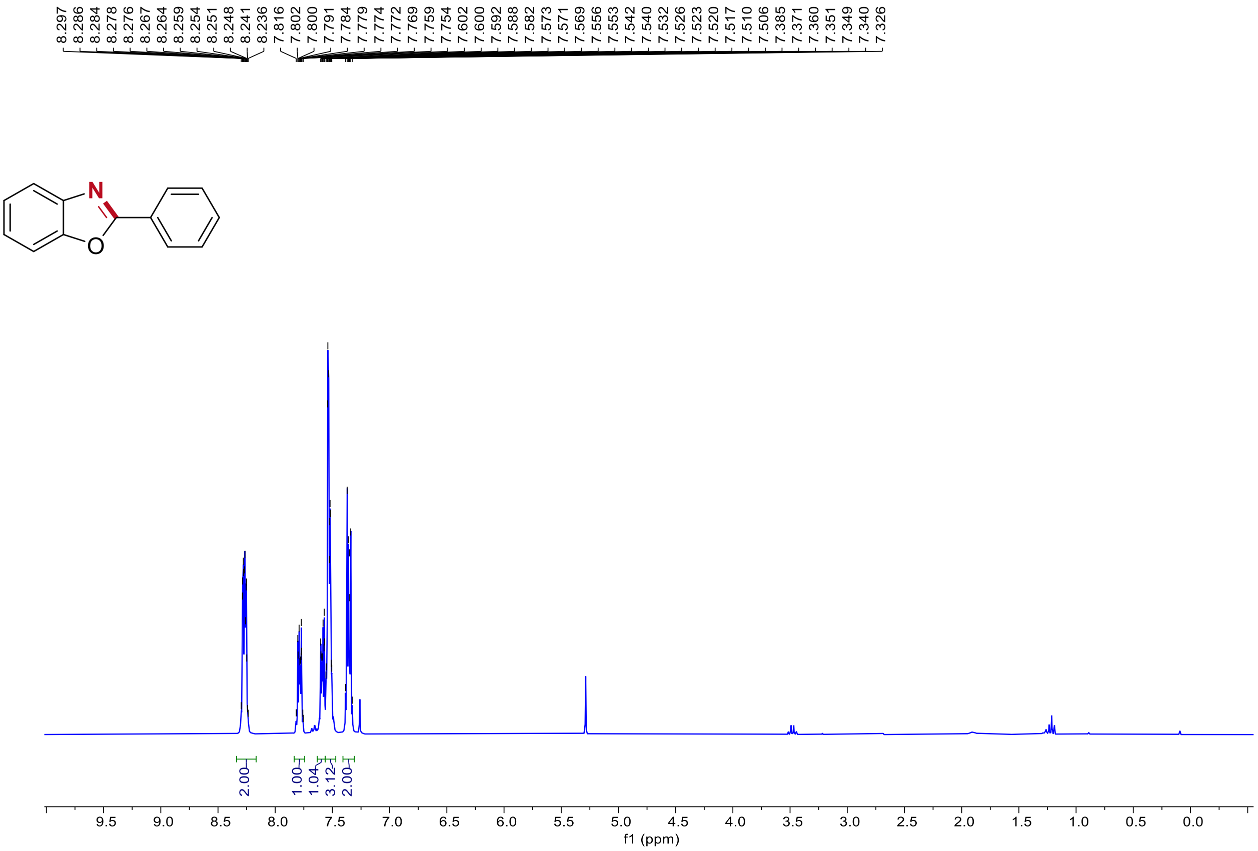

^{13}C NMR (76 MHz, $CDCl_3$)

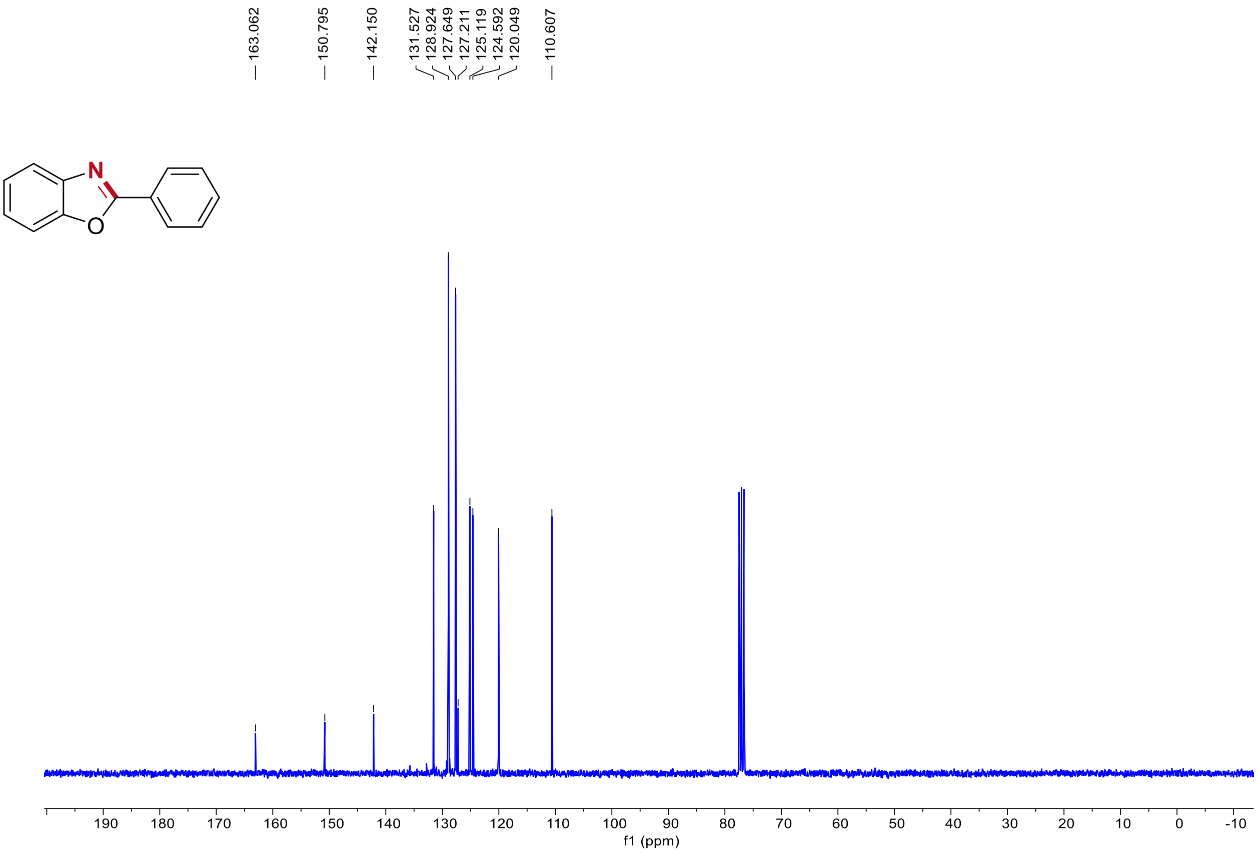

3-Isopropylbenzo[*d*]isoxazole 5n

^{1}H NMR (400 MHz, $CDCl_3$)

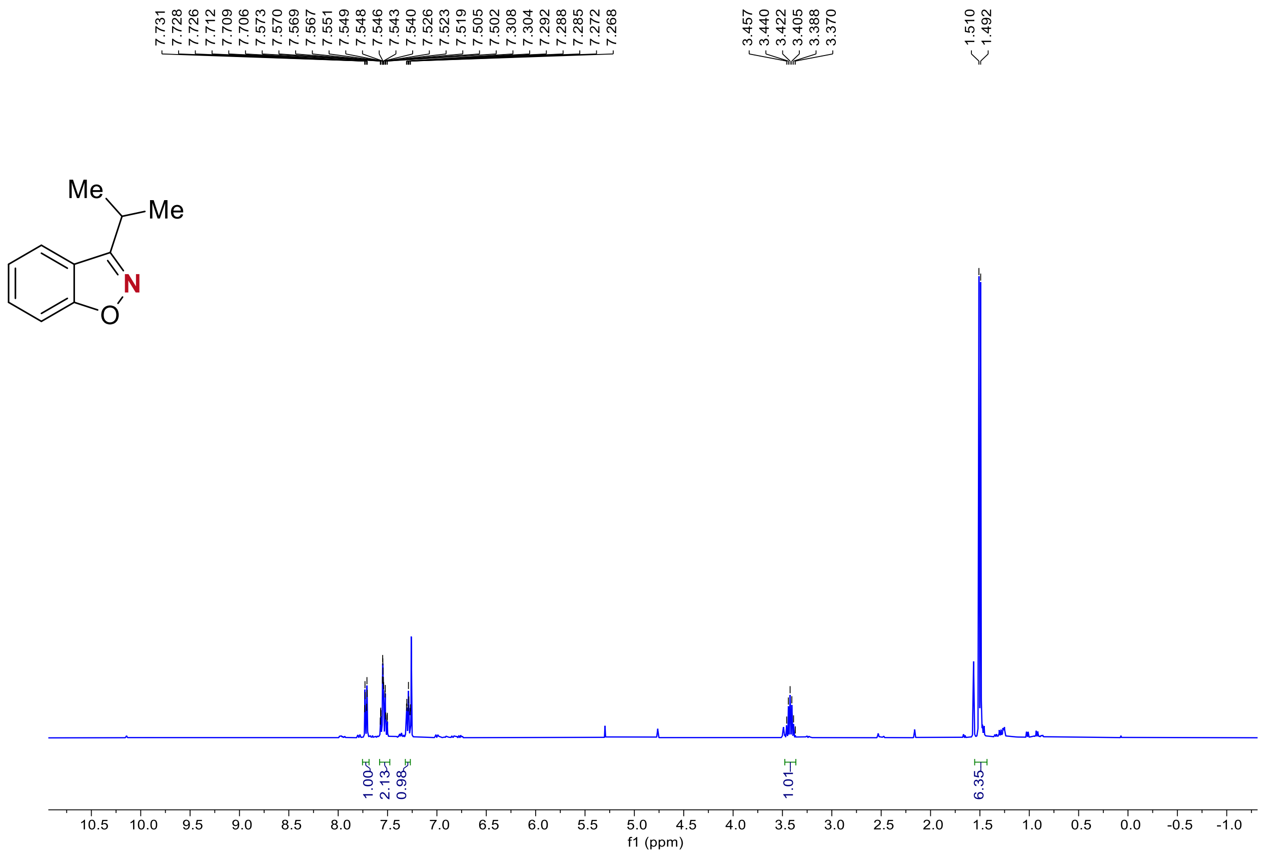

^{13}C NMR (101 MHz, $CDCl_3$)

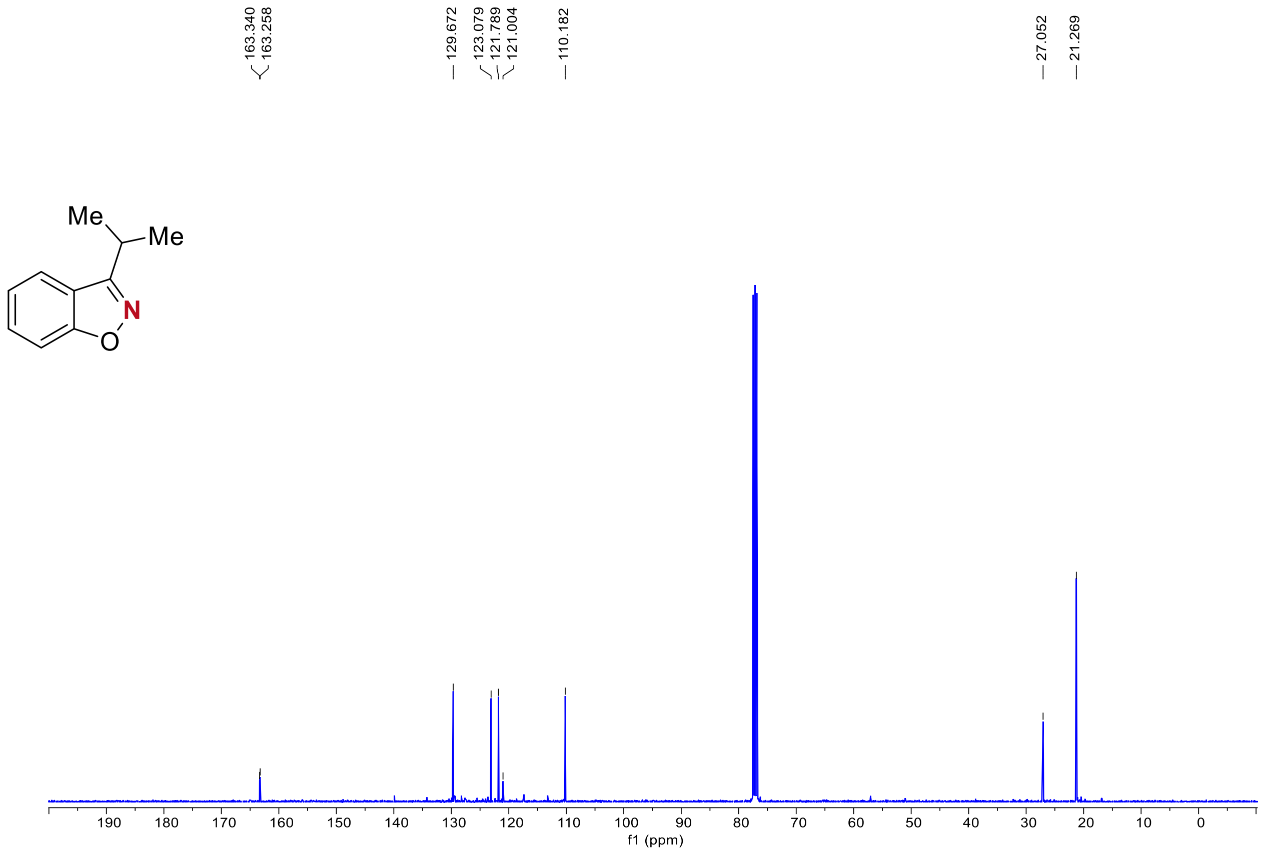

2-Isopropylbenzo[*d*]oxazole 6n

^{1}H NMR (300 MHz, $CDCl_3$)

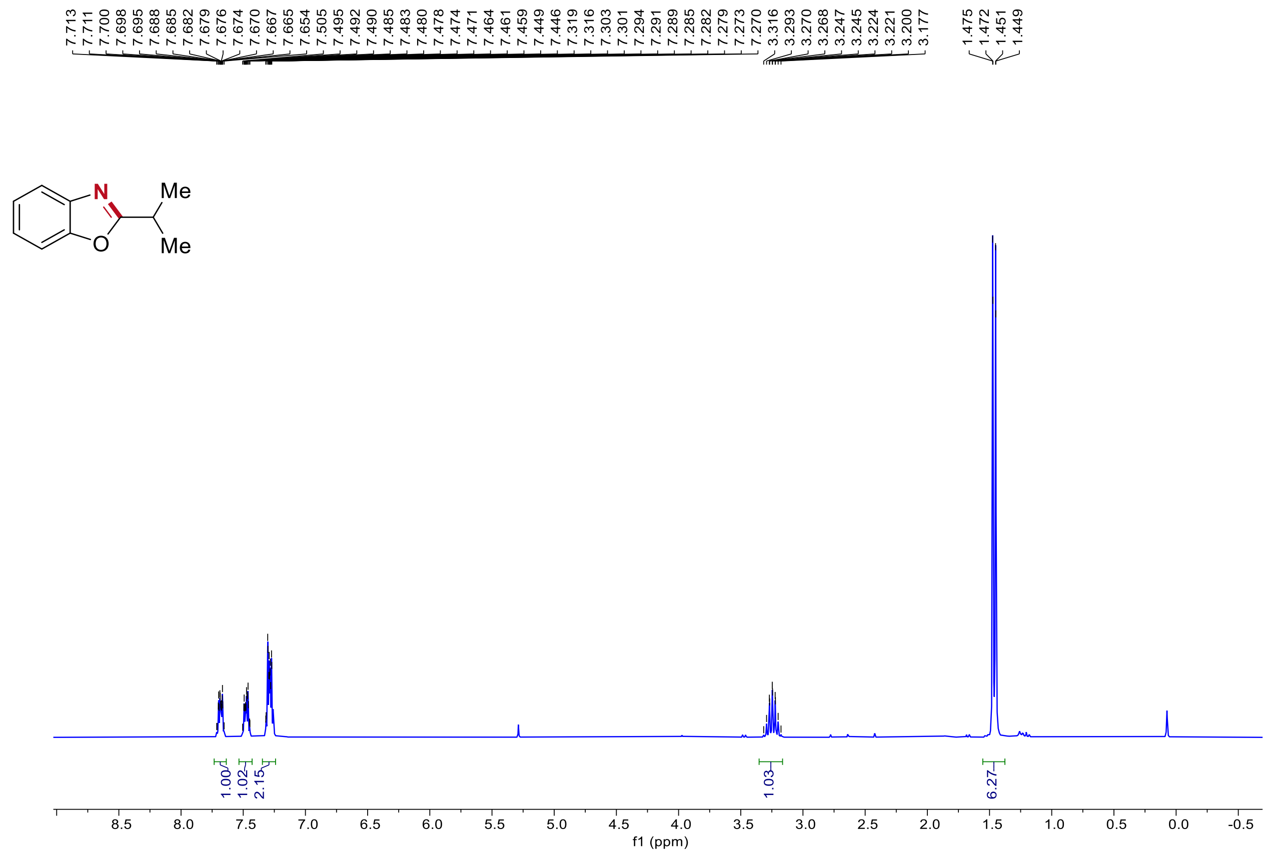

^{13}C NMR (76 MHz, $CDCl_3$)

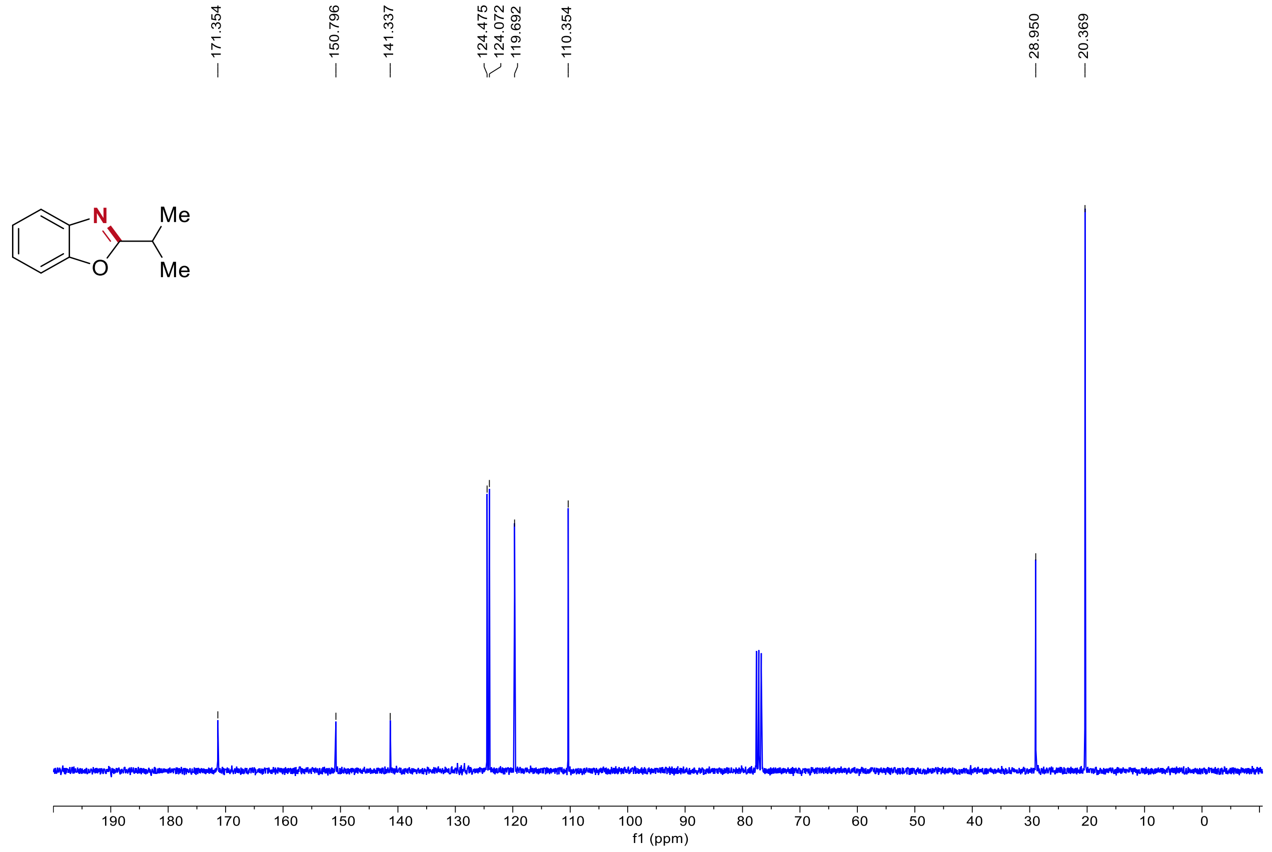

5-(Benzo[*d*]isoxazol-3-yl)pentanamide 5o

^{1}H NMR (400 MHz, $CDCl_3$)

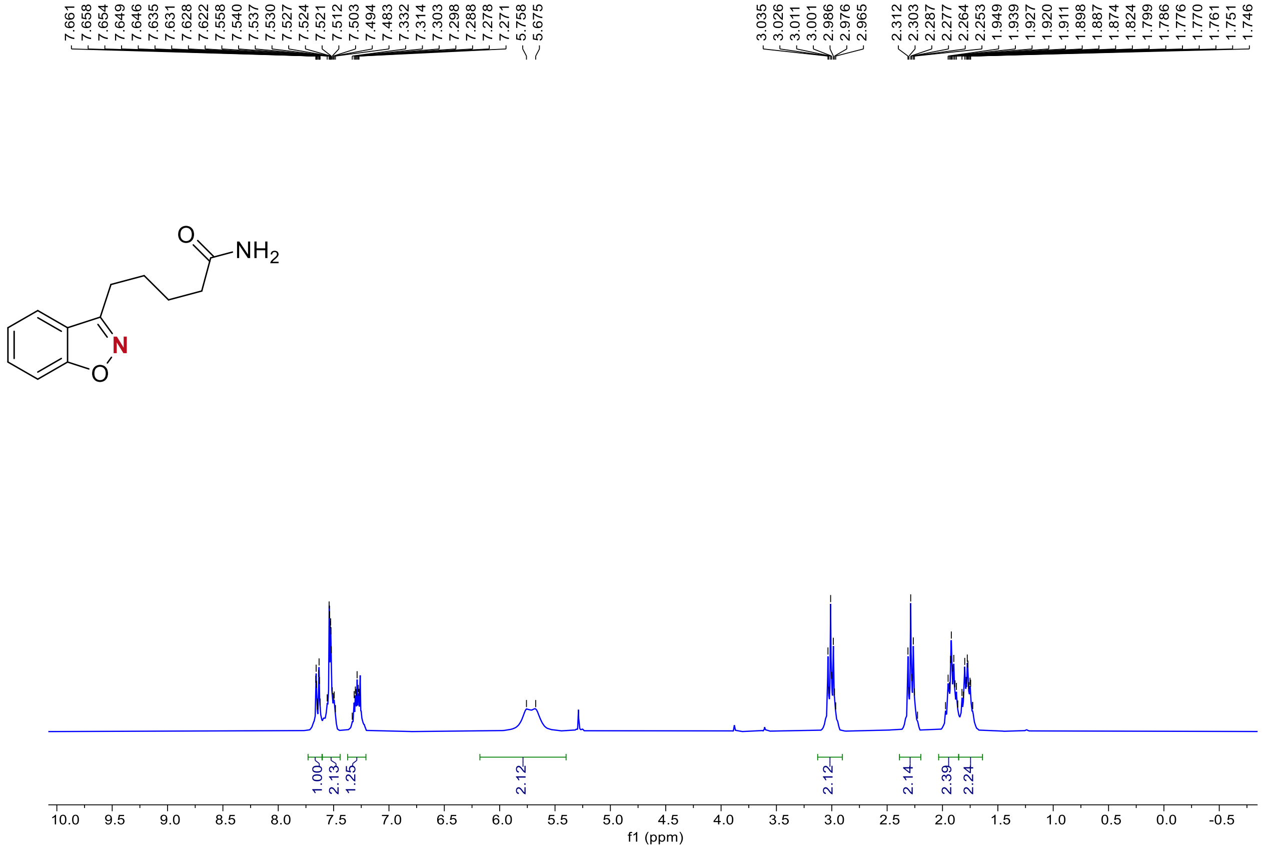

^{13}C NMR (101 MHz, $CDCl_3$)

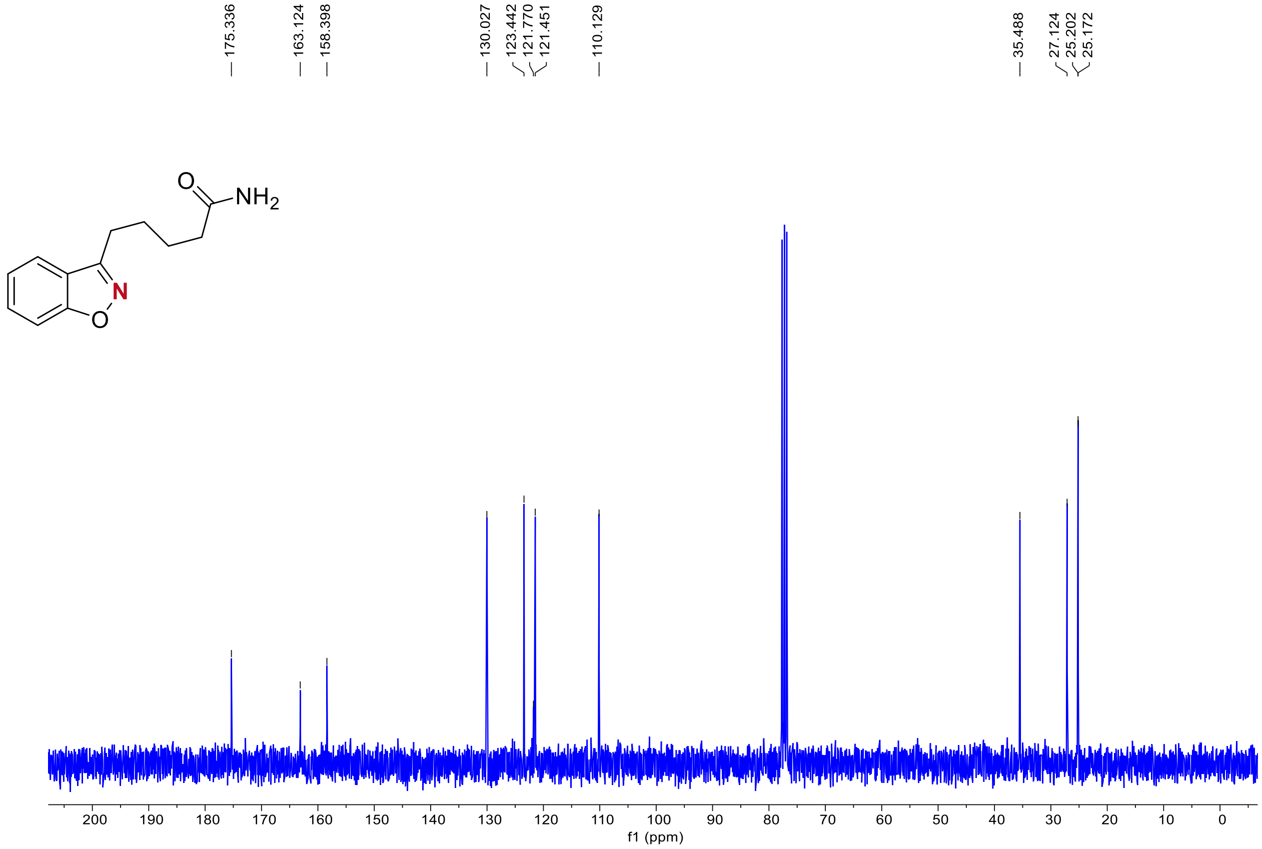

5-(Benzo[*d*]oxazol-2-yl)pentanamide 6o

^{1}H NMR (400 MHz, $CDCl_3$)

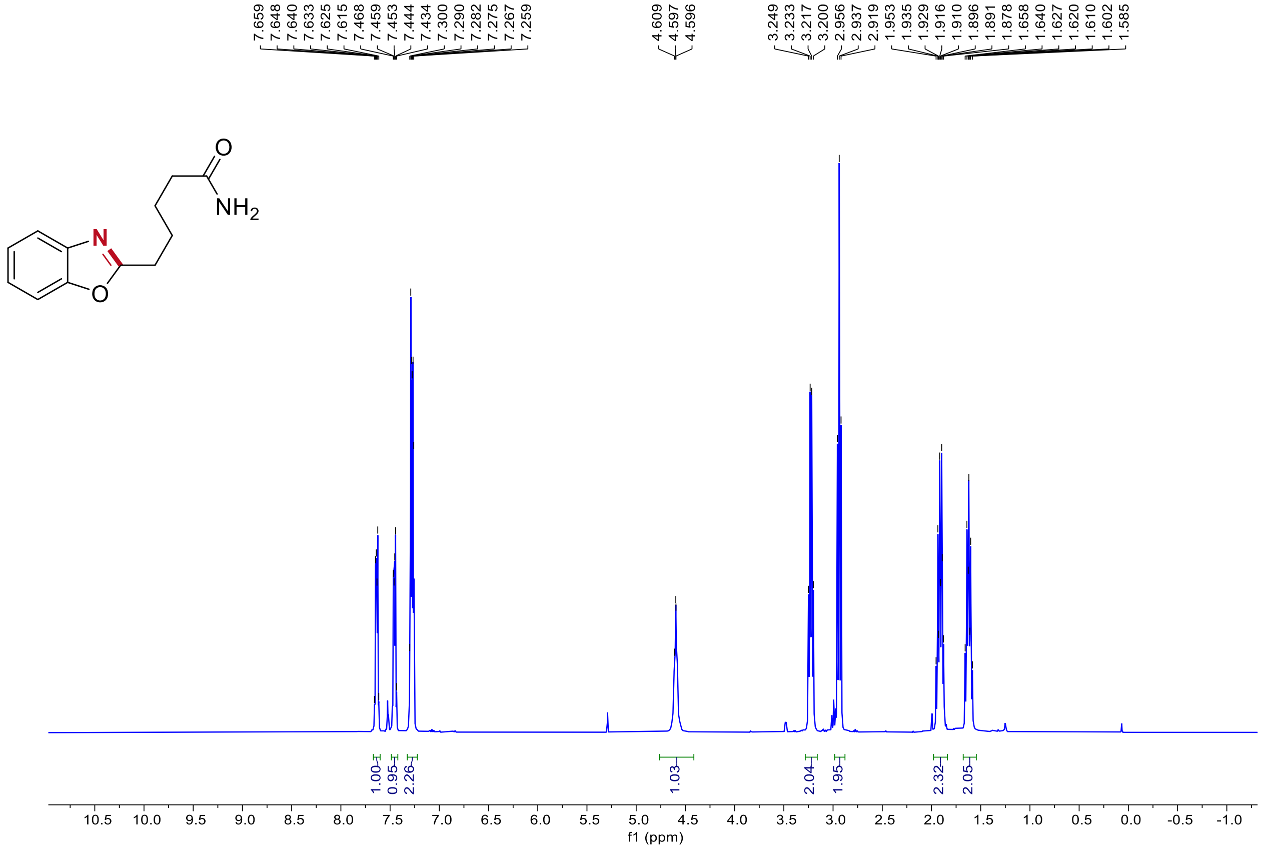

^{13}C NMR (101 MHz, $CDCl_3$)

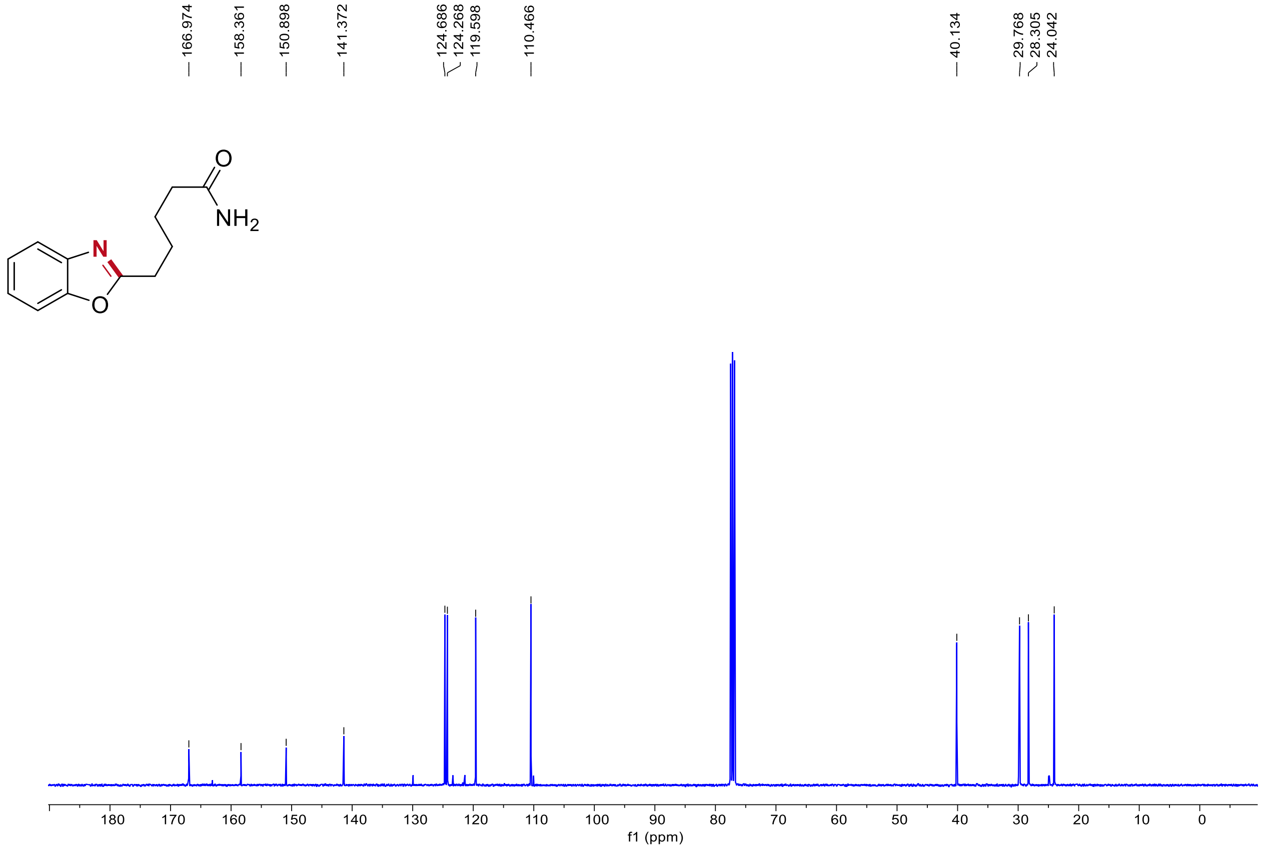

Methyl (*S*)-3-(3-((2-(6-methoxynaphthalen-2-yl)propanoyl)oxy)propyl)-1*H*-indazole-1-carboxylate (Moc-**2r**)

^{1}H NMR (400 MHz, $CDCl_3$)

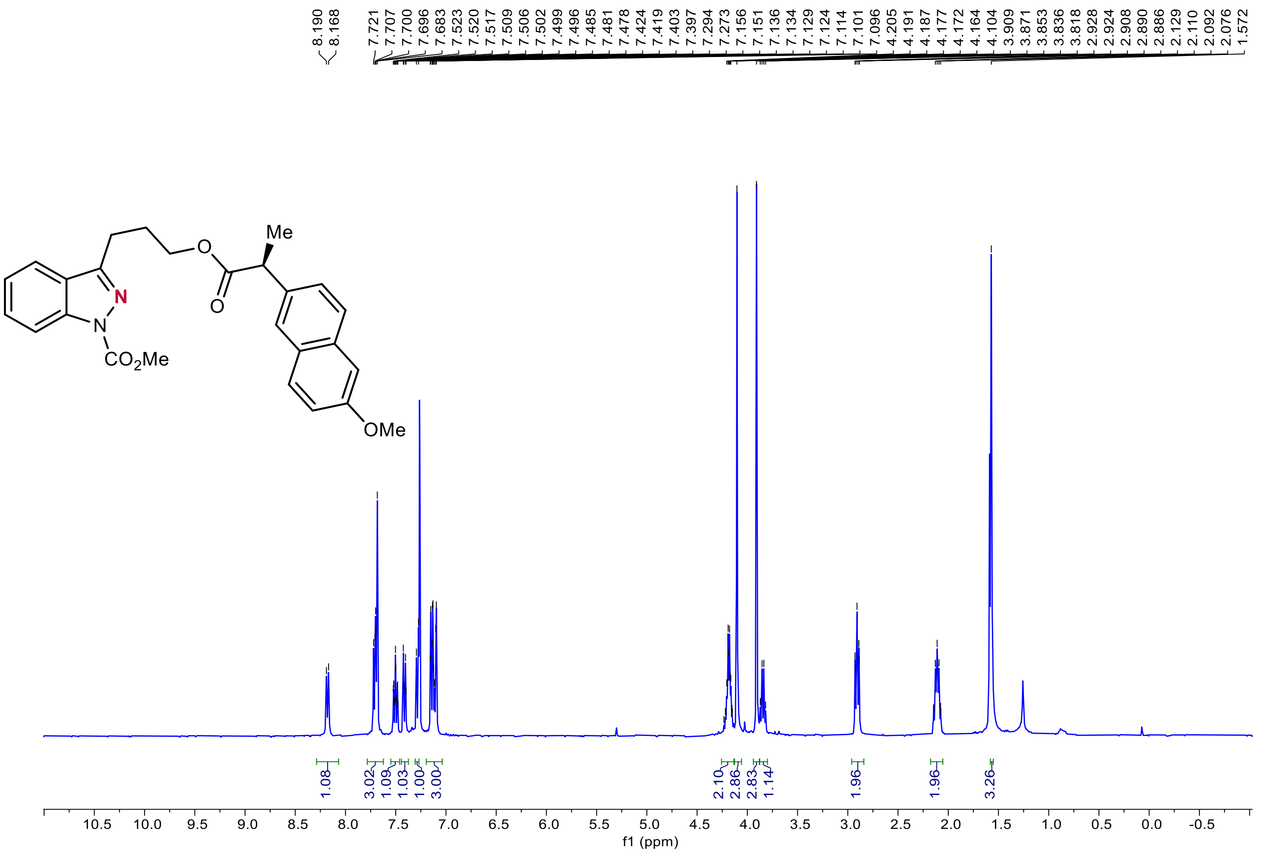

^{13}C NMR (76 MHz, $CDCl_3$)

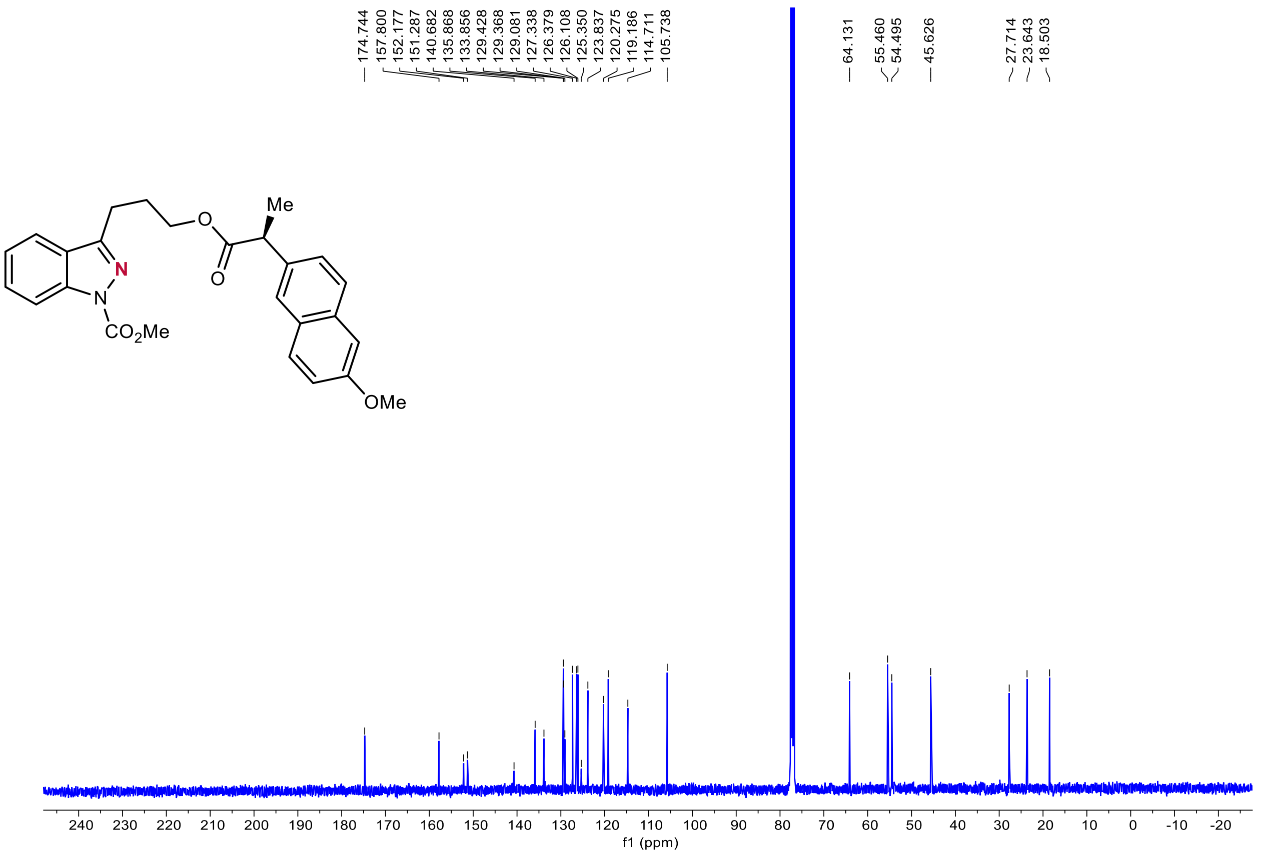

methyl (*S*)-2-(3-((2-(6-methoxynaphthalen-2-yl)propanoyl)oxy)propyl)-1*H*-benzo[d]imidazole-1-carboxylate (Moc-3r) ^{1}**H NMR** (599 MHz, CDCl$_3$)

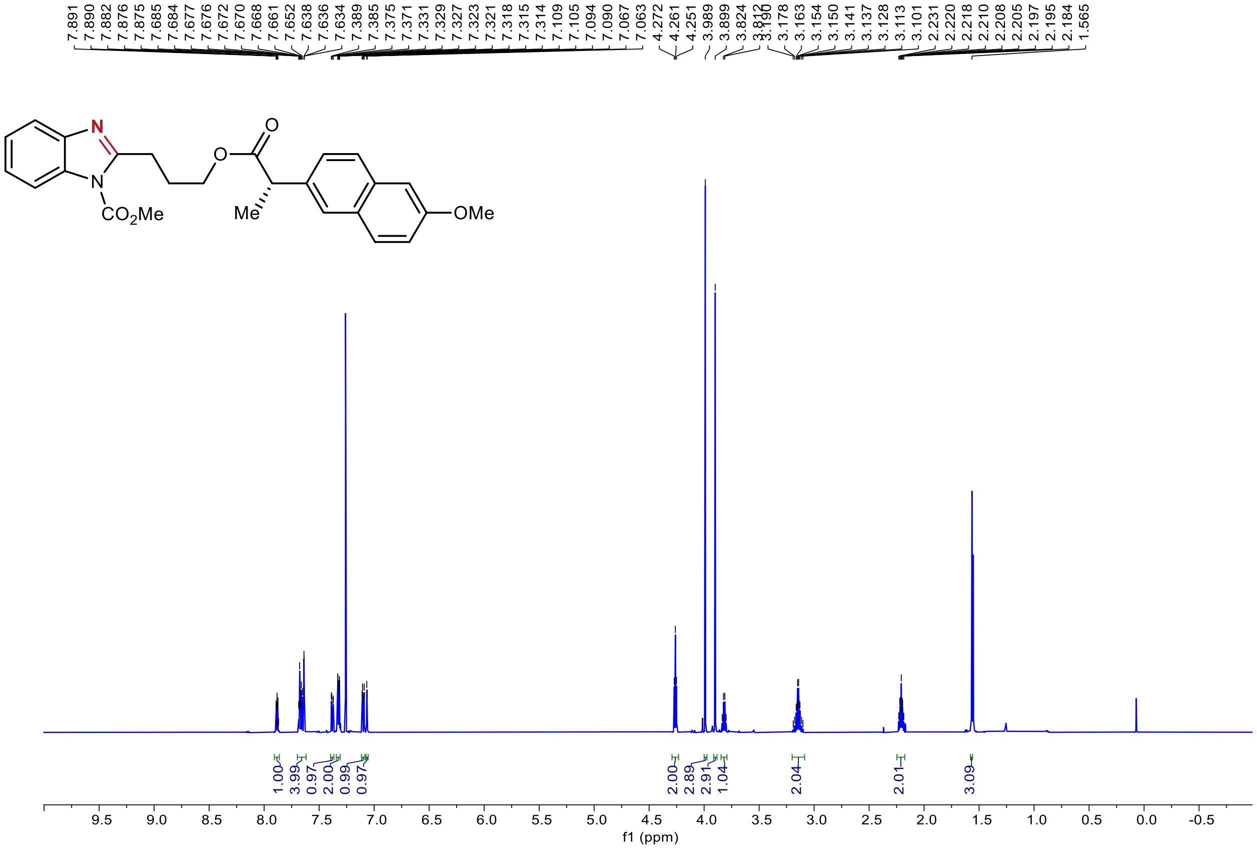

^{13}**C NMR** (151 MHz, CDCl$_3$)

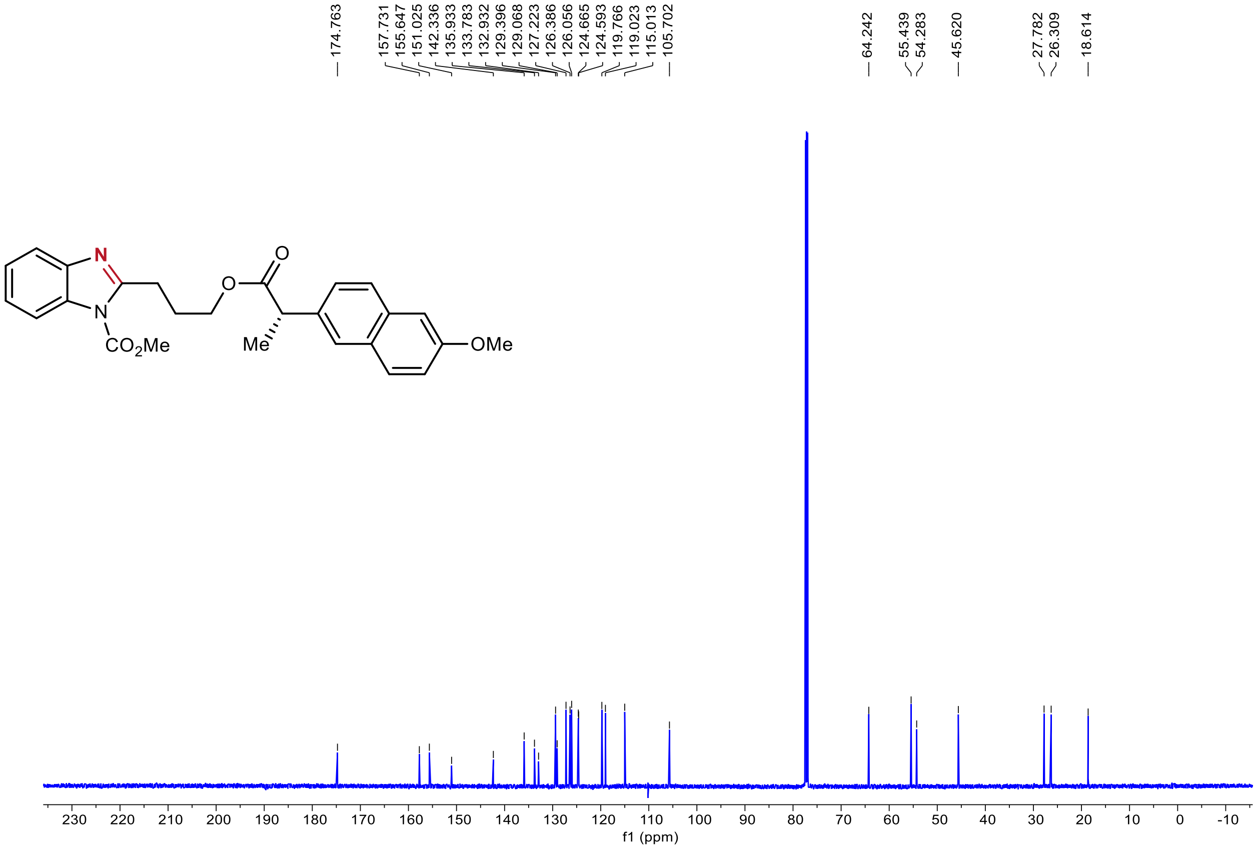

3-(1*H*-indazol-3-yl)propyl (*S*)-2-(6-methoxynaphthalen-2-yl)propanoate 2r

^{1}H NMR (400 MHz, $CDCl_3$)

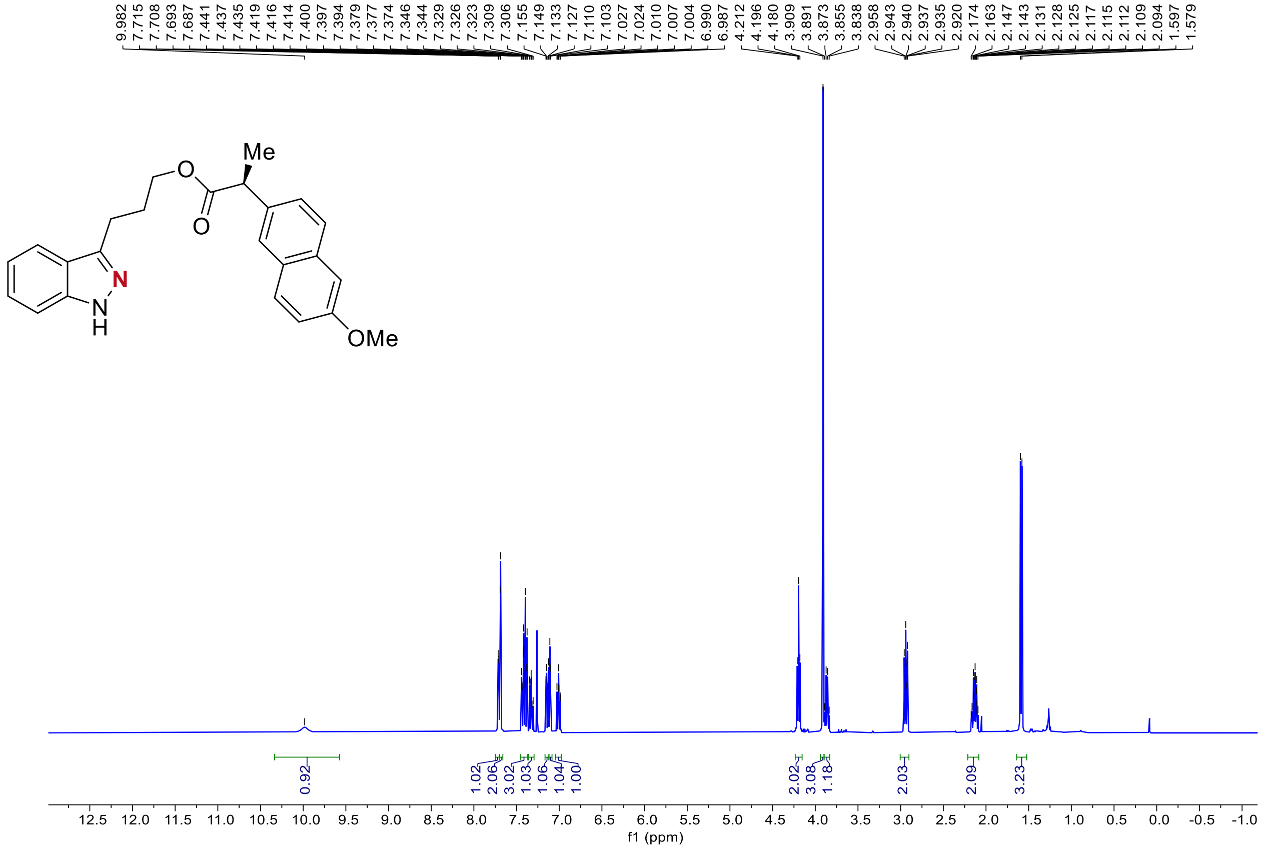

^{13}C NMR (101 MHz, $CDCl_3$)

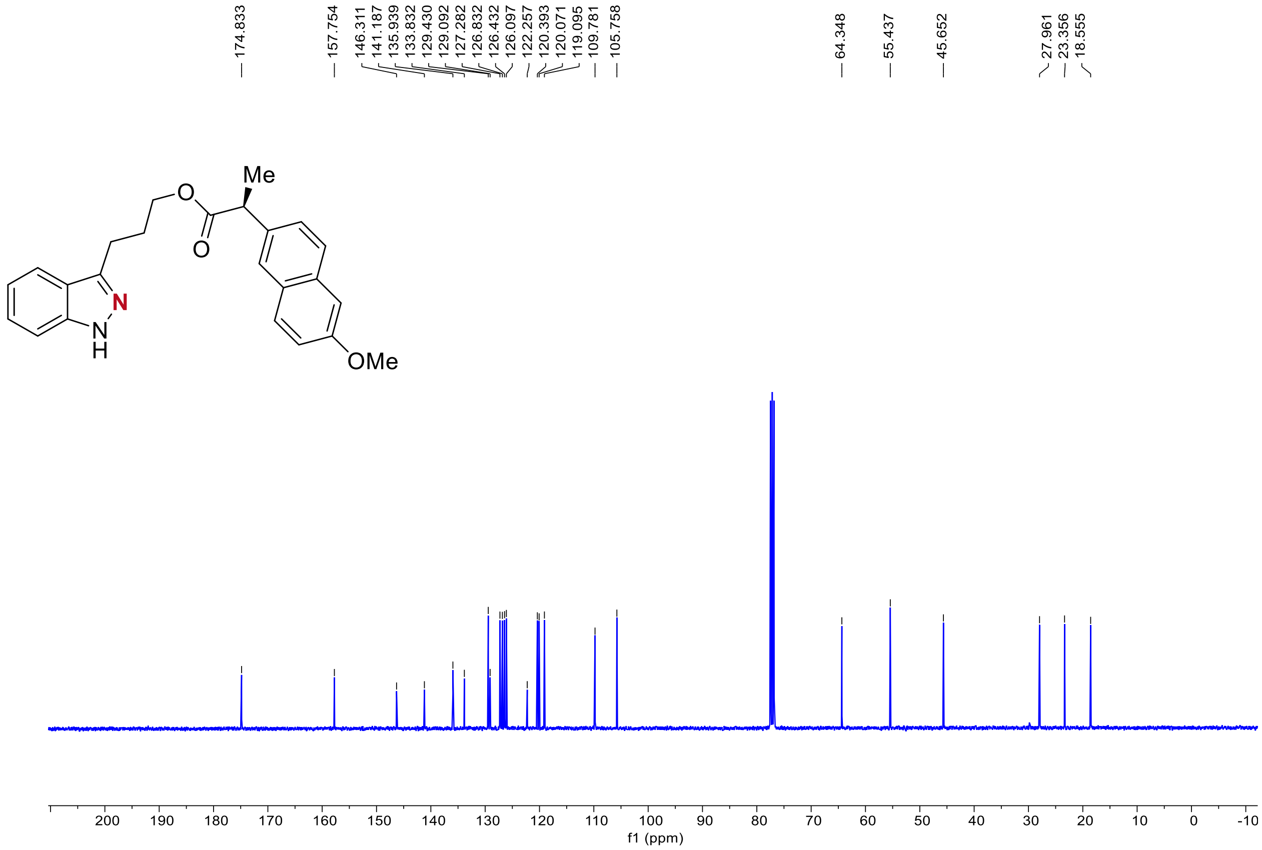

3-(1-Tosyl-1*H*-indazol-3-yl)propyl (*S*)-2-(6-methoxynaphthalen-2-yl)propanoate Ts-2r

^{1}H NMR (400 MHz, $CDCl_3$)

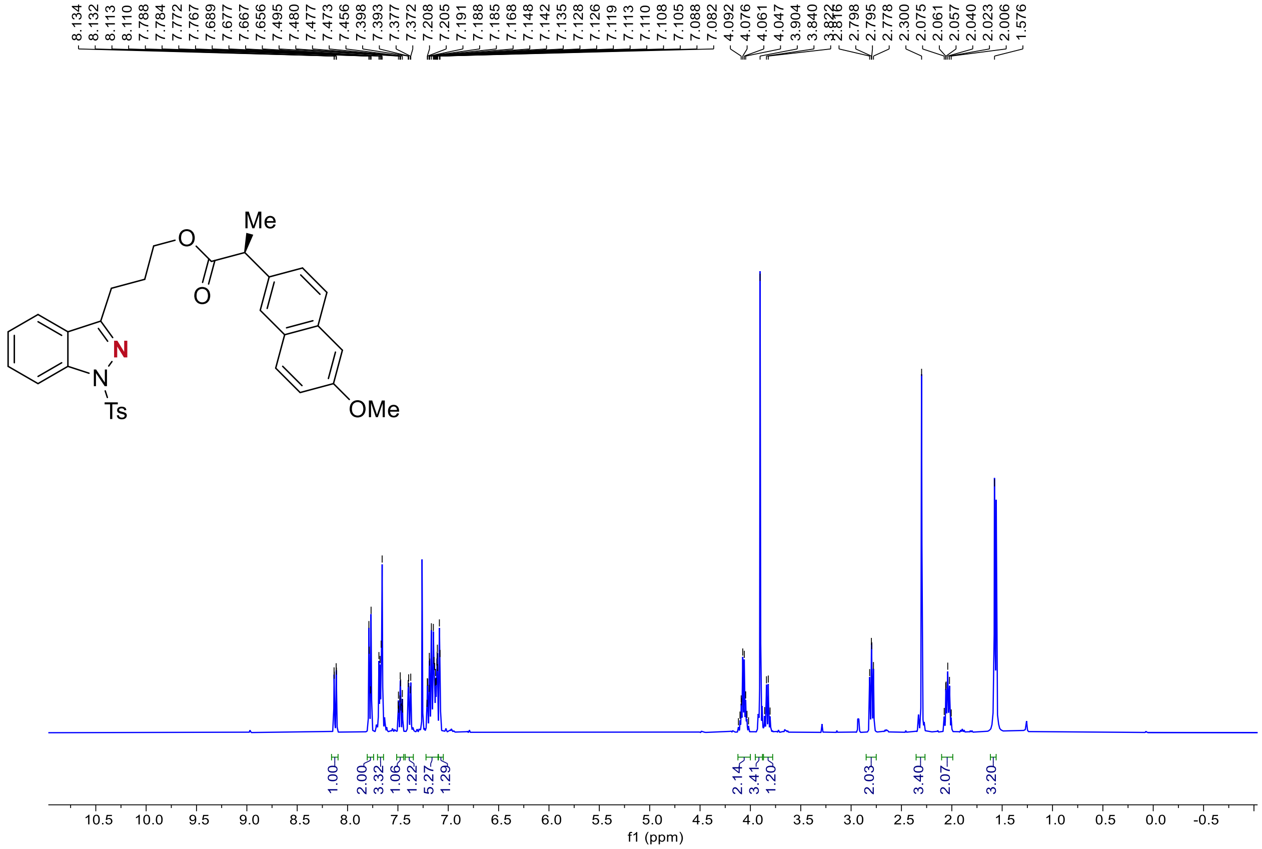

^{13}C NMR (101 MHz, $CDCl_3$)

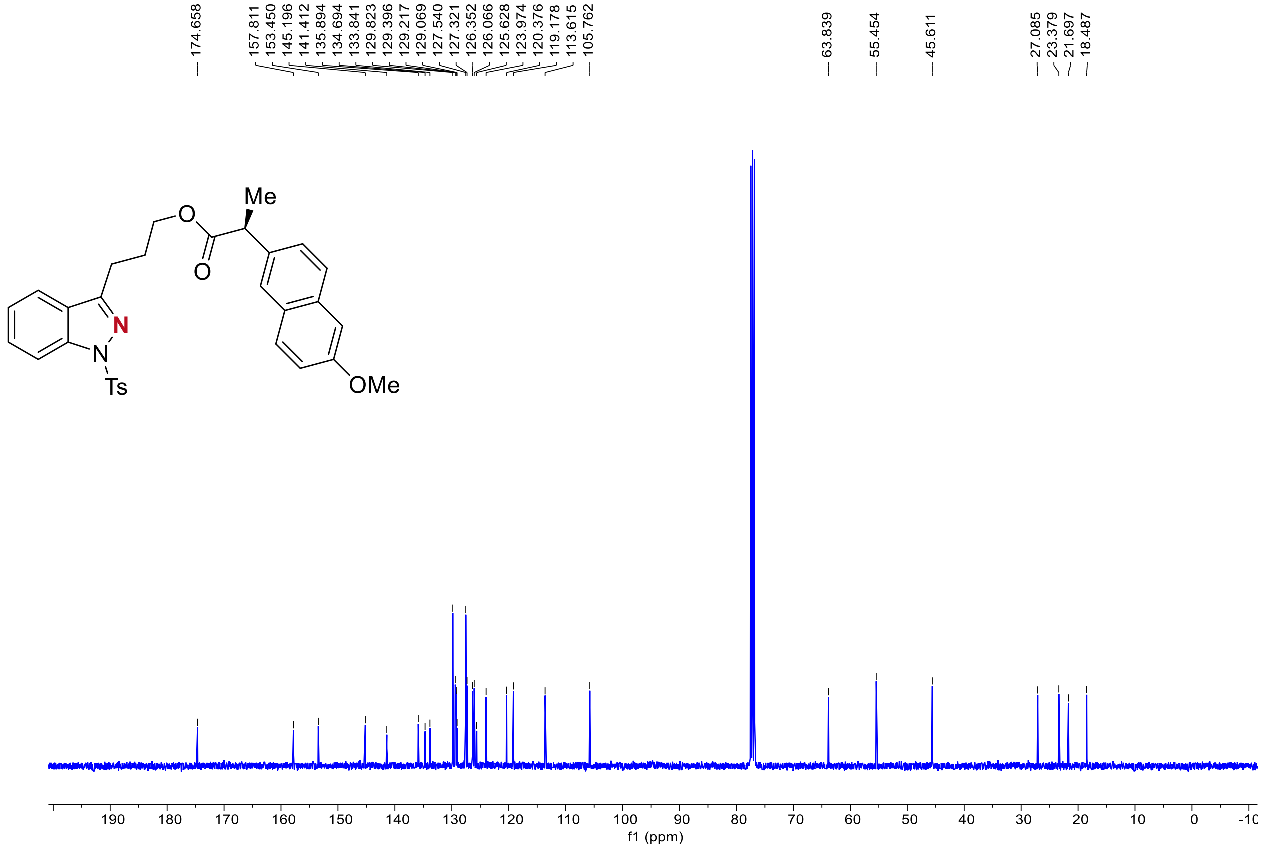

3-(1-Tosyl-1*H*-benzo[d]imidazol-2-yl)propyl (*S*)-2-(6-methoxynaphthalen-2-yl)propanoate Ts-3r

^{1}H NMR (400 MHz, $CDCl_3$)

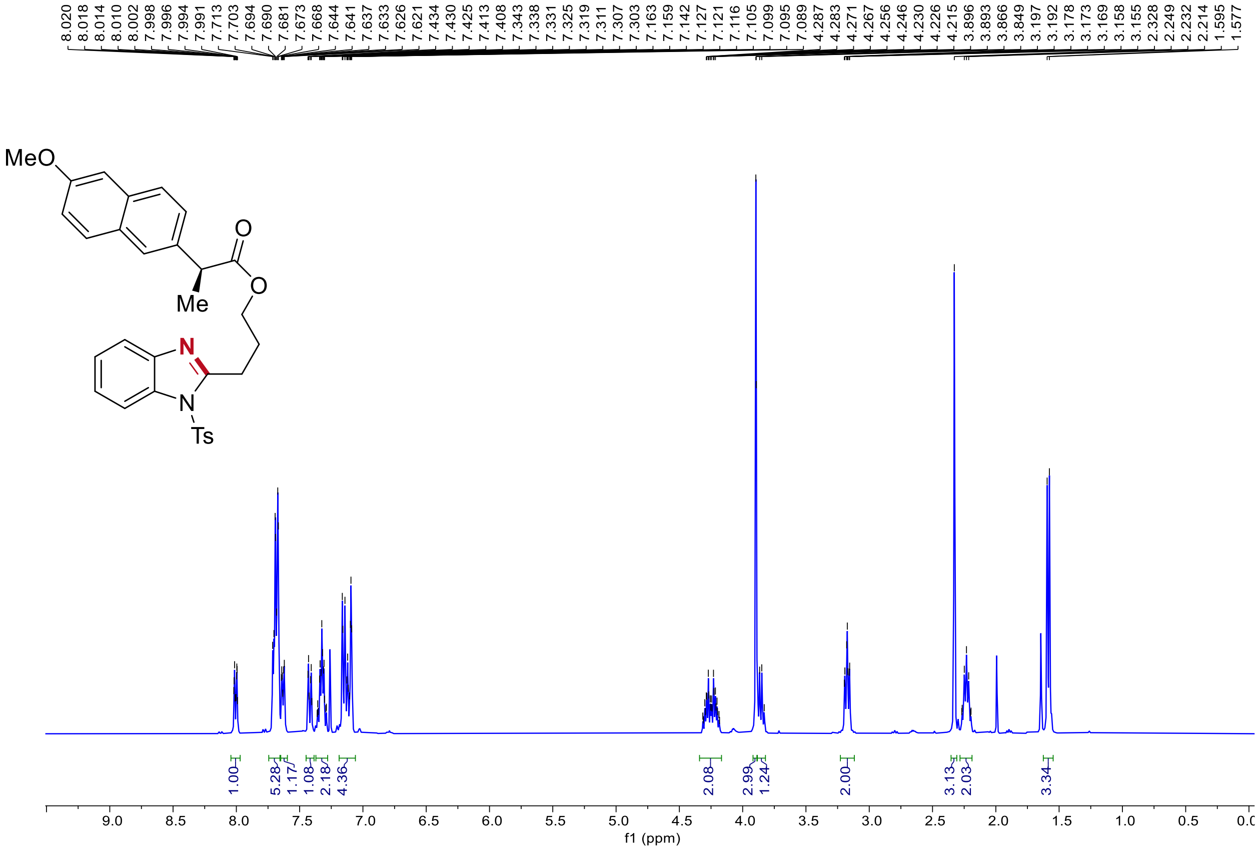

^{13}C NMR (101 MHz, $CDCl_3$)

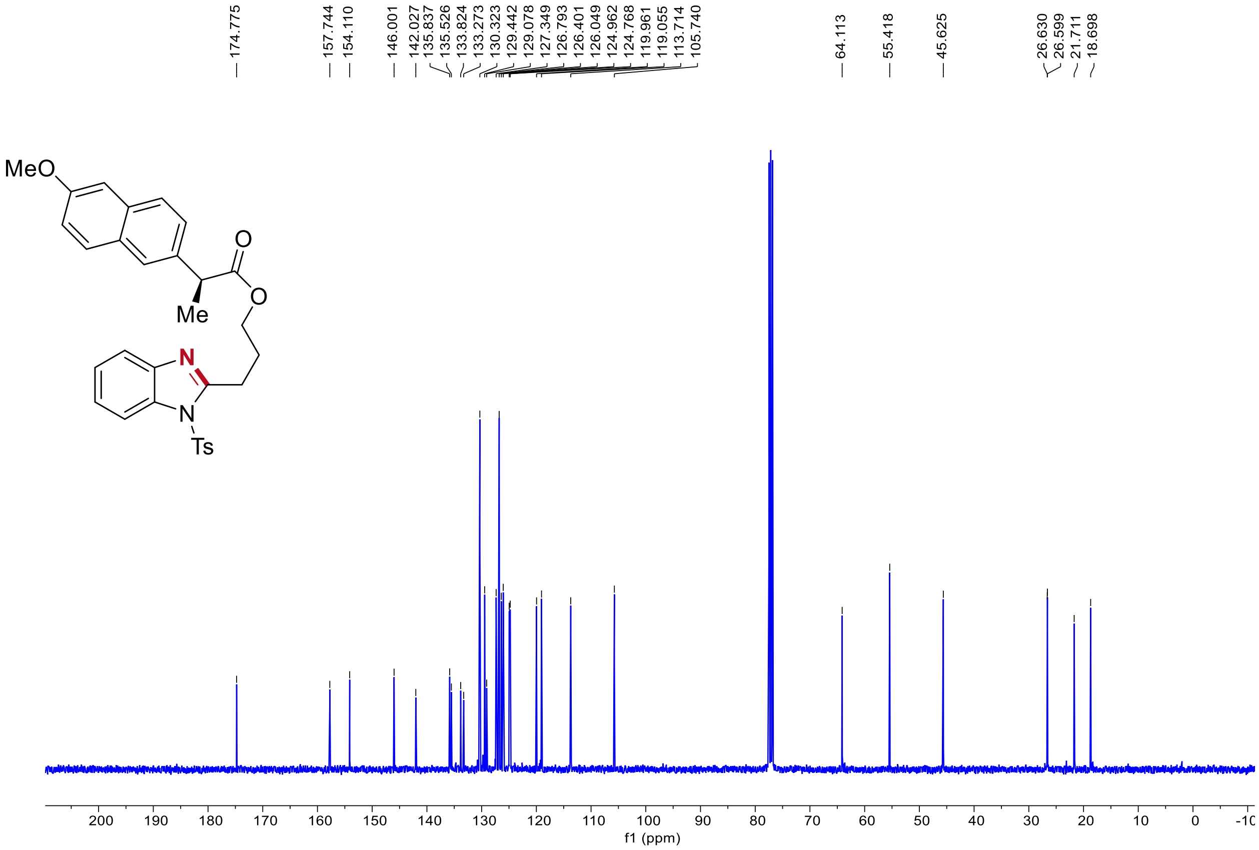

Methyl 3-(3-methoxy-3-oxopropyl)-1H-indazole-1-carboxylate Moc-**2s**

^{1}H NMR (300 MHz, $CDCl_3$)

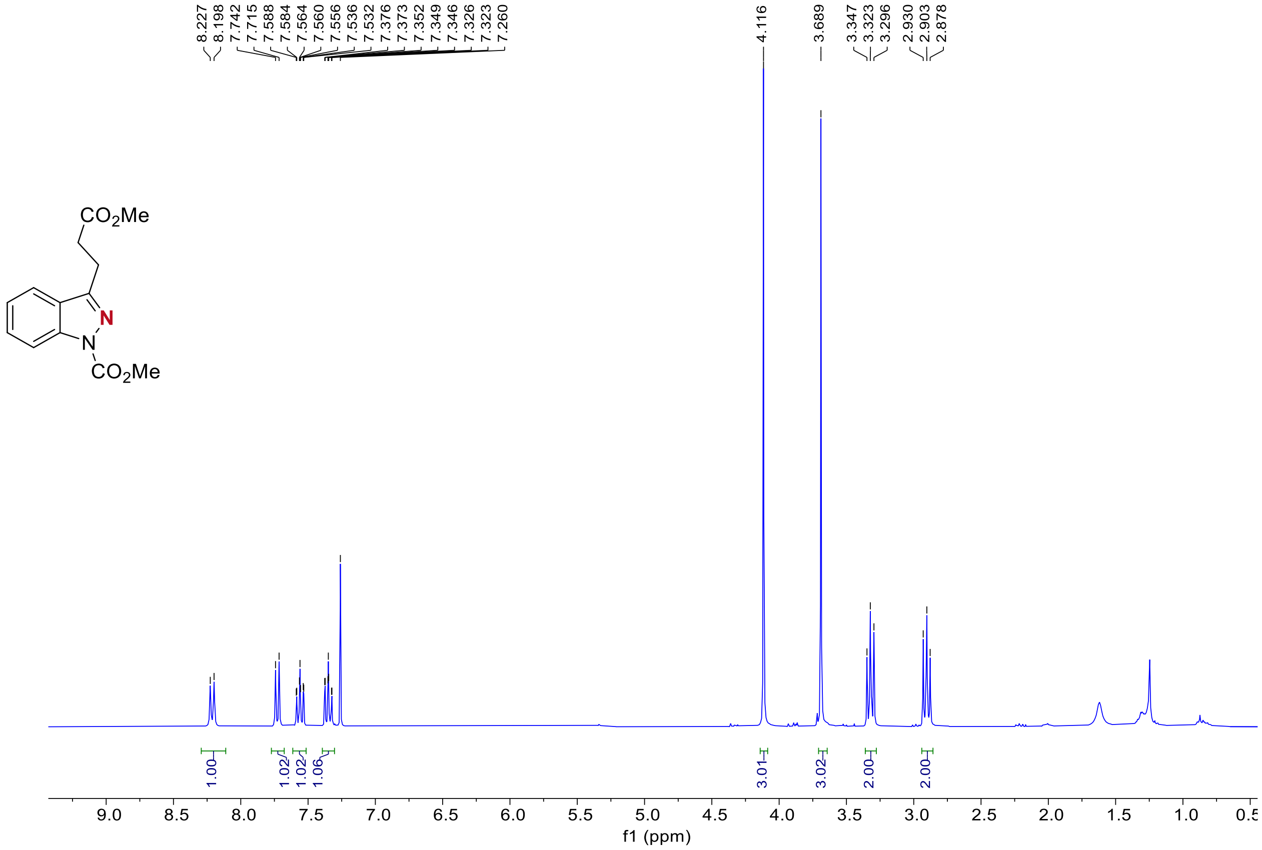

^{13}C NMR (100 MHz, $CDCl_3$)

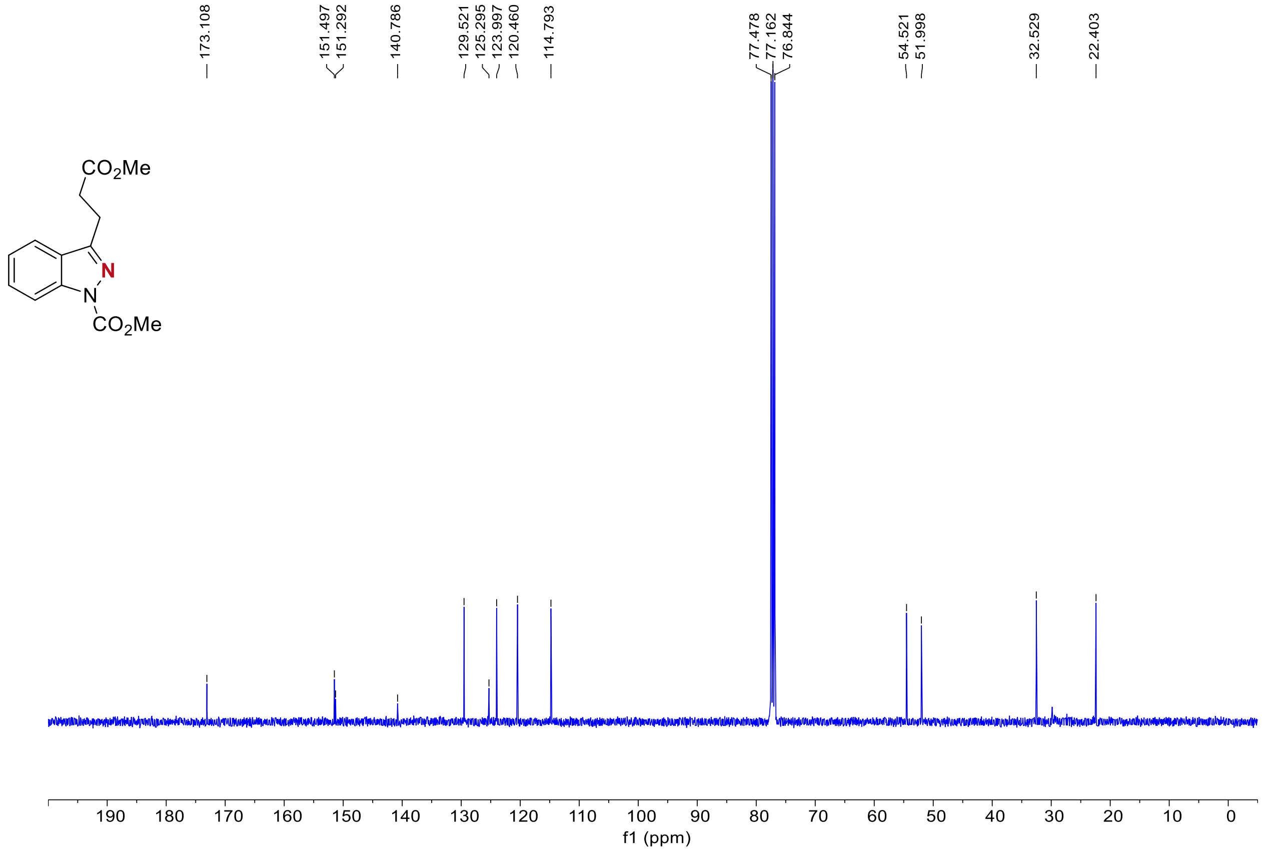

Methyl 2-(3-methoxy-3-oxopropyl)-1*H*-benzo[d]imidazole-1-carboxylate Moc-**3s**

^{1}H NMR (300 MHz, $CDCl_3$)

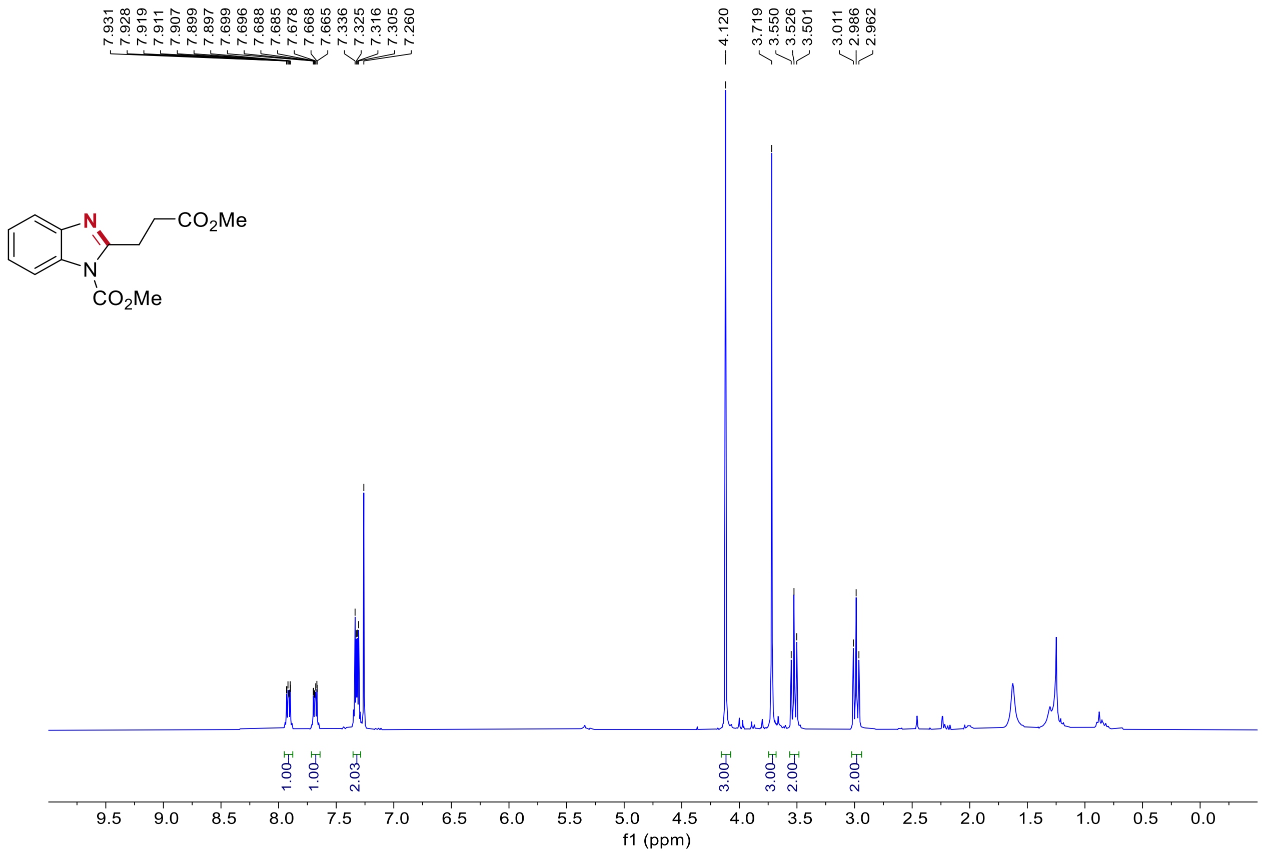

^{13}C NMR (100 MHz, $CDCl_3$)

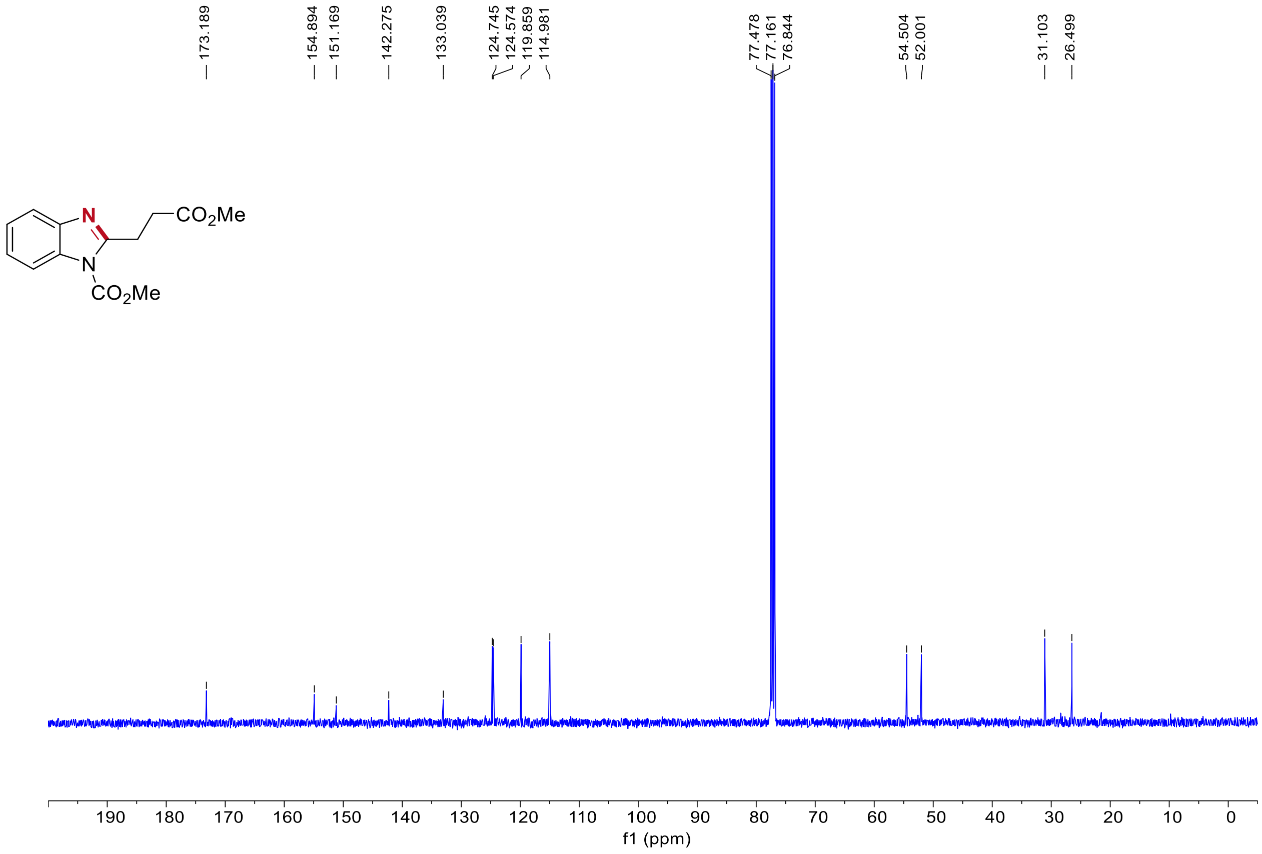

Methyl 3-(1*H*-indazol-3-yl)propanoate 2s

^{1}**H NMR** (400 MHz, $CDCl_3$)

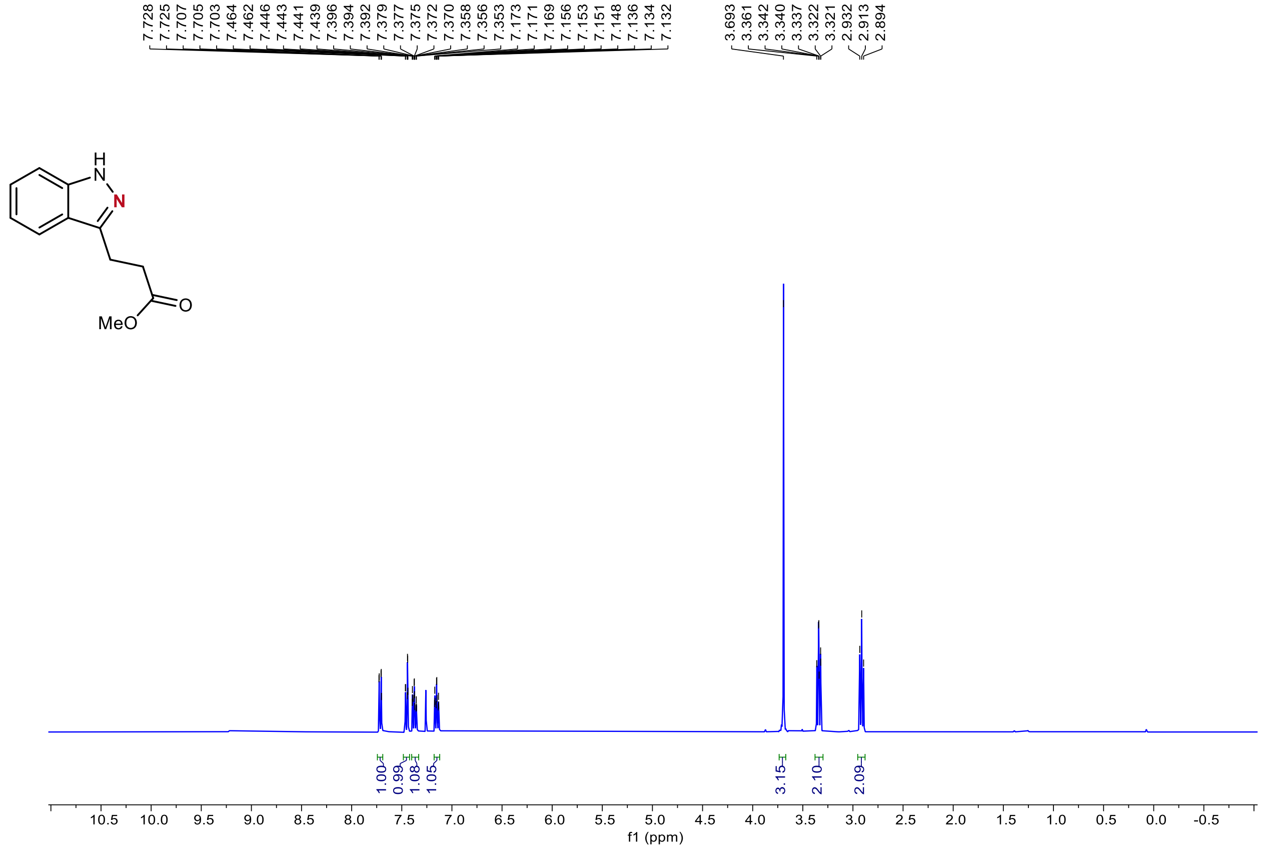

^{13}**C NMR** (76 MHz, $CDCl_3$)

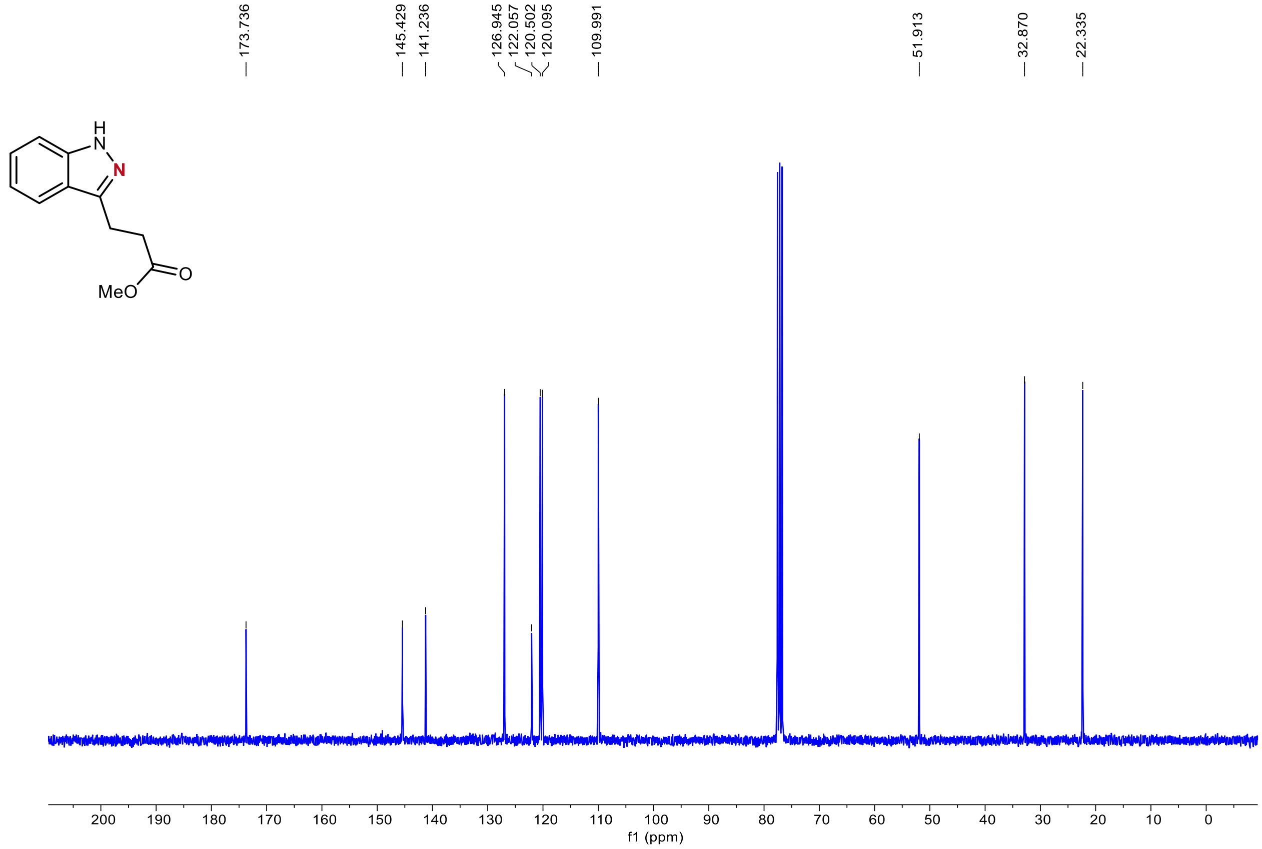

Methyl 3-(1-tosyl-1H-benzo[*d*]imidazol-2-yl)propanoate Ts-3s

^{1}H NMR (300 MHz, $CDCl_3$)

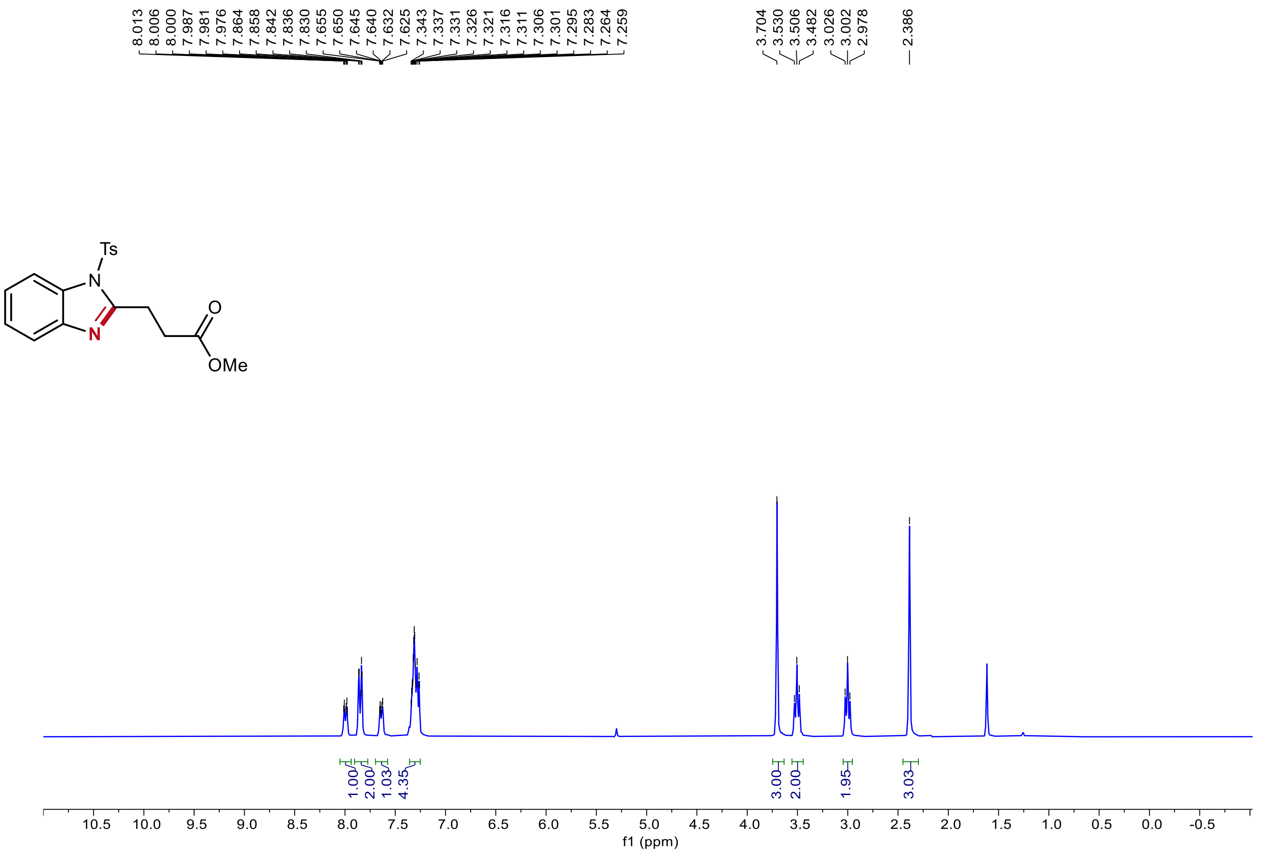

^{13}C NMR (76 MHz, $CDCl_3$)

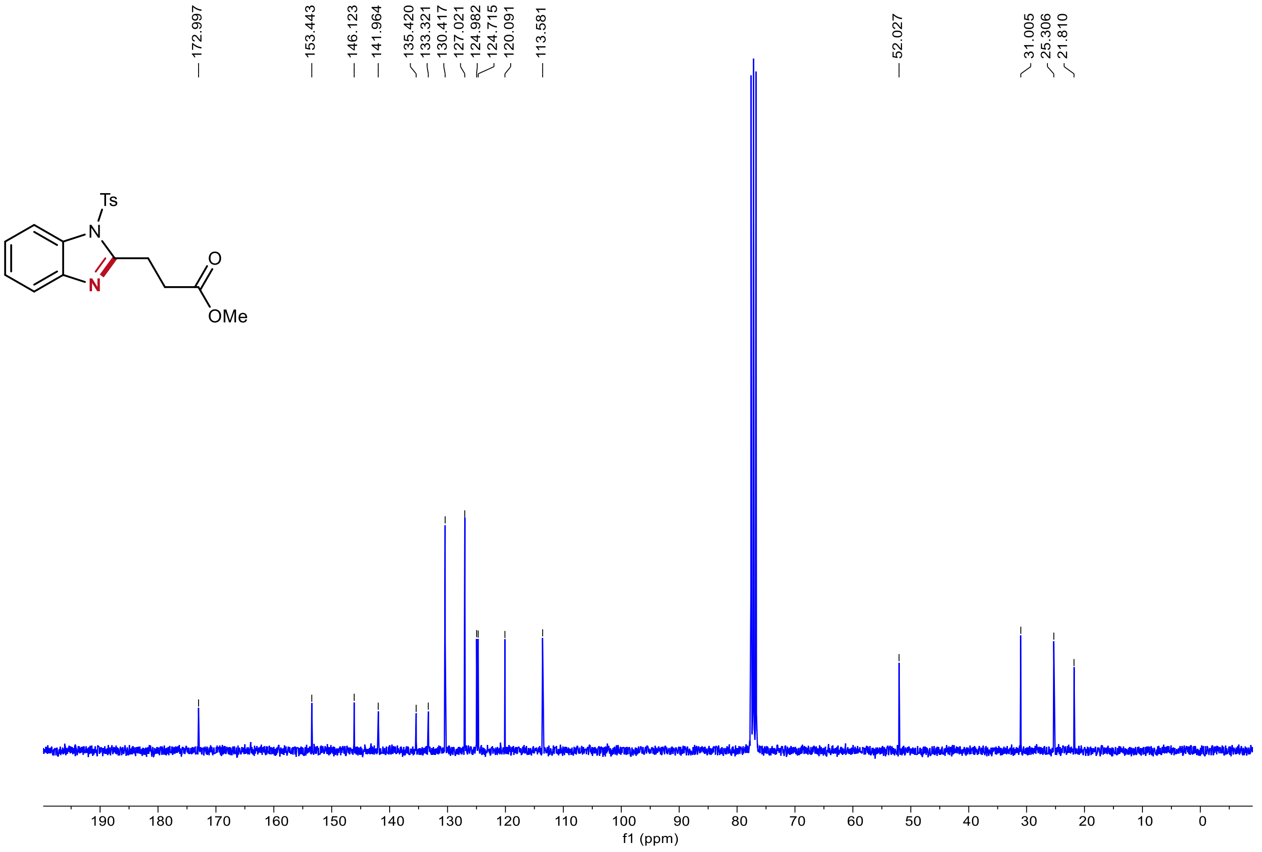

Methyl 3-(1-tosyl-1*H*-indazol-3-yl)propanoate Ts-2s

^{1}H NMR (300 MHz, $CDCl_3$)

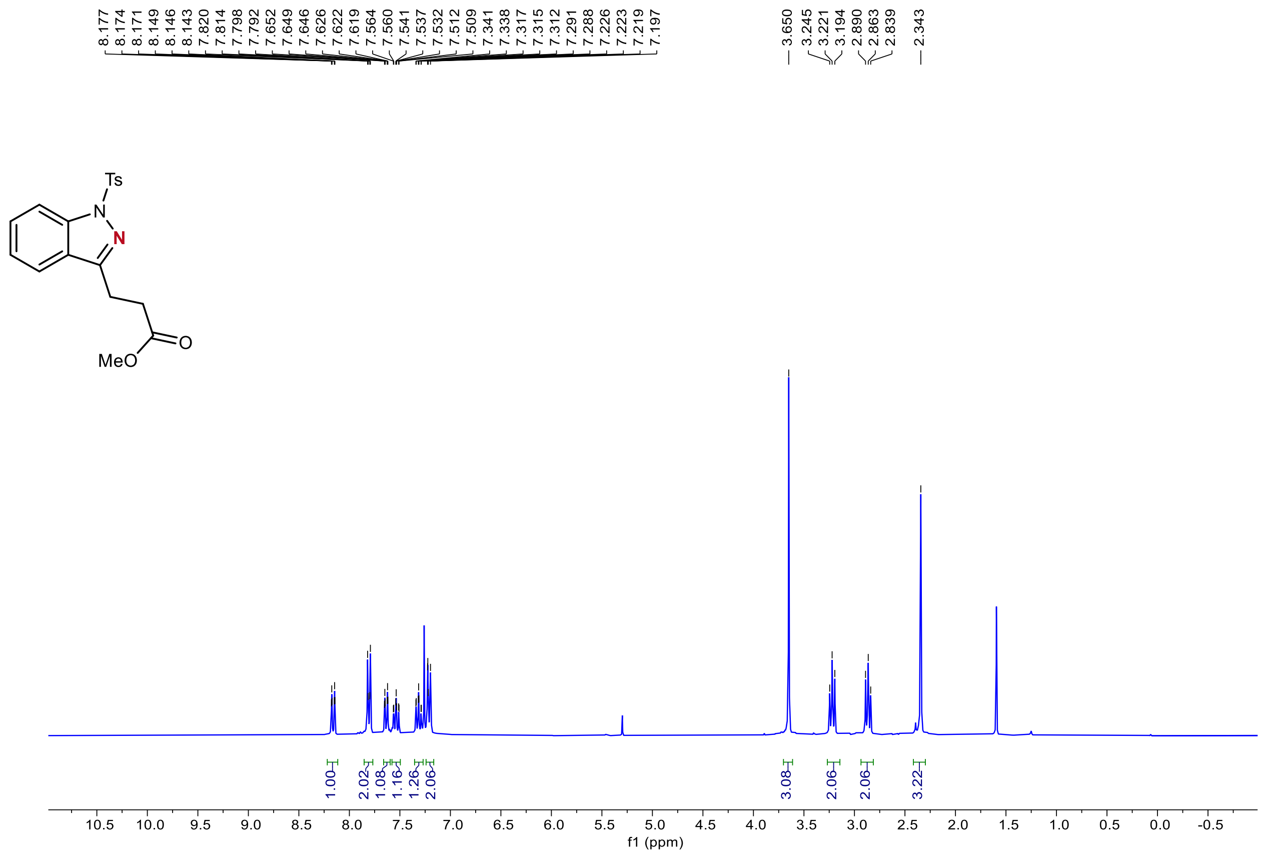

^{13}C NMR (76 MHz, $CDCl_3$)

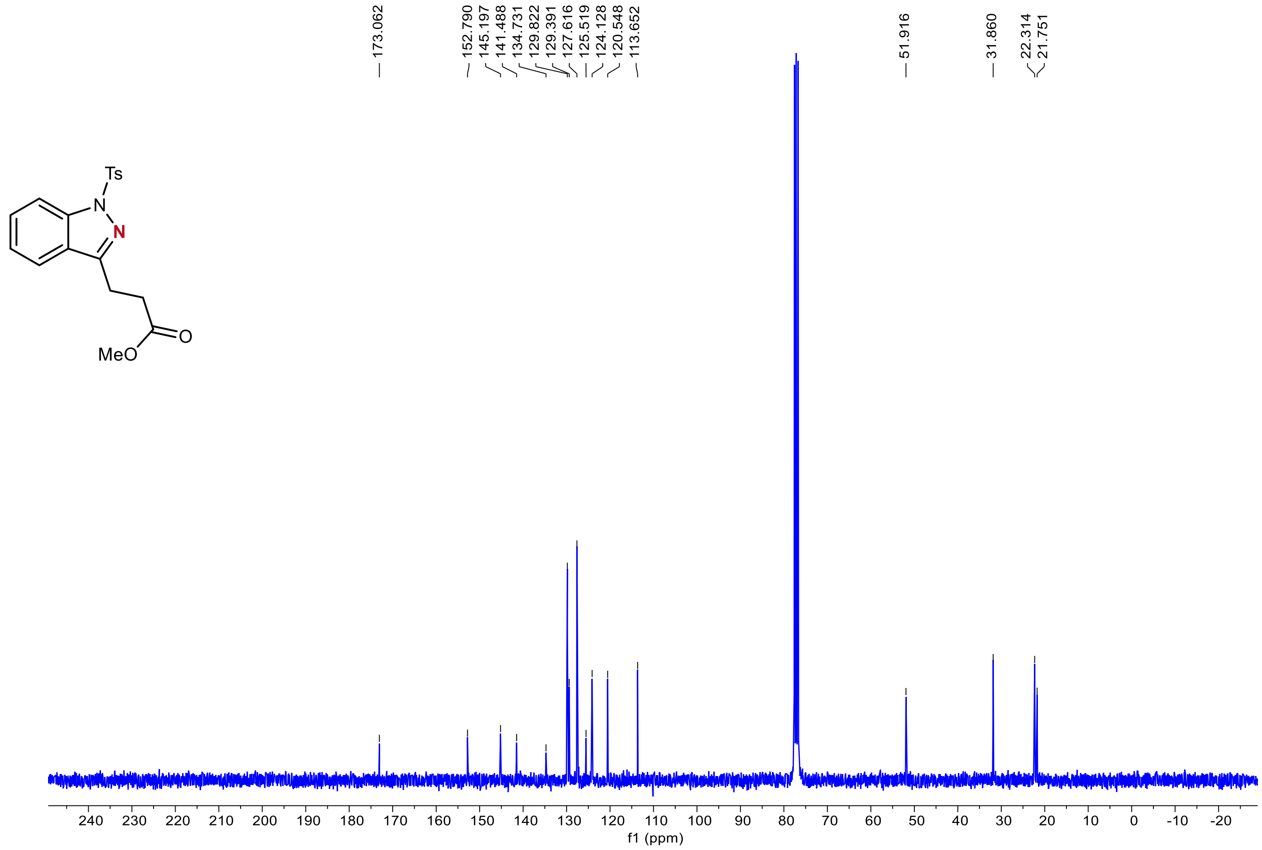

3-(1-(methoxycarbonyl)-1H-indazol-3-yl)propyl 2-(3-cyano-4-isobutoxyphenyl)-4-methylthiazole-5-carboxylate Moc-**2t** **^{1}H NMR** (400 MHz, $CDCl_3$)

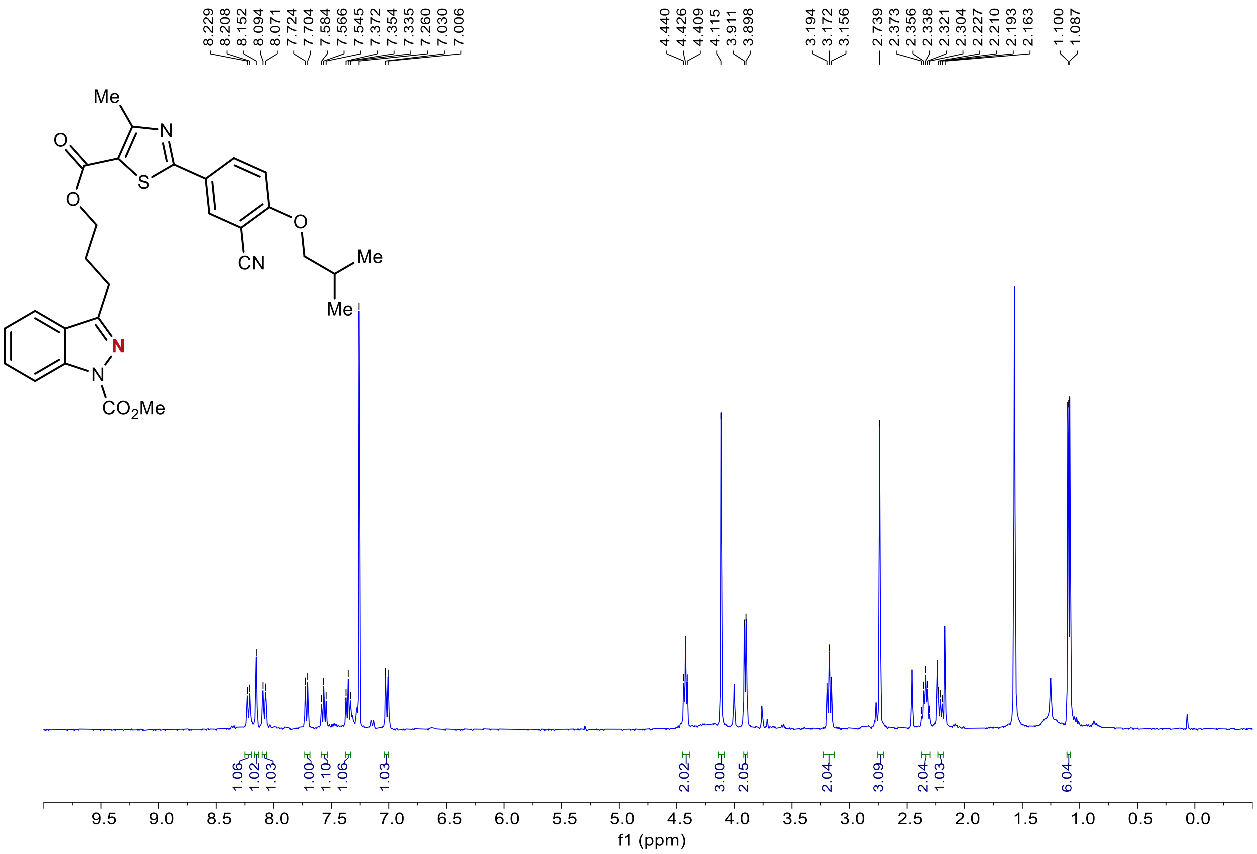

^{13}C NMR (125 MHz, $CDCl_3$)

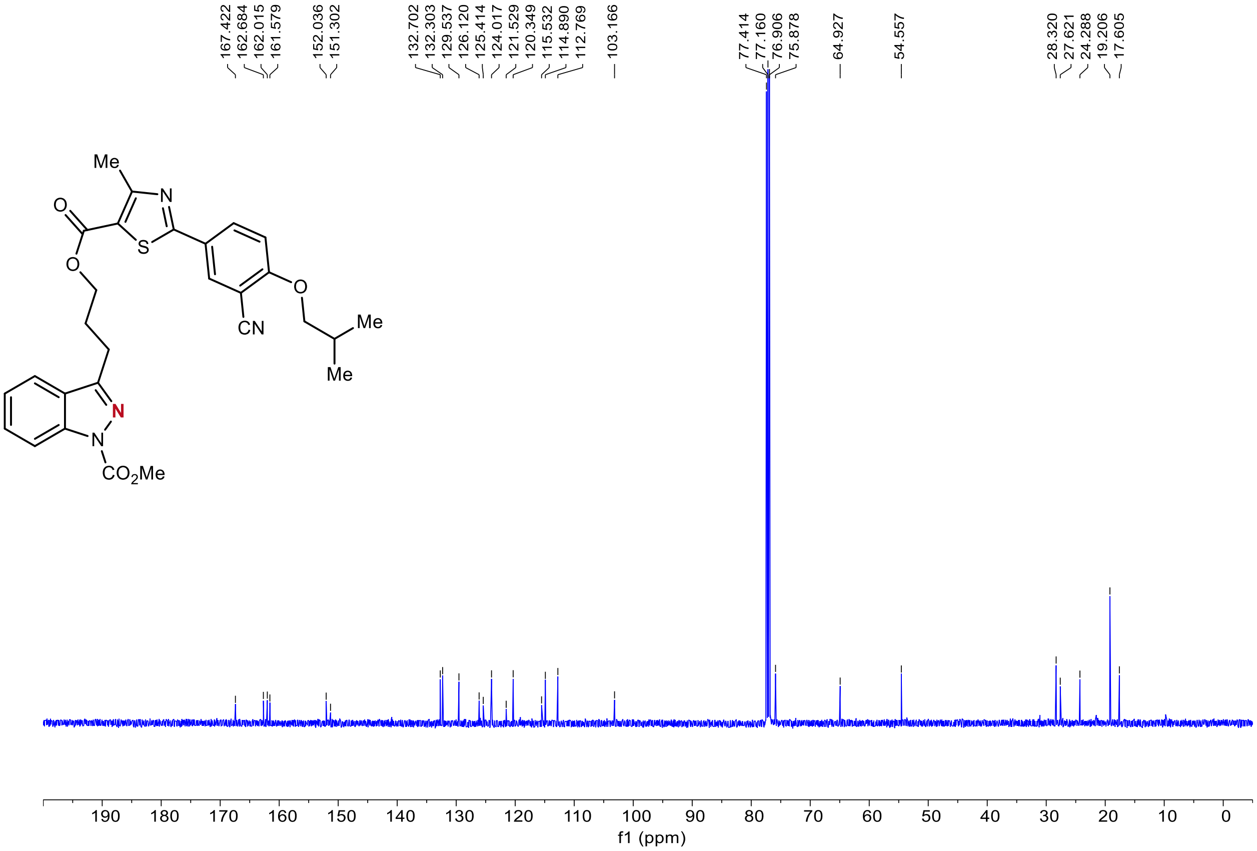

3-(1*H*-Indazol-3-yl)propyl 2-(3-cyano-4-isobutoxyphenyl)-4-methylthiazole-5-carboxylate 2t

^{1}H NMR (400 MHz, $CDCl_3$)

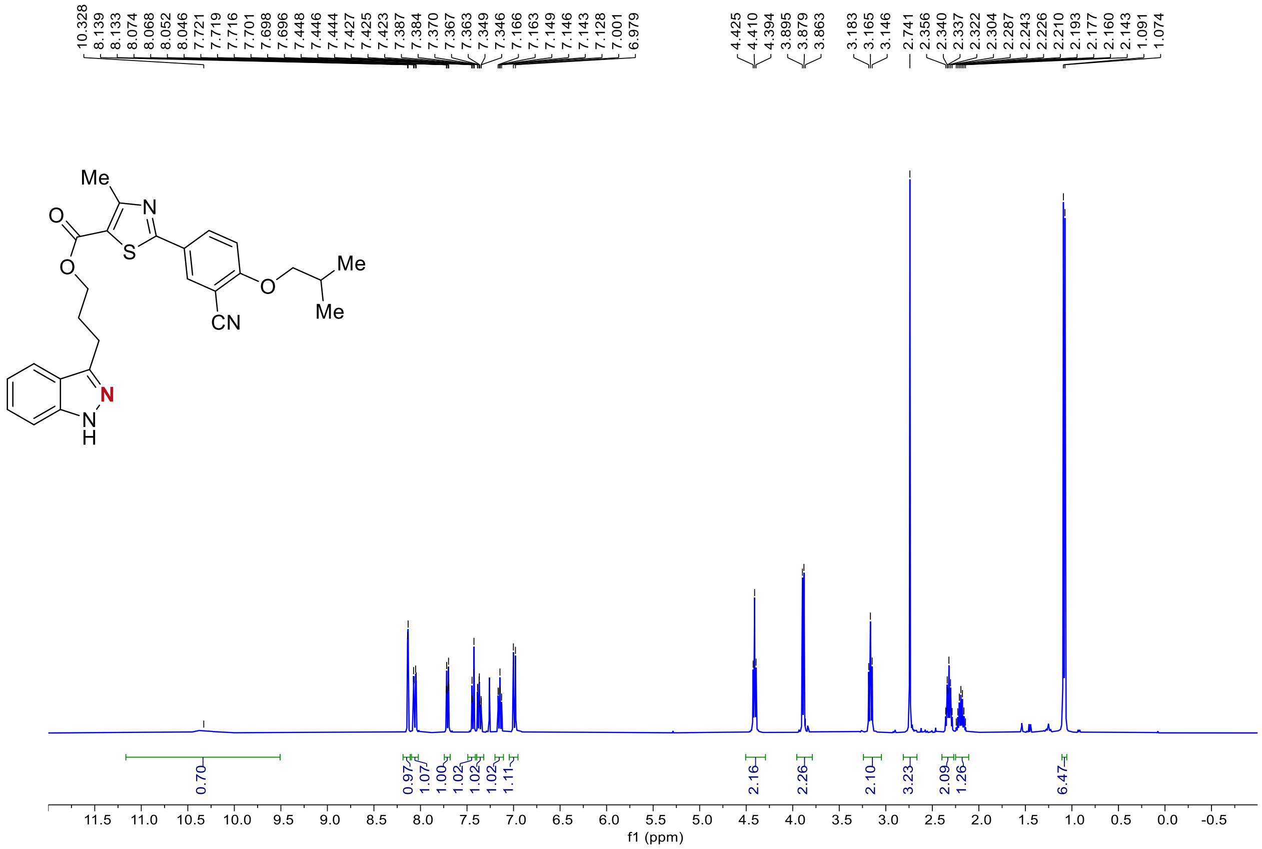

^{13}C NMR (101 MHz, $CDCl_3$)

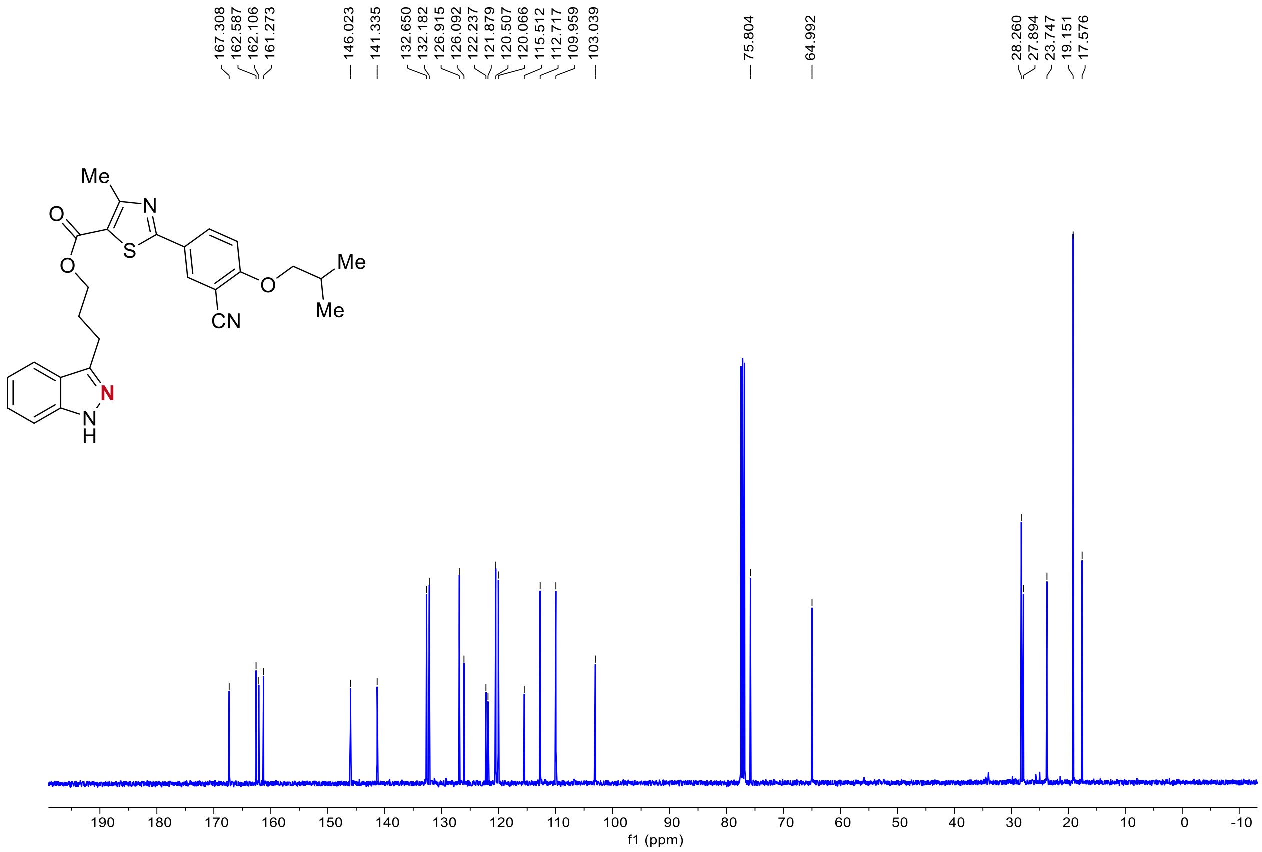

Methyl (*S*)-3-(1*H*-indazol-3-yl)-2-(2,2,2-trifluoroacetamido)propanoate 2u

^{1}H NMR (400 MHz, $CDCl_3$)

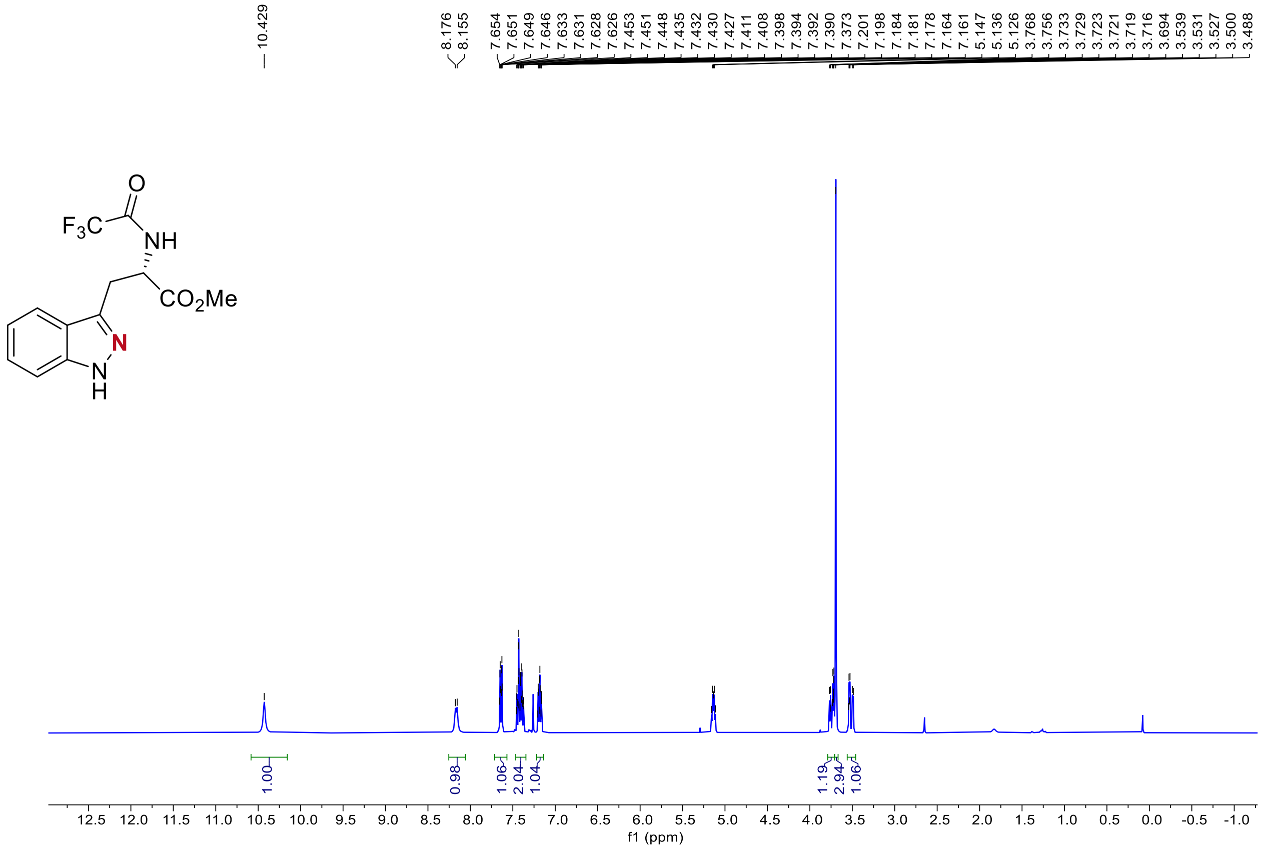

^{13}C NMR (101 MHz, $CDCl_3$)

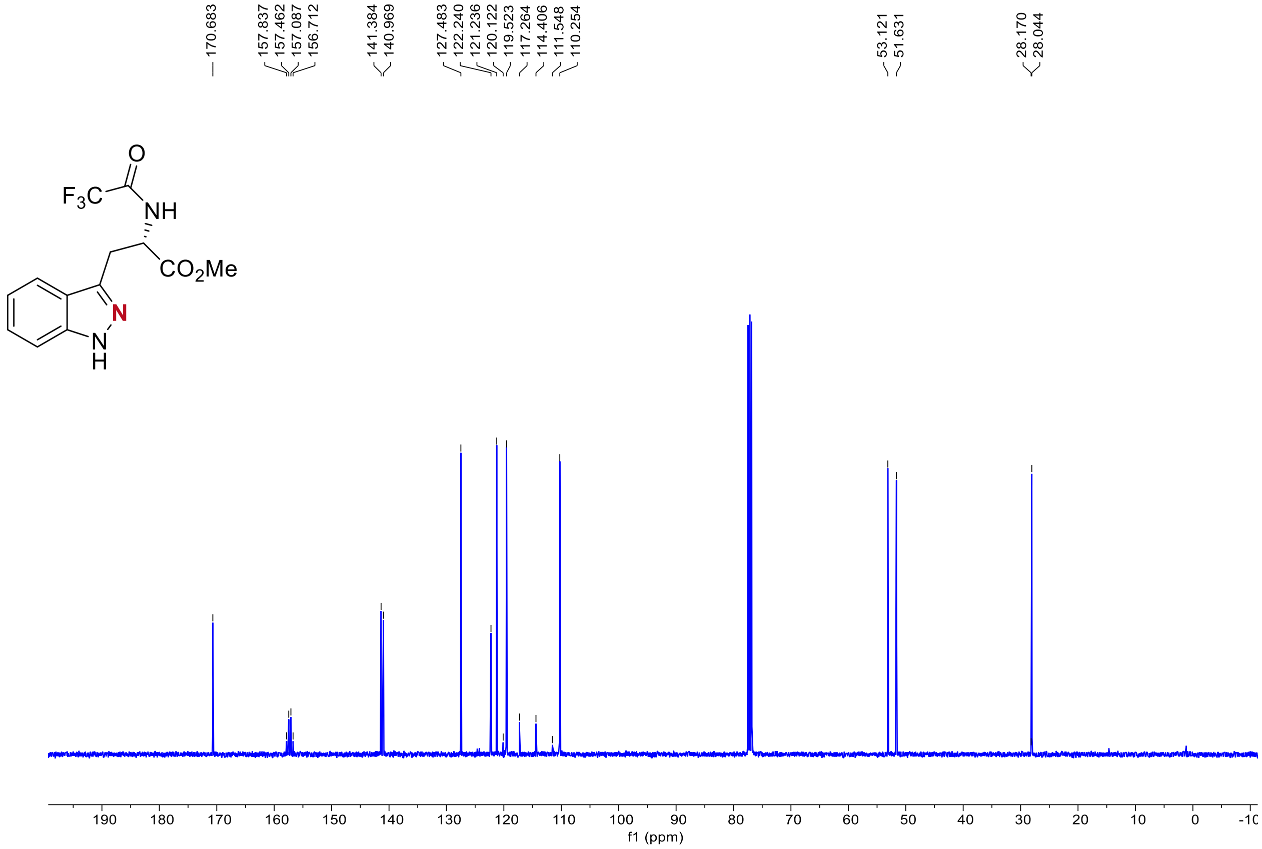

^{19}F NMR (376 MHz, $CDCl_3$)

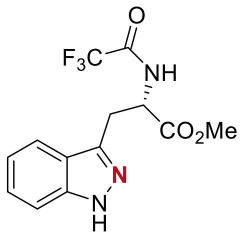

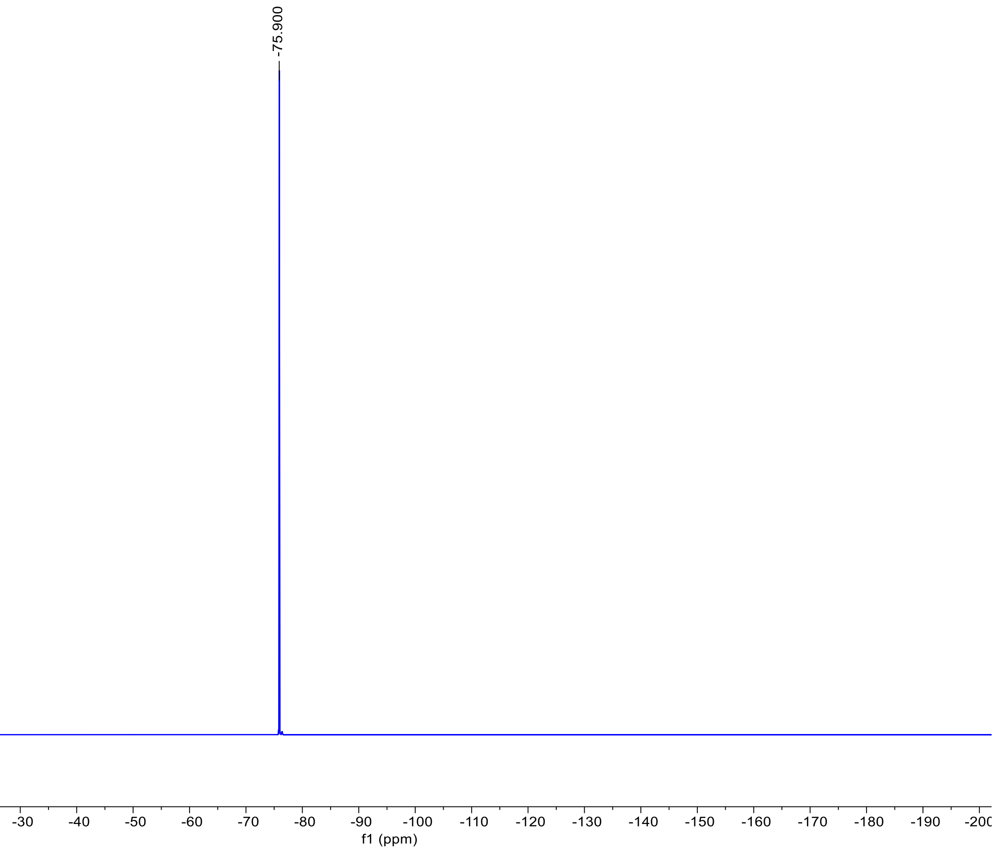

Methyl ((*S*)-3-(1H-indazol-3-yl)-2-(2,2,2-trifluoroacetamido)propanoyl)-*L*-phenylalaninate 2v

^{1}H NMR (400 MHz, $CDCl_3$)

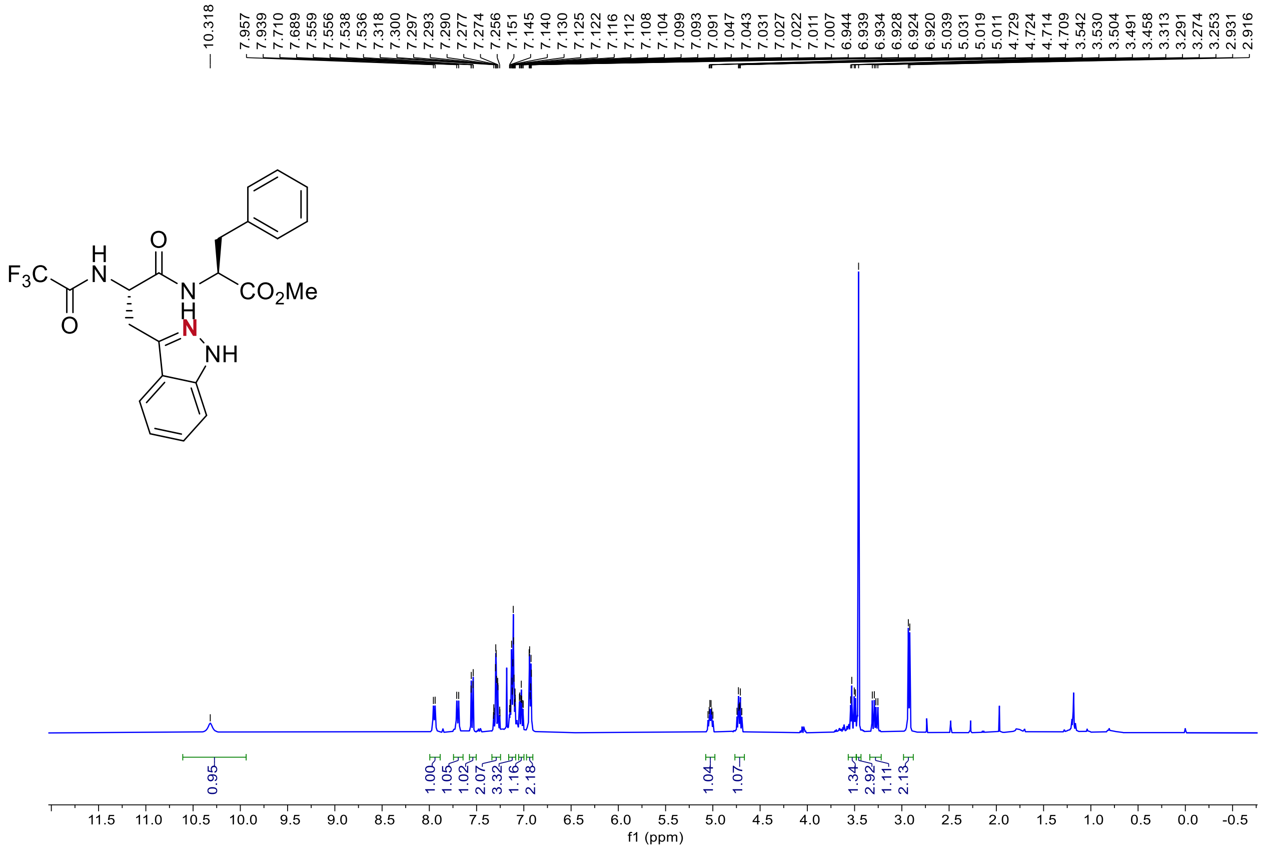

^{13}C NMR (101 MHz, $CDCl_3$)

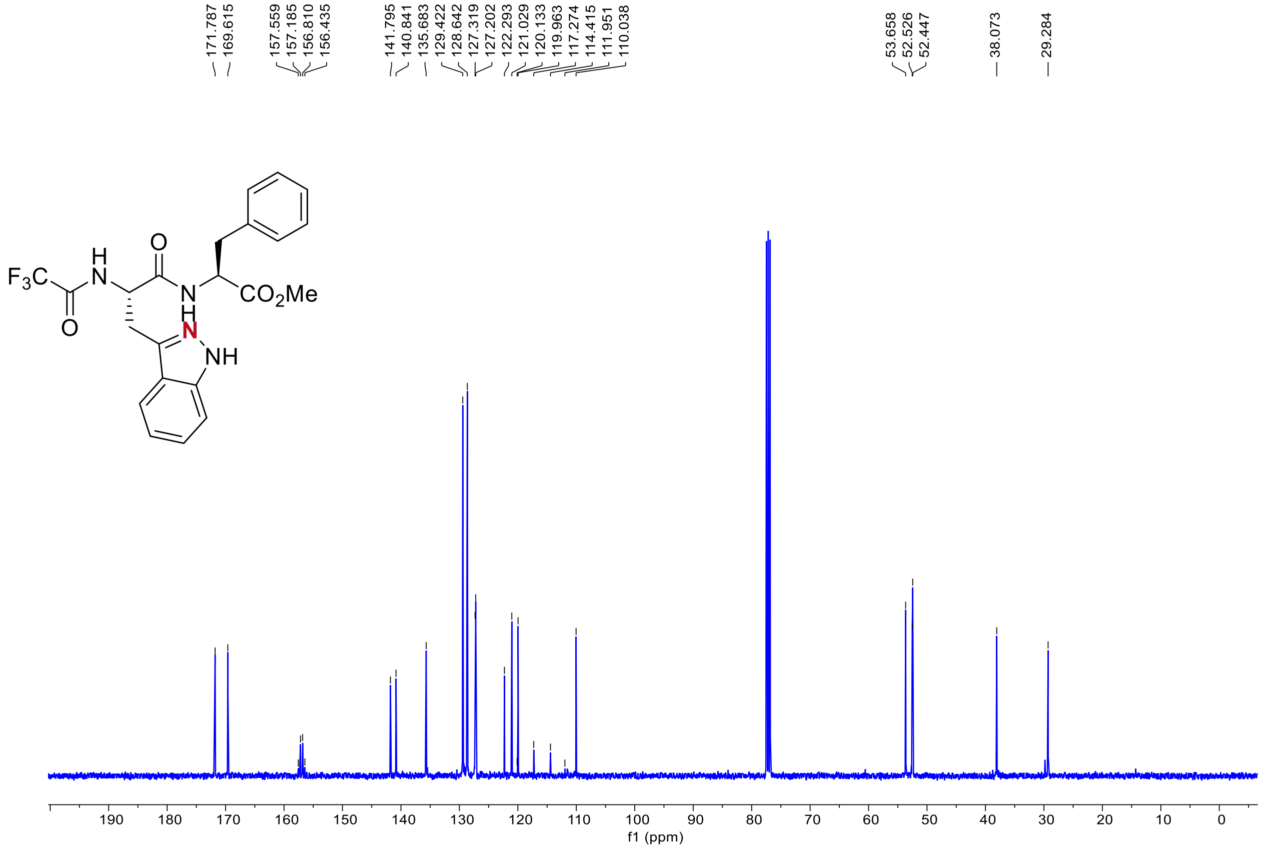

^{19}F NMR (376 MHz, $CDCl_3$)

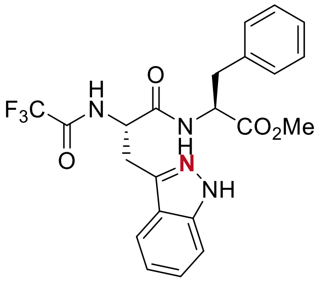

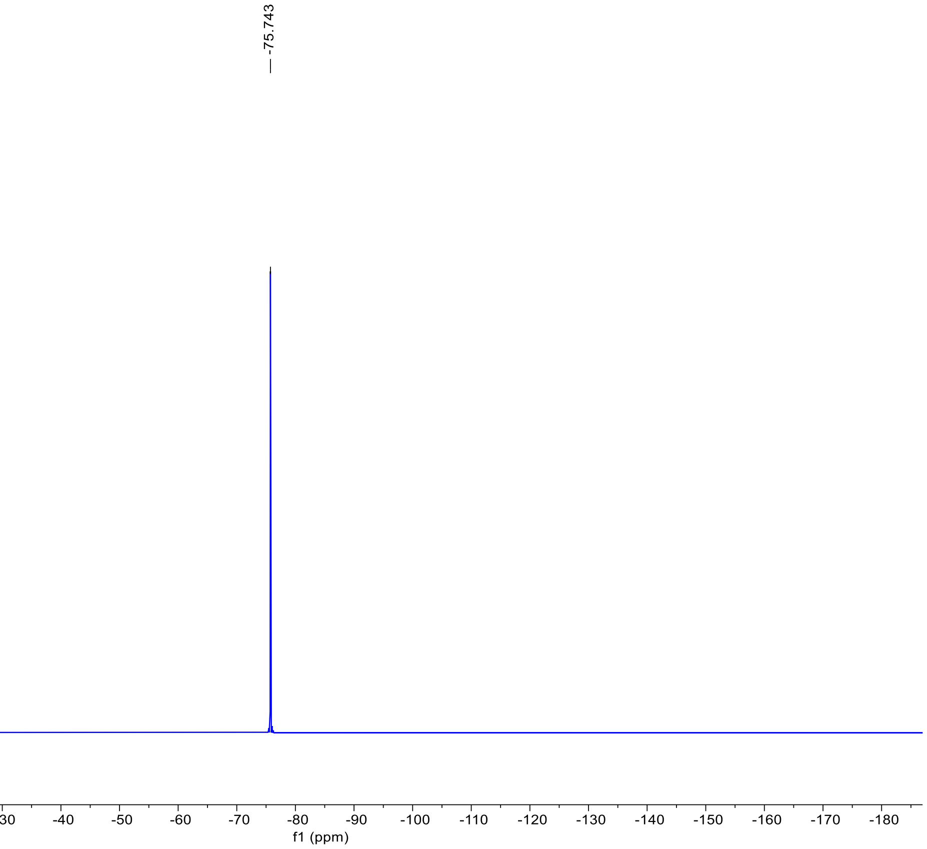

Methyl ((*S*)-2-((*S*)-2-acetamidopropanamido)-3-(1*H*-indazol-3-yl)propanoyl)-*L*-valinate 2w

^{1}H NMR (400 MHz, MeOD)

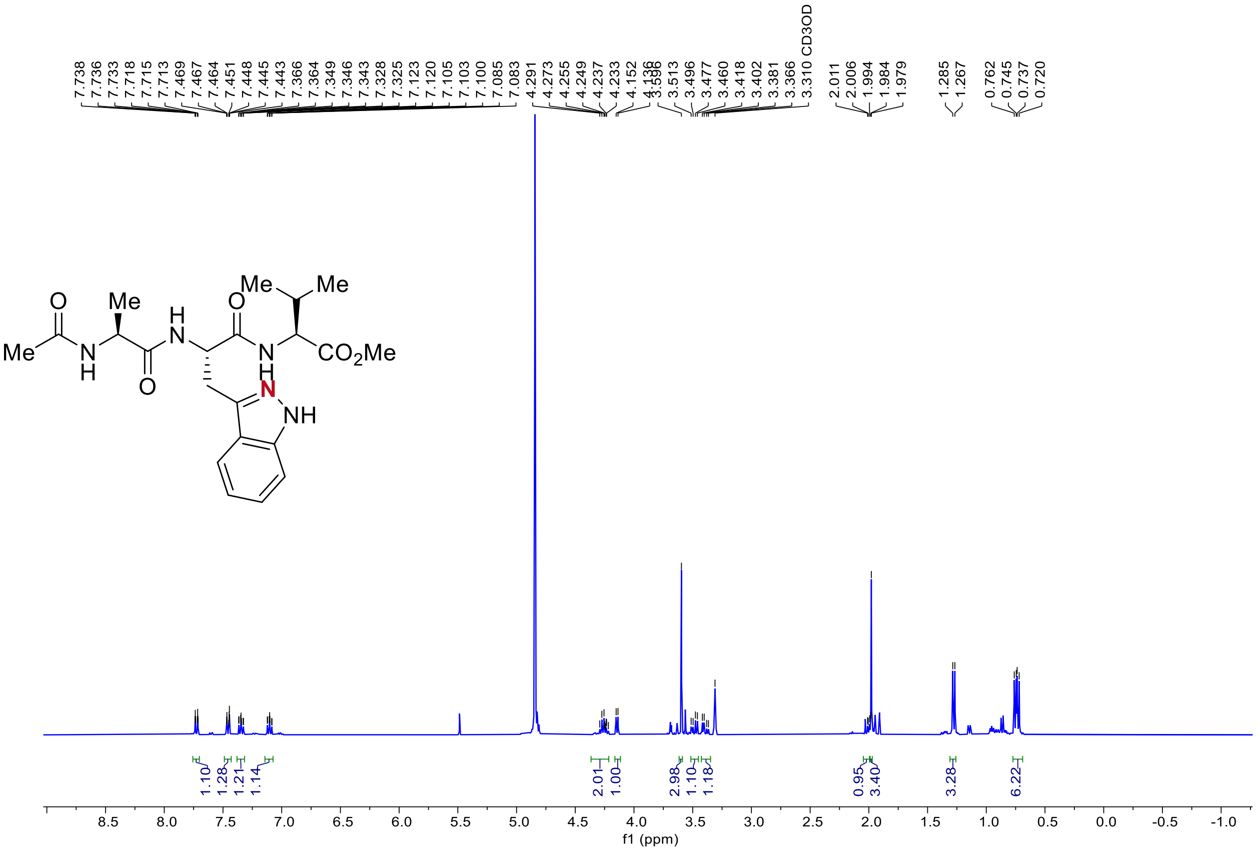

^{13}C NMR (101 MHz, MeOD)

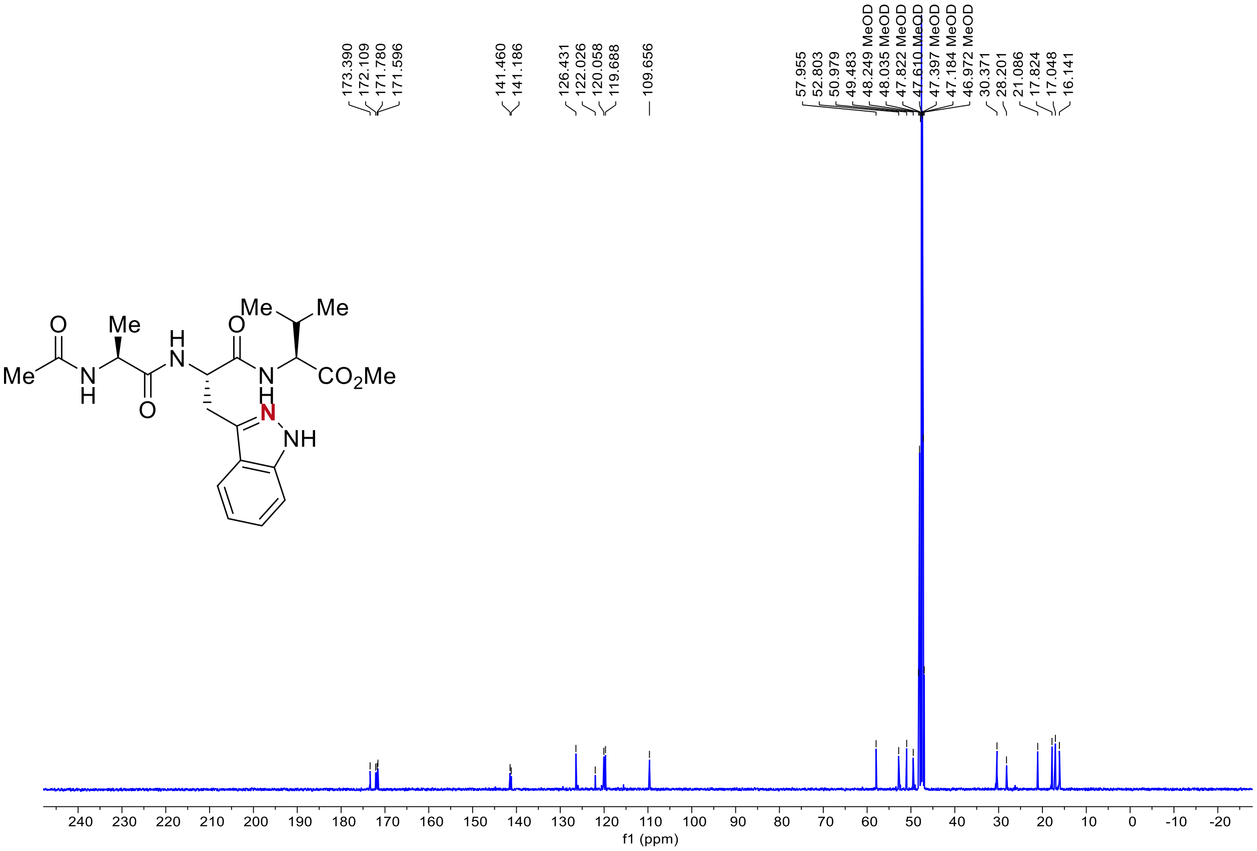

(3*S*,8a*S*)-3-((1*H*-indazol-3-yl)methyl)hexahydropyrrolo[1,2-*a*]pyrazine-1,4-dione 2x

^{1}H NMR (400 MHz, DMSO)

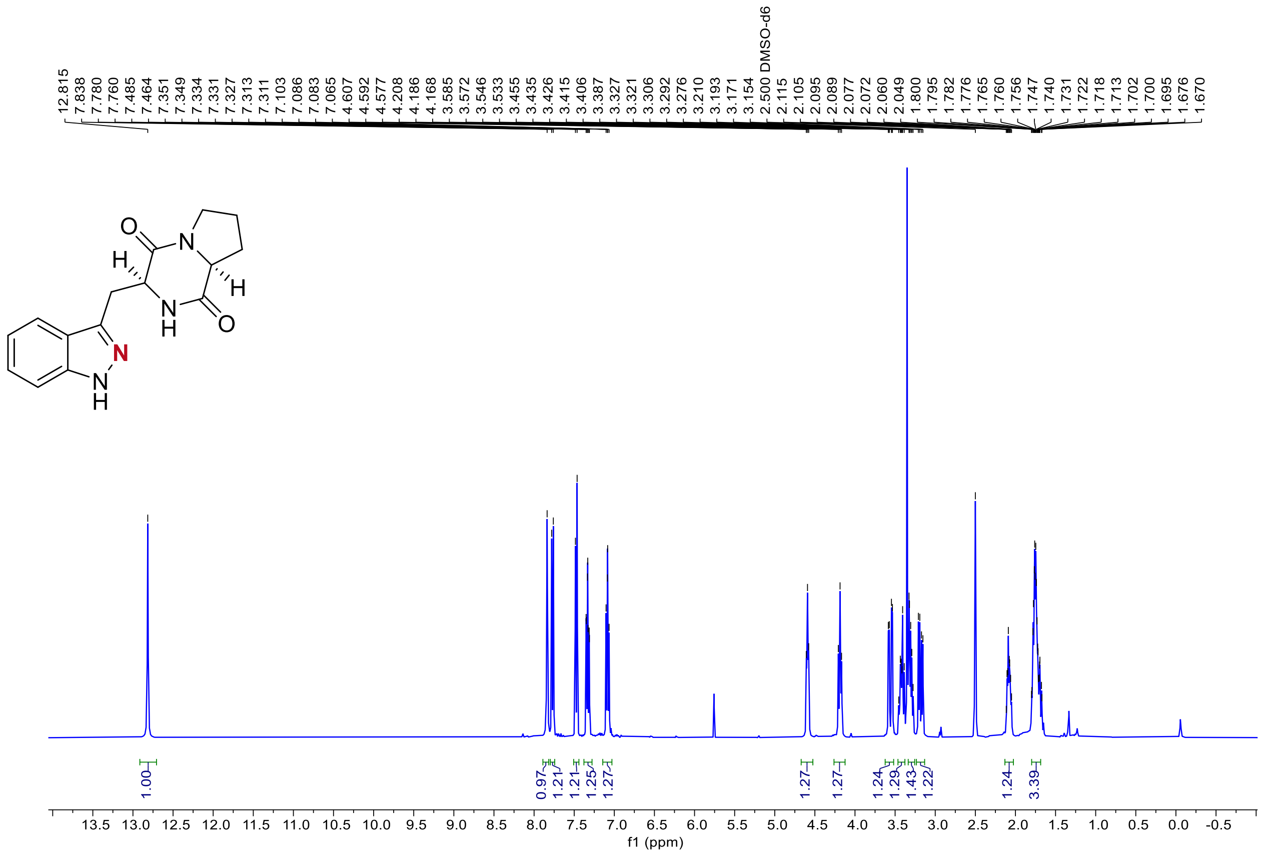

^{13}C NMR (101 MHz, DMSO)

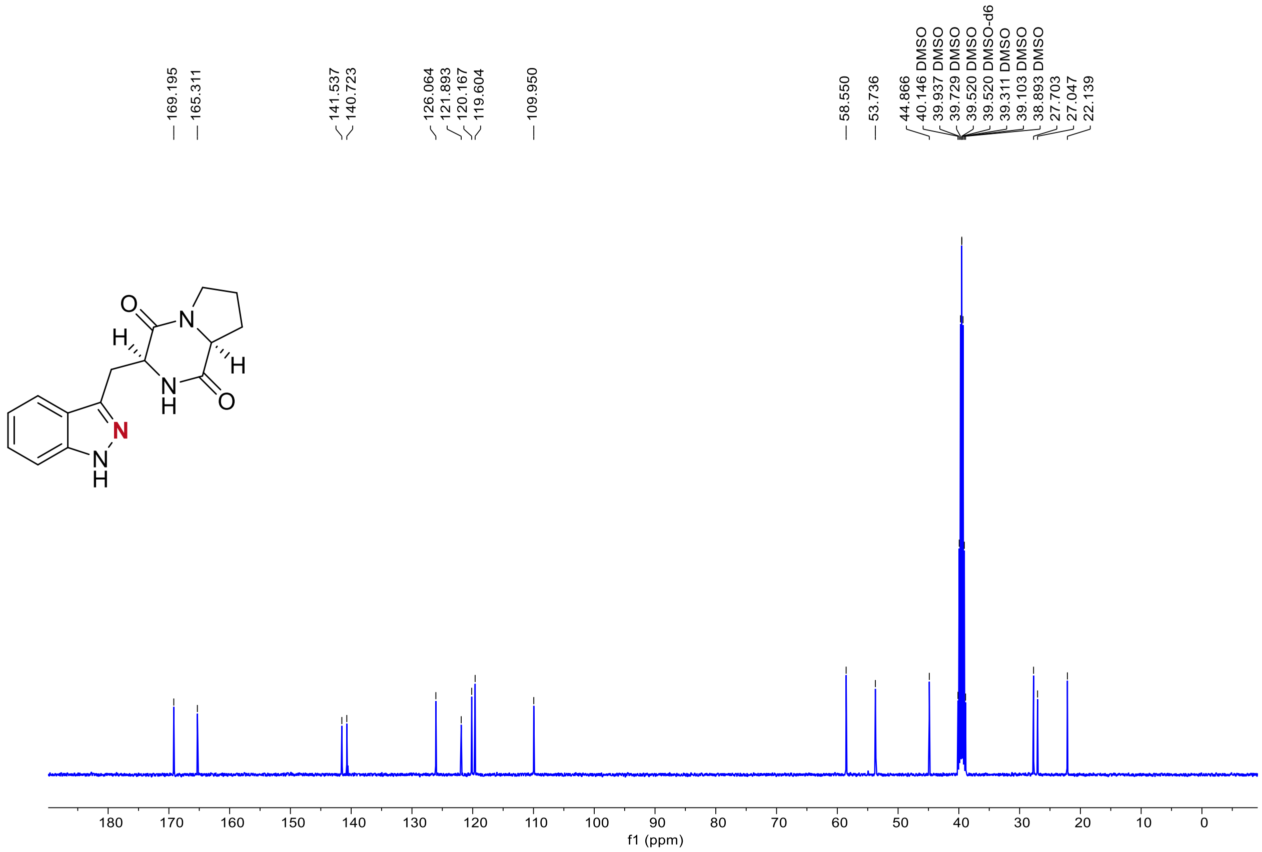

(3*S*,8a*S*)-3-((1-Tosyl-1*H*-benzo[*d*]imidazol-2-yl)methyl)hexahydropyrrolo[1,2-*a*]pyrazine-1,4-dione

Ts-3x **^{1}H NMR** (400 MHz, $CDCl_3$)

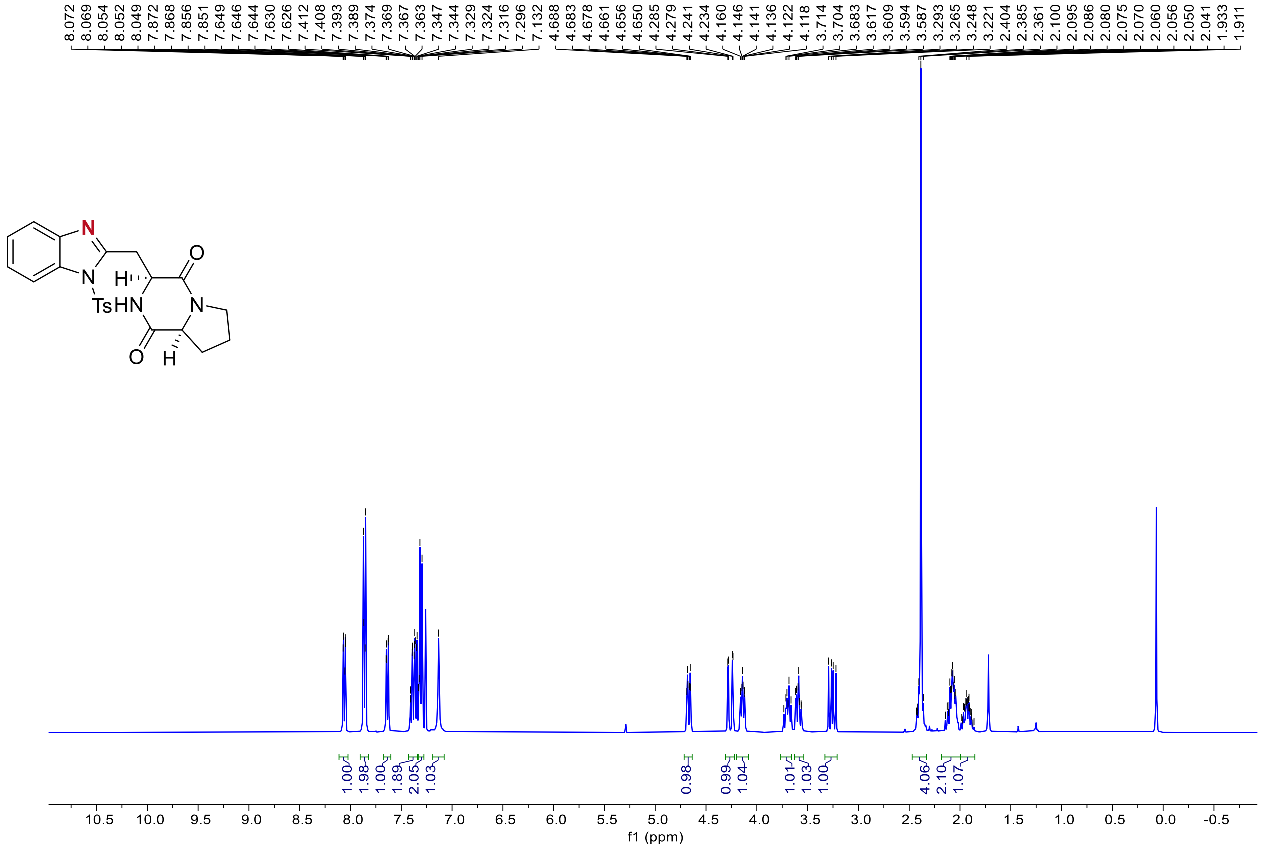

^{13}C NMR (101 MHz, $CDCl_3$)

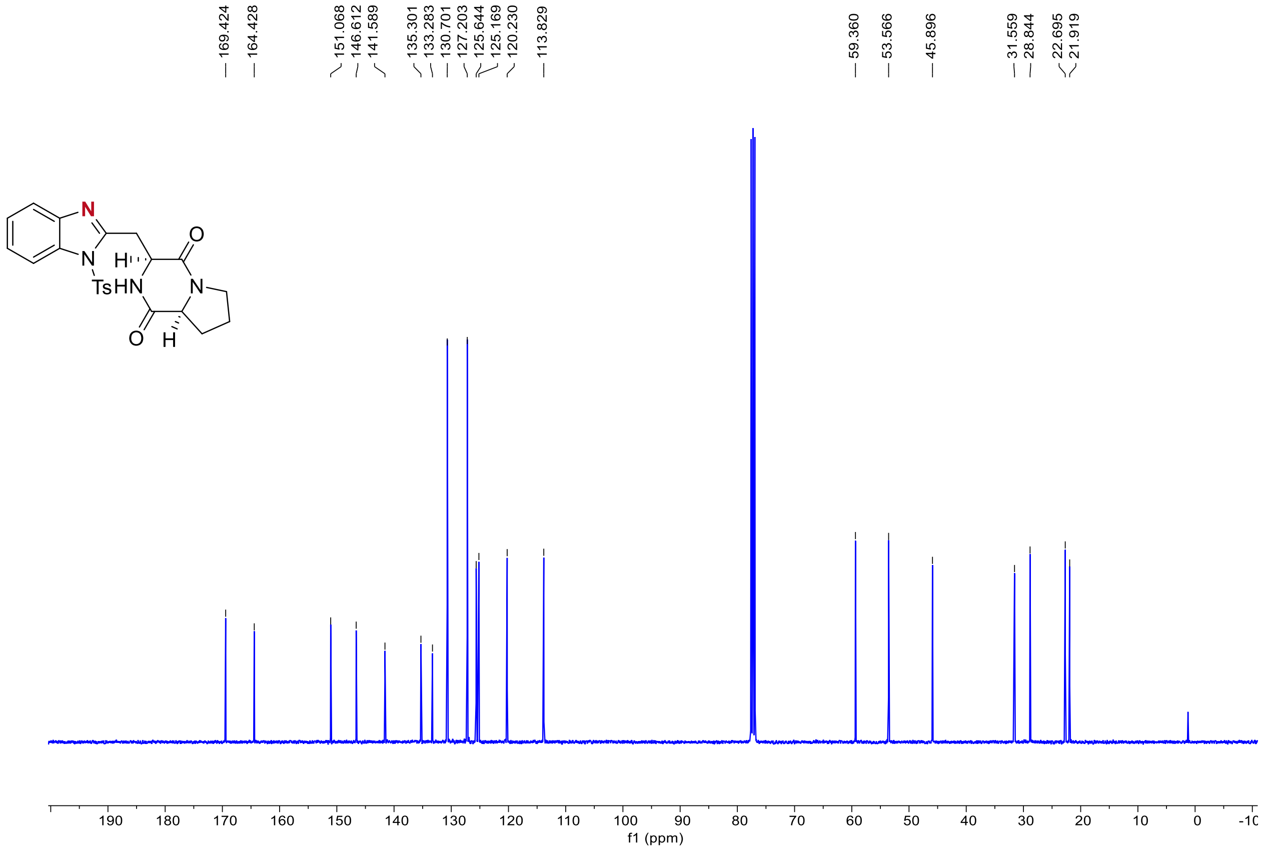

(3*S*,8a*S*)-3-((1-Tosyl-1*H*-indazol-3-yl)methyl)hexahydropyrrolo[1,2-*a*]pyrazine-1,4-dione Ts-2x

^{1}H NMR (400 MHz, $CDCl_3$)

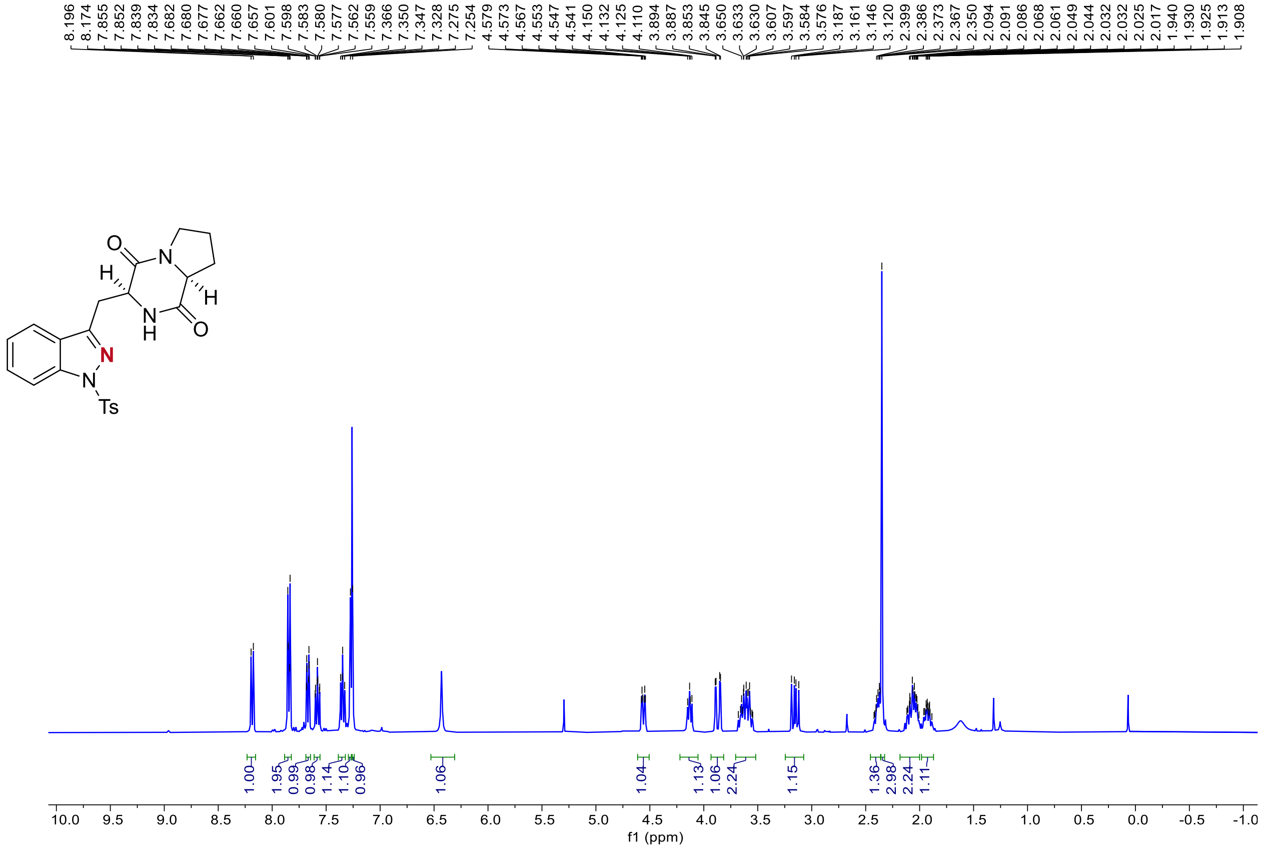

^{13}C NMR (101 MHz, $CDCl_3$)

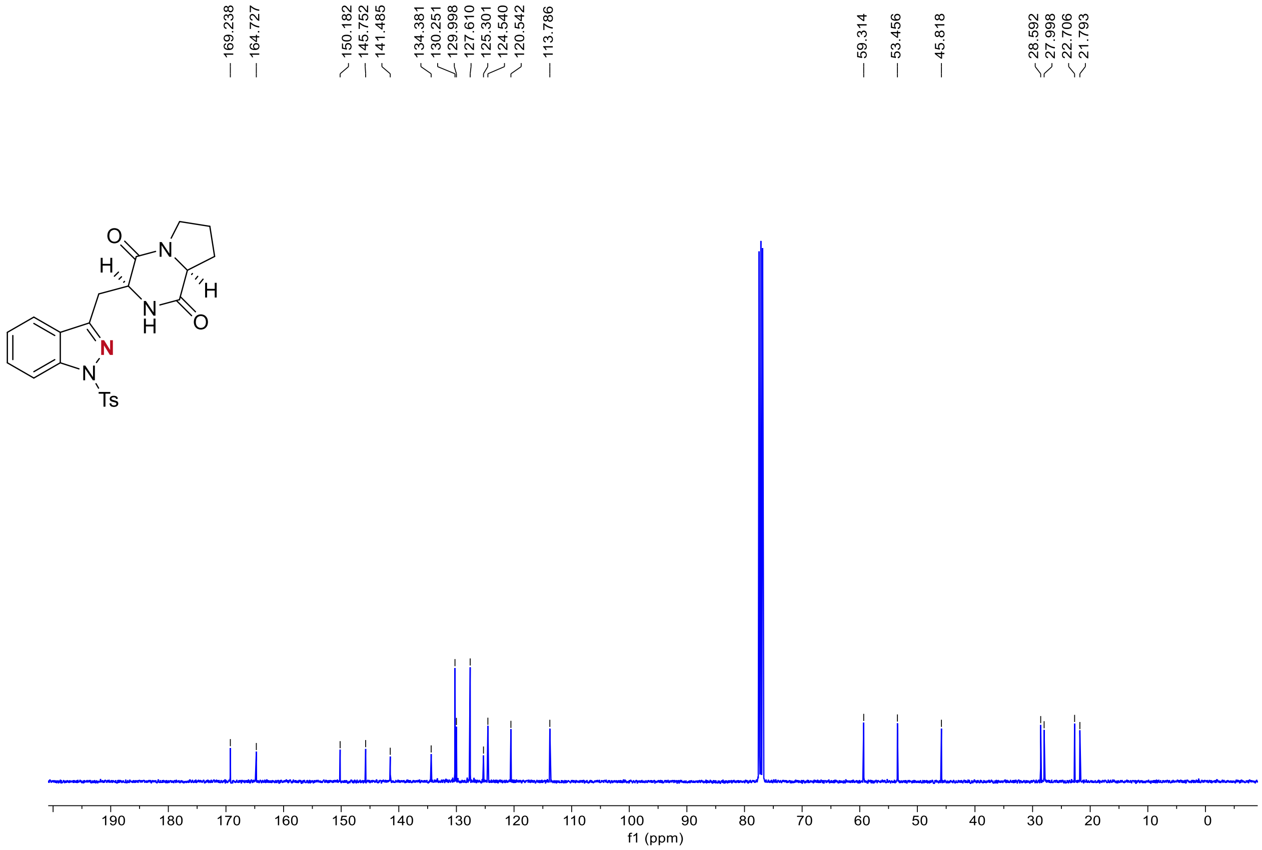

Ethyl 3-(2-(2,2,2-trifluoroacetamido)ethyl)-1*H*-indazole-1-carboxylate Eoc-**2y**

^{1}H NMR (400 MHz, $CDCl_3$)

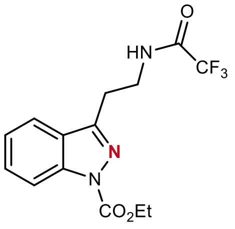

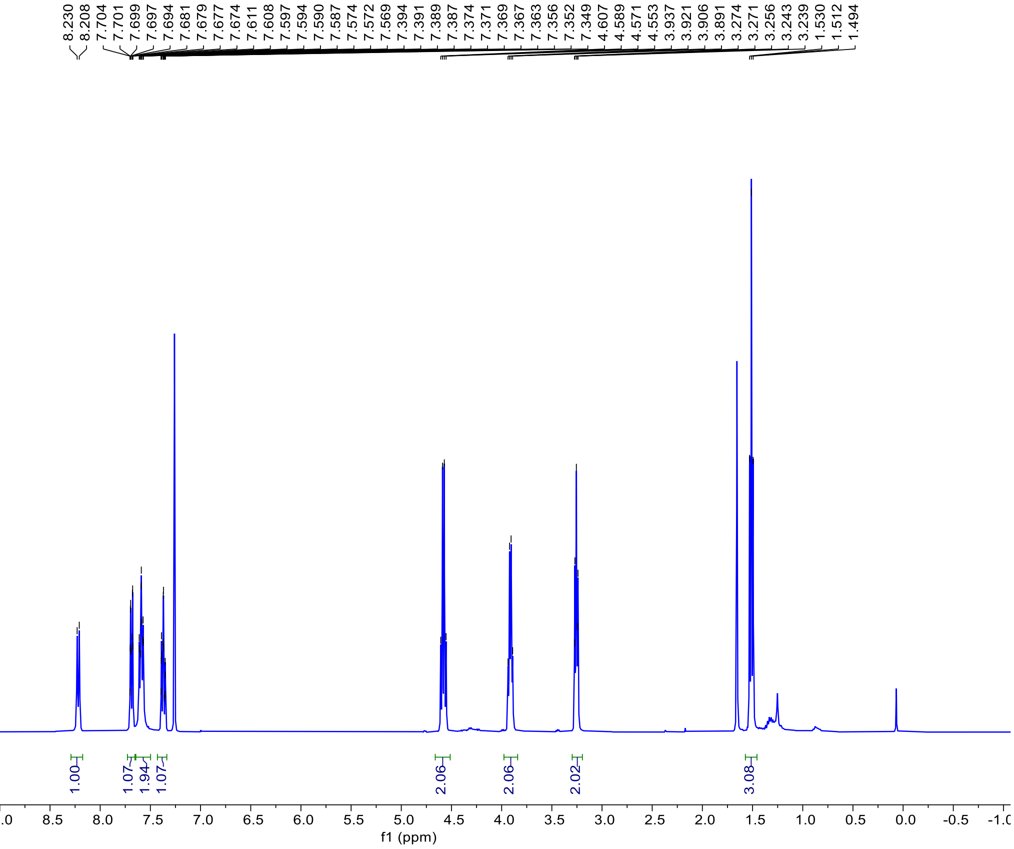

^{13}C NMR (101 MHz, $CDCl_3$)

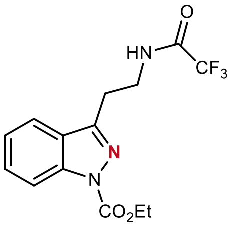

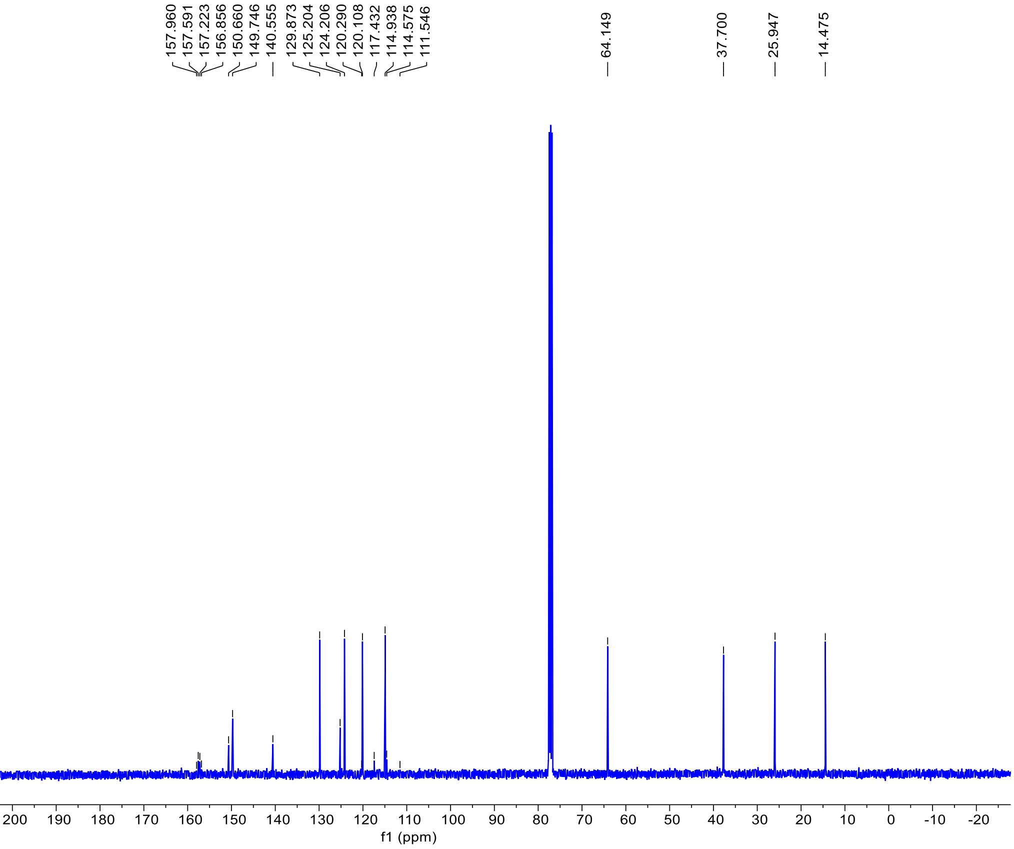

^{19}F NMR (282 MHz, $CDCl_3$)

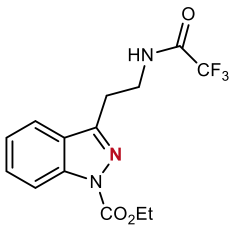

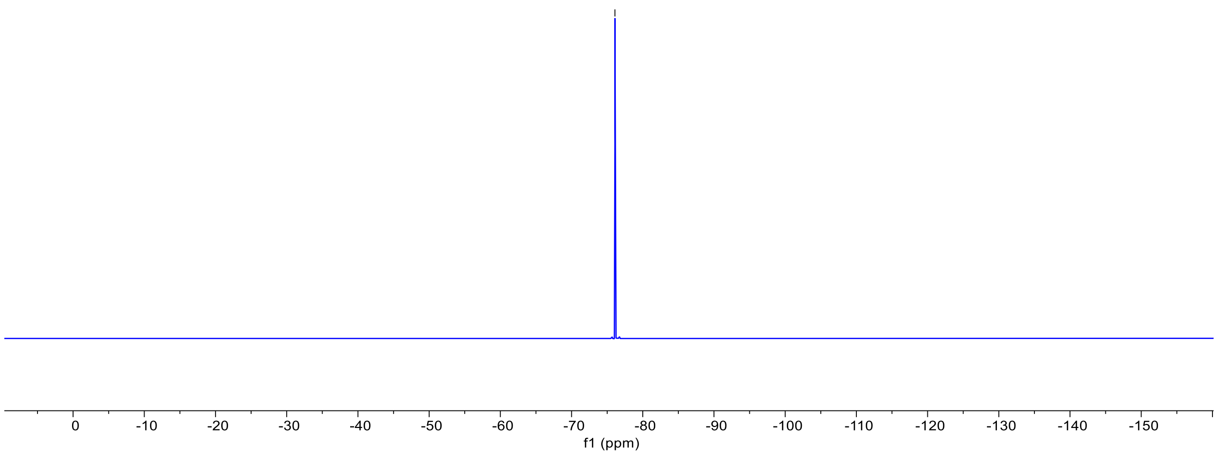

Ethyl 2-(2-(2,2,2-trifluoroacetamido)ethyl)-1*H*-benzo[*d*]imidazole-1-carboxylate Eoc-**3y**

^{1}H NMR (400 MHz, $CDCl_3$)

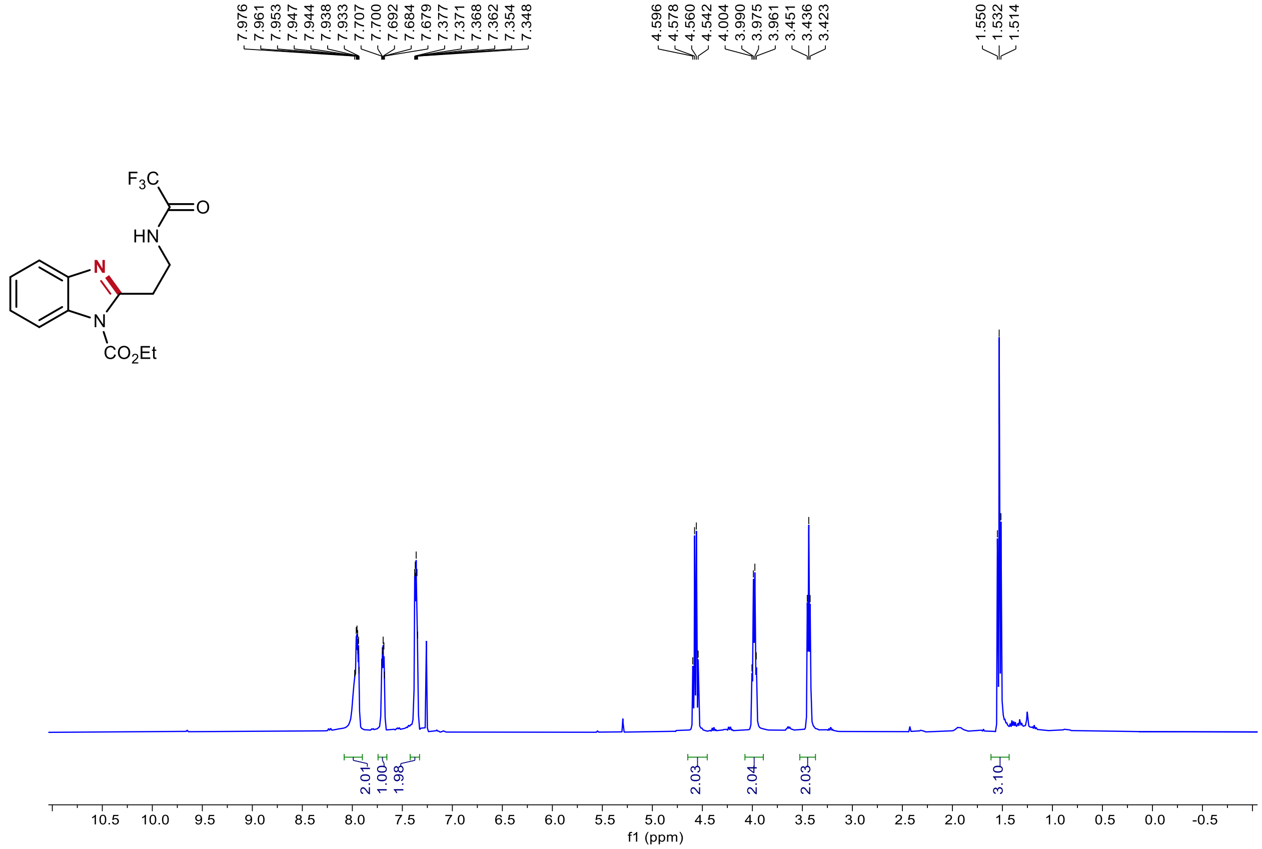

^{13}C NMR (76 MHz, $CDCl_3$)

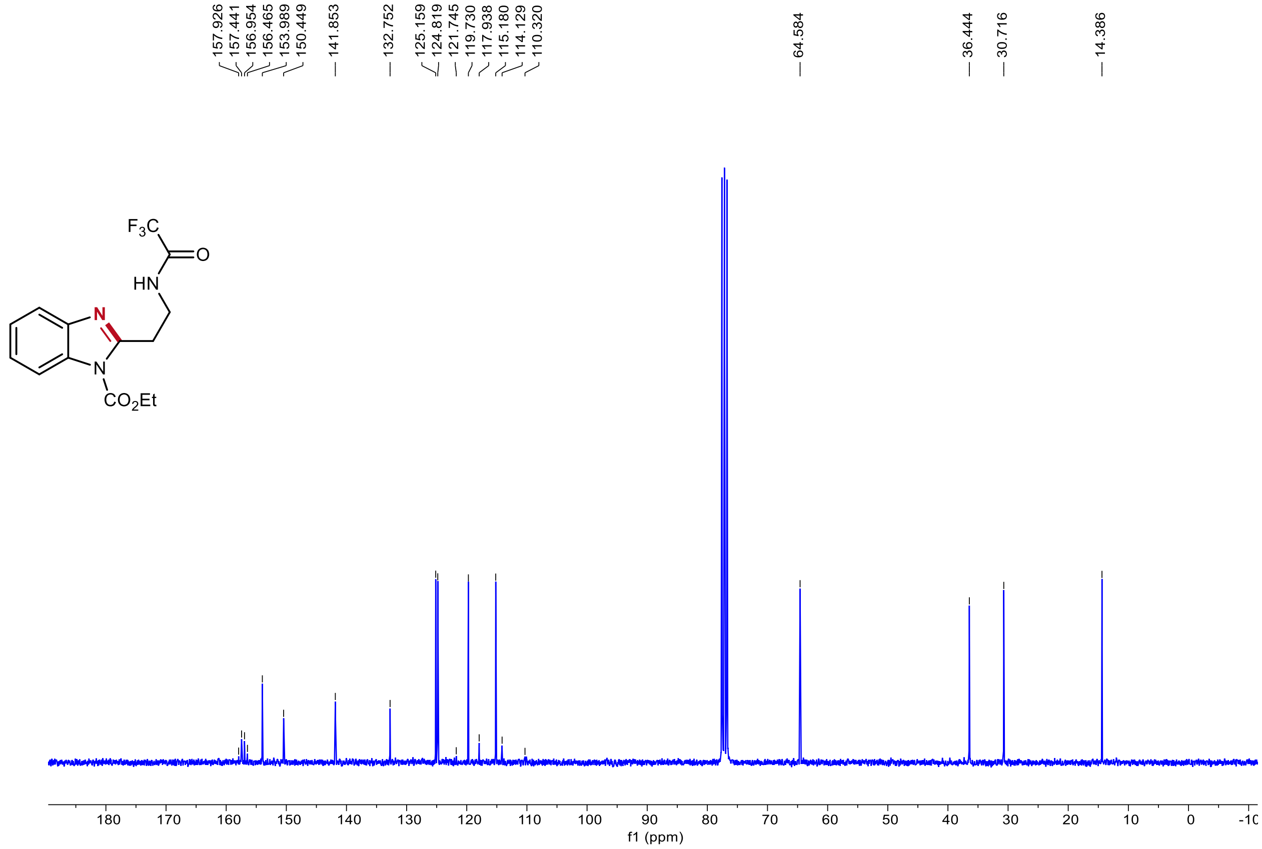

^{19}F NMR (282 MHz, $CDCl_3$)

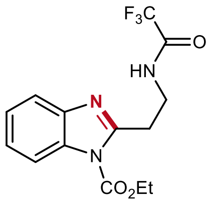

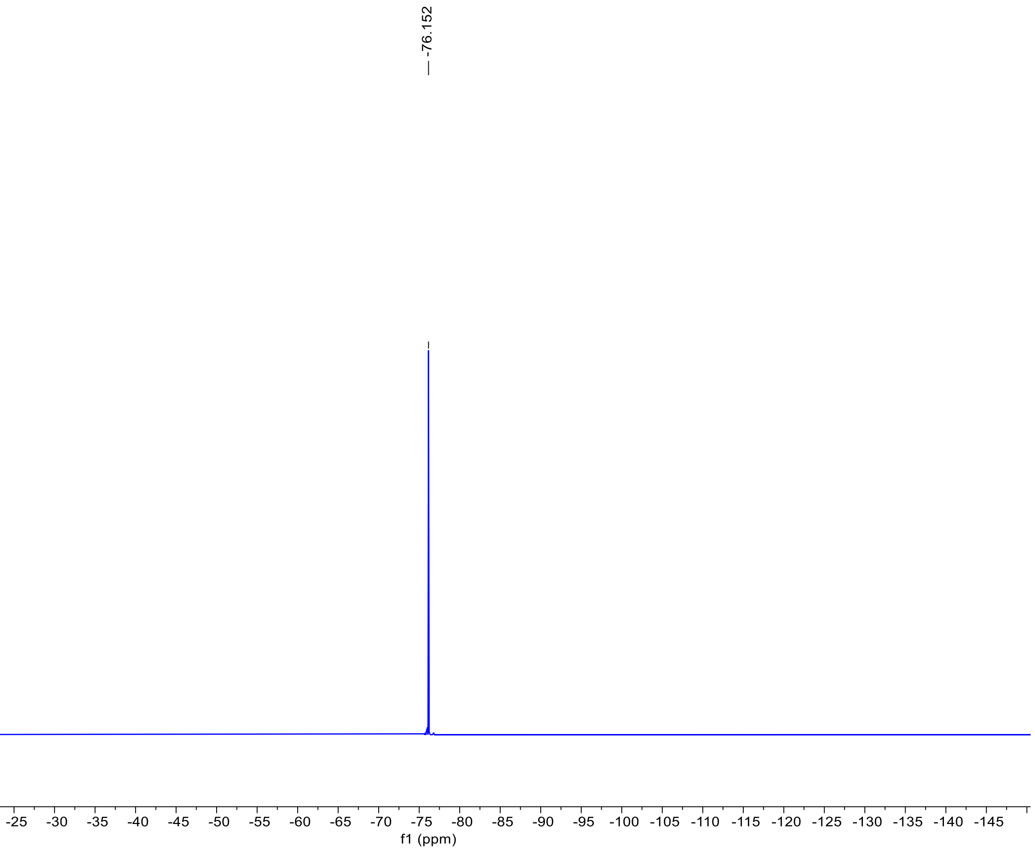

N-(2-(1*H*-indazol-3-yl)ethyl)-2,2,2-trifluoroacetamide 2y

^{1}H NMR (400 MHz, CD_3CN)

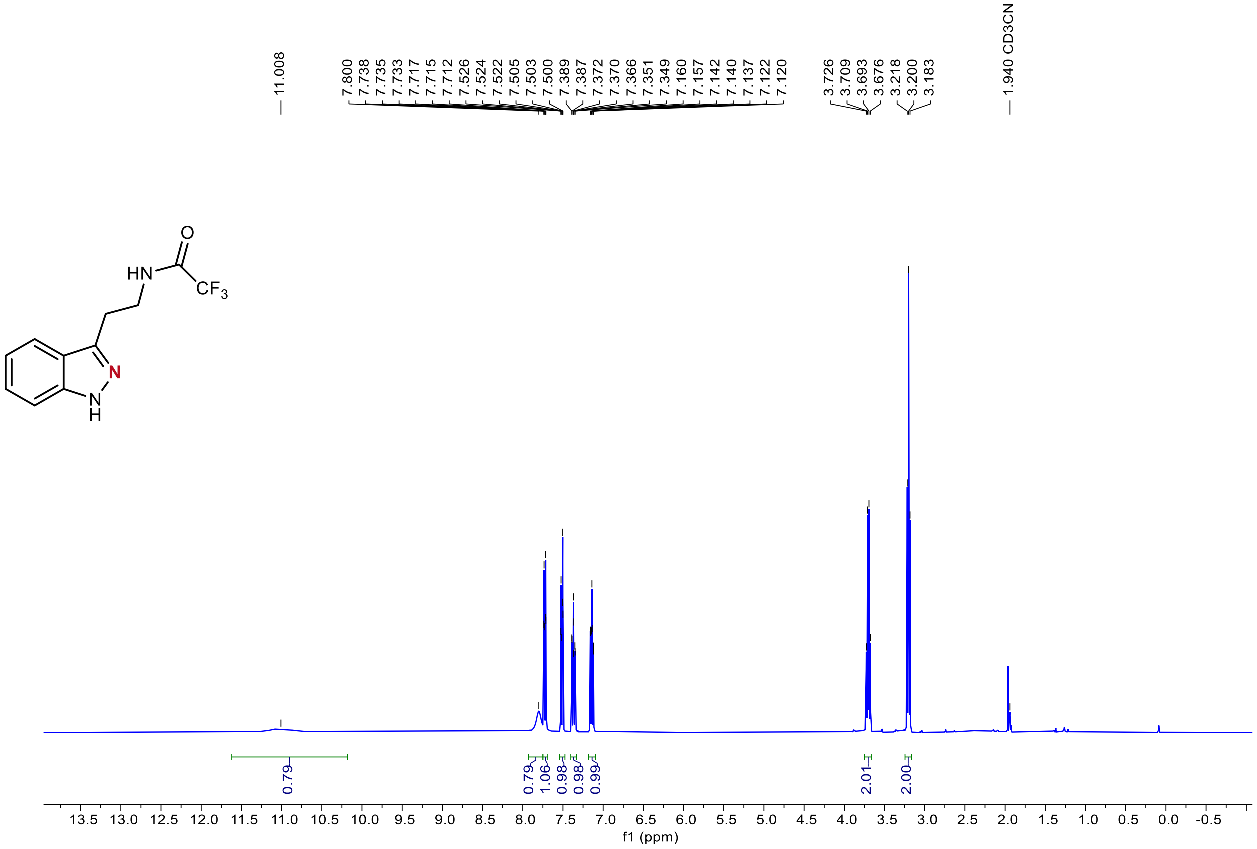

^{13}C NMR (101 MHz, CD_3CN)

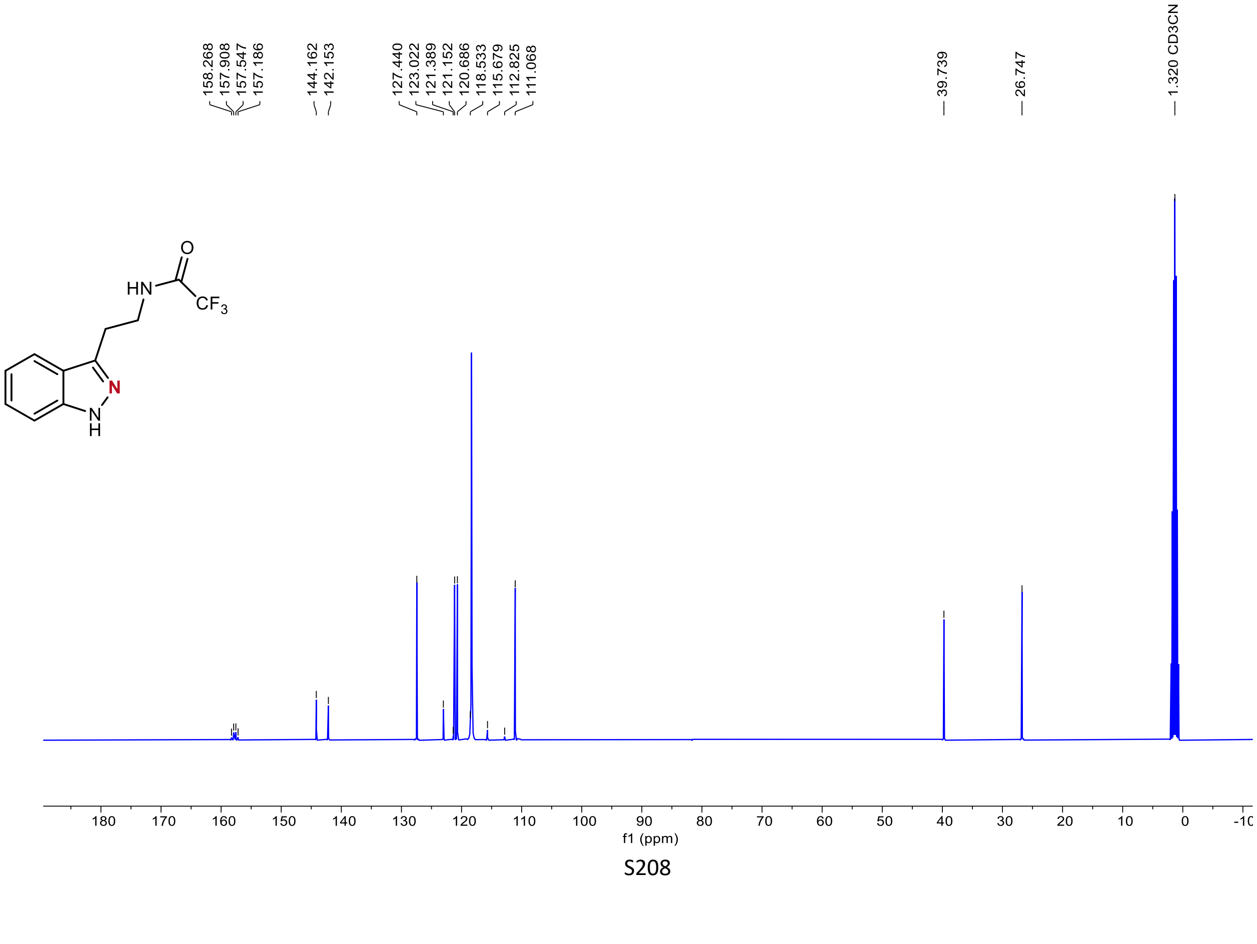

^{19}F NMR (376 MHz, CD_3CN) δ -76.8.

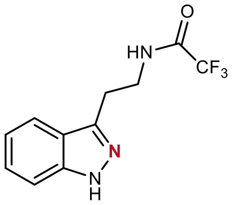

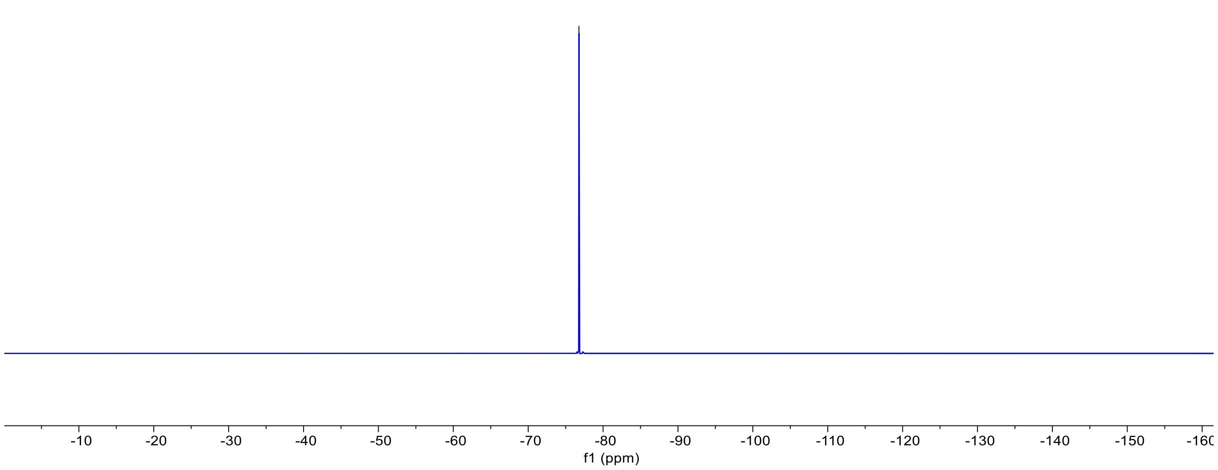

2,2,2-trifluoro-*N*-(2-(1-tosyl-1H-benzo[*d*]imidazol-2-yl)ethyl)acetamide Ts-**3y**

^{1}H NMR (400 MHz, $CDCl_3$)

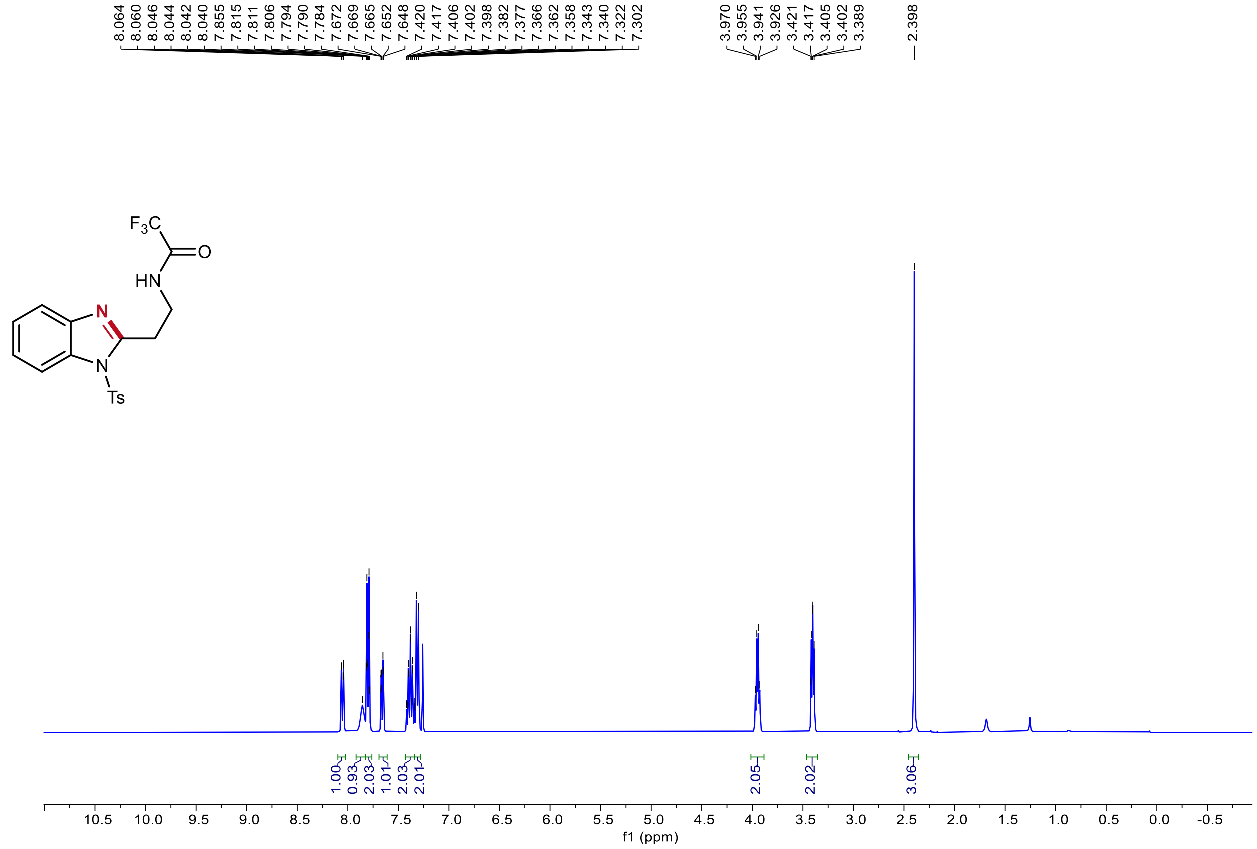

^{13}C NMR (101 MHz, $CDCl_3$)

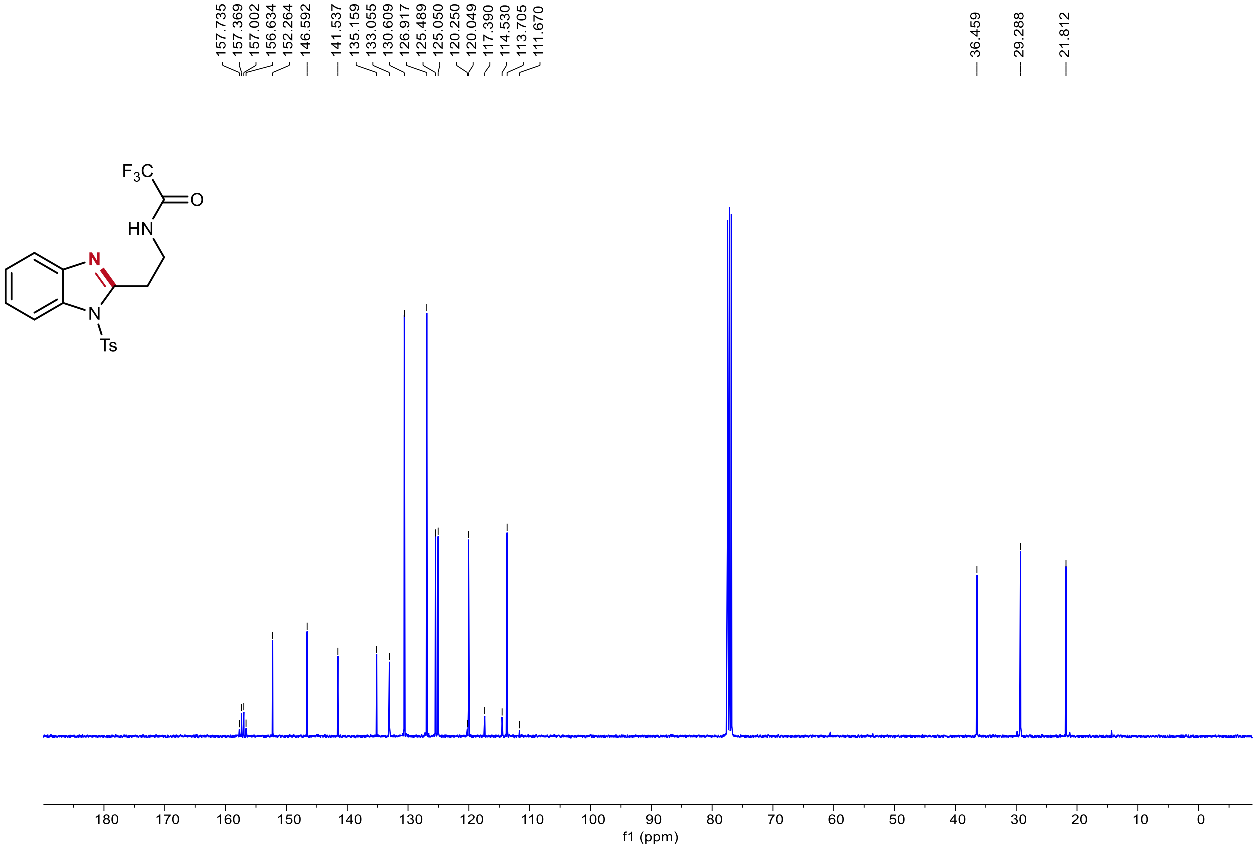

^{19}F NMR (376 MHz, $CDCl_3$)

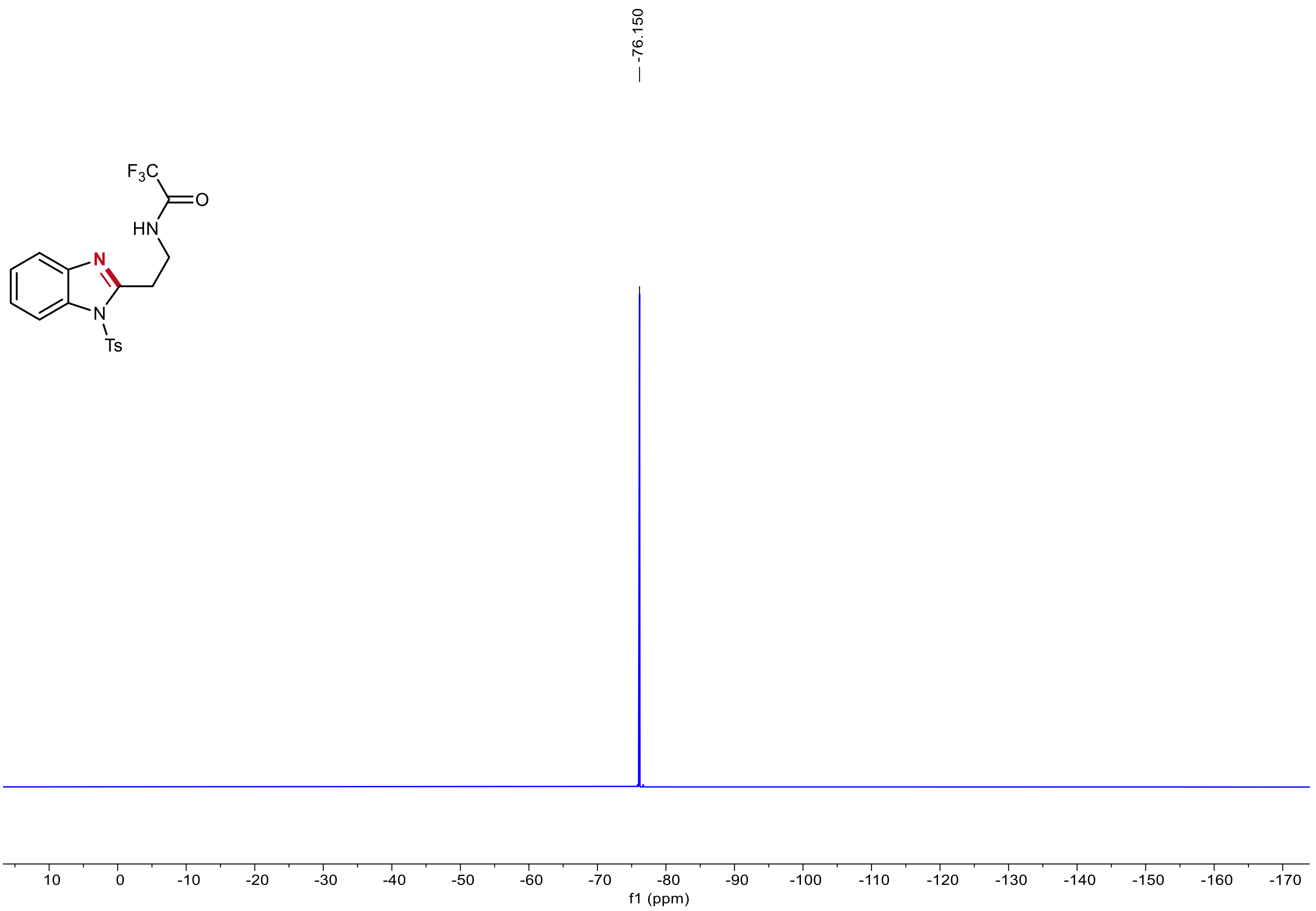

2,2,2-trifluoro-*N*-(2-(1-tosyl-1*H*-indazol-3-yl)ethyl)acetamide Ts-**2y**

^{1}H NMR (400 MHz, $CDCl_3$)

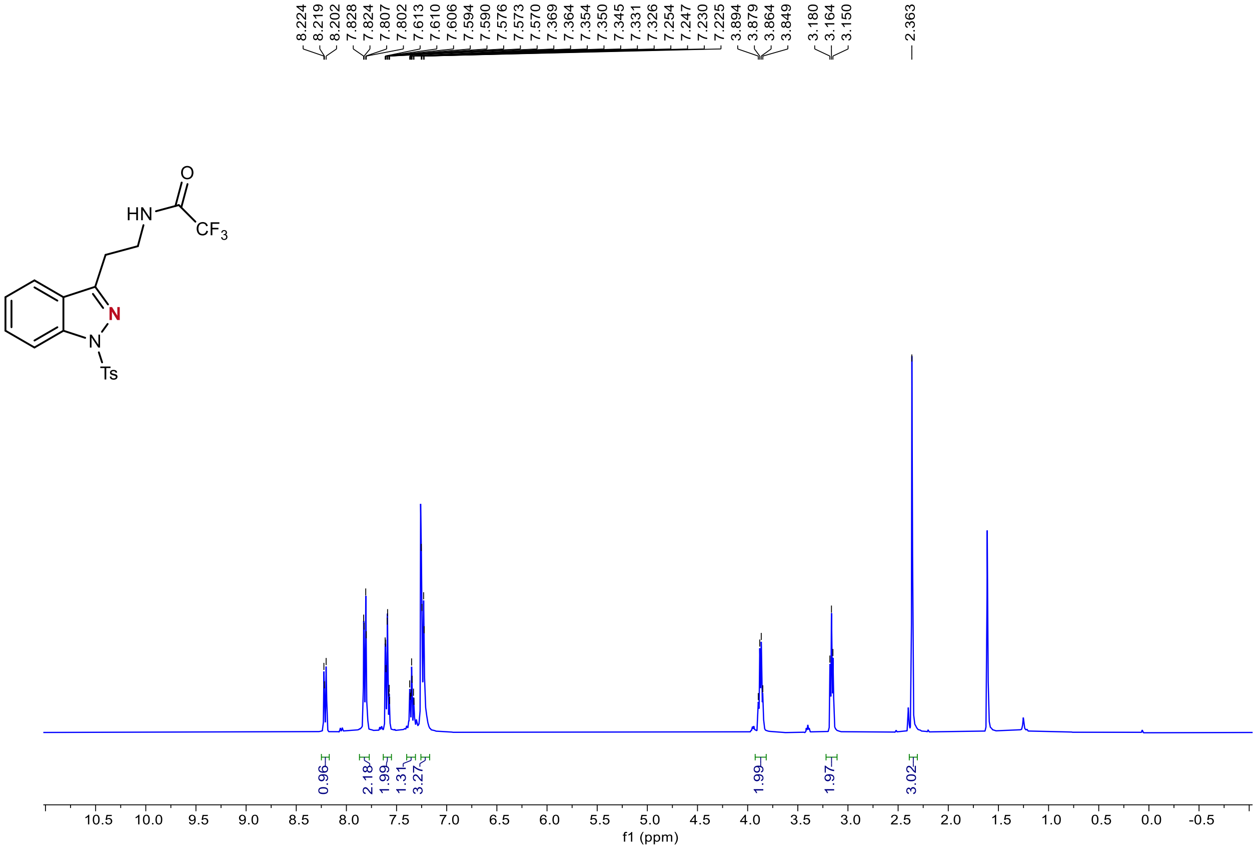

^{13}C NMR{^{19}F} (126 MHz, $CDCl_3$)

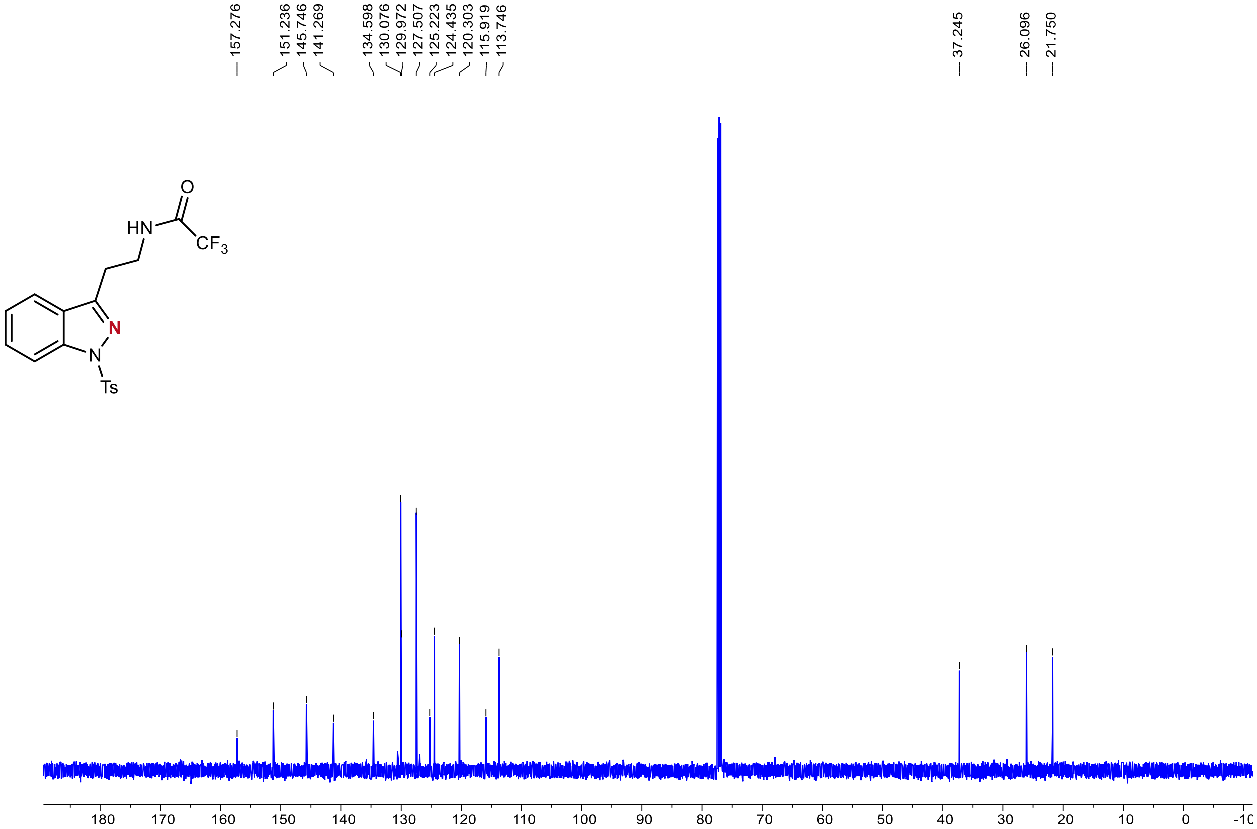

^{19}F NMR (470 MHz, cdcl$_3$)

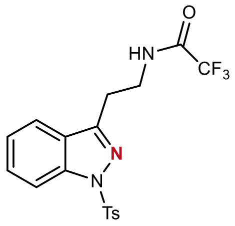

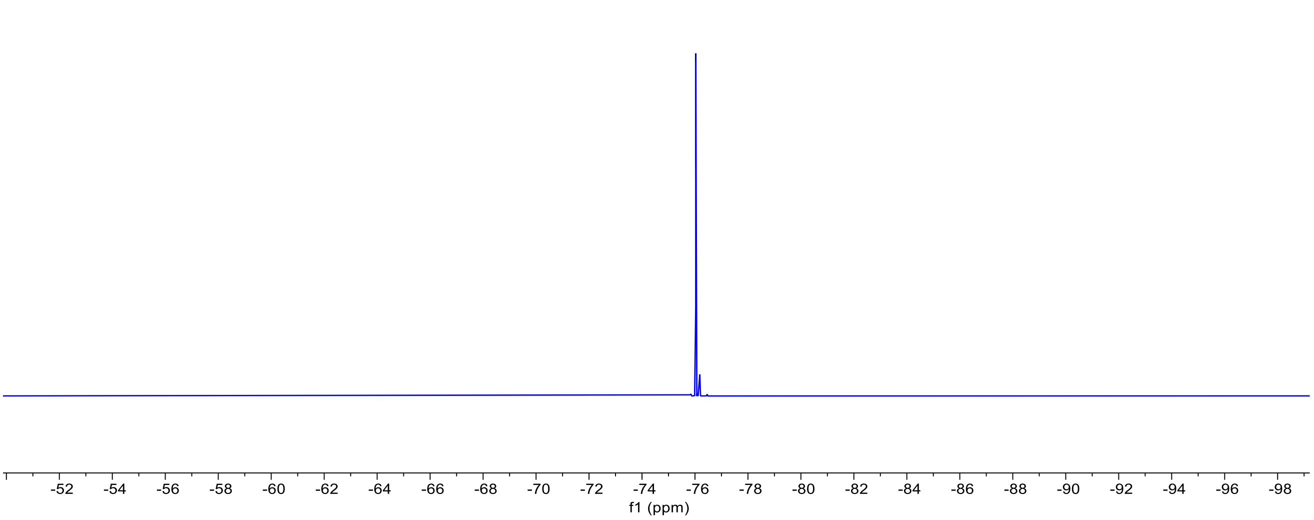

3-(1*H*-indazol-3-yl)-1-morpholinopropan-1-one 2z

^{1}H NMR (400 MHz, $CDCl_3$)

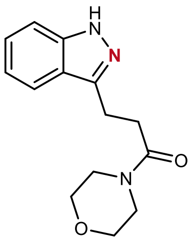

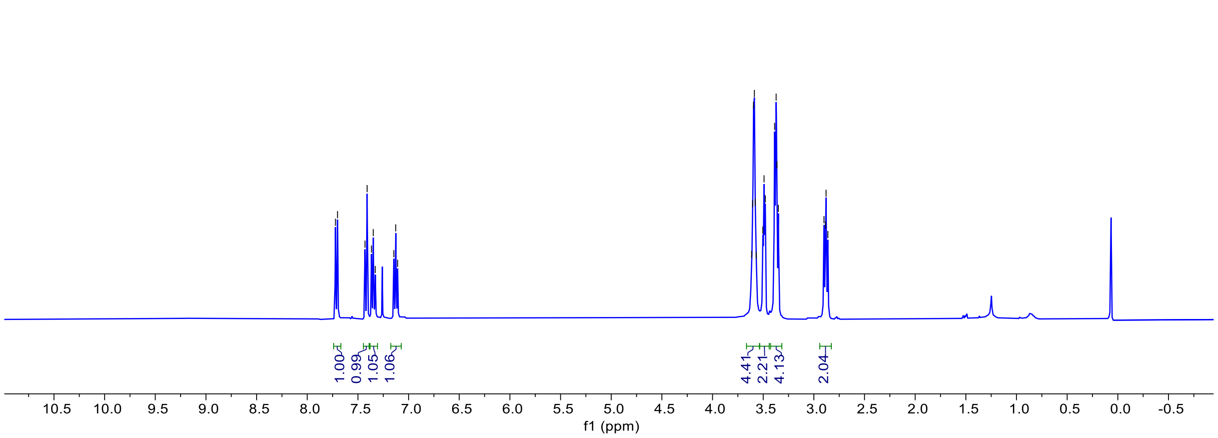

^{13}C NMR (76 MHz, $CDCl_3$)

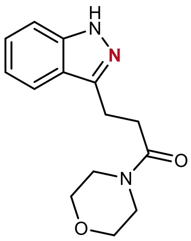

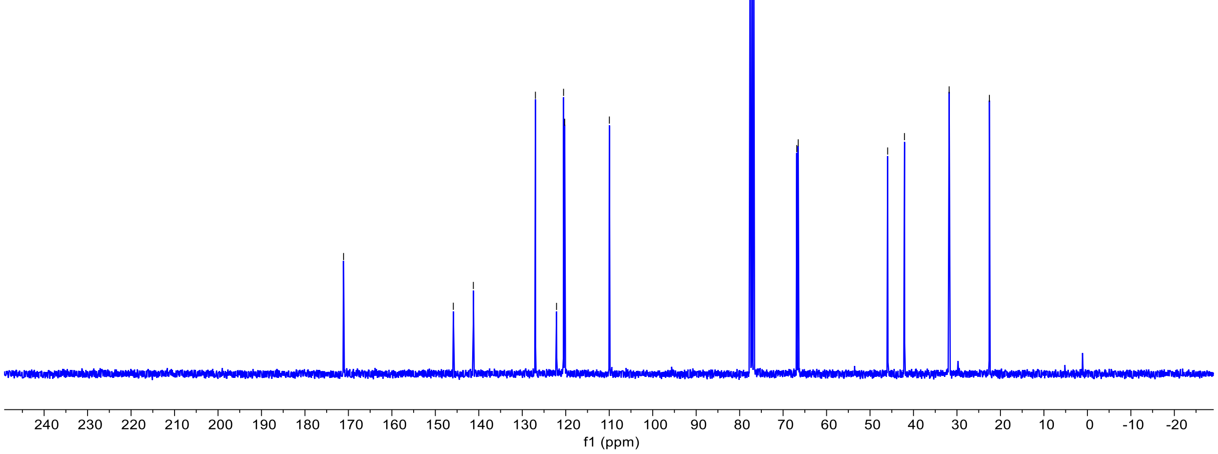

1-Morpholino-3-(1-tosyl-1*H*-benzo[*d*]imidazol-2-yl)propan-1-one Ts-**3z**

^{1}H NMR (400 MHz, $CDCl_3$)

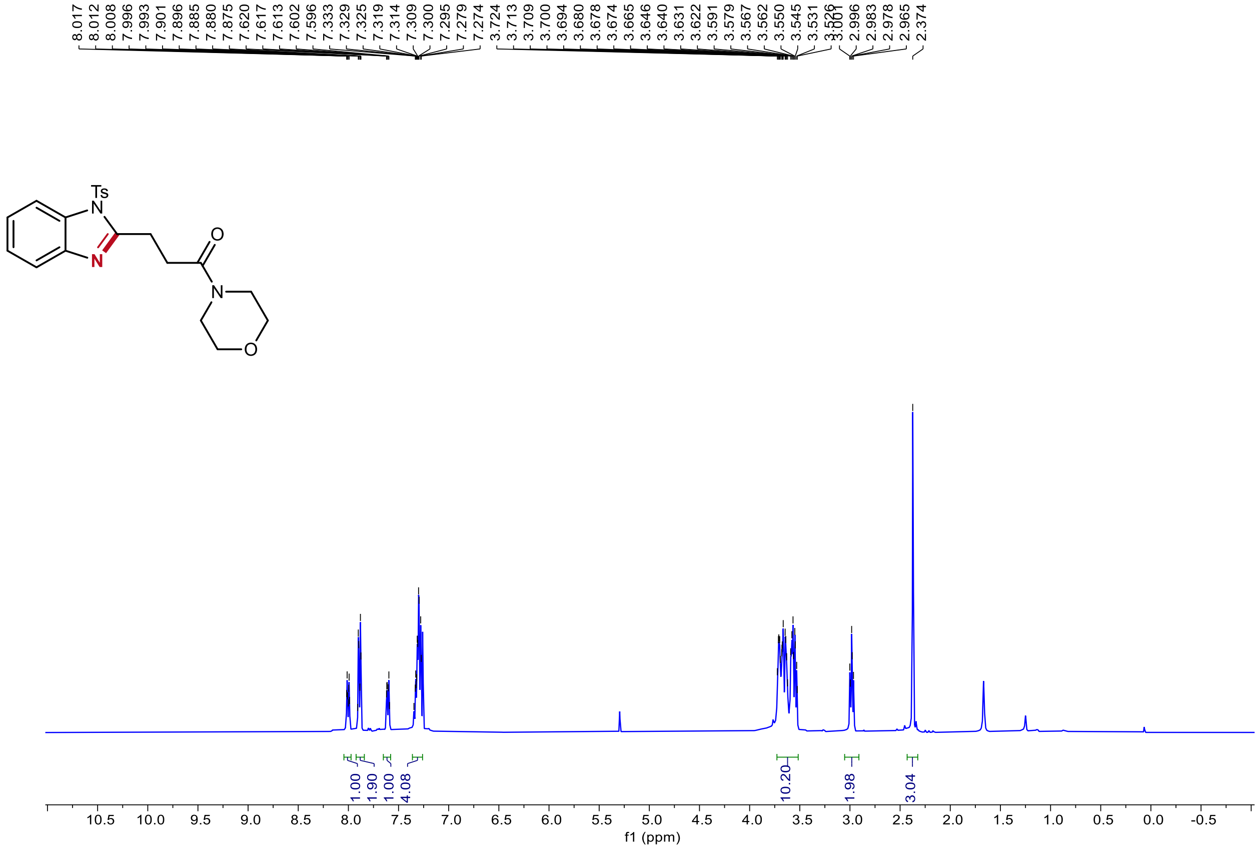

^{13}C NMR (101 MHz, $CDCl_3$)

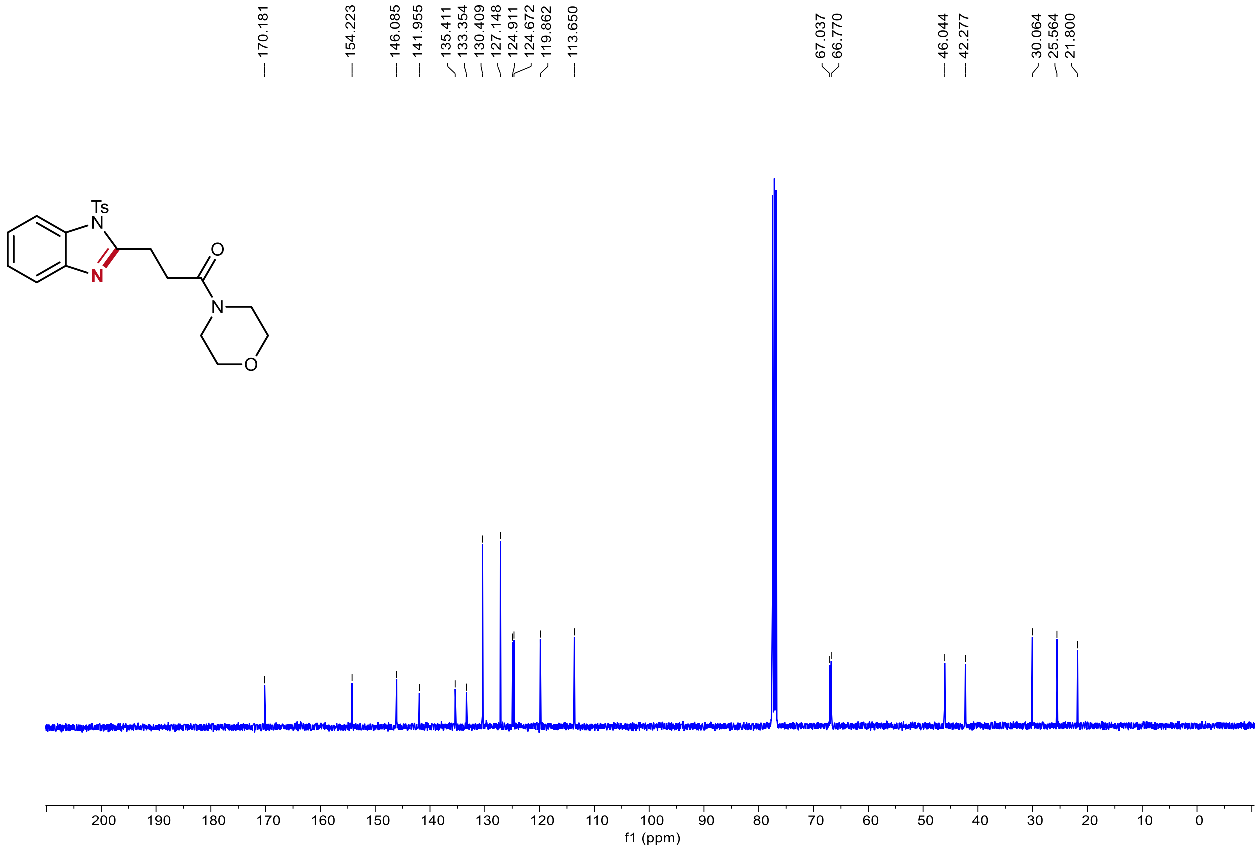

2-((3-isopropyl-1*H*-indazol-4-yl)oxy)-*N*-(pyridin-2-ylmethyl)acetamide 2aa

^{1}**H NMR** (300 MHz, MeOD)

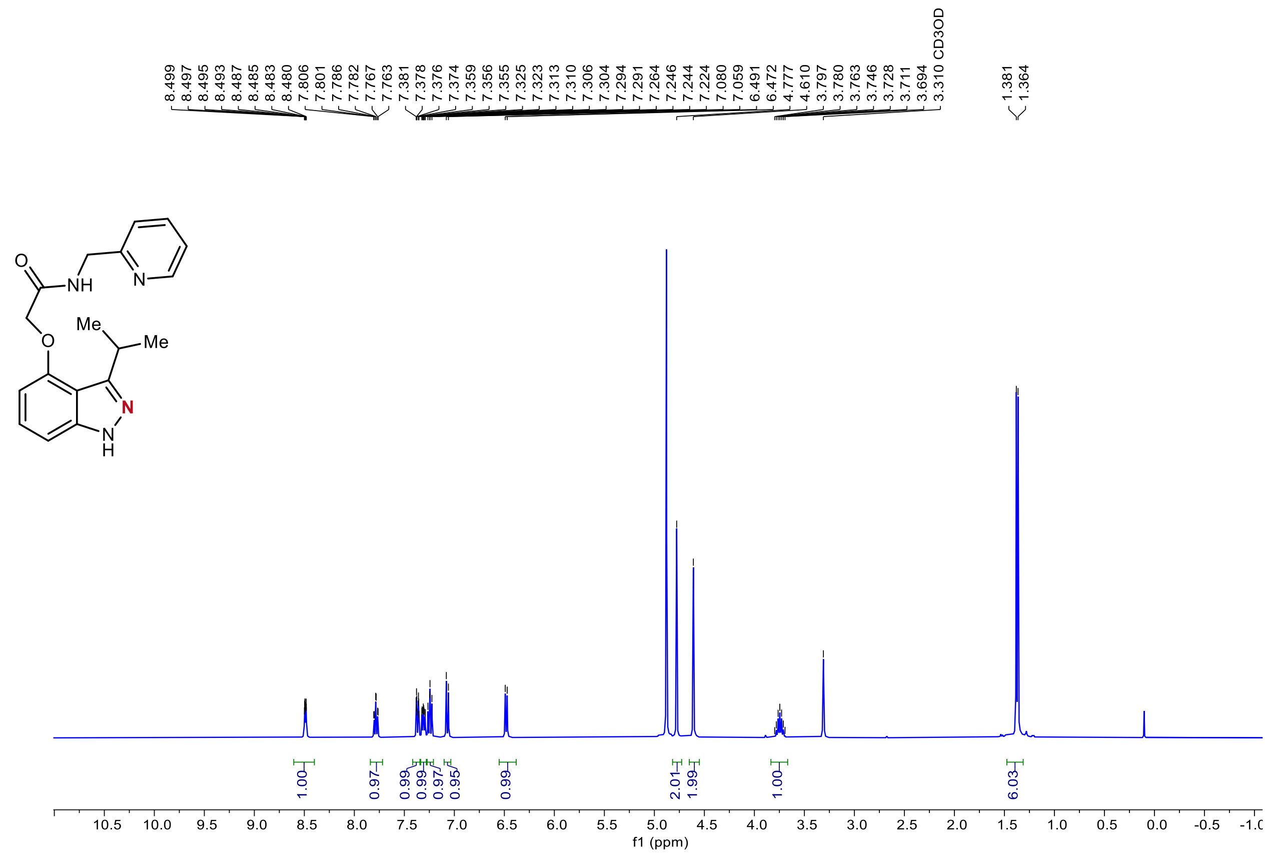

^{13}**C NMR** (76 MHz, MeOD)

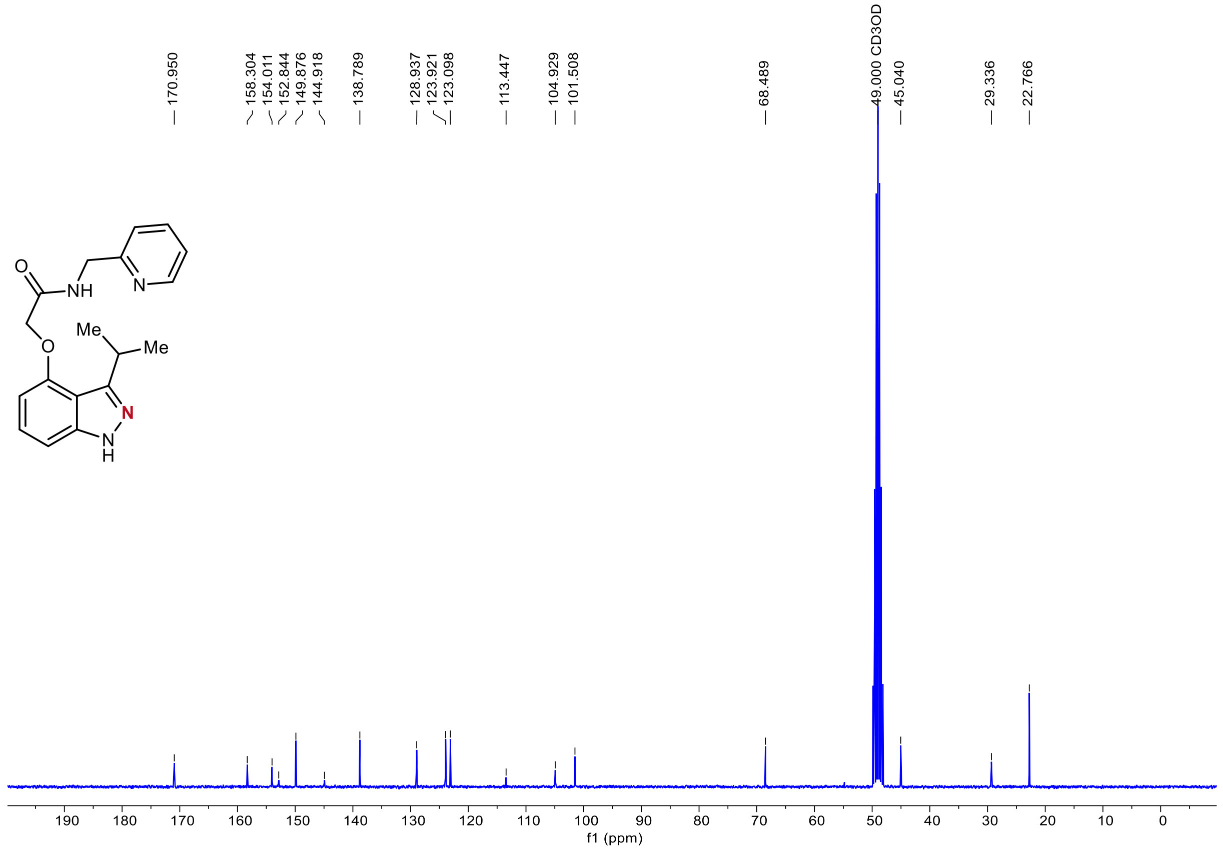

5-(1*H*-Indazol-3-yl)-2-methyloxazole 2ab

^{1}H NMR (400 MHz, MeOD)

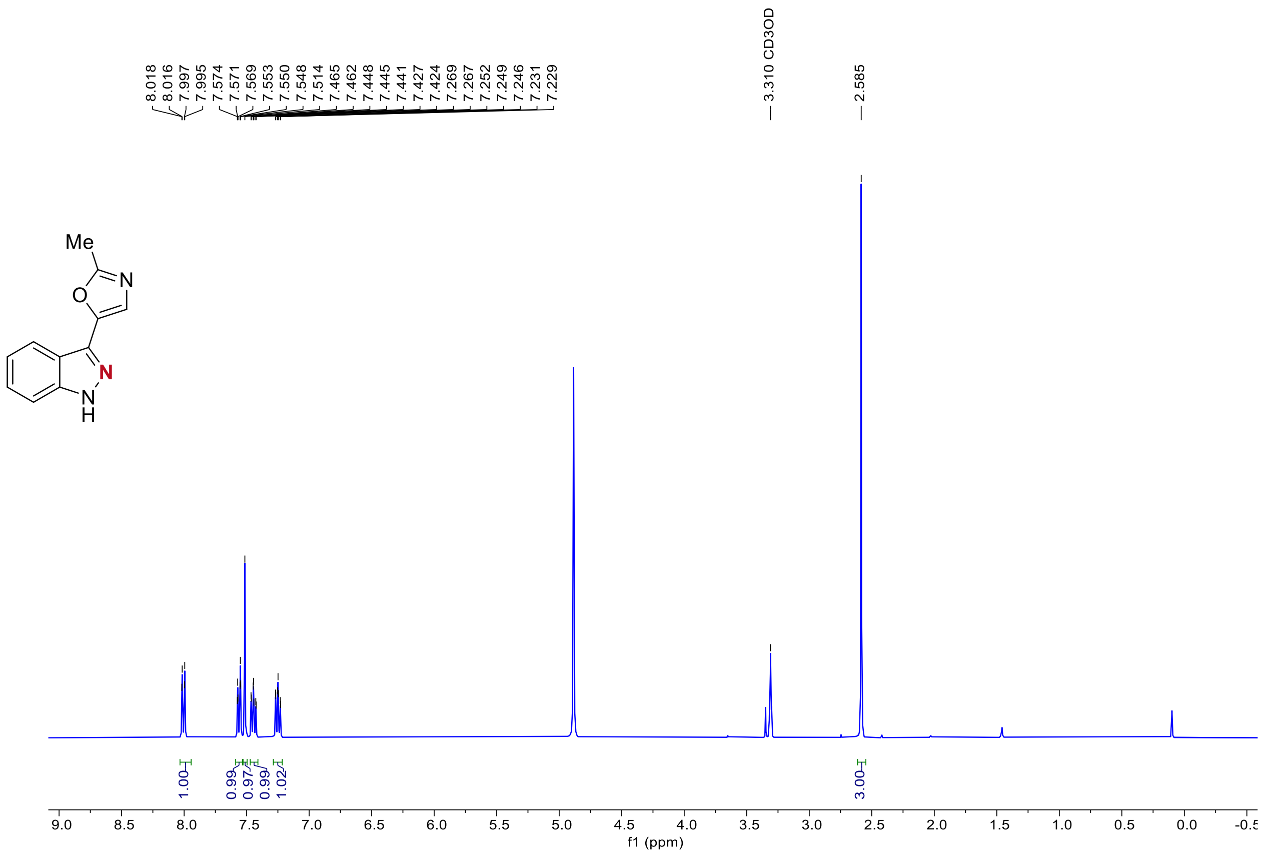

^{13}C NMR (101 MHz, MeOD)

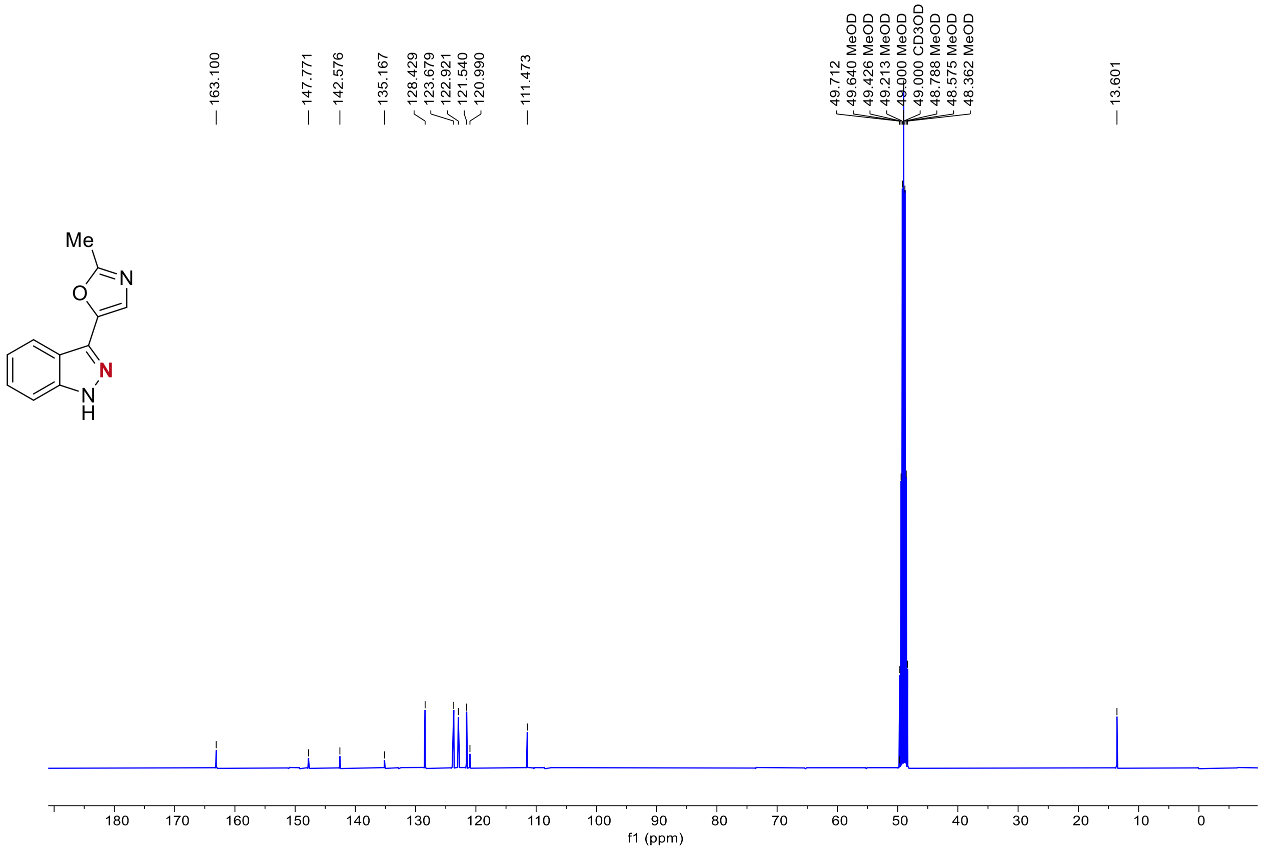

Methyl 2-(1,9-diethyl-10-oxo-7,8,9,10-tetrahydro-6H-benzo[4,5]imidazo[1,2-*a*]azepin-9-yl)acetate 3ac

[1]**H NMR** (400 MHz, $CDCl_3$)

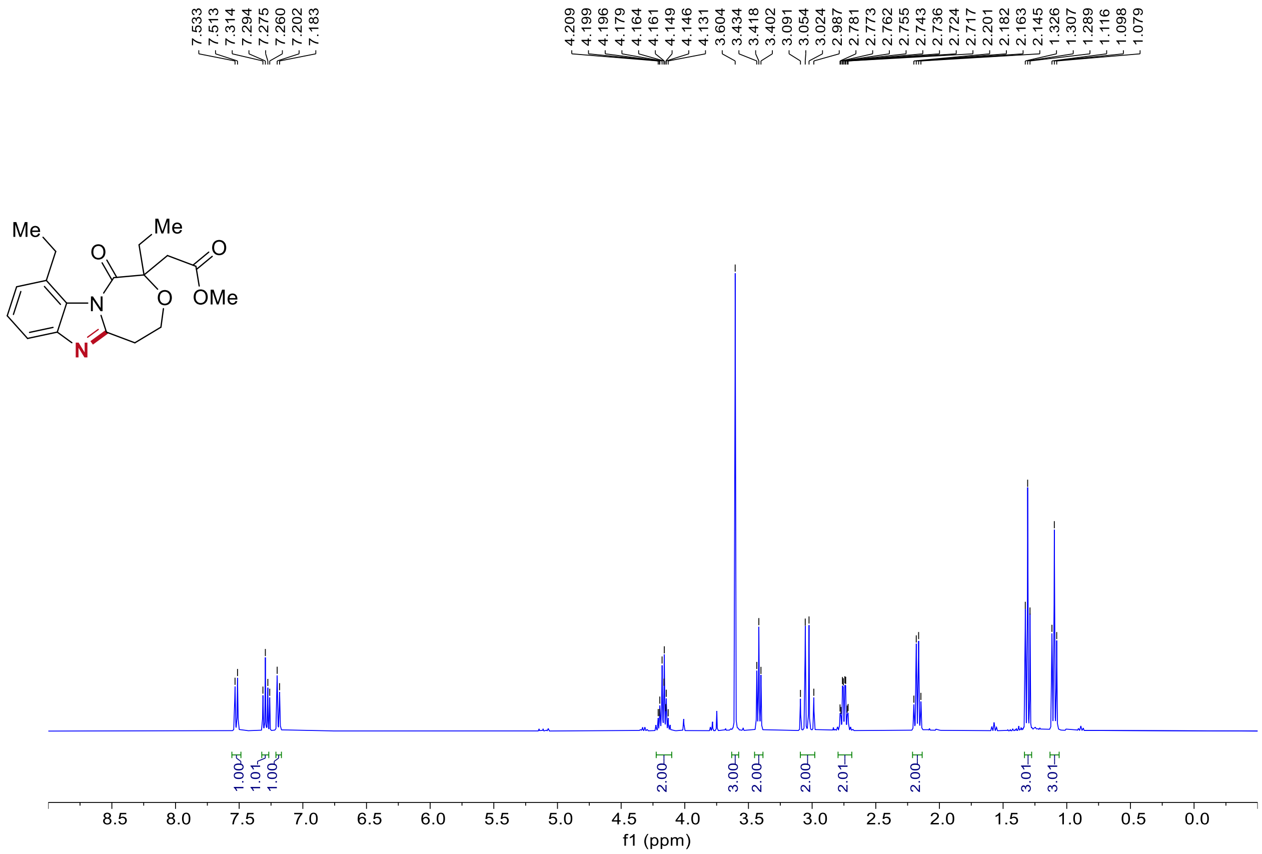

[13]**C NMR** (100 MHz, $CDCl_3$)

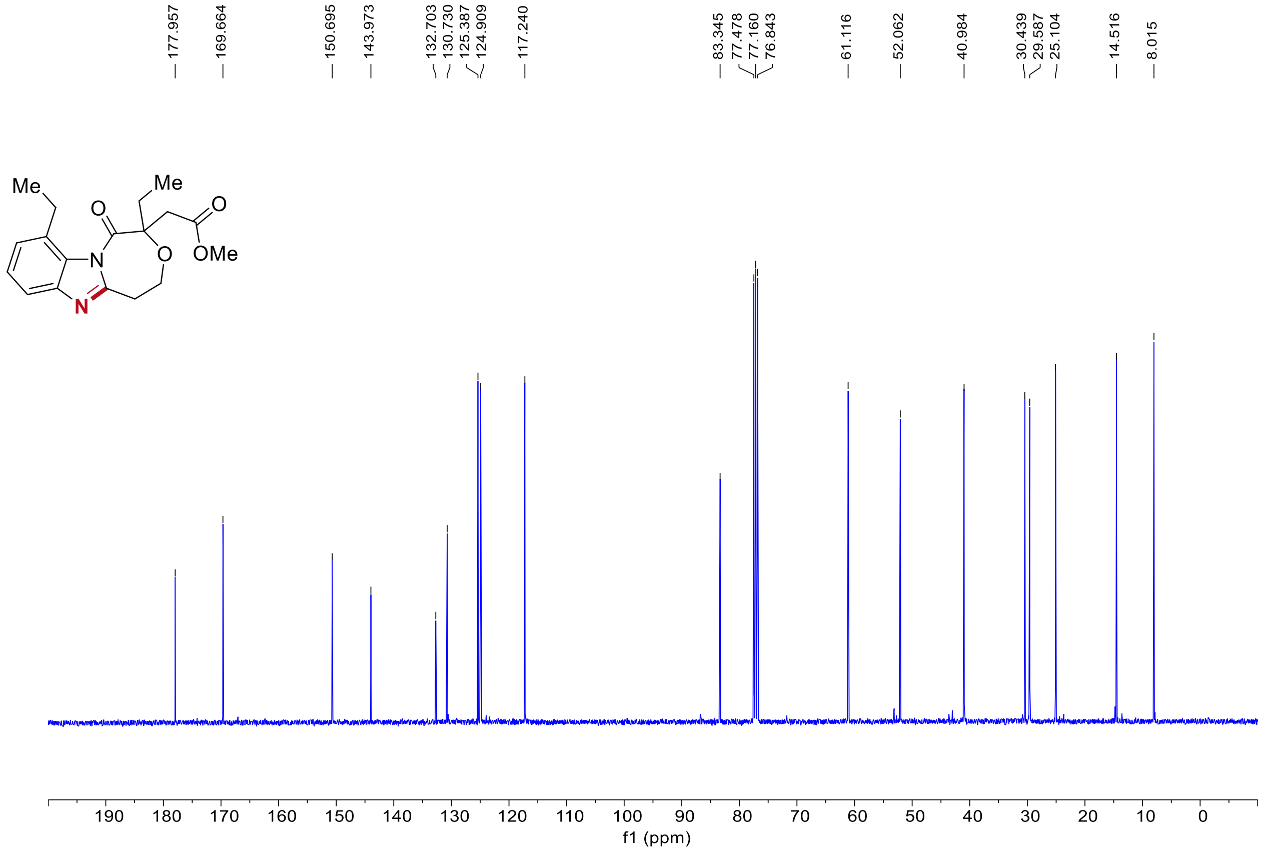

2-((Pyridin-4-ylthio)methyl)benzo[*d*]oxazole 6p

^{1}H NMR (400 MHz, $CDCl_3$)

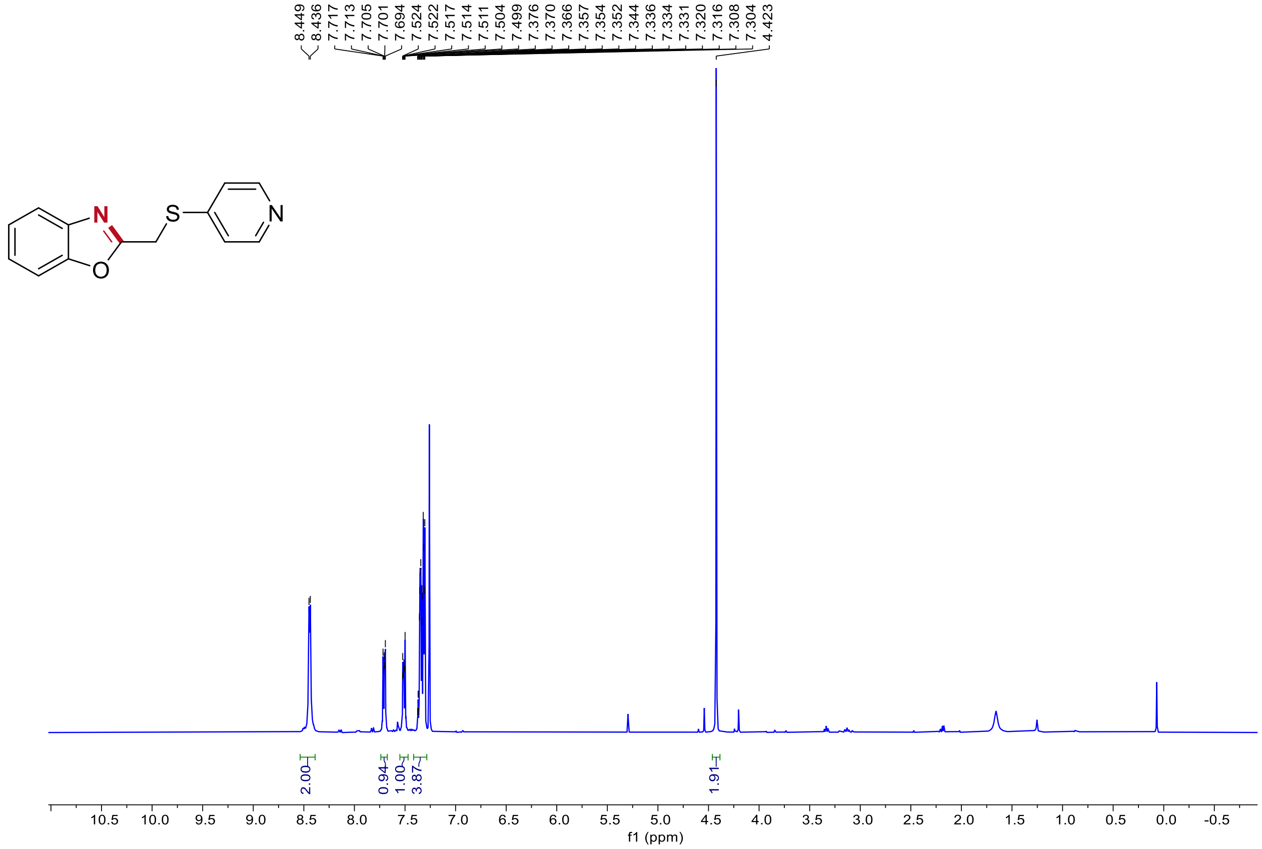

^{13}C NMR (101 MHz, $CDCl_3$)

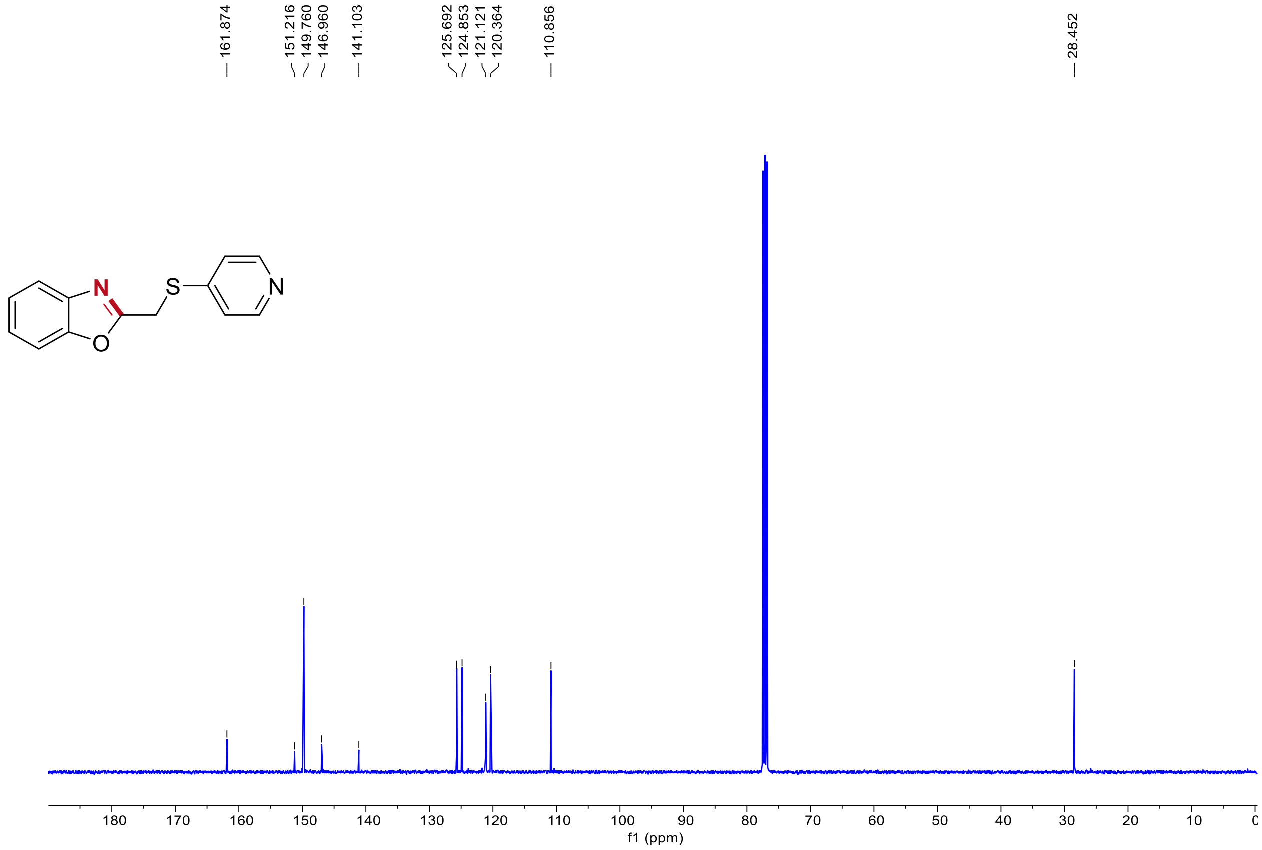

7,10-Dimethyl-1,2,3,4-tetrahydro-5*H*-benzo[3,4]chromeno[6,7-*d*]isoxazol-5-one 5q

^{1}H NMR (400 MHz, $CDCl_3$)

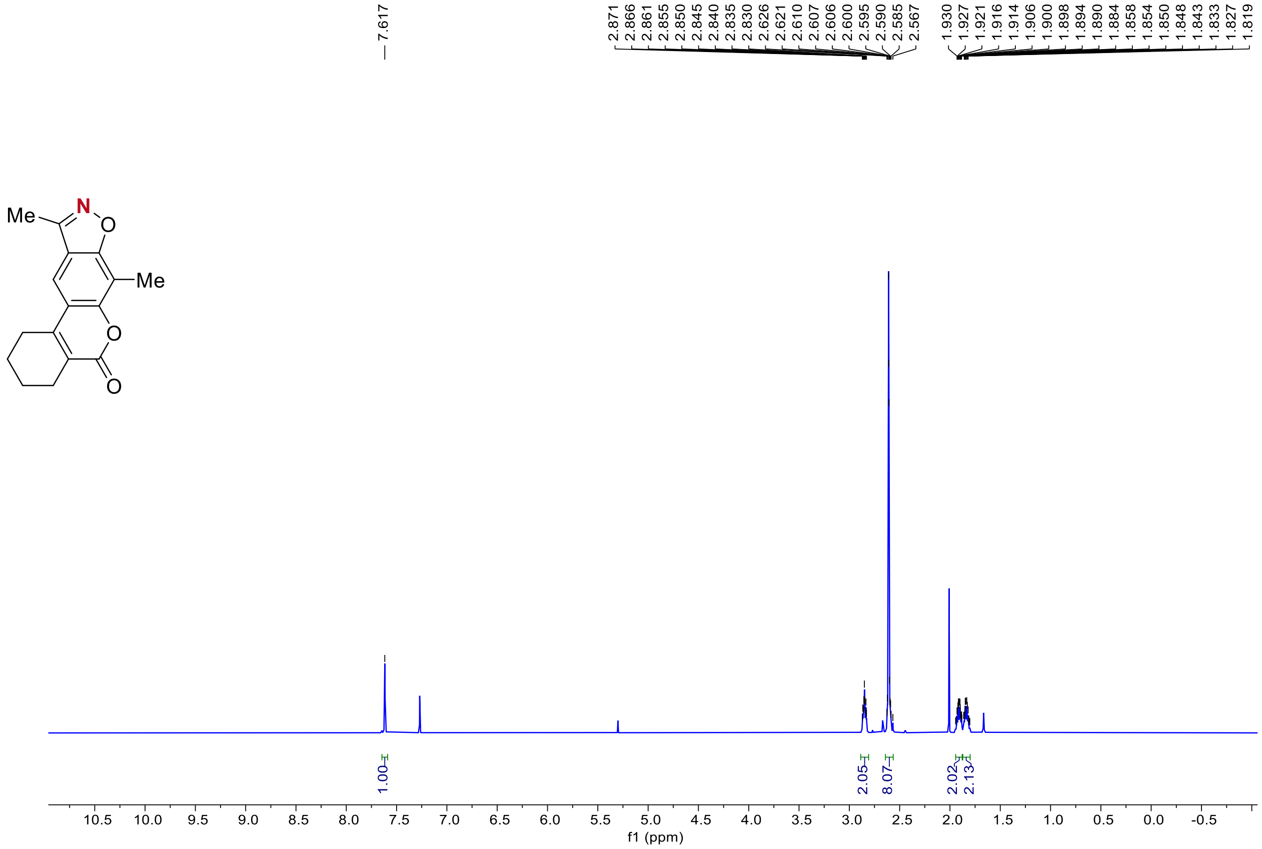

^{13}C NMR (101 MHz, $CDCl_3$)

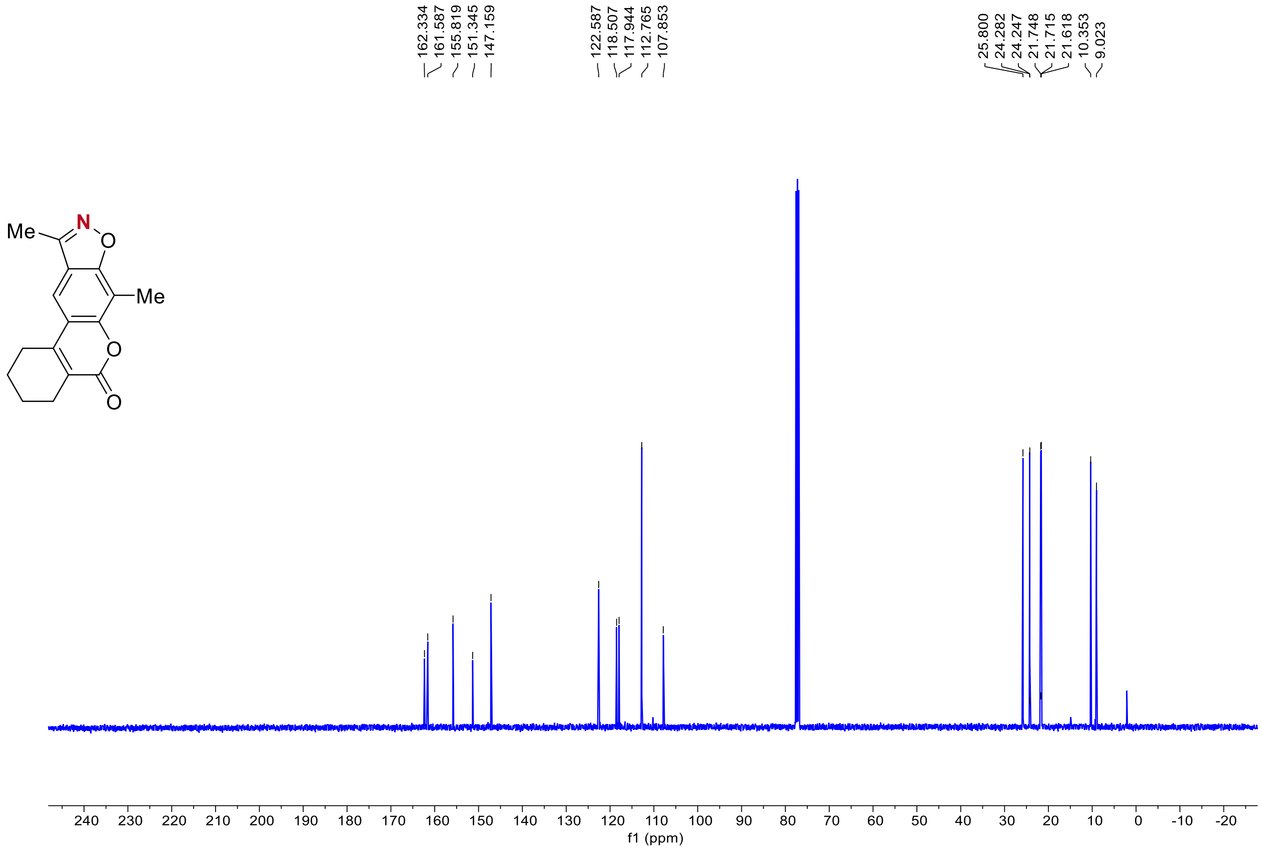

7,9-Dimethyl-1,2,3,4-tetrahydro-5*H*-benzo[3,4]chromeno[6,7-*d*]oxazol-5-one 6q

^{1}H NMR (400 MHz, $CDCl_3$)

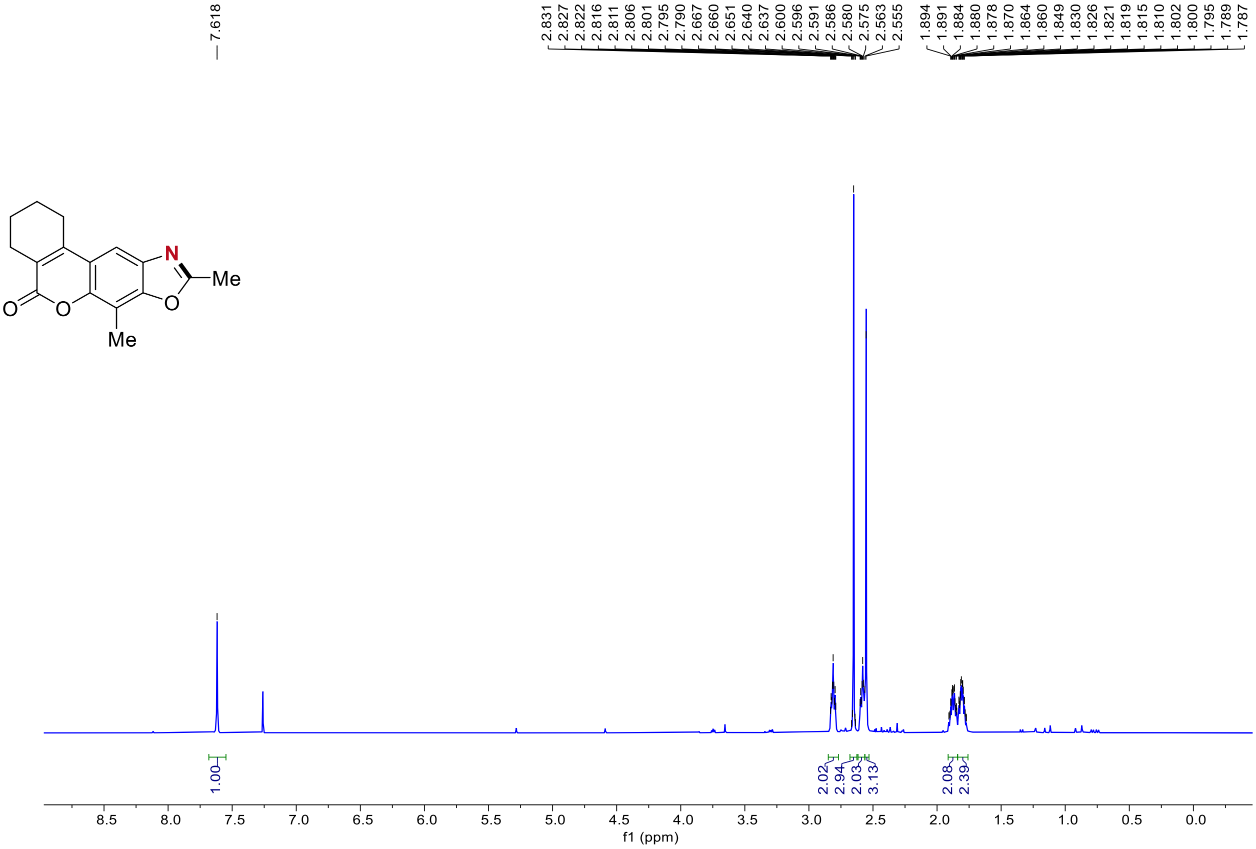

^{13}C NMR (101 MHz, $CDCl_3$)

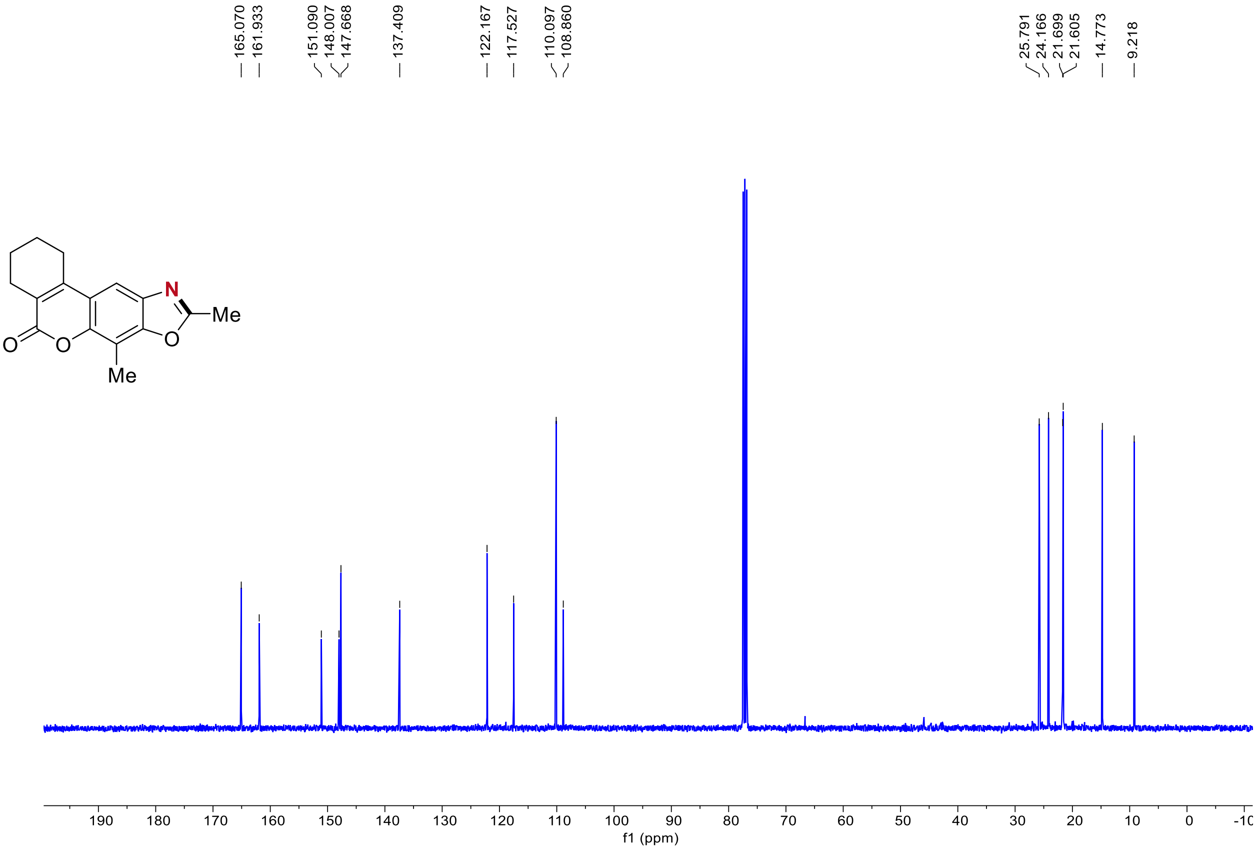

3-Methylbenzo[*d*]isoxazol-5-yl 3-(4,5-diphenyloxazol-2-yl)propanoate 5r

^{1}H NMR (400 MHz, $CDCl_3$)

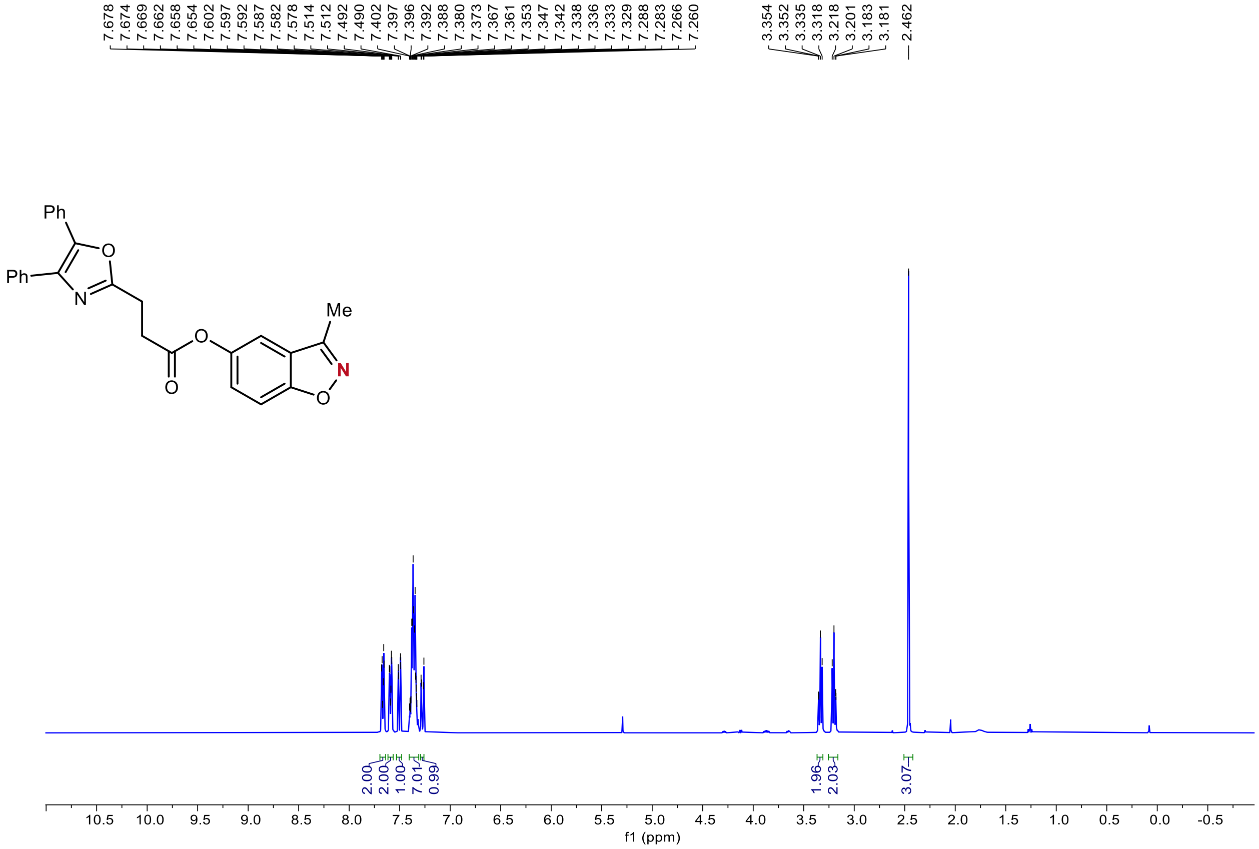

^{13}C NMR (101 MHz, $CDCl_3$)

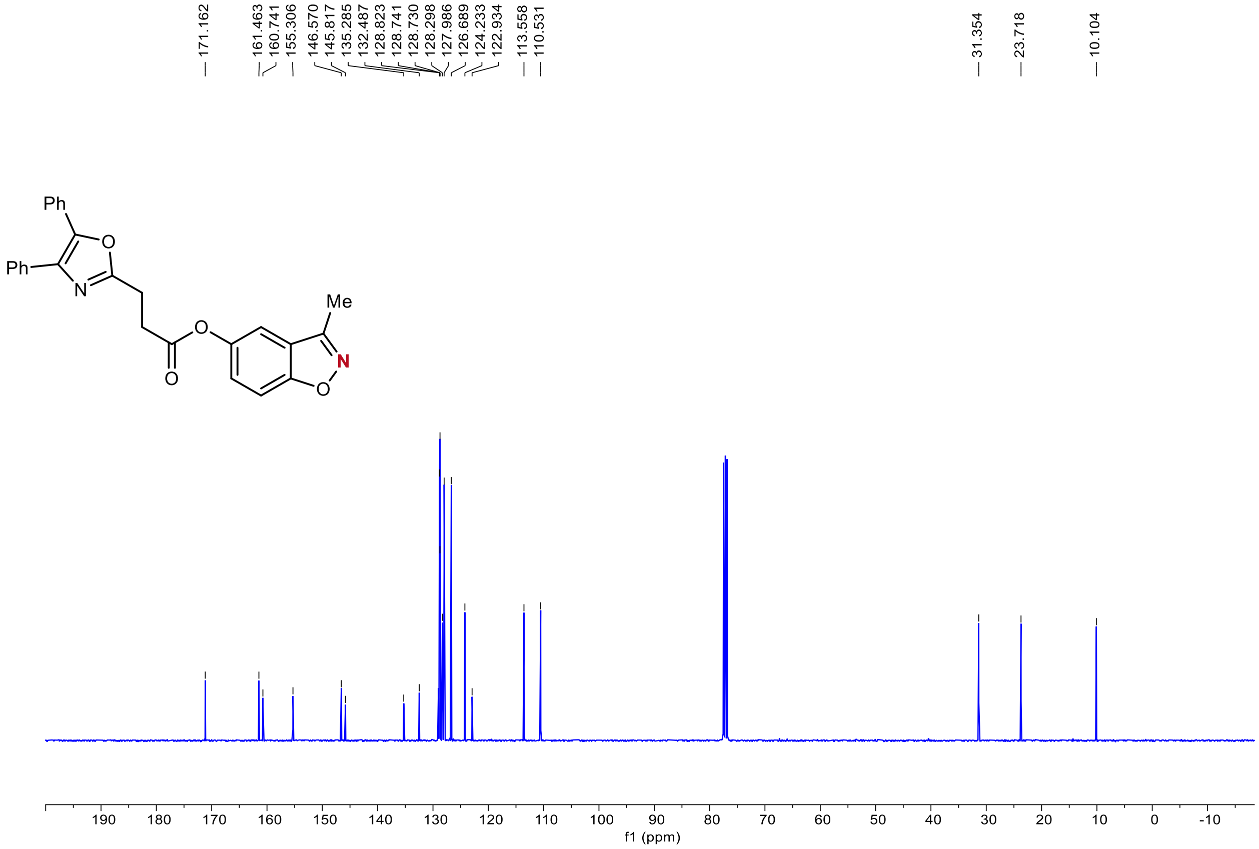

2-Methylbenzo[*d*]oxazol-5-yl 3-(4,5-diphenyloxazol-2-yl)propanoate 6r

^{1}H NMR (300 MHz, $CDCl_3$)

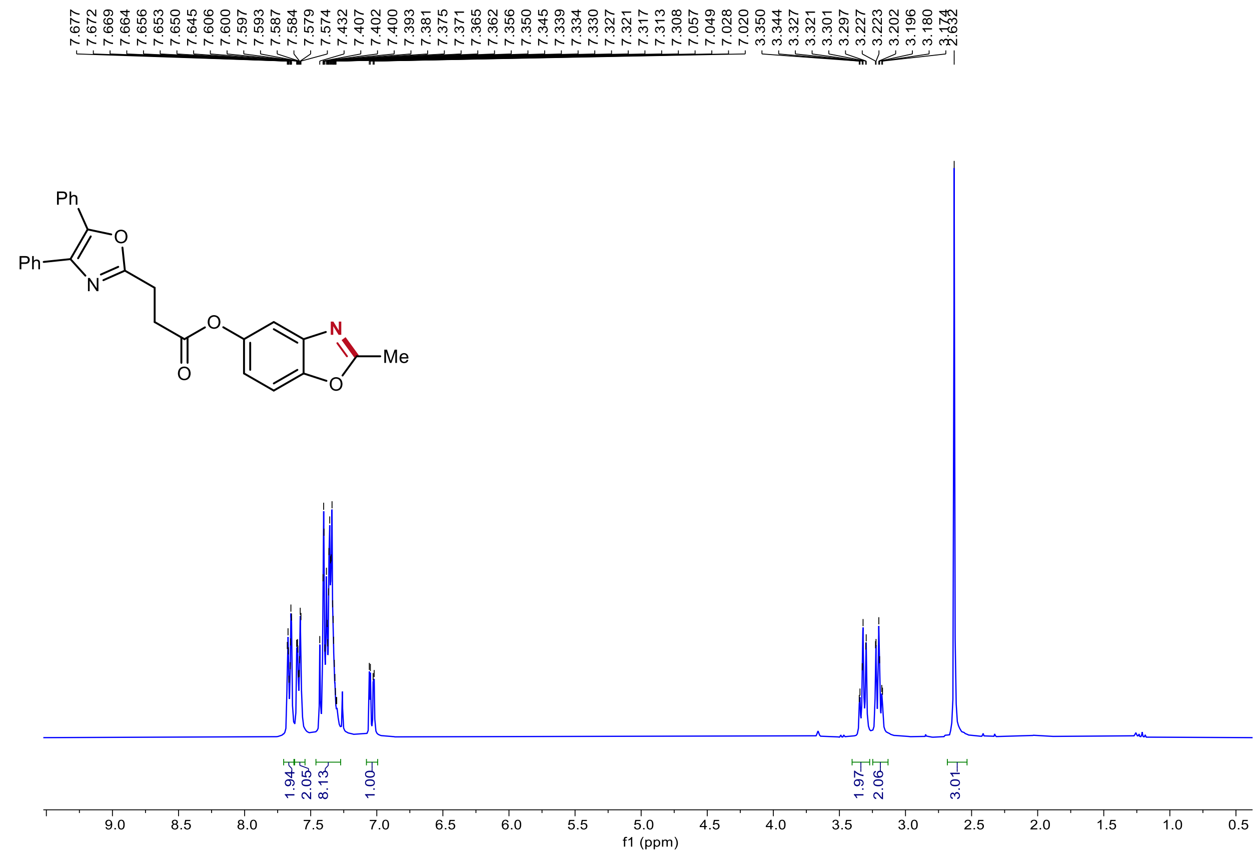

^{13}C NMR (76 MHz, $CDCl_3$)

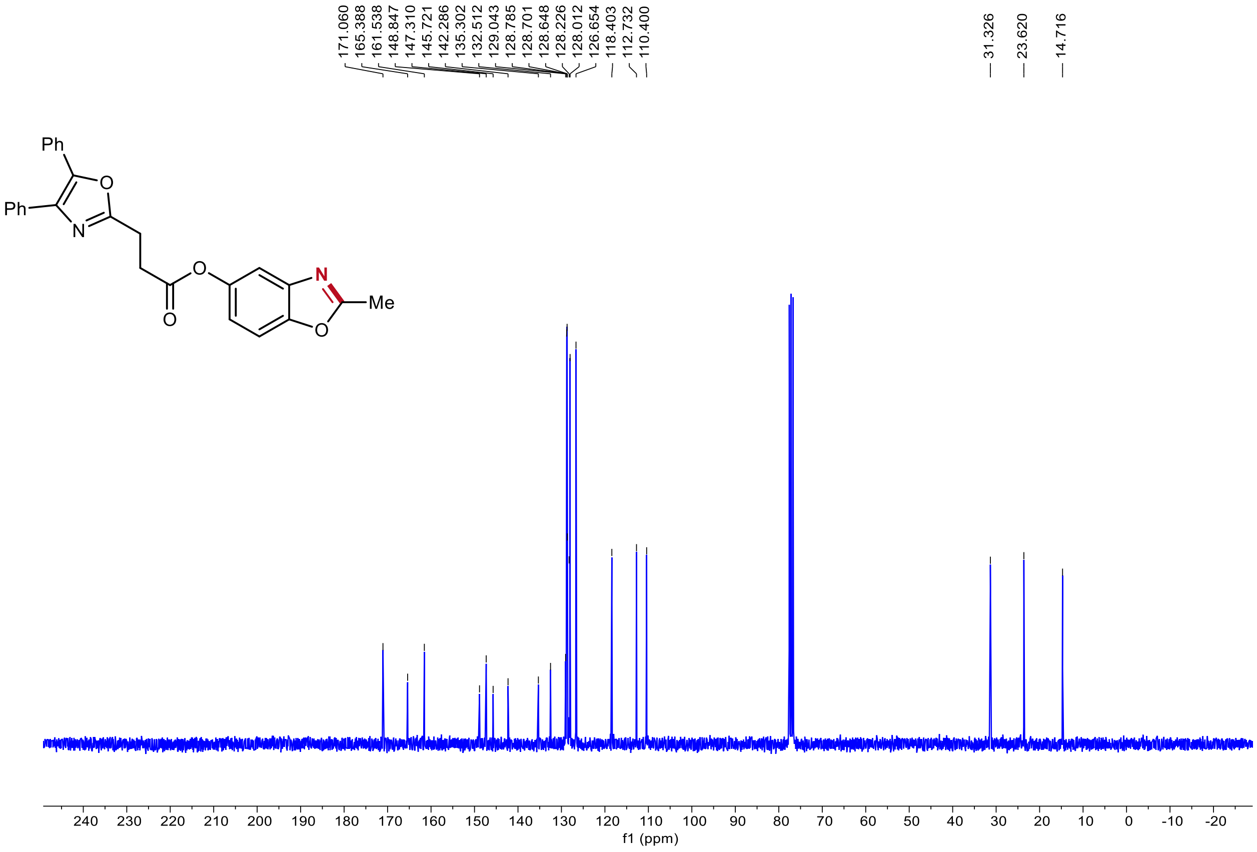

3-Methylbenzo[*d*]isoxazol-5-yl 2-(1-(4-chlorobenzoyl)-5-methoxy-2-methyl-1H-indol-3-yl)acetate 5s

^{1}H NMR (300 MHz, $CDCl_3$)

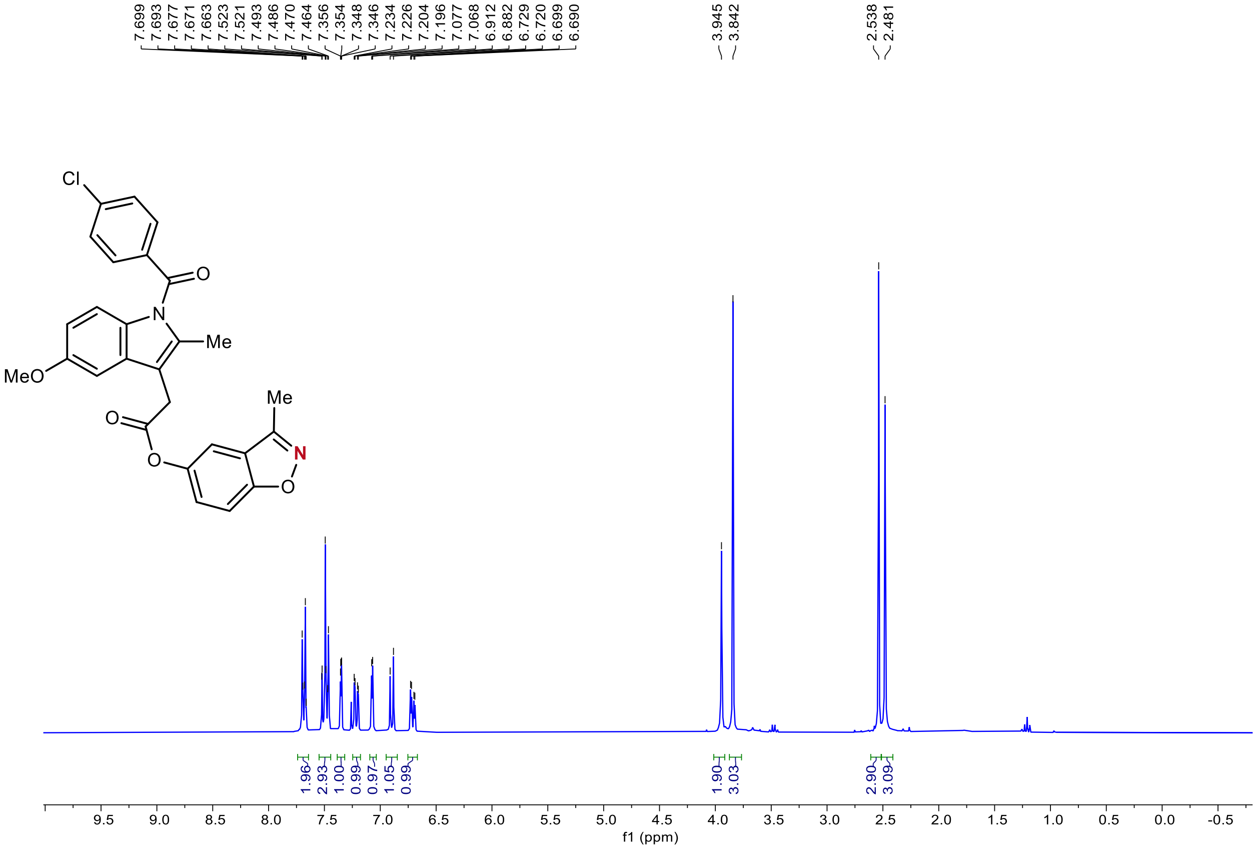

^{13}C NMR (76 MHz, $CDCl_3$)

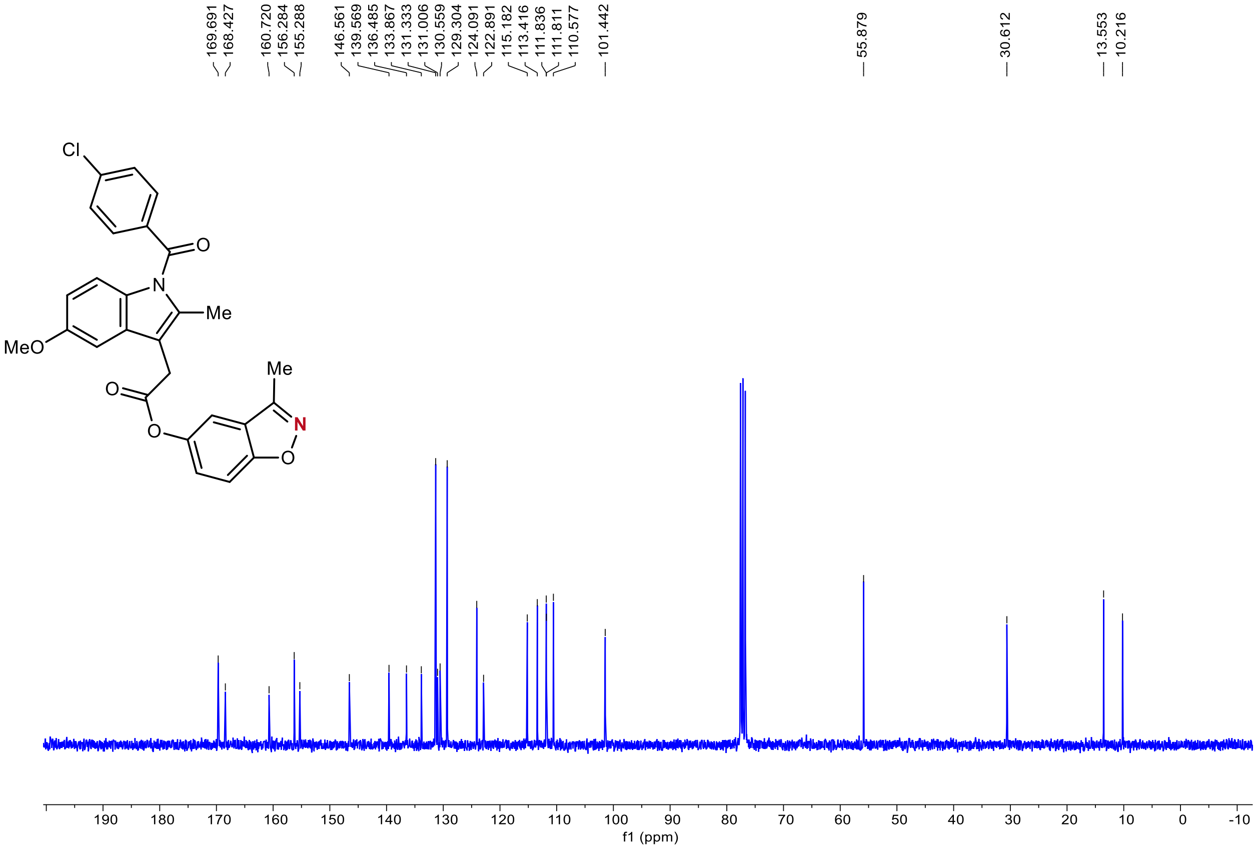

2-Methylbenzo[*d*]oxazol-5-yl 2-(1-(4-chlorobenzoyl)-5-methoxy-2-methyl-1H-indol-3-yl)acetate 6s

^{1}**H NMR** (400 MHz, $CDCl_3$)

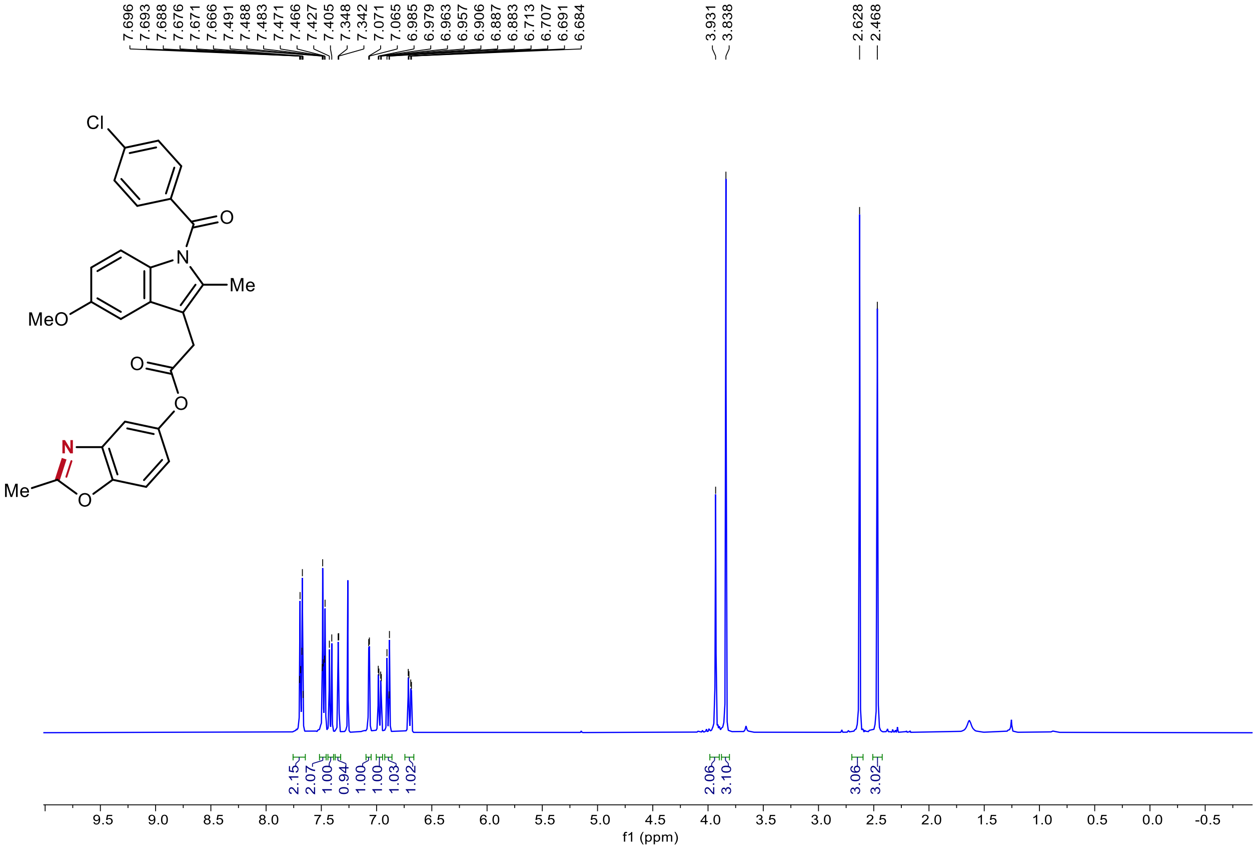

^{13}**C NMR** (76 MHz, $CDCl_3$)

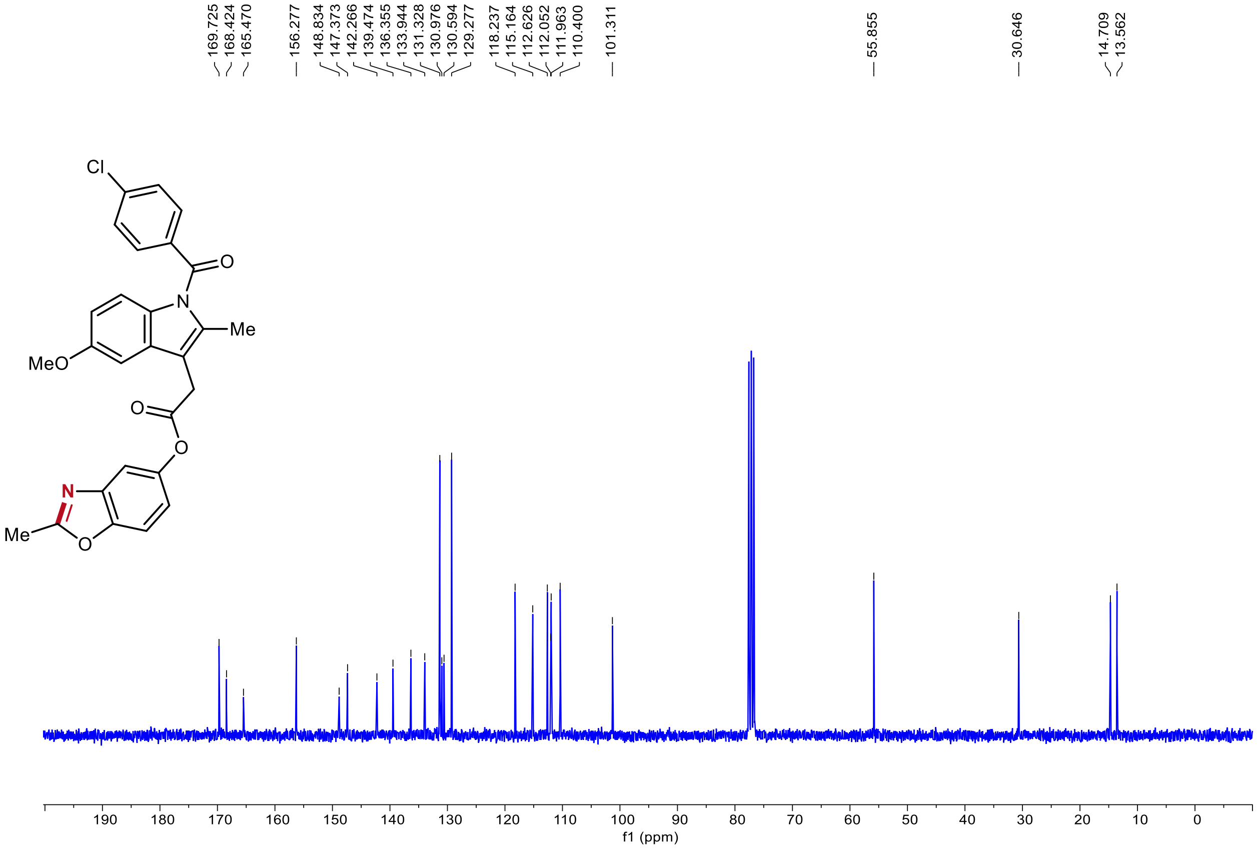

Methyl 3-methyl-1*H*-indazole-1-carboxylate-2-^{15}N Moc-^{15}N-**2a**

^{1}**H NMR** (300 MHz, $CDCl_3$)

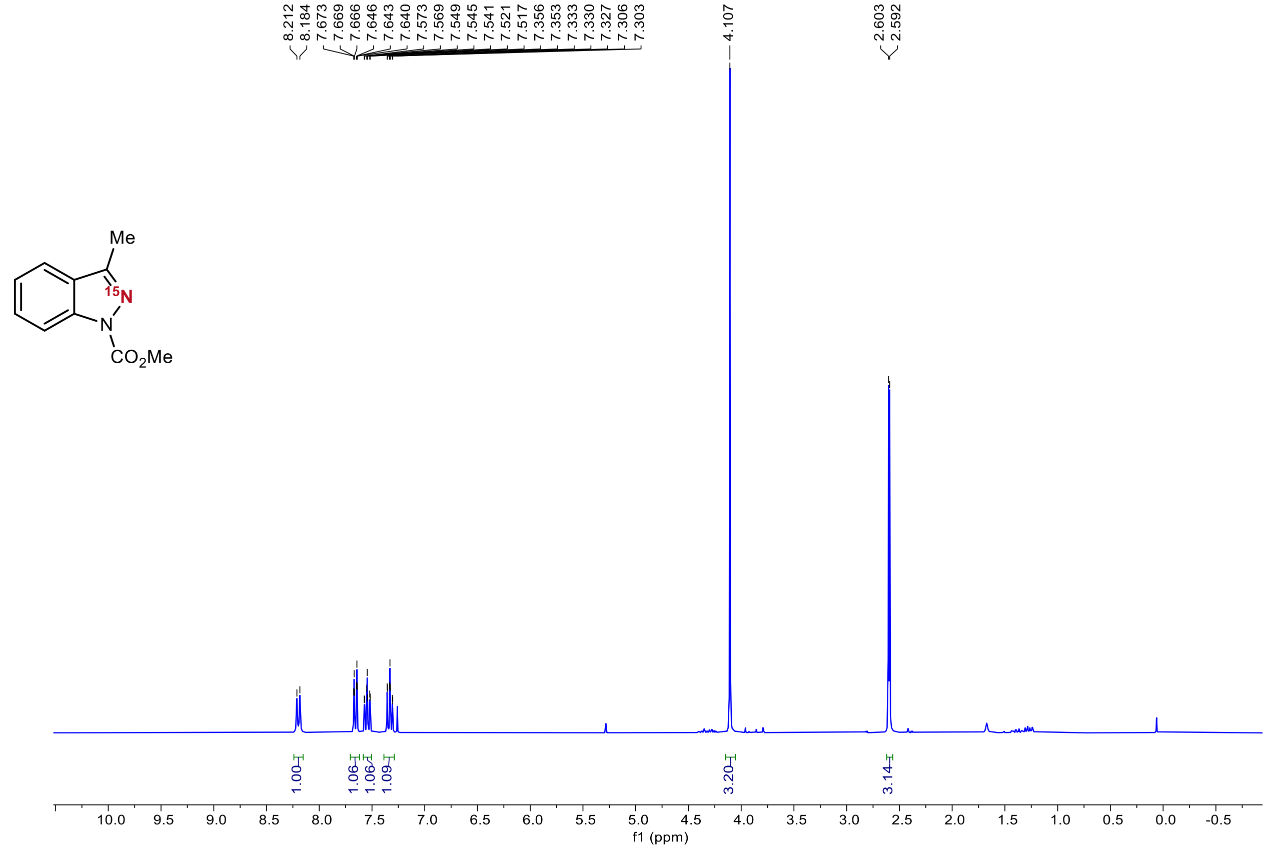

^{13}**C NMR** (76 MHz, $CDCl_3$)

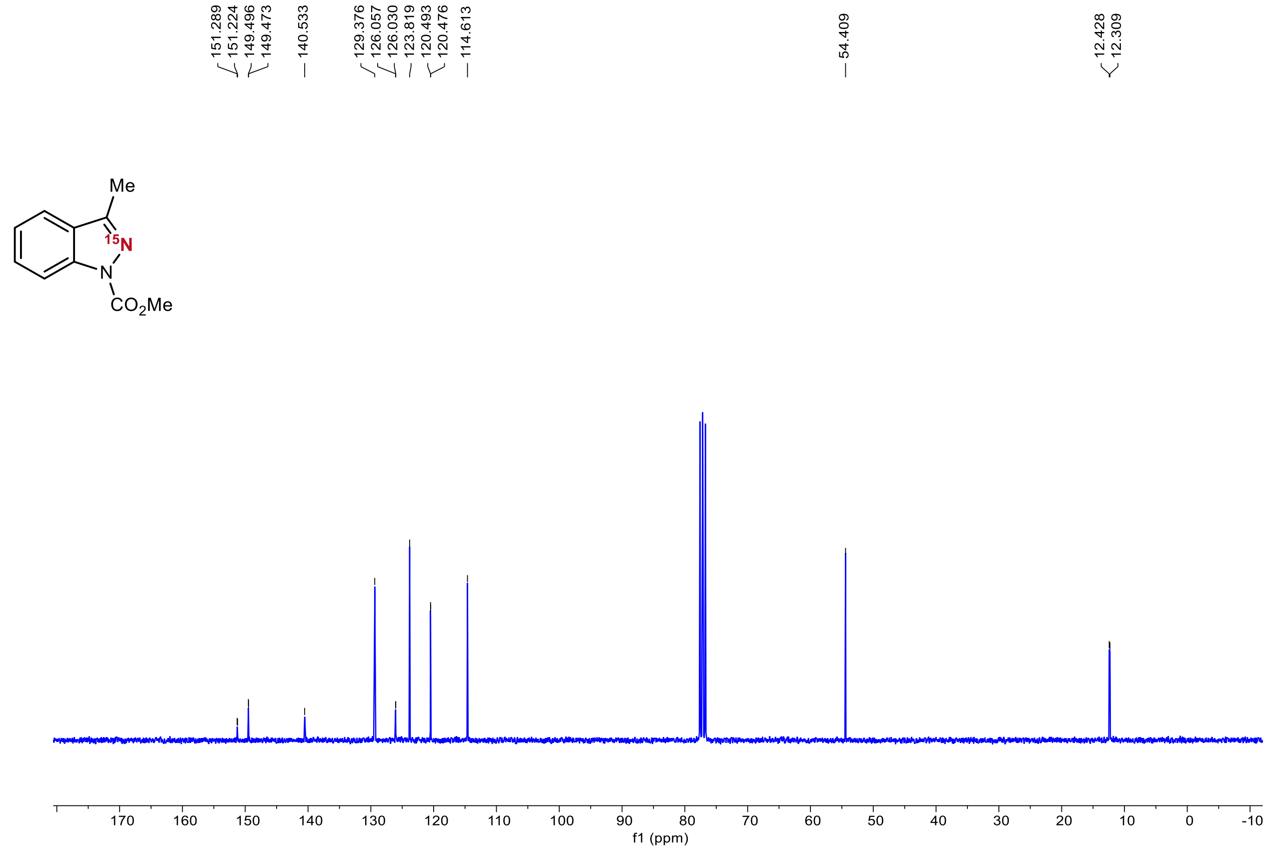

Dimethyl 3-methyl-1*H*-indazole-1,5-dicarboxylate-^{15}N Moc-^{15}N-**2ad**

^{1}H NMR (599 MHz, $CDCl_3$)

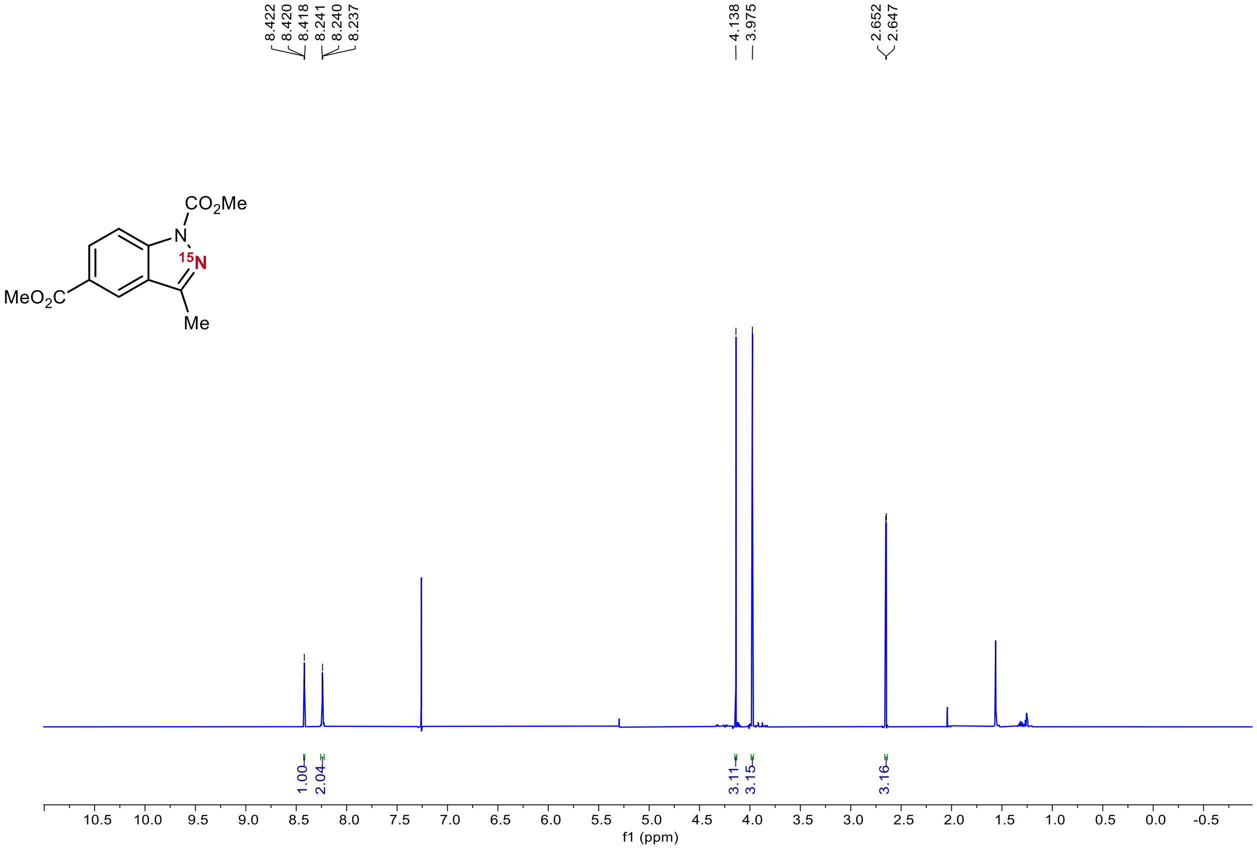

^{13}C NMR (151 MHz, $CDCl_3$)

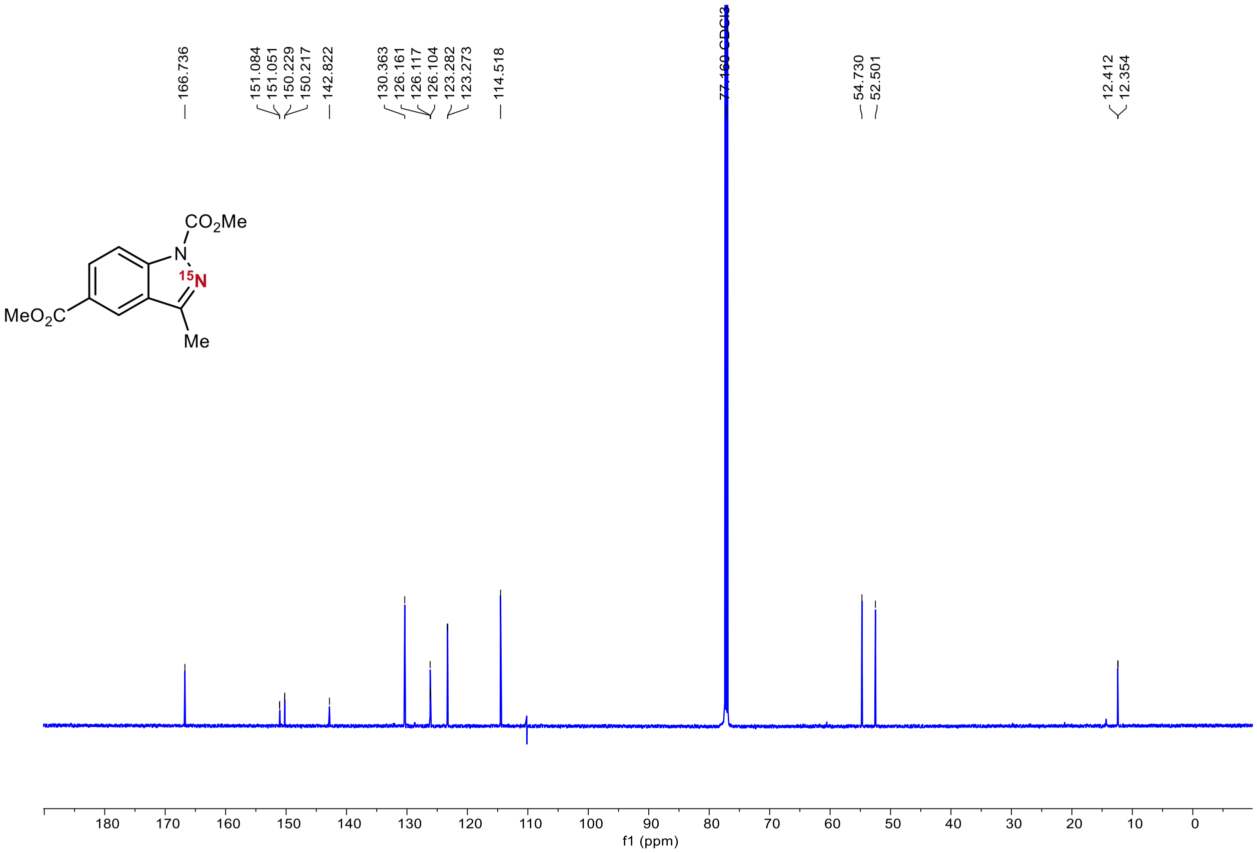

^{15}N NMR (61 MHz, $CDCl_3$)

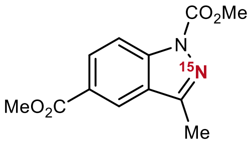

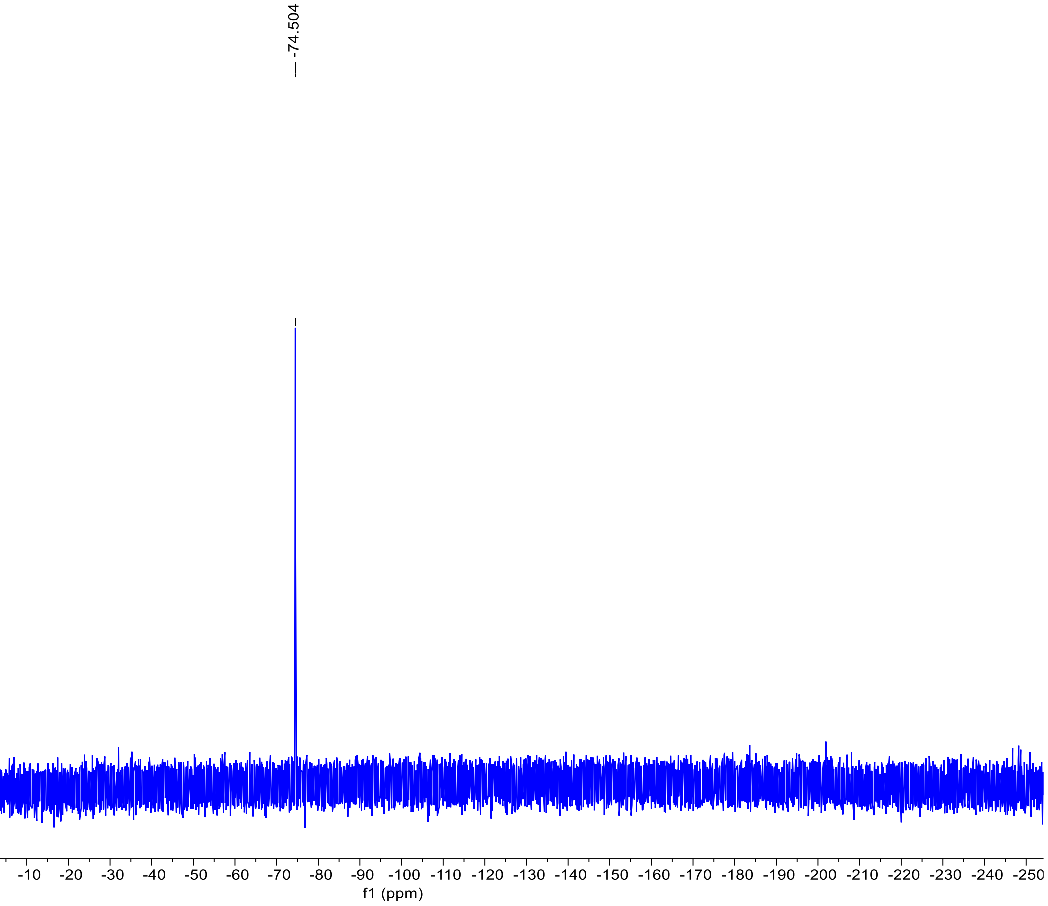

Supplement: Supplementary file 1 — This Supplementary Information file contains the following sections: 1. General information; 2. Preparation of starting materials; 3. Detailed optimization of reaction conditions; 4. Reactions with oxime intermediates; 5. General procedure; 6. Spectral data of products; 7. X-ray crystal structure analysis of 2aa, 5r and 6s; 8. References and 9. NMR spectra of products. [file 41586_2025_9019_MOESM1_ESM.pdf]
